# Genetic heterogeneity and evolutionary history of high-grade ovarian carcinoma and matched distant metastases

**DOI:** 10.1038/s41416-020-0763-4

**Published:** 2020-02-26

**Authors:** Tariq Masoodi, Sarah Siraj, Abdul K. Siraj, Saud Azam, Zeeshan Qadri, Sandeep K. Parvathareddy, Asma Tulbah, Fouad Al-Dayel, Hamed AlHusaini, Osama AlOmar, Ismail A. Al-Badawi, Fowzan S. Alkuraya, Khawla S. Al-Kuraya

**Affiliations:** 1 Human Cancer Genomic Research, King Faisal Specialist Hospital and Research Centre, P.O. Box 3354, Riyadh, 11211 Saudi Arabia; 2 Department of Pathology, King Faisal Specialist Hospital and Research Centre, P.O. Box 3354, Riyadh, 11211 Saudi Arabia; 3 Department of Medical Oncology, King Faisal Specialist Hospital and Research Centre, P.O. Box 3354, Riyadh, 11211 Saudi Arabia; 4 Department of Obstetrics and Gynaecology, King Faisal Specialist Hospital and Research Centre, P.O. Box 3354, Riyadh, 11211 Saudi Arabia; 5 Department of Genetics, King Faisal Specialist Hospital and Research Centre, P.O. Box 3354, Riyadh, 11211 Saudi Arabia; 6 Department of Anatomy and Cell Biology, College of Medicine, Alfaisal University, Riyadh, Saudi Arabia

**Keywords:** Cancer genomics, Ovarian cancer

## Abstract

**Background:**

High-grade serous ovarian carcinoma (HGSOC) is the most frequent type of ovarian carcinoma, associated with poor clinical outcome and metastatic disease. Although metastatic processes are becoming more understandable, the genomic landscape and metastatic progression in HGSOC has not been elucidated.

**Methods:**

Multi-region whole-exome sequencing was performed on HGSOC primary tumours and their metastases (*n* = 33 tumour regions) from six patients. The resulting somatic variants were analysed to delineate tumour evolution and metastatic dissemination, and to compare the repertoire of events between primary HGSOC and metastasis.

**Results:**

All cases presented branching evolution patterns in primary HGSOC, with three cases further showing parallel evolution in which different mutations on separate branches of a phylogenetic tree converge on the same gene. Furthermore, linear metastatic progression was observed in 67% of cases with late dissemination, in which the metastatic tumour mostly acquires the same mutational process active in primary tumour, and parallel metastatic progression, with early dissemination in the remaining 33.3% of cases. Metastatic-specific SNVs were further confirmed as late dissemination events. We also found the involvement of metastatic-specific driver events in the Wnt/β-catenin pathway, and identified potential clinically actionable events in individual patients of the metastatic HGSOC cohort.

**Conclusions:**

This study provides deeper insights into clonal evolution and mutational processes that can pave the way to new therapeutic targets.

## Background

High-grade serous ovarian cancer (HGSOC) is the most frequent and leading cause of death from gynaecologic cancers,^1^ with a 10-year survival of <30%, despite efforts over the last three decades to change the outlook of this highly lethal disease.^2^ HGSOC is a heterogeneous cancer with a high rate of relapse and metastasis. HGSOC is typically diagnosed at advanced stages after dispersing to multiple sites, ensuing exceptionally poor prognosis with ~80% of patients relapsing despite initial responses to surgery and chemotherapy,^3^ of which many relent to treatment-resistant disease.^4^

As most cancer-related deaths are due to metastatic disease, prevention of metastasis is of great clinical priority, currently achieved via earlier ablative surgery with systemic neoadjuvant or adjuvant therapy.^5^ Due to the lack of distinct anatomical barriers in the peritoneal cavity, HGSOC metastases characteristically result in early and widespread disease at distal peritoneal sites, with exfoliating HGSOC cells transported via the physiological peritoneal fluid, and disseminating within the abdominal cavity.^6^

In 66% of cases, HGSOC occurs bilaterally and often synchronously, affecting both ovaries.^7^ However, origins and relatedness of the HGSOC tumours present in each ovary remain unclear. It is debated whether they are independent primary tumours from multifocal oncogenesis, arising spontaneously from a similar genetic background, clonally related due to tumorigenesis initiating from one ovary and then metastasising to the contralateral ovary, or two metastases.^8^ Although some studies have revealed clonal relationships between the two tumours in patients,^9,10^ limited information is available on the genomic landscape spurring both bilateral and unilateral HGSOC evolution and metastatic processes.

Deep spatial and longitudinal sequencing studies of primary tumours and matched metastases can successfully establish the history of somatic and genetic events, allowing the dissemination and establishment of the new founding tumour cells at distant sites by increasing metastatic potential.^11,12^ Like tumour evolution, many competing models of metastasis exist, some of which include the linear progression model, where metastases arise from late-occurring advanced clonal subpopulations; parallel progression, suggesting early metastatic seeding and the independent acquisition of primary tumour mutations, with widespread disease at an early time point, and metastasis cross-seeding, where clones from distinct metastatic sites, not present in the primary tumour, can disseminate to a new metastatic site.^13,14^

As various models of tumour evolution and metastatic progression in cancer can have diverse clinical implications for the clinical diagnosis, prognosis and treatment of the patient,^15,16^ such as the need for systemic adjuvant therapy in patients displaying parallel metastatic progression to counteract early systemic spread of tumour cells, understanding these models is of great clinical significance.^17^ Given the low survival and high recurrence rate, there is a potential need to report and understand the metastatic evolution of HGSOC. Next-generation sequencing has identified the landscape and potential therapeutic targets in primary ovarian cancer, and the genome of primary ovarian cancer has been well established.^18^ Far less analysis has been performed on ovarian metastases due to the difficulties in accessing metastatic tissues, particularly in distant metastasis; thereby, a number of important clinical and biological queries remain unanswered.

In this study, to examine the variation in mutational concordance and metastatic progression of HGSOC, we use exome-wide sequence analysis of multiple tumour regions of paired primary and metastatic tumours to reveal the dynamic mutational evolutionary process, similarities and differences between primary HGSOC and its metastatic progression, potentially revealing novel molecular targets or markers, and contributing to decisions in personalised therapy for patients with metastatic disease in HGSOC. We also attempted to identify cancer driver genes or molecular pathways specific to these metastases that might offer the opportunities for personalised therapy for this subset of patients with very poor outcome.

## Methods

### Patients and tumour samples

We collected a total of 33 tumour regions with the corresponding normal, whole blood and metastatic tumour (three spleen, two liver, two contralateral ovary and one brain) from six patients with high-grade serous ovarian carcinoma from King Faisal Specialist Hospital and Research Centre. The clinical characteristics of the ovarian cancer cohort are provided in Table 1.

**Table 1:** Clinical characteristics of HGSOC patients.

S.N.	Sample	Age	Histopathology	pT	pN	pM	Stage	FIGO grade	Family history	Site of metastasis
1	OVA_003	60	Serous	T1	N0	M1	Stage IV	Grade 2	Negative	Spleen
2	OVA_013	49	Serous	T2	N0	M1	Stage IV	Grade 2	Negative	Liver
3	OVA_047	42	Serous	T3	N0	M1	Stage IV	Grade 3	Negative	Spleen
4	OVA_048	48	Serous	T1	N0	M1	Stage III	Grade 3	Negative	Brain
5	OVA_365	44	Serous	T3	N0	M1	Stage IV	Grade 3	NA	Liver
6	OVA_378	55	Serous	T3	N1	M1	Stage IV	Grade 3	NA	Spleen

### Whole-exome sequencing

For each tumour region (*n* = 33) and matched germline (*n* = 6), whole-exome sequencing (WES) was performed using SureSelectXT Target Enrichment (Agilent) on Illumina NovaSeq 6000. Further, target capture sequencing using SureSelect DNA Design at a median depth of 3048× (range 2470–3862) was performed to validate all putative somatic variants (Supplementary Table S1).

### Statistical analysis

All statistical analyses were executed on IBM SPSS Statistics (v.21). Where relevant, Mann–Whitney *U* test was utilised to compare continuous variables, Chi-Square test for categorical variables and Spearman’s rank correlation tests were used to determine associations. For all the statistical tests performed, *p* < 0.05 was considered statistically significant.

Complete ‘Materials' and ‘Methods' are described in the Supplementary Methods.

## Results

### Overview of the HGSOC cohort

To elucidate the relationship between primary HGSOC and its distant metastatic tumours, we performed multiregional WES (median coverage of 196×) on 33 tumour regions, resected from 15 primary tumours and 18 metastatic tumours from six patients (total of 4–6 tumour regions/patient; primary ranging 1–3 regions/patient and metastases ranging 1–4 regions/patient). WES results from all tumour regions were further validated using deep-targeted capture sequencing at a median coverage of 3048× and validation rate of 92.4%. Three patients (OVA_047, OVA_365 and OVA_378) were diagnosed with synchronous bilateral HGSOC, whereas the remaining three (OVA_003, OVA_013 and OVA_048) were identified with unilateral HGSOC. All patients, excluding (OVA_378) developed metachronous distant metastases, post chemotherapy, after a median of 18 months (ranging 10–22 months) from primary diagnosis. Four of these patients (OVA_003, OVA_013, OVA_047 and OVA_365) had intra-abdominal metastases (two of the liver and two of the spleen), except for one patient (OVA_048), who had incurred brain metastasis. Hence, primary HGSOC (with synchronous contralateral HGSOC, where relevant) and metastatic tumours were obtained at two separate time points. In addition, 80% of these patients were of young age (<50 years). Contrarily, one patient (OVA_378) presented synchronous metastasis of the spleen at primary diagnosis; therefore, all samples from this patient were obtained during the initial debulking surgery; however, contralateral HGSOC from this patient was not available for study. This patient was of older age (55 years). Upon primary diagnosis, all patients underwent initial debulking surgery, followed by the standard chemotherapeutic protocol of a carboplatin regimen (Table 1).

### Genomic architecture of primary HGSOC and matched metastatic tumours

**Fig. 1:**
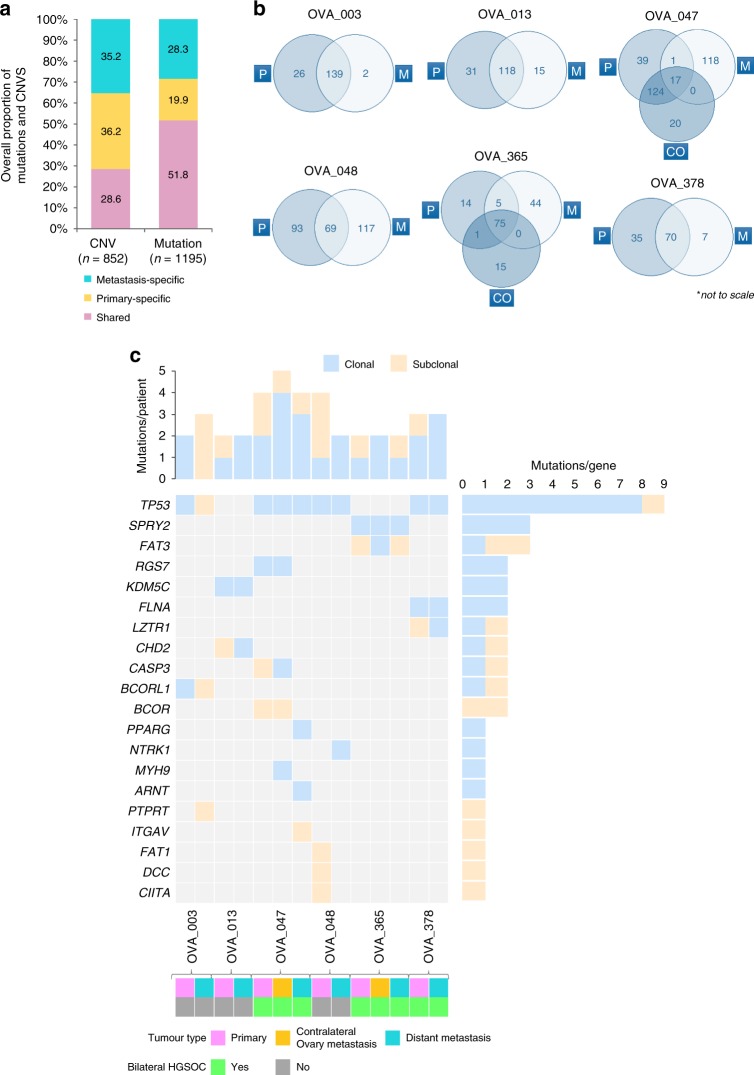
Distribution of alterations through HGSOC metastatic tumour progression. **a** Overall percentage of CNVs (*n* = 852) and mutations (*n* = 1195) shared between the primary and metastasis, specific to the primary and specific to the metastasis. **b** Venn diagrams show the number of unique and shared mutations in primary and metastatic tumours in each case; ‘P’ represents primary tumour, ‘CO’ represents the contralateral ovarian metastatic tumour and ‘M’ represents metastatic tumour. Venn diagrams are not to scale. **c** All cancer driver mutations (SNVs and Indels) detected for each HGSOC primary and corresponding metastasis, including contralateral ovary metastasis, where relevant. The top panel displays the number of driver mutations/patient identified across tumours, whereas the right panel shows the total count of driver mutations/gene.

We identified a median of 156 somatic mutations (ranging 95–181), including SNVs (single-nucleotide variants) and indels (small insertions and deletions), and a median of 97 copy number variants (CNVs) (ranging 25–153) in primary tumour regions, whereas metastatic tumour regions had a median of 151 somatic mutations (ranging 77–286) and 74.5 copy number variants (ranging 27–179). The events (mutations and CNVs) were categorised as clonal (ubiquitous presence in all tumour cells) or subclonal (partially present in all tumour cells) (Supplementary Table S2). In order to further corroborate the true status of mutations, private and branch mutations were evaluated across all sampled regions using Integrative Genomics Viewer (IGV) software, where mutations were manually retrieved if they reached ≥1% variant allele frequency (VAF), but had not been called by the variant-calling software (see Supplementary Methods). Across all sampled primary-metastasis pairs, a total of 619 (51.8%) mutations and 244 (28.6%) CNVs were shared between primary tumours and their corresponding metastases; 238 (19.9%) mutations and 308 (36.2%) CNVs were private to primary tumours, with 338 (28.3%) mutations and 300 (35.2%) CNVs private to metastases (Fig. 1a, b; Supplementary Fig. S1). Overall, the mutation and CNV burden did not differ significantly between primary and metastatic tumours (*p* > 0.05, Mann–Whitney *U* test); however, using contingency tables, we found significant differences between the proportions of CNVs and mutations shared and specific to either primary HGSOC or metastatic tumours (*p* = 0.0026, Chi-squared test). Upon stratification, we found a significantly higher proportion of shared mutations compared with shared CNVs pre-dissemination (*p* < 0.001, Chi-squared test), whereas, following dissemination, a significantly higher proportion of primary- and metastatic-specific CNVs were observed compared with tumour-type-specific mutations (primary-specific: *p* < 0.001; metastatic-specific: *p* = 0.0009; Chi-squared test). Thus, we indicate an increased mutational rather than CNV-based role during tumorigenesis and progression, whereafter this is reversed post dissemination, with increased chromosomal instability through CNVs rather than mutational involvement observed following dissemination in primary HGSOC and metastatic tumours, possibly contributing to therapy resistance and tumour maintenance.

**Fig. 2:**
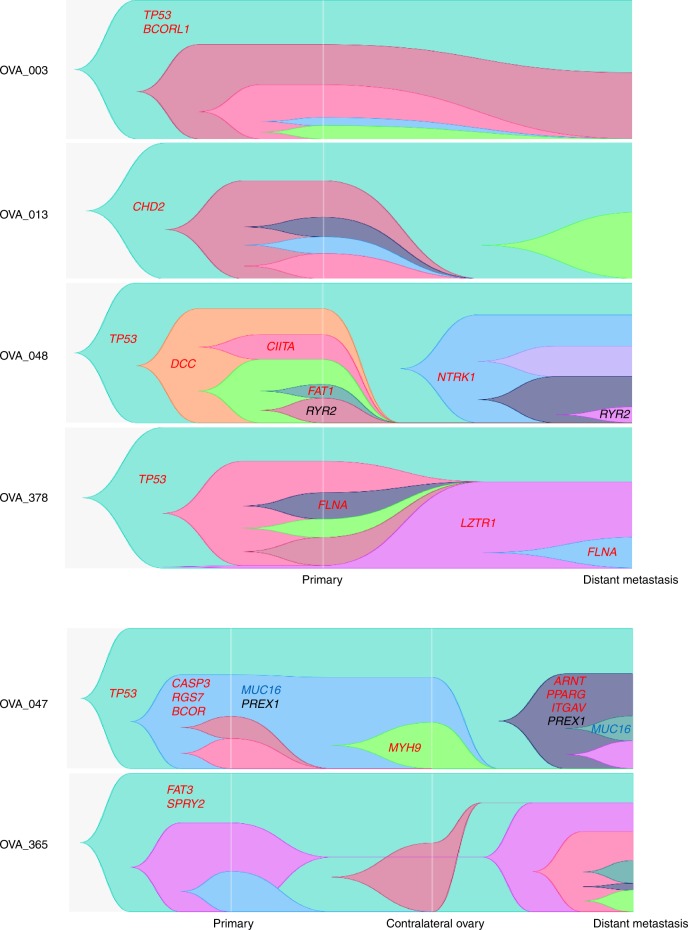
Evolution of HGSOC metastasis. Evolution of HGSOC metastasis based on cell fractions at different time points, depicting the history of individual clusters over time. Different colours represent separate clusters in a sample. Centred in relevant clusters are driver genes (in red), and parallel events converging at cancer genes (in blue) and non-cancer genes (in black).

We further investigated the distribution of driver events in the multiple tumour regions sampled from each patient. All patients harboured oncogenic and/or tumour-suppressor gene driver events (median 3, range 2–8 mutations and median 264, range 113–301 genes involving CNVs). The only recurrent driver mutation was *TP53*, which was shared between HGSOC primary and metastatic tumours, observed in four patients (OVA_003, OVA_047, OVA_048 and OVA_378) as clonal, except for one case (OVA_003) that became subclonal in the metastasis. Patient OVA_047 had a higher driver mutation rate of 35%, with a clonal *TP53* mutation in addition to subclonal copy number loss in primary tumours. Other mutations included *KDM5C* (1/6, OVA_013), *FLNA* (1/6, OVA_378), *RGS7* (1/6, OVA_047) and *SPRY2* (1/6, OVA_365), which were also clonal in both primary and metastatic tumours. Whereas, mutations in the *BCOR* gene (OVA_047) were consistently subclonal in primary and metastatic lesions. Other genes, such as *BCORL1* (OVA_003), *CASP3* (OVA_047), *CHD2* (OVA_013), *FAT3* (OVA_365) and *LZTR1* (OVA_378), were heterogeneous, having differing clonality in the primary versus the metastatic tumours. Despite the lack of identifiable recurrent mutations specific to either primary or metastatic tumours, single subclonal mutations in *CITTA* (OVA_048), *DCC* (OVA_048) and *FAT1* (OVA_048) were all specific to primary tumours. On the other hand, clonal mutations in *ARNT* (OVA_047), *NTRK1* (OVA_048), *MYH9* (OVA_047) and *PPARG* (OVA_047), and subclonal mutations in *ITGAV* (OVA_047) and *PTPRT* (OVA_003) were all specific to metastatic tumours (Figs. 1c and 2).

In contrast, copy number deletions in *NOTCH2* (3/6), *NRAS* (3/6), *RAF1* (3/6), *MPL* (2/6) and *PIK3CB* (2/6) were found recurrent and specific to primary tumours, whereby deletions in *IRF4* (2/6), *MUC4* (2/6), *RARA* (2/6), *STAT3* (2/6), *STAT5B* (2/6) and *TAF15* (2/6) were found recurrent and only specific to metastatic tumours. The most commonly recurring CNVs (≥3 patients) were typically deletions shared by both primary and metastatic tumours spanning p11–q26 on different chromosome regions (Supplementary Table S3). CNV events were slightly more clonal compared with subclonal, with a median of 56% (range 2–91%) being clonal and 44% (range 9–98%) as subclonal, indicating an initial role in tumorigenesis and disease progression in the primary tumour; however, this difference was not statistically significant (*p* > 0.05, Mann–Whitney *U* test). Furthermore, early occurring CNVs tended to be deletions, with 87% (range 0–100%) of all losses identified as clonal compared with 13% (range 0–100%) of gains (*p* > 0.05, Mann–Whitney *U* test). Clonal CNVs were found with a median of 18.0 Mbp (range 0.0–191) compared with 13.0 Mbp (range 0–161) as subclonal (*p* < 0.05, Mann–Whitney *U* test significant). Although the total number of CNVs were lower in metastases (median 74.5, range 27–179), compared with primary tumours (median 97, range 25–153, *p* > 0.05, Mann–Whitney *U* test), the number of driver mutations did not differ considerably between the tumour types. Metastatic tumour mutations were slightly more clonal (proportion = 69.0%) compared with primary tumours (proportion = 60.0%, *p* > 0.05).

### Analysis of pathways associated with the HGSOC metastatic process

To identify putative pathways associated with the HGSOC metastatic process, using ingenuity pathway analysis (IPA), we analysed all metastatic-specific mutations and CNVs affecting driver genes, absent in all primary regions but present in at least one matched metastatic region. Despite HGSOC primary tumours metastasising to distinct organ sites, such as the brain, liver and spleen, patients displayed convergence at selected pathways. Pathway analysis of metastatic-specific events revealed significant enrichment for genes associated with various pathways (*p* < 0.0001) (Supplementary Table S4), amongst which three patients (OVA_048, OVA_365 and OVA_378) converged at the previously identified Wnt/β-catenin signalling pathway in leukaemia, melanoma, breast and gastrointestinal cancers.^19^

### Evolutionary history and tumour clonal architecture between primary HGSOC and metastasis

**Fig. 3:**
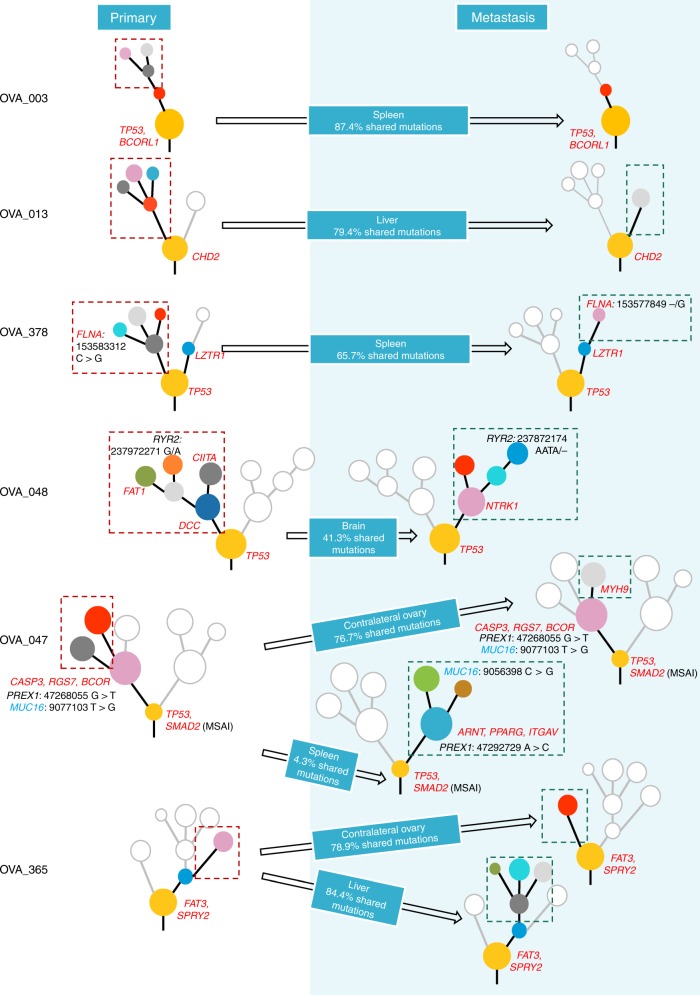
Phylogenetic tree summary. Phylogenetic trees for six HGSOC tumours from primary to metastasis. Highlighted are driver genes (in red), and parallel events converging at cancer genes (in blue) and non-cancer genes (in black). Different coloured nodes represent distinct clones. Arrows indicate unidirectional metastatic progression after X% shared mutations at primary tumour to stated regional and distant sites. Clones specific to primary tumour tissues, ablated by therapy, not selected during metastatic seeding or occurred after metastatic seeding, have been outlined with red dashed lines. New clones in metastatic tumour tissues have been outlined with dark green dashed lines.

To ascertain the clonal architecture and evolutionary dynamics from tumorigenesis to metastatic sites, phylogenies were mapped spatially and temporally for each HGSOC tumour and the corresponding distant metastasis tumour, based on mutational cancer cell fractions (CCFs) from both SNVs and Indels^20^ (Fig. 3, Supplementary Fig. S2). Putative driver genes, based on The Cancer Genome Atlas—Ovary,^18^ and other large genomic studies^21–23^ and parallel events converging at the same gene were also detailed on each phylogenetic tree. Despite independent evolution of subclones arising from a single ancestral clone, parallel events between the primary and metastatic tissues show convergence of somatic mutations in distinct branches on the same gene.^24^ Three patients (OVA_047, OVA_048 and OVA_378) had evidence of parallel evolution, with *MUC16*, *PREX1*, *RYR2* and driver gene *FLNA*. We also analysed the parallel evolution of CNVs, by mirrored subclonal allelic imbalance (MSAI),^25^ where only patient OVA_047 showed MSAI events, with parallel deletions in chromosomes 7 and 18.

We identified a total of 44 mutation clusters in six patients, with a median of ~7 (ranging 2–10) distinct clusters per patient. All sampled regions from all six patients had various sharing of mutations from 4.3 to 87.4% of all clustered mutations indicating a clonal relationship. We further identified the relatedness of each ovarian tumour to its contralateral tumour, in synchronous bilateral HGSOC cases with available tumour tissues (OVA_047 and OVA_365), establishing a primary-to-metastasis ancestral relationship, where the primary tumour in one ovary was seeded in the other after a period of time, with the majority of somatic mutations although shared with the primary tumour, also further diversified upon migration.

All patients demonstrated at least one driver, such as *TP53*, *BCORL1*, *CHD2*, *FAT3* and *SPRY2* mutation in their common ancestral clone, followed by further subclonal acquisition of driver events as the tumour progressed in three patients (OVA_047, OVA_048 and OVA_378).

Furthermore, we sought to identify the patterns of progression towards metastasis from primary HGSOC, whilst determining whether a subclone may have arisen from a tumour region at low cellular prevalence before becoming dominant in a distant region. We found several SNVs with increasing/decreasing CCFs during metastatic dissemination, possibly due to selection pressures or the effect of treatment at the metastatic tumour site. Lower CCFs in some metastatic regions showed sensitivity to treatment. Therefore, clusters showing lower CCFs in the primary tumour, but a higher CCF in their metastasis counterpart, might contain important chemotherapeutic-resistant mutations.

Most of our patients were diagnosed with metachronous metastatic disease after a latency period of 10–22 months, apart from one patient (OVA_378), who was diagnosed with synchronous metastasis, at the same time as the primary tumour; however, no significant difference was observed between metachronous and synchronous metastatic progression types. In fact, most clonal diversity in our cases emerged at the primary HGSOC site, where metastatic divergence within the primary tumour occurred after a median of 77.8% (range 4.3–87.4%) final molecular time at the primary tumour (based on phylogenetic analysis of all clustered mutations in genomic regions consistent across all sampled regions), followed by unidirectional monoclonal or polyclonal seeding to regional and distant sites. Regional metastasis to the contralateral ovary in both patients (OVA_047 and OVA_365) occurred late (≥ 50%^26^), after the accumulation of the majority of primary tumour diversity, at a final molecular time of 76.7% in the primary tumour of OVA_047 and 78.9% in OVA_365 (Fig. 3). In addition, the direction of metastasis and the original primary tumour amongst the tumour pair was also established, by comparing tumour sizes in cm^3^, with larger tumours having more tumour cells, and more likely to have established themselves earlier than smaller-sized tumours.

Apart from two patients (OVA_047 and OVA_048), all patients exhibited linear metastatic progression^26^ patterns in their distant metastases. Despite the fact that patient OVA_378 also demonstrates linear progression, all sampled regions were treatment naive, where the primary and synchronous metastatic tumours continued to clonally expand in parallel converging on driver gene *FLNA* in late clonal expansions irrespective of therapeutic pressures. Since previous studies have shown carboplatin treatment having limited blood–brain barrier (BBB) penetration (2.6%) and subsequently minimal therapeutic effect in brain metastases, despite its ability to penetrate abnormal BBB,^27^ the evolutionary processes in the brain metastasis (OVA_048) were less likely to be affected or shaped by the carboplatin treatment, with all metastatic clones reaching near 100% CCFs. Interestingly, one patient (OVA_003) acquired all somatic mutations in the metastatic tumour from the primary tumour, with no further clonal expansion, even after 20 months, post therapy. Furthermore, amongst the primary tumour clones that had disseminated to the spleen, advanced subclones had been completely eradicated by treatment, whereas residual clones remained partially resistant, with reduced CCF only in selected metastatic regions. Although two patients (OVA_013 and OVA_365) incurred late disseminating metastases of the liver, both had highly treatment-resistant ancestral clones with further clonal expansions post metastatic dissemination, all reaching near 100% CCF at metastatic sampling, albeit limited clonal expansion was observed in OVA_013 compared with the multiple diverging clonal expansions in OVA_365. Possibly, further subclones in OVA_013 may have been too small to discriminate, or this was due to an earlier detection of metastasis at 16 months (OVA_013) compared with 22 months (OVA_365) post therapy. Therefore, if patient OVA_013 was sampled at a further time point, eventual clonal expansions may have produced similar divergent subclones as seen in patient OVA_365.

The two remaining patients (OVA_047 and OVA_048) incurred early dissemination of only the main ancestral primary tumour clone, which was therapy-resistant, to distal organs during the development of the primary tumour, where only 4.3% (OVA_047) and 41.3% (OVA_048) of clustered mutations were shared with their matched distant metastases, from which primary and metastatic tumours evolved in parallel. Both patients only carried a single *TP53* mutation in their disseminating clone. Despite early or late dissemination to distant metastases, both synchronous bilateral HGSOC primary tumours showed equivalent molecular timing for dissemination to their contralateral ovaries.

Like previous reports, most of our patients also demonstrated abundant concordant mutations between primary and metastatic tumours, with only a small number of metastatic-specific mutations.^28^ To further confirm whether metastatic-specific SNVs in our cohort occurred prior to (early) or following (late) dissemination, we investigated the presence of metastatic-specific mutations in the primary tumour, to see if they were present at a low frequency. Using Bayesian hypothesis testing and deepSNV, the significance of each mutation frequency was assessed.^29^ Upon analysis, all VAFs were reported significant by deepSNV in metastatic tumours relative to the matched primary tumours, apart from a mutation of *KIAA1429* gene in patient OVA_013 and a mutation of *BEGAIN* gene in patient OVA_048 (Supplementary Table S5, *p* < 0.05). This suggests that most metastatic-specific mutations evolved post dissemination to the metastatic sites. However, this does not exclude the possibility of some of these mutations existing in the primary tumour at a frequency lower than our detectable range.

Interestingly, regardless of the type of metastasis (regional or distant), or the metastatic progression model (early/parallel or late/linear), all metachronous metastases had disseminated prior to diagnosis of the primary tumour, which was also confirmed by the occurrence of the contralateral ovarian tumour present in synchronous bilateral HGSOC tumours at diagnosis. At this time point, the distant metastasis may have survived treatment and avoided detection, by comprising only a selected number of tumour cells containing all disseminated primary clones with resistant components, which then continued growing rapidly, acquiring further clones until subsequent diagnosis of distant metastasis.

### Differential selection from primary HGSOC to metastasis

Ongoing selection during HGSOC evolution can help in identifying evolutionary constraints, eventually dictating the evolutionary routes of this tumour and metastasis. We sought to further estimate positive selection via a dN/dS ratio, which parallels substitution rates at nonsynonymous sites to those at synonymous sites. Thus, we account for the trinucleotide context of each mutation, and determine the enrichment of protein-altering mutations compared with the background mutation rate.^30^ Positive selection (dN/dS >1) was observed, when considering all exonic missense mutations in primary tumours, but not metastatic regions. Positive selection was also observed in all exonic nonsense mutations in both primary and metastatic tumours. However, upon temporal dissection, positive selection was only observed in clonal and subclonal nonsense mutations of metastatic and primary tumours, respectively. Clonal nonsense mutations were found depleted in the primary tumours compared with the metastasis, whereas depletion of nonsense subclonal mutations was observed in metastasis by comparing it with primary tumours. Contrarily, despite depletion in clonal mutations being observed, positive selection was observed in all exonic missense and nonsense subclonal mutations, from primary tumours (Supplementary Table S6, Supplementary Fig. S3). This suggests that mutations may be shaped by positive selection after tumorigenesis over various stages between primary HGSOC and metastasis.

### Dynamic mutational spectra and signatures from primary HGSOC to metastasis

**Fig. 4:**
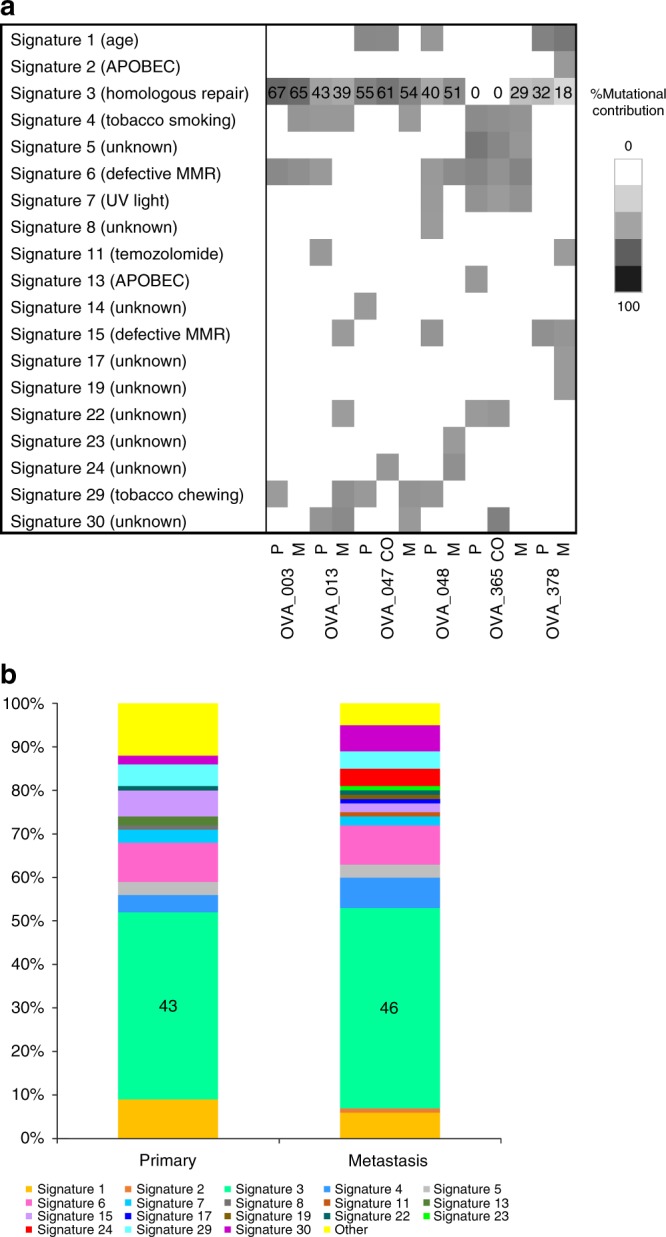
Mutational signature processes in HGSOC metastasis. **a** The percentage mutational contribution, in primary HGSOC and metastasis, to the 30 published mutational signatures from the COSMIC database. Increasing intensity represents increasing contribution of mutations. **b** The overall mutational contribution from the cohort to the 30 mutational signatures at primary and metastasis. Integers for significantly correlated signatures have been presented. ‘P’ represents primary tumour, ‘CO’ contralateral ovarian metastatic tumour and ‘M’ metastatic tumour.

Varying subclones and patterns of trinucleotide signatures are the result of alternating mutational stresses over tumour history. It is particularly important to understand the effect of these mutational processes that shape the evolution between HGSOC and metastasis, to potentially inform clinical strategies to limit tumour adaptation and metastatic progression. Published mutational signatures were used to analyse primary and metastatic tumour SNVs. We identified several signatures within the primary-metastasis tumour pairs, of which the most prominent were signature 1 (3/6, 50%), involving the endogenous spontaneous deamination of methylated cytosines correlating with patient age at the time of cancer diagnosis; signature 3 (6/6, 100%), associated with failure of DNA double-strand break repair by homologous recombination; signature 4 and/or 29 (5/6, 83%), associated with tobacco carcinogens; signature 6 and/or 15 (5/6, 83%), two of the four signatures related to defective mismatch repair (MMR); signature 30 (3/6, 50%), of unknown aetiology (Fig. 4; Supplementary Figs. S4, S5 and S6). Signature 3 (homologous recombination deficiency) was significantly correlated with increased mutational burden (Spearman’s rank correlation, rho  =82.9%, *p* = 0.042), and showed consistent contribution of SNVs across primary and metastatic tumours in all six patients. Furthermore, signature 3 only generated higher proportions of subclonal SNVs in both primary and metastatic tumours, upon temporal dissection (Primary: Spearman’s rank correlation, rho=82.9%, *p* = 0.042; Metastasis: Spearman’s rank correlation, rho=94.3%, *p* = 0.005) (Fig. 4; Supplementary Figs. S4, S5 and S6).

### Clinical relevance of genomic alterations from primary HGSOC to metastasis

**Fig. 5:**
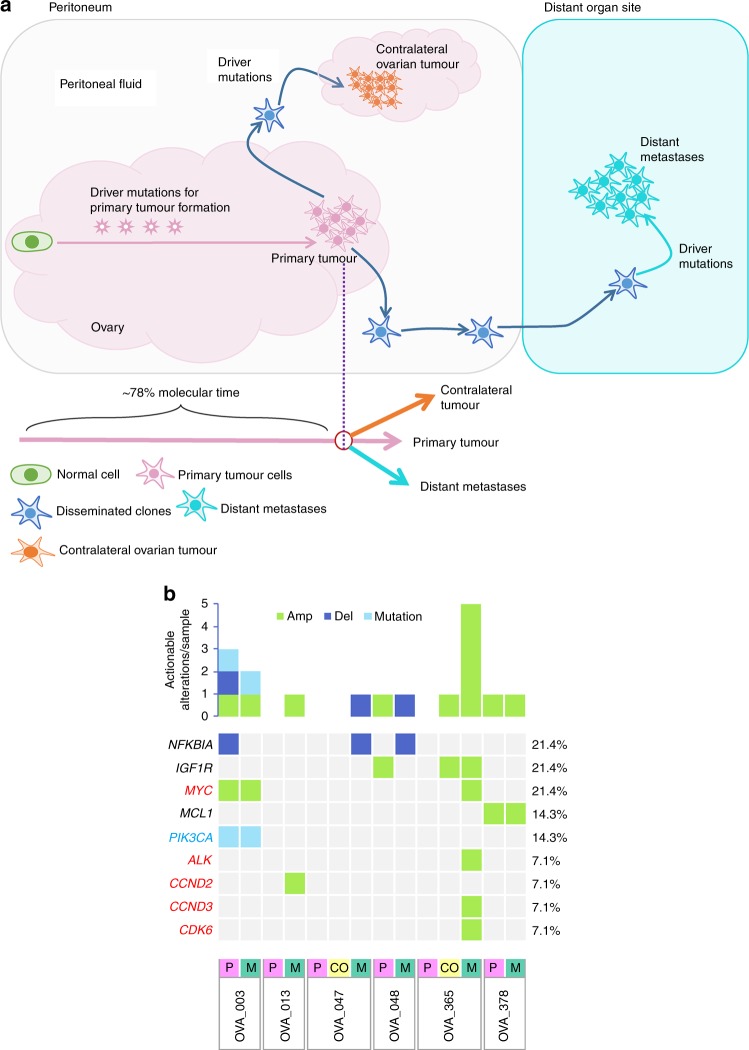
Final molecular time of metastatic diversions and clinically actionable events in HGSOC metastasis. **a** Metastatic tumours disseminate after a median of ~78% molecular time. Primary tumours show more genomic similarities with distant metastases compared with contralateral ovarian tumours. Driver mutations were mostly shared by primary, distant metastasis and contralateral tumours. Potentially clinically actionable events in the metastatic HGSOC cohort have been indicated, where (**b**) a more detailed overview of actionable events in individual cases is also presented. All clinically actionable genes were detected for each primary HGSOC, contralateral ovary metastasis (where relevant) and the corresponding distant metastasis. The top panel shows the number of actionable genes per case identified across the tumour types in a case, whereas the right-hand side panel shows the percentage of actionable variants per gene found across the cohort. On the left-hand side panel, genes coloured red are driver cancer genes, blue are non-driver cancer genes and in black are the non-cancer genes. ‘Amp’ represents gene amplifications, ‘Del’ represents gene deletions, ‘P’ represents primary HGSOC tumour, ‘CO’ represents contralateral ovary metastasis, where relevant and ‘M’ represents metastatic tumour lesions.

Clinically actionable events occurring early in molecular time, which are present in all sampled regions, may present robust therapeutic targets for patients with metastatic disease. Hence, immune checkpoint inhibitors and poly ADP-ribose polymerase (PARP) inhibitors may be suitable for the treatment of both primary HGSOC tumours and metastases in our cohort, via antitumour synthetic lethality, due to the sustained involvement of defective DNA repair pathways over both tumour types.^31,32^ Subclonal events can further inform the clinician about potential prognostic or diagnostic factors, and/or resistance to certain therapies, increasingly so in patients exhibiting parallel metastatic progression, where the primary tumour varies considerably from the metastasis. Using the TARGET database (v3), we were able to establish potentially clinically actionable events for individual cases in our metastatic HGSOC cohort. We identified clinically actionable genes in all of our six cases, with a median of two (range 1–6) actionable genes per patient, with a total of nine actionable genes across the cohort, including genes *NFKBIA*, *IGF1R*, *MYC*, *MCL1*, *PIK3CA*, *ALK*, *CCND2*, *CCND3* and *CDK6* (Fig. 5).

Recurrently actionable genes in the cohort included deletions in the *NFKBIA* gene (3/6 cases), amplifications of *IGF1R* (2/6 cases) and *MYC* (2/6 cases). These alterations in *NFKBIA* and *MYC* are shown to potentially be prognostic in some cancer types. On the contrary, amplifications in *IGF1R* may predict sensitivity to IGFR1-R inhibitors. Similarly, amplifications in *CCDND3*, *CCDN2* and *CDK6*, may be targetable by CDK4/6 inhibitors. Amplifications in *ALK* may be targeted with ALK inhibitors such as crizotinib. Whereas, mutations in *PIK3CA* are possibly targetable by PI3K, AKT and MTOR inhibitors, whilst also possibly predicting resistance to anti-RTK therapy (e.g., cetuximab), anti-EGFR tyrosine kinase inhibitors, trastuzumab and lapatinib. Similarly, amplifications in *MCL1* may predict resistance to anti-tubulin chemotherapy.

Case OVA_003 presented three actionable genes, whereby mutated *PIK3CA* and amplifications in *MYC* were shared between primary HGSOC and metastatic tumours, but deletions in *NFKBIA* were specific to the primary tumour. Case OVA_013 exhibited a single actionable amplified *CCDN2* gene in its distant metastatic tumour, with no actionable genes found shared between primary and metastatic tumour, or found specific to the primary tumour. Similarly, case OVA_047, also did not present any actionable genes shared between primary and metastatic tumour, or specific to the primary tumour or contralateral ovary metastasis, with only a single actionable deleted *NFKBIA* gene in its metastatic tumour. Case OVA_048 also presented with a single actionable deleted *NFKBIA* gene in its metastatic tumour, but also had a single actionable *IGF1R* amplification in its primary tumour, whereas no actionable genes were shared between the primary HGSOC and metastatic tumours. Interestingly, although case OVA_365 did not present any primary tumour-specific or shared actionable genes, it did present with one shared actionable IGF1R amplification between its contralateral ovary and distant metastasis, with an additional four actionable gene amplifications in *MYC*, *ALK*, *CCND3* and *CDK6*, specific to its distant metastatic tumour. Lastly, case OVA_378 presented a single shared actionable *MCL1* amplification between its primary and metastatic tumour, with no additional actionable genes specific to either primary HGSOC, or its corresponding distant metastasis.

Furthermore, as metastatic-specific events, in three patients (OVA_048, OVA_365 and OVA_378) converged at the Wnt/β-catenin signalling pathway, the metastatic lesions in these patients may potentially benefit from targeting this pathway. Depleted *RARA* may be targeted using oral retinoids, such as acitretin; depleted *SRC* using Src-tyrosine kinase inhibitors, such as bosutinib; *SMO* with smoothened inhibitors or hedgehog pathway antagonists, such as vismodegib *RARA*, *SRC* and *SMO*, can also be used as diagnostic, prognostic and/or therapy-response biomarkers in cancer.

## Discussion

Understanding the complete genomic background of HGSOC tumours will guide targeted therapies, paving the way for the use of precision medicine in this highly lethal cancer. In the limited samples studied, we observed that the majority of the primary tumour genome was seeded at metastatic sites, including most driver and passenger mutations. However, the only recurrent clonal driver mutation shared between primary and metastatic HGSOC was *TP53*. Interestingly, a higher driver mutation rate of 35% was observed in patient OVA_047 with a clonal *TP53* mutation in addition to subclonal copy number loss in primary tumours, contributing to the prevalent instability in HGSOC as previously reported.^33,34^ Other clonal mutations observed in both primary and metastatic tumours were seen in *KDM5C* and *SPRY2* genes suggesting their sustained involvement in tumour initiation and maintenance or progression. Although previous studies^35–37^ have suggested *TP53* as the most potentially targetable gene to be used as a biomarker, and in the development of specific targeted drugs for HGSOC, biallelic inactivation of *TP53* is required in order to reach clinical significance, where our cohort only presented single mutations. In contrast, we reported a total of nine actionable genes across the cohort, including genes *NFKBIA*, *IGF1R*, *MYC*, *MCL1*, *PIK3CA*, *ALK*, *CCND2*, *CCND3* and *CDK6*. Only three genes, including *MCL1* (OVA_378), *MYC* and *PIK3CA* (OVA_003), were found to be clinically actionable in both primary HGSOC and metastatic tumours. Furthermore, other suitable agents for the treatment of both primary HGSOC tumours and metastases in our cohort may include immune checkpoint and PARP inhibitors, due to the sustained involvement of defective DNA repair pathways over both tumour types.^31^ Metastatic-specific events, in three patients (OVA_048, OVA_365 and OVA_378) converging at the Wnt/β-catenin signalling pathway, also indicate the potential use of targeted therapies, such as oral retinoids, e.g., acitretin, Src-tyrosine kinase inhibitors, e.g., bosutinib and smoothened inhibitors or hedgehog pathway antagonists, such as vismodegib, for the treatment of metastatic lesions in these patients. *RARA*, *SRC* and *SMO* can also potentially be used as diagnostic, prognostic and/or therapy-response biomarkers in cancer.

As expected with evolutionary bottlenecking, there were slightly more clonal metastatic mutations (median proportion = 69.0%) compared with primary mutations (median proportion = 60.0%, *p* > 0.05).^38,39^

All our primary HGSOC tumours followed Darwinian-based branching evolutionary patterns during tumorigenesis, with divergence of subclones from a common ancestral clone, leading to the coexistence of subclones, congruent with a previous study identifying branched evolution with metastatic cross-seeding.^14^ Other studies have also reported neutral evolution^40,41^ in primary ovarian cancer. During tumorigenesis, early tumour growth is inevitably vulnerable to external pressures and microenvironmental niches, such as the immune system, nutrient deprivation and anatomical barriers,^42,43^ where fitness gained through the acquisition of driver mutations, and subsequent selective sweeps to generate multiple subclones, as well as driver CNVs, allow the tumour to overcome such obstacles.^44^

Upon metastasis, a clone or multiple clones from the primary tumour, migrate to a distant site to form a new colony often generating a founder effect, where previous cancer studies have shown varying degrees of genetic divergence between metastatic and primary clones, where due to evolutionary bottlenecking, there can be overall reduced genetic variability in the new population.^45,46^ In spite of three patients (OVA_003, OVA_365 and OVA_378) having metastases at other sites, such as brain (OVA_003), contralateral ovary (OVA_378), spleen and lower chest wall (OVA_365), for the purpose of this study metastatic cross-seeding could not be explored as tumour tissue from these sites was not available for study. Metastatic clones can disseminate early (before primary tumour diversification) or late (after primary tumour diversification) in metastases.^47^ Metastatic divergence occurred after a median of ~78% final molecular time of the primary tumour based on phylogenetic analysis of all clustered mutations (Fig. 5). Hence, the majority of our cases supported the linear progression model (4/6 patients) of metastasis, in which the primary tumour cells diversified over a period of time, gaining several mutations and CNVs including driver events (e.g., *TP53, FAT3* and *BCORL1*) before achieving metastatic potential to migrate and colonise distal sites.^48^ Linear progression models have overall less heterogeneity and more sharing of mutations between primary and metastatic tumours, as seen in our patients.^49^ Therefore, targeting trunk alterations in this patient population seems highly desirable. In contrast, two patients demonstrated parallel progression models where dissemination occurred early, including only a single driver *TP53* mutation in their disseminating clone, with extensive primary and metastatic tumour diversification occurring post metastatic seeding; hence, very limited mutations were shared between primary and metastatic tumours, as seen in a previous HGSOC study.^50^ This suggests that the metastatic potential of a tumour cell is not dependent on a complex repertoire of mutations, where few alterations can suffice for dissemination and ectopic survival, with most somatic evolution occurring at the metastatic site due to extensive adaptation to the new microenvironment.^13^ Due to the higher genetic divergence between primary and metastatic tumour, the usage of the primary tumour in these cases to predict therapeutic strategies in metastatic disease may therefore be inapt. No distinct clinicopathological characteristics were found between the two groups of patients that could explain the difference in progression models discerned. Ideally, larger studies will be required to get a complete understanding and explicate the reasons for the types of metastatic progression patterns observed in HGSOC.

We also found that signature 3 (homologous recombination deficiency) was significantly correlated with increased mutational burden. Concurring with previous studies, the majority of HGSOC genomes corresponded with deficiencies in homologous recombination and other DNA mismatch repair pathways;^51^ hence, the resulting intratumour heterogeneity is caused by a collective dysregulation of apoptosis and DNA repair processes.^14,52^ Despite the need to be validated in a larger study, the overall data indicates that various mutational processes have ongoing important roles during HGSOC evolution and metastatic progression, with mostly greater heterogeneity amongst patients than across different evolutionary stages, except signature 3, which indicates consistent relative contribution over time (Fig. 4; Supplementary Figs. S4, S5 and S6).

Our analysis also identified metastatic-specific events associated with gene enrichment in genes related to the regulation of the Wnt/β-catenin pathway. Interestingly so, since gene expression data from TCGA ovarian cancer also found involvement of disrupted Wnt/β-catenin pathways in tumours with poor prognosis, we suggest a possible role of Wnt/β-catenin in ovarian cancer.^53^ With several clinical associations between altered Wnt/β-catenin pathways, patient outcome and drug resistance have also been observed. Our findings support that targeting Wnt/β-catenin pathway might be an important therapeutic target in metastatic HGSOC.

In order to clearly map this genetic complexity, larger studies with more metastatic tumour samples are needed despite the difficulties of obtaining or accessing metastatic tumours. Despite the limited number of available samples, this study shows that such effort has a significant clinical implication, providing deeper insights into clonal evolution and mutational processes that can pave the way to new therapeutic targets.

## Supplementary information

Supplementary_Material

## Data Availability

Raw data are deposited in the Sequence Read Archive database of National Center for Biotechnology Information (NCBI) with accession number PRJNA603873.
