# Supplementary material for: Genetic heterogeneity and evolutionary history of high-grade ovarian carcinoma and matched distant metastases
Source: Br J Cancer. 2020 Feb 26;122(8):1219–30. doi: 10.1038/s41416-020-0763-4 (PMC7156387; doi:10.1038/s41416-020-0763-4)
Supplement: Supplementary file 1 — Supplementary_Material [file 41416_2020_763_MOESM1_ESM.pdf]

SUPPLEMENTARY INFORMATION

Supplementary Methods. Supplementary materials and methods. (File type: .docx)

Supplementary Table S1. The quality control metrics of the targeted capture sequencing. (File type: .xlsx)

Supplementary Table S2. Somatic mutations validated by target capture sequencing. (File type: .xlsx)

Supplementary Table S3. Copy number variations predicted by using Facets algorithm. (File type: .xlsx)

Supplementary Table S4. Significant pathways (P < 0.01) of subclonal mutations using Ingenuity pathway analysis. (File type: .xlsx)

Supplementary Table S5. Metastatic specific mutations with VAF. (File type: .xlsx)

Supplementary Table S6. dN/dS ratio. (File type: .xlsx)

Supplementary Fig. S1. Mutations cluster heatmap of individual samples.

Coloured bar represents all mutation clusters, with corresponding cancer cell fractions (CCF) and variant allele frequency (VAF) values as a heatmap. Driver genes are

highlighted in red and cancer genes in blue and non-cancer genes in black, with corresponding parallel mutations converging at the same gene indicated in orange. (File type: .pdf)

Supplementary Fig. S2. Phylogenetic trees.

Phylogenetic trees for 6 metastatic HGSOC tumours. For each sample, only the optimal tree derived using LICHeE (Lineage Inference for Cancer Heterogeneity and Evolution) has been presented. Highlighted are driver genes (in red), and parallel events converging at cancer genes (in blue) and non-cancer genes (in black). The middle top pane shows the complete phylogenetic tree as constructed based on mutation clusters, with primary and metastatic phylogenetic trees have been shown separately in a black box. Different coloured nodes represent distinct clones, with the number of mutations contributing to the cluster displayed inside each node. Below are the phylogenetic trees shown for each region, where clusters not found in a given region have been represented as grey empty nodes. “P”, represents regions from primary tumours; “CO”, represents contralateral ovarian metastatic tumour tissue; “M”, represents metastatic tumour tissue. Beneath each regional phylogenetic tree, a grid of a 100 representative cells shows the mutational clusters present via coinciding cluster colours. (File type: .pdf)

Supplementary Fig. S3. dN/dS ratio.

Plot showing dN/dS ratio for overall and temporally dissected (clonal and subclonal) mutations, calculated as Missense and Nonsense mutations, for metastatic HGSOC cohort. (File type: .pdf)

Supplementary Fig. S4. Overall signature shifts.

Overall signature shifts between primary and metastasis. Individual legends have been included with each sample. “Primary” represents primary tumour whereas “Mets” represents metastatic tumour. (File type: .pdf)

Supplementary Fig. S5. Clonal signature shifts.

Clonal signature shifts between primary and metastasis. Individual legends have been included with each sample. “CP” represents clonal mutations in the primary tumour, whereas “CM” represents clonal mutations in the metastatic tumour. (File type: .pdf)

Supplementary Fig. S6. Subclonal signature shifts.

Subclonal signature shifts between primary and metastasis. Individual legends have been included with each sample. “SP” represents subclonal mutations in the primary tumour, whereas “SM” represents subclonal mutations in the metastatic tumour. (File type: .pdf)

Supplementary materials and methods

Patients and tumour samples

We collected a total of 33 tumour regions with corresponding normal whole blood and metastatic tumour (3 spleen, 2 liver, 2 contralateral ovary and 1 brain) from 6 patients with high-grade serous ovarian from King Faisal Specialist Hospital & Research Centre. All six patients presented a primary tumour histological subtype of serous cystadenocarcinoma. Three patients (OVA_047, OVA_365 and OVA_378) were diagnosed with synchronous bilateral HGSOC, with primary tissues and contralateral ovary metastases sampled at the same time-point, whereas patients OVA_003, OVA_013 and OVA_048 had unilateral HGSOC. However, metastatic tissue obtained from the contralateral ovary of patient OVA_378 was not available for study. Five patients were sampled at two time-points, at primary diagnosis and after the development of metastasis, whereas the remaining patient (OVA_378) had synchronous metastasis, therefore primary and metastatic tumours were acquired at a single time-point. To assess intra-tumour heterogeneity, samples of at least two tumour regions, separated by a margin of 0.5 cm to 1.0 cm (depending on the available tumour size) have been taken for the study. Clinical characteristics of the ovarian cancer cohort are provided in Table 1. Institutional Review Board (IRB) of the King Faisal Specialist Hospital & Research Centre approved the study under the Project RAC # 2060 008 on ovarian archival clinical samples.

Sample processing

Tumour tissue from each region was used for genomic DNA extraction using Gentra DNA isolation kit (Gentra, Minneapolis, MN, USA), following the manufacturer's

recommendations as described previously [1]. DNA was quantified by Qubit (Invitrogen).

Whole exome sequencing and mutation calling

For each tumour region (n=33) and matched germline (n=6), Whole exome sequencing (WES) was performed using SureSelectXT Target Enrichment (Agilent) on Illumina NovaSeq 6000. We sequenced to a median coverage of 196x (range 153-246), 33 tumour regions (17 primary and 16 metastatic tumour tissues), with a median of 3 regions per tumour (ranging 1-3) and 6 matched germline samples derived from whole blood (S6 Table). Quality metrics, mapping variant-calling and annotation were performed as previously described [2].

Only SNVs identified as 'KEEP' by MuTect and indels with a somatic p-value of ≤0.001 identified by VarScan, were retained. In addition to satisfying standard MuTect and VarScan filters, variants presenting with the following features were excluded: common SNPs (minor allele frequency of >0.01) found in dbSNP, the National Heart, Lung, and Blood Institute exome sequencing project, 1000 Genomes, Exome Aggregation Consortium (ExAC) and in our in-house data from exome sequencing of ~800 normal samples. All mutations were manually verified using the Integrated Genomics Viewer (IGV) v2.4.10 to filter out false-positives.

Only mutations with a variant allele frequency (VAF) ≥5%, regional sequencing depth ≥15 and ≥4 altered sequence reads, where germline reads ≥8 with a AF<5% were retained. Variants with more than 2 mapping quality zero reads were considered false-positives

and removed from the analysis.

Mutation validation

Further target capture sequencing using SureSelect DNA Design at a median depth of 3048× (range 2470-3862) was performed to validate all putative somatic variants (S6 Table). All filters were applied as above with the addition of ≥5 reads supporting the variant call and the tumor VAF of ≥1%. Variants absent in any of the sampled regions were manually checked to exclude presence at a low VAF and added if VAF satisfied ≥1%.

Gene copy number profiling and cancer cell fraction

FACETS v0.5.13 [3] was used to determine copy number variations (CNVs) and regions of loss of heterozygosity (LOH). Mean allelic frequency (MAF) was produced from mutation data. ABSOLUTE v1.0.6 [4] defined the integer copy number and cancer cell fractions (CCFs) of mutations and CNVs. A mutation with a probability of >50%, or with a lower bound 95% confidence interval CCF >90%, was classified as clonal, and subclonal otherwise [5].

CNVs were further defined as gains and losses, amplifications and deletions, in relation to the average ploidy of all sampled regions from a given patient using the copy number for each segment from facets. Genome-wide copy number gains and losses were defined by dividing the copy number data of each sample by the sample mean ploidy.
Gene-level amplifications and deletions were classified by mean gene copy number ≥2x

ploidy + 1 copy or ≤2x ploidy - 1 copy respectively. Furthermore, if all tumour regions incurred the equivalent CNV, the gene was classified as clonally amplified or clonally deleted. Absence of the CNV in any one region was classified as subclonal [6].

Driver mutation Classification

Non-silent variants were classified based on a list of potential driver cancer genes (n=745), in the COSMIC cancer gene census (v87), genes identified in TCGA-Ovary [7], and other large genomic studies [8-10]. Putative driver genes were classified: if the gene was identified as tumour suppressor by COSMIC and the variant was found to be deleterious (stop-gain or predicted deleterious in two of the three computational tools - Sift, Polyphen and MutationTaster; or if the gene was classified as tumour oncogene and an exact variant was found ≥3 times in COSMIC [6].

Mirrored subclonal allelic imbalance

B-allele fractions (BAFs) were calculated for all heterozygous SNPs as the ratio of the minor allele to the total allele count across different tumour regions, where copy number segments were considered to have allelic imbalance arising from distinct parental alleles, if mirrored independent copy number events were found in at least one region [6] .

Phylogenetic trees

Cancer cell lineages were reconstructed using LICHeE [11], by clustering high depth somatic variants from multiple tumour biopsies of a single patient to via presence/absence across multiple tumours and their variant allele frequency (VAFs) or

cancer cell fractions (CCFs) as lineage markers by relying on the perfect phylogeny model.

Molecular time at dissemination

Final molecular time at the primary tumour was estimated from the phylogenetic analysis of all mutations in genomic regions with consistent CCF across all sampled regions, by calculating the percentage of shared clustered mutations in the primary tumour and the metastasis for each patient, where additional private mutations in either tumour are assumed to have occurred post-dissemination [12].

deepSNV for metastatic-specific mutations

The statistical significance of metastatic-specific mutations was calculated using R package deepSNV v.1.30.0, to investigate the possible presence of these mutations in matched primary tumours at low frequencies. Bayesian hypothesis testing was used to determine the significance of each mutation.

dN/dS analysis

Maximum-likelihood method implemented in dNdScv R package was used to estimate dN/dS values for missense (ωmis) and nonsense (ωnon) mutations exome-wide as well as for the cancer genes. The substitution models provided in the package were used and maximum-likelihood estimates for ωmis and ωnon were obtained.

Mutational signature analysis

Mutational signatures for each set of primary and metastatic SNVs, with additional temporal dissection were separately predicted using the deconstructSigs package in R, by comparing 30 published mutational signatures from the COSMIC database reported in different cancer types (https://cancer.sanger.ac.uk/cosmic/signatures).

Pathway analysis

Ingenuity Pathway Analysis (IPA, http://www.ingenuity.com) was used to identify putative pathways by analysing metastatic-specific driver genes.

Statistical analysis

All statistical analyses were executed on IBM SPSS Statistics (v.21). Where relevant, Mann-Whitney U test was utilized to compare continuous variables, Chi-Square test for categorical variables and Spearman's rank correlation tests were used to determine associations. For all the statistical tests performed, $p<0.05$ was considered statistically significant.

REFERENCES

1. Siraj AK, Masoodi T, Bu R, Beg S, Al-Sobhi SS, Al-Dayel F, Al-Dawish M, Alkuraya FS, Al-Kuraya KS: **Genomic Profiling of Thyroid Cancer Reveals a Role for Thyroglobulin in Metastasis.** *Am J Hum Genet* 2016, **98:**1170-1180.
2. Siraj AK, Masoodi T, Bu R, Pratheeshkumar P, Al-Sanea N, Ashari LH, Abduljabbar A, Alhomoud S, Al-Dayel F, Alkuraya FS, Al-Kuraya KS: **MED12 is recurrently mutated in Middle Eastern colorectal cancer.** *Gut* 2018, **67:**663-671.
3. Shen R, Seshan VE: **FACETS: allele-specific copy number and clonal heterogeneity analysis tool for high-throughput DNA sequencing.** *Nucleic Acids Res* 2016, **44:**e131.
4. Carter SL, Cibulskis K, Helman E, McKenna A, Shen H, Zack T, Laird PW, Onofrio RC, Winckler W, Weir BA, et al: **Absolute quantification of somatic DNA alterations in human cancer.** *Nat Biotechnol* 2012, **30:**413-421.
5. Ng CKY, Bidard FC, Piscuoglio S, Geyer FC, Lim RS, de Bruijn I, Shen R, Pareja F, Berman SH, Wang L, et al: **Genetic Heterogeneity in Therapy-Naive Synchronous Primary Breast Cancers and Their Metastases.** *Clin Cancer Res* 2017, **23:**4402-4415.
6. Jamal-Hanjani M, Wilson GA, McGranahan N, Birkbak NJ, Watkins TBK, Veeriah S, Shafi S, Johnson DH, Mitter R, Rosenthal R, et al: **Tracking the Evolution of Non-Small-Cell Lung Cancer.** *N Engl J Med* 2017, **376:**2109-2121.
7. Cancer Genome Atlas Research N: **Integrated genomic analyses of ovarian carcinoma.** *Nature* 2011, **474:**609-615.
8. Corvigno S, Wisman GB, Mezheyeuski A, van der Zee AG, Nijman HW, Avall-Lundqvist E, Ostman A, Dahlstrand H: **Markers of fibroblast-rich tumor stroma and perivascular cells in serous ovarian cancer: Inter- and intra-patient heterogeneity and impact on survival.** *Oncotarget* 2016, **7:**18573-18584.
9. Bailey MH, Tokheim C, Porta-Pardo E, Sengupta S, Bertrand D, Weerasinghe A, Colaprico A, Wendl MC, Kim J, Reardon B, et al: **Comprehensive Characterization of Cancer Driver Genes and Mutations.** *Cell* 2018, **173:**371-385 e318.
10. Yang SYC, Lheureux S, Karakasis K, Burnier JV, Bruce JP, Clouthier DL, Danesh A, Quevedo R, Dowar M, Hanna Y, et al: **Landscape of genomic alterations in high-grade serous ovarian cancer from exceptional long- and short-term survivors.** *Genome Med* 2018, **10:**81.
11. Popic V, Salari R, Hajirasouliha I, Kashef-Haghighi D, West RB, Batzoglou S: **Fast and scalable inference of multi-sample cancer lineages.** *Genome Biol* 2015, **16:**91.
12. Yates LR, Knappskog S, Wedge D, Farmery JHR, Gonzalez S, Martincorena I, Alexandrov LB, Van Loo P, Haugland HK, Lilleng PK, et al: **Genomic Evolution of Breast Cancer Metastasis and Relapse.** *Cancer Cell* 2017, **32:**169-184 e167.

Supplementary Table S1: The quality control metrics of the targeted capture sequencing

##Abbreviations of column headers

##Sample type: Type of tissue, e.g Primary, metastasis

##Sequencing Type: sequencing strategy

##Total Bases: Total number of bases count

##Read Count: Total number of read count

##GC (%): percentage of GC bases

##AT (%): Percentage of AT bases

##Q20 (%): Yield of bases with Q20 or higher

##Q30 (%): Yield of bases with Q30 or higher

Sample	Sample Type	Sequencing Type	Total Bases	Read Count	GC (%)	AT (%)	Q20 (%)	Q30 (%)
OVA_003_C1	Primary	Whole Exome	11,36,11,68,728	7,52,39,528	51.24	48.76	96.20	94.37
OVA_003_C2	Primary	Whole Exome	9,60,66,69,528	6,36,20,328	51.02	48.98	96.42	94.61
OVA_003_C3	Primary	Whole Exome	11,89,12,38,222	7,87,49,922	50.94	49.06	96.26	94.40
OVA_003_M1	Metastasis	Whole Exome	10,66,13,39,296	7,06,04,896	50.14	49.86	96.36	94.60
OVA_003_M2	Metastasis	Whole Exome	11,41,84,30,646	7,56,18,746	50.09	49.91	96.14	94.32
OVA_003_M3	Metastasis	Whole Exome	9,87,47,22,916	6,53,95,516	48.82	51.18	96.48	94.69
OVA_003_N1	Normal	Whole Exome	11,08,59,83,912	7,34,17,112	50.29	49.71	96.58	94.79
OVA_013_C1	Primary	Whole Exome	10,62,27,81,748	7,03,49,548	50.61	49.39	96.66	94.93
OVA_013_C2	Primary	Whole Exome	10,84,59,57,332	7,18,27,532	51.45	48.55	96.64	94.88
OVA_013_C3	Primary	Whole Exome	10,31,84,00,176	6,83,33,776	50.51	49.49	96.37	94.53
OVA_013_M1	Metastasis	Whole Exome	8,78,79,64,742	5,81,98,442	49.29	50.71	96.56	94.78
OVA_013_N1	Normal	Whole Exome	9,35,62,69,550	6,19,62,050	50.13	49.87	96.26	94.37
OVA_047_C1	Primary	Whole Exome	9,52,86,28,802	6,31,03,502	50.67	49.33	96.58	94.79
OVA_047_C2	Primary	Whole Exome	10,99,83,54,384	7,28,36,784	49.91	50.09	96.54	94.74
OVA_047_C3	Contralateral ovary	Whole Exome	8,11,65,76,160	5,37,52,160	50.91	49.09	96.60	94.81
OVA_047_M1	Metastasis	Whole Exome	9,29,83,60,748	6,15,78,548	50.74	49.26	96.63	94.84
OVA_047_M2	Metastasis	Whole Exome	9,51,89,22,220	6,30,39,220	50.59	49.41	96.47	94.61
OVA_047_M3	Metastasis	Whole Exome	8,49,18,82,130	5,62,37,630	51.03	48.97	96.68	94.90
OVA_047_N1	Normal	Whole Exome	9,95,24,54,092	6,59,10,292	50.19	49.81	96.74	94.97
OVA_048_C1	Primary	Whole Exome	8,32,75,23,764	5,51,49,164	49.49	50.51	96.29	94.44
OVA_048_C2	Primary	Whole Exome	10,47,62,25,376	6,93,78,976	49.67	50.33	96.69	94.92
OVA_048_C3	Primary	Whole Exome	7,83,49,81,394	5,18,87,294	49.68	50.32	96.48	94.67
OVA_048_M1	Metastasis	Whole Exome	10,03,09,82,548	6,64,30,348	50.59	49.41	96.65	94.89
OVA_048_M2	Metastasis	Whole Exome	9,50,72,57,470	6,29,61,970	51.0	49.0	96.37	94.51
OVA_048_M3	Metastasis	Whole Exome	11,19,03,15,550	7,41,08,050	50.80	49.20	96.53	94.72
OVA_048_N1	Normal	Whole Exome	9,34,64,07,438	6,18,96,738	50.24	49.76	96.57	94.90
OVA_365_C1	Contralateral ovary	Whole Exome	9,76,15,30,296	6,46,45,896	50.05	49.95	96.15	94.31
OVA_365_C2	Primary	Whole Exome	10,55,19,70,600	6,98,80,600	49.98	50.02	96.32	94.53
OVA_365_M1	Metastasis	Whole Exome	9,13,72,70,324	6,05,11,724	49.06	50.94	96.25	94.43
OVA_365_M2	Metastasis	Whole Exome	9,86,13,48,846	6,53,06,946	49.05	50.95	96.60	94.91
OVA_365_M3	Metastasis	Whole Exome	11,14,61,15,434	7,38,15,334	49.0	51.0	96.64	94.90
OVA_365_N1	Normal	Whole Exome	10,68,08,47,590	7,07,34,090	51.37	48.63	96.59	94.82
OVA_378_C1	Primary	Whole Exome	8,79,86,31,080	5,82,69,080	50.59	49.41	95.60	93.41
OVA_378_C2	Primary	Whole Exome	10,39,59,70,688	6,88,47,488	50.14	49.86	96.53	94.73
OVA_378_C3	Primary	Whole Exome	10,04,08,74,256	6,64,95,856	50.54	49.46	96.10	94.15
OVA_378_M1	Metastasis	Whole Exome	12,59,78,20,676	8,34,29,276	50.88	49.12	96.15	94.21
OVA_378_M2	Metastasis	Whole Exome	10,38,50,52,784	6,87,75,184	50.82	49.18	96.43	94.60
OVA_378_M3	Metastasis	Whole Exome	8,71,68,99,310	5,77,27,810	50.66	49.34	95.94	93.93
OVA_378_N1	Normal	Whole Exome	11,33,54,74,266	7,50,69,366	50.90	49.10	96.47	94.63
OVA_003_C1	Primary	Targeted Capture	5,89,18,77,758	3,90,19,058	47.61	52.39	97.72	94.65
OVA_003_C2	Primary	Targeted Capture	6,32,42,06,160	4,18,82,160	48.33	51.67	97.73	94.71
OVA_003_C3	Primary	Targeted Capture	6,72,22,55,146	4,45,18,246	48.18	51.82	97.75	94.74
OVA_003_M1	Metastasis	Targeted Capture	4,79,78,42,592	3,17,73,792	47.42	52.58	97.74	94.8
OVA_003_M2	Metastasis	Targeted Capture	6,46,65,48,726	4,28,24,826	47.29	52.71	97.82	94.92
OVA_003_M3	Metastasis	Targeted Capture	6,51,25,81,378	4,31,29,678	47.5	52.5	97.84	94.89
OVA_013_C1	Primary	Targeted Capture	6,38,02,75,480	4,22,53,480	47.93	52.07	97.81	94.83
OVA_013_C2	Primary	Targeted Capture	5,77,19,46,310	3,82,24,810	48.5	51.5	97.83	94.85
OVA_013_C3	Primary	Targeted Capture	7,17,92,51,210	4,75,44,710	47.89	52.11	97.8	94.78
OVA_013_M1	Metastasis	Targeted Capture	5,29,77,36,850	3,50,84,350	46.84	53.16	97.61	94.53
OVA_047_C1	Primary	Targeted Capture	6,41,76,04,492	4,25,00,692	48.23	51.77	97.46	94.18
OVA_047_C2	Primary	Targeted Capture	6,80,92,55,004	4,50,94,404	48.16	51.84	97.66	94.54
OVA_047_C3	Contralateral ovary	Targeted Capture	6,08,94,16,864	4,03,27,264	47.95	52.05	97.61	94.42
OVA_047_M1	Metastasis	Targeted Capture	7,35,80,70,544	4,87,28,944	46.93	53.07	97.57	94.41
OVA_047_M2	Metastasis	Targeted Capture	7,05,52,24,944	4,67,23,344	47.83	52.17	97.52	94.29
OVA_047_M3	Metastasis	Targeted Capture	4,77,34,75,118	3,16,12,418	46.18	53.82	96.89	93.32
OVA_048_C1	Primary	Targeted Capture	4,70,69,37,874	3,11,71,774	48.11	51.89	96.78	93.07
OVA_048_C2	Primary	Targeted Capture	5,69,02,75,242	3,76,83,942	48.5	51.5	97.4	93.73
OVA_048_C3	Primary	Targeted Capture	5,09,51,54,948	3,37,42,748	48.95	51.05	96.8	93.02

Sample	Sample Type	Sequencing Type	Total Bases	Read Count	GC (%)	AT (%)	Q20 (%)	Q30 (%)
OVA_048_M1	Metastasis	Targeted Capture	5,09,24,37,854	3,37,24,754	49.26	50.74	97.27	93.43
OVA_048_M2	Metastasis	Targeted Capture	6,32,24,11,072	4,18,70,272	49.22	50.78	97.37	93.67
OVA_048_M3	Metastasis	Targeted Capture	6,04,44,67,788	4,00,29,588	49.38	50.62	97.43	93.8
OVA_365_C1	Contralateral ovary	Targeted Capture	6,15,51,37,500	4,07,62,500	48.64	51.36	97.19	93.68
OVA_365_C2	Primary	Targeted Capture	6,63,43,20,598	4,39,35,898	47.9	52.1	97.37	93.88
OVA_365_M1	Metastasis	Targeted Capture	5,68,27,65,106	3,76,34,206	46.87	53.13	98.42	96.19
OVA_365_M2	Metastasis	Targeted Capture	5,31,88,60,240	3,52,24,240	48.14	51.86	98.03	95.74
OVA_365_M3	Metastasis	Targeted Capture	5,13,13,37,870	3,39,82,370	47.37	52.63	98.5	96.29
OVA_378_C1	Primary	Targeted Capture	5,80,82,49,730	3,84,65,230	48.43	51.57	98.28	95.78
OVA_378_C2	Primary	Targeted Capture	5,36,53,77,300	3,55,32,300	48.31	51.69	97.75	94.36
OVA_378_C3	Primary	Targeted Capture	5,12,95,12,884	3,39,70,284	48.41	51.59	97.74	94.34
OVA_378_M1	Metastasis	Targeted Capture	5,01,73,34,380	3,32,27,380	48.87	51.13	97.62	94.11
OVA_378_M2	Metastasis	Targeted Capture	5,01,47,19,966	3,32,10,066	48.64	51.36	97.7	94.25
OVA_378_M3	Metastasis	Targeted Capture	5,66,27,08,682	3,75,01,382	48.03	51.97	97.61	94.06

Supplementary Table S2: Somatic mutations validated by target capture sequencing

##Abbreviations of column headers
##Sample: Unique tissue sample identifier
##Type: Tissue type primary, metastasis etc.
##Chr: Chromosome
##Start: hg19 start coordinate
##End: hg19 end coordinate
##Ref: Reference base
##Alt: Altered base
##Gene: Hugo gene symbol
##Func.ensGene: Ensembl gene function
##ExonicFunc.ensGene: Mutation type
##AAChange.ensGene: Amino acid change
##t_ref: Tumor, total read count of the reference base
##t_alt: Tumor, total read count of the altered base
##t_total: Tumor, Total read count
##t_VAF: Tumor, variant allele frequency
##n_ref: Normal, total read count of the reference base
##n_alt: Normal, total read count of the altered base
##n_total: Normal, total read count
##normal_VAF: Normal, variant allele frequency
##cancer_cell_frac: cancer cell fraction
##purity: Tumor purity
##ploidy: Tumor ploidy
##Cancer Gene: Whether the gene classified as cancer gene
##Driver Genes: Whether the gene classified as driver
##Mutation Taster: Classification of variants for their disease-causing potential
##CADD(PHRED): CADD is a tool for scoring the deleteriousness of single nucleotide variants as well as insertion/deletions variants in the human genome
##Protein_change: Protein change
##CHASM: "Cancer-specific High-throughput Annotation of Somatic Mutations" a method that predicts the functional significance of somatic missense mutations observed in the genomes of cancer cells
##FATHMM_Pred: "Functional Analysis through Hidden Markov Models" A high-throughput web-server capable of predicting the functional consequences of both coding variants and non-coding variants.
##POLYPHEN-2: (Polymorphism Phenotyping v2) is a tool which predicts possible impact of an amino acid substitution on the structure and function of a human protein using straightforward physical and comparative considerations
##PROVEAN PREDICTION (cutoff=-2.5): "Protein Variation Effect Analyzer" is a software tool which predicts whether an amino acid substitution or indel has an impact on the biological function of a protein
##SIFT PREDICTION (cutoff=0.05): SIFT predicts whether an amino acid substitution affects protein function based on sequence homology and the physical properties of amino acids

Sample	Type	Chr	Start	End	Ref	Alt	Gene	unc.ensGen	icFunc.ens	hange.ens	t_ref	t_alt	t_total	t_VAF	n_ref	n_alt	n_total	normal_VA	ncer_cell_f	on-based C	purity	ploidy	Cancer Gene	Driver Gene	Mutation Taster	ADD(PHRE	otein_chan	CHASM	ATHMM_Pr	OLYPHEN-	REDICTION	DICTION (cutoff=0.05)
OVA_047_M1	METASTASIS	chr17	13821	13821	C	T	DOC2B	intronic	NA	NA	37	179	216	0.828704	39	0	39	0	1	Clonal	0.47	2.01				5.871						
OVA_047_M2	METASTASIS	chr17	13821	13821	C	T	DOC2B	intronic	NA	NA	83	181	264	0.685606	39	0	39	0	1	Clonal	0.44	2.01				5.871						
OVA_047_M3	METASTASIS	chr17	13821	13821	C	T	DOC2B	intronic	NA	NA	25	73	98	0.744898	39	0	39	0	1	Clonal	0.6	3.02				5.871						
OVA_047_C1	PRIMARY	chr17	13821	13821	C	T	DOC2B	intronic	NA	NA	71	158	229	0.689956	39	0	39	0	1	Clonal	0.6	2.91				5.871						
OVA_047_C2	PRIMARY	chr17	13821	13821	C	T	DOC2B	intronic	NA	NA	72	195	267	0.730337	39	0	39	0	1	Clonal	0.64	2.76				5.871						
OVA_047_C3	Contralateral ovary	chr17	13821	13821	C	T	DOC2B	intronic	NA	NA	75	148	223	0.663677	39	0	39	0	1	Clonal	0.6	2.94				5.871						
OVA_047_M1	METASTASIS	chr11	281391	281391	G	T	NLRP6	exonic	ynonymou	A553S,ENSG	342	77	419	0.183771	76	0	76	0	0.96	Sub	0.47	2.01			polymorphism	11.12	p.A553S	Passenger	PASSENGE	benign	Neutral	Tolerated
OVA_047_M2	METASTASIS	chr11	281391	281391	G	T	NLRP6	exonic	ynonymou	A553S,ENSG	396	69	465	0.148387	76	0	76	0	0.82	Sub	0.44	2.01			polymorphism	11.12	p.A553S	Passenger	PASSENGE	benign	Neutral	Tolerated
OVA_047_M3	METASTASIS	chr11	281391	281391	G	T	NLRP6	exonic	ynonymou	A553S,ENSG	126	49	175	0.28	76	0	76	0	1	Sub	0.6	3.02			polymorphism	11.12	p.A553S	Passenger	PASSENGE	benign	Neutral	Tolerated
OVA_378_M2	METASTASIS	chr16	311971	312015	GTCATTGCA	-	TFG3;LUC7	intronic	NA	NA	11	128	139	0.920863	9	0	9		1	Clonal	0.85	2.57										
OVA_378_C1	PRIMARY	chr16	311971	312015	GTCATTGCA	-	TFG3;LUC7	intronic	NA	NA	31	192	223	0.860987	9	0	9		1	Clonal	0.59	2.6										
OVA_378_C2	PRIMARY	chr16	311971	312015	GTCATTGCA	-	TFG3;LUC7	intronic	NA	NA	12	118	130	0.907692	9	0	9		1	Clonal	0.56	2.59										
OVA_048_M1	METASTASIS	chr6	397137	397137	C	T	IRF4	exonic	ionymous S	L174L,ENSG	515	121	636	0.190252	96	0	96	0	1	Clonal	0.56	3.94	Yes		disease_causing	12.09		Passenger			Neutral	Tolerated
OVA_048_M2	METASTASIS	chr6	397137	397137	C	T	IRF4	exonic	ionymous S	L174L,ENSG	494	118	612	0.19281	96	0	96	0	1	Clonal	0.4	3.87	Yes		disease_causing	12.09		Passenger			Neutral	Tolerated
OVA_048_M3	METASTASIS	chr6	397137	397137	C	T	IRF4	exonic	ionymous S	L174L,ENSG	508	100	608	0.164474	96	0	96	0	1	Clonal	0.59	3.39	Yes		disease_causing	12.09		Passenger			Neutral	Tolerated
OVA_048_C1	PRIMARY	chr6	397137	397137	C	T	IRF4	exonic	ionymous S	L174L,ENSG	165	68	233	0.291845	96	0	96	0	0.87	Clonal	0.67	1.82	Yes		disease_causing	12.09		Passenger			Neutral	Tolerated
OVA_048_C3	PRIMARY	chr6	397137	397137	C	T	IRF4	exonic	ionymous S	L174L,ENSG	227	144	371	0.38814	96	0	96	0	1	Clonal	0.74	1.8	Yes		disease_causing	12.09		Passenger			Neutral	Tolerated
OVA_013_M1	METASTASIS	chr5	434944	434944	C	G	AHRR	exonic	ynonymou	47A,ENSG0	402	44	446	0.098655	20	0	20	0	0.69	Sub	0.67	3.06			polymorphism	15.28	p.P719A	Passenger	PASSENGE	damaging	Neutral	Damaging
OVA_013_C1	PRIMARY	chr5	434944	434944	C	G	AHRR	exonic	ynonymou	47A,ENSG0	496	85	581	0.146299	20	0	20	0	1	Sub	0.72	3.1			polymorphism	15.28	p.P719A	Passenger	PASSENGE	damaging	Neutral	Damaging
OVA_013_C2	PRIMARY	chr5	434944	434944	C	G	AHRR	exonic	ynonymou	47A,ENSG0	520	70	590	0.118644	20	0	20	0	0.75	Sub	0.87	3.05			polymorphism	15.28	p.P719A	Passenger	PASSENGE	damaging	Neutral	Damaging
OVA_013_C3	PRIMARY	chr5	434944	434944	C	G	AHRR	exonic	ynonymou	47A,ENSG0	500	104	604	0.172185	20	0	20	0	1	Sub	0.91	3.01			polymorphism	15.28	p.P719A	Passenger	PASSENGE	damaging	Neutral	Damaging
OVA_365_C1	Contralateral ovary	chr16	436623	436623	T	G	OC1001343	RNA_intro	NA	NA	117	54	171	0.315789	8	0	8	0	1	Clonal	0.42	1.83				0.84						
OVA_013_C2	PRIMARY	chr11	624806	624806	G	A	CDHR5	intronic	NA	NA	85	37	122	0.303279	37	0	37	0	1	Clonal	0.87	3.05			polymorphism	1.492						
OVA_013_M1	METASTASIS	chr20	628667	628667	C	T	SRXN1	UTR3	NA	NA	441	142	583	0.243568	49	1	50	0.02	0.97	Clonal	0.67	3.06			polymorphism	7.429						
OVA_013_C1	PRIMARY	chr20	628667	628667	C	T	SRXN1	UTR3	NA	NA	433	171	604	0.283113	49	1	50	0.02	1	Sub	0.72	3.1			polymorphism	7.429						
OVA_013_C2	PRIMARY	chr20	628667	628667	C	T	SRXN1	UTR3	NA	NA	439	142	581	0.244406	49	1	50	0.02	0.81	Sub	0.87	3.05			polymorphism	7.429						
OVA_013_C3	PRIMARY	chr20	628667	628667	C	T	SRXN1	UTR3	NA	NA	458	165	623	0.264848	49	1	50	0.02	0.84	Sub	0.91	3.01			polymorphism	7.429						
OVA_047_C1	PRIMARY	chr16	680057	680057	T	G	AK128777	eam;downs	NA	NA	133	65	198	0.328283	39	0	39	0	1	Clonal	0.6	2.91				5.733						
OVA_047_C2	PRIMARY	chr16	680057	680057	T	G	AK128777	eam;downs	NA	NA	123	51	174	0.293103	39	0	39	0	0.92	Clonal	0.64	2.76				5.733						
OVA_047_C3	Contralateral ovary	chr16	680057	680057	T	G	AK128777	eam;downs	NA	NA	120	45	165	0.272727	39	0	39	0	0.91	Clonal	0.6	2.94				5.733						
OVA_365_M1	METASTASIS	chr11	803587	803587	C	T	PIDD	exonic	ynonymou	G99E,ENSG	58	27	85	0.317647	33	0	33	0	0.75	Sub	0.85	1.87			disease_causing	24.9	p.G99E	Passenger	PASSENGE	damaging	Neutral	Damaging
OVA_365_M2	METASTASIS	chr11	803587	803587	C	T	PIDD	exonic	ynonymou	G99E,ENSG	41	9	50	0.18	33	0	33	0	0.89	Sub	0.4	1.81			disease_causing	24.9	p.G99E	Passenger	PASSENGE	damaging	Neutral	Damaging
OVA_365_M3	METASTASIS	chr11	803587	803587	C	T	PIDD	exonic	ynonymou	G99E,ENSG	45	40	85	0.470588	33	0	33	0	1	Sub	0.82	1.89			disease_causing	24.9	p.G99E	Passenger	PASSENGE	damaging	Neutral	Damaging
OVA_365_C1	Contralateral ovary	chr11	803587	803587	C	T	PIDD	exonic	ynonymou	G99E,ENSG	45	17	62	0.274194	33	0	33	0	1	Clonal	0.42	1.83			disease_causing	24.9	p.G99E	Passenger	PASSENGE	damaging	Neutral	Damaging
OVA_365_C2	PRIMARY	chr11	803587	803587	C	T	PIDD	exonic	ynonymou	G99E,ENSG	75	21	96	0.21875	33	0	33	0	0.71	Sub	0.62	1.92			disease_causing	24.9	p.G99E	Passenger	PASSENGE	damaging	Neutral	Damaging
OVA_047_M1	METASTASIS	chr17	882820	882820	C	A	NXN	exonic	ionymous S	T00000336	85	17	102	0.166667	66	0	66	0	0.54	Sub	0.47	2.01			disease_causing	21.1		Passenger			Neutral	Tolerated
OVA_048_M1	METASTASIS	chr5	893734	893741	GGGCCTGT	-	TRIP13	intronic	NA	NA	254	139	393	0.35369	46	0	46		1	Clonal	0.56	3.94										
OVA_048_M2	METASTASIS	chr5	893734	893741	GGGCCTGT	-	TRIP13	intronic	NA	NA	361	116	477	0.243187	46	0	46		1	Clonal	0.4	3.87										
OVA_048_M3	METASTASIS	chr5	893734	893741	GGGCCTGT	-	TRIP13	intronic	NA	NA	350	156	506	0.3083	46	0	46		1	Clonal	0.59	3.39										
OVA_003_M1	METASTASIS	chr18	908290	908290	C	G	ADCYAP1	exonic	ynonymou	N89K,ENSG	77	21	98	0.214286	72	0	72	0	0.76	Sub	0.57	1.84			disease_causing	20.2	p.N89K	Passenger	PASSENGE	damaging	Neutral	Damaging
OVA_003_M2	METASTASIS	chr18	908290	908290	C	G	ADCYAP1	exonic	ynonymou	N89K,ENSG	99	18	117	0.153846	72	0	72	0	0.67	Sub	0.46	1.81			disease_causing	20.2	p.N89K	Passenger	PASSENGE	damaging	Neutral	Damaging
OVA_003_M3	METASTASIS	chr18	908290	908290	C	G	ADCYAP1	exonic	ynonymou	N89K,ENSG	127	7	134	0.052239	72	0	72	0	0.55	Sub	0.34	2.67			disease_causing	20.2	p.N89K	Passenger	PASSENGE	damaging	Neutral	Damaging
OVA_003_C1	PRIMARY	chr18	908290	908290	C	G	ADCYAP1	exonic	ynonymou	N89K,ENSG	89	69	158	0.436709	72	0	72	0	1	Clonal	0.86	1.81			disease_causing	20.2	p.N89K	Passenger	PASSENGE	damaging	Neutral	Damaging
OVA_003_C2	PRIMARY	chr18	908290	908290	C	G	ADCYAP1	exonic	ynonymou	N89K,ENSG	119	59	178	0.331461	72	0	72	0	1	Clonal	0.7	3.61			disease_causing	20.2	p.N89K	Passenger	PASSENGE	damaging	Neutral	Damaging
OVA_003_C3	PRIMARY	chr18	908290	908290	C	G	ADCYAP1	exonic	ynonymou	N89K,ENSG	101	91	192	0.473958	72	0	72	0	1	Clonal	0.82	1.81			disease_causing	20.2	p.N89K	Passenger	PASSENGE	damaging	Neutral	Damaging
OVA_365_C1	Contralateral ovary	chr4	964894	964894	G	A	DGKQ	intronic	NA	NA	348	97	445	0.217978	134	0	134	0	1	Clonal	0.42	1.83			polymorphism	1.291						
OVA_047_C1	PRIMARY	chr4	983646	983646	C	G	SLC26A1	exonic	ynonymou	361Q,ENSG	324	90	414	0.217391	32	0	32	0	1	Clonal	0.6	2.91			disease_causing	24.9	p.E361Q	Passenger	PASSENGE	damaging	Neutral	Damaging
OVA_047_C2	PRIMARY	chr4	983646	983646	C	G	SLC26A1	exonic	ynonymou	361Q,ENSG	194	85	279	0.304659	32	0	32	0	0.95	Clonal	0.64	2.76			disease_causing	24.9	p.E361Q	Passenger	PASSENGE	damaging	Neutral	Damaging
OVA_047_C3	Contralateral ovary	chr4	983646	983646	C	G	SLC26A1	exonic	ynonymou	361Q,ENSG	297	56	353	0.15864	32	0	32	0	0.85	Clonal	0.6	2.94			disease_causing	24.9	p.E361Q	Passenger	PASSENGE	damaging	Neutral	Damaging
OVA_378_M1	METASTASIS	chr19	1007946	1007946	G	A	GRIN3B	exonic	ynonymou	000023438	117	62	179	0.346369	99	0	99	0	0.96	Clonal	0.72	2.57			disease_causing	23.7	p.V764M	Passenger	PASSENGE	damaging	Neutral	Damaging
OVA_378_M2	METASTASIS	chr19	1007946	1007946	G	A	GRIN3B	exonic	ynonymou	000023438	50	41	91	0.450549	99	0	99	0	1	Clonal	0.85	2.57			disease_causing	23.7	p.V764M	Passenger	PASSENGE	damaging	Neutral	Damaging
OVA_378_M3	METASTASIS	chr19	1007946	1007946	G	A	GRIN3B	exonic	ynonymou	000023438	123	55	178	0.308989	99	0	99	0	0.93	Clonal	0.67	2.57			disease_causing	23.7	p.V764M	Passenger	PASSENGE	damaging	Neutral	Damaging
OVA_378_C1	PRIMARY	chr19	1007946	1007946	G	A	GRIN3B	exonic	ynonymou	000023438	131	51	182	0.28022	99	0	99	0	0.95	Clonal	0.59	2.6			disease_causing	23.7	p.V764M	Passenger	PASSENGE	damaging	Neutral	Damaging
OVA_378_C2	PRIMARY	chr19	1007946	1007946	G	A	GRIN3B	exonic	ynonymou	000023438	89	35	124	0.282258	99	0	99	0	1	Clonal	0.56	2.59			disease_causing	23.7	p.V764M	Passenger	PASSENGE	damaging	Neutral	Damaging
OVA_378_C3	PRIMARY	chr19	1007946	1007946	G	A	GRIN3B	exonic	ynonymou	000023438	97	32	129	0.248062	99	0	99	0	0.97	Clonal	0.51	2.6			disease_causing	23.7	p.V764M	Passenger	PASSENGE	damaging	Neutral	Damaging
OVA_378_C3	PRIMARY	chr19	1010455	1010455	G	C	TMEM259	exonic	ynonymou	23R,ENSG0	12	4	16	0.25	87	0	87	0	0.98	Clonal	0.51	2.6			polymorphism	6.958	p.P586R	Passenger	no weights	benign	Neutral	Tolerated
OVA_047_C1	PRIMARY	chr1	1148866	1148866	G	T	TNFRSF4	intronic	NA	NA	94	51	145	0.351724	17	0	17	0	1	Clonal	0.6	2.91			polymorphism	0.588					Neutral	Damaging
OVA_047_C2	PRIMARY	chr1	1148866	1148866	G	T	TNFRSF4	intronic	NA	NA	60	39	99	0.393939	17	0	17	0	1	Clonal	0.64	2.76			polymorphism	0.588					Neutral	Damaging
OVA_047_C3	Contralateral ovary	chr1	1148866	1148866	G	T	TNFRSF4	intronic	NA	NA	53	38	91	0.417582	17	0	17	0	1	Clonal	0.6	2.94			polymorphism	0.588					Neutral	Damaging
OVA_013_M1	METASTASIS	chr5	1201794	1201794	G	T	SLC6A19	exonic	ynonymou	G10V,ENSG	148	35	183	0.191257	23	0	23	0	1	Clonal	0.67	3.06			disease_causing	23.3	p.G10V	Passenger	PASSENGE	damaging	Neutral	Damaging
OVA_013_C1	PRIMARY	chr5	1201794	1201794	G	T	SLC6A19	exonic	ynonymou	G10V,ENSG	256	46	302	0.152318	23	0	23	0	1	Clonal	0.72	3.1			disease_causing	23.3	p.G10V	Passenger	PASSENGE	damaging	Neutral	Damaging
OVA_013_C2	PRIMARY	chr5	1201794	1201794	G	T	SLC6A19	exonic	ynonymou	G10V,ENSG	203	42	245	0.171429	23	0	23	0	1	Clonal	0.87	3.05			disease_causing	23.3	p.G10V	Passenger	PASSENGE	damaging	Neutral	Damaging
OVA_013_C3	PRIMARY	chr5	1201794	1201794	G	T	SLC6A19	exonic	ynonymou	G10V,ENSG	265	55	320	0.171875	23	0	23	0	1	Clonal	0.91	3.01			disease_causing	23.3	p.G10V	Passenger	PASSENGE	damaging	Neutral	Damaging
OVA_047_M1	METASTASIS	chr1	1225978	1225978	C	G	SCNN1D	intronic	NA	NA	111	56	167	0.335329	90	0	90	0	1	Clonal	0.47	2.01			polymorphism	0.08						
OVA_047_M2	METASTASIS	chr1	1225978	1225978	C	G	SCNN1D	intronic	NA	NA	168	56	224	0.25	90	0	90	0	0.88	Clonal	0.44	2.01			polymorphism	0.08						
OVA_047_M3	METASTASIS	chr1	1225978	1225978	C	G	SCNN1D	intronic	NA	NA	42	21	63	0.333333	90	0	90	0	1	Clonal	0.6	3.02			polymorphism	0.08						

Sample	Type	Chr	Start	End	Ref	Alt	Gene	unc.ensGen	icFunc.ens	hange.ens	t_ref	t_alt	t_total	t_VAF	n_ref	n_alt	n_total	normal_VA	ncer_cell_f	on-based C	purity	ploidy	Cancer Gene	Driver Gene	Mutation Taster	ADD(PHRE	otein_chan	CHASM	ATHMM_Pr	OLYPHEN-	REDICTION	ICTION (cutoff=0.05)
OVA_013_M1	METASTASIS	chr16	1250469	1250469	C	G	CACNA1H	exonic	ynonymous	000034826	205	103	308	0.334416	70	0	70	0	1	Clonal	0.67	3.06			disease_causing	26.5	p.N339K	Passenger	PASSENGE	damaging	Deleterious	Damaging
OVA_013_C1	PRIMARY	chr16	1250469	1250469	C	G	CACNA1H	exonic	ynonymous	000034826	304	218	522	0.417625	70	0	70	0	1	Clonal	0.72	3.1			disease_causing	26.5	p.N339K	Passenger	PASSENGE	damaging	Deleterious	Damaging
OVA_013_C2	PRIMARY	chr16	1250469	1250469	C	G	CACNA1H	exonic	ynonymous	000034826	296	224	520	0.430769	70	0	70	0	1	Clonal	0.87	3.05			disease_causing	26.5	p.N339K	Passenger	PASSENGE	damaging	Deleterious	Damaging
OVA_013_C3	PRIMARY	chr16	1250469	1250469	C	G	CACNA1H	exonic	ynonymous	000034826	308	240	548	0.437956	70	0	70	0	1	Clonal	0.91	3.01			disease_causing	26.5	p.N339K	Passenger	PASSENGE	damaging	Deleterious	Damaging
OVA_013_M1	METASTASIS	chr19	1257170	1257170	G	A	MIDN	exonic	ynonymous	436S,ENSG	115	61	176	0.346591	74	0	74	0	1	Clonal	0.67	3.06			polymorphism	2.776	p.G436S	Passenger	no weights	benign	Neutral	Tolerated
OVA_013_C1	PRIMARY	chr19	1257170	1257170	G	A	MIDN	exonic	ynonymous	436S,ENSG	92	72	164	0.439024	74	0	74	0	1	Clonal	0.72	3.1			polymorphism	2.776	p.G436S	Passenger	no weights	benign	Neutral	Tolerated
OVA_013_C2	PRIMARY	chr19	1257170	1257170	G	A	MIDN	exonic	ynonymous	436S,ENSG	81	57	138	0.413043	74	0	74	0	1	Clonal	0.87	3.05			polymorphism	2.776	p.G436S	Passenger	no weights	benign	Neutral	Tolerated
OVA_013_C3	PRIMARY	chr19	1257170	1257170	G	A	MIDN	exonic	ynonymous	436S,ENSG	98	75	173	0.433526	74	0	74	0	0.95	Clonal	0.91	3.01			polymorphism	2.776	p.G436S	Passenger	no weights	benign	Neutral	Tolerated
OVA_047_C1	PRIMARY	chr20	1286615	1286615	G	A	SNPH	exonic	ynonymous	468T,ENSG	231	42	273	0.153846	26	0	26	0	1	Sub	0.6	2.91			disease_causing	28.7	p.A512T	Passenger		damaging	Neutral	Damaging
OVA_047_C2	PRIMARY	chr20	1286615	1286615	G	A	SNPH	exonic	ynonymous	468T,ENSG	204	27	231	0.116883	26	0	26	0	0.72	Sub	0.64	2.76			disease_causing	28.7	p.A512T	Passenger		damaging	Neutral	Damaging
OVA_047_C3	Contralateral ovary	chr20	1286615	1286615	G	A	SNPH	exonic	ynonymous	468T,ENSG	202	31	233	0.133047	26	0	26	0	0.98	Clonal	0.6	2.94			disease_causing	28.7	p.A512T	Passenger		damaging	Neutral	Damaging
OVA_048_C2	PRIMARY	chr5	1470908	1470908	C	G	LPCAT1	intronic	NA	NA	426	103	529	0.194707	41	0	41	0	0.74	Sub	0.72	1.85			polymorphism	1.21						
OVA_365_M1	METASTASIS	chr4	1659302	1659302	C	A	FAM53A	intronic	NA	NA	230	203	433	0.468822	38	0	38	0	1	Clonal	0.85	1.87			polymorphism	4.753						
OVA_048_M1	METASTASIS	chr1	1747328	1747328	T	C	GNB1	intronic	NA	NA	505	198	703	0.28165	53	0	53	0	1	Clonal	0.56	3.94			polymorphism	6.093						
OVA_048_M2	METASTASIS	chr1	1747328	1747328	T	C	GNB1	intronic	NA	NA	521	162	683	0.237189	53	0	53	0	1	Clonal	0.4	3.87			polymorphism	6.093						
OVA_048_M3	METASTASIS	chr1	1747328	1747328	T	C	GNB1	intronic	NA	NA	512	143	655	0.218321	53	0	53	0	1	Clonal	0.59	3.39			polymorphism	6.093						
OVA_003_M1	METASTASIS	chr16	1831274	1831274	C	T	SPSB3	UTR3	NA	NA	390	157	547	0.28702	142	0	142	0	1	Sub	0.57	1.84			polymorphism	5.968				damaging		
OVA_003_M2	METASTASIS	chr16	1831274	1831274	C	T	SPSB3	UTR3	NA	NA	451	118	569	0.207381	142	0	142	0	0.9	Sub	0.46	1.81			polymorphism	5.968				damaging		
OVA_003_M3	METASTASIS	chr16	1831274	1831274	C	T	SPSB3	UTR3	NA	NA	585	42	627	0.066986	142	0	142	0	0.71	Sub	0.34	2.67			polymorphism	5.968				damaging		
OVA_003_C1	PRIMARY	chr16	1831274	1831274	C	T	SPSB3	UTR3	NA	NA	350	264	614	0.429967	142	0	142	0	1	Sub	0.86	1.81			polymorphism	5.968				damaging		
OVA_003_C2	PRIMARY	chr16	1831274	1831274	C	T	SPSB3	UTR3	NA	NA	366	247	613	0.402936	142	0	142	0	1	Sub	0.7	3.61			polymorphism	5.968				damaging		
OVA_003_C3	PRIMARY	chr16	1831274	1831274	C	T	SPSB3	UTR3	NA	NA	409	227	636	0.356918	142	0	142	0	0.87	Sub	0.82	1.81			polymorphism	5.968				damaging		
OVA_047_C1	PRIMARY	chr12	1863662	1863662	A	G	ADIPOR2	exonic	onymous S	000003571	496	129	625	0.2064	31	0	31	0	1	Clonal	0.6	2.91			polymorphism	0.787		Passenger			Neutral	Tolerated
OVA_047_C2	PRIMARY	chr12	1863662	1863662	A	G	ADIPOR2	exonic	onymous S	000003571	486	121	607	0.199341	31	0	31	0	1	Clonal	0.64	2.76			polymorphism	0.787		Passenger			Neutral	Tolerated
OVA_047_C3	Contralateral ovary	chr12	1863662	1863662	A	G	ADIPOR2	exonic	onymous S	000003571	490	127	617	0.205835	31	0	31	0	1	Clonal	0.6	2.94			polymorphism	0.787		Passenger			Neutral	Tolerated
OVA_047_C1	PRIMARY	chr1	1888076	1888076	G	-	KIAA1751	exonic	unknown	UNKNOWN	376	145	521	0.278311	73	0	73		1	Sub	0.6	2.91					p.Q667Sfs	Passenger			NA	NA
OVA_047_C2	PRIMARY	chr1	1888076	1888076	G	-	KIAA1751	exonic	unknown	UNKNOWN	441	134	575	0.233043	73	0	73		0.73	Sub	0.64	2.76					p.Q667Sfs	Passenger			NA	NA
OVA_047_C3	Contralateral ovary	chr1	1888076	1888076	G	-	KIAA1751	exonic	unknown	UNKNOWN	336	139	475	0.292632	73	0	73		0.98	Clonal	0.6	2.94					p.Q667Sfs	Passenger			NA	NA
OVA_047_M1	METASTASIS	chr19	1986631	1986631	G	A	BTBD2	exonic	onymous S	000025560	143	32	175	0.182857	54	0	54	0	0.59	Sub	0.47	2.01			disease_causing	16.17		Passenger			Neutral	Tolerated
OVA_003_C1	PRIMARY	chr16	2042891	2042891	C	T	SYNGR3	exonic	ynonymous	170W,ENSG	155	11	166	0.066265	104	0	104	0	0.15	Sub	0.86	1.81			disease_causing	24.9	p.R170W	Passenger	SENGER/OT	damaging	Deleterious	NA
OVA_003_C2	PRIMARY	chr16	2042891	2042891	C	T	SYNGR3	exonic	ynonymous	170W,ENSG	214	18	232	0.077586	104	0	104	0	0.38	Sub	0.7	3.61			disease_causing	24.9	p.R170W	Passenger	SENGER/OT	damaging	Deleterious	NA
OVA_013_M1	METASTASIS	chr16	2070481	2070481	G	T	NPW	intronic	NA	NA	58	24	82	0.292683	48	1	49	0.020408	1	Clonal	0.67	3.06			polymorphism	6.239						
OVA_013_C1	PRIMARY	chr16	2070481	2070481	G	T	NPW	intronic	NA	NA	47	34	81	0.419753	48	1	49	0.020408	1	Clonal	0.72	3.1			polymorphism	6.239						
OVA_013_C2	PRIMARY	chr16	2070481	2070481	G	T	NPW	intronic	NA	NA	46	39	85	0.458824	48	1	49	0.020408	1	Clonal	0.87	3.05			polymorphism	6.239						
OVA_013_C3	PRIMARY	chr16	2070481	2070481	G	T	NPW	intronic	NA	NA	64	40	104	0.384615	48	1	49	0.020408	1	Clonal	0.91	3.01			polymorphism	6.239						
OVA_003_M1	METASTASIS	chr19	2343980	2343980	G	A	SPPL2B	cRNA_exon	NA	NA	42	36	78	0.461538	18	0	18	0	1	Sub	0.57	1.84				14.89		Passenger			Neutral	Tolerated
OVA_003_M2	METASTASIS	chr19	2343980	2343980	G	A	SPPL2B	cRNA_exon	NA	NA	63	34	97	0.350515	18	0	18	0	1	Sub	0.46	1.81				14.89		Passenger			Neutral	Tolerated
OVA_003_M3	METASTASIS	chr19	2343980	2343980	G	A	SPPL2B	cRNA_exon	NA	NA	235	3	238	0.012605	18	0	18	0	0.1	Sub	0.34	2.67				14.89		Passenger			Neutral	Tolerated
OVA_003_C1	PRIMARY	chr19	2343980	2343980	G	A	SPPL2B	cRNA_exon	NA	NA	17	72	89	0.808989	18	0	18	0	1	Clonal	0.86	1.81				14.89		Passenger			Neutral	Tolerated
OVA_003_C2	PRIMARY	chr19	2343980	2343980	G	A	SPPL2B	cRNA_exon	NA	NA	19	92	111	0.828829	18	0	18	0	1	Clonal	0.7	3.61				14.89		Passenger			Neutral	Tolerated
OVA_003_C3	PRIMARY	chr19	2343980	2343980	G	A	SPPL2B	cRNA_exon	NA	NA	22	97	119	0.815126	18	0	18	0	1	Clonal	0.82	1.81				14.89		Passenger			Neutral	Tolerated
OVA_378_C2	PRIMARY	chr16	2374586	2374586	A	G	ABCA3	intronic	NA	NA	300	24	324	0.074074	76	0	76	0	0.26	Sub	0.56	2.59			polymorphism	0.334						
OVA_048_M1	METASTASIS	chr11	2415235	2415274	GCACCCAC	-	CD81	exonic	neshift dele	00481687:	142	88	230	0.382609	72	0	72		1	Clonal	0.56	3.94					p.P38Afs	Passenger			NA	NA
OVA_048_M2	METASTASIS	chr11	2415235	2415274	GCACCCAC	-	CD81	exonic	neshift dele	00481687:	141	70	211	0.331754	72	0	72		1	Clonal	0.4	3.87					p.P38Afs	Passenger			NA	NA
OVA_048_M3	METASTASIS	chr11	2415235	2415274	GCACCCAC	-	CD81	exonic	neshift dele	00481687:	178	88	266	0.330827	72	0	72		1	Clonal	0.59	3.39					p.P38Afs	Passenger			NA	NA
OVA_048_C1	PRIMARY	chr11	2415235	2415274	GCACCCAC	-	CD81	exonic	neshift dele	00481687:	180	51	231	0.220779	72	0	72		0.44	Sub	0.72	1.85					p.P38Afs	Passenger			NA	NA
OVA_048_C2	PRIMARY	chr11	2415235	2415274	GCACCCAC	-	CD81	exonic	neshift dele	00481687:	157	66	223	0.295964	72	0	72		0.53	Sub	0.72	1.85					p.P38Afs	Passenger			NA	NA
OVA_048_C3	PRIMARY	chr11	2415235	2415274	GCACCCAC	-	CD81	exonic	neshift dele	00481687:	77	29	106	0.273585	72	0	72		0.47	Sub	0.74	1.8					p.P38Afs	Passenger			NA	NA
OVA_003_C1	PRIMARY	chr19	2767594	2767594	G	A	SGTA	exonic	ynonymous	000005878	207	48	255	0.188235	60	0	60	0	0.25	Sub	0.86	1.81			disease_causing	18.13	p.A64V	Passenger	SENGER/OT	damaging	Neutral	Tolerated
OVA_003_C2	PRIMARY	chr19	2767594	2767594	G	A	SGTA	exonic	ynonymous	000005878	231	43	274	0.156934	60	0	60	0	0.45	Sub	0.7	3.61			disease_causing	18.13	p.A64V	Passenger	SENGER/OT	damaging	Neutral	Tolerated
OVA_378_C1	PRIMARY	chr16	2790255	2790255	G	A	SRRM2-AS1	cRNA_exon	NA	NA	119	44	163	0.269939	72	0	72	0	0.92	Clonal	0.59	2.6				3.255					Deleterious	NA
OVA_365_M1	METASTASIS	chr11	2924658	2924658	T	-	SLC22A18	exonic	neshift dele	000003479	242	174	416	0.418269	147	0	147		0.99	Clonal	0.85	1.87					p.T29Pfs	Passenger			NA	NA
OVA_365_M2	METASTASIS	chr11	2924658	2924658	T	-	SLC22A18	exonic	neshift dele	000003479	191	53	244	0.217213	147	0	147		1	Clonal	0.4	1.81					p.T29Pfs	Passenger			NA	NA
OVA_365_M3	METASTASIS	chr11	2924658	2924658	T	-	SLC22A18	exonic	neshift dele	000003479	155	109	264	0.412879	147	0	147		1	Clonal	0.82	1.89					p.T29Pfs	Passenger			NA	NA
OVA_365_C1	Contralateral ovary	chr11	2924658	2924658	T	-	SLC22A18	exonic	neshift dele	000003479	257	77	334	0.230539	147	0	147		1	Clonal	0.42	1.83					p.T29Pfs	Passenger			NA	NA
OVA_365_C2	PRIMARY	chr11	2924658	2924658	T	-	SLC22A18	exonic	neshift dele	000003479	251	114	365	0.312329	147	0	147		1	Clonal	0.62	1.92					p.T29Pfs	Passenger			NA	NA
OVA_048_M1	METASTASIS	chr18	2960707	2960707	C	G	LPIN2	exonic	ynonymous	000005842	327	265	592	0.447635	87	0	87	0	1	Clonal	0.56	3.94			disease_causing	25.3	p.Q44H	Passenger	SENGER/OT	damaging	Deleterious	Damaging
OVA_048_M3	METASTASIS	chr18	2960707	2960707	C	G	LPIN2	exonic	ynonymous	000005842	391	220	611	0.360065	87	0	87	0	1	Clonal	0.59	3.39			disease_causing	25.3	p.Q44H	Passenger	SENGER/OT	damaging	Deleterious	Damaging
OVA_048_M2	METASTASIS	chr17	2995529	2995529	T	A	OR1D2	exonic	onymous S	000003314	445	106	551	0.192377	40	0	40	0	0.96	Clonal	0.4	3.87			disease_causing	6.482		Passenger			Neutral	Tolerated
OVA_048_M3	METASTASIS	chr17	2995529	2995529	T	A	OR1D2	exonic	onymous S	000003314	214	286	500	0.572	40	0	40	0	1	Clonal	0.59	3.39			disease_causing	6.482		Passenger			Neutral	Tolerated
OVA_013_C1	PRIMARY	chr19	2997966	2997966	G	A	TLE2	intronic	NA	NA	474	32	506	0.063241	10	0	10	0	0.24	Sub	0.72	3.1			polymorphism	1.909						
OVA_365_M1	METASTASIS	chr8	3038703	3038703	G	A	CSMD1	exonic	ynonymous	000400186	322	205	527	0.388994	40	0	40	0	0.92	Sub	0.85	1.87			disease_causing	27.9	p.S1886F	Passenger	SENGER/OT	damaging	Deleterious	Damaging
OVA_365_M2	METASTASIS	chr8	3038703	3038703	G	A	CSMD1	exonic	ynonymous	000400186	362	92	454	0.202643	40	0	40	0	0.8	Sub	0.4	1.81			disease_causing	27.9	p.S1886F	Passenger	SENGER/OT	damaging	Deleterious	Damaging
OVA_365_M3	METASTASIS	chr8	3038703	3038703	G	A	CSMD1	exonic	ynonymous	000400186	321	203	524	0.387405	40	0	40	0	0.95	Sub	0.82	1.89			disease_causing	27.9	p.S1886F	Passenger	SENGER/OT	damaging	Deleterious	Damaging
OVA_365_C1	Contralateral ovary	chr8	3038703	3038703	G	A	CSMD1	exonic	ynonymous	000400186	369	87	456	0.190789	40	0	40	0	0.92	Clonal	0.42	1.83			disease_causing	27.9	p.S1886F	Passenger	SENGER/OT	damaging	Deleterious	Damaging
OVA_365_C2	PRIMARY	chr8	3038703	3038703	G	A	CSMD1	exonic	ynonymous	000400186	254	207	461	0.449024	40	0	40	0	1	Clonal	0.62	1.92			disease_causing	27.9	p.S1886F	Passenger	SENGER/OT	damaging	Deleterious	Damaging
OVA_003_C2	PRIMARY	chr16	3076904	3076904	T	C	THOC6	exonic	ynonymous	208A,ENSG	545	65	610	0.106557	139	0	139	0	0.52	Sub	0.7	3.61			disease_causing	22.4	p.V208A	Passenger	CANCER	damaging	Neutral	NA
OVA_047_C1	PRIMARY	chr16	3086610	3086610	C	A	OC1001287	cRNA_exon	NA	NA	42	9	51	0.176471	23	0	23	0	0.59	Sub	0.6	2.91			polymorphism	6.772					Neutral	Tolerated
OVA_047_M1	METASTASIS	chr20	3214965	3214965	G	C	SLC4A11	intronic	NA	NA	302	75	377	0.198939	37	0	37	0	1	Clonal	0.47	2.01			disease_causing	10.46						
OVA_047_M2	METASTASIS	chr20	3214965	3214965	G	C	SLC4A11	intronic	NA	NA	311	58	369	0.157182	37	0	37	0	0.87	Clonal	0.44	2.01			disease_causing	10.46						
OVA_047_M3	METASTASIS	chr20	3214965	3214965	G	C	SLC4A11	intronic	NA	NA	129	22	151	0.145695	37	0	37	0	0.78	Clonal	0.6	3.02			disease_causing	10.46						
OVA_365_M1	METASTASIS	chr20	3219711	3219711	C	T	SLC4A11	UTR5	NA	NA	12	6	18	0.333333	39	0	39	0	0.79	Clonal	0.85	1.87			polymorphism	8.685						
OVA_365_M2	METASTASIS	chr20	3219711	3219711	C	T	SLC4A11	UTR5	NA	NA	12	2	14	0.142857	39	0	39	0	0.71	Clonal	0.85	1.87			polymorphism	8.685						
OVA_365_M3	METASTASIS	chr20	3219711	3219711	C	T	SLC4A11	UTR5	NA	NA	5	7	12	0.583333	39	0	39	0	1	Clonal	0.82	1.89			polymorphism	8.685						
OVA_365_C1	Contralateral ovary	chr20	3219711	3219711	C	T	SLC4A11	UTR5	NA	NA	12	6	18	0.333333	39	0	39	0	1	Clonal	0.85	1.87			polymorphism	8.685						
OVA_365_C2	PRIMARY	chr20	3219711	3219711	C	T	SLC4A11	UTR5	NA	NA	8	10	18	0.555556	39	0	39	0	1	Clonal	0.82	1.89			polymorphism	8.685						
OVA_048_C2	PRIMARY	chr4	3419104	3419104	T	C	RGS12	intronic	NA	NA	331	114	445	0.25618	35	0	35	0	0.71	Sub	0.72	1.85			polymorphism	13.58						
OVA_047_C1	PRIMARY	chr18	3499357	3499357	C	T	DLGAP1	exonic	onymous S	000584874	119	35	154	0.227273	55	0	55	0	0.76	Sub	0.6	2.91			disease_causing	13.8		Passenger			Neutral	Tolerated
OVA_378_M1	METASTASIS	chr1	3703673	3703673	C	T	LRRC47	exonic	ynonymous	000037825	249	76	325	0.233846	55	0	55	0	1	Clonal	0.72	2.57			polymorphism	13.92	p.E273K	Passenger	SENGER/OT	damaging	Neutral	Tolerated
OVA_378_M2	METASTASIS	chr1	3703673	3703673	C	T	LRRC47	exonic	ynonymous	000037825	212	72	284	0.253521	55	0	55	0	1	Clonal	0.85	2.57			polymorphism	13.92	p.E273K	Passenger	SENGER/OT	damaging	Neutral	Tolerated
OVA_378_M3	METASTASIS	chr1	3703673	3703673	C	T	LRRC47	exonic	ynonymous	000037825	296	95	391	0.242967	55	0	55	0	1	Clonal	0.67	2.57			polymorphism	13.92	p.E273K	Passenger	SENGER/OT	damaging	Neutral	Tolerated
OVA_378_C1	PRIMARY	chr1	3703673	3703673	C	T	LRRC47	exonic	ynonymous	000037825	349	90	439	0.205011	55	0	55	0	1	Sub	0.59	2.6			polymorphism	13.92	p.E273K	Passenger	SENGER/OT	damaging	Neutral	Tolerated
OVA_378_C2	PRIMARY	chr1	3703673	3703673	C	T	LRRC47	exonic	ynonymous	000037825	241	52	293	0.177474	55	0	55	0	0.98	Sub	0.56	2.59			polymorphism	13.92	p.E273K	Passenger	SENGER/OT	damaging	Neutral	Tolerated
OVA_378_C3	PRIMARY	chr1	3703673	3703673	C	T	LRRC47	exonic	ynonymous	000037825	198	21	219	0.09589	55	0	55	0	0.47	Sub	0.51	2.6			polymorphism	13.92	p.E273K	Passenger	SENGER/OT	damaging	Neutral	Tolerated
OVA_048_M2	METASTASIS	chr16	3711948	3711948	G	A	BC095475	intronic	NA	NA	215	16	231	0.069264	49	0	49	0	0.34	Sub	0.4	3.87			polymorphism	0.878						
OVA_047_C1	PRIMARY	chr2	3743377	3743377	A	C	ALLC	exonic	ynonymous	000025250	541	107	648	0.165123	67	0	67	0	0.72	Sub	0.6	2.91			polymorphism	6.639	p.E194D	Passenger	no weights	benign	Neutral	Tolerated
OVA_047_M1	METASTASIS	chr18	3879149	3879149	T	A	DLGAP1	exonic	ynonymous	307V,ENSG	495	136	631	0.215531	153	1	154	0.006494	0.91	Sub	0.47	2.01			disease_causing	24	p.E307V	Passenger	CANCER	damaging	Deleterious	Damaging
OVA_047_M2	METASTASIS	chr18	3879149	3879149	T	A	DLGAP1	exonic	ynonymous	307V,ENSG	524	124	648	0.191358	153	1	154	0.006494	0.86	Sub	0.44	2.01			disease_causing	24	p.E307V	Passenger	CANCER	damaging	Deleterious	Damaging
OVA_047_M3	METASTASIS	chr18	3879149	3879149	T	A	DLGAP1	exonic	ynonymous	307V,ENSG	345	48	393	0.122137	153	1	154	0.006494	0.53	Sub	0.6	3.02			disease_causing	24	p.E307V	Passenger	CANCER	damaging	Deleterious	Damaging
OVA_047_C2	PRIMARY	chr7	4014053	4014053	A	G	SDK1	exonic	ynonymous	I624V,ENSG	567	30	597	0.050251	69	0	69	0	0.16	Sub	0.64	2.76			polymorphism	6.159	p.I624V	Passenger	SENGER/OT	benign	Neutral	Tolerated
OVA_048_M1	METASTASIS	chr6	4032096	4032096	G	A	PRPF4B	exonic	onymous S	000004800	456	166	622	0.266881	49	0	49	0	1	Clonal	0.56	3.94			disease_causing	11.76		Passenger			Neutral	Tolerated
OVA_048_M2	METASTASIS	chr6	4032096	4032096	G	A	PRPF4B	exonic	onymous S	000004800	461	142	603	0.235489	49	0	49	0	1	Clonal	0.4	3.87			disease_causing	11.76		Passenger			Neutral	Tolerated
OVA_048_M3	METASTASIS	chr6	4032096	4032096	G	A	PRPF4B	exonic	onymous S	000004800	470	120	590	0.20339	49	0	49	0	1	Clonal	0.59	3.39			disease_causing	11.76		Passenger			Neutral	Tolerated
OVA_048_C1	PRIMARY	chr6	4032096	4032096	G	A	PRPF4B	exonic	onymous S	000004800	299	145	444	0.326577	49	0	49	0	0.98	Clonal	0.67	1.82			disease_causing	11.76		Passenger			Neutral	Tolerated
OVA_048_C3	PRIMARY	chr6	4032096	4032096	G	A	PRPF4B	exonic	onymous S	000004800	294	203	497	0.408451	49	0	49	0	1	Clonal	0.74	1.8			disease_causing	11.76		Passenger			Neutral	Tolerated
OVA_003_M1	METASTASIS	chr16	4385027	4385027	C	T	GLIS2	intronic	NA	NA	121	53	174	0.304598	90	0	90	0	1	Clonal	0.57	1.84			polymorphism	4.169						
OVA_003_M2	METASTASIS	chr16	4385027	4385027	C	T	GLIS2	intronic	NA	NA	183	66	249	0.26506	90	0	90	0	1	Clonal	0.46	1.81			polymorphism	4.169						
OVA_003_M3	METASTASIS	chr16	4385027	4385027	C	T	GLIS2	intronic	NA	NA	191	20	211	0.094787	90	0	90	0	1	Clonal	0.34	2.67			polymorphism	4.169						
OVA_003_C1	PRIMARY	chr16	4385027	4385027	C	T	GLIS2	intronic	NA	NA	157	135	292	0.462329	90	0	90	0	1	Clonal	0.86	1.81			polymorphism	4.169						
OVA_003_C2	PRIMARY	chr16	4385027	4385027	C	T	GLIS2	intronic	NA	NA	127	125	252	0.496032	90	0	90	0	1	Clonal	0.7	3.61			polymorphism	4.169						

Sample	Type	Chr	Start	End	Ref	Alt	Gene	unc.ensGen	icFunc.ens	hange.ens	t_ref	t_alt	t_total	t_VAF	n_ref	n_alt	n_total	normal_VA	ncer_cell_f	on-based C	purity	ploidy	Cancer Gene	Driver Gene	Mutation Taster	ADD(PHRE	otein_chan	CHASM	ATHMM_Pr	OLYPHEN-	REDICTION	ICTION (cutoff=0.05)
OVA_003_C3	PRIMARY	chr16	4385027	4385027	C	T	GLIS2	intronic	NA	NA	180	129	309	0.417476	90	0	90	0	1	Clonal	0.82	1.81			polymorphism	4.169						
OVA_048_M3	METASTASIS	chr16	4414664	4414664	G	A	7;CORO7-P	exonic	nonymous S	0251166:ex	59	22	81	0.271605	17	0	17	0	1	Clonal	0.59	3.39			disease_causing	10.47		Passenger			Neutral	Tolerated
OVA_013_C3	PRIMARY	chr16	4511864	4511864	C	T	NMRAL1	exonic	ynonymou	273K,ENSG	389	135	524	0.257634	63	0	63	0	0.82	Sub	0.91	3.01			disease_causing	17.47	p.E273K	Passenger	SENGER/OT	damaging	Neutral	Tolerated
OVA_003_M1	METASTASIS	chr20	4713086	4713086	T	C	PRNT	exonic	nonymous S	000004185	1198	17	1215	0.013992	23	0	23	0	0.05	Sub	0.57	1.84			polymorphism	0.008		Passenger			Neutral	Tolerated
OVA_003_C1	PRIMARY	chr20	4713086	4713086	T	C	PRNT	exonic	nonymous S	000004185	366	277	643	0.430793	23	0	23	0	1	Clonal	0.86	1.81			polymorphism	0.008		Passenger			Neutral	Tolerated
OVA_003_C2	PRIMARY	chr20	4713086	4713086	T	C	PRNT	exonic	nonymous S	000004185	512	117	629	0.18601	23	0	23	0	0.9	Clonal	0.7	3.61			polymorphism	0.008		Passenger			Neutral	Tolerated
OVA_003_C3	PRIMARY	chr20	4713086	4713086	T	C	PRNT	exonic	nonymous S	000004185	429	273	702	0.388889	23	0	23	0	0.94	Clonal	0.82	1.81			polymorphism	0.008		Passenger			Neutral	Tolerated
OVA_047_M1	METASTASIS	chr12	4763714	4763714	T	C	NDUFA9	intronic	NA	NA	651	20	671	0.029806	73	0	73	0	0.13	Sub	0.47	2.01			polymorphism	9.016						
OVA_047_M2	METASTASIS	chr12	4763714	4763714	T	C	NDUFA9	intronic	NA	NA	564	142	706	0.201133	73	0	73	0	1	Sub	0.44	2.01			polymorphism	9.016						
OVA_047_M3	METASTASIS	chr12	4763714	4763714	T	C	NDUFA9	intronic	NA	NA	400	89	489	0.182004	73	0	73	0	1	Sub	0.6	3.02			polymorphism	9.016						
OVA_048_M1	METASTASIS	chr7	4798660	4798660	G	A	FOXK1	intronic	NA	NA	16	42	58	0.724138	9	0	9	0	1	Clonal	0.56	3.94			polymorphism	0.004						
OVA_048_M2	METASTASIS	chr7	4798660	4798660	G	A	FOXK1	intronic	NA	NA	39	46	85	0.541176	9	0	9	0	1	Clonal	0.4	3.87			polymorphism	0.004						
OVA_048_M3	METASTASIS	chr7	4798660	4798660	G	A	FOXK1	intronic	NA	NA	23	63	86	0.732558	9	0	9	0	1	Clonal	0.59	3.39			polymorphism	0.004						
OVA_013_M1	METASTASIS	chr17	4836914	4836914	C	T	GP1BA	exonic	ynonymou	000032912	299	140	439	0.318907	56	0	56	0	0.95	Clonal	0.67	3.06			polymorphism	2.629	p.P339S	Passenger	SENGER/OT	benign	Neutral	Damaging
OVA_013_C1	PRIMARY	chr17	4836914	4836914	C	T	GP1BA	exonic	ynonymou	000032912	435	156	591	0.263959	56	0	56	0	1	Sub	0.72	3.1			polymorphism	2.629	p.P339S	Passenger	SENGER/OT	benign	Neutral	Damaging
OVA_013_C2	PRIMARY	chr17	4836914	4836914	C	T	GP1BA	exonic	ynonymou	000032912	307	219	526	0.41635	56	0	56	0	0.96	Sub	0.87	3.05			polymorphism	2.629	p.P339S	Passenger	SENGER/OT	benign	Neutral	Damaging
OVA_013_C3	PRIMARY	chr17	4836914	4836914	C	T	GP1BA	exonic	ynonymou	000032912	344	229	573	0.399651	56	0	56	0	0.87	Sub	0.91	3.01			polymorphism	2.629	p.P339S	Passenger	SENGER/OT	benign	Neutral	Damaging
OVA_378_M1	METASTASIS	chr19	5114013	5114013	C	T	BC032415	intronic	NA	NA	160	124	284	0.43662	81	0	81	0	1	Clonal	0.72	2.57			polymorphism	2.131						
OVA_378_M2	METASTASIS	chr19	5114013	5114013	C	T	BC032415	intronic	NA	NA	120	121	241	0.502075	81	0	81	0	1	Clonal	0.85	2.57			polymorphism	2.131						
OVA_378_M3	METASTASIS	chr19	5114013	5114013	C	T	BC032415	intronic	NA	NA	221	127	348	0.364943	81	0	81	0	1	Clonal	0.67	2.57			polymorphism	2.131						
OVA_378_C1	PRIMARY	chr19	5114013	5114013	C	T	BC032415	intronic	NA	NA	252	120	372	0.322581	81	0	81	0	1	Clonal	0.59	2.6			polymorphism	2.131						
OVA_378_C2	PRIMARY	chr19	5114013	5114013	C	T	BC032415	intronic	NA	NA	165	87	252	0.345238	81	0	81	0	1	Clonal	0.56	2.59			polymorphism	2.131						
OVA_378_C3	PRIMARY	chr19	5114013	5114013	C	T	BC032415	intronic	NA	NA	195	64	259	0.247104	81	0	81	0	0.97	Clonal	0.51	2.6			polymorphism	2.131						
OVA_047_M1	METASTASIS	chr5	5186414	5186414	-	GGGT	ADAMTS16	intronic	NA	NA	129	370	499	0.741483	12	0	12		1	Clonal	0.47	2.01										
OVA_047_M2	METASTASIS	chr5	5186414	5186414	-	GGGT	ADAMTS16	intronic	NA	NA	126	405	531	0.762712	12	0	12		1	Clonal	0.44	2.01										
OVA_047_M3	METASTASIS	chr5	5186414	5186414	-	GGGT	ADAMTS16	intronic	NA	NA	60	240	300	0.8	12	0	12		1	Clonal	0.6	3.02										
OVA_047_C1	PRIMARY	chr5	5186414	5186414	-	GGGT	ADAMTS16	intronic	NA	NA	130	411	541	0.759704	12	0	12		1	Clonal	0.6	2.91										
OVA_047_C2	PRIMARY	chr5	5186414	5186414	-	GGGT	ADAMTS16	intronic	NA	NA	99	378	477	0.792453	12	0	12		1	Clonal	0.64	2.76										
OVA_047_C3	Contralateral ovary	chr5	5186414	5186414	-	GGGT	ADAMTS16	intronic	NA	NA	107	368	475	0.774737	12	0	12		1	Clonal	0.6	2.94										
OVA_047_M1	METASTASIS	chr5	5232442	5232442	G	A	ADAMTS16	intronic	NA	NA	577	114	691	0.164978	62	0	62	0	1	Sub	0.47	2.01			polymorphism	0.489						
OVA_047_M2	METASTASIS	chr5	5232442	5232442	G	A	ADAMTS16	intronic	NA	NA	603	77	680	0.113235	62	0	62	0	0.74	Sub	0.44	2.01			polymorphism	0.489						
OVA_047_M3	METASTASIS	chr5	5232442	5232442	G	A	ADAMTS16	intronic	NA	NA	523	94	617	0.15235	62	0	62	0	0.97	Sub	0.6	3.02			polymorphism	0.489						
OVA_047_C3	Contralateral ovary	chr19	5286014	5286014	G	A	PTPRS	intronic	NA	NA	374	45	419	0.107399	81	0	81	0	0.36	Sub	0.6	2.94			polymorphism	4.867						
OVA_048_C2	PRIMARY	chr9	5787230	5787230	G	C	ERMP1	exonic	ynonymou	877A,ENSG	334	165	499	0.330661	41	0	41	0	0.92	Clonal	0.72	1.85			disease_causing	10.91	p.P877A	Passenger	SENGER/OT	benign	Neutral	Tolerated
OVA_365_M1	METASTASIS	chr20	5924218	5924218	C	T	TRMT6	exonic	ynonymou	M48I,ENSG	359	283	642	0.44081	31	0	31	0	1	Clonal	0.85	1.87			disease_causing	29.6	p.M218I	Passenger	SENGER/OT	damaging	Neutral	Damaging
OVA_365_M2	METASTASIS	chr20	5924218	5924218	C	T	TRMT6	exonic	ynonymou	M48I,ENSG	462	107	569	0.188049	31	0	31	0	0.93	Clonal	0.4	1.81			disease_causing	29.6	p.M218I	Passenger	SENGER/OT	damaging	Neutral	Damaging
OVA_365_M3	METASTASIS	chr20	5924218	5924218	C	T	TRMT6	exonic	ynonymou	M48I,ENSG	371	294	665	0.442105	31	0	31	0	1	Clonal	0.82	1.89			disease_causing	29.6	p.M218I	Passenger	SENGER/OT	damaging	Neutral	Damaging
OVA_365_C1	Contralateral ovary	chr20	5924218	5924218	C	T	TRMT6	exonic	ynonymou	M48I,ENSG	454	115	569	0.202109	31	0	31	0	0.97	Clonal	0.42	1.83			disease_causing	29.6	p.M218I	Passenger	SENGER/OT	damaging	Neutral	Damaging
OVA_365_C2	PRIMARY	chr20	5924218	5924218	C	T	TRMT6	exonic	ynonymou	M48I,ENSG	432	210	642	0.327103	31	0	31	0	1	Clonal	0.62	1.92			disease_causing	29.6	p.M218I	Passenger	SENGER/OT	damaging	Neutral	Damaging
OVA_365_M1	METASTASIS	chr11	5989692	5989692	T	A	OR56A5	cRNA_exon	NA	NA	185	165	350	0.471429	61	0	61	0	1	Clonal	0.85	1.87				3.197		Passenger				
OVA_365_M2	METASTASIS	chr11	5989692	5989692	T	A	OR56A5	cRNA_exon	NA	NA	129	41	170	0.241176	61	0	61	0	1	Clonal	0.4	1.81				3.197		Passenger				
OVA_365_M3	METASTASIS	chr11	5989692	5989692	T	A	OR56A5	cRNA_exon	NA	NA	142	119	261	0.455939	61	0	61	0	1	Clonal	0.82	1.89				3.197		Passenger				
OVA_365_C1	Contralateral ovary	chr11	5989692	5989692	T	A	OR56A5	cRNA_exon	NA	NA	113	31	144	0.215278	61	0	61	0	1	Clonal	0.42	1.83				3.197		Passenger				
OVA_365_C2	PRIMARY	chr11	5989692	5989692	T	A	OR56A5	cRNA_exon	NA	NA	184	87	271	0.321033	61	0	61	0	1	Clonal	0.62	1.92				3.197		Passenger				
OVA_048_M3	METASTASIS	chr6	6320968	6320968	G	C	F13A1	UTR5	NA	NA	140	31	171	0.181287	39	0	39	0	1	Clonal	0.59	3.39			polymorphism	13.21						
OVA_048_C2	PRIMARY	chr11	6432629	6432629	G	T	APBB1	intronic	NA	NA	121	178	299	0.595318	10	0	10	0	1	Clonal	0.72	1.85			polymorphism	10.83						
OVA_047_M1	METASTASIS	chr7	6544447	6544447	G	C	GRID2IP	exonic	ynonymou	000452113	156	149	305	0.488525	80	0	80	0	1	Clonal	0.47	2.01			disease_causing	33	p.S896C	Passenger	SENGER/OT	damaging	Deleterious	Damaging
OVA_047_M2	METASTASIS	chr7	6544447	6544447	G	C	GRID2IP	exonic	ynonymou	000452113	184	144	328	0.439024	80	0	80	0	1	Clonal	0.44	2.01			disease_causing	33	p.S896C	Passenger	SENGER/OT	damaging	Deleterious	Damaging
OVA_047_M3	METASTASIS	chr7	6544447	6544447	G	C	GRID2IP	exonic	ynonymou	000452113	81	57	138	0.413043	80	0	80	0	1	Clonal	0.6	3.02			disease_causing	33	p.S896C	Passenger	SENGER/OT	damaging	Deleterious	Damaging
OVA_013_M1	METASTASIS	chr12	6570032	6570032	G	T	TAPBPL	exonic	ynonymou	000026655	545	62	607	0.102142	75	1	76	0.013158	0.82	Clonal	0.67	3.06			polymorphism	5.216	p.A407S	Passenger	SENGER/OT	benign	Neutral	Tolerated
OVA_013_C1	PRIMARY	chr12	6570032	6570032	G	T	TAPBPL	exonic	ynonymou	000026655	523	73	596	0.122483	75	1	76	0.013158	0.95	Sub	0.72	3.1			polymorphism	5.216	p.A407S	Passenger	SENGER/OT	benign	Neutral	Tolerated
OVA_013_C2	PRIMARY	chr12	6570032	6570032	G	T	TAPBPL	exonic	ynonymou	000026655	536	56	592	0.094595	75	1	76	0.013158	0.6	Sub	0.87	3.05			polymorphism	5.216	p.A407S	Passenger	SENGER/OT	benign	Neutral	Tolerated
OVA_013_C3	PRIMARY	chr12	6570032	6570032	G	T	TAPBPL	exonic	ynonymou	000026655	513	110	623	0.176565	75	1	76	0.013158	1	Sub	0.91	3.01			polymorphism	5.216	p.A407S	Passenger	SENGER/OT	benign	Neutral	Tolerated
OVA_003_M1	METASTASIS	chr12	6574961	6574961	C	T	VAMP1	intronic	NA	NA	487	21	508	0.041339	71	0	71	0	0.15	Sub	0.57	1.84			polymorphism	4.88						
OVA_003_M2	METASTASIS	chr12	6574961	6574961	C	T	VAMP1	intronic	NA	NA	510	42	552	0.076087	71	0	71	0	0.33	Sub	0.46	1.81			polymorphism	4.88						
OVA_003_M3	METASTASIS	chr12	6574961	6574961	C	T	VAMP1	intronic	NA	NA	576	10	586	0.017065	71	0	71	0	0.18	Sub	0.34	2.67			polymorphism	4.88						
OVA_003_C2	PRIMARY	chr12	6574961	6574961	C	T	VAMP1	intronic	NA	NA	1457	31	1489	0.020819	71	0	71	0	0.1	Sub	0.7	3.61			polymorphism	4.88						
OVA_378_M1	METASTASIS	chr1	6646783	6646783	C	T	ZBTB48	exonic	ynonymou	113I,ENSG	503	108	611	0.176759	119	0	119	0	0.84	Clonal	0.72	2.57			disease_causing	19.55	p.T358I	Passenger	SENGER/OT	benign	Neutral	Tolerated
OVA_378_M2	METASTASIS	chr1	6646783	6646783	C	T	ZBTB48	exonic	ynonymou	113I,ENSG	446	128	574	0.222997	119	0	119	0	0.97	Clonal	0.85	2.57			disease_causing	19.55	p.T358I	Passenger	SENGER/OT	benign	Neutral	Tolerated
OVA_378_M3	METASTASIS	chr1	6646783	6646783	C	T	ZBTB48	exonic	ynonymou	113I,ENSG	487	129	616	0.209416	119	0	119	0	1	Clonal	0.67	2.57			disease_causing	19.55	p.T358I	Passenger	SENGER/OT	benign	Neutral	Tolerated
OVA_378_C1	PRIMARY	chr1	6646783	6646783	C	T	ZBTB48	exonic	ynonymou	113I,ENSG	521	108	629	0.171701	119	0	119	0	0.93	Sub	0.59	2.6			disease_causing	19.55	p.T358I	Passenger	SENGER/OT	benign	Neutral	Tolerated
OVA_378_C2	PRIMARY	chr1	6646783	6646783	C	T	ZBTB48	exonic	ynonymou	113I,ENSG	475	94	569	0.165202	119	0	119	0	0.92	Sub	0.56	2.59			disease_causing	19.55	p.T358I	Passenger	SENGER/OT	benign	Neutral	Tolerated
OVA_378_C3	PRIMARY	chr1	6646783	6646783	C	T	ZBTB48	exonic	ynonymou	113I,ENSG	485	57	542	0.105166	119	0	119	0	0.52	Sub	0.51	2.6			disease_causing	19.55	p.T358I	Passenger	SENGER/OT	benign	Neutral	Tolerated
OVA_013_M1	METASTASIS	chr1	6698330	6698330	T	A	DNAJC11	intronic	NA	NA	347	96	443	0.216704	95	1	96	0.010417	1	Clonal	0.67	3.06			polymorphism	0.518						
OVA_013_C1	PRIMARY	chr1	6698330	6698330	T	A	DNAJC11	intronic	NA	NA	443	126	569	0.221441	95	1	96	0.010417	1	Clonal	0.72	3.1			polymorphism	0.518						
OVA_013_C2	PRIMARY	chr1	6698330	6698330	T	A	DNAJC11	intronic	NA	NA	385	153	538	0.284387	95	1	96	0.010417	1	Clonal	0.87	3.05			polymorphism	0.518						
OVA_013_C3	PRIMARY	chr1	6698330	6698330	T	A	DNAJC11	intronic	NA	NA	461	119	580	0.205172	95	1	96	0.010417	1	Clonal	0.91	3.01			polymorphism	0.518						
OVA_047_C2	PRIMARY	chr18	6876218	6876218	T	A	ARHGAP28	intronic	NA	NA	623	24	647	0.037094	64	0	64	0	0.12	Sub	0.64	2.76			polymorphism	6.956						
OVA_013_M1	METASTASIS	chr11	6898603	6898603	G	C	OR10A4	exonic	ynonymou	000037982	278	135	413	0.326877	49	0	49	0	0.98	Clonal	0.67	3.06			disease_causing	25.6	p.C242S	Passenger	SENGER/OT	damaging	Deleterious	Damaging
OVA_013_C1	PRIMARY	chr11	6898603	6898603	G	C	OR10A4	exonic	ynonymou	000037982	344	224	568	0.394366	49	0	49	0	1	Clonal	0.72	3.1			disease_causing	25.6	p.C242S	Passenger	SENGER/OT	damaging	Deleterious	Damaging
OVA_013_C2	PRIMARY	chr11	6898603	6898603	G	C	OR10A4	exonic	ynonymou	000037982	334	229	563	0.40675	49	0	49	0	0.94	Clonal	0.87	3.05			disease_causing	25.6	p.C242S	Passenger	SENGER/OT	damaging	Deleterious	Damaging
OVA_013_C3	PRIMARY	chr11	6898603	6898603	G	C	OR10A4	exonic	ynonymou	000037982	319	271	590	0.459322	49	0	49	0	0.91	Clonal	0.91	3.01			disease_causing	25.6	p.C242S	Passenger	SENGER/OT	damaging	Deleterious	Damaging
OVA_047_M1	METASTASIS	chr19	6908817	6908817	T	A	EMR1	intronic	NA	NA	384	191	575	0.332174	18	0	18	0	1	Clonal	0.47	2.01			polymorphism	4.114						
OVA_047_M2	METASTASIS	chr19	6908817	6908817	T	A	EMR1	intronic	NA	NA	426	166	592	0.280405	18	0	18	0	0.98	Clonal	0.44	2.01			polymorphism	4.114						
OVA_047_M3	METASTASIS	chr19	6908817	6908817	T	A	EMR1	intronic	NA	NA	185	94	279	0.336918	18	0	18	0	1	Clonal	0.6	3.02			polymorphism	4.114						
OVA_048_C1	PRIMARY	chr19	6921777	6921777	G	T	EMR1	exonic	ynonymou	000381404	112	28	140	0.2	37	0	37	0	0.4	Sub	0.67	1.82			polymorphism	8.841	p.K558N	Passenger	SENGER/OT	benign	Neutral	Tolerated
OVA_003_M1	METASTASIS	chr12	6933825	6933825	G	A	GPR162	exonic	ynonymou	000031126	200	66	266	0.24812	147	0	147	0	0.88	Sub	0.57	1.84			disease_causing	28.7	p.R254Q	Passenger	SENGER/OT	damaging	Neutral	Damaging
OVA_003_M2	METASTASIS	chr12	6933825	6933825	G	A	GPR162	exonic	ynonymou	000031126	275	62	337	0.183976	147	0	147	0	0.8	Sub	0.46	1.81			disease_causing	28.7	p.R254Q	Passenger	SENGER/OT	damaging	Neutral	Damaging
OVA_003_M3	METASTASIS	chr12	6933825	6933825	G	A	GPR162	exonic	ynonymou	000031126	346	21	367	0.057221	147	0	147	0	0.6	Sub	0.34	2.67			disease_causing	28.7	p.R254Q	Passenger	SENGER/OT	damaging	Neutral	Damaging
OVA_003_C1	PRIMARY	chr12	6933825	6933825	G	A	GPR162	exonic	ynonymou	000031126	200	132	332	0.39759	147	0	147	0	0.92	Clonal	0.86	1.81			disease_causing	28.7	p.R254Q	Passenger	SENGER/OT	damaging	Neutral	Damaging
OVA_003_C2	PRIMARY	chr12	6933825	6933825	G	A	GPR162	exonic	ynonymou	000031126	262	155	417	0.371703	147	0	147	0	1	Clonal	0.7	3.61			disease_causing	28.7	p.R254Q	Passenger	SENGER/OT	damaging	Neutral	Damaging
OVA_003_C3	PRIMARY	chr12	6933825	6933825	G	A	GPR162	exonic	ynonymou	000031126	249	160	409	0.391198	147	0	147	0	0.95	Clonal	0.82	1.81			disease_causing	28.7	p.R254Q	Passenger	SENGER/OT	damaging	Neutral	Damaging
OVA_047_M2	METASTASIS	chr18	7012173	7012173	A	T	LAMA1	intronic	NA	NA	500	85	585	0.145299	65	0	65	0	0.66	Sub	0.44	2.01			polymorphism	0.431						
OVA_047_M1	METASTASIS	chr4	7016217	7016217	A	C	TBC1D14	exonic	ynonymou	00410031:e	515	179	694	0.257925	41	0	41	0	1	Sub	0.47	2.01			disease_causing	26.6	p.N564H	Passenger	SENGER/OT	benign	Deleterious	Tolerated
OVA_047_M2	METASTASIS	chr4	7016217	7016217	A	C	TBC1D14	exonic	ynonymou	00410031:e	520	160	680	0.235294	41	0	41	0	1	Sub	0.44	2.01			disease_causing	26.6	p.N564H	Passenger	SENGER/OT	benign	Deleterious	Tolerated
OVA_047_M3	METASTASIS	chr4	7016217	7016217	A	C	TBC1D14	exonic	ynonymou	00410031:e	430	66	496	0.133065	41	0	41	0	0.58	Sub	0.6	3.02			disease_causing	26.6	p.N564H	Passenger	SENGER/OT	benign	Deleterious	Tolerated
OVA_048_C2	PRIMARY	chr4	7032128	7032128	C	T	TBC1D14	UTR3	NA	NA	356	19	375	0.050667	42	1	43	0.023256	0.14	Sub	0.72	1.85			polymorphism	4.581						
OVA_365_M1	METASTASIS	chr4	7044639	7044639	C	G	CCDC96	exonic	ynonymou	T00000310	27	37	64	0.578125	75	0	75	0	1	Clonal	0.85	1.87			polymorphism	10.66	p.K9N	Passenger	SENGER/OT	benign	Neutral	Tolerated
OVA_365_M2	METASTASIS	chr4	7044639	7044639	C	G	CCDC96	exonic	ynonymou	T00000310	27	5	32	0.15625	75	0	75	0	0.78	Clonal	0.4	1.81			polymorphism	10.66	p.K9N	Passenger	SENGER/OT	benign	Neutral	Tolerated
OVA_365_M3	METASTASIS	chr4	7044639	7044639	C	G	CCDC96	exonic	ynonymou	T00000310	29	16	45	0.355556	75	0	75	0	0.87	Clonal	0.82	1.89			polymorphism	10.66	p.K9N	Passenger	SENGER/OT	benign	Neutral	Tolerated
OVA_365_C2	PRIMARY	chr4	7044639	7044639	C	G	CCDC96	exonic	ynonymou	T00000310	50	19	69	0.275362	75	0	75	0	0.89	Clonal	0.62	1.92			polymorphism	10.66	p.K9N	Passenger	SENGER/OT	benign	Neutral	Tolerated
OVA_013_M1	METASTASIS	chr17	7094711	7094711	G	T	DLG4	intronic	NA	NA	129	53	182	0.291209	44	1	45	0.022222	0.87	Clonal	0.67	3.06			polymorphism	1.71						
OVA_013_C1	PRIMARY	chr17	7094711	7094711	G	T	DLG4	intronic	NA	NA	151	117	268	0.436567	44	1	45	0.022222	1	Sub	0.72	3.1			polymorphism	1.71						
OVA_013_C2	PRIMARY	chr17	7094711	7094711	G	T	DLG4	intronic	NA	NA	151	89	240	0.370833	44	1	45	0.022222	0.85	Sub	0.87	3.05			polymorphism	1.71						
OVA_013_C3	PRIMARY	chr17	7094711	7094711	G	T	DLG4	intronic	NA	NA	159	117	276	0.423913	44	1	45	0.022222	0.93	Sub	0.91	3.01			polymorphism	1.71						
OVA_378_M1	METASTASIS	chr12	7187941	7187941	C	T	C1R	exonic	nonymous S	000542285	223	94	317	0.29653	78	0	78	0	1	Clonal	0.72	2.57			disease_causing	6.898		Passenger			Neutral	Tolerated
OVA_378_M2	METASTASIS	chr12	7187941	7187941	C	T	C1R	exonic	nonymous S	000542285	234	98	332	0.295181	78	0	78	0	0.99	Clonal	0.85	2.57			disease_causing	6.898		Passenger			Neutral	Tolerated
OVA_378_M3	METASTASIS	chr12	7187941	7187941	C	T	C1R	exonic	nonymous S	000542285	297	94	391	0.240409	78	0	78	0	0.96	Clonal	0.67	2.57			disease_causing	6.898		Passenger			Neutral	Tolerated
OVA_378_C1	PRIMARY	chr12	7187941	7187941	C	T	C1R	exonic	nonymous S	000542285	314	113	427	0.264637	78	0	78	0	1	Clonal	0.59	2.6			disease_causing	6.898		Passenger			Neutral	Tolerated
OVA_378_C2	PRIMARY	chr12	7187941	7187941	C	T	C1R	exonic	nonymous S	000542285	299	72	371	0.19407	78	0	78	0	0.88	Clonal	0.56	2.59			disease_causing	6.898		Passenger			Neutral	Tolerated

Sample	Type	Chr	Start	End	Ref	Alt	Gene	unc.ensGen	icFunc.ens	hange.ens	t_ref	t_alt	t_total	t_VAF	n_ref	n_alt	n_total	normal_VA	ncer_cell_f	on-based C	purity	ploidy	Cancer Gene	Driver Gene	Mutation Taster	ADD(PHRE	otein_chan	CHASM	ATHMM_Pr	OLYPHEN-	REDICTION	ICTION (cutoff=0.05)
OVA_378_C3	PRIMARY	chr12	7187941	7187941	C	T	C1R	exonic	nonymous S	000054228	264	82	346	0.236994	78	0	78	0	1	Clonal	0.51	2.6			disease_causing	6.898		Passenger			Neutral	Tolerated
OVA_048_M1	METASTASIS	chr17	7330259	7330259	G	C	C17orf74	exonic	ynonymou	000033387	27	108	135	0.8	61	0	61	0	1	Clonal	0.56	3.94			polymorphism	11.29	p.G317R	Passenger	SENGER/OT	benign	Deleterious	Damaging
OVA_048_M2	METASTASIS	chr17	7330259	7330259	G	C	C17orf74	exonic	ynonymou	000033387	68	78	146	0.534247	61	0	61	0	1	Clonal	0.4	3.87			polymorphism	11.29	p.G317R	Passenger	SENGER/OT	benign	Deleterious	Damaging
OVA_048_M3	METASTASIS	chr17	7330259	7330259	G	C	C17orf74	exonic	ynonymou	000033387	31	45	76	0.592105	61	0	61	0	1	Clonal	0.59	3.39			polymorphism	11.29	p.G317R	Passenger	SENGER/OT	benign	Deleterious	Damaging
OVA_365_M1	METASTASIS	chr17	7348638	7348638	T	A	CHRNB1	exonic	nonymous S	.S36S,ENSG	10	51	61	0.836066	129	0	129	0	1	Clonal	0.85	1.87			polymorphism	15.96		Passenger			Neutral	Tolerated
OVA_365_M2	METASTASIS	chr17	7348638	7348638	T	A	CHRNB1	exonic	nonymous S	.S36S,ENSG	28	21	49	0.428571	129	0	129	0	1	Clonal	0.4	1.81			polymorphism	15.96		Passenger			Neutral	Tolerated
OVA_365_M3	METASTASIS	chr17	7348638	7348638	T	A	CHRNB1	exonic	nonymous S	.S36S,ENSG	11	39	50	0.78	129	0	129	0	1	Clonal	0.82	1.89			polymorphism	15.96		Passenger			Neutral	Tolerated
OVA_365_C1	Contralateral ovary	chr17	7348638	7348638	T	A	CHRNB1	exonic	nonymous S	.S36S,ENSG	23	19	42	0.452381	129	0	129	0	1	Clonal	0.42	1.83			polymorphism	15.96		Passenger			Neutral	Tolerated
OVA_365_C2	PRIMARY	chr17	7348638	7348638	T	A	CHRNB1	exonic	nonymous S	.S36S,ENSG	33	40	73	0.547945	129	0	129	0	1	Clonal	0.62	1.92			polymorphism	15.96		Passenger			Neutral	Tolerated
OVA_048_M1	METASTASIS	chr17	7495631	7495631	G	A	FXR2	exonic	ynonymou	000025011	81	378	459	0.823529	54	1	55	0.018182	1	Clonal	0.56	3.94			disease_causing	29.8	p.R623C	Passenger	SENGER/OT	damaging	Neutral	Damaging
OVA_048_M2	METASTASIS	chr17	7495631	7495631	G	A	FXR2	exonic	ynonymou	000025011	152	253	405	0.624691	54	1	55	0.018182	1	Clonal	0.4	3.87			disease_causing	29.8	p.R623C	Passenger	SENGER/OT	damaging	Neutral	Damaging
OVA_048_M3	METASTASIS	chr17	7495631	7495631	G	A	FXR2	exonic	ynonymou	000025011	101	229	330	0.693939	54	1	55	0.018182	1	Clonal	0.59	3.39			disease_causing	29.8	p.R623C	Passenger	SENGER/OT	damaging	Neutral	Damaging
OVA_048_C1	PRIMARY	chr17	7495631	7495631	G	A	FXR2	exonic	ynonymou	000025011	69	89	158	0.563291	54	1	55	0.018182	1	Clonal	0.67	1.82			disease_causing	29.8	p.R623C	Passenger	SENGER/OT	damaging	Neutral	Damaging
OVA_048_C2	PRIMARY	chr17	7495631	7495631	G	A	FXR2	exonic	ynonymou	000025011	101	203	304	0.667763	54	1	55	0.018182	1	Clonal	0.72	1.85			disease_causing	29.8	p.R623C	Passenger	SENGER/OT	damaging	Neutral	Damaging
OVA_048_C3	PRIMARY	chr17	7495631	7495631	G	A	FXR2	exonic	ynonymou	000025011	64	92	156	0.589744	54	1	55	0.018182	1	Clonal	0.74	1.8			disease_causing	29.8	p.R623C	Passenger	SENGER/OT	damaging	Neutral	Damaging
OVA_047_M1	METASTASIS	chr17	7574012	7574012	C	A	TP53	exonic	stopgain	339X,ENSG	190	449	639	0.70266	40	0	40	0	1	Clonal	0.47	2.01	Yes	Yes	isease_causing_automat	37	p.E339X	Passenger			NA	NA
OVA_047_M2	METASTASIS	chr17	7574012	7574012	C	A	TP53	exonic	stopgain	339X,ENSG	311	354	665	0.532331	40	0	40	0	1	Clonal	0.44	2.01	Yes	Yes	isease_causing_automat	37	p.E339X	Passenger			NA	NA
OVA_047_M3	METASTASIS	chr17	7574012	7574012	C	A	TP53	exonic	stopgain	339X,ENSG	156	261	417	0.625899	40	0	40	0	1	Clonal	0.6	3.02	Yes	Yes	isease_causing_automat	37	p.E339X	Passenger			NA	NA
OVA_047_C1	PRIMARY	chr17	7574012	7574012	C	A	TP53	exonic	stopgain	339X,ENSG	186	499	685	0.728467	40	0	40	0	1	Clonal	0.6	2.91	Yes	Yes	isease_causing_automat	37	p.E339X	Passenger			NA	NA
OVA_047_C2	PRIMARY	chr17	7574012	7574012	C	A	TP53	exonic	stopgain	339X,ENSG	197	493	690	0.714493	40	0	40	0	1	Clonal	0.64	2.76	Yes	Yes	isease_causing_automat	37	p.E339X	Passenger			NA	NA
OVA_047_C3	Contralateral ovary	chr17	7574012	7574012	C	A	TP53	exonic	stopgain	339X,ENSG	192	498	690	0.721739	40	0	40	0	1	Clonal	0.6	2.94	Yes	Yes	isease_causing_automat	37	p.E339X	Passenger			NA	NA
OVA_378_M1	METASTASIS	chr17	7574030	7574030	G	-	TP53	exonic	neshift dele	333fs,ENSG	214	702	916	0.766376	45	0	45		1	Clonal	0.72	2.57	Yes	Yes			p.R333Vfs	Passenger			NA	NA
OVA_378_M2	METASTASIS	chr17	7574030	7574030	G	-	TP53	exonic	neshift dele	333fs,ENSG	115	732	847	0.864227	45	0	45		1	Clonal	0.85	2.57	Yes	Yes			p.R333Vfs	Passenger			NA	NA
OVA_378_M3	METASTASIS	chr17	7574030	7574030	G	-	TP53	exonic	neshift dele	333fs,ENSG	241	635	876	0.724886	45	0	45		1	Clonal	0.67	2.57	Yes	Yes			p.R333Vfs	Passenger			NA	NA
OVA_378_C1	PRIMARY	chr17	7574030	7574030	G	-	TP53	exonic	neshift dele	333fs,ENSG	401	673	1074	0.626629	45	0	45		1	Clonal	0.59	2.6	Yes	Yes			p.R333Vfs	Passenger			NA	NA
OVA_378_C2	PRIMARY	chr17	7574030	7574030	G	-	TP53	exonic	neshift dele	333fs,ENSG	418	461	879	0.52446	45	0	45		1	Clonal	0.56	2.59	Yes	Yes			p.R333Vfs	Passenger			NA	NA
OVA_378_C3	PRIMARY	chr17	7574030	7574030	G	-	TP53	exonic	neshift dele	333fs,ENSG	455	440	895	0.49162	45	0	45		1	Clonal	0.51	2.6	Yes	Yes			p.R333Vfs	Passenger			NA	NA
OVA_003_M1	METASTASIS	chr17	7578234	7578234	A	C	TP53	exonic	stopgain	112X,ENSG	279	228	507	0.449704	67	0	67	0	1	Sub	0.57	1.84	Yes	Yes	isease_causing_automat	35	p.Y205X	Passenger			NA	NA
OVA_003_M2	METASTASIS	chr17	7578234	7578234	A	C	TP53	exonic	stopgain	112X,ENSG	388	148	536	0.276119	67	0	67	0	0.93	Sub	0.46	1.81	Yes	Yes	isease_causing_automat	35	p.Y205X	Passenger			NA	NA
OVA_003_M3	METASTASIS	chr17	7578234	7578234	A	C	TP53	exonic	stopgain	112X,ENSG	520	55	575	0.095652	67	0	67	0	0.72	Sub	0.34	2.67	Yes	Yes	isease_causing_automat	35	p.Y205X	Passenger			NA	NA
OVA_003_C1	PRIMARY	chr17	7578234	7578234	A	C	TP53	exonic	stopgain	112X,ENSG	115	439	554	0.792419	67	0	67	0	1	Clonal	0.86	1.81	Yes	Yes	isease_causing_automat	35	p.Y205X	Passenger			NA	NA
OVA_003_C2	PRIMARY	chr17	7578234	7578234	A	C	TP53	exonic	stopgain	112X,ENSG	134	409	543	0.753223	67	0	67	0	1	Clonal	0.7	3.61	Yes	Yes	isease_causing_automat	35	p.Y205X	Passenger			NA	NA
OVA_003_C3	PRIMARY	chr17	7578234	7578234	A	C	TP53	exonic	stopgain	112X,ENSG	148	413	561	0.736185	67	0	67	0	1	Clonal	0.82	1.81	Yes	Yes	isease_causing_automat	35	p.Y205X	Passenger			NA	NA
OVA_365_M1	METASTASIS	chr17	7578266	7578271	TAAGAT	-	TP53	exonic	ameshift de	2del,ENSG0	91	251	342	0.733918	79	0	79		1	Sub	0.85	1.87	Yes		disease causing		193_I195de	Passenger			Deleterious	NA
OVA_365_M2	METASTASIS	chr17	7578266	7578271	TAAGAT	-	TP53	exonic	ameshift de	2del,ENSG0	227	87	314	0.27707	79	0	79		1	Sub	0.4	1.81	Yes		disease causing		193_I195de	Passenger			Deleterious	NA
OVA_365_M3	METASTASIS	chr17	7578266	7578271	TAAGAT	-	TP53	exonic	ameshift de	2del,ENSG0	374	130	504	0.257937					0.37	Sub	0.82	1.89	Yes		disease causing		193_I195de	Passenger			Deleterious	NA
OVA_365_C1	Contralateral ovary	chr17	7578266	7578271	TAAGAT	-	TP53	exonic	ameshift de	2del,ENSG0	274	99	373	0.265416	79	0	79		1	Clonal	0.42	1.83	Yes		disease causing		193_I195de	Passenger			Deleterious	NA
OVA_365_C2	PRIMARY	chr17	7578266	7578271	TAAGAT	-	TP53	exonic	ameshift de	2del,ENSG0	202	133	335	0.397015	79	0	79		0.89	Clonal	0.62	1.92	Yes		disease causing		193_I195de	Passenger			Deleterious	NA
OVA_048_M1	METASTASIS	chr17	7578538	7578538	T	A	TP53	exonic	ynonymou	I131I,ENSG	128	454	582	0.780069	43	1	44	0.022727	1	Clonal	0.56	3.94	Yes	Yes	disease_causing	29.3	p.N131I	Driver		damaging	Deleterious	Damaging
OVA_048_M2	METASTASIS	chr17	7578538	7578538	T	A	TP53	exonic	ynonymou	I131I,ENSG	246	337	583	0.578045	43	1	44	0.022727	1	Clonal	0.4	3.87	Yes	Yes	disease_causing	29.3	p.N131I	Driver		damaging	Deleterious	Damaging
OVA_048_M3	METASTASIS	chr17	7578538	7578538	T	A	TP53	exonic	ynonymou	I131I,ENSG	185	357	542	0.658672	43	1	44	0.022727	1	Clonal	0.59	3.39	Yes	Yes	disease_causing	29.3	p.N131I	Driver		damaging	Deleterious	Damaging
OVA_048_C1	PRIMARY	chr17	7578538	7578538	T	A	TP53	exonic	ynonymou	I131I,ENSG	105	110	215	0.511628	43	1	44	0.022727	1	Clonal	0.67	1.82	Yes	Yes	disease_causing	29.3	p.N131I	Driver		damaging	Deleterious	Damaging
OVA_048_C2	PRIMARY	chr17	7578538	7578538	T	A	TP53	exonic	ynonymou	I131I,ENSG	203	280	483	0.57971	43	1	44	0.022727	1	Clonal	0.72	1.85	Yes	Yes	disease_causing	29.3	p.N131I	Driver		damaging	Deleterious	Damaging
OVA_048_C3	PRIMARY	chr17	7578538	7578538	T	A	TP53	exonic	ynonymou	I131I,ENSG	134	187	321	0.582555	43	1	44	0.022727	0.99	Clonal	0.74	1.8	Yes	Yes	disease_causing	29.3	p.N131I	Driver		damaging	Deleterious	Damaging
OVA_048_M1	METASTASIS	chr17	7754469	7754469	T	G	KDM6B	exonic	ynonymou	268W,ENSG	154	425	579	0.734024	135	1	136	0.007353	1	Clonal	0.56	3.94			disease_causing	20.7	p.C1268W	Passenger	CANCER	damaging	Deleterious	Damaging
OVA_048_M2	METASTASIS	chr17	7754469	7754469	T	G	KDM6B	exonic	ynonymou	268W,ENSG	273	288	561	0.513369	135	1	136	0.007353	1	Clonal	0.4	3.87			disease_causing	20.7	p.C1268W	Passenger	CANCER	damaging	Deleterious	Damaging
OVA_048_M3	METASTASIS	chr17	7754469	7754469	T	G	KDM6B	exonic	ynonymou	268W,ENSG	210	336	546	0.615385	135	1	136	0.007353	1	Clonal	0.59	3.39			disease_causing	20.7	p.C1268W	Passenger	CANCER	damaging	Deleterious	Damaging
OVA_048_C1	PRIMARY	chr17	7754469	7754469	T	G	KDM6B	exonic	ynonymou	268W,ENSG	181	173	354	0.488701	135	1	136	0.007353	0.97	Sub	0.67	1.82			disease_causing	20.7	p.C1268W	Passenger	CANCER	damaging	Deleterious	Damaging
OVA_048_C2	PRIMARY	chr17	7754469	7754469	T	G	KDM6B	exonic	ynonymou	268W,ENSG	234	267	501	0.532934	135	1	136	0.007353	0.95	Sub	0.72	1.85			disease_causing	20.7	p.C1268W	Passenger	CANCER	damaging	Deleterious	Damaging
OVA_048_C3	PRIMARY	chr17	7754469	7754469	T	G	KDM6B	exonic	ynonymou	268W,ENSG	141	131	272	0.481618	135	1	136	0.007353	0.82	Sub	0.74	1.8			disease_causing	20.7	p.C1268W	Passenger	CANCER	damaging	Deleterious	Damaging
OVA_047_M1	METASTASIS	chr17	7758556	7758556	T	C	TMEM88	exonic	ynonymou	.L55P,ENSG	173	54	227	0.237885	87	0	87	0	0.76	Sub	0.47	2.01			disease_causing	28.2	p.L55P	Passenger	SENGER/OT	damaging	Deleterious	Damaging
OVA_003_M1	METASTASIS	chr19	7762048	7762048	G	T	FCER2	intronic	NA	NA	93	36	129	0.27907	14	0	14	0	0.99	Sub	0.57	1.84			polymorphism	0.425						
OVA_003_M2	METASTASIS	chr19	7762048	7762048	G	T	FCER2	intronic	NA	NA	166	47	213	0.220657	14	0	14	0	0.96	Sub	0.46	1.81			polymorphism	0.425						
OVA_003_M3	METASTASIS	chr19	7762048	7762048	G	T	FCER2	intronic	NA	NA	293	24	317	0.07571	14	0	14	0	0.72	Sub	0.34	2.67			polymorphism	0.425						
OVA_003_C1	PRIMARY	chr19	7762048	7762048	G	T	FCER2	intronic	NA	NA	163	136	299	0.454849	14	0	14	0	1	Clonal	0.86	1.81			polymorphism	0.425						
OVA_003_C2	PRIMARY	chr19	7762048	7762048	G	T	FCER2	intronic	NA	NA	193	188	381	0.493438	14	0	14	0	1	Clonal	0.7	3.61			polymorphism	0.425						
OVA_003_C3	PRIMARY	chr19	7762048	7762048	G	T	FCER2	intronic	NA	NA	196	176	372	0.473118	14	0	14	0	1	Clonal	0.82	1.81			polymorphism	0.425						
OVA_048_M1	METASTASIS	chr19	7937071	7937071	C	T	FLJ22184	exonic	nonymous S	000053942	17	43	60	0.716667	40	0	40	0	1	Clonal	0.56	3.94			polymorphism	4.603		Passenger			Neutral	Tolerated
OVA_048_M2	METASTASIS	chr19	7937071	7937071	C	T	FLJ22184	exonic	nonymous S	000053942	35	26	61	0.42623	40	0	40	0	1	Clonal	0.4	3.87			polymorphism	4.603		Passenger			Neutral	Tolerated
OVA_048_M3	METASTASIS	chr19	7937071	7937071	C	T	FLJ22184	exonic	nonymous S	000053942	20	53	73	0.726027	40	0	40	0	1	Clonal	0.59	3.39			polymorphism	4.603		Passenger			Neutral	Tolerated
OVA_048_M1	METASTASIS	chr17	8383800	8383800	C	T	MYH10	exonic	ynonymou	0379980:ex	93	9	102	0.088235	33	0	33	0	0.49	Sub	0.56	3.94			disease_causing	24.7	p.A1770T	Passenger	SENGER/OT	benign	Neutral	Tolerated
OVA_047_C1	PRIMARY	chr17	8790015	8790015	C	A	PIK3R5	intronic	NA	NA	270	66	336	0.196429	21	0	21	0	0.85	Sub	0.6	2.91			polymorphism	2.81						
OVA_047_C2	PRIMARY	chr17	8790015	8790015	C	A	PIK3R5	intronic	NA	NA	273	69	342	0.201754	21	0	21	0	0.83	Sub	0.64	2.76			polymorphism	2.81						
OVA_047_C3	Contralateral ovary	chr17	8790015	8790015	C	A	PIK3R5	intronic	NA	NA	269	70	339	0.20649	21	0	21	0	0.9	Clonal	0.6	2.94			polymorphism	2.81						
OVA_003_M1	METASTASIS	chr12	8802115	8802115	C	G	MFAP5	exonic	ynonymou	109Q,ENSG	433	194	627	0.30941	31	0	31	0	1	Sub	0.57	1.84			disease_causing	25.4	p.E131Q	Passenger	no weights	damaging	Neutral	Damaging
OVA_003_M2	METASTASIS	chr12	8802115	8802115	C	G	MFAP5	exonic	ynonymou	109Q,ENSG	496	142	638	0.222571	31	0	31	0	0.97	Sub	0.46	1.81			disease_causing	25.4	p.E131Q	Passenger	no weights	damaging	Neutral	Damaging
OVA_003_M3	METASTASIS	chr12	8802115	8802115	C	G	MFAP5	exonic	ynonymou	109Q,ENSG	634	45	679	0.066274	31	0	31	0	0.7	Sub	0.34	2.67			disease_causing	25.4	p.E131Q	Passenger	no weights	damaging	Neutral	Damaging
OVA_003_C1	PRIMARY	chr12	8802115	8802115	C	G	MFAP5	exonic	ynonymou	109Q,ENSG	354	301	655	0.459542	31	0	31	0	1	Clonal	0.86	1.81			disease_causing	25.4	p.E131Q	Passenger	no weights	damaging	Neutral	Damaging
OVA_003_C2	PRIMARY	chr12	8802115	8802115	C	G	MFAP5	exonic	ynonymou	109Q,ENSG	350	280	630	0.444444	31	0	31	0	1	Clonal	0.7	3.61			disease_causing	25.4	p.E131Q	Passenger	no weights	damaging	Neutral	Damaging
OVA_003_C3	PRIMARY	chr12	8802115	8802115	C	G	MFAP5	exonic	ynonymou	109Q,ENSG	393	275	668	0.411677	31	0	31	0	1	Clonal	0.82	1.81			disease_causing	25.4	p.E131Q	Passenger	no weights	damaging	Neutral	Damaging
OVA_013_M1	METASTASIS	chr19	9049804	9049804	A	T	MUC16	exonic	nonymous S	000397910:	447	154	601	0.25624	23	0	23	0	1	Clonal	0.67	3.06	Yes		polymorphism	0.004		Passenger			Neutral	Tolerated
OVA_013_C1	PRIMARY	chr19	9049804	9049804	A	T	MUC16	exonic	nonymous S	000397910:	421	158	579	0.272884	23	0	23	0	1	Clonal	0.72	3.1	Yes		polymorphism	0.004		Passenger			Neutral	Tolerated
OVA_013_C2	PRIMARY	chr19	9049804	9049804	A	T	MUC16	exonic	nonymous S	000397910:	409	177	586	0.302048	23	0	23	0	1	Clonal	0.87	3.05	Yes		polymorphism	0.004		Passenger			Neutral	Tolerated
OVA_013_C3	PRIMARY	chr19	9049804	9049804	A	T	MUC16	exonic	nonymous S	000397910:	416	208	624	0.333333	23	0	23	0	1	Clonal	0.91	3.01	Yes		polymorphism	0.004		Passenger			Neutral	Tolerated
OVA_047_M2	METASTASIS	chr19	9056398	9056398	C	G	MUC16	exonic	ynonymou	00397910:	576	121	697	0.173601	92	0	92	0	0.61	Sub	0.44	2.01	Yes	No	polymorphism	7.946	p.E10350Q	Passenger	SENGER/OT	damaging	Neutral	Tolerated
OVA_048_C3	PRIMARY	chr19	9059055	9059055	G	A	MUC16	exonic	ynonymou	000397910	145	225	370	0.608108	67	0	67	0	1	Clonal	0.74	1.8	Yes	No	polymorphism	2.301	p.T9464I	Passenger	SENGER/OT	benign	Deleterious	Tolerated
OVA_047_C1	PRIMARY	chr19	9077103	9077103	T	G	MUC16	exonic	ynonymou	000397910	448	222	670	0.331343	40	0	40	0	1	Clonal	0.6	2.91	Yes	No	polymorphism	8.678	p.N3448T	Passenger	SENGER/OT	benign	Neutral	Damaging
OVA_047_C2	PRIMARY	chr19	9077103	9077103	T	G	MUC16	exonic	ynonymou	000397910	472	201	673	0.298663	40	0	40	0	0.93	Clonal	0.64	2.76	Yes	No	polymorphism	8.678	p.N3448T	Passenger	SENGER/OT	benign	Neutral	Damaging
OVA_047_C3	Contralateral ovary	chr19	9077103	9077103	T	G	MUC16	exonic	ynonymou	000397910	461	230	691	0.332851	40	0	40	0	1	Clonal	0.6	2.94	Yes	No	polymorphism	8.678	p.N3448T	Passenger	SENGER/OT	benign	Neutral	Damaging
OVA_047_C3	Contralateral ovary	chr11	9087511	9087511	G	C	SCUBE2	exonic	nonymous S	259V,ENSG	414	185	599	0.308848	59	0	59	0	1	Clonal	0.6	2.94			polymorphism	8.143		Passenger			Neutral	Tolerated
OVA_048_M1	METASTASIS	chr19	9251072	9251072	C	T	ZNF317	UTR5	NA	NA	406	74	480	0.154167	56	0	56	0	1	Clonal	0.56	3.94			disease_causing	17.01						
OVA_048_M2	METASTASIS	chr19	9251072	9251072	C	T	ZNF317	UTR5	NA	NA	352	39	391	0.099744	56	0	56	0	1	Clonal	0.4	3.87			disease_causing	17.01						
OVA_048_C3	PRIMARY	chr18	9258096	9258112	AGTCTTCCA	-	ANKRD12	exonic	neshift dele	0400020:ex	440	129	569	0.226714	47	0	47		0.61	Sub	0.74	1.8					p.Y1611Pfs	Passenger			NA	NA
OVA_365_M1	METASTASIS	chr20	9353867	9353867	G	T	PLCB4	intronic	NA	NA	328	238	566	0.420495	22	0	22	0	0.99	Clonal	0.85	1.87			disease_causing	19.18						
OVA_365_M2	METASTASIS	chr20	9353867	9353867	G	T	PLCB4	intronic	NA	NA	405	95	500	0.19	22	0	22	0	0.94	Clonal	0.4	1.81			disease_causing	19.18						
OVA_365_M3	METASTASIS	chr20	9353867	9353867	G	T	PLCB4	intronic	NA	NA	339	234	573	0.408377	22	0	22	0	1	Clonal	0.82	1.89			disease_causing	19.18						
OVA_365_C1	Contralateral ovary	chr20	9353867	9353867	G	T	PLCB4	intronic	NA	NA	439	74	513	0.14425	22	0	22	0	0.69	Sub	0.42	1.83			disease_causing	19.18						
OVA_365_C2	PRIMARY	chr20	9353867	9353867	G	T	PLCB4	intronic	NA	NA	398	163	561	0.290553	22	0	22	0	0.94	Sub	0.62	1.92			disease_causing	19.18						
OVA_047_C1	PRIMARY	chr19	9362754	9362754	T	C	OR7E24	UTR3	NA	NA	377	168	545	0.308257	23	0	23	0	1	Clonal	0.6	2.91			polymorphism	3.546						
OVA_047_C2	PRIMARY	chr19	9362754	9362754	T	C	OR7E24	UTR3	NA	NA	433	175	608	0.287829	23	0	23	0	0.9	Clonal	0.64	2.76			polymorphism	3.546						
OVA_047_C3	Contralateral ovary	chr19	9362754	9362754	T	C	OR7E24	UTR3	NA	NA	395	190	585	0.324786	23	0	23	0	1	Clonal	0.6	2.94			polymorphism	3.546						
OVA_048_C2	PRIMARY	chr3	9547843	9547843	C	T	LHFPL4	exonic	ynonymou	000028758	295	199	494	0.402834	40	0	40	0	1	Clonal	0.72	1.85			disease_causing	24	p.D151N	Passenger	SENGER/OT	benign	Neutral	Tolerated
OVA_365_M3	METASTASIS	chr8	9562252	9562252	A	T	TNKS	exonic	ynonymou	000005182	516	40	556	0.071942	45	0	45	0	0.18	Sub	0.82	1.89			disease_causing	24.7	p.H396L	Passenger	CANCER	damaging	Deleterious	Damaging
OVA_047_C2	PRIMARY	chr1	9780692	9780692	C	G	PIK3CD	exonic	ynonymou	22R,ENSG0	109	47	156	0.301282	16	0	16	0	0.94	Clonal	0.64	2.76			polymorphism	0.17	p.S522R	Passenger	CANCER	benign	Neutral	Tolerated
OVA_048_C3	PRIMARY	chr3	9876540	9876540	C	G	TTLL3	exonic	nonymous S	597L,ENSG	175	82	257	0.319066	72	0	72	0	0.86	Clonal	0.74	1.8			disease_causing	17.12		Passenger			Neutral	Tolerated
OVA_047_C3	Contralateral ovary	chr3	9952894	9952894	G	A	IL17RE	intronic	NA	NA	619	44	663	0.066365	67	0	67	0	0.36	Sub	0.6	2.94			polymorphism	6.349						
OVA_003_C2	PRIMARY	chrX	10092260	10092260	G	A	WWC3	intronic	NA	NA	368	81	449	0.180401	108	1	109	0.009174	0.52	Sub	0.7	3.61			polymorphism	0.08						
OVA_048_M1	METASTASIS	chr2	10102642	10102642	T	A	GRHL1	exonic	ynonymou	000047216	327	216	543	0.39779	33	0	33	0	1	Clonal	0.56	3.94			disease_causing	23.3	p.V243D	Passenger	SENGER/OT	benign	Neutral	Damaging
OVA_048_M2	METASTASIS	chr2	10102642	10102642	T	A	GRHL1	exonic	ynonymou	000047216	373	188	561	0.335116	33	0	33	0	1	Clonal	0.4	3.87			disease_causing	23.3	p.V243D	Passenger	SENGER/OT	benign	Neutral	Damaging
OVA_048_M3	METASTASIS	chr2	10102642	10102642	T	A	GRHL1	exonic	ynonymou	000047216	328	199	527	0.377609	33	0	33	0	1	Clonal	0.59	3.39			disease_causing	23.3	p.V243D	Passenger	SENGER/OT	benign	Neutral	Damaging

Sample	Type	Chr	Start	End	Ref	Alt	Gene	unc.ensGen	icFunc.ens	hange.ens(	t_ref	t_alt	t_total	t_VAF	n_ref	n_alt	n_total	normal_VA	ncer_cell_f	on-based C	purity	ploidy	Cancer Gene	Driver Gene	Mutation Taster	ADD(PHRE	otein_chan	CHASM	ATHMM_Pr	OLYPHEN-	REDICTION	ICTION (cutoff=0.05)
OVA_013_M1	METASTASIS	chr19	10114779	10114779	C	T	COL5A3	exonic	ynonymou	000026482	160	54	214	0.252336	75	0	75	0	1	Clonal	0.67	3.06			disease_causing	24.9	p.A213T	Passenger	SENGER/O1	damaging	Deleterious	Damaging
OVA_013_C1	PRIMARY	chr19	10114779	10114779	C	T	COL5A3	exonic	ynonymou	000026482	238	92	330	0.278788	75	0	75	0	1	Clonal	0.72	3.1			disease_causing	24.9	p.A213T	Passenger	SENGER/O1	damaging	Deleterious	Damaging
OVA_013_C2	PRIMARY	chr19	10114779	10114779	C	T	COL5A3	exonic	ynonymou	000026482	250	94	344	0.273256	75	0	75	0	0.9	Clonal	0.87	3.05			disease_causing	24.9	p.A213T	Passenger	SENGER/O1	damaging	Deleterious	Damaging
OVA_013_C3	PRIMARY	chr19	10114779	10114779	C	T	COL5A3	exonic	ynonymou	000026482	272	147	419	0.350835	75	0	75	0	1	Clonal	0.91	3.01			disease_causing	24.9	p.A213T	Passenger	SENGER/O1	damaging	Deleterious	Damaging
OVA_048_C2	PRIMARY	chr12	10133459	10133459	C	T	CLEC12A	intronic	NA	NA	375	183	558	0.327957	20	0	20	0	0.91	Clonal	0.72	1.85			polymorphism	0.482						
OVA_047_M1	METASTASIS	chr5	10262811	10262811	C	G	CCT5	intronic	NA	NA	576	133	709	0.187588	116	0	116	0	1	Clonal	0.47	2.01			polymorphism	5.12						
OVA_047_M2	METASTASIS	chr5	10262811	10262811	C	G	CCT5	intronic	NA	NA	584	129	713	0.180926	116	0	116	0	1	Clonal	0.44	2.01			polymorphism	5.12						
OVA_047_M3	METASTASIS	chr5	10262811	10262811	C	G	CCT5	intronic	NA	NA	511	116	627	0.185008	116	0	116	0	1	Clonal	0.6	3.02			polymorphism	5.12						
OVA_047_C1	PRIMARY	chr19	10439704	10439704	A	C	RAVER1	exonic	ynonymou	000029367	217	78	295	0.264407	66	0	66	0	0.88	Sub	0.6	2.91			disease_causing	23.7	p.S141A	Passenger		damaging	Neutral	Tolerated
OVA_047_C2	PRIMARY	chr19	10439704	10439704	A	C	RAVER1	exonic	ynonymou	000029367	282	90	372	0.241935	66	0	66	0	0.76	Sub	0.64	2.76			disease_causing	23.7	p.S141A	Passenger		damaging	Neutral	Tolerated
OVA_047_C3	Contralateral ovary	chr19	10439704	10439704	A	C	RAVER1	exonic	ynonymou	000029367	221	64	285	0.224561	66	0	66	0	0.75	Sub	0.6	2.94			disease_causing	23.7	p.S141A	Passenger		damaging	Neutral	Tolerated
OVA_378_C1	PRIMARY	chr4	10515153	10515153	C	G	CLNK	exonic	ynonymou	19Q,ENSG0	407	146	553	0.264014	37	0	37	0	0.9	Clonal	0.59	2.6			polymorphism	9.817	p.E281Q	Passenger	SENGER/O1	damaging	Neutral	Damaging
OVA_378_C2	PRIMARY	chr4	10515153	10515153	C	G	CLNK	exonic	ynonymou	19Q,ENSG0	265	116	381	0.304462	37	0	37	0	1	Clonal	0.56	2.59			polymorphism	9.817	p.E281Q	Passenger	SENGER/O1	damaging	Neutral	Damaging
OVA_378_C3	PRIMARY	chr4	10515153	10515153	C	G	CLNK	exonic	ynonymou	19Q,ENSG0	298	98	396	0.247475	37	0	37	0	0.97	Clonal	0.51	2.6			polymorphism	9.817	p.E281Q	Passenger	SENGER/O1	damaging	Neutral	Damaging
OVA_047_C1	PRIMARY	chr18	10689683	10689683	G	A	PIEZO2	exonic	ionymous S	302079:exc	435	196	631	0.310618	31	0	31	0	1	Clonal	0.6	2.91			disease_causing	10.46		Passenger			Neutral	Tolerated
OVA_047_C2	PRIMARY	chr18	10689683	10689683	G	A	PIEZO2	exonic	ionymous S	302079:exc	436	191	627	0.304625	31	0	31	0	0.95	Clonal	0.64	2.76			disease_causing	10.46		Passenger			Neutral	Tolerated
OVA_047_C3	Contralateral ovary	chr18	10689683	10689683	G	A	PIEZO2	exonic	ionymous S	302079:exc	394	259	653	0.396631	31	0	31	0	1	Clonal	0.6	2.94			disease_causing	10.46		Passenger			Neutral	Tolerated
OVA_048_C2	PRIMARY	chr16	11001643	11001643	C	T	CIITA	exonic	ynonymou	000324288	65	51	116	0.439655	32	0	32	0	0.78	Sub	0.72	1.85	Yes	Yes	disease_causing	22.9	p.P766L	Passenger		damaging	Deleterious	Tolerated
OVA_013_M1	METASTASIS	chr16	11217608	11217608	G	T	CLEC16A	exonic	ynonymou	/742L,ENSG	400	139	539	0.257885	100	1	101	0.009901	1	Clonal	0.67	3.06			disease_causing	25.8	p.V760L	Passenger	SENGER/O1	damaging	Neutral	Tolerated
OVA_013_C1	PRIMARY	chr16	11217608	11217608	G	T	CLEC16A	exonic	ynonymou	/742L,ENSG	425	166	591	0.28088	100	1	101	0.009901	1	Clonal	0.72	3.1			disease_causing	25.8	p.V760L	Passenger	SENGER/O1	damaging	Neutral	Tolerated
OVA_013_C2	PRIMARY	chr16	11217608	11217608	G	T	CLEC16A	exonic	ynonymou	/742L,ENSG	411	179	590	0.30339	100	1	101	0.009901	1	Clonal	0.87	3.05			disease_causing	25.8	p.V760L	Passenger	SENGER/O1	damaging	Neutral	Tolerated
OVA_013_C3	PRIMARY	chr16	11217608	11217608	G	T	CLEC16A	exonic	ynonymou	/742L,ENSG	425	189	614	0.307818	100	1	101	0.009901	0.98	Clonal	0.91	3.01			disease_causing	25.8	p.V760L	Passenger	SENGER/O1	damaging	Neutral	Tolerated
OVA_003_M1	METASTASIS	chr2	11362319	11362319	G	C	ROCK2	intronic	NA	NA	172	58	230	0.252174	55	0	55	0	0.89	Sub	0.57	1.84			polymorphism	5.908						
OVA_003_M2	METASTASIS	chr2	11362319	11362319	G	C	ROCK2	intronic	NA	NA	218	77	295	0.261017	55	0	55	0	1	Sub	0.46	1.81			polymorphism	5.908						
OVA_003_M3	METASTASIS	chr2	11362319	11362319	G	C	ROCK2	intronic	NA	NA	425	33	458	0.072052	55	0	55	0	0.76	Sub	0.34	2.67			polymorphism	5.908						
OVA_003_C1	PRIMARY	chr2	11362319	11362319	G	C	ROCK2	intronic	NA	NA	221	197	418	0.471292	55	0	55	0	1	Clonal	0.86	1.81			polymorphism	5.908						
OVA_003_C2	PRIMARY	chr2	11362319	11362319	G	C	ROCK2	intronic	NA	NA	250	204	454	0.449339	55	0	55	0	1	Clonal	0.7	3.61			polymorphism	5.908						
OVA_003_C3	PRIMARY	chr2	11362319	11362319	G	C	ROCK2	intronic	NA	NA	281	206	487	0.422998	55	0	55	0	1	Clonal	0.82	1.81			polymorphism	5.908						
OVA_365_M3	METASTASIS	chr19	11473410	11473410	G	C	LPPR2	intronic	NA	NA	186	25	211	0.118483	38	0	38	0	0.17	Sub	0.82	1.89			polymorphism	5.315						
OVA_365_M3	METASTASIS	chr19	11488921	11488921	G	A	EPOR	exonic	ionymous S	000022213	113	12	125	0.096	65	0	65	0	0.14	Sub	0.82	1.89			disease_causing	12.28		Passenger			Neutral	Tolerated
OVA_365_C1	Contralateral ovary	chr19	11488921	11488921	G	A	EPOR	exonic	ionymous S	000022213	125	53	178	0.297753	65	0	65	0	1	Clonal	0.42	1.83			disease_causing	12.28		Passenger			Neutral	Tolerated
OVA_365_C2	PRIMARY	chr19	11488921	11488921	G	A	EPOR	exonic	ionymous S	000022213	156	101	257	0.392996	65	0	65	0	1	Clonal	0.62	1.92			disease_causing	12.28		Passenger			Neutral	Tolerated
OVA_378_M1	METASTASIS	chr2	11767117	11767117	C	T	GREB1	exonic	ynonymou	00234142:	477	121	598	0.202341	103	0	103	0	0.97	Sub	0.72	2.57			polymorphism	8.004	p.L1446F	Passenger	SENGER/O1	benign	Neutral	Tolerated
OVA_378_M2	METASTASIS	chr2	11767117	11767117	C	T	GREB1	exonic	ynonymou	00234142:	481	110	591	0.186125	103	0	103	0	0.81	Sub	0.85	2.57			polymorphism	8.004	p.L1446F	Passenger	SENGER/O1	benign	Neutral	Tolerated
OVA_378_M3	METASTASIS	chr2	11767117	11767117	C	T	GREB1	exonic	ynonymou	00234142:	533	115	648	0.177469	103	0	103	0	0.89	Sub	0.67	2.57			polymorphism	8.004	p.L1446F	Passenger	SENGER/O1	benign	Neutral	Tolerated
OVA_378_C1	PRIMARY	chr2	11767117	11767117	C	T	GREB1	exonic	ynonymou	00234142:	2138	24	2163	0.011096	103	0	103	0	0.06	Sub	0.59	2.6			polymorphism	8.004	p.L1446F	Passenger	SENGER/O1	benign	Neutral	Tolerated
OVA_047_M1	METASTASIS	chr10	12139705	12139705	A	C	DHTKD1	exonic	ynonymou	000026303	390	272	662	0.410876	39	0	39	0	1	Clonal	0.47	2.01			disease_causing	20.6	p.T461P	Passenger	SENGER/O1	benign	Neutral	Tolerated
OVA_047_M2	METASTASIS	chr10	12139705	12139705	A	C	DHTKD1	exonic	ynonymou	000026303	419	253	672	0.376488	39	0	39	0	1	Clonal	0.44	2.01			disease_causing	20.6	p.T461P	Passenger	SENGER/O1	benign	Neutral	Tolerated
OVA_047_M3	METASTASIS	chr10	12139705	12139705	A	C	DHTKD1	exonic	ynonymou	000026303	295	164	459	0.357298	39	0	39	0	1	Clonal	0.6	3.02			disease_causing	20.6	p.T461P	Passenger	SENGER/O1	benign	Neutral	Tolerated
OVA_048_M1	METASTASIS	chr10	12288259	12288259	G	A	CDC123	exonic	ynonymou	V236I,ENSG	304	204	508	0.401575	48	1	49	0.020408	1	Clonal	0.56	3.94			polymorphism	21.6	p.V277I	Passenger	no weights	benign	Neutral	Tolerated
OVA_048_M2	METASTASIS	chr10	12288259	12288259	G	A	CDC123	exonic	ynonymou	V236I,ENSG	391	166	557	0.298025	48	1	49	0.020408	1	Clonal	0.4	3.87			polymorphism	21.6	p.V277I	Passenger	no weights	benign	Neutral	Tolerated
OVA_048_M3	METASTASIS	chr10	12288259	12288259	G	A	CDC123	exonic	ynonymou	V236I,ENSG	381	142	523	0.271511	48	1	49	0.020408	1	Clonal	0.59	3.39			polymorphism	21.6	p.V277I	Passenger	no weights	benign	Neutral	Tolerated
OVA_365_C1	Contralateral ovary	chr18	12329147	12329147	C	T	TUBB6	exonic	ynonymou	000005919	324	91	415	0.219277	41	0	41	0	1	Clonal	0.42	1.83			polymorphism	4.37	p.H94Y	Passenger	SENGER/O1			
OVA_378_M1	METASTASIS	chr12	12332942	12332942	A	G	LRP6	intronic	NA	NA	168	89	257	0.346304	20	0	20	0	1	Clonal	0.72	2.57			polymorphism	15.03						
OVA_378_M2	METASTASIS	chr12	12332942	12332942	A	G	LRP6	intronic	NA	NA	172	88	260	0.338462	20	0	20	0	1	Clonal	0.85	2.57			polymorphism	15.03						
OVA_378_M3	METASTASIS	chr12	12332942	12332942	A	G	LRP6	intronic	NA	NA	262	126	388	0.324742	20	0	20	0	1	Clonal	0.67	2.57			polymorphism	15.03						
OVA_378_C1	PRIMARY	chr12	12332942	12332942	A	G	LRP6	intronic	NA	NA	559	12	572	0.020979	20	0	20	0	0.09	Sub	0.72	2.57			polymorphism	15.03						
OVA_013_C1	PRIMARY	chr7	12381832	12381832	T	C	VWDE	intronic	NA	NA	378	158	536	0.294776	22	0	22	0	1	Clonal	0.72	3.1			polymorphism	9.689						
OVA_013_C2	PRIMARY	chr7	12381832	12381832	T	C	VWDE	intronic	NA	NA	291	128	419	0.305489	22	0	22	0	1	Clonal	0.87	3.05			polymorphism	9.689						
OVA_013_C3	PRIMARY	chr7	12381832	12381832	T	C	VWDE	intronic	NA	NA	379	162	541	0.299445	22	0	22	0	0.95	Clonal	0.91	3.01			polymorphism	9.689						
OVA_047_M1	METASTASIS	chr3	12475528	12475528	A	G	PPARG	exonic	ynonymou	440A,ENSG	496	139	635	0.218898	56	0	56	0	0.92	Clonal	0.47	2.01	Yes	Yes	disease_causing	18.75	p.T468A	Passenger	SENGER/O1	damaging	Neutral	Tolerated
OVA_047_M2	METASTASIS	chr3	12475528	12475528	A	G	PPARG	exonic	ynonymou	440A,ENSG	528	137	665	0.206015	56	0	56	0	0.93	Clonal	0.44	2.01	Yes	Yes	disease_causing	18.75	p.T468A	Passenger	SENGER/O1	damaging	Neutral	Tolerated
OVA_047_M3	METASTASIS	chr3	12475528	12475528	A	G	PPARG	exonic	ynonymou	440A,ENSG	282	86	368	0.233696	56	0	56	0	1	Clonal	0.6	3.02	Yes	Yes	disease_causing	18.75	p.T468A	Passenger	SENGER/O1	damaging	Neutral	Tolerated
OVA_378_C1	PRIMARY	chr8	12589322	12589322	T	G	LONRF1	exonic	ynonymou	000039824	496	125	621	0.201288	46	0	46	0	0.89	Clonal	0.59	2.6			polymorphism	17.09	p.E537D	Passenger	SENGER/O1	benign	Neutral	Tolerated
OVA_047_M1	METASTASIS	chr19	12783761	12783761	G	C	WDR83	intronic	NA	NA	76	36	112	0.321429	27	0	27	0	1	Clonal	0.47	2.01			polymorphism	5.305						
OVA_047_M2	METASTASIS	chr19	12783761	12783761	G	C	WDR83	intronic	NA	NA	116	42	158	0.265823	27	0	27	0	0.93	Clonal	0.44	2.01			polymorphism	5.305						
OVA_047_M3	METASTASIS	chr19	12783761	12783761	G	C	WDR83	intronic	NA	NA	28	14	42	0.333333	27	0	27	0	1	Clonal	0.6	3.02			polymorphism	5.305						
OVA_047_M1	METASTASIS	chrX	12809717	12809717	A	T	PRPS2	exonic	ynonymou	K34M,ENSG	88	25	113	0.221239	34	0	34	0	0.71	Sub	0.47	2.01			disease_causing	25.3	p.K34M	Passenger	CANCER	damaging	Deleterious	Damaging
OVA_047_M2	METASTASIS	chrX	12809717	12809717	A	T	PRPS2	exonic	ynonymou	K34M,ENSG	76	26	102	0.254902	34	0	34	0	0.89	Sub	0.44	2.01			disease_causing	25.3	p.K34M	Passenger	CANCER	damaging	Deleterious	Damaging
OVA_047_M3	METASTASIS	chrX	12809717	12809717	A	T	PRPS2	exonic	ynonymou	K34M,ENSG	44	6	50	0.12	34	0	34	0	0.4	Sub	0.6	3.02			disease_causing	25.3	p.K34M	Passenger	CANCER	damaging	Deleterious	Damaging
OVA_365_C2	PRIMARY	chr19	13003023	13003023	G	C	GCDH	intronic	NA	NA	244	93	337	0.275964	54	0	54	0	0.9	Clonal	0.62	1.92			polymorphism	1.014						
OVA_047_M1	METASTASIS	chr19	13041385	13041385	T	A	FARSA	RNA_intro	NA	NA	152	93	245	0.379592	86	0	86	0	1	Clonal	0.47	2.01			polymorphism	1.727						
OVA_047_M2	METASTASIS	chr19	13041385	13041385	T	A	FARSA	RNA_intro	NA	NA	188	81	269	0.301115	86	0	86	0	1	Clonal	0.44	2.01			polymorphism	1.727						
OVA_047_M3	METASTASIS	chr19	13041385	13041385	T	A	FARSA	RNA_intro	NA	NA	64	35	99	0.353535	86	0	86	0	1	Clonal	0.6	3.02			polymorphism	1.727						
OVA_048_M1	METASTASIS	chr12	13154645	13154645	C	A	HTR7P1	cRNA_exon	NA	NA	26	22	48	0.458333	53	0	53	0	1	Clonal	0.56	3.94				13.09						
OVA_048_M2	METASTASIS	chr12	13154645	13154645	C	A	HTR7P1	cRNA_exon	NA	NA	37	21	58	0.362069	53	0	53	0	1	Clonal	0.4	3.87				13.09						
OVA_048_M3	METASTASIS	chr12	13154645	13154645	C	A	HTR7P1	cRNA_exon	NA	NA	30	24	54	0.444444	53	0	53	0	1	Clonal	0.59	3.39				13.09						
OVA_048_C1	PRIMARY	chr12	13154645	13154645	C	A	HTR7P1	cRNA_exon	NA	NA	20	15	35	0.428571	53	0	53	0	1	Clonal	0.67	1.82				13.09						
OVA_048_C2	PRIMARY	chr12	13154645	13154645	C	A	HTR7P1	cRNA_exon	NA	NA	35	26	61	0.42623	53	0	53	0	1	Clonal	0.72	1.85				13.09						
OVA_048_C3	PRIMARY	chr12	13154645	13154645	C	A	HTR7P1	cRNA_exon	NA	NA	23	14	37	0.378378	53	0	53	0	1	Clonal	0.74	1.8				13.09						
OVA_047_C1	PRIMARY	chr8	13356984	13356984	T	A	DLC1	exonic	ynonymou	000003166	273	236	509	0.463654	47	0	47	0	1	Sub	0.6	2.91			polymorphism	14.51	p.L199F	Passenger	SENGER/O1	benign	Neutral	Damaging
OVA_047_C2	PRIMARY	chr8	13356984	13356984	T	A	DLC1	exonic	ynonymou	000003166	339	227	566	0.40106	47	0	47	0	0.85	Sub	0.64	2.76			polymorphism	14.51	p.L199F	Passenger	SENGER/O1	benign	Neutral	Damaging
OVA_047_C3	Contralateral ovary	chr8	13356984	13356984	T	A	DLC1	exonic	ynonymou	000003166	285	187	472	0.396186	47	0	47	0	0.93	Clonal	0.6	2.94			polymorphism	14.51	p.L199F	Passenger	SENGER/O1	benign	Neutral	Damaging
OVA_003_C3	PRIMARY	chr3	13543189	13543189	A	T	HDAC11	exonic	ionymous S	000045864	169	18	187	0.096257	87	0	87	0	0.14	Sub	0.82	1.81			polymorphism	1.666					Neutral	NA
OVA_378_M1	METASTASIS	chr12	13716629	13716629	G	A	GRIN2B	exonic	ionymous S	000609686	418	166	584	0.284247	91	1	92	0.01087	1	Clonal	0.72	2.57			disease_causing	0.299		Passenger			Neutral	Tolerated
OVA_378_M2	METASTASIS	chr12	13716629	13716629	G	A	GRIN2B	exonic	ionymous S	000609686	433	187	620	0.301613	91	1	92	0.01087	1	Clonal	0.85	2.57			disease_causing	0.299		Passenger			Neutral	Tolerated
OVA_378_M3	METASTASIS	chr12	13716629	13716629	G	A	GRIN2B	exonic	ionymous S	000609686	418	182	600	0.303333	91	1	92	0.01087	1	Clonal	0.67	2.57			disease_causing	0.299		Passenger			Neutral	Tolerated
OVA_378_C1	PRIMARY	chr12	13716629	13716629	G	A	GRIN2B	exonic	ionymous S	000609686	469	148	617	0.23987	91	1	92	0.01087	1	Clonal	0.59	2.6			disease_causing	0.299		Passenger			Neutral	Tolerated
OVA_378_C2	PRIMARY	chr12	13716629	13716629	G	A	GRIN2B	exonic	ionymous S	000609686	449	131	580	0.225862	91	1	92	0.01087	1	Clonal	0.56	2.59			disease_causing	0.299		Passenger			Neutral	Tolerated
OVA_378_C3	PRIMARY	chr12	13716629	13716629	G	A	GRIN2B	exonic	ionymous S	000609686	437	142	579	0.24525	91	1	92	0.01087	1	Clonal	0.51	2.6			disease_causing	0.299		Passenger			Neutral	Tolerated
OVA_047_C1	PRIMARY	chr5	13811921	13811921	C	G	DNAH5	exonic	ynonymou	000265104	639	71	710	0.1	19	0	19	0	0.73	Sub	0.6	2.91			disease_causing	17.74	p.K2414N	Passenger	SENGER/O1	benign	Deleterious	Tolerated
OVA_047_C2	PRIMARY	chr5	13811921	13811921	C	G	DNAH5	exonic	ynonymou	000265104	576	93	669	0.139013	19	0	19	0	0.71	Sub	0.64	2.76			disease_causing	17.74	p.K2414N	Passenger	SENGER/O1	benign	Deleterious	Tolerated
OVA_047_C3	Contralateral ovary	chr5	13811921	13811921	C	G	DNAH5	exonic	ynonymou	000265104	603	77	680	0.113235	19	0	19	0	0.72	Sub	0.6	2.94			disease_causing	17.74	p.K2414N	Passenger	SENGER/O1	benign	Deleterious	Tolerated
OVA_047_C1	PRIMARY	chr19	13879589	13879589	G	C	MRI1	intronic	NA	NA	80	35	115	0.304348	65	0	65	0	1	Clonal	0.6	2.91			polymorphism	0.007						
OVA_047_C2	PRIMARY	chr19	13879589	13879589	G	C	MRI1	intronic	NA	NA	61	46	107	0.429907	65	0	65	0	1	Clonal	0.64	2.76			polymorphism	0.007						
OVA_047_C3	Contralateral ovary	chr19	13879589	13879589	G	C	MRI1	intronic	NA	NA	88	28	116	0.241379	65	0	65	0	0.81	Clonal	0.6	2.94			polymorphism	0.007						
OVA_003_M1	METASTASIS	chr5	13923502	13923502	T	C	DNAH5	exonic	ynonymou	000002651C	389	135	524	0.257634	41	0	41	0	0.91	Clonal	0.57	1.84			disease_causing	22.7	p.K109E	Passenger	SENGER/O1	benign	Neutral	Tolerated
OVA_003_M2	METASTASIS	chr5	13923502	13923502	T	C	DNAH5	exonic	ynonymou	000002651C	405	118	523	0.225621	41	0	41	0	0.98	Clonal	0.46	1.81			disease_causing	22.7	p.K109E	Passenger	SENGER/O1	benign	Neutral	Tolerated
OVA_003_M3	METASTASIS	chr5	13923502	13923502	T	C	DNAH5	exonic	ynonymou	000002651C	548	54	602	0.089701	41	0	41	0	0.95	Clonal	0.34	2.67			disease_causing	22.7	p.K109E	Passenger	SENGER/O1	benign	Neutral	Tolerated
OVA_003_C1	PRIMARY	chr5	13923502	13923502	T	C	DNAH5	exonic	ynonymou	000002651C	303	276	579	0.476684	41	0	41	0	1	Clonal	0.86	1.81			disease_causing	22.7	p.K109E	Passenger	SENGER/O1	benign	Neutral	Tolerated
OVA_003_C2	PRIMARY	chr5	13923502	13923502	T	C	DNAH5	exonic	ynonymou	000002651C	319	259	578	0.448097	41	0	41	0	1	Clonal	0.7	3.61			disease_causing	22.7	p.K109E	Passenger	SENGER/O1	benign	Neutral	Tolerated
OVA_003_C3	PRIMARY	chr5	13923502	13923502	T	C	DNAH5	exonic	ynonymou	000002651C	369	239	608	0.393092	41	0	41	0	0.95	Clonal	0.82	1.81			disease_causing	22.7	p.K109E	Passenger	SENGER/O1	benign	Neutral	Tolerated
OVA_048_C1	PRIMARY	chr7	13971435	13971435	G	C	ETV1	intronic	NA	NA	162	171	333	0.513514	31	0	31	0	1	Clonal	0.67	1.82	Yes		polymorphism	0.984						
OVA_048_C2	PRIMARY	chr7	13971435	13971435	G	C	ETV1	intronic	NA	NA	225	271	496	0.546371	31	0	31	0	0.97	Clonal	0.72	1.85	Yes		polymorphism	0.984						
OVA_048_C3	PRIMARY	chr7	13971435	13971435	G	C	ETV1	intronic	NA	NA	126	169	295	0.572881	31	0	31	0	0.98	Clonal	0.74	1.8	Yes		polymorphism	0.984						
OVA_048_M3	METASTASIS	chr6	13978053	13978053	G	A	RNF182	exonic	ynonymou	V235I,ENSG	466	100	566	0.176678	15	0	15	0	1	Clonal	0.59	3.39			polymorphism	17.18	p.V235I	Passenger	SENGER/O1	benign	Neutral	Tolerated
OVA_048_C2	PRIMARY	chr1	14108895	14108918	GCTCAGG	-	PRDM2	exonic	ameshift de	42del,ENSG	247	80	327	0.244648	61	0	61		0.68	Sub	0.72	1.85	Yes		disease causing		1536_Q154	Passenger			Deleterious	NA
OVA_048_M1	METASTASIS	chr19	14270308	14270308	G	T	LPHN1	exonic	ynonymou	00361434:	438	97	535	0.181308	18	0	18	0	1	Clonal	0.56	3.94			disease_causing	29.4	p.A648D	Passenger	SENGER/O1	damaging	Deleterious	Damaging
OVA_048_M2	METASTASIS	chr19	14270308	14270308	G	T	LPHN1	exonic	ynonymou	00361434:	388	114	502	0.227092	18	0	18	0	1	Clonal	0.4	3.87			disease_causing	29.4	p.A648D	Passenger	SENGER/O1	damaging	Deleterious	Damaging
OVA_048_M3	METASTASIS	chr19	14270308	14270308	G	T	LPHN1	exonic	ynonymou	00361434:	421	78	499	0.156313	18	0	18	0	1	Clonal	0.59	3.39			disease_causing	29.4	p.A648D	Passenger	SENGER/O1	damaging	Deleterious	Damaging

Sample	Type	Chr	Start	End	Ref	Alt	Gene	unc.ensGen	icFunc.ens	hange.ens	t_ref	t_alt	t_total	t_VAF	n_ref	n_alt	n_total	normal_VA	ncer_cell_f	on-based C	purity	ploidy	Cancer Gene	Driver Gene	Mutation Taster	ADD(PHRE	otein_chan	CHASM	ATHMM_Pr	POLYPHEN-	REDICTION	ICTION (cutoff=0.05)
OVA_047_M1	METASTASIS	chr5	14487991	14487991	C	A	TRIO	exonic	nonymous S	151P,ENSG	87	1	88	0.011364	38	0	38	0	0.07	Sub	0.47	2.01			disease_causing	13.16		Passenger			Neutral	Tolerated
OVA_047_C1	PRIMARY	chr5	14487991	14487991	C	A	TRIO	exonic	nonymous S	151P,ENSG	50	32	82	0.390244	38	0	38	0	1	Clonal	0.6	2.91			disease_causing	13.16		Passenger			Neutral	Tolerated
OVA_047_C2	PRIMARY	chr5	14487991	14487991	C	A	TRIO	exonic	nonymous S	151P,ENSG	45	30	75	0.4	38	0	38	0	1	Clonal	0.64	2.76			disease_causing	13.16		Passenger			Neutral	Tolerated
OVA_047_C3	Contralateral ovary	chr5	14487991	14487991	C	A	TRIO	exonic	nonymous S	151P,ENSG	39	25	64	0.390625	38	0	38	0	1	Clonal	0.6	2.94			disease_causing	13.16		Passenger			Neutral	Tolerated
OVA_365_M3	METASTASIS	chr12	14609563	14609563	T	G	ATF7IP	exonic	nonymous S	000053644	448	68	516	0.131783	31	0	31	0	0.32	Sub	0.82	1.89			disease_causing	1.614		Passenger			Neutral	Tolerated
OVA_003_M1	METASTASIS	chr16	14722029	14722029	C	T	PARN	exonic	nonymous S	000005392	393	164	557	0.294434	76	0	76	0	1	Sub	0.57	1.84			disease_causing	19.74		Passenger			Neutral	Tolerated
OVA_003_M2	METASTASIS	chr16	14722029	14722029	C	T	PARN	exonic	nonymous S	000005392	458	118	576	0.204861	76	0	76	0	0.89	Sub	0.46	1.81			disease_causing	19.74		Passenger			Neutral	Tolerated
OVA_003_M3	METASTASIS	chr16	14722029	14722029	C	T	PARN	exonic	nonymous S	000005392	594	39	633	0.061611	76	0	76	0	0.65	Sub	0.34	2.67			disease_causing	19.74		Passenger			Neutral	Tolerated
OVA_003_C1	PRIMARY	chr16	14722029	14722029	C	T	PARN	exonic	nonymous S	000005392	330	245	575	0.426087	76	0	76	0	0.99	Clonal	0.86	1.81			disease_causing	19.74		Passenger			Neutral	Tolerated
OVA_003_C2	PRIMARY	chr16	14722029	14722029	C	T	PARN	exonic	nonymous S	000005392	378	244	622	0.392283	76	0	76	0	1	Clonal	0.7	3.61			disease_causing	19.74		Passenger			Neutral	Tolerated
OVA_003_C3	PRIMARY	chr16	14722029	14722029	C	T	PARN	exonic	nonymous S	000005392	379	230	609	0.377668	76	0	76	0	0.92	Clonal	0.82	1.81			disease_causing	19.74		Passenger			Neutral	Tolerated
OVA_365_M1	METASTASIS	chr3	14724369	14724369	C	G	C3orf20	exonic	ynonymou	000002536	342	94	436	0.215596	68	0	68	0	0.51	Sub	0.85	1.87			disease_causing	24.4	p.T50S	Passenger	SENGER/OT	damaging	Neutral	Damaging
OVA_047_C1	PRIMARY	chr9	14775888	14775888	A	G	FREM1	exonic	ynonymou	86P,ENSG0	593	82	675	0.121481	90	0	90	0	0.77	Sub	0.6	2.91			polymorphism	17.34	p.S1586P	Passenger	SENGER/OT	damaging	Deleterious	Damaging
OVA_047_C2	PRIMARY	chr9	14775888	14775888	A	G	FREM1	exonic	ynonymou	86P,ENSG0	558	99	657	0.150685	90	0	90	0	0.77	Sub	0.64	2.76			polymorphism	17.34	p.S1586P	Passenger	SENGER/OT	damaging	Deleterious	Damaging
OVA_047_C3	Contralateral ovary	chr9	14775888	14775888	A	G	FREM1	exonic	ynonymou	86P,ENSG0	601	67	668	0.100299	90	0	90	0	0.74	Sub	0.6	2.94			polymorphism	17.34	p.S1586P	Passenger	SENGER/OT	damaging	Deleterious	Damaging
OVA_048_C1	PRIMARY	chr19	15574993	15574993	G	C	RASAL3	exonic	nonymous S	000003436	68	16	84	0.190476	17	0	17	0	0.95	Clonal	0.67	1.82			disease_causing	15.45		Passenger			Neutral	Tolerated
OVA_048_C3	PRIMARY	chr19	15574993	15574993	G	C	RASAL3	exonic	nonymous S	000003436	126	27	153	0.176471	17	0	17	0	1	Clonal	0.74	1.8			disease_causing	15.45		Passenger			Neutral	Tolerated
OVA_003_M1	METASTASIS	chr16	15596128	15596128	A	G	C16orf45	UTR5	NA	NA	425	120	545	0.220183	45	0	45	0	0.78	Sub	0.57	1.84			polymorphism	6.308						
OVA_003_M2	METASTASIS	chr16	15596128	15596128	A	G	C16orf45	UTR5	NA	NA	437	104	541	0.192237	45	0	45	0	0.84	Sub	0.46	1.81			polymorphism	6.308						
OVA_003_M3	METASTASIS	chr16	15596128	15596128	A	G	C16orf45	UTR5	NA	NA	539	40	579	0.069085	45	0	45	0	0.73	Sub	0.34	2.67			polymorphism	6.308						
OVA_003_C1	PRIMARY	chr16	15596128	15596128	A	G	C16orf45	UTR5	NA	NA	354	216	570	0.378947	45	0	45	0	0.88	Sub	0.86	1.81			polymorphism	6.308						
OVA_003_C2	PRIMARY	chr16	15596128	15596128	A	G	C16orf45	UTR5	NA	NA	340	223	563	0.396092	45	0	45	0	1	Sub	0.7	3.61			polymorphism	6.308						
OVA_003_C3	PRIMARY	chr16	15596128	15596128	A	G	C16orf45	UTR5	NA	NA	357	249	606	0.410891	45	0	45	0	1	Sub	0.82	1.81			polymorphism	6.308						
OVA_048_M1	METASTASIS	chr19	15636264	15636264	G	T	CYP4F22	exonic	nonymous S	L39L,ENSG	161	24	185	0.12973	11	0	11	0	1	Clonal	0.56	3.94			polymorphism	7.099		Passenger			Neutral	Tolerated
OVA_048_M2	METASTASIS	chr19	15636264	15636264	G	T	CYP4F22	exonic	nonymous S	L39L,ENSG	130	23	153	0.150327	11	0	11	0	1	Clonal	0.4	3.87			polymorphism	7.099		Passenger			Neutral	Tolerated
OVA_048_M3	METASTASIS	chr19	15636264	15636264	G	T	CYP4F22	exonic	nonymous S	L39L,ENSG	134	16	150	0.106667	11	0	11	0	1	Clonal	0.59	3.39			polymorphism	7.099		Passenger			Neutral	Tolerated
OVA_048_C1	PRIMARY	chr19	15636264	15636264	G	T	CYP4F22	exonic	nonymous S	L39L,ENSG	54	7	61	0.114754	11	0	11	0	0.57	Sub	0.67	1.82			polymorphism	7.099		Passenger			Neutral	Tolerated
OVA_048_C2	PRIMARY	chr19	15636264	15636264	G	T	CYP4F22	exonic	nonymous S	L39L,ENSG	139	25	164	0.152439	11	0	11	0	0.88	Sub	0.72	1.85			polymorphism	7.099		Passenger			Neutral	Tolerated
OVA_048_C3	PRIMARY	chr19	15636264	15636264	G	T	CYP4F22	exonic	nonymous S	L39L,ENSG	88	23	111	0.207207	11	0	11	0	1	Sub	0.74	1.8			polymorphism	7.099		Passenger			Neutral	Tolerated
OVA_013_M1	METASTASIS	chr16	15847321	15847321	C	T	MYH11	exonic	nonymous S	598P,ENSG	352	127	479	0.265136	17	0	17	0	1	Clonal	0.67	3.06	Yes		disease_causing	6.045		Passenger			Neutral	Tolerated
OVA_013_C1	PRIMARY	chr16	15847321	15847321	C	T	MYH11	exonic	nonymous S	598P,ENSG	407	167	574	0.290941	17	0	17	0	1	Clonal	0.72	3.1	Yes		disease_causing	6.045		Passenger			Neutral	Tolerated
OVA_013_C2	PRIMARY	chr16	15847321	15847321	C	T	MYH11	exonic	nonymous S	598P,ENSG	387	184	571	0.322242	17	0	17	0	1	Clonal	0.87	3.05	Yes		disease_causing	6.045		Passenger			Neutral	Tolerated
OVA_013_C3	PRIMARY	chr16	15847321	15847321	C	T	MYH11	exonic	nonymous S	598P,ENSG	401	180	581	0.309811	17	0	17	0	0.99	Clonal	0.91	3.01	Yes		disease_causing	6.045		Passenger			Neutral	Tolerated
OVA_003_M1	METASTASIS	chr19	16259540	16259540	T	A	HSH2D	UTR5	NA	NA	139	66	205	0.321951	36	0	36	0	1	Sub	0.57	1.84			disease_causing	2.133						
OVA_003_M2	METASTASIS	chr19	16259540	16259540	T	A	HSH2D	UTR5	NA	NA	230	73	303	0.240924	36	0	36	0	1	Sub	0.46	1.81			disease_causing	2.133						
OVA_003_M3	METASTASIS	chr19	16259540	16259540	T	A	HSH2D	UTR5	NA	NA	336	18	354	0.050847	36	0	36	0	0.49	Sub	0.34	2.67			disease_causing	2.133						
OVA_003_C1	PRIMARY	chr19	16259540	16259540	T	A	HSH2D	UTR5	NA	NA	210	134	344	0.389535	36	0	36	0	0.9	Clonal	0.86	1.81			disease_causing	2.133						
OVA_003_C2	PRIMARY	chr19	16259540	16259540	T	A	HSH2D	UTR5	NA	NA	227	182	409	0.444988	36	0	36	0	1	Clonal	0.7	3.61			disease_causing	2.133						
OVA_003_C3	PRIMARY	chr19	16259540	16259540	T	A	HSH2D	UTR5	NA	NA	256	187	443	0.422122	36	0	36	0	1	Clonal	0.82	1.81			disease_causing	2.133						
OVA_047_C1	PRIMARY	chr1	16332739	16332739	G	T	C1orf64	exonic	ynonymou	000032945	155	51	206	0.247573	45	0	45	0	1	Clonal	0.6	2.91			polymorphism	22.4	p.K136N	Passenger	SENGER/OT	damaging	Deleterious	Damaging
OVA_047_C2	PRIMARY	chr1	16332739	16332739	G	T	C1orf64	exonic	ynonymou	000032945	162	61	223	0.273543	45	0	45	0	1	Clonal	0.64	2.76			polymorphism	22.4	p.K136N	Passenger	SENGER/OT	damaging	Deleterious	Damaging
OVA_047_C3	Contralateral ovary	chr1	16332739	16332739	G	T	C1orf64	exonic	ynonymou	000032945	130	52	182	0.285714	45	0	45	0	0.96	Clonal	0.6	2.94			polymorphism	22.4	p.K136N	Passenger	SENGER/OT	damaging	Deleterious	Damaging
OVA_013_M1	METASTASIS	chr1	17397981	17397981	C	A	PADI2	exonic	ynonymou	403C,ENSG	165	75	240	0.3125	23	0	23	0	0.93	Clonal	0.67	3.06			disease_causing	27.3	p.G519C	Passenger	SENGER/OT	damaging	Neutral	Damaging
OVA_013_C1	PRIMARY	chr1	17397981	17397981	C	A	PADI2	exonic	ynonymou	403C,ENSG	219	102	321	0.317757	23	0	23	0	0.88	Sub	0.72	3.1			disease_causing	27.3	p.G519C	Passenger	SENGER/OT	damaging	Neutral	Damaging
OVA_013_C2	PRIMARY	chr1	17397981	17397981	C	A	PADI2	exonic	ynonymou	403C,ENSG	206	106	312	0.339744	23	0	23	0	0.78	Sub	0.87	3.05			disease_causing	27.3	p.G519C	Passenger	SENGER/OT	damaging	Neutral	Damaging
OVA_013_C3	PRIMARY	chr1	17397981	17397981	C	A	PADI2	exonic	ynonymou	403C,ENSG	239	145	384	0.377604	23	0	23	0	0.83	Sub	0.91	3.01			disease_causing	27.3	p.G519C	Passenger	SENGER/OT	damaging	Neutral	Damaging
OVA_013_M1	METASTASIS	chr1	17397982	17397982	C	T	PADI2	exonic	nonymous S	L402L,ENSG	166	75	241	0.311203	23	0	23	0	0.93	Clonal	0.67	3.06			disease_causing	10.42		Passenger			Neutral	Tolerated
OVA_013_C1	PRIMARY	chr1	17397982	17397982	C	T	PADI2	exonic	nonymous S	L402L,ENSG	219	103	322	0.319876	23	0	23	0	0.89	Sub	0.72	3.1			disease_causing	10.42		Passenger			Neutral	Tolerated
OVA_013_C2	PRIMARY	chr1	17397982	17397982	C	T	PADI2	exonic	nonymous S	L402L,ENSG	206	108	314	0.343949	23	0	23	0	0.79	Sub	0.87	3.05			disease_causing	10.42		Passenger			Neutral	Tolerated
OVA_013_C3	PRIMARY	chr1	17397982	17397982	C	T	PADI2	exonic	nonymous S	L402L,ENSG	237	141	378	0.373016	23	0	23	0	0.82	Sub	0.91	3.01			disease_causing	10.42		Passenger			Neutral	Tolerated
OVA_048_C1	PRIMARY	chr19	17417210	17417210	G	A	MRPL34	UTR3	NA	NA	319	68	387	0.175711	21	1	22	0.045455	0.88	Clonal	0.67	1.82			polymorphism	9.713						
OVA_048_C3	PRIMARY	chr19	17417210	17417210	G	A	MRPL34	UTR3	NA	NA	326	76	402	0.189055	21	1	22	0.045455	0.89	Clonal	0.74	1.8			polymorphism	9.713						
OVA_047_C3	Contralateral ovary	chr11	17582550	17582550	G	C	OTOG	intronic	NA	NA	545	96	641	0.149766	60	0	60	0	1	Clonal	0.6	2.94			polymorphism	4.306						
OVA_047_M2	METASTASIS	chr19	17720772	17720772	G	C	UNC13A	exonic	nonymous S	596P,ENSG	593	77	670	0.114925	26	0	26	0	0.52	Sub	0.44	2.01			disease_causing	17.67		Passenger			Neutral	Tolerated
OVA_013_M1	METASTASIS	chr2	17860082	17860082	C	T	SMC6	intronic	NA	NA	79	268	347	0.772334	10	0	10	0	1	Clonal	0.67	3.06			polymorphism	2.318						
OVA_013_C1	PRIMARY	chr2	17860082	17860082	C	T	SMC6	intronic	NA	NA	40	218	258	0.844961	10	0	10	0	1	Clonal	0.72	3.1			polymorphism	2.318						
OVA_013_C2	PRIMARY	chr2	17860082	17860082	C	T	SMC6	intronic	NA	NA	18	171	189	0.904762	10	0	10	0	1	Clonal	0.87	3.05			polymorphism	2.318						
OVA_013_C3	PRIMARY	chr2	17860082	17860082	C	T	SMC6	intronic	NA	NA	31	191	222	0.86036	10	0	10	0	1	Clonal	0.91	3.01			polymorphism	2.318						
OVA_378_M1	METASTASIS	chr3	18004084	18004084	C	T	LOC339862	intergenic	NA	NA	102	73	175	0.417143	30	0	30	0	1	Clonal	0.72	2.57				2.4						
OVA_378_M2	METASTASIS	chr3	18004084	18004084	C	T	LOC339862	intergenic	NA	NA	70	72	142	0.507042	30	0	30	0	1	Clonal	0.85	2.57				2.4						
OVA_378_M3	METASTASIS	chr3	18004084	18004084	C	T	LOC339862	intergenic	NA	NA	147	96	243	0.395062	30	0	30	0	1	Clonal	0.67	2.57				2.4						
OVA_378_C1	PRIMARY	chr3	18004084	18004084	C	T	LOC339862	intergenic	NA	NA	142	63	205	0.307317	30	0	30	0	1	Clonal	0.59	2.6				2.4						
OVA_378_C2	PRIMARY	chr3	18004084	18004084	C	T	LOC339862	intergenic	NA	NA	107	45	152	0.296053	30	0	30	0	1	Clonal	0.56	2.59				2.4						
OVA_378_C3	PRIMARY	chr3	18004084	18004084	C	T	LOC339862	intergenic	NA	NA	109	39	148	0.263514	30	0	30	0	1	Clonal	0.51	2.6				2.4						
OVA_048_C2	PRIMARY	chr17	18111600	18111600	A	C	ALKBH5	exonic	nonymous S	R359R,ENS	37	53	90	0.588889	29	0	29	0	1	Clonal	0.72	1.85			disease_causing	12.51		Passenger			Neutral	Tolerated
OVA_047_C1	PRIMARY	chr2	18113369	18113369	C	A	KCNS3	exonic	ynonymou	365N,ENSG	528	130	658	0.197568	80	0	80	0	0.86	Sub	0.6	2.91			disease_causing	26.5	p.T365N	Passenger	SENGER/OT	damaging	Deleterious	Damaging
OVA_047_C2	PRIMARY	chr2	18113369	18113369	C	A	KCNS3	exonic	ynonymou	365N,ENSG	513	141	654	0.215596	80	0	80	0	0.89	Sub	0.64	2.76			disease_causing	26.5	p.T365N	Passenger	SENGER/OT	damaging	Deleterious	Damaging
OVA_047_C3	Contralateral ovary	chr2	18113369	18113369	C	A	KCNS3	exonic	ynonymou	365N,ENSG	488	146	634	0.230284	80	0	80	0	0.77	Sub	0.6	2.94			disease_causing	26.5	p.T365N	Passenger	SENGER/OT	damaging	Deleterious	Damaging
OVA_047_M1	METASTASIS	chr10	18250713	18250713	T	A	SLC39A12	exonic	ynonymou	55E,ENSG0	502	142	644	0.220497	58	0	58	0	0.93	Clonal	0.47	2.01			disease_causing	23.5	p.D155E	Passenger	SENGER/OT	damaging	Neutral	Tolerated
OVA_047_M2	METASTASIS	chr10	18250713	18250713	T	A	SLC39A12	exonic	ynonymou	55E,ENSG0	536	146	682	0.214076	58	0	58	0	0.97	Clonal	0.44	2.01			disease_causing	23.5	p.D155E	Passenger	SENGER/OT	damaging	Neutral	Tolerated
OVA_047_M3	METASTASIS	chr10	18250713	18250713	T	A	SLC39A12	exonic	ynonymou	55E,ENSG0	302	89	391	0.227621	58	0	58	0	0.99	Clonal	0.6	3.02			disease_causing	23.5	p.D155E	Passenger	SENGER/OT	damaging	Neutral	Tolerated
OVA_003_M1	METASTASIS	chr19	18391351	18391351	C	G	JUND	exonic	ynonymou	315T,ENSG	238	107	345	0.310145	226	0	226	0	1	Sub	0.57	1.84			disease_causing	21.6	p.S315T	Passenger	SENGER/OT	benign	Neutral	Damaging
OVA_003_M2	METASTASIS	chr19	18391351	18391351	C	G	JUND	exonic	ynonymou	315T,ENSG	390	86	476	0.180672	226	0	226	0	0.79	Sub	0.46	1.81			disease_causing	21.6	p.S315T	Passenger	SENGER/OT	benign	Neutral	Damaging
OVA_003_M3	METASTASIS	chr19	18391351	18391351	C	G	JUND	exonic	ynonymou	315T,ENSG	445	17	462	0.036797	226	0	226	0	0.35	Sub	0.34	2.67			disease_causing	21.6	p.S315T	Passenger	SENGER/OT	benign	Neutral	Damaging
OVA_003_C1	PRIMARY	chr19	18391351	18391351	C	G	JUND	exonic	ynonymou	315T,ENSG	218	186	404	0.460396	226	0	226	0	1	Clonal	0.86	1.81			disease_causing	21.6	p.S315T	Passenger	SENGER/OT	benign	Neutral	Damaging
OVA_003_C2	PRIMARY	chr19	18391351	18391351	C	G	JUND	exonic	ynonymou	315T,ENSG	278	195	473	0.412262	226	0	226	0	1	Clonal	0.7	3.61			disease_causing	21.6	p.S315T	Passenger	SENGER/OT	benign	Neutral	Damaging
OVA_003_C3	PRIMARY	chr19	18391351	18391351	C	G	JUND	exonic	ynonymou	315T,ENSG	293	185	478	0.387029	226	0	226	0	0.94	Clonal	0.82	1.81			disease_causing	21.6	p.S315T	Passenger	SENGER/OT	benign	Neutral	Damaging
OVA_048_C2	PRIMARY	chr12	18715702	18715702	A	G	PIK3C2G	exonic	ynonymou	00433979	351	192	543	0.353591	59	0	59	0	0.98	Clonal	0.72	1.85			disease_causing	25.3	p.Q1219R	Passenger	CANCER	damaging	Deleterious	Damaging
OVA_048_M1	METASTASIS	chr21	18923949	18923949	T	-	CXADR	intronic	NA	NA	265	206	471	0.437367	12	0	12		1	Clonal	0.56	3.94										
OVA_048_M2	METASTASIS	chr21	18923949	18923949	T	-	CXADR	intronic	NA	NA	276	240	516	0.465116	12	0	12		1	Clonal	0.4	3.87										
OVA_048_M3	METASTASIS	chr21	18923949	18923949	T	-	CXADR	intronic	NA	NA	276	230	506	0.454545	12	0	12		1	Clonal	0.59	3.39										
OVA_048_C1	PRIMARY	chr21	18923949	18923949	T	-	CXADR	intronic	NA	NA	533	88	621	0.141707	12	0	12		0.42	Sub	0.74	1.8			disease_causing	32	p.L853I	Passenger	SENGER/OT	damaging	NA	NA
OVA_048_C2	PRIMARY	chr21	18923949	18923949	T	-	CXADR	intronic	NA	NA	498	94	592	0.158784	12	0	12		0.44	Sub	0.74	1.8			disease_causing	32	p.L853I	Passenger	SENGER/OT	damaging	NA	NA
OVA_048_C3	PRIMARY	chr21	18923949	18923949	T	-	CXADR	intronic	NA	NA	298	497	795	0.625157	12	0	12		1	Clonal	0.74	1.8										
OVA_003_M1	METASTASIS	chr19	19297737	19297737	C	A	MEF2BNB	exonic	ynonymou	V39L,ENSG	185	58	243	0.238683	86	0	86	0	0.84	Sub	0.57	1.84			disease_causing	26.1	p.V39L	Passenger	no weights	damaging	Deleterious	Damaging
OVA_003_M2	METASTASIS	chr19	19297737	19297737	C	A	MEF2BNB	exonic	ynonymou	V39L,ENSG	235	55	290	0.189655	86	0	86	0	0.83	Sub	0.46	1.81			disease_causing	26.1	p.V39L	Passenger	no weights	damaging	Deleterious	Damaging
OVA_003_M3	METASTASIS	chr19	19297737	19297737	C	A	MEF2BNB	exonic	ynonymou	V39L,ENSG	261	14	275	0.050909	86	0	86	0	0.49	Sub	0.34	2.67			disease_causing	26.1	p.V39L	Passenger	no weights	damaging	Deleterious	Damaging
OVA_003_C1	PRIMARY	chr19	19297737	19297737	C	A	MEF2BNB	exonic	ynonymou	V39L,ENSG	166	99	265	0.373585	86	0	86	0	0.87	Clonal	0.86	1.81			disease_causing	26.1	p.V39L	Passenger	no weights	damaging	Deleterious	Damaging
OVA_003_C2	PRIMARY	chr19	19297737	19297737	C	A	MEF2BNB	exonic	ynonymou	V39L,ENSG	189	135	324	0.416667	86	0	86	0	1	Clonal	0.7	3.61			disease_causing	26.1	p.V39L	Passenger	no weights	damaging	Deleterious	Damaging
OVA_003_C3	PRIMARY	chr19	19297737	19297737	C	A	MEF2BNB	exonic	ynonymou	V39L,ENSG	212	151	363	0.415978	86	0	86	0	1	Clonal	0.82	1.81			disease_causing	26.1	p.V39L	Passenger	no weights	damaging	Deleterious	Damaging
OVA_047_M1	METASTASIS	chr20	19496156	19496156	T	G	SLC24A3	exonic	ynonymou	000003280	539	138	677	0.20384	41	0	41	0	1	Clonal	0.47	2.01			disease_causing	24	p.I99S	Passenger		benign	Deleterious	Damaging
OVA_047_M2	METASTASIS	chr20	19496156	19496156	T	G	SLC24A3	exonic	ynonymou	000003280	585	110	695	0.158273	41	0	41	0	0.87	Clonal	0.44	2.01			disease_causing	24	p.I99S	Passenger		benign	Deleterious	Damaging
OVA_047_M3	METASTASIS	chr20	19496156	19496156	T	G	SLC24A3	exonic	ynonymou	000003280	468	114	582	0.195876	41	0	41	0	1	Clonal	0.6	3.02			disease_causing	24	p.I99S	Passenger		benign	Deleterious	Damaging
OVA_013_M1	METASTASIS	chr3	19575354	19575354	T	A	KCNH8	exonic	nonymous S	000328405	353	187	540	0.346296	44	0	44	0	1	Clonal	0.67	3.06			disease_causing	3.581		Passenger			Neutral	Tolerated
OVA_013_C1	PRIMARY	chr3	19575354	19575354	T	A	KCNH8	exonic	nonymous S	000328405	343	236	579	0.407599	44	0	44	0	1	Sub	0.72	3.1			disease_causing	3.581		Passenger			Neutral	Tolerated
OVA_013_C2	PRIMARY	chr3	19575354	19575354	T	A	KCNH8	exonic	nonymous S	000328405	353	243	596	0.407718	44	0	44	0	0.94	Sub	0.87	3.05			disease_causing	3.581		Passenger			Neutral	Tolerated
OVA_013_C3	PRIMARY	chr3	19575354	19575354	T	A	KCNH8	exonic	nonymous S	000328405	364	244	608	0.401316	44	0	44	0	0.88	Sub	0.91	3.01			disease_causing	3.581		Passenger			Neutral	Tolerated
OVA_378_C3	PRIMARY	chr19	19737432	19737432	C	T	LPAR2	exonic	ynonymou	000058670	216	29	245	0.118367	90	0	90	0	0.46	Sub	0.51	2.6			disease_causing	22.7	p.R221H	Passenger	SENGER/OT	damaging	Deleterious	Damaging
OVA_013_M1	METASTASIS	chr19	19823675	19823675	G	A	ZNF14	exonic	stopgain	000034409	471	145	616	0.23539	52	0	52	0	0.94	Clonal	0.67	3.06			disease_causing	24.7	p.Q139X	Passenger			NA	NA
OVA_013_C1	PRIMARY	chr19	19823675	19823675	G	A	ZNF14	exonic	stopgain	000034409	474	176	650	0.270769	52	0	52	0	1	Sub	0.72	3.1			disease_causing	24.7	p.Q139X	Passenger			NA	NA

Sample	Type	Chr	Start	End	Ref	Alt	Gene	unc.ensGer	icFunc.ens	hange.ens	t_ref	t_alt	t_total	t_VAF	n_ref	n_alt	n_total	normal_VA	ncer_cell_f	on-based C	purity	ploidy	Cancer Gene	Driver Gene	Mutation Taster	ADD(PHRE	otein_chan	CHASM	THMM_Pr	POLYPHEN-	REDICTION	ICTION (cutoff=0.05)
OVA_013_C2	PRIMARY	chr19	19823675	19823675	G	A	ZNF14	exonic	stopgain	000034409	443	184	627	0.293461	52	0	52	0	0.97	Sub	0.87	3.05			disease_causing	24.7	p.Q139X	Passenger			NA	NA
OVA_013_C3	PRIMARY	chr19	19823675	19823675	G	A	ZNF14	exonic	stopgain	000034409	497	176	673	0.261516	52	0	52	0	0.83	Sub	0.91	3.01			disease_causing	24.7	p.Q139X	Passenger			NA	NA
OVA_003_M1	METASTASIS	chr8	20112393	20112393	C	T	LZTS1	exonic	nonymous S	0000052229	185	60	245	0.244898	44	0	44	0	0.87	Sub	0.57	1.84			disease_causing	3.952		Passenger			Neutral	Tolerated
OVA_003_M2	METASTASIS	chr8	20112393	20112393	C	T	LZTS1	exonic	nonymous S	0000052229	237	63	300	0.21	44	0	44	0	0.92	Sub	0.46	1.81			disease_causing	3.952		Passenger			Neutral	Tolerated
OVA_003_M3	METASTASIS	chr8	20112393	20112393	C	T	LZTS1	exonic	nonymous S	0000052229	311	21	332	0.063253	44	0	44	0	0.67	Sub	0.34	2.67			disease_causing	3.952		Passenger			Neutral	Tolerated
OVA_003_C1	PRIMARY	chr8	20112393	20112393	C	T	LZTS1	exonic	nonymous S	0000052229	188	161	349	0.461318	44	0	44	0	1	Clonal	0.86	1.81			disease_causing	3.952		Passenger			Neutral	Tolerated
OVA_003_C2	PRIMARY	chr8	20112393	20112393	C	T	LZTS1	exonic	nonymous S	0000052229	242	142	384	0.369792	44	0	44	0	1	Clonal	0.7	3.61			disease_causing	3.952		Passenger			Neutral	Tolerated
OVA_003_C3	PRIMARY	chr8	20112393	20112393	C	T	LZTS1	exonic	nonymous S	0000052229	222	135	357	0.378151	44	0	44	0	0.92	Clonal	0.82	1.81			disease_causing	3.952		Passenger			Neutral	Tolerated
OVA_013_C2	PRIMARY	chr22	20138232	20138232	C	G	LOC388849	exonic	ynonymous	T000005400	47	6	53	0.113208	13	0	13	0	0.37	Sub	0.87	3.05				17.11	p.R93T	Passenger			Neutral	Damaging
OVA_013_M1	METASTASIS	chr10	20290838	20290838	G	A	PLXDC2	exonic	ynonymous	G83S,ENSG	470	148	618	0.239482	32	0	32	0	1	Clonal	0.67	3.06			polymorphism	13.46	p.G83S	Passenger	SENGER/OT	benign	Neutral	Tolerated
OVA_378_M1	METASTASIS	chr14	20345224	20345224	C	T	OR4K2	exonic	nonymous S	0000029864	417	154	571	0.269702	57	0	57	0	0.75	Sub	0.72	2.57			polymorphism	0.333		Passenger			Neutral	Tolerated
OVA_378_M2	METASTASIS	chr14	20345224	20345224	C	T	OR4K2	exonic	nonymous S	0000029864	388	179	567	0.315697	57	0	57	0	0.74	Sub	0.85	2.57			polymorphism	0.333		Passenger			Neutral	Tolerated
OVA_378_M3	METASTASIS	chr14	20345224	20345224	C	T	OR4K2	exonic	nonymous S	0000029864	469	162	631	0.256735	57	0	57	0	0.77	Sub	0.67	2.57			polymorphism	0.333		Passenger			Neutral	Tolerated
OVA_378_C1	PRIMARY	chr14	20345224	20345224	C	T	OR4K2	exonic	nonymous S	0000029864	475	140	615	0.227642	57	0	57	0	0.78	Sub	0.59	2.6			polymorphism	0.333		Passenger			Neutral	Tolerated
OVA_378_C2	PRIMARY	chr14	20345224	20345224	C	T	OR4K2	exonic	nonymous S	0000029864	490	105	595	0.176471	57	0	57	0	0.63	Sub	0.56	2.59			polymorphism	0.333		Passenger			Neutral	Tolerated
OVA_378_C3	PRIMARY	chr14	20345224	20345224	C	T	OR4K2	exonic	nonymous S	0000029864	469	114	583	0.19554	57	0	57	0	0.77	Sub	0.51	2.6			polymorphism	0.333		Passenger			Neutral	Tolerated
OVA_047_C1	PRIMARY	chr1	20470007	20470007	G	A	PLA2G2F	exonic	ynonymous	0000037510	322	118	440	0.268182	55	0	55	0	0.89	Clonal	0.6	2.91			disease_causing	23.6	p.A80T	Passenger	SENGER/OT	damaging	Neutral	Damaging
OVA_047_C2	PRIMARY	chr1	20470007	20470007	G	A	PLA2G2F	exonic	ynonymous	0000037510	298	136	434	0.313364	55	0	55	0	0.98	Clonal	0.64	2.76			disease_causing	23.6	p.A80T	Passenger	SENGER/OT	damaging	Neutral	Damaging
OVA_047_C3	Contralateral ovary	chr1	20470007	20470007	G	A	PLA2G2F	exonic	ynonymous	0000037510	248	116	364	0.318681	55	0	55	0	1	Clonal	0.6	2.94			disease_causing	23.6	p.A80T	Passenger	SENGER/OT	damaging	Neutral	Damaging
OVA_003_M1	METASTASIS	chr1	20503875	20503875	G	T	A2G2C;UBX	UTR5	NA	NA	94	154	248	0.620968	38	0	38	0	1	Clonal	0.57	1.84			polymorphism	6.866						
OVA_003_M2	METASTASIS	chr1	20503875	20503875	G	T	A2G2C;UBX	UTR5	NA	NA	141	134	275	0.487273	38	0	38	0	1	Clonal	0.46	1.81			polymorphism	6.866						
OVA_003_M3	METASTASIS	chr1	20503875	20503875	G	T	A2G2C;UBX	UTR5	NA	NA	270	43	313	0.13738	38	0	38	0	1	Clonal	0.34	2.67			polymorphism	6.866						
OVA_003_C1	PRIMARY	chr1	20503875	20503875	G	T	A2G2C;UBX	UTR5	NA	NA	30	331	361	0.916898	38	0	38	0	1	Clonal	0.86	1.81			polymorphism	6.866						
OVA_003_C2	PRIMARY	chr1	20503875	20503875	G	T	A2G2C;UBX	UTR5	NA	NA	49	364	413	0.881356	38	0	38	0	1	Clonal	0.7	3.61			polymorphism	6.866						
OVA_003_C3	PRIMARY	chr1	20503875	20503875	G	T	A2G2C;UBX	UTR5	NA	NA	68	442	510	0.866667	38	0	38	0	1	Clonal	0.82	1.81			polymorphism	6.866						
OVA_048_M1	METASTASIS	chr1	20680522	20680542	GGCCACGGT	-	VWA5B1	exonic	ameshift de	19del,ENSG	141	50	191	0.26178	30	0	30		1	Clonal	0.56	3.94			disease causing		L144_G115	Passenger			Deleterious	NA
OVA_048_M3	METASTASIS	chr1	20680522	20680542	GGCCACGGT	-	VWA5B1	exonic	ameshift de	19del,ENSG	120	48	168	0.285714	30	0	30		1	Clonal	0.59	3.39			disease causing		L144_G115	Passenger			Deleterious	NA
OVA_048_C2	PRIMARY	chr1	20680522	20680542	GGCCACGGT	-	VWA5B1	exonic	ameshift de	19del,ENSG	110	50	160	0.3125	30	0	30		0.87	Sub	0.72	1.85			disease causing		L144_G115	Passenger			Deleterious	NA
OVA_048_C3	PRIMARY	chr1	20680522	20680542	GGCCACGGT	-	VWA5B1	exonic	ameshift de	19del,ENSG	39	27	66	0.409091	30	0	30		0.7	Sub	0.74	1.8			disease causing		L144_G115	Passenger			Deleterious	NA
OVA_003_M1	METASTASIS	chr1	20811782	20811782	C	A	CAMK2N1	exonic	ynonymous	T000003750	62	72	134	0.537313	65	0	65	0	1	Clonal	0.57	1.84			polymorphism	26.9	p.D31Y	Passenger	no weights	damaging	Deleterious	Damaging
OVA_003_M2	METASTASIS	chr1	20811782	20811782	C	A	CAMK2N1	exonic	ynonymous	T000003750	82	68	150	0.453333	65	0	65	0	1	Clonal	0.46	1.81			polymorphism	26.9	p.D31Y	Passenger	no weights	damaging	Deleterious	Damaging
OVA_003_M3	METASTASIS	chr1	20811782	20811782	C	A	CAMK2N1	exonic	ynonymous	T000003750	91	15	106	0.141509	65	0	65	0	1	Clonal	0.34	2.67			polymorphism	26.9	p.D31Y	Passenger	no weights	damaging	Deleterious	Damaging
OVA_003_C1	PRIMARY	chr1	20811782	20811782	C	A	CAMK2N1	exonic	ynonymous	T000003750	14	127	141	0.900709	65	0	65	0	1	Clonal	0.86	1.81			polymorphism	26.9	p.D31Y	Passenger	no weights	damaging	Deleterious	Damaging
OVA_003_C2	PRIMARY	chr1	20811782	20811782	C	A	CAMK2N1	exonic	ynonymous	T000003750	19	170	189	0.899471	65	0	65	0	1	Clonal	0.7	3.61			polymorphism	26.9	p.D31Y	Passenger	no weights	damaging	Deleterious	Damaging
OVA_003_C3	PRIMARY	chr1	20811782	20811782	C	A	CAMK2N1	exonic	ynonymous	T000003750	32	197	229	0.860262	65	0	65	0	1	Clonal	0.82	1.81			polymorphism	26.9	p.D31Y	Passenger	no weights	damaging	Deleterious	Damaging
OVA_003_M1	METASTASIS	chr19	20844322	20844322	C	A	ZNF626	cRNA_exon	NA	NA	187	84	271	0.309963	114	1	115	0.008696	1	Sub	0.57	1.84			polymorphism	2.678						
OVA_003_M2	METASTASIS	chr19	20844322	20844322	C	A	ZNF626	cRNA_exon	NA	NA	317	107	424	0.252358	114	1	115	0.008696	1	Sub	0.46	1.81			polymorphism	2.678						
OVA_003_M3	METASTASIS	chr19	20844322	20844322	C	A	ZNF626	cRNA_exon	NA	NA	335	21	356	0.058989	114	1	115	0.008696	0.62	Sub	0.34	2.67			polymorphism	2.678						
OVA_003_C1	PRIMARY	chr19	20844322	20844322	C	A	ZNF626	cRNA_exon	NA	NA	202	156	358	0.435754	114	1	115	0.008696	1	Clonal	0.86	1.81			polymorphism	2.678						
OVA_003_C2	PRIMARY	chr19	20844322	20844322	C	A	ZNF626	cRNA_exon	NA	NA	234	165	399	0.413534	114	1	115	0.008696	1	Clonal	0.7	3.61			polymorphism	2.678						
OVA_003_C3	PRIMARY	chr19	20844322	20844322	C	A	ZNF626	cRNA_exon	NA	NA	239	167	406	0.41133	114	1	115	0.008696	1	Clonal	0.82	1.81			polymorphism	2.678						
OVA_003_M1	METASTASIS	chr14	20917205	20917205	T	C	OSGEP	UTR5	NA	NA	358	128	486	0.263374	51	1	52	0.019231	0.93	Sub	0.57	1.84			polymorphism	1.776						
OVA_003_M2	METASTASIS	chr14	20917205	20917205	T	C	OSGEP	UTR5	NA	NA	464	90	554	0.162455	51	1	52	0.019231	0.71	Sub	0.46	1.81			polymorphism	1.776						
OVA_003_M3	METASTASIS	chr14	20917205	20917205	T	C	OSGEP	UTR5	NA	NA	523	39	562	0.069395	51	1	52	0.019231	0.73	Sub	0.34	2.67			polymorphism	1.776						
OVA_003_C1	PRIMARY	chr14	20917205	20917205	T	C	OSGEP	UTR5	NA	NA	292	264	556	0.47482	51	1	52	0.019231	1	Clonal	0.86	1.81			polymorphism	1.776						
OVA_003_C2	PRIMARY	chr14	20917205	20917205	T	C	OSGEP	UTR5	NA	NA	322	240	562	0.427046	51	1	52	0.019231	1	Clonal	0.7	3.61			polymorphism	1.776						
OVA_003_C3	PRIMARY	chr14	20917205	20917205	T	C	OSGEP	UTR5	NA	NA	352	215	567	0.379189	51	1	52	0.019231	0.92	Clonal	0.82	1.81			polymorphism	1.776						
OVA_047_C1	PRIMARY	chr16	21036038	21036038	G	A	DNAH3	intronic	NA	NA	575	41	616	0.066558	58	0	58	0	0.29	Sub	0.6	2.91			polymorphism	11.57						
OVA_047_C1	PRIMARY	chr2	21227159	21227159	G	T	APOB	exonic	ynonymous	000233242:	592	109	701	0.155492	58	1	59	0.016949	0.67	Sub	0.6	2.91			polymorphism	8.121	p.N4023K	Passenger	SENGER/OT	benign	Neutral	Tolerated
OVA_047_C2	PRIMARY	chr2	21227159	21227159	G	T	APOB	exonic	ynonymous	000233242:	574	98	672	0.145833	58	1	59	0.016949	0.6	Sub	0.64	2.76			polymorphism	8.121	p.N4023K	Passenger	SENGER/OT	benign	Neutral	Tolerated
OVA_047_C3	Contralateral ovary	chr2	21227159	21227159	G	T	APOB	exonic	ynonymous	000233242:	543	126	669	0.188341	58	1	59	0.016949	1	Clonal	0.6	2.94			polymorphism	8.121	p.N4023K	Passenger	SENGER/OT	benign	Neutral	Tolerated
OVA_048_M1	METASTASIS	chr20	21337313	21337313	A	T	XRN2	intronic	NA	NA	149	70	219	0.319635	9	0	9	0	1	Clonal	0.56	3.94			polymorphism	1.713						
OVA_048_M2	METASTASIS	chr20	21337313	21337313	A	T	XRN2	intronic	NA	NA	151	66	217	0.304147	9	0	9	0	1	Clonal	0.4	3.87			polymorphism	1.713						
OVA_048_M3	METASTASIS	chr20	21337313	21337313	A	T	XRN2	intronic	NA	NA	135	43	178	0.241573	9	0	9	0	1	Clonal	0.59	3.39			polymorphism	1.713						
OVA_048_C1	PRIMARY	chr20	21337313	21337313	A	T	XRN2	intronic	NA	NA	108	62	170	0.364706	9	0	9	0	1	Sub	0.67	1.82			polymorphism	1.713						
OVA_048_C2	PRIMARY	chr20	21337313	21337313	A	T	XRN2	intronic	NA	NA	178	69	247	0.279352	9	0	9	0	0.78	Sub	0.72	1.85			polymorphism	1.713						
OVA_048_C3	PRIMARY	chr20	21337313	21337313	A	T	XRN2	intronic	NA	NA	112	61	173	0.352601	9	0	9	0	0.95	Sub	0.74	1.8			polymorphism	1.713						
OVA_378_M1	METASTASIS	chr22	21348253	21348253	C	T	LZTR1	exonic	ynonymous	A446V,ENSG	222	130	352	0.369318	67	0	67	0	1	Clonal	0.72	2.57	Yes	Yes	disease_causing	24.6	p.A465V	Passenger	SENGER/OT	damaging	Deleterious	Damaging
OVA_378_M2	METASTASIS	chr22	21348253	21348253	C	T	LZTR1	exonic	ynonymous	A446V,ENSG	166	109	275	0.396364	67	0	67	0	0.93	Clonal	0.85	2.57	Yes	Yes	disease_causing	24.6	p.A465V	Passenger	SENGER/OT	damaging	Deleterious	Damaging
OVA_378_M3	METASTASIS	chr22	21348253	21348253	C	T	LZTR1	exonic	ynonymous	A446V,ENSG	214	103	317	0.324921	67	0	67	0	0.97	Clonal	0.67	2.57	Yes	Yes	disease_causing	24.6	p.A465V	Passenger	SENGER/OT	damaging	Deleterious	Damaging
OVA_378_C1	PRIMARY	chr22	21348253	21348253	C	T	LZTR1	exonic	ynonymous	A446V,ENSG	560	19	580	0.032759	67	0	67	0	0.08	Sub	0.59	2.6	Yes	Yes	disease_causing	24.6	p.A465V	Passenger	SENGER/OT	damaging	Deleterious	Damaging
OVA_048_M1	METASTASIS	chr9	21350877	21350877	G	T	IFNA6	exonic	ynonymous	T00000380	412	225	637	0.353218	97	1	98	0.010204	1	Clonal	0.56	3.94			polymorphism	0.008	p.P4T	Passenger	SENGER/OT	benign	Deleterious	Tolerated
OVA_048_M2	METASTASIS	chr9	21350877	21350877	G	T	IFNA6	exonic	ynonymous	T00000380	448	172	620	0.277419	97	1	98	0.010204	1	Clonal	0.4	3.87			polymorphism	0.008	p.P4T	Passenger	SENGER/OT	benign	Deleterious	Tolerated
OVA_048_M3	METASTASIS	chr9	21350877	21350877	G	T	IFNA6	exonic	ynonymous	T00000380	401	214	615	0.347967	97	1	98	0.010204	1	Clonal	0.59	3.39			polymorphism	0.008	p.P4T	Passenger	SENGER/OT	benign	Deleterious	Tolerated
OVA_048_C1	PRIMARY	chr9	21350877	21350877	G	T	IFNA6	exonic	ynonymous	T00000380	269	199	468	0.425214	97	1	98	0.010204	1	Clonal	0.67	1.82			polymorphism	0.008	p.P4T	Passenger	SENGER/OT	benign	Deleterious	Tolerated
OVA_048_C2	PRIMARY	chr9	21350877	21350877	G	T	IFNA6	exonic	ynonymous	T00000380	406	204	610	0.334426	97	1	98	0.010204	0.93	Clonal	0.72	1.85			polymorphism	0.008	p.P4T	Passenger	SENGER/OT	benign	Deleterious	Tolerated
OVA_048_C3	PRIMARY	chr9	21350877	21350877	G	T	IFNA6	exonic	ynonymous	T00000380	290	203	493	0.411765	97	1	98	0.010204	1	Clonal	0.74	1.8			polymorphism	0.008	p.P4T	Passenger	SENGER/OT	benign	Deleterious	Tolerated
OVA_047_C3	Contralateral ovary	chr18	21364033	21364033	C	T	LAMA3	exonic	nonymous S	0000399516	449	163	612	0.26634	63	0	63	0	0.89	Clonal	0.6	2.94			disease_causing	6.663		Passenger			Neutral	Tolerated
OVA_365_M1	METASTASIS	chr18	21404346	21404346	G	A	LAMA3	intronic	NA	NA	341	225	566	0.397527	58	0	58	0	0.94	Clonal	0.85	1.87			polymorphism	12.11						
OVA_365_M2	METASTASIS	chr18	21404346	21404346	G	A	LAMA3	intronic	NA	NA	383	98	481	0.203742	58	0	58	0	1	Clonal	0.4	1.81			polymorphism	12.11						
OVA_365_M3	METASTASIS	chr18	21404346	21404346	G	A	LAMA3	intronic	NA	NA	338	209	547	0.382084	58	0	58	0	0.93	Clonal	0.82	1.89			polymorphism	12.11						
OVA_365_C1	Contralateral ovary	chr18	21404346	21404346	G	A	LAMA3	intronic	NA	NA	379	108	487	0.221766	58	0	58	0	1	Clonal	0.42	1.83			polymorphism	12.11						
OVA_365_C2	PRIMARY	chr18	21404346	21404346	G	A	LAMA3	intronic	NA	NA	373	162	535	0.302804	58	0	58	0	0.98	Clonal	0.62	1.92			polymorphism	12.11						
OVA_003_M1	METASTASIS	chr18	21484002	21484015	GAAAAGCA	-	LAMA3	exonic	neshift dele	33fs,ENSG	661	147	808	0.181931	55	0	55		0.64	Sub	0.57	1.84					p.E2143Vfs	Passenger			NA	NA
OVA_003_M2	METASTASIS	chr18	21484002	21484015	GAAAAGCA	-	LAMA3	exonic	neshift dele	33fs,ENSG	905	120	1025	0.117073	55	0	55		0.51	Sub	0.46	1.81					p.E2143Vfs	Passenger			NA	NA
OVA_003_M3	METASTASIS	chr18	21484002	21484015	GAAAAGCA	-	LAMA3	exonic	neshift dele	33fs,ENSG	1032	51	1083	0.047091	55	0	55		0.5	Sub	0.34	2.67					p.E2143Vfs	Passenger			NA	NA
OVA_003_C1	PRIMARY	chr18	21484002	21484015	GAAAAGCA	-	LAMA3	exonic	neshift dele	33fs,ENSG	381	147	528	0.278409	55	0	55		0.65	Sub	0.86	1.81					p.E2143Vfs	Passenger			NA	NA
OVA_003_C2	PRIMARY	chr18	21484002	21484015	GAAAAGCA	-	LAMA3	exonic	neshift dele	33fs,ENSG	439	187	626	0.298722	55	0	55		1	Sub	0.7	3.61					p.E2143Vfs	Passenger			NA	NA
OVA_003_C3	PRIMARY	chr18	21484002	21484015	GAAAAGCA	-	LAMA3	exonic	neshift dele	33fs,ENSG	486	159	645	0.246512	55	0	55		0.6	Sub	0.82	1.81					p.E2143Vfs	Passenger			NA	NA
OVA_047_M1	METASTASIS	chr14	21500520	21500520	G	T	NDRG2	exonic	ynonymous	0000048153	501	138	639	0.215962	41	0	41	0	0.91	Sub	0.47	2.01			disease_causing	13.26					Neutral	Tolerated
OVA_047_M2	METASTASIS	chr14	21500520	21500520	G	T	NDRG2	exonic	ynonymous	0000048153	503	148	651	0.227343	41	0	41	0	1	Sub	0.44	2.01			disease_causing	13.26					Neutral	Tolerated
OVA_047_M3	METASTASIS	chr14	21500520	21500520	G	T	NDRG2	exonic	ynonymous	0000048153	334	40	374	0.106952	41	0	41	0	0.46	Sub	0.6	3.02			disease_causing	13.26					Neutral	Tolerated
OVA_047_C1	PRIMARY	chr14	21570231	21570231	C	T	TMEM253	exonic	nonymous S	0000005565	363	137	500	0.274	93	0	93	0	1	Clonal	0.6	2.91			disease_causing	19.9		Passenger			Neutral	Tolerated
OVA_047_C2	PRIMARY	chr14	21570231	21570231	C	T	TMEM253	exonic	nonymous S	0000005565	307	284	591	0.480541	93	0	93	0	1	Clonal	0.64	2.76			disease_causing	19.9		Passenger			Neutral	Tolerated
OVA_047_C3	Contralateral ovary	chr14	21570231	21570231	C	T	TMEM253	exonic	nonymous S	0000005565	340	109	449	0.242762	93	0	93	0	1	Clonal	0.6	2.94			disease_causing	19.9		Passenger			Neutral	Tolerated
OVA_378_M1	METASTASIS	chr7	21655887	21655887	G	T	DNAH11	exonic	ynonymous	1358L,ENSG	381	209	590	0.354237	67	0	67	0	0.98	Sub	0.72	2.57			polymorphism	17.99	p.V1358L	Passenger		benign	Neutral	Tolerated
OVA_378_M2	METASTASIS	chr7	21655887	21655887	G	T	DNAH11	exonic	ynonymous	1358L,ENSG	372	222	594	0.373737	67	0	67	0	0.88	Sub	0.85	2.57			polymorphism	17.99	p.V1358L	Passenger		benign	Neutral	Tolerated
OVA_378_M3	METASTASIS	chr7	21655887	21655887	G	T	DNAH11	exonic	ynonymous	1358L,ENSG	430	218	648	0.33642	67	0	67	0	1	Sub	0.67	2.57			polymorphism	17.99	p.V1358L	Passenger		benign	Neutral	Tolerated
OVA_378_C1	PRIMARY	chr7	21655887	21655887	G	T	DNAH11	exonic	ynonymous	1358L,ENSG	436	215	651	0.330261	67	0	67	0	1	Clonal	0.59	2.6			polymorphism	17.99	p.V1358L	Passenger		benign	Neutral	Tolerated
OVA_378_C2	PRIMARY	chr7	21655887	21655887	G	T	DNAH11	exonic	ynonymous	1358L,ENSG	417	213	630	0.338095	67	0	67	0	1	Clonal	0.56	2.59			polymorphism	17.99	p.V1358L	Passenger		benign	Neutral	Tolerated
OVA_378_C3	PRIMARY	chr7	21655887	21655887	G	T	DNAH11	exonic	ynonymous	1358L,ENSG	464	174	638	0.272727	67	0	67	0	1	Clonal	0.51	2.6			polymorphism	17.99	p.V1358L	Passenger		benign	Neutral	Tolerated
OVA_047_M1	METASTASIS	chrX	21675953	21675953	G	A	KLHL34	UTR5	NA	NA	214	64	278	0.230216	45	0	45	0	0.74	Sub	0.47	2.01			polymorphism	7.529						
OVA_365_M1	METASTASIS	chr18	21735998	21735998	A	C	CABYR	exonic	ynonymous	.78P,ENSG	314	267	581	0.459552	81	0	81	0	1	Sub	0.85	1.87			disease_causing	26.2	p.Q178P	Passenger		damaging	Deleterious	Damaging
OVA_365_M2	METASTASIS	chr18	21735998	21735998	A	C	CABYR	exonic	ynonymous	.78P,ENSG	393	98	491	0.199593	81	0	81	0	0.99	Sub	0.4	1.81			disease_causing	26.2	p.Q178P	Passenger		damaging	Deleterious	Damaging
OVA_365_M3	METASTASIS	chr18	21735998	21735998	A	C	CABYR	exonic	ynonymous	.78P,ENSG	362	186	548	0.339416	81	0	81	0	0.83	Sub	0.82	1.89			disease_causing	26.2	p.Q178P	Passenger		damaging	Deleterious	Damaging
OVA_003_M1	METASTASIS	chr14	21771583	21771583	G	A	RPGRIP1	exonic	ynonymous	M227I,ENSG	374	153	527	0.290323	20	0	20	0	1	Sub	0.57	1.84			polymorphism	12.74	p.M227I	Passenger	SENGER/OT	benign	Neutral	Tolerated
OVA_003_M2	METASTASIS	chr14	21771583	21771583	G	A	RPGRIP1	exonic	ynonymous	M227I,ENSG	416	117	533	0.219512	20	0	20	0	0.96	Sub	0.46	1.81			polymorphism	12.74	p.M227I	Passenger	SENGER/OT	benign	Neutral	Tolerated
OVA_003_M3	METASTASIS	chr14	21771583	21771583	G	A	RPGRIP1	exonic	ynonymous	M227I,ENSG	527	36	563	0.063943	20	0	20	0	0.67	Sub	0.34	2.67			polymorphism	12.74	p.M227I	Passenger	SENGER/OT	benign	Neutral	Tolerated
OVA_003_C1	PRIMARY	chr14	21771583	21771583	G	A	RPGRIP1	exonic	ynonymous	M227I,ENSG	310	283	593	0.477234	20	0	20	0	1	Clonal	0.86	1.81			polymorphism	12.74	p.M227I	Passenger	SENGER/OT	benign	Neutral	Tolerated
OVA_003_C2	PRIMARY	chr14	21771583	21771583	G	A	RPGRIP1	exonic	ynonymous	M227I,ENSG	337	246	583	0.421955	20	0	20	0	1	Clonal	0.7	3.61			polymorphism	12.74	p.M227I	Passenger	SENGER/OT	benign	Neutral	Tolerated

Sample	Type	Chr	Start	End	Ref	Alt	Gene	unc.ensGen	icFunc.ens	hange.ens(	t_ref	t_alt	t_total	t_VAF	n_ref	n_alt	n_total	normal_VA	ncer_cell_f	on-based C	purity	ploidy	Cancer Gene	Driver Gene	Mutation Taster	ADD(PHRE	otein_chan	CHASM	ATHMM_Pr	OLYPHEN-	REDICTION	ICTION (cutoff=0.05)
OVA_003_C3	PRIMARY	chr14	21771583	21771583	G	A	RPGRIP1	exonic	ynonymou	M227I,ENSG	342	252	594	0.424242	20	0	20	0	1	Clonal	0.82	1.81			polymorphism	12.74	p.M227I	Passenger	SENGER/O1	benign	Neutral	Tolerated
OVA_048_C3	PRIMARY	chr8	21839379	21839379	C	G	XPO7	exonic	onymous S	000433566	120	172	292	0.589041	34	0	34	0	1	Clonal	0.74	1.8			disease_causing	12.14		Passenger			Neutral	Tolerated
OVA_047_C2	PRIMARY	chr7	21934452	21934452	T	C	DNAH11	intronic	NA	NA	517	143	660	0.216667	57	0	57	0	0.68	Sub	0.64	2.76			polymorphism	3.934						
OVA_048_M1	METASTASIS	chr8	22052513	22052513	A	T	BMP1	intronic	NA	NA	88	71	159	0.446541	22	0	22	0	1	Clonal	0.56	3.94			polymorphism	2.651						
OVA_048_M2	METASTASIS	chr8	22052513	22052513	A	T	BMP1	intronic	NA	NA	149	56	205	0.273171	22	0	22	0	1	Clonal	0.4	3.87			polymorphism	2.651						
OVA_048_M3	METASTASIS	chr8	22052513	22052513	A	T	BMP1	intronic	NA	NA	135	62	197	0.314721	22	0	22	0	1	Clonal	0.59	3.39			polymorphism	2.651						
OVA_048_C1	PRIMARY	chr8	22052513	22052513	A	T	BMP1	intronic	NA	NA	39	18	57	0.315789	22	0	22	0	0.94	Clonal	0.67	1.82			polymorphism	2.651						
OVA_048_C2	PRIMARY	chr8	22052513	22052513	A	T	BMP1	intronic	NA	NA	98	39	137	0.284672	22	0	22	0	0.79	Clonal	0.72	1.85			polymorphism	2.651						
OVA_048_M1	METASTASIS	chr7	22349635	22349635	G	A	RAPGEF5	exonic	ynonymou	2V,ENSG00	327	219	546	0.401099	54	1	55	0.018182	1	Clonal	0.56	3.94			disease_causing	24.2	p.A2V	Passenger	SENGER/O1	benign	Neutral	Damaging
OVA_048_M2	METASTASIS	chr7	22349635	22349635	G	A	RAPGEF5	exonic	ynonymou	2V,ENSG00	352	221	573	0.385689	54	1	55	0.018182	1	Clonal	0.4	3.87			disease_causing	24.2	p.A2V	Passenger	SENGER/O1	benign	Neutral	Damaging
OVA_048_M3	METASTASIS	chr7	22349635	22349635	G	A	RAPGEF5	exonic	ynonymou	2V,ENSG00	346	221	567	0.389771	54	1	55	0.018182	1	Clonal	0.59	3.39			disease_causing	24.2	p.A2V	Passenger	SENGER/O1	benign	Neutral	Damaging
OVA_048_C1	PRIMARY	chr7	22349635	22349635	G	A	RAPGEF5	exonic	ynonymou	2V,ENSG00	243	159	402	0.395522	54	1	55	0.018182	1	Clonal	0.67	1.82			disease_causing	24.2	p.A2V	Passenger	SENGER/O1	benign	Neutral	Damaging
OVA_048_C2	PRIMARY	chr7	22349635	22349635	G	A	RAPGEF5	exonic	ynonymou	2V,ENSG00	315	227	542	0.418819	54	1	55	0.018182	1	Clonal	0.72	1.85			disease_causing	24.2	p.A2V	Passenger	SENGER/O1	benign	Neutral	Damaging
OVA_048_C3	PRIMARY	chr7	22349635	22349635	G	A	RAPGEF5	exonic	ynonymou	2V,ENSG00	231	161	392	0.410714	54	1	55	0.018182	1	Clonal	0.74	1.8			disease_causing	24.2	p.A2V	Passenger	SENGER/O1	benign	Neutral	Damaging
OVA_365_M1	METASTASIS	chr11	22398199	22398199	G	A	SLC17A6	exonic	ynonymou	000263160	340	232	572	0.405594	44	0	44	0	0.96	Sub	0.85	1.87			disease_causing	23.8	p.G465D	Passenger	SENGER/O1	damaging	Deleterious	Tolerated
OVA_365_M2	METASTASIS	chr11	22398199	22398199	G	A	SLC17A6	exonic	ynonymou	000263160	436	72	508	0.141732	44	0	44	0	0.7	Sub	0.4	1.81			disease_causing	23.8	p.G465D	Passenger	SENGER/O1	damaging	Deleterious	Tolerated
OVA_365_M3	METASTASIS	chr11	22398199	22398199	G	A	SLC17A6	exonic	ynonymou	000263160	339	232	571	0.406305	44	0	44	0	0.99	Sub	0.82	1.89			disease_causing	23.8	p.G465D	Passenger	SENGER/O1	damaging	Deleterious	Tolerated
OVA_365_C1	Contralateral ovary	chr11	22398199	22398199	G	A	SLC17A6	exonic	ynonymou	000263160	425	96	521	0.184261	44	0	44	0	0.88	Clonal	0.42	1.83			disease_causing	23.8	p.G465D	Passenger	SENGER/O1	damaging	Deleterious	Tolerated
OVA_365_C2	PRIMARY	chr11	22398199	22398199	G	A	SLC17A6	exonic	ynonymou	000263160	384	170	554	0.306859	44	0	44	0	1	Clonal	0.62	1.92			disease_causing	23.8	p.G465D	Passenger	SENGER/O1	damaging	Deleterious	Tolerated
OVA_048_M1	METASTASIS	chr18	22804534	22804534	T	A	ZNF521	exonic	onymous S	116P,ENSG	419	139	558	0.249104	67	0	67	0	1	Clonal	0.56	3.94	Yes		polymorphism	2.859		Passenger			Neutral	Tolerated
OVA_048_M2	METASTASIS	chr18	22804534	22804534	T	A	ZNF521	exonic	onymous S	116P,ENSG	413	132	545	0.242202	67	0	67	0	1	Clonal	0.4	3.87	Yes		polymorphism	2.859		Passenger			Neutral	Tolerated
OVA_048_M3	METASTASIS	chr18	22804534	22804534	T	A	ZNF521	exonic	onymous S	116P,ENSG	342	188	530	0.354717	67	0	67	0	1	Clonal	0.59	3.39	Yes		polymorphism	2.859		Passenger			Neutral	Tolerated
OVA_365_M1	METASTASIS	chr22	22890659	22890659	T	C	PRAME	exonic	ynonymou	000039874	21	70	91	0.769231	74	0	74	0	1	Clonal	0.85	1.87			polymorphism	10.45	p.T454A	Passenger	SENGER/O1	damaging	Neutral	Tolerated
OVA_365_M2	METASTASIS	chr22	22890659	22890659	T	C	PRAME	exonic	ynonymou	000039874	98	30	128	0.234375	74	0	74	0	0.93	Clonal	0.4	1.81			polymorphism	10.45	p.T454A	Passenger	SENGER/O1	damaging	Neutral	Tolerated
OVA_365_M3	METASTASIS	chr22	22890659	22890659	T	C	PRAME	exonic	ynonymou	000039874	32	77	109	0.706422	74	0	74	0	1	Clonal	0.82	1.89			polymorphism	10.45	p.T454A	Passenger	SENGER/O1	damaging	Neutral	Tolerated
OVA_378_M1	METASTASIS	chr14	22928635	22928635	T	C	AK093552	RNA_intro	NA	NA	427	198	625	0.3168	64	0	64	0	1	Clonal	0.72	2.57				3.536						
OVA_378_M2	METASTASIS	chr14	22928635	22928635	T	C	AK093552	RNA_intro	NA	NA	415	186	601	0.309484	64	0	64	0	1	Clonal	0.85	2.57				3.536						
OVA_378_M3	METASTASIS	chr14	22928635	22928635	T	C	AK093552	RNA_intro	NA	NA	504	175	679	0.257732	64	0	64	0	1	Clonal	0.67	2.57				3.536						
OVA_378_C1	PRIMARY	chr14	22928635	22928635	T	C	AK093552	RNA_intro	NA	NA	2616	49	2668	0.018366	64	0	64	0	0.08	Sub	0.67	2.57				3.536						
OVA_047_C3	Contralateral ovary	chr14	22978497	22978497	C	T	TRA@;TRA	downstrear	NA	NA	499	145	644	0.225155	31	0	31	0	0.98	Clonal	0.6	2.94				6.692						
OVA_365_M1	METASTASIS	chr16	23387026	23387026	G	C	SCNN1B	intronic	NA	NA	37	198	235	0.842553	57	0	57	0	1	Clonal	0.85	1.87			polymorphism	3.719						
OVA_365_M2	METASTASIS	chr16	23387026	23387026	G	C	SCNN1B	intronic	NA	NA	264	105	369	0.284553	57	0	57	0	1	Clonal	0.4	1.81			polymorphism	3.719						
OVA_365_M3	METASTASIS	chr16	23387026	23387026	G	C	SCNN1B	intronic	NA	NA	82	217	299	0.725753	57	0	57	0	1	Clonal	0.82	1.89			polymorphism	3.719						
OVA_365_C1	Contralateral ovary	chr16	23387026	23387026	G	C	SCNN1B	intronic	NA	NA	326	100	426	0.234742	57	0	57	0	1	Clonal	0.42	1.83			polymorphism	3.719						
OVA_365_C2	PRIMARY	chr16	23387026	23387026	G	C	SCNN1B	intronic	NA	NA	293	142	435	0.326437	57	0	57	0	1	Clonal	0.62	1.92			polymorphism	3.719						
OVA_365_C1	Contralateral ovary	chr14	23392467	23392467	C	T	PRMT5	cRNA_exon	NA	NA	406	132	538	0.245353	27	0	27	0	1	Clonal	0.42	1.83			polymorphism	0.864						
OVA_047_C2	PRIMARY	chrX	23398234	23398234	T	A	PTCHD1	exonic	ynonymou	000037936	444	24	468	0.051282	63	0	63	0	0.16	Sub	0.64	2.76			disease_causing	27.3	p.V293D	Passenger	SENGER/O1	damaging	Deleterious	Damaging
OVA_365_M1	METASTASIS	chr18	23720176	23720176	G	C	PSMA8	intronic	NA	NA	153	115	268	0.429104	31	0	31	0	1	Clonal	0.85	1.87			polymorphism	1.719						
OVA_365_M2	METASTASIS	chr18	23720176	23720176	G	C	PSMA8	intronic	NA	NA	133	35	168	0.208333	31	0	31	0	1	Clonal	0.4	1.81			polymorphism	1.719						
OVA_365_M3	METASTASIS	chr18	23720176	23720176	G	C	PSMA8	intronic	NA	NA	150	100	250	0.4	31	0	31	0	0.98	Clonal	0.82	1.89			polymorphism	1.719						
OVA_365_C1	Contralateral ovary	chr18	23720176	23720176	G	C	PSMA8	intronic	NA	NA	148	20	168	0.119048	31	0	31	0	0.57	Sub	0.42	1.83			polymorphism	1.719						
OVA_365_C2	PRIMARY	chr18	23720176	23720176	G	C	PSMA8	intronic	NA	NA	177	68	245	0.277551	31	0	31	0	0.9	Sub	0.62	1.92			polymorphism	1.719						
OVA_048_C1	PRIMARY	chr10	23729760	23729760	C	T	OTUD1	exonic	onymous S	000037649	280	163	443	0.367946	66	0	66	0	1	Clonal	0.67	1.82			disease_causing	16.53		Passenger			Neutral	Tolerated
OVA_048_C3	PRIMARY	chr10	23729760	23729760	C	T	OTUD1	exonic	onymous S	000037649	348	180	528	0.340909	66	0	66	0	0.92	Clonal	0.74	1.8			disease_causing	16.53		Passenger			Neutral	Tolerated
OVA_365_M1	METASTASIS	chr14	23845069	23845069	C	T	IL25	exonic	ynonymou	172W,ENSG	172	120	292	0.410959	82	0	82	0	0.97	Sub	0.85	1.87			polymorphism	25.7	p.R172W	Passenger	SENGER/O1	damaging	Deleterious	Damaging
OVA_365_M2	METASTASIS	chr14	23845069	23845069	C	T	IL25	exonic	ynonymou	172W,ENSG	172	29	201	0.144279	82	0	82	0	0.72	Sub	0.4	1.81			polymorphism	25.7	p.R172W	Passenger	SENGER/O1	damaging	Deleterious	Damaging
OVA_365_M3	METASTASIS	chr14	23845069	23845069	C	T	IL25	exonic	ynonymou	172W,ENSG	161	96	257	0.373541	82	0	82	0	0.91	Sub	0.82	1.89			polymorphism	25.7	p.R172W	Passenger	SENGER/O1	damaging	Deleterious	Damaging
OVA_365_M1	METASTASIS	chr14	23876270	23876270	C	T	MYH6	exonic	ynonymou	E55K,ENSG	170	154	324	0.475309	93	0	93	0	1	Clonal	0.85	1.87			disease_causing	19.41	p.E55K	Passenger	SENGER/O1	benign	Neutral	Damaging
OVA_365_M2	METASTASIS	chr14	23876270	23876270	C	T	MYH6	exonic	ynonymou	E55K,ENSG	166	64	230	0.278261	93	0	93	0	1	Clonal	0.4	1.81			disease_causing	19.41	p.E55K	Passenger	SENGER/O1	benign	Neutral	Damaging
OVA_365_M3	METASTASIS	chr14	23876270	23876270	C	T	MYH6	exonic	ynonymou	E55K,ENSG	146	131	277	0.472924	93	0	93	0	1	Clonal	0.82	1.89			disease_causing	19.41	p.E55K	Passenger	SENGER/O1	benign	Neutral	Damaging
OVA_365_C1	Contralateral ovary	chr14	23876270	23876270	C	T	MYH6	exonic	ynonymou	E55K,ENSG	166	57	223	0.255605	93	0	93	0	1	Clonal	0.42	1.83			disease_causing	19.41	p.E55K	Passenger	SENGER/O1	benign	Neutral	Damaging
OVA_365_C2	PRIMARY	chr14	23876270	23876270	C	T	MYH6	exonic	ynonymou	E55K,ENSG	173	91	264	0.344697	93	0	93	0	1	Clonal	0.62	1.92			disease_causing	19.41	p.E55K	Passenger	SENGER/O1	benign	Neutral	Damaging
OVA_047_M1	METASTASIS	chr13	23909502	23909502	G	T	SACS	exonic	stopgain	000382292	507	147	654	0.224771	51	0	51	0	0.95	Clonal	0.47	2.01			disease_causing	41	p.S2838X	Passenger			NA	NA
OVA_047_M2	METASTASIS	chr13	23909502	23909502	G	T	SACS	exonic	stopgain	000382292	524	130	654	0.198777	51	0	51	0	0.9	Clonal	0.44	2.01			disease_causing	41	p.S2838X	Passenger			NA	NA
OVA_047_M3	METASTASIS	chr13	23909502	23909502	G	T	SACS	exonic	stopgain	000382292	307	97	404	0.240099	51	0	51	0	1	Clonal	0.6	3.02			disease_causing	41	p.S2838X	Passenger			NA	NA
OVA_048_M1	METASTASIS	chr13	23914304	23914304	T	C	SACS	exonic	onymous S	000382292:	119	269	388	0.693299	40	0	40	0	1	Clonal	0.56	3.94			disease_causing	7.046		Passenger			Neutral	Tolerated
OVA_048_M2	METASTASIS	chr13	23914304	23914304	T	C	SACS	exonic	onymous S	000382292:	248	222	470	0.47234	40	0	40	0	1	Clonal	0.4	3.87			disease_causing	7.046		Passenger			Neutral	Tolerated
OVA_048_M3	METASTASIS	chr13	23914304	23914304	T	C	SACS	exonic	onymous S	000382292:	148	214	362	0.59116	40	0	40	0	1	Clonal	0.59	3.39			disease_causing	7.046		Passenger			Neutral	Tolerated
OVA_048_C1	PRIMARY	chr13	23914304	23914304	T	C	SACS	exonic	onymous S	000382292:	82	111	193	0.57513	40	0	40	0	1	Clonal	0.67	1.82			disease_causing	7.046		Passenger			Neutral	Tolerated
OVA_048_C2	PRIMARY	chr13	23914304	23914304	T	C	SACS	exonic	onymous S	000382292:	153	294	447	0.657718	40	0	40	0	1	Clonal	0.72	1.85			disease_causing	7.046		Passenger			Neutral	Tolerated
OVA_048_C3	PRIMARY	chr13	23914304	23914304	T	C	SACS	exonic	onymous S	000382292:	90	124	214	0.579439	40	0	40	0	0.99	Clonal	0.74	1.8			disease_causing	7.046		Passenger			Neutral	Tolerated
OVA_003_M1	METASTASIS	chr22	24034613	24034613	G	A	RGL4	exonic	ynonymou	000004233	136	81	217	0.373272	39	0	39	0	0.95	Sub	0.57	1.84			polymorphism	12.33	p.A91T	Passenger	SENGER/O1	damaging	Neutral	Damaging
OVA_003_M2	METASTASIS	chr22	24034613	24034613	G	A	RGL4	exonic	ynonymou	000004233	284	89	373	0.238606	39	0	39	0	0.8	Sub	0.46	1.81			polymorphism	12.33	p.A91T	Passenger	SENGER/O1	damaging	Neutral	Damaging
OVA_003_M3	METASTASIS	chr22	24034613	24034613	G	A	RGL4	exonic	ynonymou	000004233	390	23	413	0.05569	39	0	39	0	0.42	Sub	0.34	2.67			polymorphism	12.33	p.A91T	Passenger	SENGER/O1	damaging	Neutral	Damaging
OVA_003_C1	PRIMARY	chr22	24034613	24034613	G	A	RGL4	exonic	ynonymou	000004233	36	149	185	0.805405	39	0	39	0	1	Clonal	0.86	1.81			polymorphism	12.33	p.A91T	Passenger	SENGER/O1	damaging	Neutral	Damaging
OVA_003_C2	PRIMARY	chr22	24034613	24034613	G	A	RGL4	exonic	ynonymou	000004233	66	163	229	0.71179	39	0	39	0	1	Clonal	0.7	3.61			polymorphism	12.33	p.A91T	Passenger	SENGER/O1	damaging	Neutral	Damaging
OVA_003_C3	PRIMARY	chr22	24034613	24034613	G	A	RGL4	exonic	ynonymou	000004233	80	152	232	0.655172	39	0	39	0	0.93	Clonal	0.82	1.81			polymorphism	12.33	p.A91T	Passenger	SENGER/O1	damaging	Neutral	Damaging
OVA_047_C1	PRIMARY	chr1	24119596	24119596	T	G	LYPLA2	intronic	NA	NA	329	129	458	0.281659	115	0	115	0	0.94	Sub	0.6	2.91			polymorphism	7.426						
OVA_047_C2	PRIMARY	chr1	24119596	24119596	T	G	LYPLA2	intronic	NA	NA	334	122	456	0.267544	115	0	115	0	0.84	Sub	0.64	2.76			polymorphism	7.426						
OVA_047_C3	Contralateral ovary	chr1	24119596	24119596	T	G	LYPLA2	intronic	NA	NA	307	93	400	0.2325	115	0	115	0	0.78	Sub	0.6	2.94			polymorphism	7.426						
OVA_003_M1	METASTASIS	chr16	24373042	24373042	G	A	CACNG3	exonic	ynonymou	000000528	288	97	385	0.251948	63	0	63	0	0.89	Sub	0.57	1.84			disease_causing	29.2	p.R269Q	Passenger	SENGER/O1	damaging	Deleterious	Damaging
OVA_003_M2	METASTASIS	chr16	24373042	24373042	G	A	CACNG3	exonic	ynonymou	000000528	333	77	410	0.187805	63	0	63	0	0.82	Sub	0.46	1.81			disease_causing	29.2	p.R269Q	Passenger	SENGER/O1	damaging	Deleterious	Damaging
OVA_003_M3	METASTASIS	chr16	24373042	24373042	G	A	CACNG3	exonic	ynonymou	000000528	514	34	548	0.062044	63	0	63	0	0.65	Sub	0.34	2.67			disease_causing	29.2	p.R269Q	Passenger	SENGER/O1	damaging	Deleterious	Damaging
OVA_003_C1	PRIMARY	chr16	24373042	24373042	G	A	CACNG3	exonic	ynonymou	000000528	318	235	553	0.424955	63	0	63	0	0.98	Clonal	0.86	1.81			disease_causing	29.2	p.R269Q	Passenger	SENGER/O1	damaging	Deleterious	Damaging
OVA_003_C2	PRIMARY	chr16	24373042	24373042	G	A	CACNG3	exonic	ynonymou	000000528	294	266	560	0.475	63	0	63	0	1	Clonal	0.7	3.61			disease_causing	29.2	p.R269Q	Passenger	SENGER/O1	damaging	Deleterious	Damaging
OVA_003_C3	PRIMARY	chr16	24373042	24373042	G	A	CACNG3	exonic	ynonymou	000000528	347	225	572	0.393357	63	0	63	0	0.95	Clonal	0.82	1.81			disease_causing	29.2	p.R269Q	Passenger	SENGER/O1	damaging	Deleterious	Damaging
OVA_048_M1	METASTASIS	chr5	24488297	24488297	G	A	CDH10	intronic	NA	NA	148	61	209	0.291866	8	0	8	0	1	Clonal	0.56	3.94	Yes		polymorphism	0.149						
OVA_048_M2	METASTASIS	chr5	24488297	24488297	G	A	CDH10	intronic	NA	NA	140	64	204	0.313725	8	0	8	0	1	Clonal	0.4	3.87	Yes		polymorphism	0.149						
OVA_048_M3	METASTASIS	chr5	24488297	24488297	G	A	CDH10	intronic	NA	NA	122	79	201	0.393035	8	0	8	0	1	Clonal	0.59	3.39	Yes		polymorphism	0.149						
OVA_048_C1	PRIMARY	chr5	24488297	24488297	G	A	CDH10	intronic	NA	NA	87	55	142	0.387324	8	0	8	0	1	Clonal	0.67	1.82	Yes		polymorphism	0.149						
OVA_048_C2	PRIMARY	chr5	24488297	24488297	G	A	CDH10	intronic	NA	NA	121	184	305	0.603279	8	0	8	0	1	Clonal	0.72	1.85	Yes		polymorphism	0.149						
OVA_048_C3	PRIMARY	chr5	24488297	24488297	G	A	CDH10	intronic	NA	NA	115	83	198	0.419192	8	0	8	0	1	Clonal	0.74	1.8	Yes		polymorphism	0.149						
OVA_013_C2	PRIMARY	chr14	24877298	24877298	T	A	NYNRIN	exonic	ynonymou	000038255	150	29	179	0.162011	111	0	111	0	0.53	Sub	0.87	3.05			polymorphism	25.5	p.L141Q	Passenger	SENGER/O1	damaging	Deleterious	Damaging
OVA_013_M1	METASTASIS	chr2	25141719	25141719	G	A	ADCY3	exonic	onymous S	000004351	134	126	260	0.484615	56	0	56	0	1	Clonal	0.67	3.06			disease_causing	13.34		Passenger			Neutral	Tolerated
OVA_013_C1	PRIMARY	chr2	25141719	25141719	G	A	ADCY3	exonic	onymous S	000004351	131	97	228	0.425439	56	0	56	0	1	Clonal	0.72	3.1			disease_causing	13.34		Passenger			Neutral	Tolerated
OVA_013_C2	PRIMARY	chr2	25141719	25141719	G	A	ADCY3	exonic	onymous S	000004351	107	93	200	0.465	56	0	56	0	1	Clonal	0.87	3.05			disease_causing	13.34		Passenger			Neutral	Tolerated
OVA_013_C3	PRIMARY	chr2	25141719	25141719	G	A	ADCY3	exonic	onymous S	000004351	120	88	208	0.423077	56	0	56	0	0.93	Clonal	0.91	3.01			disease_causing	13.34		Passenger			Neutral	Tolerated
OVA_048_C2	PRIMARY	chr12	25147196	25147196	A	C	C12orf77	UTR3	NA	NA	241	78	319	0.244514	22	0	22	0	0.68	Sub	0.72	1.85			polymorphism	2.395						
OVA_003_M1	METASTASIS	chr2	25362097	25362097	A	G	EFR3B	intronic	NA	NA	206	92	298	0.308725	36	0	36	0	1	Sub	0.57	1.84			polymorphism	2.487						
OVA_003_M2	METASTASIS	chr2	25362097	25362097	A	G	EFR3B	intronic	NA	NA	315	99	414	0.23913	36	0	36	0	1	Sub	0.46	1.81			polymorphism	2.487						
OVA_003_M3	METASTASIS	chr2	25362097	25362097	A	G	EFR3B	intronic	NA	NA	517	31	548	0.056569	36	0	36	0	0.6	Sub	0.34	2.67			polymorphism	2.487						
OVA_003_C1	PRIMARY	chr2	25362097	25362097	A	G	EFR3B	intronic	NA	NA	267	231	498	0.463855	36	0	36	0	1	Clonal	0.86	1.81			polymorphism	2.487						
OVA_003_C2	PRIMARY	chr2	25362097	25362097	A	G	EFR3B	intronic	NA	NA	303	215	518	0.415058	36	0	36	0	1	Clonal	0.7	3.61			polymorphism	2.487						
OVA_003_C3	PRIMARY	chr2	25362097	25362097	A	G	EFR3B	intronic	NA	NA	331	216	547	0.394881	36	0	36	0	0.96	Clonal	0.82	1.81			polymorphism	2.487						
OVA_003_M2	METASTASIS	chr10	25464553	25464568	TTGGCGCA	-	GPR158	exonic	neshift dele	000376351:	188	5	193	0.025907	72	0	72		0.11	Sub	0.46	1.81					p.I69Sfs	Passenger			NA	NA
OVA_003_C1	PRIMARY	chr10	25464553	25464568	TTGGCGCA	-	GPR158	exonic	neshift dele	000376351:	71	30	101	0.29703	72	0	72		0.69	Sub	0.86	1.81					p.I69Sfs	Passenger			NA	NA
OVA_003_C2	PRIMARY	chr10	25464553	25464568	TTGGCGCA	-	GPR158	exonic	neshift dele	000376351:	207	35	242	0.144628	72	0	72		0.7	Sub	0.7	3.61					p.I69Sfs	Passenger			NA	NA
OVA_003_C3	PRIMARY	chr10	25464553	25464568	TTGGCGCA	-	GPR158	exonic	neshift dele	000376351:	97	45	142	0.316901	72	0	72		0.77	Sub	0.82	1.81					p.I69Sfs	Passenger			NA	NA
OVA_048_M1	METASTASIS	chr1	25572949	25572949	C	G	C1orf63	exonic	ynonymou	000043184	375	237	612	0.387255	142	0	142	0	1	Clonal	0.56	3.94			polymorphism	24.5	p.R169P	Passenger	SENGER/O1	damaging	Deleterious	Damaging
OVA_048_M2	METASTASIS	chr1	25572949	25572949	C	G	C1orf63	exonic	ynonymou	000043184	426	177	603	0.293532	142	0	142	0	1	Clonal	0.4	3.87			polymorphism	24.5	p.R169P	Passenger	SENGER/O1	damaging	Deleterious	Damaging

Sample	Type	Chr	Start	End	Ref	Alt	Gene	unc.ensGen	icFunc.ens	hange.ens	t_ref	t_alt	t_total	t_VAF	n_ref	n_alt	n_total	normal_VA	ncer_cell_f	on-based C	purity	ploidy	Cancer Gene	Driver Gene	Mutation Taster	ADD(PHRE	otein_chan	CHASM	ATHMM_Pr	OLYPHEN-	REDICTION	ICTION (cutoff=0.05)
OVA_048_M3	METASTASIS	chr1	25572949	25572949	C	G	C1orf63	exonic	ynonymou	000043184	280	285	565	0.504425	142	0	142	0	1	Clonal	0.59	3.39			polymorphism	24.5	p.R169P	Passenger	SENGER/OT	damaging	Deleterious	Damaging
OVA_003_M1	METASTASIS	chr15	25947136	25947136	G	A	ATP10A	exonic	ynonymou	000356865	400	156	556	0.280576	49	0	49	0	0.99	Clonal	0.57	1.84			disease_causing	23.3	p.A896V	Passenger	SENGER/OT	damaging	Deleterious	Damaging
OVA_003_M2	METASTASIS	chr15	25947136	25947136	G	A	ATP10A	exonic	ynonymou	000356865	427	133	560	0.2375	49	0	49	0	1	Clonal	0.46	1.81			disease_causing	23.3	p.A896V	Passenger	SENGER/OT	damaging	Deleterious	Damaging
OVA_003_M3	METASTASIS	chr15	25947136	25947136	G	A	ATP10A	exonic	ynonymou	000356865	557	55	612	0.089869	49	0	49	0	0.95	Clonal	0.34	2.67			disease_causing	23.3	p.A896V	Passenger	SENGER/OT	damaging	Deleterious	Damaging
OVA_003_C1	PRIMARY	chr15	25947136	25947136	G	A	ATP10A	exonic	ynonymou	000356865	335	262	597	0.438861	49	0	49	0	1	Clonal	0.86	1.81			disease_causing	23.3	p.A896V	Passenger	SENGER/OT	damaging	Deleterious	Damaging
OVA_003_C2	PRIMARY	chr15	25947136	25947136	G	A	ATP10A	exonic	ynonymou	000356865	374	230	604	0.380795	49	0	49	0	1	Clonal	0.7	3.61			disease_causing	23.3	p.A896V	Passenger	SENGER/OT	damaging	Deleterious	Damaging
OVA_003_C3	PRIMARY	chr15	25947136	25947136	G	A	ATP10A	exonic	ynonymou	000356865	383	254	637	0.398744	49	0	49	0	0.97	Clonal	0.82	1.81			disease_causing	23.3	p.A896V	Passenger	SENGER/OT	damaging	Deleterious	Damaging
OVA_365_M1	METASTASIS	chr6	26056053	26056067	GGGCTTAC	-	HIST1H1C	exonic	ameshift de	343677:exo	167	196	363	0.539945	94	0	94		1	Clonal	0.85	1.87			polymorphism		A197_K201	Passenger			Deleterious	NA
OVA_365_M2	METASTASIS	chr6	26056053	26056067	GGGCTTAC	-	HIST1H1C	exonic	ameshift de	343677:exo	101	42	143	0.293706	94	0	94		1	Clonal	0.4	1.81			polymorphism		A197_K201	Passenger			Deleterious	NA
OVA_365_M3	METASTASIS	chr6	26056053	26056067	GGGCTTAC	-	HIST1H1C	exonic	ameshift de	343677:exo	99	119	218	0.545872	94	0	94		1	Clonal	0.82	1.89			polymorphism		A197_K201	Passenger			Deleterious	NA
OVA_365_C1	Contralateral ovary	chr6	26056053	26056067	GGGCTTAC	-	HIST1H1C	exonic	ameshift de	343677:exo	171	46	217	0.211982	94	0	94		1	Clonal	0.42	1.83			polymorphism		A197_K201	Passenger			Deleterious	NA
OVA_365_C2	PRIMARY	chr6	26056053	26056067	GGGCTTAC	-	HIST1H1C	exonic	ameshift de	343677:exo	178	75	253	0.296443	94	0	94		1	Clonal	0.62	1.92			polymorphism		A197_K201	Passenger			Deleterious	NA
OVA_013_C3	PRIMARY	chr6	26235044	26235044	G	C	HIST1H1D	exonic	ynonymou	000002445	554	98	652	0.150307	112	0	112	0	0.63	Sub	0.91	3.01			disease_causing	22.2	p.P40A	Driver	SENGER/OT	benign	Neutral	Tolerated
OVA_047_M1	METASTASIS	chr1	26349122	26349122	G	C	EXTL1	UTR5	NA	NA	194	107	301	0.355482	46	0	46	0	1	Clonal	0.47	2.01			polymorphism	10.15						
OVA_047_M2	METASTASIS	chr1	26349122	26349122	G	C	EXTL1	UTR5	NA	NA	232	117	349	0.335244	46	0	46	0	1	Clonal	0.44	2.01			polymorphism	10.15						
OVA_047_M3	METASTASIS	chr1	26349122	26349122	G	C	EXTL1	UTR5	NA	NA	88	41	129	0.317829	46	0	46	0	1	Clonal	0.6	3.02			polymorphism	10.15						
OVA_048_M1	METASTASIS	chr22	26351072	26351072	C	G	MYO18B	intronic	NA	NA	97	65	162	0.401235	15	0	15	0	1	Clonal	0.56	3.94			polymorphism	0.557						
OVA_048_M2	METASTASIS	chr22	26351072	26351072	C	G	MYO18B	intronic	NA	NA	126	74	200	0.37	15	0	15	0	1	Clonal	0.4	3.87			polymorphism	0.557						
OVA_048_M3	METASTASIS	chr22	26351072	26351072	C	G	MYO18B	intronic	NA	NA	114	98	212	0.462264	15	0	15	0	1	Clonal	0.59	3.39			polymorphism	0.557						
OVA_048_M1	METASTASIS	chr2	26534415	26534415	C	T	GPR113	exonic	nonymous S	00447444:e	361	26	387	0.067183	51	0	51	0	0.37	Sub	0.56	3.94			disease_causing	15.95		Passenger			Neutral	Tolerated
OVA_378_M1	METASTASIS	chr2	26677522	26677522	C	T	DRC1	exonic	ynonymou	000288710	452	116	568	0.204225	60	0	60	0	0.97	Clonal	0.72	2.57			polymorphism	8.544	p.R643W	Passenger	SENGER/OT	benign	Neutral	Tolerated
OVA_378_M2	METASTASIS	chr2	26677522	26677522	C	T	DRC1	exonic	ynonymou	000288710	430	119	549	0.216758	60	0	60	0	0.94	Clonal	0.85	2.57			polymorphism	8.544	p.R643W	Passenger	SENGER/OT	benign	Neutral	Tolerated
OVA_378_M3	METASTASIS	chr2	26677522	26677522	C	T	DRC1	exonic	ynonymou	000288710	470	119	589	0.202037	60	0	60	0	1	Clonal	0.67	2.57			polymorphism	8.544	p.R643W	Passenger	SENGER/OT	benign	Neutral	Tolerated
OVA_378_C1	PRIMARY	chr2	26677522	26677522	C	T	DRC1	exonic	ynonymou	000288710	487	114	601	0.189684	60	0	60	0	1	Clonal	0.59	2.6			polymorphism	8.544	p.R643W	Passenger	SENGER/OT	benign	Neutral	Tolerated
OVA_378_C2	PRIMARY	chr2	26677522	26677522	C	T	DRC1	exonic	ynonymou	000288710	427	98	525	0.186667	60	0	60	0	1	Clonal	0.56	2.59			polymorphism	8.544	p.R643W	Passenger	SENGER/OT	benign	Neutral	Tolerated
OVA_378_C3	PRIMARY	chr2	26677522	26677522	C	T	DRC1	exonic	ynonymou	000288710	442	81	523	0.154876	60	0	60	0	0.92	Clonal	0.51	2.6			polymorphism	8.544	p.R643W	Passenger	SENGER/OT	benign	Neutral	Tolerated
OVA_003_M1	METASTASIS	chr17	26684869	26684871	TCA	-	RM1;TMEM	exonic	ameshift de	60del,ENSG	160	78	238	0.327731	167	0	167		0.83	Sub	0.57	1.84			polymorphism		p.I60del	Passenger			Deleterious	NA
OVA_003_M2	METASTASIS	chr17	26684869	26684871	TCA	-	RM1;TMEM	exonic	ameshift de	60del,ENSG	291	98	389	0.251928	167	0	167		0.85	Sub	0.46	1.81			polymorphism		p.I60del	Passenger			Deleterious	NA
OVA_003_M3	METASTASIS	chr17	26684869	26684871	TCA	-	RM1;TMEM	exonic	ameshift de	60del,ENSG	707	36	743	0.048452	167	0	167		0.37	Sub	0.34	2.67			polymorphism		p.I60del	Passenger			Deleterious	NA
OVA_003_C1	PRIMARY	chr17	26684869	26684871	TCA	-	RM1;TMEM	exonic	ameshift de	60del,ENSG	55	152	207	0.7343	167	0	167		0.97	Clonal	0.86	1.81			polymorphism		p.I60del	Passenger			Deleterious	NA
OVA_003_C2	PRIMARY	chr17	26684869	26684871	TCA	-	RM1;TMEM	exonic	ameshift de	60del,ENSG	83	182	265	0.686792	167	0	167		1	Clonal	0.7	3.61			polymorphism		p.I60del	Passenger			Deleterious	NA
OVA_003_C3	PRIMARY	chr17	26684869	26684871	TCA	-	RM1;TMEM	exonic	ameshift de	60del,ENSG	91	186	277	0.67148	167	0	167		0.96	Clonal	0.82	1.81			polymorphism		p.I60del	Passenger			Deleterious	NA
OVA_003_C3	PRIMARY	chr2	26700117	26700117	C	A	OTOF	exonic	ynonymou	000339598:	430	21	451	0.046563	53	0	53	0	0.11	Sub	0.82	1.81			disease_causing	22.4	p.V816L	Passenger	SENGER/OT	benign	Neutral	Tolerated
OVA_048_M3	METASTASIS	chr17	26861690	26861690	G	T	FOXN1	intronic	NA	NA	64	19	83	0.228916	20	0	20	0	1	Clonal	0.59	3.39			polymorphism	14.23						
OVA_013_M1	METASTASIS	chr17	26919484	26919484	C	T	SPAG5	exonic	ynonymou	000032176	369	144	513	0.280702	46	0	46	0	0.84	Sub	0.67	3.06	Yes	No	polymorphism	13.88	p.V260I	Passenger	SENGER/OT	benign	Neutral	Damaging
OVA_013_C1	PRIMARY	chr17	26919484	26919484	C	T	SPAG5	exonic	ynonymou	000032176	351	238	589	0.404075	46	0	46	0	1	Sub	0.72	3.1	Yes	No	polymorphism	13.88	p.V260I	Passenger	SENGER/OT	benign	Neutral	Damaging
OVA_013_C2	PRIMARY	chr17	26919484	26919484	C	T	SPAG5	exonic	ynonymou	000032176	349	231	580	0.398276	46	0	46	0	0.92	Sub	0.87	3.05	Yes	No	polymorphism	13.88	p.V260I	Passenger	SENGER/OT	benign	Neutral	Damaging
OVA_013_C3	PRIMARY	chr17	26919484	26919484	C	T	SPAG5	exonic	ynonymou	000032176	366	240	606	0.39604	46	0	46	0	0.87	Sub	0.91	3.01	Yes	No	polymorphism	13.88	p.V260I	Passenger	SENGER/OT	benign	Neutral	Damaging
OVA_047_M2	METASTASIS	chr21	26979751	26979751	G	T	MRPL39	exonic	ynonymou	T00000352	355	52	407	0.127764	111	0	111	0	0.58	Sub	0.44	2.01			polymorphism	13.51	p.L13I	Passenger	SENGER/OT	damaging	Neutral	Damaging
OVA_048_M1	METASTASIS	chr5	26988241	26988241	G	T	CDH9	exonic	ynonymou	000005132	363	255	618	0.412621	33	0	33	0	1	Clonal	0.56	3.94			disease_causing	26	p.T67K	Passenger	SENGER/OT	damaging	Deleterious	Damaging
OVA_048_M2	METASTASIS	chr5	26988241	26988241	G	T	CDH9	exonic	ynonymou	000005132	417	227	644	0.352484	33	0	33	0	1	Clonal	0.4	3.87			disease_causing	26	p.T67K	Passenger	SENGER/OT	damaging	Deleterious	Damaging
OVA_048_M3	METASTASIS	chr5	26988241	26988241	G	T	CDH9	exonic	ynonymou	000005132	371	252	623	0.404494	33	0	33	0	1	Clonal	0.59	3.39			disease_causing	26	p.T67K	Passenger	SENGER/OT	damaging	Deleterious	Damaging
OVA_048_C1	PRIMARY	chr5	26988241	26988241	G	T	CDH9	exonic	ynonymou	000005132	304	181	485	0.373196	33	0	33	0	1	Clonal	0.67	1.82			disease_causing	26	p.T67K	Passenger	SENGER/OT	damaging	Deleterious	Damaging
OVA_048_C2	PRIMARY	chr5	26988241	26988241	G	T	CDH9	exonic	ynonymou	000005132	397	296	693	0.427128	33	0	33	0	1	Clonal	0.72	1.85			disease_causing	26	p.T67K	Passenger	SENGER/OT	damaging	Deleterious	Damaging
OVA_048_C3	PRIMARY	chr5	26988241	26988241	G	T	CDH9	exonic	ynonymou	000005132	321	202	523	0.386233	33	0	33	0	1	Clonal	0.74	1.8			disease_causing	26	p.T67K	Passenger	SENGER/OT	damaging	Deleterious	Damaging
OVA_047_C1	PRIMARY	chr17	27074874	27074874	C	T	TRAF4	exonic	ynonymou	000026239	257	123	380	0.323684	76	0	76	0	0.76	Sub	0.6	2.91			disease_causing	22.6	p.P70L	Passenger	SENGER/OT	benign	Deleterious	Tolerated
OVA_047_C2	PRIMARY	chr17	27074874	27074874	C	T	TRAF4	exonic	ynonymou	000026239	285	103	388	0.265464	76	0	76	0	0.83	Sub	0.64	2.76			disease_causing	22.6	p.P70L	Passenger	SENGER/OT	benign	Deleterious	Tolerated
OVA_047_C3	Contralateral ovary	chr17	27074874	27074874	C	T	TRAF4	exonic	ynonymou	000026239	249	80	329	0.243161	76	0	76	0	0.81	Sub	0.6	2.94			disease_causing	22.6	p.P70L	Passenger	SENGER/OT	benign	Deleterious	Tolerated
OVA_047_M1	METASTASIS	chr1	27210702	27210702	C	G	GPN2	exonic	ynonymou	S91T,ENSG	389	164	553	0.296564	36	0	36	0	0.95	Sub	0.47	2.01			disease_causing	18.25	p.S270T	Passenger	SENGER/OT	benign	Neutral	Tolerated
OVA_047_M2	METASTASIS	chr1	27210702	27210702	C	G	GPN2	exonic	ynonymou	S91T,ENSG	498	127	625	0.2032	36	0	36	0	0.71	Sub	0.44	2.01			disease_causing	18.25	p.S270T	Passenger	SENGER/OT	benign	Neutral	Tolerated
OVA_047_M3	METASTASIS	chr1	27210702	27210702	C	G	GPN2	exonic	ynonymou	S91T,ENSG	207	42	249	0.168675	36	0	36	0	0.56	Sub	0.6	3.02			disease_causing	18.25	p.S270T	Passenger	SENGER/OT	benign	Neutral	Tolerated
OVA_003_M1	METASTASIS	chr3	27216405	27216413	TTAACTGC	-	NEK10	intronic	NA	NA	499	191	690	0.276812	18	0	18		0.7	Sub	0.57	1.84										
OVA_003_M2	METASTASIS	chr3	27216405	27216413	TTAACTGC	-	NEK10	intronic	NA	NA	663	181	844	0.214455	18	0	18		0.72	Sub	0.46	1.81										
OVA_003_M3	METASTASIS	chr3	27216405	27216413	TTAACTGC	-	NEK10	intronic	NA	NA	30	828	858	0.965035	18	0	18		1	Sub	0.34	2.67										
OVA_003_C1	PRIMARY	chr3	27216405	27216413	TTAACTGC	-	NEK10	intronic	NA	NA	95	200	295	0.677966	18	0	18		0.89	Sub	0.86	1.81										
OVA_003_C2	PRIMARY	chr3	27216405	27216413	TTAACTGC	-	NEK10	intronic	NA	NA	99	186	285	0.652632	18	0	18		1	Sub	0.7	3.61										
OVA_003_C3	PRIMARY	chr3	27216405	27216413	TTAACTGC	-	NEK10	intronic	NA	NA	103	182	285	0.638596	18	0	18		0.91	Sub	0.82	1.81										
OVA_048_M2	METASTASIS	chr17	27234780	27234780	C	T	PHF12	UTR3	NA	NA	498	30	528	0.056818	55	0	55	0	0.28	Sub	0.4	3.87			polymorphism	5.738						
OVA_047_C1	PRIMARY	chr10	27335377	27335377	A	C	ANKRD26	exonic	ynonymou	00376087:	502	106	608	0.174342	30	0	30	0	0.76	Sub	0.6	2.91			polymorphism	3.101	p.N646K	Passenger	SENGER/OT	benign	Neutral	Tolerated
OVA_047_C2	PRIMARY	chr10	27335377	27335377	A	C	ANKRD26	exonic	ynonymou	00376087:	460	146	606	0.240924	30	0	30	0	0.99	Sub	0.64	2.76			polymorphism	3.101	p.N646K	Passenger	SENGER/OT	benign	Neutral	Tolerated
OVA_047_C3	Contralateral ovary	chr10	27335377	27335377	A	C	ANKRD26	exonic	ynonymou	00376087:	444	144	588	0.244898	30	0	30	0	1	Clonal	0.6	2.94			polymorphism	3.101	p.N646K	Passenger	SENGER/OT	benign	Neutral	Tolerated
OVA_047_C1	PRIMARY	chr17	27448769	27448769	T	A	MYO18A	intronic	NA	NA	97	99	196	0.505102	47	0	47	0	1	Clonal	0.6	2.91			polymorphism	0.611						
OVA_047_C2	PRIMARY	chr17	27448769	27448769	T	A	MYO18A	intronic	NA	NA	90	95	185	0.513514	47	0	47	0	1	Clonal	0.64	2.76			polymorphism	0.611						
OVA_047_C3	Contralateral ovary	chr17	27448769	27448769	T	A	MYO18A	intronic	NA	NA	81	79	160	0.49375	47	0	47	0	1	Clonal	0.6	2.94			polymorphism	0.611						
OVA_003_M1	METASTASIS	chr2	27479957	27479979	AGTGGGCT	-	SLC30A3	intronic	NA	NA	261	65	326	0.199387	29	0	29		0.71	Sub	0.57	1.84										
OVA_003_M2	METASTASIS	chr2	27479957	27479979	AGTGGGCT	-	SLC30A3	intronic	NA	NA	327	43	370	0.116216	29	0	29		0.51	Sub	0.46	1.81										
OVA_003_M3	METASTASIS	chr2	27479957	27479979	AGTGGGCT	-	SLC30A3	intronic	NA	NA	401	21	422	0.049763	29	0	29		0.52	Sub	0.34	2.67										
OVA_003_C1	PRIMARY	chr2	27479957	27479979	AGTGGGCT	-	SLC30A3	intronic	NA	NA	131	43	174	0.247126	29	0	29		0.57	Sub	0.86	1.81										
OVA_003_C2	PRIMARY	chr2	27479957	27479979	AGTGGGCT	-	SLC30A3	intronic	NA	NA	157	65	222	0.292793	29	0	29		1	Sub	0.7	3.61										
OVA_003_C3	PRIMARY	chr2	27479957	27479979	AGTGGGCT	-	SLC30A3	intronic	NA	NA	174	68	242	0.280992	29	0	29		0.68	Sub	0.82	1.81										
OVA_365_C2	PRIMARY	chr8	27779365	27779365	C	T	SCARA5	exonic	nonymous S	170V,ENSG	96	43	139	0.309353	34	0	34	0	1	Clonal	0.62	1.92			disease_causing	10.44		Passenger			Neutral	Tolerated
OVA_365_M1	METASTASIS	chr2	27844341	27844341	A	G	ZNF512	RNA_intro	NA	NA	448	190	638	0.297806	72	0	72	0	1	Clonal	0.85	1.87			polymorphism	7.91						
OVA_365_M2	METASTASIS	chr2	27844341	27844341	A	G	ZNF512	RNA_intro	NA	NA	451	87	538	0.16171	72	0	72	0	0.96	Clonal	0.4	1.81			polymorphism	7.91						
OVA_365_M3	METASTASIS	chr2	27844341	27844341	A	G	ZNF512	RNA_intro	NA	NA	454	209	663	0.315234	72	0	72	0	1	Clonal	0.82	1.89			polymorphism	7.91						
OVA_365_C1	Contralateral ovary	chr2	27844341	27844341	A	G	ZNF512	RNA_intro	NA	NA	434	106	540	0.196296	72	0	72	0	1	Clonal	0.42	1.83			polymorphism	7.91						
OVA_365_C2	PRIMARY	chr2	27844341	27844341	A	G	ZNF512	RNA_intro	NA	NA	447	142	589	0.241087	72	0	72	0	1	Clonal	0.62	1.92			polymorphism	7.91						
OVA_003_M1	METASTASIS	chr2	27930796	27930796	A	G	AK124439	exonic	nonymous S	000003796	366	138	504	0.27381	47	0	47	0	0.97	Sub	0.57	1.84				5.637					Neutral	NA
OVA_003_M2	METASTASIS	chr2	27930796	27930796	A	G	AK124439	exonic	nonymous S	000003796	391	137	528	0.25947	47	0	47	0	1	Sub	0.46	1.81				5.637					Neutral	NA
OVA_003_M3	METASTASIS	chr2	27930796	27930796	A	G	AK124439	exonic	nonymous S	000003796	461	33	494	0.066802	47	0	47	0	0.7	Sub	0.34	2.67				5.637					Neutral	NA
OVA_003_C1	PRIMARY	chr2	27930796	27930796	A	G	AK124439	exonic	nonymous S	000003796	327	218	545	0.4	47	0	47	0	0.93	Clonal	0.86	1.81				5.637					Neutral	NA
OVA_003_C2	PRIMARY	chr2	27930796	27930796	A	G	AK124439	exonic	nonymous S	000003796	338	220	558	0.394265	47	0	47	0	1	Clonal	0.7	3.61				5.637					Neutral	NA
OVA_003_C3	PRIMARY	chr2	27930796	27930796	A	G	AK124439	exonic	nonymous S	000003796	336	225	561	0.40107	47	0	47	0	0.97	Clonal	0.82	1.81				5.637					Neutral	NA
OVA_365_M1	METASTASIS	chr13	28127385	28127385	T	A	LNX2	exonic	ynonymou	000031633	312	237	549	0.431694	78	0	78	0	1	Clonal	0.85	1.87			disease_causing	25.2	p.S580C	Passenger	CANCER	damaging	Neutral	Damaging
OVA_365_M2	METASTASIS	chr13	28127385	28127385	T	A	LNX2	exonic	ynonymou	000031633	354	77	431	0.178654	78	0	78	0	0.89	Clonal	0.4	1.81			disease_causing	25.2	p.S580C	Passenger	CANCER	damaging	Neutral	Damaging
OVA_365_M3	METASTASIS	chr13	28127385	28127385	T	A	LNX2	exonic	ynonymou	000031633	329	232	561	0.413547	78	0	78	0	1	Clonal	0.82	1.89			disease_causing	25.2	p.S580C	Passenger	CANCER	damaging	Neutral	Damaging
OVA_365_C1	Contralateral ovary	chr13	28127385	28127385	T	A	LNX2	exonic	ynonymou	000031633	359	94	453	0.207506	78	0	78	0	1	Clonal	0.42	1.83			disease_causing	25.2	p.S580C	Passenger	CANCER	damaging	Neutral	Damaging
OVA_365_C2	PRIMARY	chr13	28127385	28127385	T	A	LNX2	exonic	ynonymou	000031633	355	157	512	0.306641	78	0	78	0	0.99	Clonal	0.62	1.92			disease_causing	25.2	p.S580C	Passenger	CANCER	damaging	Neutral	Damaging
OVA_047_M1	METASTASIS	chr1	28167527	28167527	C	A	PPP1R8	exonic	ynonymou	000003117	459	223	682	0.326979	83	0	83	0	1	Clonal	0.47	2.01			disease_causing	27.5	p.H92N	Passenger	SENGER/OT	damaging	Deleterious	Damaging
OVA_047_M2	METASTASIS	chr1	28167527	28167527	C	A	PPP1R8	exonic	ynonymou	000003117	508	185	693	0.266955	83	0	83	0	0.94	Clonal	0.44	2.01			disease_causing	27.5	p.H92N	Passenger	SENGER/OT	damaging	Deleterious	Damaging
OVA_047_M3	METASTASIS	chr1	28167527	28167527	C	A	PPP1R8	exonic	ynonymou	000003117	313	137	450	0.304444	83	0	83	0	1	Clonal	0.6	3.02			disease_causing	27.5	p.H92N	Passenger	SENGER/OT	damaging	Deleterious	Damaging
OVA_013_M1	METASTASIS	chr21	28304486	28304486	C	G	ADAMTS5	exonic	ynonymou	000028498	164	329	493	0.667343	45	0	45	0	1	Clonal	0.67	3.06			disease_causing	33	p.R629P	Passenger	SENGER/OT	damaging	Deleterious	Damaging
OVA_013_C1	PRIMARY	chr21	28304486	28304486	C	G	ADAMTS5	exonic	ynonymou	000028498	320	274	594	0.461279	45	0	45	0	1	Clonal	0.72	3.1			disease_causing	33	p.R629P	Passenger	SENGER/OT	damaging	Deleterious	Damaging
OVA_013_C2	PRIMARY	chr21	28304486	28304486	C	G	ADAMTS5	exonic	ynonymou	000028498	339	229	568	0.403169	45	0	45	0	1	Clonal	0.87	3.05			disease_causing	33	p.R629P	Passenger	SENGER/OT	damaging	Deleterious	Damaging
OVA_013_C3	PRIMARY	chr21	28304486	28304486	C	G	ADAMTS5	exonic	ynonymou	000028498	109	453	562	0.80605	45	0	45	0	1	Clonal	0.91	3.01			disease_causing	33	p.R629P	Passenger	SENGER/OT	damaging	Deleterious	Damaging
OVA_047_C1	PRIMARY	chr6	29408070	29408070	G	A	OR10C1	exonic	ynonymou	000004441	550	36	586	0.061433	77	0	77	0	0.33	Sub	0.6	2.91			polymorphism	20.9	p.R93H	Passenger		damaging	Neutral	Damaging
OVA_047_C2	PRIMARY	chr6	29408070	29408070	G	A	OR10C1	exonic	ynonymou	000004441	500	95	595	0.159664	77	0	77	0	0.82	Sub	0.64	2.76			polymorphism	20.9	p.R93H	Passenger		damaging	Neutral	Damaging
OVA_047_C3	Contralateral ovary	chr6	29408070	29408070	G	A	OR10C1	exonic	ynonymou	000004441	491	103	594	0.173401	77	0	77	0	1	Clonal	0.6	2.94			polymorphism	20.9	p.R93H	Passenger		damaging	Neutral	Damaging
OVA_013_M1	METASTASIS	chr17	29557401	29557401	G	T	NF1	splicing	NA	NA	181	402	583	0.689537	50	1	51	0.019608	1	Clonal	0.67	3.06	Yes		disease_causing_automat	33		Passenger				
OVA_013_C1	PRIMARY	chr17	29557401	29557401	G	T	NF1	splicing	NA	NA	115	454	569	0.797891	50	1	51	0.019608	1	Clonal	0.72	3.1	Yes		disease_causing_automat	33		Passenger				
OVA_013_C2	PRIMARY	chr17	29557401	29557401	G	T	NF1	splicing	NA	NA	102	441	543	0.812155	50	1	51	0.019608	1	Clonal	0.87	3.05	Yes		disease_causing_automat	33		Passenger				

Sample	Type	Chr	Start	End	Ref	Alt	Gene	unc.ensGen	icFunc.ens	hange.ens	t_ref	t_alt	t_total	t_VAF	n_ref	n_alt	n_total	ormal_VA	ncer_cell_f	on-based C	purity	ploidy	Cancer Gene	Driver Gene	Mutation Taster	ADD(PHRE	otein_chan	CHASM	THMM_Pr	OLYPHEN-	REDICTION	ICTION (cutoff=0.05)
OVA_013_C3	PRIMARY	chr17	29557401	29557401	G	T	NF1	splicing	NA	NA	72	491	563	0.872114	50	1	51	0.019608	1	Clonal	0.91	3.01	Yes		disease_causing_automat	33		Passenger				
OVA_003_M1	METASTASIS	chr13	29855850	29855850	T	A	MTUS2	exonic	ynonymou	000043153	202	12	214	0.056075	39	0	39	0	0.14	Sub	0.57	1.84			polymorphism	21.7	p.F895Y	Passenger		damaging	Neutral	Damaging
OVA_003_M2	METASTASIS	chr13	29855850	29855850	T	A	MTUS2	exonic	ynonymou	000043153	258	10	268	0.037313	39	0	39	0	0.13	Sub	0.46	1.81			polymorphism	21.7	p.F895Y	Passenger		damaging	Neutral	Damaging
OVA_003_M3	METASTASIS	chr13	29855850	29855850	T	A	MTUS2	exonic	ynonymou	000043153	354	7	361	0.019391	39	0	39	0	0.17	Sub	0.34	2.67			polymorphism	21.7	p.F895Y	Passenger		damaging	Neutral	Damaging
OVA_003_C1	PRIMARY	chr13	29855850	29855850	T	A	MTUS2	exonic	ynonymou	000043153	182	153	335	0.456716	39	0	39	0	1	Sub	0.86	1.81			polymorphism	21.7	p.F895Y	Passenger		damaging	Neutral	Damaging
OVA_003_C2	PRIMARY	chr13	29855850	29855850	T	A	MTUS2	exonic	ynonymou	000043153	249	126	375	0.336	39	0	39	0	1	Sub	0.7	3.61			polymorphism	21.7	p.F895Y	Passenger		damaging	Neutral	Damaging
OVA_003_C3	PRIMARY	chr13	29855850	29855850	T	A	MTUS2	exonic	ynonymou	000043153	241	125	366	0.34153	39	0	39	0	0.83	Sub	0.82	1.81			polymorphism	21.7	p.F895Y	Passenger		damaging	Neutral	Damaging
OVA_003_M1	METASTASIS	chr20	30193735	30193735	G	T	ID1	UTR3	NA	NA	253	102	355	0.287324	141	0	141	0	1	Sub	0.57	1.84			polymorphism	7.346						
OVA_003_M2	METASTASIS	chr20	30193735	30193735	G	T	ID1	UTR3	NA	NA	318	93	411	0.226277	141	0	141	0	0.99	Sub	0.46	1.81			polymorphism	7.346						
OVA_003_M3	METASTASIS	chr20	30193735	30193735	G	T	ID1	UTR3	NA	NA	408	30	438	0.068493	141	0	141	0	0.72	Sub	0.34	2.67			polymorphism	7.346						
OVA_003_C1	PRIMARY	chr20	30193735	30193735	G	T	ID1	UTR3	NA	NA	264	223	487	0.457906	141	0	141	0	1	Clonal	0.86	1.81			polymorphism	7.346						
OVA_003_C2	PRIMARY	chr20	30193735	30193735	G	T	ID1	UTR3	NA	NA	296	215	511	0.420744	141	0	141	0	1	Clonal	0.7	3.61			polymorphism	7.346						
OVA_003_C3	PRIMARY	chr20	30193735	30193735	G	T	ID1	UTR3	NA	NA	323	209	532	0.392857	141	0	141	0	0.95	Clonal	0.82	1.81			polymorphism	7.346						
OVA_013_M1	METASTASIS	chr20	30450392	30450392	G	T	DUSP15	exonic	onymous S	S139G,ENS	150	78	228	0.342105	26	0	26	0	1	Clonal	0.67	3.06			disease_causing	12.14		Passenger			Neutral	Tolerated
OVA_013_C1	PRIMARY	chr20	30450392	30450392	G	T	DUSP15	exonic	onymous S	S139G,ENS	250	105	355	0.295775	26	0	26	0	1	Clonal	0.72	3.1			disease_causing	12.14		Passenger			Neutral	Tolerated
OVA_013_C2	PRIMARY	chr20	30450392	30450392	G	T	DUSP15	exonic	onymous S	S139G,ENS	217	100	317	0.315457	26	0	26	0	1	Clonal	0.87	3.05			disease_causing	12.14		Passenger			Neutral	Tolerated
OVA_013_C3	PRIMARY	chr20	30450392	30450392	G	T	DUSP15	exonic	onymous S	S139G,ENS	267	147	414	0.355072	26	0	26	0	1	Clonal	0.91	3.01			disease_causing	12.14		Passenger			Neutral	Tolerated
OVA_048_M1	METASTASIS	chr16	30528968	30528968	A	T	ITGAL	splicing	NA	NA	80	11	91	0.120879	25	0	25	0	0.43	Sub	0.56	3.94			disease_causing	24.8		Passenger				
OVA_047_C1	PRIMARY	chr16	30582912	30582912	G	T	ZNF688	exonic	onymous S	T000005637	7	6	13	0.461538	36	0	36	0	1	Sub	0.6	2.91			polymorphism	5.279		Passenger			Neutral	Tolerated
OVA_047_C2	PRIMARY	chr16	30582912	30582912	G	T	ZNF688	exonic	onymous S	T000005637	14	3	17	0.176471	36	0	36	0	0.55	Sub	0.64	2.76			polymorphism	5.279		Passenger			Neutral	Tolerated
OVA_047_C3	Contralateral ovary	chr16	30582912	30582912	G	T	ZNF688	exonic	onymous S	T000005637	4	4	8	0.5	36	0	36	0	1	Clonal	0.6	2.94			polymorphism	5.279		Passenger			Neutral	Tolerated
OVA_047_C1	PRIMARY	chr6	30860073	30860073	G	A	DDR1	exonic	ynonymou	I,ENSG000	322	60	382	0.157068	73	0	73	0	0.84	Sub	0.6	2.91			disease_causing	22.9	p.V285I	Passenger	SENGER/OT	benign	Neutral	Tolerated
OVA_047_C2	PRIMARY	chr6	30860073	30860073	G	A	DDR1	exonic	ynonymou	I,ENSG000	402	71	473	0.150106	73	0	73	0	0.77	Sub	0.64	2.76			disease_causing	22.9	p.V285I	Passenger	SENGER/OT	benign	Neutral	Tolerated
OVA_047_C3	Contralateral ovary	chr6	30860073	30860073	G	A	DDR1	exonic	ynonymou	I,ENSG000	339	47	386	0.121762	73	0	73	0	0.77	Sub	0.6	2.94			disease_causing	22.9	p.V285I	Passenger	SENGER/OT	benign	Neutral	Tolerated
OVA_047_M1	METASTASIS	chr16	31092739	31092739	C	A	ZNF646	exonic	onymous S	.698A,ENSG	383	126	509	0.247544	63	0	63	0	1	Clonal	0.47	2.01			disease_causing	16.58		Passenger			Neutral	Tolerated
OVA_047_M2	METASTASIS	chr16	31092739	31092739	C	A	ZNF646	exonic	onymous S	.698A,ENSG	467	120	587	0.204429	63	0	63	0	0.92	Clonal	0.44	2.01			disease_causing	16.58		Passenger			Neutral	Tolerated
OVA_047_M3	METASTASIS	chr16	31092739	31092739	C	A	ZNF646	exonic	onymous S	.698A,ENSG	228	79	307	0.257329	63	0	63	0	1	Clonal	0.6	3.02			disease_causing	16.58		Passenger			Neutral	Tolerated
OVA_013_M1	METASTASIS	chr16	31154828	31154828	G	A	PRSS36	intronic	NA	NA	208	48	256	0.1875	105	0	105	0	0.75	Sub	0.67	3.06			polymorphism	4.683						
OVA_013_C1	PRIMARY	chr16	31154828	31154828	G	A	PRSS36	intronic	NA	NA	242	79	321	0.246106	105	0	105	0	0.93	Sub	0.72	3.1			polymorphism	4.683						
OVA_013_C2	PRIMARY	chr16	31154828	31154828	G	A	PRSS36	intronic	NA	NA	234	67	301	0.222591	105	0	105	0	0.73	Sub	0.87	3.05			polymorphism	4.683						
OVA_013_C3	PRIMARY	chr16	31154828	31154828	G	A	PRSS36	intronic	NA	NA	210	107	317	0.337539	105	0	105	0	1	Sub	0.91	3.01			polymorphism	4.683						
OVA_378_M1	METASTASIS	chr15	31283779	31283779	C	T	MTMR10	UTR5	NA	NA	15	2	17	0.117647	55	0	55	0	0.33	Sub	0.85	2.57			polymorphism	9.444						
OVA_378_M2	METASTASIS	chr15	31283779	31283779	C	T	MTMR10	UTR5	NA	NA	8	6	14	0.428571	55	0	55	0	1	Sub	0.85	2.57			polymorphism	9.444						
OVA_378_M3	METASTASIS	chr15	31283779	31283779	C	T	MTMR10	UTR5	NA	NA	17	4	21	0.190476	55	0	55	0	0.57	Sub	0.67	2.57			polymorphism	9.444						
OVA_378_C1	PRIMARY	chr15	31283779	31283779	C	T	MTMR10	UTR5	NA	NA	15	4	19	0.210526	55	0	55	0	0.72	Sub	0.59	2.6			polymorphism	9.444						
OVA_378_C2	PRIMARY	chr15	31283779	31283779	C	T	MTMR10	UTR5	NA	NA	21	2	23	0.086957	55	0	55	0	0.31	Sub	0.59	2.6			polymorphism	9.444						
OVA_378_C3	PRIMARY	chr15	31283779	31283779	C	T	MTMR10	UTR5	NA	NA	9	4	13	0.307692	55	0	55	0	1	Sub	0.51	2.6			polymorphism	9.444						
OVA_047_C1	PRIMARY	chr2	31348085	31348085	C	G	GALNT14	exonic	ynonymou	000003245	326	111	437	0.254005	36	0	36	0	0.85	Sub	0.6	2.91			polymorphism	0.247	p.S55T	Passenger	SENGER/OT	benign	Neutral	Tolerated
OVA_047_C2	PRIMARY	chr2	31348085	31348085	C	G	GALNT14	exonic	ynonymou	000003245	416	107	523	0.204589	36	0	36	0	0.84	Sub	0.64	2.76			polymorphism	0.247	p.S55T	Passenger	SENGER/OT	benign	Neutral	Tolerated
OVA_047_C3	Contralateral ovary	chr2	31348085	31348085	C	G	GALNT14	exonic	ynonymou	000003245	381	83	464	0.178879	36	0	36	0	0.78	Sub	0.6	2.94			polymorphism	0.247	p.S55T	Passenger	SENGER/OT	benign	Neutral	Tolerated
OVA_048_M1	METASTASIS	chr2	31417292	31417292	G	T	CAPN14	exonic	ynonymou	D311E,ENSG	226	123	349	0.352436	35	0	35	0	1	Clonal	0.56	3.94			polymorphism	0.242	p.D311E	Passenger	SENGER/OT	benign	Neutral	Tolerated
OVA_048_M2	METASTASIS	chr2	31417292	31417292	G	T	CAPN14	exonic	ynonymou	D311E,ENSG	291	87	378	0.230159	35	0	35	0	1	Clonal	0.4	3.87			polymorphism	0.242	p.D311E	Passenger	SENGER/OT	benign	Neutral	Tolerated
OVA_048_M3	METASTASIS	chr2	31417292	31417292	G	T	CAPN14	exonic	ynonymou	D311E,ENSG	171	140	311	0.450161	35	0	35	0	1	Clonal	0.59	3.39			polymorphism	0.242	p.D311E	Passenger	SENGER/OT	benign	Neutral	Tolerated
OVA_378_M1	METASTASIS	chr2	31425111	31425111	G	A	CAPN14	exonic	onymous S	000044491	564	12	576	0.020833	24	0	24	0	0.1	Sub	0.72	2.57			disease_causing	11.6		Passenger			Neutral	Tolerated
OVA_378_M2	METASTASIS	chr2	31425111	31425111	G	A	CAPN14	exonic	onymous S	000044491	537	39	576	0.067708	24	0	24	0	0.29	Sub	0.85	2.57			disease_causing	11.6		Passenger			Neutral	Tolerated
OVA_378_M3	METASTASIS	chr2	31425111	31425111	G	A	CAPN14	exonic	onymous S	000044491	600	40	640	0.0625	24	0	24	0	0.31	Sub	0.67	2.57			disease_causing	11.6		Passenger			Neutral	Tolerated
OVA_047_C1	PRIMARY	chr6	31496908	31496908	C	A	MCCD1	exonic	ynonymou	000003761	489	87	576	0.151042	39	0	39	0	0.81	Sub	0.6	2.91			polymorphism	13.44	p.S39R	Passenger	SENGER/OT	damaging	Neutral	Damaging
OVA_047_C2	PRIMARY	chr6	31496908	31496908	C	A	MCCD1	exonic	ynonymou	000003761	517	91	608	0.149671	39	0	39	0	0.77	Sub	0.64	2.76			polymorphism	13.44	p.S39R	Passenger	SENGER/OT	damaging	Neutral	Damaging
OVA_047_C3	Contralateral ovary	chr6	31496908	31496908	C	A	MCCD1	exonic	ynonymou	000003761	486	87	573	0.151832	39	0	39	0	0.96	Clonal	0.6	2.94			polymorphism	13.44	p.S39R	Passenger	SENGER/OT	damaging	Neutral	Damaging
OVA_048_M1	METASTASIS	chr21	31588105	31588105	A	G	CLDN8	exonic	ynonymou	W47R,ENSG	382	233	615	0.378862	35	0	35	0	1	Clonal	0.56	3.94			disease_causing	25.8	p.W47R	Passenger	SENGER/OT	damaging	Deleterious	Damaging
OVA_048_M2	METASTASIS	chr21	31588105	31588105	A	G	CLDN8	exonic	ynonymou	W47R,ENSG	424	177	601	0.294509	35	0	35	0	1	Clonal	0.4	3.87			disease_causing	25.8	p.W47R	Passenger	SENGER/OT	damaging	Deleterious	Damaging
OVA_048_M3	METASTASIS	chr21	31588105	31588105	A	G	CLDN8	exonic	ynonymou	W47R,ENSG	396	211	607	0.347611	35	0	35	0	1	Clonal	0.59	3.39			disease_causing	25.8	p.W47R	Passenger	SENGER/OT	damaging	Deleterious	Damaging
OVA_048_C1	PRIMARY	chr21	31588105	31588105	A	G	CLDN8	exonic	ynonymou	W47R,ENSG	117	173	290	0.596552	35	0	35	0	1	Clonal	0.67	1.82			disease_causing	25.8	p.W47R	Passenger	SENGER/OT	damaging	Deleterious	Damaging
OVA_048_C2	PRIMARY	chr21	31588105	31588105	A	G	CLDN8	exonic	ynonymou	W47R,ENSG	175	312	487	0.640657	35	0	35	0	1	Clonal	0.72	1.85			disease_causing	25.8	p.W47R	Passenger	SENGER/OT	damaging	Deleterious	Damaging
OVA_048_C3	PRIMARY	chr21	31588105	31588105	A	G	CLDN8	exonic	ynonymou	W47R,ENSG	143	174	317	0.548896	35	0	35	0	0.93	Clonal	0.74	1.8			disease_causing	25.8	p.W47R	Passenger	SENGER/OT	damaging	Deleterious	Damaging
OVA_048_M1	METASTASIS	chr14	31614057	31614057	C	A	HECTD1	exonic	ynonymou	863S,ENSG	386	236	622	0.379421	55	0	55	0	1	Clonal	0.56	3.94			disease_causing	22.4	p.A863S	Passenger		benign	Neutral	Tolerated
OVA_048_M2	METASTASIS	chr14	31614057	31614057	C	A	HECTD1	exonic	ynonymou	863S,ENSG	535	87	622	0.139871	55	0	55	0	0.98	Clonal	0.4	3.87			disease_causing	22.4	p.A863S	Passenger		benign	Neutral	Tolerated
OVA_013_M1	METASTASIS	chr21	31655145	31655145	A	T	KRTAP24-1	exonic	ynonymou	000003403	347	145	492	0.294715	45	0	45	0	0.88	Clonal	0.67	3.06			polymorphism	17.42	p.S36T	Passenger	SENGER/OT	damaging	Neutral	Tolerated
OVA_013_C1	PRIMARY	chr21	31655145	31655145	A	T	KRTAP24-1	exonic	ynonymou	000003403	443	155	598	0.259197	45	0	45	0	0.98	Sub	0.72	3.1			polymorphism	17.42	p.S36T	Passenger	SENGER/OT	damaging	Neutral	Tolerated
OVA_013_C2	PRIMARY	chr21	31655145	31655145	A	T	KRTAP24-1	exonic	ynonymou	000003403	477	128	605	0.21157	45	0	45	0	0.7	Sub	0.87	3.05			polymorphism	17.42	p.S36T	Passenger	SENGER/OT	damaging	Neutral	Tolerated
OVA_013_C3	PRIMARY	chr21	31655145	31655145	A	T	KRTAP24-1	exonic	ynonymou	000003403	345	247	592	0.41723	45	0	45	0	0.91	Sub	0.91	3.01			polymorphism	17.42	p.S36T	Passenger	SENGER/OT	damaging	Neutral	Tolerated
OVA_047_C1	PRIMARY	chr6	32064031	32064045	GCAGTCCC	-	TNXB	exonic	ameshift de	75247:exon	432	157	589	0.266553	81	0	81		1	Clonal	0.6	2.91			polymorphism		P529_R533	Passenger			Deleterious	NA
OVA_047_C2	PRIMARY	chr6	32064031	32064045	GCAGTCCC	-	TNXB	exonic	ameshift de	75247:exon	469	118	587	0.201022	81	0	81		1	Clonal	0.64	2.76			polymorphism		P529_R533	Passenger			Deleterious	NA
OVA_047_C3	Contralateral ovary	chr6	32064031	32064045	GCAGTCCC	-	TNXB	exonic	ameshift de	75247:exon	473	126	599	0.210351	81	0	81		1	Clonal	0.6	2.94			polymorphism		P529_R533	Passenger			Deleterious	NA
OVA_365_C1	Contralateral ovary	chr6	32064774	32064774	G	A	TNXB	exonic	stopgain	000037524	328	66	394	0.167513	83	0	83	0	1	Clonal	0.42	1.83			disease_causing_automat	35	p.Q286X	Passenger			NA	NA
OVA_365_C1	Contralateral ovary	chr6	32096020	32096020	C	G	ATF6B;FKBP	UTR5	NA	NA	53	18	71	0.253521	67	0	67	0	1	Clonal	0.42	1.83			polymorphism	17.77						
OVA_365_M3	METASTASIS	chr6	32150709	32150709	T	A	AGER	exonic	onymous S	186G,ENSG	363	32	395	0.081013	96	0	96	0	0.36	Sub	0.82	1.89			polymorphism	5.457		Passenger			Neutral	Tolerated
OVA_047_C2	PRIMARY	chr6	32188176	32188176	C	A	NOTCH4	intronic	NA	NA	425	51	476	0.107143	55	0	55	0	0.55	Sub	0.64	2.76	Yes		polymorphism	4.327						
OVA_003_M1	METASTASIS	chr9	32485231	32485231	G	A	DDX58	exonic	onymous S	000545044	363	162	525	0.308571	47	0	47	0	1	Sub	0.57	1.84			polymorphism	2.187		Passenger			Neutral	Tolerated
OVA_003_M2	METASTASIS	chr9	32485231	32485231	G	A	DDX58	exonic	onymous S	000545044	404	130	534	0.243446	47	0	47	0	1	Sub	0.46	1.81			polymorphism	2.187		Passenger			Neutral	Tolerated
OVA_003_M3	METASTASIS	chr9	32485231	32485231	G	A	DDX58	exonic	onymous S	000545044	516	34	550	0.061818	47	0	47	0	0.65	Sub	0.34	2.67			polymorphism	2.187		Passenger			Neutral	Tolerated
OVA_003_C1	PRIMARY	chr9	32485231	32485231	G	A	DDX58	exonic	onymous S	000545044	324	215	539	0.398887	47	0	47	0	0.92	Clonal	0.86	1.81			polymorphism	2.187		Passenger			Neutral	Tolerated
OVA_003_C2	PRIMARY	chr9	32485231	32485231	G	A	DDX58	exonic	onymous S	000545044	340	202	542	0.372694	47	0	47	0	1	Clonal	0.7	3.61			polymorphism	2.187		Passenger			Neutral	Tolerated
OVA_003_C3	PRIMARY	chr9	32485231	32485231	G	A	DDX58	exonic	onymous S	000545044	321	241	562	0.428826	47	0	47	0	1	Clonal	0.82	1.81			polymorphism	2.187		Passenger			Neutral	Tolerated
OVA_047_M1	METASTASIS	chr1	32568255	32568255	T	G	TMEM39B	exonic	ynonymou	288R,ENSG	475	109	584	0.186644	65	0	65	0	0.97	Clonal	0.47	2.01			disease_causing	23.7	p.L487R	Passenger	no weights	benign	Neutral	Damaging
OVA_047_M2	METASTASIS	chr1	32568255	32568255	T	G	TMEM39B	exonic	ynonymou	288R,ENSG	456	119	575	0.206957	65	0	65	0	1	Clonal	0.44	2.01			disease_causing	23.7	p.L487R	Passenger	no weights	benign	Neutral	Damaging
OVA_047_M3	METASTASIS	chr1	32568255	32568255	T	G	TMEM39B	exonic	ynonymou	288R,ENSG	195	47	242	0.194215	65	0	65	0	1	Clonal	0.6	3.02			disease_causing	23.7	p.L487R	Passenger	no weights	benign	Neutral	Damaging
OVA_365_M1	METASTASIS	chr6	32939923	32939923	G	T	BRD2	cRNA_exon	NA	NA	140	36	176	0.204545	152	0	152	0	0.89	Clonal	0.85	1.87			disease_causing	22						
OVA_365_M2	METASTASIS	chr6	32939923	32939923	G	T	BRD2	cRNA_exon	NA	NA	66	8	74	0.108108	152	0	152	0	0.75	Clonal	0.4	1.87			disease_causing	22						
OVA_365_M3	METASTASIS	chr6	32939923	32939923	G	T	BRD2	cRNA_exon	NA	NA	91	28	119	0.235294	152	0	152	0	1	Clonal	0.82	1.89			disease_causing	22						
OVA_365_C1	Contralateral ovary	chr6	32939923	32939923	G	T	BRD2	cRNA_exon	NA	NA	66	14	80	0.175	152	0	152	0	1	Clonal	0.42	1.83			disease_causing	22						
OVA_365_C2	PRIMARY	chr6	32939923	32939923	G	T	BRD2	cRNA_exon	NA	NA	125	48	173	0.277457	152	0	152	0	1	Clonal	0.62	1.92			disease_causing	22						
OVA_048_M1	METASTASIS	chr12	32945435	32945435	T	C	PKP2	intronic	NA	NA	340	220	560	0.392857	30	0	30	0	1	Clonal	0.56	3.94			polymorphism	15.87						
OVA_048_M2	METASTASIS	chr12	32945435	32945435	T	C	PKP2	intronic	NA	NA	382	165	547	0.301645	30	0	30	0	1	Clonal	0.4	3.87			polymorphism	15.87						
OVA_048_M3	METASTASIS	chr12	32945435	32945435	T	C	PKP2	intronic	NA	NA	407	125	532	0.234962	30	0	30	0	1	Clonal	0.59	3.39			polymorphism	15.87						
OVA_048_C1	PRIMARY	chr12	32945435	32945435	T	C	PKP2	intronic	NA	NA	193	124	317	0.391167	30	0	30	0	1	Clonal	0.67	1.82			polymorphism	15.87						
OVA_048_C2	PRIMARY	chr12	32945435	32945435	T	C	PKP2	intronic	NA	NA	369	194	563	0.344583	30	0	30	0	0.96	Clonal	0.72	1.85			polymorphism	15.87						
OVA_048_C3	PRIMARY	chr12	32945435	32945435	T	C	PKP2	intronic	NA	NA	234	144	378	0.380952	30	0	30	0	1	Clonal	0.74	1.8			polymorphism	15.87						
OVA_047_M1	METASTASIS	chr11	33054552	33054552	C	A	DEPDC7	intronic	NA	NA	499	108	607	0.177924	15	0	15	0	0.75	Sub	0.47	2.01			polymorphism	2.817						
OVA_047_M2	METASTASIS	chr11	33054552	33054552	C	A	DEPDC7	intronic	NA	NA	500	97	597	0.162479	15	0	15	0	0.73	Sub	0.44	2.01			polymorphism	2.817						
OVA_047_M3	METASTASIS	chr11	33054552	33054552	C	A	DEPDC7	intronic	NA	NA	193	56	249	0.2249	15	0	15	0	0.97	Sub	0.6	3.02			polymorphism	2.817						
OVA_047_C1	PRIMARY	chr3	33114202	33114202	C	T	GLB1	exonic	ynonymou	A27T,ENSG	479	165	644	0.256211	39	0	39	0	1	Sub	0.6	2.91			polymorphism	12.9	p.A27T	Passenger	SENGER/OT	benign	Neutral	Tolerated
OVA_047_C3	Contralateral ovary	chr3	33114202	33114202	C	T	GLB1	exonic	ynonymou	A27T,ENSG	574	86	660	0.130303	39	0	39	0	0.57	Sub	0.6	2.94			polymorphism	12.9	p.A27T	Passenger	SENGER/OT	benign	Neutral	Tolerated
OVA_003_M1	METASTASIS	chr6	33134886	33134886	C	A	COL11A2	exonic	onymous S	L350L,ENSG	82	26	108	0.240741	18	0	18	0	0.85	Clonal	0.57	1.84			disease_causing	8.624		Passenger			Neutral	Tolerated
OVA_003_M2	METASTASIS	chr6	33134886	33134886	C	A	COL11A2	exonic	onymous S	L350L,ENSG	109	26	135	0.192593	18	0	18	0	0.84	Clonal	0.46	1.81			disease_causing	8.624		Passenger			Neutral	Tolerated
OVA_003_C1	PRIMARY	chr6	33134886	33134886	C	A	COL11A2	exonic	onymous S	L350L,ENSG	108	70	178	0.393258	18	0	18	0	0.91	Clonal	0.86	1.81			disease_causing	8.624		Passenger			Neutral	Tolerated
OVA_003_C2	PRIMARY	chr6	33134886	33134886	C	A	COL11A2	exonic	onymous S	L350L,ENSG	129	79	208	0.379808	18	0	18	0	1	Clonal	0.7	3.61			disease_causing	8.624		Passenger			Neutral	Tolerated
OVA_003_C3	PRIMARY	chr6	33134886	33134886	C	A	COL11A2	exonic	onymous S	L350L,ENSG	143	88	231	0.380952	18	0	18	0	0.92	Clonal	0.82	1.81			disease_causing	8.624		Passenger			Neutral	Tolerated
OVA_003_C1	PRIMARY	chr6	33138158	33138158	T	A	COL11A2	splicing	NA	NA	320	24	344	0.069767	39	0	39	0	0.16	Sub	0.86	1.81			disease_causing	28.2		Passenger				
OVA_003_C2	PRIMARY	chr6	33138158	33138158	T	A	COL11A2	splicing	NA	NA	353	31	384	0.080729	39	0	39	0	0.31	Sub	0.7	3.61			disease_causing	28.2		Passenger				

Sample	Type	Chr	Start	End	Ref	Alt	Gene	unc.ensGen	icFunc.ens	hange.ens	t_ref	t_alt	t_total	t_VAF	n_ref	n_alt	n_total	normal_VA	ncer_cell_f	on-based C	purity	ploidy	Cancer Gene	Driver Gene	Mutation Taster	ADD(PHRE	otein_chan	CHASM	THMM_Pr	OLYPHEN-	REDICTION	ICTION (cutoff=0.05)
OVA_048_M1	METASTASIS	chr1	33231446	33231446	G	T	KIAA1522	intronic	NA	NA	277	27	304	0.088816	67	0	67	0	0.49	Sub	0.56	3.94			polymorphism	8.81						
OVA_365_M2	METASTASIS	chr5	33637773	33637773	C	A	ADAMTS12	exonic	ynonymous	599D,ENSG	371	79	450	0.175556	60	0	60	0	0.87	Clonal	0.4	1.81			polymorphism	0.773	p.E599D	Passenger	SENGER/OT	benign	Neutral	Tolerated
OVA_003_M1	METASTASIS	chr19	33647242	33647242	C	A	WDR88	intronic	NA	NA	384	157	541	0.290203	22	0	22	0	1	Sub	0.57	1.84			polymorphism	0.946						
OVA_003_M2	METASTASIS	chr19	33647242	33647242	C	A	WDR88	intronic	NA	NA	415	139	554	0.250903	22	0	22	0	1	Sub	0.46	1.81			polymorphism	0.946						
OVA_003_M3	METASTASIS	chr19	33647242	33647242	C	A	WDR88	intronic	NA	NA	554	36	590	0.061017	22	0	22	0	0.64	Sub	0.34	2.67			polymorphism	0.946						
OVA_003_C1	PRIMARY	chr19	33647242	33647242	C	A	WDR88	intronic	NA	NA	337	251	588	0.426871	22	0	22	0	0.99	Clonal	0.86	1.81			polymorphism	0.946						
OVA_003_C2	PRIMARY	chr19	33647242	33647242	C	A	WDR88	intronic	NA	NA	369	208	577	0.360485	22	0	22	0	1	Clonal	0.7	3.61			polymorphism	0.946						
OVA_003_C3	PRIMARY	chr19	33647242	33647242	C	A	WDR88	intronic	NA	NA	383	240	623	0.385233	22	0	22	0	0.94	Clonal	0.82	1.81			polymorphism	0.946						
OVA_003_M1	METASTASIS	chr18	33793341	33793341	G	C	MOCOS	exonic	ynonymous	0000261320	175	112	287	0.390244	37	0	37	0	0.99	Sub	0.57	1.84			disease_causing	24.3	p.A411P	Passenger	SENGER/OT	damaging	Deleterious	Damaging
OVA_003_M2	METASTASIS	chr18	33793341	33793341	G	C	MOCOS	exonic	ynonymous	0000261320	248	112	360	0.311111	37	0	37	0	1	Sub	0.46	1.81			disease_causing	24.3	p.A411P	Passenger	SENGER/OT	damaging	Deleterious	Damaging
OVA_003_M3	METASTASIS	chr18	33793341	33793341	G	C	MOCOS	exonic	ynonymous	0000261320	393	35	428	0.081776	37	0	37	0	0.62	Sub	0.34	2.67			disease_causing	24.3	p.A411P	Passenger	SENGER/OT	damaging	Deleterious	Damaging
OVA_003_C1	PRIMARY	chr18	33793341	33793341	G	C	MOCOS	exonic	ynonymous	0000261320	50	180	230	0.782609	37	0	37	0	1	Clonal	0.86	1.81			disease_causing	24.3	p.A411P	Passenger	SENGER/OT	damaging	Deleterious	Damaging
OVA_003_C2	PRIMARY	chr18	33793341	33793341	G	C	MOCOS	exonic	ynonymous	0000261320	81	172	253	0.679842	37	0	37	0	1	Clonal	0.7	3.61			disease_causing	24.3	p.A411P	Passenger	SENGER/OT	damaging	Deleterious	Damaging
OVA_003_C3	PRIMARY	chr18	33793341	33793341	G	C	MOCOS	exonic	ynonymous	0000261320	85	231	316	0.731013	37	0	37	0	1	Clonal	0.82	1.81			disease_causing	24.3	p.A411P	Passenger	SENGER/OT	damaging	Deleterious	Damaging
OVA_047_C2	PRIMARY	chr21	33829969	33829969	G	C	EVA1C	exonic	ynonymous	G141A,ENS	496	145	641	0.226209	92	0	92	0	0.93	Clonal	0.64	2.76			disease_causing	26.4	p.G141A	Passenger	SENGER/OT	damaging	Deleterious	Damaging
OVA_013_M1	METASTASIS	chr15	33922065	33922065	A	G	RYR3	intronic	NA	NA	470	152	622	0.244373	55	0	55	0	0.97	Clonal	0.67	3.06			polymorphism	3.522						
OVA_013_C1	PRIMARY	chr15	33922065	33922065	A	G	RYR3	intronic	NA	NA	461	169	630	0.268254	55	0	55	0	1	Clonal	0.72	3.1			polymorphism	3.522						
OVA_013_C2	PRIMARY	chr15	33922065	33922065	A	G	RYR3	intronic	NA	NA	429	201	630	0.319048	55	0	55	0	1	Clonal	0.87	3.05			polymorphism	3.522						
OVA_013_C3	PRIMARY	chr15	33922065	33922065	A	G	RYR3	intronic	NA	NA	480	190	670	0.283582	55	0	55	0	0.9	Clonal	0.91	3.01			polymorphism	3.522						
OVA_003_M1	METASTASIS	chr9	33926645	33926645	C	A	UBAP2	exonic	ynonymous	50D,ENSG0	398	144	542	0.265683	71	0	71	0	0.94	Sub	0.57	1.84			disease_causing	20.8	p.E827D	Passenger	SENGER/OT	benign	Neutral	Tolerated
OVA_003_M2	METASTASIS	chr9	33926645	33926645	C	A	UBAP2	exonic	ynonymous	50D,ENSG0	445	102	547	0.186472	71	0	71	0	0.81	Sub	0.46	1.81			disease_causing	20.8	p.E827D	Passenger	SENGER/OT	benign	Neutral	Tolerated
OVA_003_M3	METASTASIS	chr9	33926645	33926645	C	A	UBAP2	exonic	ynonymous	50D,ENSG0	541	37	578	0.064014	71	0	71	0	0.67	Sub	0.34	2.67			disease_causing	20.8	p.E827D	Passenger	SENGER/OT	benign	Neutral	Tolerated
OVA_003_C1	PRIMARY	chr9	33926645	33926645	C	A	UBAP2	exonic	ynonymous	50D,ENSG0	320	247	567	0.435626	71	0	71	0	1	Clonal	0.86	1.81			disease_causing	20.8	p.E827D	Passenger	SENGER/OT	benign	Neutral	Tolerated
OVA_003_C2	PRIMARY	chr9	33926645	33926645	C	A	UBAP2	exonic	ynonymous	50D,ENSG0	345	242	587	0.412266	71	0	71	0	1	Clonal	0.7	3.61			disease_causing	20.8	p.E827D	Passenger	SENGER/OT	benign	Neutral	Tolerated
OVA_003_C3	PRIMARY	chr9	33926645	33926645	C	A	UBAP2	exonic	ynonymous	50D,ENSG0	358	231	589	0.39219	71	0	71	0	0.95	Clonal	0.82	1.81			disease_causing	20.8	p.E827D	Passenger	SENGER/OT	benign	Neutral	Tolerated
OVA_365_M1	METASTASIS	chr7	33945239	33945239	C	T	BMPER	exonic	ynonymous	T00000444	62	63	125	0.504	43	0	43	0	1	Clonal	0.85	1.87			polymorphism	18.33	p.S5F	Passenger	SENGER/OT	benign	Neutral	Tolerated
OVA_365_M2	METASTASIS	chr7	33945239	33945239	C	T	BMPER	exonic	ynonymous	T00000444	20	12	32	0.375	43	0	43	0	1	Clonal	0.4	1.81			polymorphism	18.33	p.S5F	Passenger	SENGER/OT	benign	Neutral	Tolerated
OVA_365_M3	METASTASIS	chr7	33945239	33945239	C	T	BMPER	exonic	ynonymous	T00000444	29	38	67	0.567164	43	0	43	0	1	Clonal	0.82	1.89			polymorphism	18.33	p.S5F	Passenger	SENGER/OT	benign	Neutral	Tolerated
OVA_365_C1	Contralateral ovary	chr7	33945239	33945239	C	T	BMPER	exonic	ynonymous	T00000444	54	16	70	0.228571	43	0	43	0	1	Clonal	0.42	1.83			polymorphism	18.33	p.S5F	Passenger	SENGER/OT	benign	Neutral	Tolerated
OVA_365_C2	PRIMARY	chr7	33945239	33945239	C	T	BMPER	exonic	ynonymous	T00000444	48	30	78	0.384615	43	0	43	0	1	Clonal	0.62	1.92			polymorphism	18.33	p.S5F	Passenger	SENGER/OT	benign	Neutral	Tolerated
OVA_365_M1	METASTASIS	chr5	33951772	33951772	C	T	SLC45A2	exonic	ynonymous	000296589	294	216	510	0.423529	39	0	39	0	1	Clonal	0.85	1.87			polymorphism	8.894	p.R348H	Passenger	SENGER/OT	benign	Neutral	Tolerated
OVA_365_M2	METASTASIS	chr5	33951772	33951772	C	T	SLC45A2	exonic	ynonymous	000296589	311	72	383	0.18799	39	0	39	0	0.93	Clonal	0.4	1.81			polymorphism	8.894	p.R348H	Passenger	SENGER/OT	benign	Neutral	Tolerated
OVA_365_M3	METASTASIS	chr5	33951772	33951772	C	T	SLC45A2	exonic	ynonymous	000296589	308	203	511	0.39726	39	0	39	0	0.97	Clonal	0.82	1.89			polymorphism	8.894	p.R348H	Passenger	SENGER/OT	benign	Neutral	Tolerated
OVA_365_C1	Contralateral ovary	chr5	33951772	33951772	C	T	SLC45A2	exonic	ynonymous	000296589	275	74	349	0.212034	39	0	39	0	1	Clonal	0.42	1.83			polymorphism	8.894	p.R348H	Passenger	SENGER/OT	benign	Neutral	Tolerated
OVA_365_C2	PRIMARY	chr5	33951772	33951772	C	T	SLC45A2	exonic	ynonymous	000296589	748	281	1029	0.273081	39	0	39	0	0.89	Sub	0.42	1.83			polymorphism	8.894	p.R348H	Passenger	SENGER/OT	benign	Neutral	Tolerated
OVA_047_C1	PRIMARY	chr15	34040695	34040695	C	G	RYR3	exonic	nonymous S	2721L,ENSG	464	169	633	0.266983	50	0	50	0	0.89	Sub	0.6	2.91			disease_causing	8.484		Passenger			Neutral	Tolerated
OVA_047_C2	PRIMARY	chr15	34040695	34040695	C	G	RYR3	exonic	nonymous S	2721L,ENSG	463	143	606	0.235974	50	0	50	0	0.74	Sub	0.64	2.76			disease_causing	8.484		Passenger			Neutral	Tolerated
OVA_047_C3	Contralateral ovary	chr15	34040695	34040695	C	G	RYR3	exonic	nonymous S	2721L,ENSG	453	158	611	0.258592	50	0	50	0	0.87	Sub	0.6	2.94			disease_causing	8.484		Passenger			Neutral	Tolerated
OVA_048_M1	METASTASIS	chr20	34092423	34092423	G	T	CEP250	exonic	stopgain	00342580:e	40	53	93	0.569892	23	0	23	0	1	Clonal	0.56	3.94			disease_causing_automat	39	p.E2076X	Passenger			NA	NA
OVA_048_M2	METASTASIS	chr20	34092423	34092423	G	T	CEP250	exonic	stopgain	00342580:e	73	44	117	0.376068	23	0	23	0	1	Clonal	0.4	3.87			disease_causing_automat	39	p.E2076X	Passenger			NA	NA
OVA_048_M3	METASTASIS	chr20	34092423	34092423	G	T	CEP250	exonic	stopgain	00342580:e	41	73	114	0.640351	23	0	23	0	1	Clonal	0.59	3.39			disease_causing_automat	39	p.E2076X	Passenger			NA	NA
OVA_048_C1	PRIMARY	chr20	34092423	34092423	G	T	CEP250	exonic	stopgain	00342580:e	23	14	37	0.378378	23	0	23	0	0.75	Clonal	0.67	1.82			disease_causing_automat	39	p.E2076X	Passenger			NA	NA
OVA_048_C2	PRIMARY	chr20	34092423	34092423	G	T	CEP250	exonic	stopgain	00342580:e	48	46	94	0.489362	23	0	23	0	0.87	Clonal	0.72	1.85			disease_causing_automat	39	p.E2076X	Passenger			NA	NA
OVA_048_C3	PRIMARY	chr20	34092423	34092423	G	T	CEP250	exonic	stopgain	00342580:e	29	35	64	0.546875	23	0	23	0	0.93	Clonal	0.74	1.8			disease_causing_automat	39	p.E2076X	Passenger			NA	NA
OVA_047_C1	PRIMARY	chr20	34171781	34171781	G	C	FER1L4	RNA_intron	NA	NA	282	52	334	0.155689	38	0	38	0	0.83	Clonal	0.6	2.91				1.148						
OVA_047_C2	PRIMARY	chr20	34171781	34171781	G	C	FER1L4	RNA_intron	NA	NA	250	57	307	0.185668	38	0	38	0	0.95	Clonal	0.64	2.76				1.148						
OVA_047_C3	Contralateral ovary	chr20	34171781	34171781	G	C	FER1L4	RNA_intron	NA	NA	278	50	328	0.152439	38	0	38	0	0.82	Clonal	0.6	2.94				1.148						
OVA_047_C1	PRIMARY	chr11	34173994	34173994	C	G	ABTB2	exonic	ynonymous	820H,ENSG	249	120	369	0.325203	8	0	8	0	1	Clonal	0.6	2.91			polymorphism	16.97	p.Q1006H	Passenger	SENGER/OT	benign	Neutral	Tolerated
OVA_047_C2	PRIMARY	chr11	34173994	34173994	C	G	ABTB2	exonic	ynonymous	820H,ENSG	225	93	318	0.292453	8	0	8	0	1	Clonal	0.64	2.76			polymorphism	16.97	p.Q1006H	Passenger	SENGER/OT	benign	Neutral	Tolerated
OVA_047_C3	Contralateral ovary	chr11	34173994	34173994	C	G	ABTB2	exonic	ynonymous	820H,ENSG	184	87	271	0.321033	8	0	8	0	1	Clonal	0.6	2.94			polymorphism	16.97	p.Q1006H	Passenger	SENGER/OT	benign	Neutral	Tolerated
OVA_048_M1	METASTASIS	chr11	34176296	34176296	G	C	ABTB2	exonic	stopgain	720X,ENSG	299	13	312	0.041667	68	0	68	0	0.15	Sub	0.56	3.94			disease_causing_automat	40	p.Y906X	Passenger	SENGER/OT		NA	NA
OVA_048_M2	METASTASIS	chr11	34176296	34176296	G	C	ABTB2	exonic	stopgain	720X,ENSG	373	18	391	0.046036	68	0	68	0	0.23	Sub	0.4	3.87			disease_causing_automat	40	p.Y906X	Passenger	SENGER/OT		NA	NA
OVA_048_M3	METASTASIS	chr11	34176296	34176296	G	C	ABTB2	exonic	stopgain	720X,ENSG	241	90	331	0.271903	68	0	68	0	1	Sub	0.59	3.39			disease_causing_automat	40	p.Y906X	Passenger	SENGER/OT		NA	NA
OVA_003_M1	METASTASIS	chr9	34398286	34398286	G	T	FAM219A	UTR3	NA	NA	324	114	438	0.260274	36	0	36	0	0.92	Sub	0.57	1.84			disease_causing	20.2						
OVA_003_M2	METASTASIS	chr9	34398286	34398286	G	T	FAM219A	UTR3	NA	NA	392	111	503	0.220676	36	0	36	0	0.96	Sub	0.46	1.81			disease_causing	20.2						
OVA_003_M3	METASTASIS	chr9	34398286	34398286	G	T	FAM219A	UTR3	NA	NA	480	37	517	0.071567	36	0	36	0	0.75	Sub	0.34	2.67			disease_causing	20.2						
OVA_003_C1	PRIMARY	chr9	34398286	34398286	G	T	FAM219A	UTR3	NA	NA	276	219	495	0.442424	36	0	36	0	1	Clonal	0.86	1.81			disease_causing	20.2						
OVA_003_C2	PRIMARY	chr9	34398286	34398286	G	T	FAM219A	UTR3	NA	NA	312	236	548	0.430657	36	0	36	0	1	Clonal	0.7	3.61			disease_causing	20.2						
OVA_003_C3	PRIMARY	chr9	34398286	34398286	G	T	FAM219A	UTR3	NA	NA	334	222	556	0.399281	36	0	36	0	0.97	Clonal	0.82	1.81			disease_causing	20.2						
OVA_048_C1	PRIMARY	chr16	34490025	34490025	G	A	MP1;LOC28	RNA_intron	NA	NA	9	4	13	0.307692	11	0	11	0	0.92	Clonal	0.67	1.82				4.529						
OVA_048_C2	PRIMARY	chr16	34490025	34490025	G	A	MP1;LOC28	RNA_intron	NA	NA	30	25	55	0.454545	11	0	11	0	1	Clonal	0.72	1.85				4.529						
OVA_048_C3	PRIMARY	chr16	34490025	34490025	G	A	MP1;LOC28	RNA_intron	NA	NA	12	13	25	0.52	11	0	11	0	1	Clonal	0.74	1.8				4.529						
OVA_013_C2	PRIMARY	chr6	34725406	34725406	G	C	SNRPC	UTR5	NA	NA	554	50	604	0.082781	117	0	117	0	0.36	Sub	0.87	3.05			polymorphism	10.61						
OVA_048_M1	METASTASIS	chr6	35088259	35088259	G	T	TCP11	exonic	ynonymous	000051201	224	86	310	0.277419	40	1	41	0.02439	1	Clonal	0.56	3.94	Yes	No	disease_causing	26.2	p.Q307K	Passenger	SENGER/OT	damaging	Neutral	Tolerated
OVA_048_M2	METASTASIS	chr6	35088259	35088259	G	T	TCP11	exonic	ynonymous	000051201	199	61	260	0.234615	40	1	41	0.02439	1	Clonal	0.4	3.87	Yes	No	disease_causing	26.2	p.Q307K	Passenger	SENGER/OT	damaging	Neutral	Tolerated
OVA_048_M3	METASTASIS	chr6	35088259	35088259	G	T	TCP11	exonic	ynonymous	000051201	207	44	251	0.175299	40	1	41	0.02439	1	Clonal	0.59	3.39	Yes	No	disease_causing	26.2	p.Q307K	Passenger	SENGER/OT	damaging	Neutral	Tolerated
OVA_048_C1	PRIMARY	chr6	35088259	35088259	G	T	TCP11	exonic	ynonymous	000051201	103	26	129	0.20155	40	1	41	0.02439	0.8	Clonal	0.67	1.82	Yes	No	disease_causing	26.2	p.Q307K	Passenger	SENGER/OT	damaging	Neutral	Tolerated
OVA_048_C3	PRIMARY	chr6	35088259	35088259	G	T	TCP11	exonic	ynonymous	000051201	122	48	170	0.282353	40	1	41	0.02439	1	Clonal	0.74	1.8	Yes	No	disease_causing	26.2	p.Q307K	Passenger	SENGER/OT	damaging	Neutral	Tolerated
OVA_013_M1	METASTASIS	chr6	35089968	35089968	A	C	TCP11	exonic	nonymous S	000512012	238	393	631	0.622821	50	1	51	0.019608	1	Clonal	0.67	3.06	Yes		polymorphism	4.668		Passenger			Neutral	Tolerated
OVA_013_C1	PRIMARY	chr6	35089968	35089968	A	C	TCP11	exonic	nonymous S	000512012	211	429	640	0.670313	50	1	51	0.019608	1	Clonal	0.72	3.1	Yes		polymorphism	4.668		Passenger			Neutral	Tolerated
OVA_013_C2	PRIMARY	chr6	35089968	35089968	A	C	TCP11	exonic	nonymous S	000512012	212	428	640	0.66875	50	1	51	0.019608	1	Clonal	0.87	3.05	Yes		polymorphism	4.668		Passenger			Neutral	Tolerated
OVA_013_C3	PRIMARY	chr6	35089968	35089968	A	C	TCP11	exonic	nonymous S	000512012	194	486	680	0.714706	50	1	51	0.019608	1	Clonal	0.91	3.01	Yes		polymorphism	4.668		Passenger			Neutral	Tolerated
OVA_003_M1	METASTASIS	chr6	35425392	35425392	A	G	FANCE	intronic	NA	NA	299	117	416	0.28125	79	0	79	0	1	Sub	0.57	1.84	Yes		polymorphism	5.707						
OVA_003_M2	METASTASIS	chr6	35425392	35425392	A	G	FANCE	intronic	NA	NA	352	114	466	0.244635	79	0	79	0	1	Sub	0.46	1.81	Yes		polymorphism	5.707						
OVA_003_M3	METASTASIS	chr6	35425392	35425392	A	G	FANCE	intronic	NA	NA	460	36	496	0.072581	79	0	79	0	0.77	Sub	0.34	2.67	Yes		polymorphism	5.707						
OVA_003_C1	PRIMARY	chr6	35425392	35425392	A	G	FANCE	intronic	NA	NA	329	245	574	0.426829	79	0	79	0	0.99	Clonal	0.86	1.81	Yes		polymorphism	5.707						
OVA_003_C2	PRIMARY	chr6	35425392	35425392	A	G	FANCE	intronic	NA	NA	333	223	556	0.401079	79	0	79	0	1	Clonal	0.7	3.61	Yes		polymorphism	5.707						
OVA_003_C3	PRIMARY	chr6	35425392	35425392	A	G	FANCE	intronic	NA	NA	367	234	601	0.389351	79	0	79	0	0.94	Clonal	0.82	1.81	Yes		polymorphism	5.707						
OVA_003_M1	METASTASIS	chr9	35649754	35649754	C	T	X748309;SI	UTR3	NA	NA	242	87	329	0.264438	9	0	9	0	0.94	Sub	0.57	1.84			polymorphism	14.42						
OVA_003_M2	METASTASIS	chr9	35649754	35649754	C	T	X748309;SI	UTR3	NA	NA	361	81	442	0.183258	9	0	9	0	0.8	Sub	0.46	1.81			polymorphism	14.42						
OVA_003_M3	METASTASIS	chr9	35649754	35649754	C	T	X748309;SI	UTR3	NA	NA	416	34	450	0.075556	9	0	9	0	0.8	Sub	0.34	2.67			polymorphism	14.42						
OVA_003_C1	PRIMARY	chr9	35649754	35649754	C	T	X748309;SI	UTR3	NA	NA	246	180	426	0.422535	9	0	9	0	0.98	Clonal	0.86	1.81			polymorphism	14.42						
OVA_003_C2	PRIMARY	chr9	35649754	35649754	C	T	X748309;SI	UTR3	NA	NA	301	196	497	0.394366	9	0	9	0	1	Clonal	0.7	3.61			polymorphism	14.42						
OVA_003_C3	PRIMARY	chr9	35649754	35649754	C	T	X748309;SI	UTR3	NA	NA	276	208	484	0.429752	9	0	9	0	1	Clonal	0.82	1.81			polymorphism	14.42						
OVA_003_M1	METASTASIS	chr9	35662177	35662177	T	A	ARHGEF39	exonic	ynonymous	000037839	400	185	585	0.316239	64	1	65	0.015385	1	Sub	0.57	1.84			disease_causing	21.9	p.S331C	Passenger	SENGER/OT	damaging	Neutral	Damaging
OVA_003_M2	METASTASIS	chr9	35662177	35662177	T	A	ARHGEF39	exonic	ynonymous	000037839	455	127	582	0.218213	64	1	65	0.015385	0.95	Sub	0.46	1.81			disease_causing	21.9	p.S331C	Passenger	SENGER/OT	damaging	Neutral	Damaging
OVA_003_M3	METASTASIS	chr9	35662177	35662177	T	A	ARHGEF39	exonic	ynonymous	000037839	627	39	666	0.058559	64	1	65	0.015385	0.62	Sub	0.34	2.67			disease_causing	21.9	p.S331C	Passenger	SENGER/OT	damaging	Neutral	Damaging
OVA_003_C1	PRIMARY	chr9	35662177	35662177	T	A	ARHGEF39	exonic	ynonymous	000037839	369	274	643	0.426128	64	1	65	0.015385	0.99	Clonal	0.86	1.81			disease_causing	21.9	p.S331C	Passenger	SENGER/OT	damaging	Neutral	Damaging
OVA_003_C2	PRIMARY	chr9	35662177	35662177	T	A	ARHGEF39	exonic	ynonymous	000037839	339	275	614	0.447883	64	1	65	0.015385	1	Clonal	0.7	3.61			disease_causing	21.9	p.S331C	Passenger	SENGER/OT	damaging	Neutral	Damaging
OVA_003_C3	PRIMARY	chr9	35662177	35662177	T	A	ARHGEF39	exonic	ynonymous	000037839	377	282	659	0.427921	64	1	65	0.015385	1	Clonal	0.82	1.81			disease_causing	21.9	p.S331C	Passenger	SENGER/OT	damaging	Neutral	Damaging
OVA_048_M1	METASTASIS	chr9	35706991	35706991	C	G	TLN1	intronic	NA	NA	391	256	647	0.395672	23	0	23	0	1	Clonal	0.56	3.94			polymorphism	0.048						
OVA_048_M2	METASTASIS	chr9	35706991	35706991	C	G	TLN1	intronic	NA	NA	443	217	660	0.328788	23	0	23	0	1	Clonal	0.4	3.87			polymorphism	0.048						
OVA_048_M3	METASTASIS	chr9	35706991	35706991	C	G	TLN1	intronic	NA	NA	419	233	652	0.357362	23	0	23	0	1	Clonal	0.59	3.39			polymorphism	0.048						
OVA_048_C1	PRIMARY	chr9	35706991	35706991	C	G	TLN1	intronic	NA	NA	348	107	455	0.235165	23	0	23	0	0.94	Sub	0.67	1.82			polymorphism	0.048						
OVA_048_C2	PRIMARY	chr9	35706991	35706991	C	G	TLN1	intronic	NA	NA	354	232	586	0.395904	23	0	23	0	1	Sub	0.72	1.85			polymorphism	0.048						
OVA_048_C3	PRIMARY	chr9	35706991	35706991	C	G	TLN1	intronic	NA	NA	355	152	507	0.299803	23	0	23	0	0.81	Sub	0.74	1.8			polymorphism	0.048						
OVA_013_M1	METASTASIS	chr6	35854650	35854650	G	A	SRPK1	intronic	NA	NA	338	236	574	0.41115	29	0	29	0	1	Clonal	0.67	3.06			polymorphism	0.23						
OVA_013_C1	PRIMARY	chr6	35854650	35854650	G	A	SRPK1	intronic	NA	NA	324	238	562	0.423488	29	0	29	0	1	Clonal	0.72	3.1			polymorphism	0.23						
OVA_013_C2	PRIMARY	chr6	35854650	35854650	G	A	SRPK1	intronic	NA	NA	315	238	553	0.43038	29	0	29	0	1	Clonal	0.87	3.05			polymorphism	0.23						
OVA_013_C3	PRIMARY	chr6	35854650	35854650	G	A	SRPK1	intronic	NA	NA	316	244	560	0.435714	29	0	29	0	1	Clonal	0.91	3.01			polymorphism	0.23						

Sample	Type	Chr	Start	End	Ref	Alt	Gene	unc.ensGe	icFunc.ens	hange.ens	t_ref	t_alt	t_total	t_VAF	n_ref	n_alt	n_total	normal_VA	ncer_cell_f	on-based C	purity	ploidy	Cancer Gene	Driver Gene	Mutation Taster	ADD(PHRE	otein_chan	CHASM	THMM_Pr	OLYPHEN-	REDICTION	ICTION (cutoff=0.05)
OVA_048_M1	METASTASIS	chr5	35954325	35954325	G	A	UGT3A1	exonic	nonymous S	000027427	232	122	354	0.344633	10	0	10	0	1	Clonal	0.56	3.94			disease_causing	2.909		Passenger			Neutral	Tolerated
OVA_048_M2	METASTASIS	chr5	35954325	35954325	G	A	UGT3A1	exonic	nonymous S	000027427	283	121	404	0.299505	10	0	10	0	1	Clonal	0.4	3.87			disease_causing	2.909		Passenger			Neutral	Tolerated
OVA_048_M3	METASTASIS	chr5	35954325	35954325	G	A	UGT3A1	exonic	nonymous S	000027427	291	161	452	0.356195	10	0	10	0	1	Clonal	0.59	3.39			disease_causing	2.909		Passenger			Neutral	Tolerated
OVA_048_C1	PRIMARY	chr5	35954325	35954325	G	A	UGT3A1	exonic	nonymous S	000027427	54	36	90	0.4	10	0	10	0	1	Clonal	0.67	1.82			disease_causing	2.909		Passenger			Neutral	Tolerated
OVA_048_C2	PRIMARY	chr5	35954325	35954325	G	A	UGT3A1	exonic	nonymous S	000027427	186	231	417	0.553957	10	0	10	0	1	Clonal	0.72	1.85			disease_causing	2.909		Passenger			Neutral	Tolerated
OVA_048_C3	PRIMARY	chr5	35954325	35954325	G	A	UGT3A1	exonic	nonymous S	000027427	108	58	166	0.349398	10	0	10	0	0.94	Clonal	0.74	1.8			disease_causing	2.909		Passenger			Neutral	Tolerated
OVA_048_M1	METASTASIS	chr19	36168972	36168972	C	T	UPK1A	UTR3	NA	NA	460	46	506	0.090909	9	0	9	0	0.5	Sub	0.56	3.94			disease_causing	9.421						
OVA_365_M2	METASTASIS	chr6	36260932	36260932	G	A	PNPLA1	intronic	NA	NA	122	15	137	0.109489	86	0	86	0	0.76	Clonal	0.4	1.81			polymorphism	3.224						
OVA_048_M1	METASTASIS	chr6	36274103	36274103	C	T	PNPLA1	exonic	nonymous S	S378S,ENSG	269	150	419	0.357995	35	0	35	0	1	Clonal	0.56	3.94			polymorphism	0.365		Passenger			Neutral	Tolerated
OVA_048_M2	METASTASIS	chr6	36274103	36274103	C	T	PNPLA1	exonic	nonymous S	S378S,ENSG	332	117	449	0.260579	35	0	35	0	1	Clonal	0.4	3.87			polymorphism	0.365		Passenger			Neutral	Tolerated
OVA_048_M3	METASTASIS	chr6	36274103	36274103	C	T	PNPLA1	exonic	nonymous S	S378S,ENSG	242	171	413	0.414044	35	0	35	0	1	Clonal	0.59	3.39			polymorphism	0.365		Passenger			Neutral	Tolerated
OVA_047_M1	METASTASIS	chr19	36303084	36303084	G	T	PRODH2	exonic	nonymous S	000030117	252	107	359	0.29805	31	0	31	0	1	Clonal	0.47	2.01			polymorphism	1.822		Passenger			Neutral	Tolerated
OVA_047_M2	METASTASIS	chr19	36303084	36303084	G	T	PRODH2	exonic	nonymous S	000030117	334	104	438	0.237443	31	0	31	0	1	Clonal	0.44	2.01			polymorphism	1.822		Passenger			Neutral	Tolerated
OVA_047_M3	METASTASIS	chr19	36303084	36303084	G	T	PRODH2	exonic	nonymous S	000030117	133	46	179	0.256983	31	0	31	0	1	Clonal	0.6	3.02			polymorphism	1.822		Passenger			Neutral	Tolerated
OVA_048_M1	METASTASIS	chr19	36333246	36333246	C	A	NPHS1	intronic	NA	NA	250	160	410	0.390244	39	1	40	0.025	1	Clonal	0.56	3.94			polymorphism	10.37						
OVA_048_M2	METASTASIS	chr19	36333246	36333246	C	A	NPHS1	intronic	NA	NA	334	142	476	0.298319	39	1	40	0.025	1	Clonal	0.4	3.87			polymorphism	10.37						
OVA_048_M3	METASTASIS	chr19	36333246	36333246	C	A	NPHS1	intronic	NA	NA	271	183	454	0.403084	39	1	40	0.025	1	Clonal	0.59	3.39			polymorphism	10.37						
OVA_048_C1	PRIMARY	chr19	36333246	36333246	C	A	NPHS1	intronic	NA	NA	86	33	119	0.277311	39	1	40	0.025	0.83	Clonal	0.67	1.82			polymorphism	10.37						
OVA_048_C2	PRIMARY	chr19	36333246	36333246	C	A	NPHS1	intronic	NA	NA	207	127	334	0.38024	39	1	40	0.025	1	Clonal	0.72	1.85			polymorphism	10.37						
OVA_048_C3	PRIMARY	chr19	36333246	36333246	C	A	NPHS1	intronic	NA	NA	118	80	198	0.40404	39	1	40	0.025	1	Clonal	0.74	1.8			polymorphism	10.37						
OVA_365_C2	PRIMARY	chr6	36343658	36343658	C	T	ETV7	exonic	nonymous S	000034018	332	106	438	0.242009	116	0	116	0	1	Clonal	0.62	1.92			disease_causing	0.354		Passenger		benign	Neutral	Tolerated
OVA_047_C1	PRIMARY	chr19	36353384	36353384	G	A	KIRREL2	intronic	NA	NA	307	119	426	0.279343	47	0	47	0	1	Clonal	0.6	2.91			polymorphism	10.77						
OVA_047_C2	PRIMARY	chr19	36353384	36353384	G	A	KIRREL2	intronic	NA	NA	365	120	485	0.247423	47	0	47	0	1	Clonal	0.64	2.76			polymorphism	10.77						
OVA_047_C3	Contralateral ovary	chr19	36353384	36353384	G	A	KIRREL2	intronic	NA	NA	310	104	414	0.251208	47	0	47	0	1	Clonal	0.6	2.94			polymorphism	10.77						
OVA_047_C3	Contralateral ovary	chr19	36594392	36594392	C	G	WDR62	exonic	ynonymou	1216S,ENSG	372	111	483	0.229814	54	0	54	0	1	Clonal	0.6	2.94			polymorphism	21.2	p.T1221S	Passenger	SENGER/OT	benign	Neutral	Damaging
OVA_365_M1	METASTASIS	chr22	36623979	36623979	G	C	APOL2	exonic	ynonymou	000024906	67	240	307	0.781759	73	0	73	0	1	Clonal	0.85	1.87			polymorphism	9.203	p.A274G	Passenger	SENGER/OT	benign	Neutral	Tolerated
OVA_365_M2	METASTASIS	chr22	36623979	36623979	G	C	APOL2	exonic	ynonymou	000024906	280	95	375	0.253333	73	0	73	0	1	Clonal	0.4	1.81			polymorphism	9.203	p.A274G	Passenger	SENGER/OT	benign	Neutral	Tolerated
OVA_365_M3	METASTASIS	chr22	36623979	36623979	G	C	APOL2	exonic	ynonymou	000024906	77	255	332	0.768072	73	0	73	0	1	Clonal	0.82	1.89			polymorphism	9.203	p.A274G	Passenger	SENGER/OT	benign	Neutral	Tolerated
OVA_365_C1	Contralateral ovary	chr22	36623979	36623979	G	C	APOL2	exonic	ynonymou	000024906	285	108	393	0.274809	73	0	73	0	1	Clonal	0.42	1.83			polymorphism	9.203	p.A274G	Passenger	SENGER/OT	benign	Neutral	Tolerated
OVA_365_C2	PRIMARY	chr22	36623979	36623979	G	C	APOL2	exonic	ynonymou	000024906	238	201	439	0.457859	73	0	73	0	1	Clonal	0.62	1.92			polymorphism	9.203	p.A274G	Passenger	SENGER/OT	benign	Neutral	Tolerated
OVA_047_C3	Contralateral ovary	chr22	36684912	36684912	A	G	MYH9	exonic	ynonymou	000216181	128	72	200	0.36	46	0	46	0	1	Clonal	0.6	2.94	Yes	Yes	disease_causing	32	p.L1544P	Passenger	SENGER/OT	damaging	Deleterious	Damaging
OVA_048_M1	METASTASIS	chr6	36742846	36742846	G	C	CPNE5	intronic	NA	NA	477	139	616	0.225649	45	0	45	0	1	Clonal	0.56	3.94			disease_causing	11.39						
OVA_048_M2	METASTASIS	chr6	36742846	36742846	G	C	CPNE5	intronic	NA	NA	494	108	602	0.179402	45	0	45	0	1	Clonal	0.4	3.87			disease_causing	11.39						
OVA_048_M3	METASTASIS	chr6	36742846	36742846	G	C	CPNE5	intronic	NA	NA	461	116	577	0.20104	45	0	45	0	1	Clonal	0.59	3.39			disease_causing	11.39						
OVA_048_C1	PRIMARY	chr6	36742846	36742846	G	C	CPNE5	intronic	NA	NA	292	96	388	0.247423	45	0	45	0	0.99	Clonal	0.67	1.82			disease_causing	11.39						
OVA_048_C2	PRIMARY	chr6	36742846	36742846	G	C	CPNE5	intronic	NA	NA	288	306	594	0.515152	45	0	45	0	1	Clonal	0.72	1.85			disease_causing	11.39						
OVA_048_C3	PRIMARY	chr6	36742846	36742846	G	C	CPNE5	intronic	NA	NA	342	112	454	0.246696	45	0	45	0	0.91	Clonal	0.74	1.8			disease_causing	11.39						
OVA_047_C1	PRIMARY	chr1	36772217	36772217	G	A	SH3D21	exonic	ynonymou	p.G8E,ENSG	33	14	47	0.297872	26	0	26	0	0.99	Sub	0.6	2.91			polymorphism	22.6	p.G8E	Passenger	SENGER/OT	damaging	Neutral	Tolerated
OVA_047_C2	PRIMARY	chr1	36772217	36772217	G	A	SH3D21	exonic	ynonymou	p.G8E,ENSG	24	4	28	0.142857	26	0	26	0	0.45	Sub	0.64	2.76			polymorphism	22.6	p.G8E	Passenger	SENGER/OT	damaging	Neutral	Tolerated
OVA_047_C3	Contralateral ovary	chr1	36772217	36772217	G	A	SH3D21	exonic	ynonymou	p.G8E,ENSG	17	12	29	0.413793	26	0	26	0	1	Clonal	0.6	2.94			polymorphism	22.6	p.G8E	Passenger	SENGER/OT	damaging	Neutral	Tolerated
OVA_378_M1	METASTASIS	chr6	36839574	36839574	C	G	PPIL1	exonic	ynonymou	000003736	401	177	578	0.306228	31	0	31	0	0.85	Sub	0.72	2.57			disease_causing	31	p.R44P	Passenger	SENGER/OT	damaging	Deleterious	Damaging
OVA_378_M2	METASTASIS	chr6	36839574	36839574	C	G	PPIL1	exonic	ynonymou	000003736	381	206	587	0.350937	31	0	31	0	0.82	Sub	0.85	2.57			disease_causing	31	p.R44P	Passenger	SENGER/OT	damaging	Deleterious	Damaging
OVA_378_M3	METASTASIS	chr6	36839574	36839574	C	G	PPIL1	exonic	ynonymou	000003736	441	199	640	0.310938	31	0	31	0	0.93	Sub	0.67	2.57			disease_causing	31	p.R44P	Passenger	SENGER/OT	damaging	Deleterious	Damaging
OVA_378_C1	PRIMARY	chr6	36839574	36839574	C	G	PPIL1	exonic	ynonymou	000003736	432	197	629	0.313196	31	0	31	0	1	Sub	0.59	2.6			disease_causing	31	p.R44P	Passenger	SENGER/OT	damaging	Deleterious	Damaging
OVA_378_C2	PRIMARY	chr6	36839574	36839574	C	G	PPIL1	exonic	ynonymou	000003736	458	147	605	0.242975	31	0	31	0	0.86	Sub	0.56	2.59			disease_causing	31	p.R44P	Passenger	SENGER/OT	damaging	Deleterious	Damaging
OVA_378_C3	PRIMARY	chr6	36839574	36839574	C	G	PPIL1	exonic	ynonymou	000003736	468	125	593	0.210793	31	0	31	0	0.83	Sub	0.51	2.6			disease_causing	31	p.R44P	Passenger	SENGER/OT	damaging	Deleterious	Damaging
OVA_003_M1	METASTASIS	chr20	36869601	36869601	G	C	KIAA1755	exonic	ynonymou	000027902	354	142	496	0.28629	67	1	68	0.014706	1	Sub	0.57	1.84			polymorphism	2.644	p.T311S	Passenger	SENGER/OT	benign	Neutral	Tolerated
OVA_003_M2	METASTASIS	chr20	36869601	36869601	G	C	KIAA1755	exonic	ynonymou	000027902	386	129	515	0.250485	67	1	68	0.014706	1	Sub	0.46	1.81			polymorphism	2.644	p.T311S	Passenger	SENGER/OT	benign	Neutral	Tolerated
OVA_003_M3	METASTASIS	chr20	36869601	36869601	G	C	KIAA1755	exonic	ynonymou	000027902	546	33	579	0.056995	67	1	68	0.014706	0.6	Sub	0.34	2.67			polymorphism	2.644	p.T311S	Passenger	SENGER/OT	benign	Neutral	Tolerated
OVA_003_C1	PRIMARY	chr20	36869601	36869601	G	C	KIAA1755	exonic	ynonymou	000027902	332	259	591	0.43824	67	1	68	0.014706	1	Clonal	0.86	1.81			polymorphism	2.644	p.T311S	Passenger	SENGER/OT	benign	Neutral	Tolerated
OVA_003_C2	PRIMARY	chr20	36869601	36869601	G	C	KIAA1755	exonic	ynonymou	000027902	357	245	602	0.406977	67	1	68	0.014706	1	Clonal	0.7	3.61			polymorphism	2.644	p.T311S	Passenger	SENGER/OT	benign	Neutral	Tolerated
OVA_003_C3	PRIMARY	chr20	36869601	36869601	G	C	KIAA1755	exonic	ynonymou	000027902	379	240	619	0.387722	67	1	68	0.014706	0.94	Clonal	0.82	1.81			polymorphism	2.644	p.T311S	Passenger	SENGER/OT	benign	Neutral	Tolerated
OVA_048_C1	PRIMARY	chr1	36897404	36897404	C	T	OSCP1	exonic	ynonymou	127Q,ENSG	177	84	261	0.321839	41	0	41	0	0.96	Clonal	0.67	1.82			disease_causing	35	p.R182Q	Passenger	SENGER/OT	damaging	Neutral	Damaging
OVA_048_C2	PRIMARY	chr1	36897404	36897404	C	T	OSCP1	exonic	ynonymou	127Q,ENSG	317	218	535	0.407477	41	0	41	0	1	Clonal	0.72	1.85			disease_causing	35	p.R182Q	Passenger	SENGER/OT	damaging	Neutral	Damaging
OVA_048_C3	PRIMARY	chr1	36897404	36897404	C	T	OSCP1	exonic	ynonymou	127Q,ENSG	218	113	331	0.34139	41	0	41	0	0.92	Clonal	0.74	1.8			disease_causing	35	p.R182Q	Passenger	SENGER/OT	damaging	Neutral	Damaging
OVA_047_C1	PRIMARY	chr7	36910050	36910050	G	A	ELMO1	exonic	ynonymou	M,ENSG000	582	110	692	0.15896	29	0	29	0	0.69	Sub	0.6	2.91			disease_causing	26.1	p.T618M	Passenger	SENGER/OT	damaging	Deleterious	Tolerated
OVA_047_C2	PRIMARY	chr7	36910050	36910050	G	A	ELMO1	exonic	ynonymou	M,ENSG000	543	110	653	0.168453	29	0	29	0	0.7	Sub	0.64	2.76			disease_causing	26.1	p.T618M	Passenger	SENGER/OT	damaging	Deleterious	Tolerated
OVA_047_C3	Contralateral ovary	chr7	36910050	36910050	G	A	ELMO1	exonic	ynonymou	M,ENSG000	576	92	668	0.137725	29	0	29	0	0.74	Sub	0.6	2.94			disease_causing	26.1	p.T618M	Passenger	SENGER/OT	damaging	Deleterious	Tolerated
OVA_013_M1	METASTASIS	chr4	37445967	37445967	T	G	KIAA1239	exonic	ynonymou	000030944	489	100	589	0.169779	64	0	64	0	0.85	Clonal	0.67	3.06			disease_causing	22.4	p.L786R	Passenger	SENGER/OT	damaging	Neutral	Damaging
OVA_013_C1	PRIMARY	chr4	37445967	37445967	T	G	KIAA1239	exonic	ynonymou	000030944	465	146	611	0.238953	64	0	64	0	1	Sub	0.72	3.1			disease_causing	22.4	p.L786R	Passenger	SENGER/OT	damaging	Neutral	Damaging
OVA_013_C2	PRIMARY	chr4	37445967	37445967	T	G	KIAA1239	exonic	ynonymou	000030944	493	110	603	0.182421	64	0	64	0	0.78	Sub	0.87	3.05			disease_causing	22.4	p.L786R	Passenger	SENGER/OT	damaging	Neutral	Damaging
OVA_048_M1	METASTASIS	chrX	37932892	37932892	G	T	SYTL5	exonic	ynonymou	000029787	213	126	339	0.371681	16	0	16	0	1	Clonal	0.56	3.94			polymorphism	13.96	p.K165N	Passenger	SENGER/OT	benign	Neutral	Tolerated
OVA_048_M2	METASTASIS	chrX	37932892	37932892	G	T	SYTL5	exonic	ynonymou	000029787	218	84	302	0.278146	16	0	16	0	1	Clonal	0.4	3.87			polymorphism	13.96	p.K165N	Passenger	SENGER/OT	benign	Neutral	Tolerated
OVA_048_M3	METASTASIS	chrX	37932892	37932892	G	T	SYTL5	exonic	ynonymou	000029787	172	127	299	0.424749	16	0	16	0	1	Clonal	0.59	3.39			polymorphism	13.96	p.K165N	Passenger	SENGER/OT	benign	Neutral	Tolerated
OVA_048_C1	PRIMARY	chrX	37932892	37932892	G	T	SYTL5	exonic	ynonymou	000029787	101	52	153	0.339869	16	0	16	0	1	Clonal	0.67	1.82			polymorphism	13.96	p.K165N	Passenger	SENGER/OT	benign	Neutral	Tolerated
OVA_048_C2	PRIMARY	chrX	37932892	37932892	G	T	SYTL5	exonic	ynonymou	000029787	209	158	367	0.430518	16	0	16	0	1	Clonal	0.72	1.85			polymorphism	13.96	p.K165N	Passenger	SENGER/OT	benign	Neutral	Tolerated
OVA_048_C3	PRIMARY	chrX	37932892	37932892	G	T	SYTL5	exonic	ynonymou	000029787	115	64	179	0.357542	16	0	16	0	0.97	Clonal	0.74	1.8			polymorphism	13.96	p.K165N	Passenger	SENGER/OT	benign	Neutral	Tolerated
OVA_378_C1	PRIMARY	chr19	38028529	38028529	G	T	ZNF793	exonic	ynonymou	000058714	579	134	713	0.187938	36	1	37	0.027027	0.83	Sub	0.59	2.6			polymorphism	21.9	p.K323N	Passenger	SENGER/OT	damaging	Deleterious	Tolerated
OVA_378_C2	PRIMARY	chr19	38028529	38028529	G	T	ZNF793	exonic	ynonymou	000058714	529	138	667	0.206897	36	1	37	0.027027	0.94	Sub	0.56	2.59			polymorphism	21.9	p.K323N	Passenger	SENGER/OT	damaging	Deleterious	Tolerated
OVA_378_C3	PRIMARY	chr19	38028529	38028529	G	T	ZNF793	exonic	ynonymou	000058714	529	136	665	0.204511	36	1	37	0.027027	1	Sub	0.51	2.6			polymorphism	21.9	p.K323N	Passenger	SENGER/OT	damaging	Deleterious	Tolerated
OVA_013_M1	METASTASIS	chr4	38097618	38097618	G	T	TBC1D1	exonic	stopgain	E769X,ENSG	518	122	640	0.190625	62	1	63	0.015873	0.95	Clonal	0.67	3.06			disease_causing_automat	46	p.E769X	Passenger			NA	NA
OVA_013_C1	PRIMARY	chr4	38097618	38097618	G	T	TBC1D1	exonic	stopgain	E769X,ENSG	497	151	648	0.233025	62	1	63	0.015873	1	Clonal	0.72	3.1			disease_causing_automat	46	p.E769X	Passenger			NA	NA
OVA_013_C2	PRIMARY	chr4	38097618	38097618	G	T	TBC1D1	exonic	stopgain	E769X,ENSG	465	141	606	0.232673	62	1	63	0.015873	1	Clonal	0.87	3.05			disease_causing_automat	46	p.E769X	Passenger			NA	NA
OVA_013_C3	PRIMARY	chr4	38097618	38097618	G	T	TBC1D1	exonic	stopgain	E769X,ENSG	445	177	622	0.284566	62	1	63	0.015873	0.91	Clonal	0.91	3.01			disease_causing_automat	46	p.E769X	Passenger			NA	NA
OVA_048_C2	PRIMARY	chr19	38315270	38315270	C	G	DC1006313	cRNA_exon	NA	NA	337	176	513	0.34308	36	0	36	0	0.95	Clonal	0.72	1.85				2.946						
OVA_047_C1	PRIMARY	chr10	38343609	38343609	A	G	ZNF33A	exonic	ynonymou	I192R,ENSG	301	102	403	0.253102	25	0	25	0	1	Clonal	0.6	2.91			polymorphism	15.4	p.H204R	Passenger		benign	Deleterious	Damaging
OVA_047_C2	PRIMARY	chr10	38343609	38343609	A	G	ZNF33A	exonic	ynonymou	I192R,ENSG	331	98	429	0.228438	25	0	25	0	0.94	Clonal	0.64	2.76			polymorphism	15.4	p.H204R	Passenger		benign	Deleterious	Damaging
OVA_047_C3	Contralateral ovary	chr10	38343609	38343609	A	G	ZNF33A	exonic	ynonymou	I192R,ENSG	267	88	355	0.247887	25	0	25	0	1	Clonal	0.6	2.94			polymorphism	15.4	p.H204R	Passenger		benign	Deleterious	Damaging
OVA_378_C3	PRIMARY	chr19	38384525	38384525	C	G	WDR87	exonic	nonymous S	L567L,ENSG	553	46	599	0.076795	70	0	70	0	0.38	Sub	0.51	2.6			polymorphism	7.634		Passenger			Neutral	Tolerated
OVA_365_M1	METASTASIS	chr5	38403829	38403829	C	T	EGFLAM	UTR5	NA	NA	154	104	258	0.403101	37	0	37	0	0.95	Sub	0.85	1.87			polymorphism	0.48						
OVA_365_M2	METASTASIS	chr5	38403829	38403829	C	T	EGFLAM	UTR5	NA	NA	103	12	115	0.104348	37	0	37	0	0.52	Sub	0.4	1.81			polymorphism	0.48						
OVA_365_M3	METASTASIS	chr5	38403829	38403829	C	T	EGFLAM	UTR5	NA	NA	151	99	250	0.396	37	0	37	0	0.97	Sub	0.82	1.89			polymorphism	0.48						
OVA_365_C1	Contralateral ovary	chr5	38403829	38403829	C	T	EGFLAM	UTR5	NA	NA	108	29	137	0.211679	37	0	37	0	1	Clonal	0.42	1.83			polymorphism	0.48						
OVA_365_C2	PRIMARY	chr5	38403829	38403829	C	T	EGFLAM	UTR5	NA	NA	172	69	241	0.286307	37	0	37	0	0.93	Clonal	0.62	1.92			polymorphism	0.48						
OVA_048_M1	METASTASIS	chr19	38573615	38573615	C	T	SIPA1L3	exonic	nonymous S	000022234	88	53	141	0.375887	49	0	49	0	1	Clonal	0.56	3.94			disease_causing	9.264		Passenger			Neutral	Tolerated
OVA_048_M2	METASTASIS	chr19	38573615	38573615	C	T	SIPA1L3	exonic	nonymous S	000022234	127	69	196	0.352041	49	0	49	0	1	Clonal	0.4	3.87			disease_causing	9.264		Passenger			Neutral	Tolerated
OVA_048_M3	METASTASIS	chr19	38573615	38573615	C	T	SIPA1L3	exonic	nonymous S	000022234	82	70	152	0.460526	49	0	49	0	1	Clonal	0.59	3.39			disease_causing	9.264		Passenger			Neutral	Tolerated
OVA_048_C2	PRIMARY	chr17	38643484	38643484	G	C	TNS4	exonic	nonymous S	000025405	133	103	236	0.436441	82	0	82	0	0.78	Sub	0.72	1.85			disease_causing	9.35		Passenger			Neutral	Tolerated
OVA_047_C1	PRIMARY	chr3	38662381	38662381	G	T	SCN5A	exonic	ynonymou	F188L,ENSG	486	165	651	0.253456	39	0	39	0	1	Clonal	0.6	2.91			disease_causing	23	p.F188L	Passenger	SENGER/OT	damaging	Deleterious	Damaging
OVA_047_C2	PRIMARY	chr3	38662381	38662381	G	T	SCN5A	exonic	ynonymou	F188L,ENSG	409	191	600	0.318333	39	0	39	0	1	Clonal	0.64	2.76			disease_causing	23	p.F188L	Passenger	SENGER/OT	damaging	Deleterious	Damaging
OVA_047_C3	Contralateral ovary	chr3	38662381	38662381	G	T	SCN5A	exonic	ynonymou	F188L,ENSG	495	133	628	0.211783	39	0	39	0	0.92	Clonal	0.6	2.94			disease_causing	23	p.F188L	Passenger	SENGER/OT	damaging	Deleterious	Damaging
OVA_047_M1	METASTASIS	chr6	38670851	38670851	G	C	GLO1	UTR5	NA	NA	503	104	607	0.171334	137	0	137	0	0.89	Sub	0.47	2.01			polymorphism	8.345						
OVA_047_M2	METASTASIS	chr6	38670851	38670851	G	C	GLO1	UTR5	NA	NA	535	96	631	0.152139	137	0	137	0	0.84	Sub	0.44	2.01			polymorphism	8.345						
OVA_047_M3	METASTASIS	chr6	38670851	38670851	G	C	GLO1	UTR5	NA	NA	194	53	247	0.214575	137	0	137	0	1	Sub	0.6	3.02			polymorphism	8.345						
OVA_013_M1	METASTASIS	chr6	38723925	38723925	T	A	DNAH8	intronic	NA	NA	512	109	621	0.175523	52	0	52	0	1	Clonal	0.67	3.06			polymorphism	3.376						
OVA_013_C1	PRIMARY	chr6	38723925	38723925	T	A	DNAH8	intronic	NA	NA	512	110	622	0.176849	52	0	52	0	1	Clonal	0.72	3.1			polymorphism	3.376						
OVA_013_C2	PRIMARY	chr6	38723925	38723925	T	A	DNAH8	intronic	NA	NA	511	102	613	0.166395	52	0	52	0	1	Clonal	0.87	3.05			polymorphism	3.376						
OVA_013_C3	PRIMARY	chr6	38723925	38723925	T	A	DNAH8	intronic	NA	NA	533	107	640	0.167188	52	0	52	0	1	Clonal	0.91	3.01			polymorphism	3.376						
OVA_013_M1	METASTASIS	chr8	38836626	38836626	G	C	HTRA4	intronic	NA	NA	463	128	591	0.216582	28	0	28	0	0.55	Sub_	0.67	3.06			polymorphism	3.416						
OVA_013_C1	PRIMARY	chr8	38836626	38836626	G	C	HTRA4	intronic	NA	NA	420	181	601	0.301165	28	0	28	0	1	Sub	0.72	3.1			polymorphism	3.416						

Sample	Type	Chr	Start	End	Ref	Alt	Gene	unc.ensGe	icFunc.ens	hange.ens(	t_ref	t_alt	t_total	t_VAF	n_ref	n_alt	n_total	hormal_VA	ncer_cell_f	on-based C	purity	ploidy	Cancer Gene	Driver Gene	Mutation Taster	ADD(PHRE	otein_chan	CHASM	ATHMM_Pr	OLYPHEN-	REDICTION	ICTION (cutoff=0.05)
OVA_013_C2	PRIMARY	chr8	38836626	38836626	G	C	HTRA4	intronic	NA	NA	425	172	597	0.288107	28	0	28	0	0.95	Sub	0.87	3.05			polymorphism	3.416						
OVA_013_C3	PRIMARY	chr8	38836626	38836626	G	C	HTRA4	intronic	NA	NA	445	165	610	0.270492	28	0	28	0	0.59	Sub	0.91	3.01			polymorphism	3.416						
OVA_003_M1	METASTASIS	chr19	38916619	38916619	C	A	RASGRP4	intronic	NA	NA	87	35	122	0.286885	32	0	32	0	1	Sub	0.57	1.84			polymorphism	5.117						
OVA_003_M2	METASTASIS	chr19	38916619	38916619	C	A	RASGRP4	intronic	NA	NA	123	26	149	0.174497	32	0	32	0	0.76	Sub	0.46	1.81			polymorphism	5.117						
OVA_003_M3	METASTASIS	chr19	38916619	38916619	C	A	RASGRP4	intronic	NA	NA	169	19	188	0.101064	32	0	32	0	1	Sub	0.34	2.67			polymorphism	5.117						
OVA_003_C1	PRIMARY	chr19	38916619	38916619	C	A	RASGRP4	intronic	NA	NA	119	90	209	0.430622	32	0	32	0	1	Clonal	0.86	1.81			polymorphism	5.117						
OVA_003_C2	PRIMARY	chr19	38916619	38916619	C	A	RASGRP4	intronic	NA	NA	146	83	229	0.362445	32	0	32	0	1	Clonal	0.7	3.61			polymorphism	5.117						
OVA_003_C3	PRIMARY	chr19	38916619	38916619	C	A	RASGRP4	intronic	NA	NA	140	101	241	0.419087	32	0	32	0	1	Clonal	0.82	1.81			polymorphism	5.117						
OVA_378_M1	METASTASIS	chr19	38993515	38993515	C	T	RYR1	intronic	NA	NA	340	147	487	0.301848	56	0	56	0	1	Clonal	0.72	2.57			polymorphism	6.024						
OVA_378_M2	METASTASIS	chr19	38993515	38993515	C	T	RYR1	intronic	NA	NA	295	138	433	0.318707	56	0	56	0	1	Clonal	0.85	2.57			polymorphism	6.024						
OVA_378_M3	METASTASIS	chr19	38993515	38993515	C	T	RYR1	intronic	NA	NA	271	94	365	0.257534	56	0	56	0	1	Clonal	0.67	2.57			polymorphism	6.024						
OVA_378_C1	PRIMARY	chr19	38993515	38993515	C	T	RYR1	intronic	NA	NA	357	123	480	0.25625	56	0	56	0	1	Clonal	0.59	2.6			polymorphism	6.024						
OVA_378_C2	PRIMARY	chr19	38993515	38993515	C	T	RYR1	intronic	NA	NA	304	88	392	0.22449	56	0	56	0	1	Clonal	0.56	2.59			polymorphism	6.024						
OVA_378_C3	PRIMARY	chr19	38993515	38993515	C	T	RYR1	intronic	NA	NA	295	72	367	0.196185	56	0	56	0	0.97	Clonal	0.51	2.6			polymorphism	6.024						
OVA_048_C1	PRIMARY	chr3	39107378	39107378	C	T	WDR48	exonic	ynonymou:	p.T89I,ENS(	199	209	408	0.512255	67	0	67	0	1	Clonal	0.67	1.82			disease_causing	24.2	p.T89I	Passenger	SENGER/OT	benign	Neutral	Tolerated
OVA_048_C2	PRIMARY	chr3	39107378	39107378	C	T	WDR48	exonic	ynonymou:	p.T89I,ENS(	191	191	382	0.5	67	0	67	0	1	Clonal	0.72	1.85			disease_causing	24.2	p.T89I	Passenger	SENGER/OT	benign	Neutral	Tolerated
OVA_048_C3	PRIMARY	chr3	39107378	39107378	C	T	WDR48	exonic	ynonymou:	p.T89I,ENS(	211	241	452	0.533186	67	0	67	0	1	Clonal	0.74	1.8			disease_causing	24.2	p.T89I	Passenger	SENGER/OT	benign	Neutral	Tolerated
OVA_048_M1	METASTASIS	chr19	39108497	39108497	G	T	MAP4K1	exonic	ionymous S	).P13P,ENS(	63	45	108	0.416667	41	0	41	0	1	Clonal	0.56	3.94			disease_causing	16.69		Passenger			Neutral	Tolerated
OVA_048_M2	METASTASIS	chr19	39108497	39108497	G	T	MAP4K1	exonic	ionymous S	).P13P,ENS(	76	39	115	0.33913	41	0	41	0	1	Clonal	0.4	3.87			disease_causing	16.69		Passenger			Neutral	Tolerated
OVA_048_M3	METASTASIS	chr19	39108497	39108497	G	T	MAP4K1	exonic	ionymous S	).P13P,ENS(	55	51	106	0.481132	41	0	41	0	1	Clonal	0.59	3.39			disease_causing	16.69		Passenger			Neutral	Tolerated
OVA_048_C1	PRIMARY	chr19	39108497	39108497	G	T	MAP4K1	exonic	ionymous S	).P13P,ENS(	44	29	73	0.39726	41	0	41	0	1	Sub	0.67	1.82			disease_causing	16.69		Passenger			Neutral	Tolerated
OVA_048_C2	PRIMARY	chr19	39108497	39108497	G	T	MAP4K1	exonic	ionymous S	).P13P,ENS(	63	46	109	0.422018	41	0	41	0	1	Sub	0.72	1.85			disease_causing	16.69		Passenger			Neutral	Tolerated
OVA_048_C3	PRIMARY	chr19	39108497	39108497	G	T	MAP4K1	exonic	ionymous S	).P13P,ENS(	55	18	73	0.246575	41	0	41	0	0.67	Sub	0.74	1.8			disease_causing	16.69		Passenger			Neutral	Tolerated
OVA_047_C1	PRIMARY	chr5	39122364	39122364	G	C	FYB	intronic	NA	NA	522	209	731	0.28591	23	0	23	0	1	Sub	0.6	2.91			polymorphism	0.9						
OVA_047_C2	PRIMARY	chr5	39122364	39122364	G	C	FYB	intronic	NA	NA	595	90	685	0.131387	23	0	23	0	0.54	Sub	0.64	2.76			polymorphism	0.9						
OVA_047_C3	Contralateral ovary	chr5	39122364	39122364	G	C	FYB	intronic	NA	NA	560	174	734	0.237057	23	0	23	0	1	Clonal	0.6	2.94			polymorphism	0.9						
OVA_048_M1	METASTASIS	chr3	39148673	39148673	C	A	GORASP1	UTR5	NA	NA	92	74	166	0.445783	79	0	79	0	1	Clonal	0.56	3.94			polymorphism	4.13						
OVA_048_M2	METASTASIS	chr3	39148673	39148673	C	A	GORASP1	UTR5	NA	NA	125	45	170	0.264706	79	0	79	0	1	Clonal	0.4	3.87			polymorphism	4.13						
OVA_048_M3	METASTASIS	chr3	39148673	39148673	C	A	GORASP1	UTR5	NA	NA	138	99	237	0.417722	79	0	79	0	1	Clonal	0.59	3.39			polymorphism	4.13						
OVA_048_C1	PRIMARY	chr3	39148673	39148673	C	A	GORASP1	UTR5	NA	NA	84	39	123	0.317073	79	0	79	0	0.95	Clonal	0.67	1.82			polymorphism	4.13						
OVA_048_C2	PRIMARY	chr3	39148673	39148673	C	A	GORASP1	UTR5	NA	NA	155	71	226	0.314159	79	0	79	0	0.87	Clonal	0.72	1.85			polymorphism	4.13						
OVA_048_C3	PRIMARY	chr3	39148673	39148673	C	A	GORASP1	UTR5	NA	NA	95	41	136	0.301471	79	0	79	0	0.81	Clonal	0.74	1.8			polymorphism	4.13						
OVA_048_M1	METASTASIS	chr6	39283130	39283130	C	T	KCNK16	exonic	ynonymou:	000042505	441	29	470	0.061702	37	0	37	0	0.47	Sub	0.56	3.94			polymorphism	16.85	p.E247K	Passenger	SENGER/OT	damaging	Neutral	Damaging
OVA_013_M1	METASTASIS	chr3	39452702	39452702	C	A	RPSA	intronic	NA	NA	474	163	637	0.255887	50	0	50	0	1	Clonal	0.67	3.06			polymorphism	1.712						
OVA_048_M1	METASTASIS	chr2	39535093	39535093	T	G	MAP4K3	exonic	ynonymou:	140S,ENSG(	284	164	448	0.366071	50	0	50	0	1	Clonal	0.56	3.94			disease_causing	22.5	p.R370S	Passenger	SENGER/OT	benign	Neutral	Tolerated
OVA_048_M2	METASTASIS	chr2	39535093	39535093	T	G	MAP4K3	exonic	ynonymou:	140S,ENSG(	334	131	465	0.28172	50	0	50	0	1	Clonal	0.4	3.87			disease_causing	22.5	p.R370S	Passenger	SENGER/OT	benign	Neutral	Tolerated
OVA_048_M3	METASTASIS	chr2	39535093	39535093	T	G	MAP4K3	exonic	ynonymou:	140S,ENSG(	270	190	460	0.413043	50	0	50	0	1	Clonal	0.59	3.39			disease_causing	22.5	p.R370S	Passenger	SENGER/OT	benign	Neutral	Tolerated
OVA_365_M1	METASTASIS	chr8	39618697	39618697	A	G	ADAM2	exonic	ionymous S	000034758(	302	228	530	0.430189	40	0	40	0	1	Clonal	0.85	1.87			polymorphism	3.646		Passenger			Neutral	Tolerated
OVA_365_M2	METASTASIS	chr8	39618697	39618697	A	G	ADAM2	exonic	ionymous S	000034758(	299	88	387	0.22739	40	0	40	0	0.9	Clonal	0.4	1.81			polymorphism	3.646		Passenger			Neutral	Tolerated
OVA_365_M3	METASTASIS	chr8	39618697	39618697	A	G	ADAM2	exonic	ionymous S	000034758(	288	224	512	0.4375	40	0	40	0	1	Clonal	0.82	1.89			polymorphism	3.646		Passenger			Neutral	Tolerated
OVA_365_C1	Contralateral ovary	chr8	39618697	39618697	A	G	ADAM2	exonic	ionymous S	000034758(	306	72	378	0.190476	40	0	40	0	0.91	Clonal	0.42	1.83			polymorphism	3.646		Passenger			Neutral	Tolerated
OVA_365_C2	PRIMARY	chr8	39618697	39618697	A	G	ADAM2	exonic	ionymous S	000034758(	357	137	494	0.277328	40	0	40	0	0.9	Clonal	0.62	1.92			polymorphism	3.646		Passenger			Neutral	Tolerated
OVA_048_M1	METASTASIS	chr12	39719694	39719694	T	G	KIF21A	exonic	ynonymou:	15P,ENSG0	71	87	158	0.550633	32	0	32	0	1	Clonal	0.56	3.94			polymorphism	18.94	p.T1135P	Passenger	SENGER/OT	benign	Neutral	Damaging
OVA_048_M2	METASTASIS	chr12	39719694	39719694	T	G	KIF21A	exonic	ynonymou:	15P,ENSG0	155	101	256	0.394531	32	0	32	0	1	Clonal	0.4	3.87			polymorphism	18.94	p.T1135P	Passenger	SENGER/OT	benign	Neutral	Damaging
OVA_048_M3	METASTASIS	chr12	39719694	39719694	T	G	KIF21A	exonic	ynonymou:	15P,ENSG0	99	118	217	0.543779	32	0	32	0	1	Clonal	0.59	3.39			polymorphism	18.94	p.T1135P	Passenger	SENGER/OT	benign	Neutral	Damaging
OVA_013_M1	METASTASIS	chr20	39741530	39741530	C	T	TOP1	exonic	ynonymou:	000361337	312	293	605	0.484298	46	0	46	0	1	Clonal	0.67	3.06	Yes	No	disease_causing	32	p.R473W	Driver	CANCER	damaging	Deleterious	Damaging
OVA_013_C1	PRIMARY	chr20	39741530	39741530	C	T	TOP1	exonic	ynonymou:	000361337	264	353	617	0.572123	46	0	46	0	1	Clonal	0.72	3.1	Yes	No	disease_causing	32	p.R473W	Driver	CANCER	damaging	Deleterious	Damaging
OVA_013_C2	PRIMARY	chr20	39741530	39741530	C	T	TOP1	exonic	ynonymou:	000361337	257	350	607	0.576606	46	0	46	0	1	Clonal	0.87	3.05	Yes	No	disease_causing	32	p.R473W	Driver	CANCER	damaging	Deleterious	Damaging
OVA_013_C3	PRIMARY	chr20	39741530	39741530	C	T	TOP1	exonic	ynonymou:	000361337	262	388	650	0.596923	46	0	46	0	1	Clonal	0.91	3.01	Yes	No	disease_causing	32	p.R473W	Driver	CANCER	damaging	Deleterious	Damaging
OVA_365_C1	Contralateral ovary	chr4	39757267	39757267	T	C	UBE2K	intronic	NA	NA	270	81	351	0.230769	25	0	25	0	1	Clonal	0.42	1.83			disease_causing	22.5						
OVA_048_M1	METASTASIS	chr17	39882046	39882046	G	A	HAP1;JUP	intronic	NA	NA	98	73	171	0.426901	8	0	8	0	1	Clonal	0.56	3.94			polymorphism	3.11						
OVA_048_M2	METASTASIS	chr17	39882046	39882046	G	A	HAP1;JUP	intronic	NA	NA	129	68	197	0.345178	8	0	8	0	1	Clonal	0.4	3.87			polymorphism	3.11						
OVA_048_M3	METASTASIS	chr17	39882046	39882046	G	A	HAP1;JUP	intronic	NA	NA	98	71	169	0.420118	8	0	8	0	1	Clonal	0.59	3.39			polymorphism	3.11						
OVA_047_C1	PRIMARY	chrX	39931831	39931831	G	C	BCOR	exonic	ynonymou:	000037845	452	146	598	0.244147	74	0	74	0	0.81	Sub	0.6	2.91	Yes	Yes	disease_causing	23.4	p.P923R	Passenger	SENGER/OT	damaging	Deleterious	Damaging
OVA_047_C2	PRIMARY	chrX	39931831	39931831	G	C	BCOR	exonic	ynonymou:	000037845	445	134	579	0.231434	74	0	74	0	0.72	Sub	0.64	2.76	Yes	Yes	disease_causing	23.4	p.P923R	Passenger	SENGER/OT	damaging	Deleterious	Damaging
OVA_047_C3	Contralateral ovary	chrX	39931831	39931831	G	C	BCOR	exonic	ynonymou:	000037845	422	121	543	0.222836	74	0	74	0	0.75	Sub	0.6	2.94	Yes	Yes	disease_causing	23.4	p.P923R	Passenger	SENGER/OT	damaging	Deleterious	Damaging
OVA_365_M2	METASTASIS	chr22	40068179	40068179	C	G	CACNA1I	intronic	NA	NA	407	35	442	0.079186	57	0	57	0	0.31	Sub	0.4	1.81			disease_causing	14.26						
OVA_013_M1	METASTASIS	chr4	40440634	40440634	C	T	RBM47	exonic	ynonymou:	A93T,ENSG	435	112	547	0.204753	86	0	86	0	1	Clonal	0.67	3.06			polymorphism	0.023	p.A93T	Passenger	SENGER/OT	benign	Neutral	Tolerated
OVA_013_C1	PRIMARY	chr4	40440634	40440634	C	T	RBM47	exonic	ynonymou:	A93T,ENSG	424	123	547	0.224863	86	0	86	0	1	Clonal	0.72	3.1			polymorphism	0.023	p.A93T	Passenger	SENGER/OT	benign	Neutral	Tolerated
OVA_013_C2	PRIMARY	chr4	40440634	40440634	C	T	RBM47	exonic	ynonymou:	A93T,ENSG	396	136	532	0.255639	86	0	86	0	1	Clonal	0.87	3.05			polymorphism	0.023	p.A93T	Passenger	SENGER/OT	benign	Neutral	Tolerated
OVA_013_C3	PRIMARY	chr4	40440634	40440634	C	T	RBM47	exonic	ynonymou:	A93T,ENSG	390	168	558	0.301075	86	0	86	0	0.96	Clonal	0.91	3.01			polymorphism	0.023	p.A93T	Passenger	SENGER/OT	benign	Neutral	Tolerated
OVA_378_M2	METASTASIS	chr3	40557311	40557312	GA	-	ZNF620	intronic	NA	NA	277	187	464	0.403017	27	0	27		0.94	Clonal	0.85	2.57										
OVA_378_C2	PRIMARY	chr3	40557311	40557312	GA	-	ZNF620	intronic	NA	NA	283	154	437	0.352403	27	0	27		1	Clonal	0.56	2.59										
OVA_378_C3	PRIMARY	chr3	40557311	40557312	GA	-	ZNF620	intronic	NA	NA	273	203	476	0.426471	27	0	27		1	Clonal	0.51	2.6										
OVA_047_C1	PRIMARY	chr15	40705142	40705142	C	T	IVD	intronic	NA	NA	407	41	448	0.091518	38	0	38	0	0.31	Sub	0.6	2.91			polymorphism	1.828						
OVA_048_C1	PRIMARY	chr12	40826344	40826344	T	A	UC19;MUC	RNA_intro	NA	NA	21	32	53	0.603774	8	0	8	0	1	Clonal	0.67	1.82				5.613						
OVA_048_C2	PRIMARY	chr12	40826344	40826344	T	A	UC19;MUC	RNA_intro	NA	NA	20	45	65	0.692308	8	0	8	0	1	Clonal	0.72	1.85				5.613						
OVA_048_C3	PRIMARY	chr12	40826344	40826344	T	A	UC19;MUC	RNA_intro	NA	NA	29	35	64	0.546875	8	0	8	0	0.93	Clonal	0.74	1.8				5.613						
OVA_003_M1	METASTASIS	chr12	40962849	40962849	G	A	IUC19;CNTI	intronic	NA	NA	855	11	866	0.012702	29	0	29	0	0.05	Sub	0.57	1.84			disease_causing	16.14						
OVA_003_C1	PRIMARY	chr12	40962849	40962849	G	A	IUC19;CNTI	intronic	NA	NA	294	181	475	0.381053	29	0	29	0	0.88	Sub	0.86	1.81			disease_causing	16.14						
OVA_003_C2	PRIMARY	chr12	40962849	40962849	G	A	IUC19;CNTI	intronic	NA	NA	398	103	501	0.205589	29	0	29	0	1	Sub	0.7	3.61			disease_causing	16.14						
OVA_003_C3	PRIMARY	chr12	40962849	40962849	G	A	IUC19;CNTI	intronic	NA	NA	364	186	550	0.338182	29	0	29	0	0.82	Sub	0.82	1.81			disease_causing	16.14						
OVA_047_C1	PRIMARY	chr17	40991294	40991294	C	T	PSME3	intronic	NA	NA	515	141	656	0.214939	71	0	71	0	0.72	Sub	0.6	2.91			polymorphism	6.298						
OVA_047_C1	PRIMARY	chr19	40998803	40998803	G	C	SPTBN4	intronic	NA	NA	220	71	291	0.243986	16	0	16	0	1	Clonal	0.6	2.91			polymorphism	7.567						
OVA_047_C2	PRIMARY	chr19	40998803	40998803	G	C	SPTBN4	intronic	NA	NA	227	68	295	0.230508	16	0	16	0	0.95	Clonal	0.64	2.76			polymorphism	7.567						
OVA_047_C3	Contralateral ovary	chr19	40998803	40998803	G	C	SPTBN4	intronic	NA	NA	191	54	245	0.220408	16	0	16	0	0.96	Clonal	0.6	2.94			polymorphism	7.567						
OVA_048_M1	METASTASIS	chr19	41099045	41099045	C	T	LTBP4	upstream	NA	NA	294	194	488	0.397541	33	1	34	0.029412	1	Clonal	0.56	3.94				0.488						
OVA_048_M2	METASTASIS	chr19	41099045	41099045	C	T	LTBP4	upstream	NA	NA	358	145	503	0.28827	33	1	34	0.029412	1	Clonal	0.4	3.87				0.488						
OVA_048_M3	METASTASIS	chr19	41099045	41099045	C	T	LTBP4	upstream	NA	NA	330	107	437	0.244851	33	1	34	0.029412	1	Clonal	0.59	3.39				0.488						
OVA_048_C1	PRIMARY	chr19	41099045	41099045	C	T	LTBP4	upstream	NA	NA	177	93	270	0.344444	33	1	34	0.029412	1	Clonal	0.67	1.82				0.488						
OVA_048_C2	PRIMARY	chr19	41099045	41099045	C	T	LTBP4	upstream	NA	NA	298	196	494	0.396761	33	1	34	0.029412	1	Clonal	0.72	1.85				0.488						
OVA_048_C3	PRIMARY	chr19	41099045	41099045	C	T	LTBP4	upstream	NA	NA	200	117	317	0.369085	33	1	34	0.029412	1	Clonal	0.74	1.8				0.488						
OVA_048_M1	METASTASIS	chr19	41198202	41198202	C	A	ADCK4	exonic	ynonymou:	000450541	45	21	66	0.318182	45	0	45	0	1	Clonal	0.56	3.94			disease_causing	28.5	p.G458V	Passenger	SENGER/OT	damaging	Deleterious	Damaging
OVA_048_M2	METASTASIS	chr19	41198202	41198202	C	A	ADCK4	exonic	ynonymou:	000450541	27	13	40	0.325	45	0	45	0	1	Clonal	0.4	3.87			disease_causing	28.5	p.G458V	Passenger	SENGER/OT	damaging	Deleterious	Damaging
OVA_048_M3	METASTASIS	chr19	41198202	41198202	C	A	ADCK4	exonic	ynonymou:	000450541	30	26	56	0.464286	45	0	45	0	1	Clonal	0.59	3.39			disease_causing	28.5	p.G458V	Passenger	SENGER/OT	damaging	Deleterious	Damaging
OVA_047_C1	PRIMARY	chr15	41245706	41245706	C	A	CHAC1	exonic	stopgain	.C17X,ENSG	110	34	144	0.236111	59	0	59	0	0.79	Sub	0.6	2.91			disease_causing_automat	36	p.C17X	Passenger			NA	NA
OVA_047_C2	PRIMARY	chr15	41245706	41245706	C	A	CHAC1	exonic	stopgain	.C17X,ENSG	97	53	150	0.353333	59	0	59	0	1	Sub	0.64	2.76			disease_causing_automat	36	p.C17X	Passenger			NA	NA
OVA_047_C3	Contralateral ovary	chr15	41245706	41245706	C	A	CHAC1	exonic	stopgain	.C17X,ENSG	86	48	134	0.358209	59	0	59	0	1	Clonal	0.6	2.94			disease_causing_automat	36	p.C17X	Passenger			NA	NA
OVA_003_M1	METASTASIS	chr20	41419958	41419958	G	C	PTPRT	exonic	ynonymou:	000037319	340	31	371	0.083558	68	0	68	0	0.3	Sub	0.57	1.84	Yes	Yes	disease_causing	23.2	p.N121K	Passenger	SENGER/OT	damaging	Neutral	Tolerated
OVA_003_M2	METASTASIS	chr20	41419958	41419958	G	C	PTPRT	exonic	ynonymou:	000037319	408	26	434	0.059908	68	0	68	0	0.26	Sub	0.46	1.81	Yes	Yes	disease_causing	23.2	p.N121K	Passenger	SENGER/OT	damaging	Neutral	Tolerated
OVA_003_M3	METASTASIS	chr20	41419958	41419958	G	C	PTPRT	exonic	ynonymou:	000037319	482	13	495	0.026263	68	0	68	0	0.28	Sub	0.34	2.67	Yes	Yes	disease_causing	23.2	p.N121K	Passenger	SENGER/OT	damaging	Neutral	Tolerated
OVA_003_M1	METASTASIS	chr17	41606547	41606547	T	A	ETV4	exonic	ynonymou:	00538265:	143	204	347	0.587896	55	0	55	0	1	Clonal	0.57	1.84	Yes	No	disease_causing	24.7	p.S396C	Passenger	SENGER/OT	damaging	Deleterious	Damaging
OVA_003_M2	METASTASIS	chr17	41606547	41606547	T	A	ETV4	exonic	ynonymou:	00538265:	267	179	446	0.401345	55	0	55	0	1	Clonal	0.46	1.81	Yes	No	disease_causing	24.7	p.S396C	Passenger	SENGER/OT	damaging	Deleterious	Damaging
OVA_003_M3	METASTASIS	chr17	41606547	41606547	T	A	ETV4	exonic	ynonymou:	00538265:	422	52	474	0.109705	55	0	55	0	0.94	Clonal	0.34	2.67	Yes	No	disease_causing	24.7	p.S396C	Passenger	SENGER/OT	damaging	Deleterious	Damaging
OVA_003_C1	PRIMARY	chr17	41606547	41606547	T	A	ETV4	exonic	ynonymou:	00538265:	50	292	342	0.853801	55	0	55	0	1	Clonal	0.86	1.81	Yes	No	disease_causing	24.7	p.S396C	Passenger	SENGER/OT	damaging	Deleterious	Damaging
OVA_003_C2	PRIMARY	chr17	41606547	41606547	T	A	ETV4	exonic	ynonymou:	00538265:	83	273	356	0.766854	55	0	55	0	1	Clonal	0.7	3.61	Yes	No	disease_causing	24.7	p.S396C	Passenger	SENGER/OT	damaging	Deleterious	Damaging
OVA_003_C3	PRIMARY	chr17	41606547	41606547	T	A	ETV4	exonic	ynonymou:	00538265:	97	303	400	0.7575	55	0	55	0	1	Clonal	0.82	1.81	Yes	No	disease_causing	24.7	p.S396C	Passenger	SENGER/OT	damaging	Deleterious	Damaging
OVA_048_M1	METASTASIS	chr6	41712382	41712382	G	T	PGC	intronic	NA	NA	282	167	449	0.371938	42	1	43	0.023256	1	Clonal	0.56	3.94			polymorphism	2.044						
OVA_048_M2	METASTASIS	chr6	41712382	41712382	G	T	PGC	intronic	NA	NA	349	125	474	0.263713	42	1	43	0.023256	1	Clonal	0.4	3.87			polymorphism	2.044						
OVA_048_M3	METASTASIS	chr6	41712382	41712382	G	T	PGC	intronic	NA	NA	330	107	437	0.244851	42	1	43	0.023256	1	Clonal	0.59	3.39			polymorphism	2.044						

Sample	Type	Chr	Start	End	Ref	Alt	Gene	unc.ensGer	icFunc.ens	hange.ens	t_ref	t_alt	t_total	t_VAF	n_ref	n_alt	n_total	hormal_VA	ncer_cell_f	on-based C	purity	ploidy	Cancer Gene	Driver Gene	Mutation Taster	ADD(PHRE	otein_chan	CHASM	ATHMM_Pr	OLYPHEN-	REDICTION	ICTION (cutoff=0.05)
OVA_048_C1	PRIMARY	chr6	41712382	41712382	G	T	PGC	intronic	NA	NA	92	60	152	0.394737	42	1	43	0.023256	1	Clonal	0.67	1.82			polymorphism	2.044						
OVA_048_C2	PRIMARY	chr6	41712382	41712382	G	T	PGC	intronic	NA	NA	191	225	416	0.540865	42	1	43	0.023256	1	Clonal	0.72	1.85			polymorphism	2.044						
OVA_048_C3	PRIMARY	chr6	41712382	41712382	G	T	PGC	intronic	NA	NA	123	100	223	0.44843	42	1	43	0.023256	1	Clonal	0.74	1.8			polymorphism	2.044						
OVA_048_M1	METASTASIS	chr8	41792396	41792396	A	T	KAT6A	intronic	NA	NA	246	150	396	0.378788	19	0	19	0	1	Clonal	0.56	3.94	Yes		polymorphism	7.043						
OVA_048_M2	METASTASIS	chr8	41792396	41792396	A	T	KAT6A	intronic	NA	NA	309	144	453	0.317881	19	0	19	0	1	Clonal	0.4	3.87	Yes		polymorphism	7.043						
OVA_048_M3	METASTASIS	chr8	41792396	41792396	A	T	KAT6A	intronic	NA	NA	162	163	325	0.501538	19	0	19	0	1	Clonal	0.59	3.39	Yes		polymorphism	7.043						
OVA_048_C1	PRIMARY	chr8	41792396	41792396	A	T	KAT6A	intronic	NA	NA	151	78	229	0.340611	19	0	19	0	1	Clonal	0.67	1.82	Yes		polymorphism	7.043						
OVA_048_C2	PRIMARY	chr8	41792396	41792396	A	T	KAT6A	intronic	NA	NA	249	144	393	0.366412	19	0	19	0	1	Clonal	0.72	1.85	Yes		polymorphism	7.043						
OVA_048_C3	PRIMARY	chr8	41792396	41792396	A	T	KAT6A	intronic	NA	NA	190	102	292	0.349315	19	0	19	0	0.94	Clonal	0.74	1.8	Yes		polymorphism	7.043						
OVA_003_M1	METASTASIS	chr19	41930436	41930436	C	T	BCKDHA	exonic	ynonymou	424C,ENSG	255	112	367	0.305177	117	0	117	0	1	Sub	0.57	1.84			disease_causing	29.6	p.R455C	Passenger	SENGER/OT	benign	Deleterious	Damaging
OVA_003_M2	METASTASIS	chr19	41930436	41930436	C	T	BCKDHA	exonic	ynonymou	424C,ENSG	338	103	441	0.23356	117	0	117	0	1	Sub	0.46	1.81			disease_causing	29.6	p.R455C	Passenger	SENGER/OT	benign	Deleterious	Damaging
OVA_003_M3	METASTASIS	chr19	41930436	41930436	C	T	BCKDHA	exonic	ynonymou	424C,ENSG	412	33	445	0.074157	117	0	117	0	0.78	Sub	0.34	2.67			disease_causing	29.6	p.R455C	Passenger	SENGER/OT	benign	Deleterious	Damaging
OVA_003_C1	PRIMARY	chr19	41930436	41930436	C	T	BCKDHA	exonic	ynonymou	424C,ENSG	251	239	490	0.487755	117	0	117	0	1	Clonal	0.86	1.81			disease_causing	29.6	p.R455C	Passenger	SENGER/OT	benign	Deleterious	Damaging
OVA_003_C2	PRIMARY	chr19	41930436	41930436	C	T	BCKDHA	exonic	ynonymou	424C,ENSG	279	220	499	0.440882	117	0	117	0	1	Clonal	0.7	3.61			disease_causing	29.6	p.R455C	Passenger	SENGER/OT	benign	Deleterious	Damaging
OVA_003_C3	PRIMARY	chr19	41930436	41930436	C	T	BCKDHA	exonic	ynonymou	424C,ENSG	294	218	512	0.425781	117	0	117	0	1	Clonal	0.82	1.81			disease_causing	29.6	p.R455C	Passenger	SENGER/OT	benign	Deleterious	Damaging
OVA_048_C1	PRIMARY	chr4	42062250	42062250	G	C	SLC30A9	exonic	ynonymou	000026445	247	174	421	0.413302	15	0	15	0	1	Clonal	0.67	1.82			disease_causing	32	p.D295H	Passenger	SENGER/OT	damaging	Deleterious	Damaging
OVA_048_C2	PRIMARY	chr4	42062250	42062250	G	C	SLC30A9	exonic	ynonymou	000026445	350	221	571	0.38704	15	0	15	0	1	Clonal	0.72	1.85			disease_causing	32	p.D295H	Passenger	SENGER/OT	damaging	Deleterious	Damaging
OVA_048_C3	PRIMARY	chr4	42062250	42062250	G	C	SLC30A9	exonic	ynonymou	000026445	266	216	482	0.448133	15	0	15	0	1	Clonal	0.74	1.8			disease_causing	32	p.D295H	Passenger	SENGER/OT	damaging	Deleterious	Damaging
OVA_047_C1	PRIMARY	chr22	42099350	42099350	C	T	MEI1	exonic	ynonymou	000004015	443	209	652	0.320552	21	0	21	0	1	Clonal	0.6	2.91			disease_causing	22.1	p.H64Y	Passenger	SENGER/OT	benign	Neutral	Damaging
OVA_047_C2	PRIMARY	chr22	42099350	42099350	C	T	MEI1	exonic	ynonymou	000004015	439	200	639	0.312989	21	0	21	0	0.98	Clonal	0.64	2.76			disease_causing	22.1	p.H64Y	Passenger	SENGER/OT	benign	Neutral	Damaging
OVA_047_C3	Contralateral ovary	chr22	42099350	42099350	C	T	MEI1	exonic	ynonymou	000004015	430	251	681	0.368576	21	0	21	0	1	Clonal	0.6	2.94			disease_causing	22.1	p.H64Y	Passenger	SENGER/OT	benign	Neutral	Damaging
OVA_047_C2	PRIMARY	chr6	42146427	42146427	A	G	GUCA1A	intronic	NA	NA	480	94	574	0.163763	48	0	48	0	0.84	Sub	0.64	2.76			polymorphism	3.275						
OVA_048_C1	PRIMARY	chr19	42406499	42406506	TGGCCCGG	-	ARHGEF1	exonic	eshift dele	0337665:ex	74	24	98	0.244898	31	1	32		0.73	Sub	0.67	1.82					54_R555de	Passenger			NA	NA
OVA_048_C2	PRIMARY	chr19	42406499	42406506	TGGCCCGG	-	ARHGEF1	exonic	eshift dele	0337665:ex	141	56	197	0.284264	31	1	32		0.79	Sub	0.72	1.85					54_R555de	Passenger			NA	NA
OVA_048_C3	PRIMARY	chr19	42406499	42406506	TGGCCCGG	-	ARHGEF1	exonic	eshift dele	0337665:ex	95	30	125	0.24	31	1	32		0.65	Sub	0.74	1.8					54_R555de	Passenger			NA	NA
OVA_013_M1	METASTASIS	chr4	42592858	42592858	A	C	ATP8A1	exonic	ynonymou	000038166	449	117	566	0.206714	36	0	36	0	1	Clonal	0.67	3.06			disease_causing	28.5	p.L165R	Passenger	SENGER/OT	damaging	Deleterious	Damaging
OVA_003_C1	PRIMARY	chrX	42637071	42637071	C	T	PPP1R2P9	cRNA_exon	NA	NA	413	235	648	0.362654	98	0	98	0	0.84	Sub	0.86	1.81				0.72				damaging		
OVA_003_C2	PRIMARY	chrX	42637071	42637071	C	T	PPP1R2P9	cRNA_exon	NA	NA	506	131	637	0.205651	98	0	98	0	1	Sub	0.7	3.61				0.72				damaging		
OVA_003_C3	PRIMARY	chrX	42637071	42637071	C	T	PPP1R2P9	cRNA_exon	NA	NA	452	220	672	0.327381	98	0	98	0	0.79	Sub	0.82	1.81				0.72				damaging		
OVA_365_M1	METASTASIS	chr3	42734629	42734629	G	C	HHATL	exonic	ynonymou	444V,ENSG	225	37	262	0.141221	92	0	92	0	0.33	Sub	0.85	1.87			polymorphism	14.72	p.L444V	Passenger	SENGER/OT	benign	Neutral	Tolerated
OVA_365_M1	METASTASIS	chr22	42828396	42828396	G	C	NFAM1	UTR5	NA	NA	8	23	31	0.741935	52	0	52	0	1	Clonal	0.85	1.87			disease_causing	13.21						
OVA_365_M2	METASTASIS	chr22	42828396	42828396	G	C	NFAM1	UTR5	NA	NA	11	4	15	0.266667	52	0	52	0	1	Clonal	0.4	1.81			disease_causing	13.21						
OVA_365_M3	METASTASIS	chr22	42828396	42828396	G	C	NFAM1	UTR5	NA	NA	6	23	29	0.793103	52	0	52	0	1	Clonal	0.82	1.89			disease_causing	13.21						
OVA_047_C1	PRIMARY	chr1	42880521	42880521	G	A	RIMKLA	exonic	ynonymou	000043147	424	187	611	0.306056	50	0	50	0	1	Clonal	0.6	2.91			disease_causing	23.8	p.S351N	Passenger	no weights	damaging	Neutral	Damaging
OVA_047_C2	PRIMARY	chr1	42880521	42880521	G	A	RIMKLA	exonic	ynonymou	000043147	409	198	607	0.326194	50	0	50	0	1	Clonal	0.64	2.76			disease_causing	23.8	p.S351N	Passenger	no weights	damaging	Neutral	Damaging
OVA_047_C3	Contralateral ovary	chr1	42880521	42880521	G	A	RIMKLA	exonic	ynonymou	000043147	416	170	586	0.290102	50	0	50	0	0.97	Clonal	0.6	2.94			disease_causing	23.8	p.S351N	Passenger	no weights	damaging	Neutral	Damaging
OVA_013_M1	METASTASIS	chr20	42891895	42891895	C	T	GDAP1L1	exonic	onymous S	133D,ENSG	197	57	254	0.224409	39	0	39	0	0.89	Clonal	0.67	3.06			disease_causing	10.35		Passenger			Neutral	Tolerated
OVA_013_C1	PRIMARY	chr20	42891895	42891895	C	T	GDAP1L1	exonic	onymous S	133D,ENSG	299	89	388	0.229381	39	0	39	0	0.87	Sub	0.72	3.1			disease_causing	10.35		Passenger			Neutral	Tolerated
OVA_013_C2	PRIMARY	chr20	42891895	42891895	C	T	GDAP1L1	exonic	onymous S	133D,ENSG	263	106	369	0.287263	39	0	39	0	0.95	Sub	0.87	3.05			disease_causing	10.35		Passenger			Neutral	Tolerated
OVA_013_C3	PRIMARY	chr20	42891895	42891895	C	T	GDAP1L1	exonic	onymous S	133D,ENSG	292	107	399	0.26817	39	0	39	0	0.85	Sub	0.91	3.01			disease_causing	10.35		Passenger			Neutral	Tolerated
OVA_048_M3	METASTASIS	chr22	43015885	43015885	T	C	CYB5R3	exonic	ynonymou	300G,ENSG	310	33	343	0.09621	77	0	77	0	0.85	Clonal	0.59	3.39			polymorphism	22.5	p.E300G	Passenger	SENGER/OT	benign	Neutral	Tolerated
OVA_013_M1	METASTASIS	chr6	43020299	43020299	G	A	CUL7	exonic	onymous S	A76A,ENSG	431	173	604	0.286424	69	1	70	0.014286	1	Clonal	0.67	3.06			disease_causing	22.6		Passenger			Neutral	Tolerated
OVA_013_C1	PRIMARY	chr6	43020299	43020299	G	A	CUL7	exonic	onymous S	A76A,ENSG	485	184	669	0.275037	69	1	70	0.014286	1	Clonal	0.72	3.1			disease_causing	22.6		Passenger			Neutral	Tolerated
OVA_013_C2	PRIMARY	chr6	43020299	43020299	G	A	CUL7	exonic	onymous S	A76A,ENSG	441	205	646	0.317337	69	1	70	0.014286	1	Clonal	0.87	3.05			disease_causing	22.6		Passenger			Neutral	Tolerated
OVA_013_C3	PRIMARY	chr6	43020299	43020299	G	A	CUL7	exonic	onymous S	A76A,ENSG	466	233	699	0.333333	69	1	70	0.014286	1	Clonal	0.91	3.01			disease_causing	22.6		Passenger			Neutral	Tolerated
OVA_047_M1	METASTASIS	chr8	43157190	43157190	T	A	POTEA	cRNA_exon	NA	NA	465	146	611	0.238953	23	0	23	0	1	Clonal	0.47	2.01				1.783				damaging		
OVA_047_M2	METASTASIS	chr8	43157190	43157190	T	A	POTEA	cRNA_exon	NA	NA	482	126	608	0.207237	23	0	23	0	0.93	Clonal	0.44	2.01				1.783				damaging		
OVA_047_M3	METASTASIS	chr8	43157190	43157190	T	A	POTEA	cRNA_exon	NA	NA	186	49	235	0.208511	23	0	23	0	0.9	Clonal	0.6	3.02				1.783				damaging		
OVA_047_C1	PRIMARY	chr17	43192871	43192871	C	G	PLCD3	UTR3	NA	NA	178	55	233	0.236052	17	0	17	0	0.79	Sub	0.6	2.91			polymorphism	0.047						
OVA_047_C2	PRIMARY	chr17	43192871	43192871	C	G	PLCD3	UTR3	NA	NA	169	80	249	0.321285	17	0	17	0	1	Sub	0.64	2.76			polymorphism	0.047						
OVA_047_C3	Contralateral ovary	chr17	43192871	43192871	C	G	PLCD3	UTR3	NA	NA	161	61	222	0.274775	17	0	17	0	0.92	Clonal	0.6	2.94			polymorphism	0.047						
OVA_003_M1	METASTASIS	chr6	43193488	43193488	G	T	DNPH1	exonic	ynonymou	170T,ENSG	317	126	443	0.284424	34	0	34	0	1	Sub	0.57	1.84			polymorphism	10.53	p.P170T	Passenger	no weights	benign	Neutral	Tolerated
OVA_003_M2	METASTASIS	chr6	43193488	43193488	G	T	DNPH1	exonic	ynonymou	170T,ENSG	384	120	504	0.238095	34	0	34	0	1	Sub	0.46	1.81			polymorphism	10.53	p.P170T	Passenger	no weights	benign	Neutral	Tolerated
OVA_003_M3	METASTASIS	chr6	43193488	43193488	G	T	DNPH1	exonic	ynonymou	170T,ENSG	508	28	536	0.052239	34	0	34	0	0.55	Sub	0.34	2.67			polymorphism	10.53	p.P170T	Passenger	no weights	benign	Neutral	Tolerated
OVA_003_C1	PRIMARY	chr6	43193488	43193488	G	T	DNPH1	exonic	ynonymou	170T,ENSG	268	206	474	0.434599	34	0	34	0	1	Clonal	0.86	1.81			polymorphism	10.53	p.P170T	Passenger	no weights	benign	Neutral	Tolerated
OVA_003_C2	PRIMARY	chr6	43193488	43193488	G	T	DNPH1	exonic	ynonymou	170T,ENSG	324	201	525	0.382857	34	0	34	0	1	Clonal	0.7	3.61			polymorphism	10.53	p.P170T	Passenger	no weights	benign	Neutral	Tolerated
OVA_003_C3	PRIMARY	chr6	43193488	43193488	G	T	DNPH1	exonic	ynonymou	170T,ENSG	325	234	559	0.418605	34	0	34	0	1	Clonal	0.82	1.81			polymorphism	10.53	p.P170T	Passenger	no weights	benign	Neutral	Tolerated
OVA_365_M1	METASTASIS	chr1	43317324	43317324	C	A	ZNF691	exonic	ynonymou	000037250	168	376	544	0.691176	135	0	135	0	1	Clonal	0.85	1.87			polymorphism	24.7	p.T263N	Passenger		damaging	Neutral	Damaging
OVA_365_M2	METASTASIS	chr1	43317324	43317324	C	A	ZNF691	exonic	ynonymou	000037250	227	148	375	0.394667	135	0	135	0	1	Clonal	0.4	1.81			polymorphism	24.7	p.T263N	Passenger		damaging	Neutral	Damaging
OVA_365_M3	METASTASIS	chr1	43317324	43317324	C	A	ZNF691	exonic	ynonymou	000037250	181	363	544	0.667279	135	0	135	0	1	Clonal	0.82	1.89			polymorphism	24.7	p.T263N	Passenger		damaging	Neutral	Damaging
OVA_365_C1	Contralateral ovary	chr1	43317324	43317324	C	A	ZNF691	exonic	ynonymou	000037250	264	175	439	0.398633	135	0	135	0	1	Clonal	0.42	1.83			polymorphism	24.7	p.T263N	Passenger		damaging	Neutral	Damaging
OVA_365_C2	PRIMARY	chr1	43317324	43317324	C	A	ZNF691	exonic	ynonymou	000037250	259	231	490	0.471429	135	0	135	0	1	Clonal	0.62	1.92			polymorphism	24.7	p.T263N	Passenger		damaging	Neutral	Damaging
OVA_013_M1	METASTASIS	chr11	43348211	43348211	A	T	API5	intronic	NA	NA	310	165	475	0.347368	42	0	42	0	1	Clonal	0.67	3.06			polymorphism	8.078						
OVA_013_C3	PRIMARY	chr11	43348211	43348211	A	T	API5	intronic	NA	NA	304	219	523	0.418738	42	0	42	0	0.92	Clonal	0.91	3.01			polymorphism	8.078						
OVA_048_M1	METASTASIS	chr6	43402571	43402571	G	C	ABCC10	exonic	ynonymou	000037251	481	61	542	0.112546	85	0	85	0	0.62	Sub	0.56	3.94			disease_causing	25.3	p.Q531H	Passenger	SENGER/OT	damaging	Deleterious	Damaging
OVA_048_M1	METASTASIS	chr21	43413943	43413964	GTTTAAAA	-	ZBTB21	exonic	eshift dele	00310826:	260	82	342	0.239766	32	0	32		1	Clonal	0.56	3.94					p.D81lf	Passenger			NA	NA
OVA_048_M2	METASTASIS	chr21	43413943	43413964	GTTTAAAA	-	ZBTB21	exonic	eshift dele	00310826:	619	161	780	0.20641	32	0	32		1	Clonal	0.4	3.87					p.D81lfs	Passenger			NA	NA
OVA_048_M3	METASTASIS	chr21	43413943	43413964	GTTTAAAA	-	ZBTB21	exonic	eshift dele	00310826:	271	97	368	0.263587	32	0	32		1	Clonal	0.59	3.39					p.D81lfs	Passenger			NA	NA
OVA_048_C1	PRIMARY	chr21	43413943	43413964	GTTTAAAA	-	ZBTB21	exonic	eshift dele	00310826:	97	37	134	0.276119	32	0	32		0.55	Sub	0.67	1.82					p.D81lfs	Passenger			NA	NA
OVA_048_C2	PRIMARY	chr21	43413943	43413964	GTTTAAAA	-	ZBTB21	exonic	eshift dele	00310826:	147	88	235	0.374468	32	0	32		0.67	Sub	0.72	1.85					p.D81lfs	Passenger			NA	NA
OVA_048_C3	PRIMARY	chr21	43413943	43413964	GTTTAAAA	-	ZBTB21	exonic	eshift dele	00310826:	93	67	160	0.41875	32	0	32		0.71	Sub	0.74	1.8					p.D81lfs	Passenger			NA	NA
OVA_047_M1	METASTASIS	chr1	43616438	43616438	C	A	FAM183A	exonic	stopgain	000004100	385	252	637	0.395604	45	0	45	0	1	Clonal	0.47	2.01			polymorphism	14.12	p.T47K	Passenger	no weights	benign	Deleterious	Tolerated
OVA_047_M2	METASTASIS	chr1	43616438	43616438	C	A	FAM183A	exonic	stopgain	000004100	421	220	641	0.343214	45	0	45	0	1	Clonal	0.44	2.01			polymorphism	14.12	p.T47K	Passenger	no weights	benign	Deleterious	Tolerated
OVA_047_M3	METASTASIS	chr1	43616438	43616438	C	A	FAM183A	exonic	stopgain	000004100	253	128	381	0.335958	45	0	45	0	1	Clonal	0.6	3.02			polymorphism	14.12	p.T47K	Passenger	no weights	benign	Deleterious	Tolerated
OVA_048_C1	PRIMARY	chr6	43638635	43638635	C	T	RSPH9	exonic	ynonymou	000037216	389	24	413	0.058111	71	1	72	0.013889	0.17	Sub	0.67	1.82			polymorphism	24.5	p.R278C	Passenger	no weights	damaging	Neutral	Damaging
OVA_365_M3	METASTASIS	chr21	43710181	43710181	A	T	ABCG1	exonic	ynonymou	000398449	431	28	459	0.061002	84	0	84	0	0.15	Sub	0.82	1.89			disease_causing	18.84	p.T574S	Passenger	SENGER/OT	benign	Neutral	Tolerated
OVA_013_M1	METASTASIS	chr1	43778175	43778175	C	G	TIE1	exonic	onymous S	255L,ENSG	206	48	254	0.188976	36	0	36	0	0.94	Clonal	0.67	3.06			disease_causing	8.009		Passenger			Neutral	Tolerated
OVA_013_C1	PRIMARY	chr1	43778175	43778175	C	G	TIE1	exonic	onymous S	255L,ENSG	267	77	344	0.223837	36	0	36	0	1	Sub	0.72	3.1			disease_causing	8.009		Passenger			Neutral	Tolerated
OVA_013_C2	PRIMARY	chr1	43778175	43778175	C	G	TIE1	exonic	onymous S	255L,ENSG	317	77	394	0.195431	36	0	36	0	0.84	Sub	0.87	3.05			disease_causing	8.009		Passenger			Neutral	Tolerated
OVA_013_C3	PRIMARY	chr1	43778175	43778175	C	G	TIE1	exonic	onymous S	255L,ENSG	377	87	464	0.1875	36	0	36	0	0.79	Sub	0.91	3.01			disease_causing	8.009		Passenger			Neutral	Tolerated
OVA_047_C1	PRIMARY	chr18	44102277	44102277	A	C	LOXHD1	intronic	NA	NA	585	120	705	0.170213	44	0	44	0	1	Clonal	0.6	2.91			disease_causing	17.53						
OVA_047_C2	PRIMARY	chr18	44102277	44102277	A	C	LOXHD1	intronic	NA	NA	569	132	701	0.188302	44	0	44	0	0.97	Clonal	0.64	2.76			disease_causing	17.53						
OVA_047_C3	Contralateral ovary	chr18	44102277	44102277	A	C	LOXHD1	intronic	NA	NA	590	124	714	0.173669	44	0	44	0	0.93	Clonal	0.6	2.94			disease_causing	17.53						
OVA_047_C1	PRIMARY	chr18	44157791	44157791	C	T	LOXHD1	exonic	ynonymou	00441551:	406	259	665	0.389474	39	0	39	0	1	Clonal	0.6	2.91			polymorphism	25	p.V617M	Passenger	SENGER/OT	damaging	Neutral	Tolerated
OVA_047_C2	PRIMARY	chr18	44157791	44157791	C	T	LOXHD1	exonic	ynonymou	00441551:	391	245	636	0.38522	39	0	39	0	1	Clonal	0.64	2.76			polymorphism	25	p.V617M	Passenger	SENGER/OT	damaging	Neutral	Tolerated
OVA_047_C3	Contralateral ovary	chr18	44157791	44157791	C	T	LOXHD1	exonic	ynonymou	00441551:	408	234	642	0.364486	39	0	39	0	1	Clonal	0.6	2.94			polymorphism	25	p.V617M	Passenger	SENGER/OT	damaging	Neutral	Tolerated
OVA_013_M1	METASTASIS	chr6	44197703	44197704	TC	AA	SLC29A1	exonic	ynonymou	125N,ENSG	368	71	439	0.161731	46	0	46	0	0.81	Sub	0.67	3.06			disease_causing	26.6	p.I125N	Passenger	SENGER/OT	damaging	Deleterious	Damaging
OVA_013_M1	METASTASIS	chr6	44197705	44197705	A	C	SLC29A1	exonic	ynonymou	126P,ENSG	367	70	437	0.160183	48	0	48	0	0.8	Sub	0.67	3.06			disease_causing	27.5	p.T126P	Passenger	SENGER/OT	damaging	Deleterious	Damaging
OVA_365_M3	METASTASIS	chr2	44223108	44223108	G	A	LRPPRC	UTR5	NA	NA	196	10	206	0.048544	67	0	67	0	0.17	Sub	0.82	1.89			polymorphism	12.13						
OVA_013_M1	METASTASIS	chr7	44298470	44298470	C	T	CAMK2B	splicing	NA	NA	243	61	304	0.200658	82	1	83	0.012048	0.8	Sub	0.67	3.06			disease_causing	33		Passenger				
OVA_013_C1	PRIMARY	chr7	44298470	44298470	C	T	CAMK2B	splicing	NA	NA	275	107	382	0.280105	82	1	83	0.012048	1	Sub	0.72	3.1			disease_causing	33		Passenger				
OVA_013_C2	PRIMARY	chr7	44298470	44298470	C	T	CAMK2B	splicing	NA	NA	248	83	331	0.250755	82	1	83	0.012048	0.83	Sub	0.87	3.05			disease_causing	33		Passenger				
OVA_013_C3	PRIMARY	chr7	44298470	44298470	C	T	CAMK2B	splicing	NA	NA	340	118	458	0.257642	82	1	83	0.012048	0.82	Sub	0.91	3.01			disease_causing	33		Passenger				
OVA_048_C1	PRIMARY	chr19	44302986	44302986	G	C	LYPD5	exonic	ynonymou	000004146	80	22	102	0.215686	25	0	25	0	0.64	Sub	0.67	1.82			polymorphism	10.4	p.D116E	Passenger	SENGER/OT	benign	Neutral	Tolerated
OVA_048_C3	PRIMARY	chr19	44302986	44302986	G	C	LYPD5	exonic	ynonymou	000004146	100	35	135	0.259259	25	0	25	0	0.7	Sub	0.74	1.8			polymorphism	10.4	p.D116E	Passenger	SENGER/OT	benign	Neutral	Tolerated
OVA_003_M1	METASTASIS	chr20	44486006	44486006	C	G	ACOT8	UTR5	NA	NA	384	137	521	0.262956	33	1	34	0.029412	1	Sub	0.57	1.84			disease_causing	9.428						
OVA_003_M2	METASTASIS	chr20	44486006	44486006	C	G	ACOT8	UTR5	NA	NA	479	93	572	0.162587	33	1	34	0.029412	0.87	Sub	0.46	1.81			disease_causing	9.428						
OVA_003_M3	METASTASIS	chr20	44486006	44486006	C	G	ACOT8	UTR5	NA	NA	519	33	552	0.059783	33	1	34	0.029412	0.81	Sub	0.34	2.67			disease_causing	9.428						
OVA_003_C1	PRIMARY	chr20	44486006	44486006	C	G	ACOT8	UTR5	NA	NA	411	151	562	0.268683	33	1	34	0.029412	0.89	Sub	0.86	1.81			disease_causing	9.428						
OVA_003_C2	PRIMARY	chr20	44486006	44486006	C	G	ACOT8	UTR5	NA	NA	429	155	584	0.265411	33	1	34	0.029412	1	Sub	0.7	3.61			disease_causing	9.428						

Sample	Type	Chr	Start	End	Ref	Alt	Gene	unc.ensGer	icFunc.ens	hange.ens	t_ref	t_alt	t_total	t_VAF	n_ref	n_alt	n_total	ormal_VA	ncer_cell_f	on-based C	purity	ploidy	Cancer Gene	Driver Gene	Mutation Taster	ADD(PHRE	otein_chan	CHASM	THMM_Pr	OLYPHEN-	REDICTION	ICTION (cutoff=0.05)
OVA_003_C3	PRIMARY	chr20	44486006	44486006	C	G	ACOT8	UTR5	NA	NA	453	149	602	0.247508	33	1	34	0.029412	0.85	Sub	0.82	1.81			disease_causing	9.428						
OVA_365_M1	METASTASIS	chr4	44684350	44684350	G	T	GUF1	splicing	NA	NA	358	282	640	0.440625	61	0	61	0	1	Clonal	0.85	1.87			disease_causing	34		Passenger				
OVA_365_M2	METASTASIS	chr4	44684350	44684350	G	T	GUF1	splicing	NA	NA	409	93	502	0.185259	61	0	61	0	0.92	Clonal	0.4	1.81			disease_causing	34		Passenger				
OVA_365_M3	METASTASIS	chr4	44684350	44684350	G	T	GUF1	splicing	NA	NA	344	266	610	0.436066	61	0	61	0	1	Clonal	0.82	1.89			disease_causing	34		Passenger				
OVA_365_C1	Contralateral ovary	chr4	44684350	44684350	G	T	GUF1	splicing	NA	NA	393	118	511	0.23092	61	0	61	0	1	Clonal	0.42	1.83			disease_causing	34		Passenger				
OVA_365_C2	PRIMARY	chr4	44684350	44684350	G	T	GUF1	splicing	NA	NA	412	170	582	0.292096	61	0	61	0	0.95	Clonal	0.62	1.92			disease_causing	34		Passenger				
OVA_048_C3	PRIMARY	chr7	44802914	44802914	G	C	ZMIZ2	exonic	ynonymou	000433667	152	91	243	0.374486	66	0	66	0	1	Sub	0.74	1.8			disease_causing	25.3	p.G591R	Passenger		damaging	Deleterious	Damaging
OVA_048_C1	PRIMARY	chr7	44802914	44802914	G	C	ZMIZ2	exonic	ynonymou	000433667	118	43	161	0.267081	66	0	66	0	0.8	Sub	0.67	1.82			disease_causing	25.3	p.G591R	Passenger		damaging	Deleterious	Damaging
OVA_047_C2	PRIMARY	chr21	44836749	44836749	G	T	SIK1	exonic	ynonymou	000270162	83	25	108	0.231481	66	0	66	0	0.96	Clonal	0.64	2.76			polymorphism	16.29	p.P742H	Passenger	SENGER/OT	damaging	Deleterious	Damaging
OVA_047_C1	PRIMARY	chr11	44968605	44968605	G	C	TP53I11	exonic	ynonymou	000005251	618	40	658	0.06079	69	0	69	0	0.32	Sub	0.6	2.91			polymorphism	1.199					Neutral	Damaging
OVA_047_M3	METASTASIS	chr2	44970762	44970762	G	A	CAMKMT	exonic	ynonymou	000003784	382	4	386	0.010363	29	0	29	0	0.05	Sub	0.6	3.02			disease_causing	19.63	p.V209I	Passenger	SENGER/OT	damaging	Neutral	Tolerated
OVA_047_C1	PRIMARY	chr2	44970762	44970762	G	A	CAMKMT	exonic	ynonymou	000003784	365	104	469	0.221748	29	0	29	0	0.96	Sub	0.6	2.91			disease_causing	19.63	p.V209I	Passenger	SENGER/OT	damaging	Neutral	Tolerated
OVA_047_C2	PRIMARY	chr2	44970762	44970762	G	A	CAMKMT	exonic	ynonymou	000003784	300	73	373	0.19571	29	0	29	0	0.81	Sub	0.64	2.76			disease_causing	19.63	p.V209I	Passenger	SENGER/OT	damaging	Neutral	Tolerated
OVA_047_C3	Contralateral ovary	chr2	44970762	44970762	G	A	CAMKMT	exonic	ynonymou	000003784	316	69	385	0.179221	29	0	29	0	0.78	Sub	0.6	2.94			disease_causing	19.63	p.V209I	Passenger	SENGER/OT	damaging	Neutral	Tolerated
OVA_048_M1	METASTASIS	chr19	45254629	45254629	C	G	BCL3	exonic	ynonymou	000016422	358	220	578	0.380623	49	0	49	0	1	Clonal	0.56	3.94	Yes	No	polymorphism	20.9	p.D134E	Passenger	CANCER	damaging	Deleterious	Tolerated
OVA_048_M2	METASTASIS	chr19	45254629	45254629	C	G	BCL3	exonic	ynonymou	000016422	423	131	554	0.236462	49	0	49	0	1	Clonal	0.4	3.87	Yes	No	polymorphism	20.9	p.D134E	Passenger	CANCER	damaging	Deleterious	Tolerated
OVA_048_M3	METASTASIS	chr19	45254629	45254629	C	G	BCL3	exonic	ynonymou	000016422	426	138	564	0.244681	49	0	49	0	1	Clonal	0.59	3.39	Yes	No	polymorphism	20.9	p.D134E	Passenger	CANCER	damaging	Deleterious	Tolerated
OVA_048_C1	PRIMARY	chr19	45254629	45254629	C	G	BCL3	exonic	ynonymou	000016422	285	130	415	0.313253	49	0	49	0	0.94	Clonal	0.67	1.82	Yes	No	polymorphism	20.9	p.D134E	Passenger	CANCER	damaging	Deleterious	Tolerated
OVA_048_C2	PRIMARY	chr19	45254629	45254629	C	G	BCL3	exonic	ynonymou	000016422	362	180	542	0.332103	49	0	49	0	0.92	Clonal	0.72	1.85	Yes	No	polymorphism	20.9	p.D134E	Passenger	CANCER	damaging	Deleterious	Tolerated
OVA_048_C3	PRIMARY	chr19	45254629	45254629	C	G	BCL3	exonic	ynonymou	000016422	306	149	455	0.327473	49	0	49	0	0.89	Clonal	0.74	1.8	Yes	No	polymorphism	20.9	p.D134E	Passenger	CANCER	damaging	Deleterious	Tolerated
OVA_048_C1	PRIMARY	chr11	45274269	45274269	C	A	SYT13	exonic	onymous S	0000002092	103	126	229	0.550218	35	0	35	0	1	Clonal	0.67	1.82			disease_causing	13.56		Passenger			Neutral	Tolerated
OVA_013_M1	METASTASIS	chr14	45369978	45369978	G	A	C14orf28	exonic	ynonymou	114K,ENSG	277	269	546	0.492674	62	0	62	0	1	Clonal	0.67	3.06			disease_causing	23.1	p.E114K	Passenger	SENGER/OT	benign	Neutral	Damaging
OVA_013_C1	PRIMARY	chr14	45369978	45369978	G	A	C14orf28	exonic	ynonymou	114K,ENSG	234	257	491	0.523422	62	0	62	0	1	Clonal	0.72	3.1			disease_causing	23.1	p.E114K	Passenger	SENGER/OT	benign	Neutral	Damaging
OVA_013_C2	PRIMARY	chr14	45369978	45369978	G	A	C14orf28	exonic	ynonymou	114K,ENSG	186	217	403	0.538462	62	0	62	0	1	Clonal	0.87	3.05			disease_causing	23.1	p.E114K	Passenger	SENGER/OT	benign	Neutral	Damaging
OVA_013_C3	PRIMARY	chr14	45369978	45369978	G	A	C14orf28	exonic	ynonymou	114K,ENSG	246	248	494	0.502024	62	0	62	0	1	Clonal	0.91	3.01			disease_causing	23.1	p.E114K	Passenger	SENGER/OT	benign	Neutral	Damaging
OVA_013_M1	METASTASIS	chr18	45377728	45377728	A	C	SMAD2	intronic	NA	NA	238	116	354	0.327684	53	0	53	0	0.98	Clonal	0.67	3.06	Yes		polymorphism	2.694						
OVA_013_C1	PRIMARY	chr18	45377728	45377728	A	C	SMAD2	intronic	NA	NA	170	160	330	0.484848	53	0	53	0	1	Clonal	0.72	3.1	Yes		polymorphism	2.694						
OVA_013_C2	PRIMARY	chr18	45377728	45377728	A	C	SMAD2	intronic	NA	NA	93	86	179	0.480447	53	0	53	0	1	Clonal	0.87	3.05	Yes		polymorphism	2.694						
OVA_013_C3	PRIMARY	chr18	45377728	45377728	A	C	SMAD2	intronic	NA	NA	151	167	318	0.525157	53	0	53	0	1	Clonal	0.91	3.01	Yes		polymorphism	2.694						
OVA_013_M1	METASTASIS	chr1	45500149	45500149	A	T	ZSWIM5	intronic	NA	NA	356	92	448	0.205357	28	0	28	0	1	Clonal	0.67	3.06			disease_causing	23						
OVA_013_C1	PRIMARY	chr1	45500149	45500149	A	T	ZSWIM5	intronic	NA	NA	414	129	543	0.237569	28	0	28	0	1	Clonal	0.72	3.1			disease_causing	23						
OVA_013_C2	PRIMARY	chr1	45500149	45500149	A	T	ZSWIM5	intronic	NA	NA	418	122	540	0.225926	28	0	28	0	0.97	Clonal	0.87	3.05			disease_causing	23						
OVA_013_C3	PRIMARY	chr1	45500149	45500149	A	T	ZSWIM5	intronic	NA	NA	424	128	552	0.231884	28	0	28	0	0.97	Clonal	0.91	3.01			disease_causing	23						
OVA_365_M1	METASTASIS	chr14	45693459	45693467	TTCACTTTC	-	MIS18BP1	exonic	ameshift de	L0806:exon	486	240	726	0.330579	50	0	50		0.78	Sub	0.85	1.87			polymorphism		E775_E777	Passenger			Deleterious	NA
OVA_378_M1	METASTASIS	chr10	45799075	45799075	C	G	OR13A1	exonic	ynonymou	000053605	358	186	544	0.341912	78	0	78	0	0.95	Clonal	0.72	2.57			disease_causing	25.2	p.V266L	Passenger	SENGER/OT	damaging	Deleterious	Damaging
OVA_378_M2	METASTASIS	chr10	45799075	45799075	C	G	OR13A1	exonic	ynonymou	000053605	304	229	533	0.429644	78	0	78	0	1	Clonal	0.85	2.57			disease_causing	25.2	p.V266L	Passenger	SENGER/OT	damaging	Deleterious	Damaging
OVA_378_M3	METASTASIS	chr10	45799075	45799075	C	G	OR13A1	exonic	ynonymou	000053605	367	195	562	0.346975	78	0	78	0	1	Clonal	0.67	2.57			disease_causing	25.2	p.V266L	Passenger	SENGER/OT	damaging	Deleterious	Damaging
OVA_378_C1	PRIMARY	chr10	45799075	45799075	C	G	OR13A1	exonic	ynonymou	000053605	402	168	570	0.294737	78	0	78	0	1	Clonal	0.59	2.6			disease_causing	25.2	p.V266L	Passenger	SENGER/OT	damaging	Deleterious	Damaging
OVA_378_C2	PRIMARY	chr10	45799075	45799075	C	G	OR13A1	exonic	ynonymou	000053605	401	143	544	0.262868	78	0	78	0	0.93	Clonal	0.56	2.59			disease_causing	25.2	p.V266L	Passenger	SENGER/OT	damaging	Deleterious	Damaging
OVA_378_C3	PRIMARY	chr10	45799075	45799075	C	G	OR13A1	exonic	ynonymou	000053605	408	145	553	0.262206	78	0	78	0	1	Clonal	0.51	2.6			disease_causing	25.2	p.V266L	Passenger	SENGER/OT	damaging	Deleterious	Damaging
OVA_365_M1	METASTASIS	chr3	45811781	45811781	G	T	SLC6A20	exonic	ynonymou	000035852	275	238	513	0.463938	66	0	66	0	1	Clonal	0.85	1.87			disease_causing	19.81	p.L340I	Passenger	SENGER/OT	damaging	Neutral	Tolerated
OVA_365_M2	METASTASIS	chr3	45811781	45811781	G	T	SLC6A20	exonic	ynonymou	000035852	299	124	423	0.293144	66	0	66	0	1	Clonal	0.4	1.81			disease_causing	19.81	p.L340I	Passenger	SENGER/OT	damaging	Neutral	Tolerated
OVA_365_M3	METASTASIS	chr3	45811781	45811781	G	T	SLC6A20	exonic	ynonymou	000035852	295	238	533	0.446529	66	0	66	0	1	Clonal	0.82	1.89			disease_causing	19.81	p.L340I	Passenger	SENGER/OT	damaging	Neutral	Tolerated
OVA_365_C1	Contralateral ovary	chr3	45811781	45811781	G	T	SLC6A20	exonic	ynonymou	000035852	335	135	470	0.287234	66	0	66	0	1	Clonal	0.42	1.83			disease_causing	19.81	p.L340I	Passenger	SENGER/OT	damaging	Neutral	Tolerated
OVA_365_C2	PRIMARY	chr3	45811781	45811781	G	T	SLC6A20	exonic	ynonymou	000035852	323	171	494	0.346154	66	0	66	0	1	Clonal	0.62	1.92			disease_causing	19.81	p.L340I	Passenger	SENGER/OT	damaging	Neutral	Tolerated
OVA_365_M1	METASTASIS	chr17	45915779	45915779	C	G	SCRN2	exonic	ynonymou	000002902	202	70	272	0.257353	121	0	121	0	0.87	Clonal	0.85	1.87			polymorphism	15.26	p.A334P	Passenger	SENGER/OT	benign	Neutral	Tolerated
OVA_048_C2	PRIMARY	chr20	45918864	45918864	G	C	ZMYND8	intronic	NA	NA	323	217	540	0.401852	45	0	45	0	0.72	Sub	0.72	1.85			polymorphism	3.262						
OVA_047_M1	METASTASIS	chr22	45937119	45937119	G	T	FBLN1	exonic	ynonymou	11D,ENSG	422	199	621	0.320451	106	0	106	0	1	Clonal	0.47	2.01			disease_causing	23.8	p.E349D	Passenger	SENGER/OT	damaging	Deleterious	Damaging
OVA_047_M2	METASTASIS	chr22	45937119	45937119	G	T	FBLN1	exonic	ynonymou	11D,ENSG	435	189	624	0.302885	106	0	106	0	1	Clonal	0.44	2.01			disease_causing	23.8	p.E349D	Passenger	SENGER/OT	damaging	Deleterious	Damaging
OVA_047_M3	METASTASIS	chr22	45937119	45937119	G	T	FBLN1	exonic	ynonymou	11D,ENSG	259	135	394	0.34264	106	0	106	0	1	Clonal	0.6	3.02			disease_causing	23.8	p.E349D	Passenger	SENGER/OT	damaging	Deleterious	Damaging
OVA_047_C1	PRIMARY	chr3	46021298	46021298	G	C	FYCO1	exonic	ynonymou	.L63V,ENSG	563	123	686	0.1793	48	0	48	0	0.96	Clonal	0.6	2.91			disease_causing	24.6	p.L63V	Passenger	SENGER/OT	damaging	Neutral	Damaging
OVA_047_C2	PRIMARY	chr3	46021298	46021298	G	C	FYCO1	exonic	ynonymou	.L63V,ENSG	505	138	643	0.214619	48	0	48	0	0.89	Clonal	0.64	2.76			disease_causing	24.6	p.L63V	Passenger	SENGER/OT	damaging	Neutral	Damaging
OVA_047_C3	Contralateral ovary	chr3	46021298	46021298	G	C	FYCO1	exonic	ynonymou	.L63V,ENSG	485	196	681	0.287812	48	0	48	0	1	Clonal	0.6	2.94			disease_causing	24.6	p.L63V	Passenger	SENGER/OT	damaging	Neutral	Damaging
OVA_365_C1	Contralateral ovary	chr21	46354211	46354211	G	A	C21orf67	exonic	ynonymou	000003978	316	107	423	0.252955	52	0	52	0	1	Clonal	0.42	1.83			polymorphism	10.81				damaging	Deleterious	Damaging
OVA_047_M2	METASTASIS	chr17	46608068	46608068	G	C	HOXB1	exonic	ynonymou	Q67E,ENSG	196	2	198	0.010101	112	0	112	0	0.04	Sub	0.44	2.01			disease_causing	17.76	p.Q67E	Passenger	SENGER/OT	benign	Neutral	Damaging
OVA_047_C1	PRIMARY	chr17	46608068	46608068	G	C	HOXB1	exonic	ynonymou	Q67E,ENSG	104	34	138	0.246377	112	0	112	0	0.82	Clonal	0.6	2.91			disease_causing	17.76	p.Q67E	Passenger	SENGER/OT	benign	Neutral	Damaging
OVA_047_C2	PRIMARY	chr17	46608068	46608068	G	C	HOXB1	exonic	ynonymou	Q67E,ENSG	86	34	120	0.283333	112	0	112	0	0.89	Clonal	0.64	2.76			disease_causing	17.76	p.Q67E	Passenger	SENGER/OT	benign	Neutral	Damaging
OVA_047_C3	Contralateral ovary	chr17	46608068	46608068	G	C	HOXB1	exonic	ynonymou	Q67E,ENSG	79	35	114	0.307018	112	0	112	0	1	Clonal	0.6	2.94			disease_causing	17.76	p.Q67E	Passenger	SENGER/OT	benign	Neutral	Damaging
OVA_048_M1	METASTASIS	chr22	46611248	46611248	C	G	PPARA	exonic	ynonymou	000042080	488	40	528	0.075758	48	0	48	0	0.42	Sub	0.56	3.94			polymorphism	0.2					Neutral	Tolerated
OVA_048_C1	PRIMARY	chr6	46655988	46655988	C	T	TDRD6	exonic	onymous S	G41G,ENSG	6	5	11	0.454545	30	0	30	0	1	Clonal	0.67	1.82			disease_causing	10.97		Passenger			Neutral	Tolerated
OVA_048_C2	PRIMARY	chr6	46655988	46655988	C	T	TDRD6	exonic	onymous S	G41G,ENSG	9	11	20	0.55	30	0	30	0	1	Clonal	0.72	1.85			disease_causing	10.97		Passenger			Neutral	Tolerated
OVA_048_C3	PRIMARY	chr6	46655988	46655988	C	T	TDRD6	exonic	onymous S	G41G,ENSG	12	15	27	0.555556	30	0	30	0	1	Clonal	0.74	1.8			disease_causing	10.97		Passenger			Neutral	Tolerated
OVA_047_C1	PRIMARY	chr13	46844501	46844501	C	G	C63;AY395	exonic	ynonymou	000044617	504	146	650	0.224615	36	0	36	0	0.97	Sub	0.6	2.91			disease_causing	5.647					Neutral	Damaging
OVA_047_C2	PRIMARY	chr13	46844501	46844501	C	G	C63;AY395	exonic	ynonymou	000044617	521	110	631	0.174326	36	0	36	0	0.55	Sub	0.64	2.76			disease_causing	5.647					Neutral	Damaging
OVA_047_C3	Contralateral ovary	chr13	46844501	46844501	C	G	C63;AY395	exonic	ynonymou	000044617	460	154	614	0.250814	36	0	36	0	0.84	Sub	0.6	2.94			disease_causing	5.647					Neutral	Damaging
OVA_047_C1	PRIMARY	chr10	46966867	46966867	C	A	SYT15	intronic	NA	NA	328	24	352	0.068182	99	0	99	0	0.23	Sub	0.6	2.91			disease_causing	13.04						
OVA_047_C1	PRIMARY	chr19	47236408	47236408	G	C	STRN4	exonic	ynonymou	T00000597	351	113	464	0.243534	59	0	59	0	1	Sub	0.6	2.91	Yes	No	polymorphism	13.73	p.L209V	Passenger	SENGER/OT	benign	Neutral	Tolerated
OVA_047_C2	PRIMARY	chr19	47236408	47236408	G	C	STRN4	exonic	ynonymou	T00000597	377	96	473	0.20296	59	0	59	0	0.84	Sub	0.64	2.76	Yes	No	polymorphism	13.73	p.L209V	Passenger	SENGER/OT	benign	Neutral	Tolerated
OVA_047_C3	Contralateral ovary	chr19	47236408	47236408	G	C	STRN4	exonic	ynonymou	T00000597	275	86	361	0.238227	59	0	59	0	1	Clonal	0.6	2.94	Yes	No	polymorphism	13.73	p.L209V	Passenger	SENGER/OT	benign	Neutral	Tolerated
OVA_378_M1	METASTASIS	chr19	47259651	47259651	C	T	FKRP	exonic	ynonymou	P315L,ENSG	48	22	70	0.314286	109	0	109	0	1	Clonal	0.72	2.57			disease_causing	24.9	p.P315L	Passenger	no weights	damaging	Deleterious	Damaging
OVA_378_M2	METASTASIS	chr19	47259651	47259651	C	T	FKRP	exonic	ynonymou	P315L,ENSG	49	22	71	0.309859	109	0	109	0	1	Clonal	0.85	2.57			disease_causing	24.9	p.P315L	Passenger	no weights	damaging	Deleterious	Damaging
OVA_378_M3	METASTASIS	chr19	47259651	47259651	C	T	FKRP	exonic	ynonymou	P315L,ENSG	86	41	127	0.322835	109	0	109	0	1	Clonal	0.67	2.57			disease_causing	24.9	p.P315L	Passenger	no weights	damaging	Deleterious	Damaging
OVA_378_C1	PRIMARY	chr19	47259651	47259651	C	T	FKRP	exonic	ynonymou	P315L,ENSG	61	25	86	0.290698	109	0	109	0	1	Clonal	0.59	2.6			disease_causing	24.9	p.P315L	Passenger	no weights	damaging	Deleterious	Damaging
OVA_378_C2	PRIMARY	chr19	47259651	47259651	C	T	FKRP	exonic	ynonymou	P315L,ENSG	42	13	55	0.236364	109	0	109	0	1	Clonal	0.56	2.59			disease_causing	24.9	p.P315L	Passenger	no weights	damaging	Deleterious	Damaging
OVA_378_C3	PRIMARY	chr19	47259651	47259651	C	T	FKRP	exonic	ynonymou	P315L,ENSG	44	16	60	0.266667	109	0	109	0	1	Clonal	0.51	2.6			disease_causing	24.9	p.P315L	Passenger	no weights	damaging	Deleterious	Damaging
OVA_047_C1	PRIMARY	chr20	47268055	47268055	G	T	PREX1	exonic	ynonymou	845N,ENSG	478	126	604	0.208609	39	0	39	0	1	Clonal	0.6	2.91			disease_causing	22.3	p.T845N	Passenger	SENGER/OT	benign	Neutral	Tolerated
OVA_047_C2	PRIMARY	chr20	47268055	47268055	G	T	PREX1	exonic	ynonymou	845N,ENSG	473	119	592	0.201014	39	0	39	0	1	Clonal	0.64	2.76			disease_causing	22.3	p.T845N	Passenger	SENGER/OT	benign	Neutral	Tolerated
OVA_047_C3	Contralateral ovary	chr20	47268055	47268055	G	T	PREX1	exonic	ynonymou	845N,ENSG	485	100	585	0.17094	39	0	39	0	0.91	Clonal	0.6	2.94			disease_causing	22.3	p.T845N	Passenger	SENGER/OT	benign	Neutral	Tolerated
OVA_047_M1	METASTASIS	chr20	47292729	47292729	A	C	PREX1	splicing	NA	NA	487	105	592	0.177365	33	0	33	0	0.92	Clonal	0.47	2.01			disease_causing	33		Passenger				
OVA_047_M2	METASTASIS	chr20	47292729	47292729	A	C	PREX1	splicing	NA	NA	504	116	620	0.187097	33	0	33	0	1	Clonal	0.44	2.01			disease_causing	33		Passenger				
OVA_047_M3	METASTASIS	chr20	47292729	47292729	A	C	PREX1	splicing	NA	NA	323	73	396	0.184343	33	0	33	0	0.98	Clonal	0.6	3.02			disease_causing	33		Passenger				
OVA_047_M2	METASTASIS	chr17	47302343	47302343	G	A	PHOSPHO1	exonic	onymous S	000005110	488	79	567	0.13933	104	0	104	0	0.49	Sub	0.44	2.01			polymorphism	16.48		Passenger			Neutral	Tolerated
OVA_365_M1	METASTASIS	chr11	47311748	47311748	A	G	MADD	exonic	ynonymou	00406482:e	308	207	515	0.401942	110	0	110	0	0.95	Clonal	0.85	1.87			disease_causing	22.6	p.T1018A	Passenger	SENGER/OT	benign	Neutral	Tolerated
OVA_365_M2	METASTASIS	chr11	47311748	47311748	A	G	MADD	exonic	ynonymou	00406482:e	344	90	434	0.207373	110	0	110	0	1	Clonal	0.4	1.81			disease_causing	22.6	p.T1018A	Passenger	SENGER/OT	benign	Neutral	Tolerated
OVA_365_M3	METASTASIS	chr11	47311748	47311748	A	G	MADD	exonic	ynonymou	00406482:e	289	234	523	0.447419	110	0	110	0	1	Clonal	0.82	1.89			disease_causing	22.6	p.T1018A	Passenger	SENGER/OT	benign	Neutral	Tolerated
OVA_365_C1	Contralateral ovary	chr11	47311748	47311748	A	G	MADD	exonic	ynonymou	00406482:e	347	115	462	0.248918	110	0	110	0	1	Clonal	0.42	1.83			disease_causing	22.6	p.T1018A	Passenger	SENGER/OT	benign	Neutral	Tolerated
OVA_365_C2	PRIMARY	chr11	47311748	47311748	A	G	MADD	exonic	ynonymou	00406482:e	343	162	505	0.320792	110	0	110	0	1	Clonal	0.62	1.92			disease_causing	22.6	p.T1018A	Passenger	SENGER/OT	benign	Neutral	Tolerated
OVA_013_M1	METASTASIS	chr11	47364546	47364546	C	A	MYBPC3	intronic	NA	NA	177	57	234	0.24359	41	0	41	0	0.97	Clonal	0.67	3.06			polymorphism	4.974						
OVA_013_C1	PRIMARY	chr11	47364546	47364546	C	A	MYBPC3	intronic	NA	NA	300	114	414	0.275362	41	0	41	0	1	Clonal	0.72	3.1			polymorphism	4.974						
OVA_013_C2	PRIMARY	chr11	47364546	47364546	C	A	MYBPC3	intronic	NA	NA	314	127	441	0.287982	41	0	41	0	0.95	Clonal	0.87	3.05			polymorphism	4.974						
OVA_013_C3	PRIMARY	chr11	47364546	47364546	C	A	MYBPC3	intronic	NA	NA	343	139	482	0.288382	41	0	41	0	0.92	Clonal	0.91	3.01			polymorphism	4.974						
OVA_365_M1	METASTASIS	chr11	47369177	47369177	C	T	MYBPC3	intronic	NA	NA	39	35	74	0.472973	70	0	70	0	1	Clonal	0.85	1.87			polymorphism	3.542						
OVA_365_M2	METASTASIS	chr11	47369177	47369177	C	T	MYBPC3	intronic	NA	NA	36	6	42	0.142857	70	0	70	0	0.71	Clonal	0.4	1.81			polymorphism	3.542						
OVA_365_M3	METASTASIS	chr11	47369177	47369177	C	T	MYBPC3	intronic	NA	NA	33	23	56	0.410714	70	0	70	0	1	Clonal	0.82	1.89			polymorphism	3.542						
OVA_365_C1	Contralateral ovary	chr11	47369177	47369177	C	T	MYBPC3	intronic	NA	NA	37	9	46	0.195652	70	0	70	0	0.94	Clonal	0.42	1.83			polymorphism	3.542						
OVA_365_C2	PRIMARY	chr11	47369177	47369177	C	T	MYBPC3	intronic	NA	NA	43	28	71	0.394366	70	0	70	0	1	Clonal	0.62	1.92			polymorphism	3.542						
OVA_047_M1	METASTASIS	chr17	47394823	47394823	C	T	ZNF652	exonic	ynonymou	000004302	455	210	665	0.315789	43	0	43	0	1	Clonal	0.47	2.01			disease_causing	22.2	p.D89N	Passenger	SENGER/OT	benign	Neutral	Tolerated
OVA_047_M2	METASTASIS	chr17	47394823	47394823	C	T	ZNF652	exonic	ynonymou	000004302	493	200	693	0.2886	43	0	43	0	1	Clonal	0.44	2.01			disease_causing	22.2	p.D89N	Passenger	SENGER/OT	benign	Neutral	Tolerated
OVA_047_M3	METASTASIS	chr17	47394823	47394823	C	T	ZNF652	exonic	ynonymou	000004302	295	159	454	0.35022	43	0	43	0	1	Clonal	0.6	3.02			disease_causing	22.2	p.D89N	Passenger	SENGER/OT	benign	Neutral	Tolerated
OVA_365_M1	METASTASIS	chr11	47470572	47470572	G	A	RAPSN	UTR5	NA	NA	53	52	105	0.495238	23	0	23	0	1	Clonal	0.85	1.87			disease_causing	15.85						

Sample	Type	Chr	Start	End	Ref	Alt	Gene	unc.ensGen	icFunc.ens	hange.ens	t_ref	t_alt	t_total	t_VAF	n_ref	n_alt	n_total	normal_VA	ncer_cell_f	on-based C	purity	ploidy	Cancer Gene	Driver Gene	Mutation Taster	ADD(PHRE	otein_chan	CHASM	ATHMM_Pr	OLYPHEN-	REDICTION	ICTION (cutoff=0.05)
OVA_365_M2	METASTASIS	chr11	47470572	47470572	G	A	RAPSN	UTR5	NA	NA	71	24	95	0.252632	23	0	23	0	1	Clonal	0.4	1.81			disease_causing	15.85						
OVA_365_M3	METASTASIS	chr11	47470572	47470572	G	A	RAPSN	UTR5	NA	NA	61	49	110	0.445455	23	0	23	0	1	Clonal	0.82	1.89			disease_causing	15.85						
OVA_365_C1	Contralateral ovary	chr11	47470572	47470572	G	A	RAPSN	UTR5	NA	NA	69	28	97	0.28866	23	0	23	0	1	Clonal	0.42	1.83			disease_causing	15.85						
OVA_365_C2	PRIMARY	chr11	47470572	47470572	G	A	RAPSN	UTR5	NA	NA	79	47	126	0.373016	23	0	23	0	1	Clonal	0.62	1.92			disease_causing	15.85						
OVA_365_M1	METASTASIS	chr11	47595276	47595276	C	G	KBTBD4	exonic	ynonymou	/255L,ENSG	324	220	544	0.404412	50	0	50	0	0.96	Sub	0.85	1.87			disease_causing	23.6	p.V280L	Passenger	SENGER/OT	damaging	Neutral	Tolerated
OVA_365_M2	METASTASIS	chr11	47595276	47595276	C	G	KBTBD4	exonic	ynonymou	/255L,ENSG	387	68	455	0.149451	50	0	50	0	0.74	Sub	0.4	1.81			disease_causing	23.6	p.V280L	Passenger	SENGER/OT	damaging	Neutral	Tolerated
OVA_365_M3	METASTASIS	chr11	47595276	47595276	C	G	KBTBD4	exonic	ynonymou	/255L,ENSG	346	195	541	0.360444	50	0	50	0	0.88	Sub	0.82	1.89			disease_causing	23.6	p.V280L	Passenger	SENGER/OT	damaging	Neutral	Tolerated
OVA_365_C1	Contralateral ovary	chr11	47595276	47595276	C	G	KBTBD4	exonic	ynonymou	/255L,ENSG	370	106	476	0.222689	50	0	50	0	1	Clonal	0.42	1.83			disease_causing	23.6	p.V280L	Passenger	SENGER/OT	damaging	Neutral	Tolerated
OVA_365_C2	PRIMARY	chr11	47595276	47595276	C	G	KBTBD4	exonic	ynonymou	/255L,ENSG	377	151	528	0.285985	50	0	50	0	0.93	Clonal	0.62	1.92			disease_causing	23.6	p.V280L	Passenger	SENGER/OT	damaging	Neutral	Tolerated
OVA_048_C3	PRIMARY	chr20	47607760	47607760	T	G	ARFGEF2	intronic	NA	NA	309	196	505	0.388119	27	0	27	0	1	Clonal	0.74	1.8			polymorphism	9.543						
OVA_047_M1	METASTASIS	chrX	47775433	47775433	T	A	ZNF81	exonic	ynonymou	463N,ENSG	451	150	601	0.249584	16	0	16	0	1	Sub	0.47	2.01			polymorphism	25.6	p.I463N	Passenger	SENGER/OT	damaging	Deleterious	Damaging
OVA_047_M2	METASTASIS	chrX	47775433	47775433	T	A	ZNF81	exonic	ynonymou	463N,ENSG	501	100	601	0.166389	16	0	16	0	0.75	Sub	0.44	2.01			polymorphism	25.6	p.I463N	Passenger	SENGER/OT	damaging	Deleterious	Damaging
OVA_047_M3	METASTASIS	chrX	47775433	47775433	T	A	ZNF81	exonic	ynonymou	463N,ENSG	201	77	278	0.276978	16	0	16	0	1	Sub	0.6	3.02			polymorphism	25.6	p.I463N	Passenger	SENGER/OT	damaging	Deleterious	Damaging
OVA_378_C3	PRIMARY	chr18	47799781	47799781	C	A	MBD1	exonic	ynonymou	00269468:	565	22	587	0.037479	92	0	92	0	0.18	Sub	0.51	2.6			polymorphism	14.82	p.A503S	Passenger	SENGER/OT	damaging	Neutral	Tolerated
OVA_047_C3	Contralateral ovary	chr21	47831176	47831176	G	T	PCNT	exonic	ynonymou	000359568	332	96	428	0.224299	38	0	38	0	0.98	Clonal	0.6	2.94			polymorphism	20.6	p.R1730L	Passenger	SENGER/OT	damaging	Deleterious	Damaging
OVA_047_C2	PRIMARY	chr21	47918610	47918610	C	A	DIP2A	exonic	onymous S	000041756	356	130	486	0.26749	70	0	70	0	1	Clonal	0.64	2.76			disease_causing	12.21		Passenger			Neutral	Tolerated
OVA_378_M1	METASTASIS	chr19	47960194	47960194	C	T	SLC8A2	exonic	ynonymou	201K,ENSG	136	36	172	0.209302	62	0	62	0	0.79	Sub	0.72	2.57			disease_causing	22.9	p.E445K	Passenger	SENGER/OT	damaging	Deleterious	Tolerated
OVA_378_M2	METASTASIS	chr19	47960194	47960194	C	T	SLC8A2	exonic	ynonymou	201K,ENSG	129	34	163	0.208589	62	0	62	0	0.7	Sub	0.85	2.57			disease_causing	22.9	p.E445K	Passenger	SENGER/OT	damaging	Deleterious	Tolerated
OVA_378_M3	METASTASIS	chr19	47960194	47960194	C	T	SLC8A2	exonic	ynonymou	201K,ENSG	137	39	176	0.221591	62	0	62	0	0.89	Sub	0.67	2.57			disease_causing	22.9	p.E445K	Passenger	SENGER/OT	damaging	Deleterious	Tolerated
OVA_378_C1	PRIMARY	chr19	47960194	47960194	C	T	SLC8A2	exonic	ynonymou	201K,ENSG	147	45	192	0.234375	62	0	62	0	1	Sub	0.59	2.6			disease_causing	22.9	p.E445K	Passenger	SENGER/OT	damaging	Deleterious	Tolerated
OVA_378_C2	PRIMARY	chr19	47960194	47960194	C	T	SLC8A2	exonic	ynonymou	201K,ENSG	113	21	134	0.156716	62	0	62	0	0.71	Sub	0.56	2.59			disease_causing	22.9	p.E445K	Passenger	SENGER/OT	damaging	Deleterious	Tolerated
OVA_378_C3	PRIMARY	chr19	47960194	47960194	C	T	SLC8A2	exonic	ynonymou	201K,ENSG	111	15	126	0.119048	62	0	62	0	0.59	Sub	0.51	2.6			disease_causing	22.9	p.E445K	Passenger	SENGER/OT	damaging	Deleterious	Tolerated
OVA_378_M1	METASTASIS	chr16	48145707	48145707	G	C	ABCC12	exonic	ynonymou	.702V,ENSG	256	153	409	0.374083	53	0	53	0	1	Clonal	0.72	2.57			disease_causing	17.92	p.L702V	Passenger	SENGER/OT	benign	Neutral	Tolerated
OVA_378_M2	METASTASIS	chr16	48145707	48145707	G	C	ABCC12	exonic	ynonymou	.702V,ENSG	216	188	404	0.465347	53	0	53	0	1	Clonal	0.85	2.57			disease_causing	17.92	p.L702V	Passenger	SENGER/OT	benign	Neutral	Tolerated
OVA_378_M3	METASTASIS	chr16	48145707	48145707	G	C	ABCC12	exonic	ynonymou	.702V,ENSG	298	197	495	0.39798	53	0	53	0	1	Clonal	0.67	2.57			disease_causing	17.92	p.L702V	Passenger	SENGER/OT	benign	Neutral	Tolerated
OVA_378_C1	PRIMARY	chr16	48145707	48145707	G	C	ABCC12	exonic	ynonymou	.702V,ENSG	970	20	991	0.020182	53	0	53	0	0.07	Sub	0.72	2.57			disease_causing	17.92	p.L702V	Passenger	SENGER/OT	benign	Neutral	Tolerated
OVA_048_M1	METASTASIS	chr7	48313918	48313918	A	G	CA13;AX746	exonic	ynonymou	000435803	71	48	119	0.403361	25	0	25	0	1	Clonal	0.56	3.94			polymorphism	12.92	p.K1552R	Passenger	SENGER/OT	benign	Neutral	Tolerated
OVA_048_M2	METASTASIS	chr7	48313918	48313918	A	G	CA13;AX746	exonic	ynonymou	000435803	109	44	153	0.287582	25	0	25	0	1	Clonal	0.4	3.87			polymorphism	12.92	p.K1552R	Passenger	SENGER/OT	benign	Neutral	Tolerated
OVA_048_M3	METASTASIS	chr7	48313918	48313918	A	G	CA13;AX746	exonic	ynonymou	000435803	102	49	151	0.324503	25	0	25	0	1	Clonal	0.59	3.39			polymorphism	12.92	p.K1552R	Passenger	SENGER/OT	benign	Neutral	Tolerated
OVA_047_C1	PRIMARY	chrX	48325509	48325509	C	A	SLC38A5	UTR5	NA	NA	461	137	598	0.229097	38	1	39	0.025641	1	Clonal	0.6	2.91			polymorphism	0.017						
OVA_047_C2	PRIMARY	chrX	48325509	48325509	C	A	SLC38A5	UTR5	NA	NA	411	161	572	0.281469	38	1	39	0.025641	1	Clonal	0.64	2.76			polymorphism	0.017						
OVA_047_C3	Contralateral ovary	chrX	48325509	48325509	C	A	SLC38A5	UTR5	NA	NA	428	155	583	0.265866	38	1	39	0.025641	1	Clonal	0.6	2.94			polymorphism	0.017						
OVA_047_M1	METASTASIS	chr7	48451920	48451920	G	C	ABCA13	intronic	NA	NA	496	130	626	0.207668	20	0	20	0	0.88	Sub	0.47	2.01			polymorphism	2.231						
OVA_047_M2	METASTASIS	chr7	48451920	48451920	G	C	ABCA13	intronic	NA	NA	529	117	646	0.181115	20	0	20	0	0.82	Sub	0.44	2.01			polymorphism	2.231						
OVA_047_M3	METASTASIS	chr7	48451920	48451920	G	C	ABCA13	intronic	NA	NA	329	58	387	0.149871	20	0	20	0	0.65	Sub	0.6	3.02			polymorphism	2.231						
OVA_047_C1	PRIMARY	chr17	48542722	48542722	G	C	CHAD	exonic	ynonymou	00258969:	223	127	350	0.362857	63	0	63	0	1	Clonal	0.6	2.91			disease_causing	16.74	p.D339E	Passenger	SENGER/OT	benign	Neutral	Tolerated
OVA_047_C2	PRIMARY	chr17	48542722	48542722	G	C	CHAD	exonic	ynonymou	00258969:	283	112	395	0.283544	63	0	63	0	0.89	Clonal	0.64	2.76			disease_causing	16.74	p.D339E	Passenger	SENGER/OT	benign	Neutral	Tolerated
OVA_047_C3	Contralateral ovary	chr17	48542722	48542722	G	C	CHAD	exonic	ynonymou	00258969:	245	89	334	0.266467	63	0	63	0	0.89	Clonal	0.6	2.94			disease_causing	16.74	p.D339E	Passenger	SENGER/OT	benign	Neutral	Tolerated
OVA_048_C1	PRIMARY	chr3	48610555	48610555	C	T	COL7A1	intronic	NA	NA	50	22	72	0.305556	65	0	65	0	0.91	Clonal	0.67	1.82			polymorphism	4.965						
OVA_048_C2	PRIMARY	chr3	48610555	48610555	C	T	COL7A1	intronic	NA	NA	89	44	133	0.330827	65	0	65	0	0.92	Clonal	0.72	1.85			polymorphism	4.965						
OVA_048_C3	PRIMARY	chr3	48610555	48610555	C	T	COL7A1	intronic	NA	NA	79	40	119	0.336134	65	0	65	0	0.91	Clonal	0.74	1.8			polymorphism	4.965						
OVA_013_M1	METASTASIS	chr16	48643845	48643845	T	A	N4BP1	exonic	onymous S	T000002623	66	18	84	0.214286	23	0	23	0	0.85	Clonal	0.67	3.06			disease_causing	13.89		Passenger			Neutral	Tolerated
OVA_013_C1	PRIMARY	chr16	48643845	48643845	T	A	N4BP1	exonic	onymous S	T000002623	50	20	70	0.285714	23	0	23	0	1	Sub	0.72	3.1			disease_causing	13.89		Passenger			Neutral	Tolerated
OVA_013_C2	PRIMARY	chr16	48643845	48643845	T	A	N4BP1	exonic	onymous S	T000002623	41	17	58	0.293103	23	0	23	0	0.97	Sub	0.87	3.05			disease_causing	13.89		Passenger			Neutral	Tolerated
OVA_013_C3	PRIMARY	chr16	48643845	48643845	T	A	N4BP1	exonic	onymous S	T000002623	65	16	81	0.197531	23	0	23	0	0.63	Sub	0.91	3.01			disease_causing	13.89		Passenger			Neutral	Tolerated
OVA_047_M1	METASTASIS	chr3	48684391	48684391	A	C	CELSR3	intronic	NA	NA	353	129	482	0.267635	70	0	70	0	1	Clonal	0.47	2.01			disease_causing	18.65						
OVA_047_M2	METASTASIS	chr3	48684391	48684391	A	C	CELSR3	intronic	NA	NA	412	131	543	0.241252	70	0	70	0	1	Clonal	0.44	2.01			disease_causing	18.65						
OVA_047_M3	METASTASIS	chr3	48684391	48684391	A	C	CELSR3	intronic	NA	NA	177	51	228	0.223684	70	0	70	0	0.97	Clonal	0.6	3.02			disease_causing	18.65						
OVA_047_M1	METASTASIS	chrX	48922612	48922612	G	T	CCDC120	exonic	ynonymou	222V,ENSG	275	66	341	0.193548	69	0	69	0	0.82	Sub	0.47	2.01			disease_causing	23.6	p.G257V	Passenger		damaging	Neutral	Tolerated
OVA_047_M2	METASTASIS	chrX	48922612	48922612	G	T	CCDC120	exonic	ynonymou	222V,ENSG	336	70	406	0.172414	69	0	69	0	0.78	Sub	0.44	2.01			disease_causing	23.6	p.G257V	Passenger		damaging	Neutral	Tolerated
OVA_047_M3	METASTASIS	chrX	48922612	48922612	G	T	CCDC120	exonic	ynonymou	222V,ENSG	149	40	189	0.21164	69	0	69	0	0.92	Sub	0.6	3.02			disease_causing	23.6	p.G257V	Passenger		damaging	Neutral	Tolerated
OVA_047_C1	PRIMARY	chrX	48922612	48922612	G	T	CCDC120	exonic	ynonymou	222V,ENSG	293	52	345	0.150725	69	0	69	0	0.8	Sub	0.6	2.91			disease_causing	23.6	p.G257V	Passenger		damaging	Neutral	Tolerated
OVA_047_C2	PRIMARY	chrX	48922612	48922612	G	T	CCDC120	exonic	ynonymou	222V,ENSG	301	83	384	0.216146	69	0	69	0	0.89	Sub	0.64	2.76			disease_causing	23.6	p.G257V	Passenger		damaging	Neutral	Tolerated
OVA_047_C3	Contralateral ovary	chrX	48922612	48922612	G	T	CCDC120	exonic	ynonymou	222V,ENSG	282	70	352	0.198864	69	0	69	0	0.87	Clonal	0.6	2.94			disease_causing	23.6	p.G257V	Passenger		damaging	Neutral	Tolerated
OVA_047_M3	METASTASIS	chr4	49019175	49019175	A	C	CWH43	intronic	NA	NA	337	4	341	0.01173	13	0	13	0	0.05	Sub	0.6	3.02			polymorphism	0.102						
OVA_047_C2	PRIMARY	chr4	49019175	49019175	A	C	CWH43	intronic	NA	NA	394	34	428	0.079439	13	0	13	0	0.33	Sub	0.64	2.76			polymorphism	0.102						
OVA_048_M2	METASTASIS	chr3	49040154	49040154	C	T	P4HTM	intronic	NA	NA	192	8	200	0.04	26	0	26	0	0.28	Sub	0.4	3.87			polymorphism	6.073						
OVA_048_M3	METASTASIS	chr3	49040154	49040154	C	T	P4HTM	intronic	NA	NA	205	38	243	0.156379	26	0	26	0	1	Sub	0.59	3.39			polymorphism	6.073						
OVA_047_C1	PRIMARY	chr19	49104369	49104369	G	T	FAM83E	exonic	onymous S	000026326	263	83	346	0.239884	34	0	34	0	1	Clonal	0.6	2.91			polymorphism	9.011		Passenger			Neutral	Tolerated
OVA_047_C2	PRIMARY	chr19	49104369	49104369	G	T	FAM83E	exonic	onymous S	000026326	268	69	337	0.204748	34	0	34	0	0.85	Clonal	0.64	2.76			polymorphism	9.011		Passenger			Neutral	Tolerated
OVA_047_C3	Contralateral ovary	chr19	49104369	49104369	G	T	FAM83E	exonic	onymous S	000026326	237	67	304	0.220395	34	0	34	0	0.96	Clonal	0.6	2.94			polymorphism	9.011		Passenger			Neutral	Tolerated
OVA_003_M1	METASTASIS	chr12	49254978	49254978	G	A	RND1	intronic	NA	NA	379	139	518	0.26834	27	0	27	0	0.95	Sub	0.57	1.84			polymorphism	4.148						
OVA_003_M2	METASTASIS	chr12	49254978	49254978	G	A	RND1	intronic	NA	NA	423	114	537	0.212291	27	0	27	0	0.93	Sub	0.46	1.81			polymorphism	4.148						
OVA_003_M3	METASTASIS	chr12	49254978	49254978	G	A	RND1	intronic	NA	NA	550	35	585	0.059829	27	0	27	0	0.63	Sub	0.34	2.67			polymorphism	4.148						
OVA_003_C1	PRIMARY	chr12	49254978	49254978	G	A	RND1	intronic	NA	NA	340	248	588	0.421769	27	0	27	0	0.98	Clonal	0.86	1.81			polymorphism	4.148						
OVA_003_C2	PRIMARY	chr12	49254978	49254978	G	A	RND1	intronic	NA	NA	330	266	596	0.446309	27	0	27	0	1	Clonal	0.7	3.61			polymorphism	4.148						
OVA_003_C3	PRIMARY	chr12	49254978	49254978	G	A	RND1	intronic	NA	NA	332	285	617	0.461912	27	0	27	0	1	Clonal	0.82	1.81			polymorphism	4.148						
OVA_047_C1	PRIMARY	chr13	49281481	49281481	T	C	CYSLTR2	exonic	onymous S	000002820	500	168	668	0.251497	59	0	59	0	1	Sub	0.6	2.91	Yes		disease_causing	1.168		Passenger			Neutral	Tolerated
OVA_047_C2	PRIMARY	chr13	49281481	49281481	T	C	CYSLTR2	exonic	onymous S	000002820	476	160	636	0.251572	59	0	59	0	0.79	Sub	0.64	2.76	Yes		disease_causing	1.168		Passenger			Neutral	Tolerated
OVA_047_C3	Contralateral ovary	chr13	49281481	49281481	T	C	CYSLTR2	exonic	onymous S	000002820	506	142	648	0.219136	59	0	59	0	0.73	Sub	0.6	2.94	Yes		disease_causing	1.168		Passenger			Neutral	Tolerated
OVA_047_M1	METASTASIS	chr20	49348330	49348330	C	G	PARD6B	exonic	ynonymou	.R3G,ENSG	19	3	22	0.136364	32	0	32	0	0.71	Clonal	0.47	2.01			disease_causing	23.3	p.R3G	Passenger	SENGER/OT	benign	Neutral	Damaging
OVA_047_M2	METASTASIS	chr20	49348330	49348330	C	G	PARD6B	exonic	ynonymou	.R3G,ENSG	8	5	13	0.384615	32	0	32	0	1	Clonal	0.44	2.01			disease_causing	23.3	p.R3G	Passenger	SENGER/OT	benign	Neutral	Damaging
OVA_365_M1	METASTASIS	chr3	49400091	49400091	C	G	RHOA	intronic	NA	NA	305	203	508	0.399606	27	0	27	0	0.94	Clonal	0.85	1.87	Yes		polymorphism	7.636						
OVA_047_M1	METASTASIS	chr20	49621067	49621067	G	C	KCNG1	exonic	ynonymou	000037157	83	13	96	0.135417	45	0	45	0	0.71	Clonal	0.47	2.01	Yes	No	disease_causing	24.9	p.L351V	Passenger	SENGER/OT	damaging	Deleterious	Damaging
OVA_047_M2	METASTASIS	chr20	49621067	49621067	G	C	KCNG1	exonic	ynonymou	000037157	119	34	153	0.222222	45	0	45	0	1	Clonal	0.44	2.01	Yes	No	disease_causing	24.9	p.L351V	Passenger	SENGER/OT	damaging	Deleterious	Damaging
OVA_047_M3	METASTASIS	chr20	49621067	49621067	G	C	KCNG1	exonic	ynonymou	000037157	48	17	65	0.261538	45	0	45	0	1	Clonal	0.6	3.02	Yes	No	disease_causing	24.9	p.L351V	Passenger	SENGER/OT	damaging	Deleterious	Damaging
OVA_378_M1	METASTASIS	chr3	49759307	49759309	CCA	-	GMPPB	exonic	ameshift de	308388:ex	398	212	610	0.347541	138	0	138		0.96	Sub	0.72	2.57			polymorphism		347_E348de	Passenger			Deleterious	NA
OVA_378_M2	METASTASIS	chr3	49759307	49759309	CCA	-	GMPPB	exonic	ameshift de	308388:ex	393	239	632	0.378165	138	0	138		0.89	Sub	0.85	2.57			polymorphism		347_E348de	Passenger			Deleterious	NA
OVA_378_M3	METASTASIS	chr3	49759307	49759309	CCA	-	GMPPB	exonic	ameshift de	308388:ex	438	207	645	0.32093	138	0	138		0.96	Sub	0.67	2.57			polymorphism		347_E348de	Passenger			Deleterious	NA
OVA_378_C1	PRIMARY	chr3	49759307	49759309	CCA	-	GMPPB	exonic	ameshift de	308388:ex	540	228	768	0.296875	138	0	138		1	Sub	0.59	2.6			polymorphism		347_E348de	Passenger			Deleterious	NA
OVA_378_C2	PRIMARY	chr3	49759307	49759309	CCA	-	GMPPB	exonic	ameshift de	308388:ex	546	191	737	0.259159	138	0	138		0.92	Sub	0.56	2.59			polymorphism		347_E348de	Passenger			Deleterious	NA
OVA_378_C3	PRIMARY	chr3	49759307	49759309	CCA	-	GMPPB	exonic	ameshift de	308388:ex	559	155	714	0.217087	138	0	138		0.85	Sub	0.51	2.6			polymorphism		347_E348de	Passenger			Deleterious	NA
OVA_365_C1	Contralateral ovary	chr13	49765258	49765258	T	A	FNDC3A	splicing	NA	NA	233	47	280	0.167857	30	0	30	0	0.81	Clonal	0.42	1.83			disease_causing	33		Passenger				
OVA_013_C2	PRIMARY	chr6	49806210	49806210	G	A	CRISP1	exonic	ynonymou	000033584	393	141	534	0.264045	35	0	35	0	0.87	Sub	0.87	3.05			disease_causing	24	p.P188S	Passenger	SENGER/OT	damaging	Deleterious	Damaging
OVA_047_M1	METASTASIS	chr3	49877377	49877377	T	C	TRAIP	intronic	NA	NA	322	304	626	0.485623	64	0	64	0	1	Clonal	0.47	2.01			polymorphism	1.894						
OVA_047_M2	METASTASIS	chr3	49877377	49877377	T	C	TRAIP	intronic	NA	NA	331	288	619	0.465267	64	0	64	0	1	Clonal	0.44	2.01			polymorphism	1.894						
OVA_047_M3	METASTASIS	chr3	49877377	49877377	T	C	TRAIP	intronic	NA	NA	182	164	346	0.473988	64	0	64	0	1	Clonal	0.6	3.02			polymorphism	1.894						
OVA_047_C1	PRIMARY	chr3	49877377	49877377	T	C	TRAIP	intronic	NA	NA	436	221	657	0.336377	64	0	64	0	1	Clonal	0.6	2.91			polymorphism	1.894						
OVA_047_C2	PRIMARY	chr3	49877377	49877377	T	C	TRAIP	intronic	NA	NA	368	249	617	0.403566	64	0	64	0	1	Clonal	0.64	2.76			polymorphism	1.894						
OVA_047_C3	Contralateral ovary	chr3	49877377	49877377	T	C	TRAIP	intronic	NA	NA	334	336	670	0.501493	64	0	64	0	1	Clonal	0.6	2.94			polymorphism	1.894						
OVA_047_C1	PRIMARY	chr3	49879343	49879343	G	C	TRAIP	exonic	ynonymou	?178R,ENSG	534	132	666	0.198198	39	0	39	0	1	Clonal	0.6	2.91			disease_causing	24.1	p.P178R	Passenger	SENGER/OT	damaging	Neutral	Damaging
OVA_047_C2	PRIMARY	chr3	49879343	49879343	G	C	TRAIP	exonic	ynonymou	?178R,ENSG	453	157	610	0.257377	39	0	39	0	1	Clonal	0.64	2.76			disease_causing	24.1	p.P178R	Passenger	SENGER/OT	damaging	Neutral	Damaging
OVA_047_C3	Contralateral ovary	chr3	49879343	49879343	G	C	TRAIP	exonic	ynonymou	?178R,ENSG	540	119	659	0.180577	39	0	39	0	1	Clonal	0.6	2.94			disease_causing	24.1	p.P178R	Passenger	SENGER/OT	damaging	Neutral	Damaging
OVA_047_C1	PRIMARY	chr12	49943315	49943315	C	T	KCNH3	exonic	onymous S	000025798	391	183	574	0.318815	150	0	150	0	1	Clonal	0.6	2.91			disease_causing	17.89		Passenger			Neutral	Tolerated
OVA_047_C2	PRIMARY	chr12	49943315	49943315	C	T	KCNH3	exonic	onymous S	000025798	381	191	572	0.333916	150	0	150	0	1	Clonal	0.64	2.76			disease_causing	17.89		Passenger			Neutral	Tolerated
OVA_047_C3	Contralateral ovary	chr12	49943315	49943315	C	T	KCNH3	exonic	onymous S	000025798	384	163	547	0.297989	150	0	150	0	1	Clonal	0.6	2.94			disease_causing	17.89		Passenger			Neutral	Tolerated
OVA_378_M1	METASTASIS	chr12	49993482	49993482	C	T	FAM186B	exonic	stopgain	000257894	371	159	530	0.3	73	0	73	0	1	Clonal	0.72	2.57			disease_causing_automat	35	p.W647X	Passenger			NA	NA
OVA_378_M2	METASTASIS	chr12	49993482	49993482	C	T	FAM186B	exonic	stopgain	000257894	350	164	514	0.319066	73	0	73	0	1	Clonal	0.85	2.57			disease_causing_automat	35	p.W647X	Passenger			NA	NA
OVA_378_M3	METASTASIS	chr12	49993482	49993482	C	T	FAM186B	exonic	stopgain	000257894	433	160	593	0.269815	73	0	73	0	1	Clonal	0.67	2.57			disease_causing_automat	35	p.W647X	Passenger			NA	NA
OVA_378_C1	PRIMARY	chr12	49993482	49993482	C	T	FAM186B	exonic	stopgain	000257894	413	159	572	0.277972	73	0	73	0	1	Clonal	0.59	2.6			disease_causing_automat	35	p.W647X	Passenger			NA	NA

Sample	Type	Chr	Start	End	Ref	Alt	Gene	unc.ensGen	icFunc.ens	hange.ens(	t_ref	t_alt	t_total	t_VAF	n_ref	n_alt	n_total	normal_VA	ncer_cell_f	on-based C	purity	ploidy	Cancer Gene	Driver Gene	Mutation Taster	ADD(PHRE	otein_chan	CHASM	THMM_Pr	OLYPHEN-	REDICTION	ICTION (cutoff=0.05)
OVA_378_C2	PRIMARY	chr12	49993482	49993482	C	T	FAM186B	exonic	stopgain	000257894	373	153	526	0.290875	73	0	73	0	1	Clonal	0.56	2.59			disease_causing_automat	35	p.W647X	Passenger			NA	NA
OVA_378_C3	PRIMARY	chr12	49993482	49993482	C	T	FAM186B	exonic	stopgain	000257894	361	89	450	0.197778	73	0	73	0	0.97	Clonal	0.51	2.6			disease_causing_automat	35	p.W647X	Passenger			NA	NA
OVA_003_M1	METASTASIS	chr22	50018499	50018499	C	A	BC033837	intronic	NA	NA	205	89	294	0.302721	53	0	53	0	0.77	Sub	0.57	1.84			polymorphism	3.402						
OVA_003_M2	METASTASIS	chr22	50018499	50018499	C	A	BC033837	intronic	NA	NA	283	71	354	0.200565	53	0	53	0	0.67	Sub	0.46	1.81			polymorphism	3.402						
OVA_003_M3	METASTASIS	chr22	50018499	50018499	C	A	BC033837	intronic	NA	NA	465	32	497	0.064386	53	0	53	0	0.49	Sub	0.34	2.67			polymorphism	3.402						
OVA_003_C1	PRIMARY	chr22	50018499	50018499	C	A	BC033837	intronic	NA	NA	64	196	260	0.753846	53	0	53	0	0.99	Clonal	0.86	1.81			polymorphism	3.402						
OVA_003_C2	PRIMARY	chr22	50018499	50018499	C	A	BC033837	intronic	NA	NA	97	211	308	0.685065	53	0	53	0	1	Clonal	0.7	3.61			polymorphism	3.402						
OVA_003_C3	PRIMARY	chr22	50018499	50018499	C	A	BC033837	intronic	NA	NA	106	220	326	0.674847	53	0	53	0	0.96	Clonal	0.82	1.81			polymorphism	3.402						
OVA_048_M1	METASTASIS	chrX	50121596	50121596	G	A	DGKK	cRNA_exon	NA	NA	482	135	617	0.218801	21	0	21	0	1	Clonal	0.56	3.94				16.57	p.H986Y	Passenger		benign		
OVA_048_M2	METASTASIS	chrX	50121596	50121596	G	A	DGKK	cRNA_exon	NA	NA	525	86	611	0.140753	21	0	21	0	0.98	Clonal	0.4	3.87				16.57	p.H986Y	Passenger		benign		
OVA_047_C1	PRIMARY	chr10	50178211	50178211	C	A	WDFY4	exonic	nonymous S	000325239:	370	198	568	0.348592	122	0	122	0	1	Clonal	0.6	2.91			disease_causing	1.049		Passenger			Neutral	Tolerated
OVA_047_C2	PRIMARY	chr10	50178211	50178211	C	A	WDFY4	exonic	nonymous S	000325239:	392	204	596	0.342282	122	0	122	0	1	Clonal	0.64	2.76			disease_causing	1.049		Passenger			Neutral	Tolerated
OVA_047_C3	Contralateral ovary	chr10	50178211	50178211	C	A	WDFY4	exonic	nonymous S	000325239:	331	166	497	0.334004	122	0	122	0	1	Clonal	0.6	2.94			disease_causing	1.049		Passenger			Neutral	Tolerated
OVA_365_C2	PRIMARY	chr3	50223641	50223641	G	C	SEMA3F	intronic	NA	NA	31	4	35	0.114286	63	0	63	0	0.37	Sub	0.62	1.92			polymorphism	7.72						
OVA_047_M1	METASTASIS	chr3	50230755	50230755	C	T	GNAT1	exonic	nonymous S	T00000433	613	35	648	0.054012	130	0	130	0	0.23	Sub	0.47	2.01			disease_causing	19.03		Passenger			Neutral	Tolerated
OVA_048_M1	METASTASIS	chr3	50324655	50324655	C	T	LSMEM2	UTR3	NA	NA	127	82	209	0.392344	26	0	26	0	1	Clonal	0.56	3.94			polymorphism	10.22						
OVA_048_M2	METASTASIS	chr3	50324655	50324655	C	T	LSMEM2	UTR3	NA	NA	132	59	191	0.308901	26	0	26	0	1	Clonal	0.4	3.87			polymorphism	10.22						
OVA_048_M3	METASTASIS	chr3	50324655	50324655	C	T	LSMEM2	UTR3	NA	NA	134	83	217	0.382488	26	0	26	0	1	Clonal	0.59	3.39			polymorphism	10.22						
OVA_365_C2	PRIMARY	chr22	50355254	50355254	G	C	PIM3	exonic	nonymous S	0000036061	45	16	61	0.262295	100	0	100	0	0.85	Clonal	0.62	1.92			disease_causing	11.22		Passenger			Neutral	Tolerated
OVA_048_M1	METASTASIS	chr12	50386022	50386022	G	A	RACGAP1	intronic	NA	NA	198	415	613	0.676998	11	0	11	0	1	Clonal	0.56	3.94			disease_causing	9.617						
OVA_048_M2	METASTASIS	chr12	50386022	50386022	G	A	RACGAP1	intronic	NA	NA	350	291	641	0.453978	11	0	11	0	1	Clonal	0.4	3.87			disease_causing	9.617						
OVA_048_M3	METASTASIS	chr12	50386022	50386022	G	A	RACGAP1	intronic	NA	NA	206	434	640	0.678125	11	0	11	0	1	Clonal	0.59	3.39			disease_causing	9.617						
OVA_048_C1	PRIMARY	chr12	50386022	50386022	G	A	RACGAP1	intronic	NA	NA	246	249	495	0.50303	11	0	11	0	1	Clonal	0.67	1.82			disease_causing	9.617						
OVA_048_C2	PRIMARY	chr12	50386022	50386022	G	A	RACGAP1	intronic	NA	NA	230	342	572	0.597902	11	0	11	0	1	Clonal	0.72	1.85			disease_causing	9.617						
OVA_048_C3	PRIMARY	chr12	50386022	50386022	G	A	RACGAP1	intronic	NA	NA	202	291	493	0.590264	11	0	11	0	1	Clonal	0.74	1.8			disease_causing	9.617						
OVA_378_M1	METASTASIS	chr3	50418144	50418144	G	A	CACNA2D2	intronic	NA	NA	169	123	292	0.421233	89	0	89	0	1	Clonal	0.72	2.57			disease_causing	9.692						
OVA_378_M2	METASTASIS	chr3	50418144	50418144	G	A	CACNA2D2	intronic	NA	NA	99	140	239	0.585774	89	0	89	0	1	Clonal	0.85	2.57			disease_causing	9.692						
OVA_378_M3	METASTASIS	chr3	50418144	50418144	G	A	CACNA2D2	intronic	NA	NA	226	135	361	0.373961	89	0	89	0	1	Clonal	0.67	2.57			disease_causing	9.692						
OVA_378_C1	PRIMARY	chr3	50418144	50418144	G	A	CACNA2D2	intronic	NA	NA	218	116	334	0.347305	89	0	89	0	1	Clonal	0.59	2.6			disease_causing	9.692						
OVA_378_C2	PRIMARY	chr3	50418144	50418144	G	A	CACNA2D2	intronic	NA	NA	149	75	224	0.334821	89	0	89	0	1	Clonal	0.56	2.59			disease_causing	9.692						
OVA_378_C3	PRIMARY	chr3	50418144	50418144	G	A	CACNA2D2	intronic	NA	NA	138	62	200	0.31	89	0	89	0	1	Clonal	0.51	2.6			disease_causing	9.692						
OVA_048_M1	METASTASIS	chr22	50471709	50471709	T	C	17REL;MLC	exonic	ynonymous	000266182:	520	39	559	0.069767	73	0	73	0	0.39	Sub	0.56	3.94			disease_causing	25.2	p.N402S	Passenger	SENGER/OT	damaging	Deleterious	Damaging
OVA_365_M1	METASTASIS	chr10	50535159	50535159	C	T	C10orf71	UTR3	NA	NA	70	59	129	0.457364	23	0	23	0	1	Clonal	0.85	1.87			polymorphism	0.608						
OVA_365_M2	METASTASIS	chr10	50535159	50535159	C	T	C10orf71	UTR3	NA	NA	64	18	82	0.219512	23	0	23	0	1	Clonal	0.4	1.81			polymorphism	0.608						
OVA_365_M3	METASTASIS	chr10	50535159	50535159	C	T	C10orf71	UTR3	NA	NA	70	47	117	0.401709	23	0	23	0	0.98	Clonal	0.82	1.89			polymorphism	0.608						
OVA_365_C1	Contralateral ovary	chr10	50535159	50535159	C	T	C10orf71	UTR3	NA	NA	67	17	84	0.202381	23	0	23	0	0.97	Clonal	0.42	1.83			polymorphism	0.608						
OVA_365_C2	PRIMARY	chr10	50535159	50535159	C	T	C10orf71	UTR3	NA	NA	81	40	121	0.330579	23	0	23	0	1	Clonal	0.62	1.92			polymorphism	0.608						
OVA_047_C1	PRIMARY	chr7	50560637	50560637	C	A	DDC	exonic	ynonymous	0000380984	611	63	674	0.093472	47	0	47	0	0.41	Sub	0.6	2.91			polymorphism	4.461	p.R337S	Passenger	SENGER/OT		Neutral	Damaging
OVA_048_C1	PRIMARY	chr22	50655579	50655579	T	G	SELO	intronic	NA	NA	36	7	43	0.162791	67	0	67	0	0.32	Sub	0.67	1.82			polymorphism	4.837						
OVA_013_M1	METASTASIS	chr22	50725604	50725604	G	C	PLXNB2	exonic	nonymous S	566R,ENSG	143	46	189	0.243386	59	1	60	0.016667	0.97	Clonal	0.67	3.06			polymorphism	0.134		Passenger			Neutral	Tolerated
OVA_013_C1	PRIMARY	chr22	50725604	50725604	G	C	PLXNB2	exonic	nonymous S	566R,ENSG	159	51	210	0.242857	59	1	60	0.016667	0.92	Clonal	0.72	3.1			polymorphism	0.134		Passenger			Neutral	Tolerated
OVA_013_C2	PRIMARY	chr22	50725604	50725604	G	C	PLXNB2	exonic	nonymous S	566R,ENSG	169	73	242	0.301653	59	1	60	0.016667	1	Clonal	0.87	3.05			polymorphism	0.134		Passenger			Neutral	Tolerated
OVA_013_C3	PRIMARY	chr22	50725604	50725604	G	C	PLXNB2	exonic	nonymous S	566R,ENSG	210	87	297	0.292929	59	1	60	0.016667	0.93	Clonal	0.91	3.01			polymorphism	0.134		Passenger			Neutral	Tolerated
OVA_048_C1	PRIMARY	chr18	50918072	50918072	A	T	DCC	exonic	ynonymous	0000442544	151	68	219	0.310502	48	0	48	0	0.93	Sub	0.67	1.82	Yes	Yes	disease_causing	23.8	p.T835S	Passenger	SENGER/OT	damaging	Neutral	Damaging
OVA_048_C2	PRIMARY	chr18	50918072	50918072	A	T	DCC	exonic	ynonymous	0000442544	276	119	395	0.301266	48	0	48	0	0.84	Sub	0.72	1.85	Yes	Yes	disease_causing	23.8	p.T835S	Passenger	SENGER/OT	damaging	Neutral	Damaging
OVA_048_C3	PRIMARY	chr18	50918072	50918072	A	T	DCC	exonic	ynonymous	0000442544	178	88	266	0.330827	48	0	48	0	0.89	Sub	0.74	1.8	Yes	Yes	disease_causing	23.8	p.T835S	Passenger	SENGER/OT	damaging	Neutral	Damaging
OVA_003_M1	METASTASIS	chr19	51016543	51016543	A	C	ASPDH	exonic	nonymous S	000003892(	158	73	231	0.316017	79	0	79	0	1	Sub	0.57	1.84			polymorphism	16.12		Passenger			Neutral	Tolerated
OVA_003_M2	METASTASIS	chr19	51016543	51016543	A	C	ASPDH	exonic	nonymous S	000003892(	235	78	313	0.249201	79	0	79	0	1	Sub	0.46	1.81			polymorphism	16.12		Passenger			Neutral	Tolerated
OVA_003_M3	METASTASIS	chr19	51016543	51016543	A	C	ASPDH	exonic	nonymous S	000003892(	370	29	399	0.072682	79	0	79	0	0.77	Sub	0.34	2.67			polymorphism	16.12		Passenger			Neutral	Tolerated
OVA_003_C1	PRIMARY	chr19	51016543	51016543	A	C	ASPDH	exonic	nonymous S	000003892(	218	159	377	0.421751	79	0	79	0	0.98	Sub	0.86	1.81			polymorphism	16.12		Passenger			Neutral	Tolerated
OVA_003_C2	PRIMARY	chr19	51016543	51016543	A	C	ASPDH	exonic	nonymous S	000003892(	234	172	406	0.423645	79	0	79	0	1	Sub	0.7	3.61			polymorphism	16.12		Passenger			Neutral	Tolerated
OVA_003_C3	PRIMARY	chr19	51016543	51016543	A	C	ASPDH	exonic	nonymous S	000003892(	307	168	475	0.353684	79	0	79	0	0.86	Sub	0.82	1.81			polymorphism	16.12		Passenger			Neutral	Tolerated
OVA_047_C1	PRIMARY	chr7	51096039	51096039	T	A	COBL	exonic	nonymous S	10P,ENSG0	584	74	658	0.112462	99	0	99	0	0.49	Sub	0.6	2.91			polymorphism	0.214		Passenger			Neutral	Tolerated
OVA_048_C1	PRIMARY	chr19	51129216	51129223	CGCCCGGC	-	SYT3	exonic	neshift dele	445fs,ENSG	225	95	320	0.296875	49	0	49		0.89	Clonal	0.67	1.82			polymorphism	20.9	p.D134E	Passenger	CANCER	damaging	NA	NA
OVA_048_C2	PRIMARY	chr19	51129216	51129223	CGCCCGGC	-	SYT3	exonic	neshift dele	445fs,ENSG	155	85	240	0.354167	49	0	49		0.99	Clonal	0.72	1.85					p.A445Pfs	Passenger			NA	NA
OVA_048_C3	PRIMARY	chr19	51129216	51129223	CGCCCGGC	-	SYT3	exonic	neshift dele	445fs,ENSG	121	57	178	0.320225	49	0	49		0.87	Clonal	0.74	1.8					p.A445Pfs	Passenger			NA	NA
OVA_048_M1	METASTASIS	chr19	51380134	51380134	G	T	KLK2	exonic	ynonymous	000032532	226	139	365	0.380822	44	0	44	0	1	Clonal	0.56	3.94	Yes	No	polymorphism	1.361	p.R167L	Passenger	SENGER/OT	benign	Neutral	Tolerated
OVA_048_M2	METASTASIS	chr19	51380134	51380134	G	T	KLK2	exonic	ynonymous	000032532	341	124	465	0.266667	44	0	44	0	1	Clonal	0.4	3.87	Yes	No	polymorphism	1.361	p.R167L	Passenger	SENGER/OT	benign	Neutral	Tolerated
OVA_048_M3	METASTASIS	chr19	51380134	51380134	G	T	KLK2	exonic	ynonymous	000032532	202	173	375	0.461333	44	0	44	0	1	Clonal	0.59	3.39	Yes	No	polymorphism	1.361	p.R167L	Passenger	SENGER/OT	benign	Neutral	Tolerated
OVA_047_M1	METASTASIS	chr13	51519641	51519641	G	A	RNASEH2B	exonic	ynonymous	197S,ENSG	596	96	692	0.138728	72	0	72	0	0.58	Sub	0.47	2.01			polymorphism	9.808	p.G197S	Passenger		benign	Neutral	Tolerated
OVA_048_M1	METASTASIS	chr15	51535096	51535096	A	G	CYP19A1	exonic	ynonymous	.M5T,ENSG	353	198	551	0.359347	30	0	30	0	1	Clonal	0.56	3.94			polymorphism	19.31	p.M5T	Passenger	SENGER/OT	benign	Neutral	Tolerated
OVA_048_M2	METASTASIS	chr15	51535096	51535096	A	G	CYP19A1	exonic	ynonymous	.M5T,ENSG	408	148	556	0.266187	30	0	30	0	1	Clonal	0.4	3.87			polymorphism	19.31	p.M5T	Passenger	SENGER/OT	benign	Neutral	Tolerated
OVA_048_M3	METASTASIS	chr15	51535096	51535096	A	G	CYP19A1	exonic	ynonymous	.M5T,ENSG	369	163	532	0.306391	30	0	30	0	1	Clonal	0.59	3.39			polymorphism	19.31	p.M5T	Passenger	SENGER/OT	benign	Neutral	Tolerated
OVA_048_C1	PRIMARY	chr15	51535096	51535096	A	G	CYP19A1	exonic	ynonymous	.M5T,ENSG	248	133	381	0.349081	30	0	30	0	1	Clonal	0.67	1.82			polymorphism	19.31	p.M5T	Passenger	SENGER/OT	benign	Neutral	Tolerated
OVA_048_C2	PRIMARY	chr15	51535096	51535096	A	G	CYP19A1	exonic	ynonymous	.M5T,ENSG	337	195	532	0.366541	30	0	30	0	1	Clonal	0.72	1.85			polymorphism	19.31	p.M5T	Passenger	SENGER/OT	benign	Neutral	Tolerated
OVA_048_C3	PRIMARY	chr15	51535096	51535096	A	G	CYP19A1	exonic	ynonymous	.M5T,ENSG	290	165	455	0.362637	30	0	30	0	0.98	Clonal	0.74	1.8			polymorphism	19.31	p.M5T	Passenger	SENGER/OT	benign	Neutral	Tolerated
OVA_047_C1	PRIMARY	chr6	51619775	51619775	T	A	PKHD1	intronic	NA	NA	556	123	679	0.181149	11	0	11	0	0.97	Clonal	0.6	2.91			polymorphism	5.388						
OVA_047_C2	PRIMARY	chr6	51619775	51619775	T	A	PKHD1	intronic	NA	NA	540	124	664	0.186747	11	0	11	0	0.96	Clonal	0.64	2.76			polymorphism	5.388						
OVA_047_C3	Contralateral ovary	chr6	51619775	51619775	T	A	PKHD1	intronic	NA	NA	542	117	659	0.177542	11	0	11	0	0.95	Clonal	0.6	2.94			polymorphism	5.388						
OVA_013_M1	METASTASIS	chr15	51676158	51676158	A	T	GLDN	intronic	NA	NA	145	211	356	0.592697	58	0	58	0	1	Clonal	0.67	3.06			polymorphism	2.051						
OVA_013_C1	PRIMARY	chr15	51676158	51676158	A	T	GLDN	intronic	NA	NA	116	288	404	0.712871	58	0	58	0	1	Sub	0.72	3.1			polymorphism	2.051						
OVA_013_C2	PRIMARY	chr15	51676158	51676158	A	T	GLDN	intronic	NA	NA	118	274	392	0.69898	58	0	58	0	0.91	Sub	0.87	3.05			polymorphism	2.051						
OVA_013_C3	PRIMARY	chr15	51676158	51676158	A	T	GLDN	intronic	NA	NA	109	331	440	0.752273	58	0	58	0	0.89	Sub	0.91	3.01			polymorphism	2.051						
OVA_013_M1	METASTASIS	chr19	51729615	51729615	A	T	CD33	intronic	NA	NA	216	105	321	0.327103	50	1	51	0.019608	0.98	Clonal	0.67	3.06			polymorphism	9.483						
OVA_013_C1	PRIMARY	chr19	51729615	51729615	A	T	CD33	intronic	NA	NA	301	183	484	0.378099	50	1	51	0.019608	1	Clonal	0.72	3.1			polymorphism	9.483						
OVA_013_C2	PRIMARY	chr19	51729615	51729615	A	T	CD33	intronic	NA	NA	274	175	449	0.389755	50	1	51	0.019608	1	Clonal	0.87	3.05			polymorphism	9.483						
OVA_013_C3	PRIMARY	chr19	51729615	51729615	A	T	CD33	intronic	NA	NA	272	199	471	0.422505	50	1	51	0.019608	0.92	Clonal	0.91	3.01			polymorphism	9.483						
OVA_013_M1	METASTASIS	chr15	51755742	51755742	T	C	DMXL2	RNA_intro	NA	NA	53	43	96	0.447917	24	0	24	0	0.89	Clonal	0.67	3.06			polymorphism	9.776						
OVA_013_C1	PRIMARY	chr15	51755742	51755742	T	C	DMXL2	RNA_intro	NA	NA	22	47	69	0.681159	24	0	24	0	0.94	Clonal	0.72	3.1			polymorphism	9.776						
OVA_013_C2	PRIMARY	chr15	51755742	51755742	T	C	DMXL2	RNA_intro	NA	NA	6	11	17	0.647059	24	0	24	0	0.93	Clonal	0.87	3.05			polymorphism	9.776						
OVA_013_C3	PRIMARY	chr15	51755742	51755742	T	C	DMXL2	RNA_intro	NA	NA	14	43	57	0.754386	24	0	24	0	0.95	Clonal	0.91	3.01			polymorphism	9.776						
OVA_013_C1	PRIMARY	chr15	51792423	51792423	G	A	DMXL2	exonic	nonymous S	1000L,ENSG	314	97	411	0.23601	31	0	31	0	0.89	Clonal	0.72	3.1			disease_causing	17.8		Passenger			Neutral	Tolerated
OVA_013_C2	PRIMARY	chr15	51792423	51792423	G	A	DMXL2	exonic	nonymous S	1000L,ENSG	213	77	290	0.265517	31	0	31	0	0.88	Clonal	0.87	3.05			disease_causing	17.8		Passenger			Neutral	Tolerated
OVA_013_C3	PRIMARY	chr15	51792423	51792423	G	A	DMXL2	exonic	nonymous S	1000L,ENSG	273	118	391	0.30179	31	0	31	0	0.96	Clonal	0.91	3.01			disease_causing	17.8		Passenger			Neutral	Tolerated
OVA_013_M1	METASTASIS	chr6	51892757	51892757	A	G	PKHD1	intronic	NA	NA	469	122	591	0.20643	22	0	22	0	0.82	Sub	0.67	3.06			polymorphism	3.701						
OVA_047_C1	PRIMARY	chr6	51892912	51892912	A	T	PKHD1	intronic	NA	NA	550	113	663	0.170437	32	0	32	0	0.91	Clonal	0.6	2.91			polymorphism	8.154						
OVA_047_C2	PRIMARY	chr6	51892912	51892912	A	T	PKHD1	intronic	NA	NA	520	105	625	0.168	32	0	32	0	0.86	Clonal	0.64	2.76			polymorphism	8.154						
OVA_047_C3	Contralateral ovary	chr6	51892912	51892912	A	T	PKHD1	intronic	NA	NA	528	111	639	0.173709	32	0	32	0	0.93	Clonal	0.6	2.94			polymorphism	8.154						
OVA_013_M1	METASTASIS	chr13	51961714	51961714	A	G	INTS6	intronic	NA	NA	273	88	361	0.243767	50	0	50	0	0.97	Clonal	0.67	3.06			polymorphism	8.608						
OVA_013_C1	PRIMARY	chr13	51961714	51961714	A	G	INTS6	intronic	NA	NA	226	107	333	0.321321	50	0	50	0	1	Sub	0.72	3.1			polymorphism	8.608						
OVA_013_C2	PRIMARY	chr13	51961714	51961714	A	G	INTS6	intronic	NA	NA	217	74	291	0.254296	50	0	50	0	0.84	Sub	0.87	3.05			polymorphism	8.608						
OVA_013_C3	PRIMARY	chr13	51961714	51961714	A	G	INTS6	intronic	NA	NA	253	103	356	0.289326	50	0	50	0	0.92	Sub	0.91	3.01			polymorphism	8.608						
OVA_013_C3	PRIMARY	chr3	52185079	52185079	C	T	POC1A	exonic	ynonymous	R19Q,ENSG	424	159	583	0.272727	96	0	96	0	0.87	Sub	0.91	3.01			disease_causing	27.1	p.R19Q	Passenger	SENGER/OT	damaging	Neutral	Damaging
OVA_365_M1	METASTASIS	chr3	52257504	52257504	A	G	TLR9	exonic	nonymous S	276H,ENSG	288	198	486	0.407407	165	0	165	0	0.96	Clonal	0.85	1.87			disease_causing	0.952		Passenger			Neutral	Tolerated
OVA_365_M2	METASTASIS	chr3	52257504	52257504	A	G	TLR9	exonic	nonymous S	276H,ENSG	307	76	383	0.198433	165	0	165	0	0.98	Clonal	0.4	1.81			disease_causing	0.952		Passenger			Neutral	Tolerated
OVA_365_M3	METASTASIS	chr3	52257504	52257504	A	G	TLR9	exonic	nonymous S	276H,ENSG	275	160	435	0.367816	165	0	165	0	0.9	Clonal	0.82	1.89			disease_causing	0.952		Passenger			Neutral	Tolerated
OVA_365_C1	Contralateral ovary	chr3	52257504	52257504	A	G	TLR9	exonic	nonymous S	276H,ENSG	335	106	441	0.240363	165	0	165	0	1	Clonal	0.42	1.83			disease_causing	0.952		Passenger			Neutral	Tolerated
OVA_365_C2	PRIMARY	chr3	52257504	52257504	A	G	TLR9	exonic	nonymous S	276H,ENSG	331	149	480	0.310417	165	0	165	0	1	Clonal	0.62	1.92			disease_causing	0.952		Passenger			Neutral	Tolerated
OVA_047_M1	METASTASIS	chr3	52416390	52416390	G	T	DNAH1	exonic	ynonymous	000420323	489	157	646	0.243034	89	0	89	0	1	Clonal	0.47	2.01			disease_causing	14.5	p.E2620D	Passenger	SENGER/OT	damaging	Neutral	Tolerated
OVA_047_M2	METASTASIS	chr3	52416390	52416390	G	T	DNAH1	exonic	ynonymous	000420323	494	152	646	0.235294	89	0	89	0	1	Clonal	0.44	2.01			disease_causing	14.5	p.E2620D	Passenger	SENGER/OT	damaging	Neutral	Tolerated

Sample	Type	Chr	Start	End	Ref	Alt	Gene	unc.ensGen	icFunc.ens	hange.ens	t_ref	t_alt	t_total	t_VAF	n_ref	n_alt	n_total	normal_VA	ncer_cell_f	on-based C	purity	ploidy	Cancer Gene	Driver Gene	Mutation Taster	ADD(PHRE	otein_chan	CHASM	ATHMM_Pr	OLYPHEN-	REDICTION	ICTION (cutoff=0.05)
OVA_047_M3	METASTASIS	chr3	52416390	52416390	G	T	DNAH1	exonic	ynonymou	000420323	303	121	424	0.285377	89	0	89	0	1	Clonal	0.6	3.02			disease_causing	14.5	p.E2620D	Passenger	SENGER/OT	damaging	Neutral	Tolerated
OVA_365_M1	METASTASIS	chr3	52472035	52472035	T	C	SEMA3G	exonic	ynonymou	00023172	80	51	131	0.389313	100	0	100	0	0.92	Clonal	0.85	1.87			polymorphism	16.39	p.I564V	Passenger	SENGER/OT	benign	Neutral	Tolerated
OVA_365_M2	METASTASIS	chr3	52472035	52472035	T	C	SEMA3G	exonic	ynonymou	00023172	49	9	58	0.155172	100	0	100	0	0.77	Clonal	0.4	1.81			polymorphism	16.39	p.I564V	Passenger	SENGER/OT	benign	Neutral	Tolerated
OVA_365_M3	METASTASIS	chr3	52472035	52472035	T	C	SEMA3G	exonic	ynonymou	00023172	65	34	99	0.343434	100	0	100	0	0.84	Clonal	0.82	1.89			polymorphism	16.39	p.I564V	Passenger	SENGER/OT	benign	Neutral	Tolerated
OVA_013_C2	PRIMARY	chr19	52543959	52543959	G	A	ZNF432	intronic	NA	NA	358	55	413	0.133172	25	0	25	0	0.44	Sub	0.87	3.05			polymorphism	1.912						
OVA_048_C2	PRIMARY	chr3	52552620	52552620	C	T	STAB1	exonic	nonymous S	000321725	239	156	395	0.394937	71	0	71	0	1	Clonal	0.72	1.85			disease_causing	15.86		Passenger			Neutral	Tolerated
OVA_047_M1	METASTASIS	chr20	52570049	52570049	G	T	BCAS1	exonic	ynonymou	00371435:	559	142	701	0.202568	71	1	72	0.013889	1	Sub	0.47	2.01			polymorphism	20.8	p.N534K	Passenger	SENGER/OT	damaging	Deleterious	Damaging
OVA_047_M2	METASTASIS	chr20	52570049	52570049	G	T	BCAS1	exonic	ynonymou	00371435:	585	121	706	0.171388	71	1	72	0.013889	0.94	Sub	0.44	2.01			polymorphism	20.8	p.N534K	Passenger	SENGER/OT	damaging	Deleterious	Damaging
OVA_047_M3	METASTASIS	chr20	52570049	52570049	G	T	BCAS1	exonic	ynonymou	00371435:	536	66	602	0.109635	71	1	72	0.013889	0.58	Sub	0.6	3.02			polymorphism	20.8	p.N534K	Passenger	SENGER/OT	damaging	Deleterious	Damaging
OVA_048_M1	METASTASIS	chr18	52608324	52608324	-	A	CCDC68	intronic	NA	NA	256	77	333	0.231231	47	1	48		1	Clonal	0.56	3.94			polymorphism							
OVA_048_M2	METASTASIS	chr18	52608324	52608324	-	A	CCDC68	intronic	NA	NA	647	118	765	0.154248	47	1	48		1	Clonal	0.4	3.87			polymorphism							
OVA_048_M3	METASTASIS	chr18	52608324	52608324	-	A	CCDC68	intronic	NA	NA	229	66	295	0.223729	47	1	48		1	Clonal	0.59	3.39			polymorphism							
OVA_048_C1	PRIMARY	chr18	52608324	52608324	-	A	CCDC68	intronic	NA	NA	281	129	410	0.314634	47	1	48		0.94	Sub	0.67	1.82			polymorphism							
OVA_048_C2	PRIMARY	chr18	52608324	52608324	-	A	CCDC68	intronic	NA	NA	304	130	434	0.299539	47	1	48		0.83	Sub	0.72	1.85			polymorphism							
OVA_048_C3	PRIMARY	chr18	52608324	52608324	-	A	CCDC68	intronic	NA	NA	263	121	384	0.315104	47	1	48		0.85	Sub	0.74	1.8			polymorphism							
OVA_048_C1	PRIMARY	chr1	52747467	52747467	C	A	ZFYVE9	exonic	ynonymou	00371591	274	141	415	0.339759	62	0	62	0	1	Clonal	0.67	1.82			disease_causing	20.6	p.L1002I	Passenger	SENGER/OT	benign	Neutral	Tolerated
OVA_048_C2	PRIMARY	chr1	52747467	52747467	C	A	ZFYVE9	exonic	ynonymou	00371591	318	218	536	0.406716	62	0	62	0	1	Clonal	0.72	1.85			disease_causing	20.6	p.L1002I	Passenger	SENGER/OT	benign	Neutral	Tolerated
OVA_048_C3	PRIMARY	chr1	52747467	52747467	C	A	ZFYVE9	exonic	ynonymou	00371591	313	172	485	0.354639	62	0	62	0	0.96	Clonal	0.74	1.8			disease_causing	20.6	p.L1002I	Passenger	SENGER/OT	benign	Neutral	Tolerated
OVA_047_M1	METASTASIS	chr12	52980689	52980689	T	A	KRT72	intronic	NA	NA	413	144	557	0.258528	37	0	37	0	0.83	Sub	0.47	2.01			polymorphism	0.026						
OVA_365_M3	METASTASIS	chr17	52991985	52991985	G	C	TOM1L1	intronic	NA	NA	620	37	657	0.056317	30	0	30	0	0.19	Sub	0.82	1.89			polymorphism	4.363						
OVA_048_M1	METASTASIS	chr12	53043697	53043697	C	A	KRT2	splicing	NA	NA	147	246	393	0.625954	14	0	14	0	1	Clonal	0.56	3.94			disease_causing	34		Passenger				
OVA_048_M2	METASTASIS	chr12	53043697	53043697	C	A	KRT2	splicing	NA	NA	280	222	502	0.442231	14	0	14	0	1	Clonal	0.4	3.87			disease_causing	34		Passenger				
OVA_048_M3	METASTASIS	chr12	53043697	53043697	C	A	KRT2	splicing	NA	NA	186	269	455	0.591209	14	0	14	0	1	Clonal	0.59	3.39			disease_causing	34		Passenger				
OVA_048_C1	PRIMARY	chr12	53043697	53043697	C	A	KRT2	splicing	NA	NA	87	103	190	0.542105	14	0	14	0	1	Clonal	0.67	1.82			disease_causing	34		Passenger				
OVA_048_C2	PRIMARY	chr12	53043697	53043697	C	A	KRT2	splicing	NA	NA	170	187	357	0.52381	14	0	14	0	1	Clonal	0.72	1.85			disease_causing	34		Passenger				
OVA_048_C3	PRIMARY	chr12	53043697	53043697	C	A	KRT2	splicing	NA	NA	90	147	237	0.620253	14	0	14	0	1	Clonal	0.74	1.8			disease_causing	34		Passenger				
OVA_048_M1	METASTASIS	chrX	53105751	53105751	G	A	GPR173	UTR5	NA	NA	109	54	163	0.331288	18	0	18	0	1	Clonal	0.56	3.94			polymorphism	3.926						
OVA_048_M2	METASTASIS	chrX	53105751	53105751	G	A	GPR173	UTR5	NA	NA	126	46	172	0.267442	18	0	18	0	1	Clonal	0.4	3.87			polymorphism	3.926						
OVA_048_M3	METASTASIS	chrX	53105751	53105751	G	A	GPR173	UTR5	NA	NA	145	45	190	0.236842	18	0	18	0	1	Clonal	0.59	3.39			polymorphism	3.926						
OVA_048_C1	PRIMARY	chrX	53105751	53105751	G	A	GPR173	UTR5	NA	NA	47	22	69	0.318841	18	0	18	0	0.95	Sub	0.67	1.82			polymorphism	3.926						
OVA_048_C2	PRIMARY	chrX	53105751	53105751	G	A	GPR173	UTR5	NA	NA	173	61	234	0.260684	18	0	18	0	0.73	Sub	0.72	1.85			polymorphism	3.926						
OVA_048_C3	PRIMARY	chrX	53105751	53105751	G	A	GPR173	UTR5	NA	NA	86	43	129	0.333333	18	0	18	0	0.9	Sub	0.74	1.8			polymorphism	3.926						
OVA_013_M1	METASTASIS	chrX	53226074	53226074	-	A	KDM5C	exonic	neshift inse	00375379:	370	120	490	0.244898	39	0	39		1	Clonal	0.67	3.06	Yes	Yes	disease_causing		p.E926X	Passenger				
OVA_013_C2	PRIMARY	chrX	53226074	53226074	-	A	KDM5C	exonic	neshift inse	00375379:	535	158	693	0.227994	39	0	39		0.98	Clonal	0.87	3.05	Yes	Yes	disease_causing		p.E926X	Passenger				
OVA_013_C3	PRIMARY	chrX	53226074	53226074	-	A	KDM5C	exonic	neshift inse	00375379:	623	175	798	0.219298	39	0	39		0.92	Clonal	0.91	3.01	Yes	Yes	disease_causing		p.E926X	Passenger				
OVA_047_C1	PRIMARY	chr12	53233705	53233705	C	T	KRT78	exonic	ynonymou	A371T,ENS	224	106	330	0.321212	35	0	35	0	1	Clonal	0.6	2.91			disease_causing	24.2	p.A371T	Passenger	SENGER/OT	damaging	Deleterious	Damaging
OVA_047_C2	PRIMARY	chr12	53233705	53233705	C	T	KRT78	exonic	ynonymou	A371T,ENS	266	107	373	0.286863	35	0	35	0	0.9	Clonal	0.64	2.76			disease_causing	24.2	p.A371T	Passenger	SENGER/OT	damaging	Deleterious	Damaging
OVA_047_C3	Contralateral ovary	chr12	53233705	53233705	C	T	KRT78	exonic	ynonymou	A371T,ENS	218	114	332	0.343373	35	0	35	0	1	Clonal	0.6	2.94			disease_causing	24.2	p.A371T	Passenger	SENGER/OT	damaging	Deleterious	Damaging
OVA_047_M1	METASTASIS	chrX	53279887	53279887	T	A	IQSEC2	exonic	ynonymou	00003753	115	44	159	0.27673	74	0	74	0	1	Sub	0.47	2.01			disease_causing	23.4	p.D624V	Passenger	SENGER/OT	benign	Neutral	Damaging
OVA_047_M2	METASTASIS	chrX	53279887	53279887	T	A	IQSEC2	exonic	ynonymou	00003753	134	52	186	0.27957	74	0	74	0	1	Sub	0.44	2.01			disease_causing	23.4	p.D624V	Passenger	SENGER/OT	benign	Neutral	Damaging
OVA_047_M3	METASTASIS	chrX	53279887	53279887	T	A	IQSEC2	exonic	ynonymou	00003753	57	10	67	0.149254	74	0	74	0	0.65	Sub	0.6	3.02			disease_causing	23.4	p.D624V	Passenger	SENGER/OT	benign	Neutral	Damaging
OVA_013_M1	METASTASIS	chr1	53393000	53393000	G	C	SCP2	UTR5	NA	NA	81	26	107	0.242991	26	0	26	0	0.97	Clonal	0.67	3.06			polymorphism	8.374						
OVA_013_C1	PRIMARY	chr1	53393000	53393000	G	C	SCP2	UTR5	NA	NA	88	18	106	0.169811	26	0	26	0	0.64	Sub	0.72	3.1			polymorphism	8.374						
OVA_013_C2	PRIMARY	chr1	53393000	53393000	G	C	SCP2	UTR5	NA	NA	55	29	84	0.345238	26	0	26	0	1	Sub	0.87	3.05			polymorphism	8.374						
OVA_013_C3	PRIMARY	chr1	53393000	53393000	G	C	SCP2	UTR5	NA	NA	100	34	134	0.253731	26	0	26	0	0.81	Sub	0.91	3.01			polymorphism	8.374						
OVA_047_C1	PRIMARY	chr6	53519777	53519777	A	G	KLHL31	exonic	nonymous S	.F98F,ENSG	584	131	715	0.183217	45	0	45	0	0.79	Sub	0.6	2.91			disease_causing	15.32		Passenger			Neutral	Tolerated
OVA_047_C2	PRIMARY	chr6	53519777	53519777	A	G	KLHL31	exonic	nonymous S	.F98F,ENSG	567	141	708	0.199153	45	0	45	0	1	Sub	0.64	2.76			disease_causing	15.32		Passenger			Neutral	Tolerated
OVA_047_C3	Contralateral ovary	chr6	53519777	53519777	A	G	KLHL31	exonic	nonymous S	.F98F,ENSG	573	142	715	0.198601	45	0	45	0	1	Clonal	0.6	2.94			disease_causing	15.32		Passenger			Neutral	Tolerated
OVA_048_M1	METASTASIS	chr10	54048450	54048450	A	T	BC039504	RNA_intro	NA	NA	330	221	551	0.401089	14	0	14	0	1	Clonal	0.56	3.94			polymorphism	6.506						
OVA_048_M2	METASTASIS	chr10	54048450	54048450	A	T	BC039504	RNA_intro	NA	NA	387	167	554	0.301444	14	0	14	0	1	Clonal	0.4	3.87			polymorphism	6.506						
OVA_048_M3	METASTASIS	chr10	54048450	54048450	A	T	BC039504	RNA_intro	NA	NA	347	167	514	0.324903	14	0	14	0	1	Clonal	0.59	3.39			polymorphism	6.506						
OVA_048_C1	PRIMARY	chr10	54048450	54048450	A	T	BC039504	RNA_intro	NA	NA	287	149	436	0.341743	14	0	14	0	1	Clonal	0.67	1.82			polymorphism	6.506						
OVA_048_C2	PRIMARY	chr10	54048450	54048450	A	T	BC039504	RNA_intro	NA	NA	355	204	559	0.364937	14	0	14	0	1	Clonal	0.72	1.85			polymorphism	6.506						
OVA_048_C3	PRIMARY	chr10	54048450	54048450	A	T	BC039504	RNA_intro	NA	NA	310	165	475	0.347368	14	0	14	0	0.94	Clonal	0.74	1.8			polymorphism	6.506						
OVA_047_C1	PRIMARY	chr19	54178891	54178891	T	C	MIR520E	upstream	NA	NA	506	160	666	0.24024	26	0	26	0	1	Clonal	0.6	2.91				3.518						
OVA_047_C2	PRIMARY	chr19	54178891	54178891	T	C	MIR520E	upstream	NA	NA	479	157	636	0.246855	26	0	26	0	1	Clonal	0.64	2.76				3.518						
OVA_047_C3	Contralateral ovary	chr19	54178891	54178891	T	C	MIR520E	upstream	NA	NA	486	163	649	0.251156	26	0	26	0	1	Clonal	0.6	2.94				3.518						
OVA_047_C1	PRIMARY	chr19	54417765	54417765	G	T	CACNG7	exonic	ynonymou	00000391	345	50	395	0.126582	30	0	30	0	0.55	Sub	0.6	2.91			disease_causing	28.6	p.G70C	Passenger	SENGER/OT	damaging	Deleterious	Damaging
OVA_013_M1	METASTASIS	chr4	54442438	54442438	G	T	NX1;PDGFR	cRNA_exon	NA	NA	376	96	472	0.20339	39	1	40	0.025	1	Clonal	0.67	3.06			polymorphism	8.078						
OVA_013_C1	PRIMARY	chr4	54442438	54442438	G	T	NX1;PDGFR	cRNA_exon	NA	NA	458	122	580	0.210345	39	1	40	0.025	1	Sub	0.72	3.1			polymorphism	8.078						
OVA_013_C2	PRIMARY	chr4	54442438	54442438	G	T	NX1;PDGFR	cRNA_exon	NA	NA	470	109	579	0.188256	39	1	40	0.025	0.81	Sub	0.87	3.05			polymorphism	8.078						
OVA_013_C3	PRIMARY	chr4	54442438	54442438	G	T	NX1;PDGFR	cRNA_exon	NA	NA	462	144	606	0.237624	39	1	40	0.025	1	Sub	0.91	3.01			polymorphism	8.078						
OVA_003_C1	PRIMARY	chrX	54476731	54476731	A	C	FGD1	exonic	nonymous S	000037513	334	185	519	0.356455	29	0	29	0	0.83	Sub	0.86	1.81			disease_causing	19.53		Passenger			Neutral	Tolerated
OVA_003_C2	PRIMARY	chrX	54476731	54476731	A	C	FGD1	exonic	nonymous S	000037513	421	109	530	0.20566	29	0	29	0	1	Sub	0.7	3.61			disease_causing	19.53		Passenger			Neutral	Tolerated
OVA_003_C3	PRIMARY	chrX	54476731	54476731	A	C	FGD1	exonic	nonymous S	000037513	502	67	569	0.11775	29	0	29	0	0.29	Sub	0.82	1.81			disease_causing	19.53		Passenger			Neutral	Tolerated
OVA_047_M1	METASTASIS	chrX	54577550	54577550	C	A	GNL3L	intronic	NA	NA	490	138	628	0.219745	33	0	33	0	0.93	Clonal	0.47	2.01			polymorphism	4.09						
OVA_047_M2	METASTASIS	chrX	54577550	54577550	C	A	GNL3L	intronic	NA	NA	541	129	670	0.192537	33	0	33	0	0.87	Clonal	0.44	2.01			polymorphism	4.09						
OVA_047_M3	METASTASIS	chrX	54577550	54577550	C	A	GNL3L	intronic	NA	NA	275	73	348	0.20977	33	0	33	0	0.91	Clonal	0.6	3.02			polymorphism	4.09						
OVA_047_C1	PRIMARY	chrX	54577550	54577550	C	A	GNL3L	intronic	NA	NA	469	198	667	0.296852	33	0	33	0	1	Clonal	0.6	2.91			polymorphism	4.09						
OVA_047_C2	PRIMARY	chrX	54577550	54577550	C	A	GNL3L	intronic	NA	NA	412	241	653	0.369066	33	0	33	0	1	Clonal	0.64	2.76			polymorphism	4.09						
OVA_047_C3	Contralateral ovary	chrX	54577550	54577550	C	A	GNL3L	intronic	NA	NA	416	233	649	0.359014	33	0	33	0	1	Clonal	0.6	2.94			polymorphism	4.09						
OVA_013_C2	PRIMARY	chr19	54602996	54602996	G	C	OSCAR	intronic	NA	NA	431	93	524	0.177481	56	0	56	0	0.59	Sub	0.87	3.05			polymorphism	0.544						
OVA_365_C1	Contralateral ovary	chr1	54649905	54649905	C	G	CYB5RL	exonic	ynonymou	00534324:	353	86	439	0.1959	55	0	55	0	1	Clonal	0.42	1.83			polymorphism	15.14	p.E157Q	Passenger	SENGER/OT	benign	Neutral	Tolerated
OVA_048_C2	PRIMARY	chr8	54730077	54730077	C	T	ATP6V1H	exonic	ynonymou	107H,ENSG	357	190	547	0.347349	48	0	48	0	0.97	Clonal	0.72	1.85			disease_causing	33	p.R107H	Passenger	no weights	damaging	Deleterious	Damaging
OVA_003_M1	METASTASIS	chrX	54776408	54776408	G	A	ITIH6	exonic	ynonymou	000218436	137	59	196	0.30102	36	0	36	0	1	Sub	0.57	1.84			polymorphism	16.8	p.R1288C	Passenger	SENGER/OT	benign	Neutral	Tolerated
OVA_003_M2	METASTASIS	chrX	54776408	54776408	G	A	ITIH6	exonic	ynonymou	000218436	180	54	234	0.230769	36	0	36	0	1	Sub	0.46	1.81			polymorphism	16.8	p.R1288C	Passenger	SENGER/OT	benign	Neutral	Tolerated
OVA_003_M3	METASTASIS	chrX	54776408	54776408	G	A	ITIH6	exonic	ynonymou	000218436	263	20	283	0.070671	36	0	36	0	0.67	Sub	0.34	2.67			polymorphism	16.8	p.R1288C	Passenger	SENGER/OT	benign	Neutral	Tolerated
OVA_003_C1	PRIMARY	chrX	54776408	54776408	G	A	ITIH6	exonic	ynonymou	000218436	167	175	342	0.511696	36	0	36	0	1	Clonal	0.86	1.81			polymorphism	16.8	p.R1288C	Passenger	SENGER/OT	benign	Neutral	Tolerated
OVA_003_C2	PRIMARY	chrX	54776408	54776408	G	A	ITIH6	exonic	ynonymou	000218436	145	147	292	0.503425	36	0	36	0	1	Clonal	0.7	3.61			polymorphism	16.8	p.R1288C	Passenger	SENGER/OT	benign	Neutral	Tolerated
OVA_003_C3	PRIMARY	chrX	54776408	54776408	G	A	ITIH6	exonic	ynonymou	000218436	212	190	402	0.472637	36	0	36	0	1	Clonal	0.82	1.81			polymorphism	16.8	p.R1288C	Passenger	SENGER/OT	benign	Neutral	Tolerated
OVA_047_C3	Contralateral ovary	chrX	54785375	54785375	G	T	ITIH6	exonic	ynonymou	00002184	261	44	305	0.144262	33	0	33	0	0.77	Sub	0.6	2.94			polymorphism	15.79	p.Q378K	Passenger	SENGER/OT	benign	Neutral	Tolerated
OVA_013_C1	PRIMARY	chr19	54786046	54786046	T	C	LILRA6	downstrean	NA	NA	122	28	150	0.186667	21	0	21	0	0.52	Sub	0.72	3.1				0.417						
OVA_003_M1	METASTASIS	chr4	54853153	54853153	C	G	RPL21P44	cRNA_exon	NA	NA	402	132	534	0.247191	27	0	27	0	0.87	Sub	0.57	1.84			disease_causing	6.235						
OVA_003_M2	METASTASIS	chr4	54853153	54853153	C	G	RPL21P44	cRNA_exon	NA	NA	444	100	544	0.183824	27	0	27	0	0.8	Sub	0.46	1.81			disease_causing	6.235						
OVA_003_M3	METASTASIS	chr4	54853153	54853153	C	G	RPL21P44	cRNA_exon	NA	NA	557	29	586	0.049488	27	0	27	0	0.52	Sub	0.34	2.67			disease_causing	6.235						
OVA_003_C1	PRIMARY	chr4	54853153	54853153	C	G	RPL21P44	cRNA_exon	NA	NA	330	247	577	0.428076	27	0	27	0	0.99	Clonal	0.86	1.81			disease_causing	6.235						
OVA_003_C2	PRIMARY	chr4	54853153	54853153	C	G	RPL21P44	cRNA_exon	NA	NA	339	234	573	0.408377	27	0	27	0	1	Clonal	0.7	3.61			disease_causing	6.235						
OVA_003_C3	PRIMARY	chr4	54853153	54853153	C	G	RPL21P44	cRNA_exon	NA	NA	358	249	607	0.410214	27	0	27	0	1	Clonal	0.82	1.81			disease_causing	6.235						
OVA_003_M1	METASTASIS	chr8	54912412	54912412	A	C	TCEA1	UTR3	NA	NA	388	134	522	0.256705	45	0	45	0	0.91	Sub	0.57	1.84	Yes		disease_causing	8.88						
OVA_003_M2	METASTASIS	chr8	54912412	54912412	A	C	TCEA1	UTR3	NA	NA	479	124	603	0.205638	45	0	45	0	0.9	Sub	0.46	1.81	Yes		disease_causing	8.88						
OVA_003_M3	METASTASIS	chr8	54912412	54912412	A	C	TCEA1	UTR3	NA	NA	505	47	552	0.085145	45	0	45	0	0.9	Sub	0.34	2.67	Yes		disease_causing	8.88						
OVA_003_C1	PRIMARY	chr8	54912412	54912412	A	C	TCEA1	UTR3	NA	NA	301	247	548	0.45073	45	0	45	0	1	Clonal	0.86	1.81	Yes		disease_causing	8.88						
OVA_003_C2	PRIMARY	chr8	54912412	54912412	A	C	TCEA1	UTR3	NA	NA	313	187	500	0.374	45	0	45	0	1	Clonal	0.7	3.61	Yes		disease_causing	8.88						
OVA_003_C3	PRIMARY	chr8	54912412	54912412	A	C	TCEA1	UTR3	NA	NA	345	212	557	0.38061	45	0	45	0	0.92	Clonal	0.82	1.81	Yes		disease_causing	8.88						
OVA_365_M1	METASTASIS	chrX	55030270	55030270	G	A	APEX2	exonic	ynonymou	000037498	59	360	419	0.859189	78	0	78	0	1	Clonal	0.85	1.87			disease_causing	23.1	p.R203H	Passenger	SENGER/OT	damaging	Deleterious	Tolerated
OVA_365_M2	METASTASIS	chrX	55030270	55030270	G	A	APEX2	exonic	ynonymou	000037498	222	122	344	0.354651	78	0	78	0	1	Clonal	0.4	1.81			disease_causing	23.1	p.R203H	Passenger	SENGER/OT	damaging	Deleterious	Tolerated
OVA_365_M3	METASTASIS	chrX	55030270	55030270	G	A	APEX2	exonic	ynonymou	000037498	66	297	363	0.818182	78	0	78	0	1	Clonal	0.82	1.89			disease_causing	23.1	p.R203H	Passenger	SENGER/OT	damaging	Deleterious	Tolerated
OVA_365_C1	Contralateral ovary	chrX	55030270	55030270	G	A	APEX2	exonic	ynonymou	000037498	201	168	369	0.455285	78	0	78	0	1	Clonal	0.42	1.83			disease_causing	23.1	p.R203H	Passenger	SENGER/OT	damaging	Deleterious	Tolerated
OVA_365_C2	PRIMARY	chrX	55030270	55030270	G	A	APEX2	exonic	ynonymou	000037498	135	207	342	0.605263	78	0	78	0	1	Clonal	0.62	1.92			disease_causing	23.1	p.R203H	Passenger	SENGER/OT	damaging	Deleterious	Tolerated
OVA_378_C1	PRIMARY	chr17	55196348	55196348	A	G	AKAP1	exonic	nonymous S	00539273:	444	193	637	0.302983	84	0	84	0	1	Clonal	0.59	2.6			disease_causing	9.26		Passenger			Neutral	Tolerated

Sample	Type	Chr	Start	End	Ref	Alt	Gene	unc.ensGen	icFunc.ens	hange.ens	t_ref	t_alt	t_total	t_VAF	n_ref	n_alt	n_total	normal_VA	ncer_cell_f	on-based C	purity	ploidy	Cancer Gene	Driver Gene	Mutation Taster	ADD(PHRE	otein_chan	CHASM	ATHMM_Pr	OLYPHEN-	REDICTION	ICTION (cutoff=0.05)
OVA_378_C2	PRIMARY	chr17	55196348	55196348	A	G	AKAP1	exonic	ionymous S	00539273:	425	165	590	0.279661	84	0	84	0	0.99	Clonal	0.56	2.59			disease_causing	9.26		Passenger			Neutral	Tolerated
OVA_378_C3	PRIMARY	chr17	55196348	55196348	A	G	AKAP1	exonic	ionymous S	00539273:	438	158	596	0.265101	84	0	84	0	1	Clonal	0.51	2.6			disease_causing	9.26		Passenger			Neutral	Tolerated
OVA_003_M1	METASTASIS	chr2	55252759	55252759	C	G	RTN4	exonic	ynonymou	620Q,ENSG	430	181	611	0.296236	88	0	88	0	1	Sub	0.57	1.84			polymorphism	25.6	p.E826Q	Passenger	SENGER/OT	damaging	Neutral	Tolerated
OVA_003_M2	METASTASIS	chr2	55252759	55252759	C	G	RTN4	exonic	ynonymou	620Q,ENSG	482	120	602	0.199336	88	0	88	0	0.87	Sub	0.46	1.81			polymorphism	25.6	p.E826Q	Passenger	SENGER/OT	damaging	Neutral	Tolerated
OVA_003_M3	METASTASIS	chr2	55252759	55252759	C	G	RTN4	exonic	ynonymou	620Q,ENSG	605	33	638	0.051724	88	0	88	0	0.55	Sub	0.34	2.67			polymorphism	25.6	p.E826Q	Passenger	SENGER/OT	damaging	Neutral	Tolerated
OVA_003_C1	PRIMARY	chr2	55252759	55252759	C	G	RTN4	exonic	ynonymou	620Q,ENSG	338	292	630	0.463492	88	0	88	0	1	Clonal	0.86	1.81			polymorphism	25.6	p.E826Q	Passenger	SENGER/OT	damaging	Neutral	Tolerated
OVA_003_C2	PRIMARY	chr2	55252759	55252759	C	G	RTN4	exonic	ynonymou	620Q,ENSG	363	270	633	0.42654	88	0	88	0	1	Clonal	0.7	3.61			polymorphism	25.6	p.E826Q	Passenger	SENGER/OT	damaging	Neutral	Tolerated
OVA_003_C3	PRIMARY	chr2	55252759	55252759	C	G	RTN4	exonic	ynonymou	620Q,ENSG	397	238	635	0.374803	88	0	88	0	0.91	Clonal	0.82	1.81			polymorphism	25.6	p.E826Q	Passenger	SENGER/OT	damaging	Neutral	Tolerated
OVA_048_C1	PRIMARY	chr1	55451801	55451801	T	G	TMEM61	exonic	ynonymou	T00003712	190	65	255	0.254902	37	0	37	0	0.76	Sub	0.67	1.82			polymorphism	23	p.L16R	Passenger	SENGER/OT	damaging	Deleterious	Damaging
OVA_048_C2	PRIMARY	chr1	55451801	55451801	T	G	TMEM61	exonic	ynonymou	T00003712	311	156	467	0.334047	37	0	37	0	0.93	Sub	0.72	1.85			polymorphism	23	p.L16R	Passenger	SENGER/OT	damaging	Deleterious	Damaging
OVA_048_C3	PRIMARY	chr1	55451801	55451801	T	G	TMEM61	exonic	ynonymou	T00003712	167	78	245	0.318367	37	0	37	0	0.86	Sub	0.74	1.8			polymorphism	23	p.L16R	Passenger	SENGER/OT	damaging	Deleterious	Damaging
OVA_048_M1	METASTASIS	chr8	55539149	55539149	T	G	RP1	exonic	ynonymou	000022067	235	129	364	0.354396	41	0	41	0	1	Clonal	0.56	3.94			polymorphism	16.13	p.F903V	Passenger	SENGER/OT	benign	Neutral	Tolerated
OVA_048_M2	METASTASIS	chr8	55539149	55539149	T	G	RP1	exonic	ynonymou	000022067	249	125	374	0.334225	41	0	41	0	1	Clonal	0.4	3.87			polymorphism	16.13	p.F903V	Passenger	SENGER/OT	benign	Neutral	Tolerated
OVA_048_M3	METASTASIS	chr8	55539149	55539149	T	G	RP1	exonic	ynonymou	000022067	252	73	325	0.224615	41	0	41	0	1	Clonal	0.59	3.39			polymorphism	16.13	p.F903V	Passenger	SENGER/OT	benign	Neutral	Tolerated
OVA_378_C3	PRIMARY	chr12	55688863	55688863	G	C	OR6C6	exonic	ynonymou	000035843	492	57	549	0.103825	53	0	53	0	0.51	Sub	0.51	2.6			polymorphism	13.02	p.R52G	Passenger	SENGER/OT	benign	Neutral	Damaging
OVA_048_M1	METASTASIS	chr11	55761897	55761897	C	T	OR5F1	exonic	ynonymou	000027840	335	184	519	0.354528	19	0	19	0	1	Clonal	0.56	3.94			polymorphism	17.49	p.V69M	Passenger	SENGER/OT	damaging	Neutral	Damaging
OVA_048_M2	METASTASIS	chr11	55761897	55761897	C	T	OR5F1	exonic	ynonymou	000027840	379	145	524	0.276718	19	0	19	0	1	Clonal	0.4	3.87			polymorphism	17.49	p.V69M	Passenger	SENGER/OT	damaging	Neutral	Damaging
OVA_048_M3	METASTASIS	chr11	55761897	55761897	C	T	OR5F1	exonic	ynonymou	000027840	343	187	530	0.35283	19	0	19	0	1	Clonal	0.59	3.39			polymorphism	17.49	p.V69M	Passenger	SENGER/OT	damaging	Neutral	Damaging
OVA_048_C1	PRIMARY	chr11	55761897	55761897	C	T	OR5F1	exonic	ynonymou	000027840	156	72	228	0.315789	19	0	19	0	0.94	Clonal	0.67	1.82			polymorphism	17.49	p.V69M	Passenger	SENGER/OT	damaging	Neutral	Damaging
OVA_048_C2	PRIMARY	chr11	55761897	55761897	C	T	OR5F1	exonic	ynonymou	000027840	313	188	501	0.37525	19	0	19	0	1	Clonal	0.72	1.85			polymorphism	17.49	p.V69M	Passenger	SENGER/OT	damaging	Neutral	Damaging
OVA_048_C3	PRIMARY	chr11	55761897	55761897	C	T	OR5F1	exonic	ynonymou	000027840	182	103	285	0.361404	19	0	19	0	0.98	Clonal	0.74	1.8			polymorphism	17.49	p.V69M	Passenger	SENGER/OT	damaging	Neutral	Damaging
OVA_365_M1	METASTASIS	chr2	55844444	55844444	C	G	SMEK2	UTR5	NA	NA	110	58	168	0.345238	39	0	39	0	0.82	Clonal	0.85	1.87			disease_causing	14.24						
OVA_365_M2	METASTASIS	chr2	55844444	55844444	C	G	SMEK2	UTR5	NA	NA	72	17	89	0.191011	39	0	39	0	0.95	Clonal	0.4	1.81			disease_causing	14.24						
OVA_365_M3	METASTASIS	chr2	55844444	55844444	C	G	SMEK2	UTR5	NA	NA	92	56	148	0.378378	39	0	39	0	0.92	Clonal	0.82	1.89			disease_causing	14.24						
OVA_365_C1	Contralateral ovary	chr2	55844444	55844444	C	G	SMEK2	UTR5	NA	NA	105	19	124	0.153226	39	0	39	0	0.74	Clonal	0.42	1.83			disease_causing	14.24						
OVA_365_C2	PRIMARY	chr2	55844444	55844444	C	G	SMEK2	UTR5	NA	NA	106	39	145	0.268966	39	0	39	0	0.87	Clonal	0.62	1.92			disease_causing	14.24						
OVA_365_C2	PRIMARY	chr6	55933881	55933881	G	T	COL21A1	exonic	ynonymou	32Q,ENSG0	413	97	510	0.190196	87	0	87	0	0.81	Sub	0.62	1.92			disease_causing	22.1	p.P685Q	Passenger	SENGER/OT	benign	Neutral	Tolerated
OVA_048_M1	METASTASIS	chr7	56065091	56065091	C	T	GBAS	exonic	ynonymou	210W,ENSG	431	207	638	0.324451	97	0	97	0	1	Clonal	0.56	3.94			disease_causing	28	p.R249W	Driver	SENGER/OT	damaging	Deleterious	Damaging
OVA_048_M2	METASTASIS	chr7	56065091	56065091	C	T	GBAS	exonic	ynonymou	210W,ENSG	552	80	632	0.126582	97	0	97	0	0.88	Clonal	0.4	3.87			disease_causing	28	p.R249W	Driver	SENGER/OT	damaging	Deleterious	Damaging
OVA_003_M1	METASTASIS	chr2	56102248	56102248	A	G	EFEMP1	intronic	NA	NA	186	53	239	0.221757	35	0	35	0	0.78	Sub	0.57	1.84	Yes		polymorphism	1.044						
OVA_003_M2	METASTASIS	chr2	56102248	56102248	A	G	EFEMP1	intronic	NA	NA	244	59	303	0.194719	35	0	35	0	0.85	Sub	0.46	1.81	Yes		polymorphism	1.044						
OVA_003_M3	METASTASIS	chr2	56102248	56102248	A	G	EFEMP1	intronic	NA	NA	267	11	278	0.039568	35	0	35	0	0.42	Sub	0.34	2.67	Yes		polymorphism	1.044						
OVA_003_C1	PRIMARY	chr2	56102248	56102248	A	G	EFEMP1	intronic	NA	NA	123	91	214	0.425234	35	0	35	0	0.99	Clonal	0.86	1.81	Yes		polymorphism	1.044						
OVA_003_C2	PRIMARY	chr2	56102248	56102248	A	G	EFEMP1	intronic	NA	NA	156	119	275	0.432727	35	0	35	0	1	Clonal	0.7	3.61	Yes		polymorphism	1.044						
OVA_003_C3	PRIMARY	chr2	56102248	56102248	A	G	EFEMP1	intronic	NA	NA	136	92	228	0.403509	35	0	35	0	0.98	Clonal	0.82	1.81	Yes		polymorphism	1.044						
OVA_378_M1	METASTASIS	chr18	56246111	56246111	C	T	ALPK2	exonic	ynonymou	000036167	494	166	660	0.251515	40	0	40	0	0.95	Clonal	0.72	2.57			polymorphism	10.05	p.E633K	Passenger	SENGER/OT	benign	Neutral	Tolerated
OVA_378_M2	METASTASIS	chr18	56246111	56246111	C	T	ALPK2	exonic	ynonymou	000036167	486	178	664	0.268072	40	0	40	0	0.9	Clonal	0.85	2.57			polymorphism	10.05	p.E633K	Passenger	SENGER/OT	benign	Neutral	Tolerated
OVA_378_M3	METASTASIS	chr18	56246111	56246111	C	T	ALPK2	exonic	ynonymou	000036167	572	162	734	0.220708	40	0	40	0	0.88	Clonal	0.67	2.57			polymorphism	10.05	p.E633K	Passenger	SENGER/OT	benign	Neutral	Tolerated
OVA_378_C1	PRIMARY	chr18	56246111	56246111	C	T	ALPK2	exonic	ynonymou	000036167	526	153	679	0.225331	40	0	40	0	0.99	Sub	0.59	2.6			polymorphism	10.05	p.E633K	Passenger	SENGER/OT	benign	Neutral	Tolerated
OVA_378_C2	PRIMARY	chr18	56246111	56246111	C	T	ALPK2	exonic	ynonymou	000036167	549	104	653	0.159265	40	0	40	0	0.72	Sub	0.56	2.59			polymorphism	10.05	p.E633K	Passenger	SENGER/OT	benign	Neutral	Tolerated
OVA_378_C3	PRIMARY	chr18	56246111	56246111	C	T	ALPK2	exonic	ynonymou	000036167	528	119	647	0.183926	40	0	40	0	0.9	Sub	0.51	2.6			polymorphism	10.05	p.E633K	Passenger	SENGER/OT	benign	Neutral	Tolerated
OVA_378_M1	METASTASIS	chr18	56246114	56246114	C	T	ALPK2	exonic	ynonymou	000036167	495	165	660	0.25	43	0	43	0	0.94	Clonal	0.72	2.57			polymorphism	9.556	p.G632R	Passenger	SENGER/OT	benign	Neutral	Tolerated
OVA_378_M2	METASTASIS	chr18	56246114	56246114	C	T	ALPK2	exonic	ynonymou	000036167	486	177	663	0.266968	43	0	43	0	0.89	Clonal	0.85	2.57			polymorphism	9.556	p.G632R	Passenger	SENGER/OT	benign	Neutral	Tolerated
OVA_378_M3	METASTASIS	chr18	56246114	56246114	C	T	ALPK2	exonic	ynonymou	000036167	569	166	735	0.22585	43	0	43	0	0.9	Clonal	0.67	2.57			polymorphism	9.556	p.G632R	Passenger	SENGER/OT	benign	Neutral	Tolerated
OVA_378_C1	PRIMARY	chr18	56246114	56246114	C	T	ALPK2	exonic	ynonymou	000036167	523	151	674	0.224036	43	0	43	0	0.99	Sub	0.59	2.6			polymorphism	9.556	p.G632R	Passenger	SENGER/OT	benign	Neutral	Tolerated
OVA_378_C2	PRIMARY	chr18	56246114	56246114	C	T	ALPK2	exonic	ynonymou	000036167	548	104	652	0.159509	43	0	43	0	0.73	Sub	0.56	2.59			polymorphism	9.556	p.G632R	Passenger	SENGER/OT	benign	Neutral	Tolerated
OVA_378_C3	PRIMARY	chr18	56246114	56246114	C	T	ALPK2	exonic	ynonymou	000036167	521	116	637	0.182104	43	0	43	0	0.9	Sub	0.51	2.6			polymorphism	9.556	p.G632R	Passenger	SENGER/OT	benign	Neutral	Tolerated
OVA_378_M1	METASTASIS	chr18	56246135	56246135	C	G	ALPK2	exonic	ynonymou	000036167	483	163	646	0.252322	54	0	54	0	0.95	Clonal	0.72	2.57			polymorphism	15.94	p.E625Q	Passenger	SENGER/OT	damaging	Neutral	Damaging
OVA_378_M2	METASTASIS	chr18	56246135	56246135	C	G	ALPK2	exonic	ynonymou	000036167	449	178	627	0.283892	54	0	54	0	0.95	Clonal	0.85	2.57			polymorphism	15.94	p.E625Q	Passenger	SENGER/OT	damaging	Neutral	Damaging
OVA_378_M3	METASTASIS	chr18	56246135	56246135	C	G	ALPK2	exonic	ynonymou	000036167	528	193	721	0.267684	54	0	54	0	1	Clonal	0.67	2.57			polymorphism	15.94	p.E625Q	Passenger	SENGER/OT	damaging	Neutral	Damaging
OVA_378_C1	PRIMARY	chr18	56246135	56246135	C	G	ALPK2	exonic	ynonymou	000036167	499	141	640	0.220313	54	0	54	0	0.97	Sub	0.59	2.6			polymorphism	15.94	p.E625Q	Passenger	SENGER/OT	damaging	Neutral	Damaging
OVA_378_C2	PRIMARY	chr18	56246135	56246135	C	G	ALPK2	exonic	ynonymou	000036167	526	109	635	0.171654	54	0	54	0	0.78	Sub	0.56	2.59			polymorphism	15.94	p.E625Q	Passenger	SENGER/OT	damaging	Neutral	Damaging
OVA_378_C3	PRIMARY	chr18	56246135	56246135	C	G	ALPK2	exonic	ynonymou	000036167	504	122	626	0.194888	54	0	54	0	0.96	Sub	0.51	2.6			polymorphism	15.94	p.E625Q	Passenger	SENGER/OT	damaging	Neutral	Damaging
OVA_378_M1	METASTASIS	chr18	56246452	56246452	C	G	ALPK2	exonic	ynonymou	000036167	435	196	631	0.310618	83	0	83	0	1	Clonal	0.72	2.57			polymorphism	11.28	p.R519T	Passenger	SENGER/OT	benign	Deleterious	Damaging
OVA_378_M2	METASTASIS	chr18	56246452	56246452	C	G	ALPK2	exonic	ynonymou	000036167	461	201	662	0.303625	83	0	83	0	1	Clonal	0.85	2.57			polymorphism	11.28	p.R519T	Passenger	SENGER/OT	benign	Deleterious	Damaging
OVA_378_M3	METASTASIS	chr18	56246452	56246452	C	G	ALPK2	exonic	ynonymou	000036167	520	190	710	0.267606	83	0	83	0	1	Clonal	0.67	2.57			polymorphism	11.28	p.R519T	Passenger	SENGER/OT	benign	Deleterious	Damaging
OVA_378_C1	PRIMARY	chr18	56246452	56246452	C	G	ALPK2	exonic	ynonymou	000036167	529	173	702	0.246439	83	0	83	0	1	Clonal	0.59	2.6			polymorphism	11.28	p.R519T	Passenger	SENGER/OT	benign	Deleterious	Damaging
OVA_378_C2	PRIMARY	chr18	56246452	56246452	C	G	ALPK2	exonic	ynonymou	000036167	501	150	651	0.230415	83	0	83	0	1	Clonal	0.56	2.59			polymorphism	11.28	p.R519T	Passenger	SENGER/OT	benign	Deleterious	Damaging
OVA_378_C3	PRIMARY	chr18	56246452	56246452	C	G	ALPK2	exonic	ynonymou	000036167	498	143	641	0.223089	83	0	83	0	1	Clonal	0.51	2.6			polymorphism	11.28	p.R519T	Passenger	SENGER/OT	benign	Deleterious	Damaging
OVA_378_M1	METASTASIS	chr18	56246459	56246459	C	T	ALPK2	exonic	ynonymou	000036167	440	189	629	0.300477	82	0	82	0	1	Clonal	0.72	2.57			polymorphism	13.15	p.D517N	Passenger	SENGER/OT	benign	Neutral	Damaging
OVA_378_M2	METASTASIS	chr18	56246459	56246459	C	T	ALPK2	exonic	ynonymou	000036167	452	194	646	0.30031	82	0	82	0	1	Clonal	0.85	2.57			polymorphism	13.15	p.D517N	Passenger	SENGER/OT	benign	Neutral	Damaging
OVA_378_M3	METASTASIS	chr18	56246459	56246459	C	T	ALPK2	exonic	ynonymou	000036167	511	195	706	0.276204	82	0	82	0	1	Clonal	0.67	2.57			polymorphism	13.15	p.D517N	Passenger	SENGER/OT	benign	Neutral	Damaging
OVA_378_C1	PRIMARY	chr18	56246459	56246459	C	T	ALPK2	exonic	ynonymou	000036167	522	168	690	0.243478	82	0	82	0	1	Clonal	0.59	2.6			polymorphism	13.15	p.D517N	Passenger	SENGER/OT	benign	Neutral	Damaging
OVA_378_C2	PRIMARY	chr18	56246459	56246459	C	T	ALPK2	exonic	ynonymou	000036167	504	140	644	0.217391	82	0	82	0	0.99	Clonal	0.56	2.59			polymorphism	13.15	p.D517N	Passenger	SENGER/OT	benign	Neutral	Damaging
OVA_378_C3	PRIMARY	chr18	56246459	56246459	C	T	ALPK2	exonic	ynonymou	000036167	494	144	638	0.225705	82	0	82	0	1	Clonal	0.51	2.6			polymorphism	13.15	p.D517N	Passenger	SENGER/OT	benign	Neutral	Damaging
OVA_378_M1	METASTASIS	chr18	56246606	56246606	C	T	ALPK2	exonic	ynonymou	000036167	457	169	626	0.269968	59	0	59	0	1	Clonal	0.72	2.57			disease_causing	27.2	p.E468K	Passenger	SENGER/OT	damaging	Neutral	Tolerated
OVA_378_M2	METASTASIS	chr18	56246606	56246606	C	T	ALPK2	exonic	ynonymou	000036167	435	164	599	0.27379	59	0	59	0	0.92	Clonal	0.85	2.57			disease_causing	27.2	p.E468K	Passenger	SENGER/OT	damaging	Neutral	Tolerated
OVA_378_M3	METASTASIS	chr18	56246606	56246606	C	T	ALPK2	exonic	ynonymou	000036167	510	171	681	0.251101	59	0	59	0	1	Clonal	0.67	2.57			disease_causing	27.2	p.E468K	Passenger	SENGER/OT	damaging	Neutral	Tolerated
OVA_378_C1	PRIMARY	chr18	56246606	56246606	C	T	ALPK2	exonic	ynonymou	000036167	518	144	662	0.217523	59	0	59	0	0.96	Sub	0.59	2.6			disease_causing	27.2	p.E468K	Passenger	SENGER/OT	damaging	Neutral	Tolerated
OVA_378_C2	PRIMARY	chr18	56246606	56246606	C	T	ALPK2	exonic	ynonymou	000036167	488	109	597	0.18258	59	0	59	0	0.83	Sub	0.56	2.59			disease_causing	27.2	p.E468K	Passenger	SENGER/OT	damaging	Neutral	Tolerated
OVA_378_C3	PRIMARY	chr18	56246606	56246606	C	T	ALPK2	exonic	ynonymou	000036167	492	119	611	0.194763	59	0	59	0	0.96	Sub	0.51	2.6			disease_causing	27.2	p.E468K	Passenger	SENGER/OT	damaging	Neutral	Tolerated
OVA_378_M1	METASTASIS	chr18	56246636	56246636	C	T	ALPK2	exonic	ynonymou	000036167	452	158	610	0.259016	60	0	60	0	0.98	Clonal	0.72	2.57			polymorphism	23.8	p.A458T	Passenger	SENGER/OT	damaging	Neutral	Tolerated
OVA_378_M2	METASTASIS	chr18	56246636	56246636	C	T	ALPK2	exonic	ynonymou	000036167	411	174	585	0.297436	60	0	60	0	0.99	Clonal	0.85	2.57			polymorphism	23.8	p.A458T	Passenger	SENGER/OT	damaging	Neutral	Tolerated
OVA_378_M3	METASTASIS	chr18	56246636	56246636	C	T	ALPK2	exonic	ynonymou	000036167	513	182	695	0.261871	60	0	60	0	1	Clonal	0.67	2.57			polymorphism	23.8	p.A458T	Passenger	SENGER/OT	damaging	Neutral	Tolerated
OVA_378_C1	PRIMARY	chr18	56246636	56246636	C	T	ALPK2	exonic	ynonymou	000036167	537	130	667	0.194903	60	0	60	0	0.86	Clonal	0.59	2.6			polymorphism	23.8	p.A458T	Passenger	SENGER/OT	damaging	Neutral	Tolerated
OVA_378_C2	PRIMARY	chr18	56246636	56246636	C	T	ALPK2	exonic	ynonymou	000036167	494	121	615	0.196748	60	0	60	0	0.89	Clonal	0.56	2.59			polymorphism	23.8	p.A458T	Passenger	SENGER/OT	damaging	Neutral	Tolerated
OVA_378_C3	PRIMARY	chr18	56246636	56246636	C	T	ALPK2	exonic	ynonymou	000036167	495	118	613	0.192496	60	0	60	0	0.95	Clonal	0.51	2.6			polymorphism	23.8	p.A458T	Passenger	SENGER/OT	damaging	Neutral	Tolerated
OVA_378_M1	METASTASIS	chr18	56246783	56246783	C	G	ALPK2	exonic	ynonymou	000036167	401	149	550	0.270909	51	0	51	0	1	Clonal	0.72	2.57			polymorphism	15.99	p.E409Q	Passenger	SENGER/OT	damaging	Neutral	Damaging
OVA_378_M2	METASTASIS	chr18	56246783	56246783	C	G	ALPK2	exonic	ynonymou	000036167	393	155	548	0.282847	51	0	51	0	0.95	Clonal	0.85	2.57			polymorphism	15.99	p.E409Q	Passenger	SENGER/OT	damaging	Neutral	Damaging
OVA_378_M3	METASTASIS	chr18	56246783	56246783	C	G	ALPK2	exonic	ynonymou	000036167	450	143	593	0.241147	51	0	51	0	0.96	Clonal	0.67	2.57			polymorphism	15.99	p.E409Q	Passenger	SENGER/OT	damaging	Neutral	Damaging
OVA_378_C1	PRIMARY	chr18	56246783	56246783	C	G	ALPK2	exonic	ynonymou	000036167	446	137	583	0.234991	51	0	51	0	1	Clonal	0.59	2.6			polymorphism	15.99	p.E409Q	Passenger	SENGER/OT	damaging	Neutral	Damaging
OVA_378_C2	PRIMARY	chr18	56246783	56246783	C	G	ALPK2	exonic	ynonymou	000036167	421	119	540	0.22037	51	0	51	0	1	Clonal	0.56	2.59			polymorphism	15.99	p.E409Q	Passenger	SENGER/OT	damaging	Neutral	Damaging
OVA_378_C3	PRIMARY	chr18	56246783	56246783	C	G	ALPK2	exonic	ynonymou	000036167	438	111	549	0.202186	51	0	51	0	0.99	Clonal	0.51	2.6			polymorphism	15.99	p.E409Q	Passenger	SENGER/OT	damaging	Neutral	Damaging
OVA_365_M1	METASTASIS	chr4	56301603	56301603	A	T	CLOCK	exonic	ionymous S	000051344	355	274	629	0.435612	41	0	41	0	1	Clonal	0.85	1.87			disease_causing	15.92		Passenger			Neutral	Tolerated
OVA_365_M2	METASTASIS	chr4	56301603	56301603	A	T	CLOCK	exonic	ionymous S	000051344	390	104	494	0.210526	41	0	41	0	1	Clonal	0.4	1.81			disease_causing	15.92		Passenger			Neutral	Tolerated
OVA_365_M3	METASTASIS	chr4	56301603	56301603	A	T	CLOCK	exonic	ionymous S	000051344	380	224	604	0.370861	41	0	41	0	0.91	Clonal	0.82	1.89			disease_causing	15.92		Passenger			Neutral	Tolerated
OVA_003_M1	METASTASIS	chrX	56310780	56310780	C	G	KLF8	exonic	ynonymou	000046866	245	76	321	0.23676	40	0	40	0	0.84	Sub	0.57	1.84			disease_causing	25.9	p.C311W	Passenger	CANCER	damaging	Deleterious	Damaging
OVA_003_M2	METASTASIS	chrX	56310780	56310780	C	G	KLF8	exonic	ynonymou	000046866	316	61	377	0.161804	40	0	40	0	0.71	Sub	0.46	1.81			disease_causing	25.9	p.C311W	Passenger	CANCER	damaging	Deleterious	Damaging
OVA_003_M3	METASTASIS	chrX	56310780	56310780	C	G	KLF8	exonic	ynonymou	000046866	437	28	465	0.060215	40	0	40	0	0.57	Sub	0.34	2.67			disease_causing	25.9	p.C311W	Passenger	CANCER	damaging	Deleterious	Damaging
OVA_003_C1	PRIMARY	chrX	56310780	56310780	C	G	KLF8	exonic	ynonymou	000046866	249	161	410	0.392683	40	0	40	0	0.91	Clonal	0.86	1.81			disease_causing	25.9	p.C311W	Passenger	CANCER	damaging	Deleterious	Damaging
OVA_003_C2	PRIMARY	chrX	56310780	56310780	C	G	KLF8	exonic	ynonymou	000046866	298	133	431	0.308585	40	0	40	0	1	Clonal	0.7	3.61			disease_causing	25.9	p.C311W	Passenger	CANCER	damaging	Deleterious	Damaging
OVA_003_C3	PRIMARY	chrX	56310780	56310780	C	G	KLF8	exonic	ynonymou	000046866	292	177	469	0.377399	40	0	40	0	0.92	Clonal	0.82	1.81			disease_causing	25.9	p.C311W	Passenger	CANCER	damaging	Deleterious	Damaging
OVA_378_M1	METASTASIS	chr19	56424468	56424468	C	T	NLRP13	exonic	ynonymou	V239I,ENSG	413	157	570	0.275439	46	0	46	0	1	Clonal	0.72	2.57			polymorphism	15.75	p.V239I	Passenger	SENGER/OT	damaging	Neutral	Tolerated
OVA_378_M2	METASTASIS	chr19	56424468	56424468	C	T	NLRP13	exonic	ynonymou	V239I,ENSG	400	170	570	0.298246	46	0	46	0	1	Clonal	0.85	2.57			polymorphism	15.75	p.V239I	Passenger	SENGER/OT	damaging	Neutral	Tolerated
OVA_378_M3	METASTASIS	chr19	56424468	56424468	C	T	NLRP13	exonic	ynonymou	V239I,ENSG	457	131	588	0.222789	46	0	46	0	0.89	Clonal	0.67	2.57			polymorphism	15.75	p.V239I	Passenger	SENGER/OT	damaging	Neutral	Tolerated
OVA_378_C1	PRIMARY	chr19	56424468	56424468	C	T	NLRP13	exonic	ynonymou	V239I,ENSG	462	149	611	0.243863	46	0	46	0	1	Clonal	0.59	2.6			polymorphism	15.75	p.V239I	Passenger	SENGER/OT	damaging	Neutral	Tolerated
OVA_378_C2	PRIMARY	chr19	56424468	56424468	C	T	NLRP13	exonic	ynonymou	V239I,ENSG	414	137	551	0.248639	46	0	46	0	1	Clonal	0.56	2.59			polymorphism	15.75	p.V239I	Passenger	SENGER/OT	damaging	Neutral	Tolerated
OVA_378_C3	PRIMARY	chr19	56424468	56424468	C	T	NLRP13	exonic	ynonymou	V239I,ENSG	429	116	545	0.212844	46	0	46	0	1	Clonal	0.51	2.6			polymorphism	15.75	p.V239I	Passenger	SENGER/OT	damaging	Neutral	Tolerated
OVA_048_M1	METASTASIS	chr6	56716273	56716273	T	C	DST	exonic	ynonymou	000037075	194	111	305	0.363934	47	0	47	0	1	Clonal	0.56	3.94				23.3	p.K183E	Passenger		damaging	Neutral	Tolerated
OVA_048_M2	METASTASIS	chr6	56716273	56716273	T	C	DST	exonic	ynonymou	000037075	237	71	308	0.230519	47	0	47	0	1	Clonal	0.4	3.87				23.3	p.K183E	Passenger		damaging	Neutral	Tolerated

Sample	Type	Chr	Start	End	Ref	Alt	Gene	unc.ensGen	icFunc.ens	hange.ens	t_ref	t_alt	t_total	t_VAF	n_ref	n_alt	n_total	normal_VA	ncer_cell_f	on-based C	purity	ploidy	Cancer Gene	Driver Gene	Mutation Taster	ADD(PHRE	otein_chan	CHASM	ATHMM_Pr	OLYPHEN-	REDICTION	ICTION (cutoff=0.05)
OVA_048_M3	METASTASIS	chr6	56716273	56716273	T	C	DST	exonic	ynonymou	000037075	191	57	248	0.229839	47	0	47	0	1	Clonal	0.59	3.39				23.3	p.K183E	Passenger		damaging	Neutral	Tolerated
OVA_048_C1	PRIMARY	chr6	56716273	56716273	T	C	DST	exonic	ynonymou	000037075	133	64	197	0.324873	47	0	47	0	0.97	Clonal	0.67	1.82				23.3	p.K183E	Passenger		damaging	Neutral	Tolerated
OVA_048_C2	PRIMARY	chr6	56716273	56716273	T	C	DST	exonic	ynonymou	000037075	219	174	393	0.442748	47	0	47	0	1	Clonal	0.72	1.85				23.3	p.K183E	Passenger		damaging	Neutral	Tolerated
OVA_048_C3	PRIMARY	chr6	56716273	56716273	T	C	DST	exonic	ynonymou	000037075	127	91	218	0.417431	47	0	47	0	1	Clonal	0.74	1.8				23.3	p.K183E	Passenger		damaging	Neutral	Tolerated
OVA_378_M1	METASTASIS	chr18	56718890	56718890	G	A	OACYLP	cRNA_exon	NA	NA	468	192	660	0.290909	69	1	70	0.014286	1	Clonal	0.72	2.57				1.11						
OVA_378_M2	METASTASIS	chr18	56718890	56718890	G	A	OACYLP	cRNA_exon	NA	NA	458	194	652	0.297546	69	1	70	0.014286	0.99	Clonal	0.85	2.57				1.11						
OVA_378_M3	METASTASIS	chr18	56718890	56718890	G	A	OACYLP	cRNA_exon	NA	NA	527	194	721	0.269071	69	1	70	0.014286	1	Clonal	0.67	2.57				1.11						
OVA_378_C1	PRIMARY	chr18	56718890	56718890	G	A	OACYLP	cRNA_exon	NA	NA	510	179	689	0.259797	69	1	70	0.014286	1	Clonal	0.59	2.6				1.11						
OVA_378_C2	PRIMARY	chr18	56718890	56718890	G	A	OACYLP	cRNA_exon	NA	NA	523	145	668	0.217066	69	1	70	0.014286	0.99	Clonal	0.56	2.59				1.11						
OVA_378_C3	PRIMARY	chr18	56718890	56718890	G	A	OACYLP	cRNA_exon	NA	NA	497	141	638	0.221003	69	1	70	0.014286	1	Clonal	0.51	2.6				1.11						
OVA_048_C2	PRIMARY	chr15	56756431	56756431	T	A	MNS1	exonic	ynonymou	T00000260	206	136	342	0.397661	24	0	24	0	1	Clonal	0.72	1.85			polymorphism	16.28	p.R6S	Passenger	SENGER/OT	benign	Neutral	Damaging
OVA_003_M1	METASTASIS	chr20	56885101	56885101	C	T	RAB22A	intronic	NA	NA	39	8	47	0.170213	42	0	42	0	0.77	Sub	0.57	1.84			polymorphism	11.26						
OVA_003_M2	METASTASIS	chr20	56885101	56885101	C	T	RAB22A	intronic	NA	NA	39	4	43	0.093023	42	0	42	0	0.5	Sub	0.46	1.81			polymorphism	11.26						
OVA_003_C1	PRIMARY	chr20	56885101	56885101	C	T	RAB22A	intronic	NA	NA	26	1	27	0.037037	42	0	42	0	0.12	Sub	0.86	1.81			polymorphism	11.26						
OVA_003_C2	PRIMARY	chr20	56885101	56885101	C	T	RAB22A	intronic	NA	NA	18	9	27	0.333333	42	0	42	0	1	Sub	0.7	3.61			polymorphism	11.26						
OVA_003_C3	PRIMARY	chr20	56885101	56885101	C	T	RAB22A	intronic	NA	NA	15	8	23	0.347826	42	0	42	0	1	Sub	0.82	1.81			polymorphism	11.26						
OVA_378_C3	PRIMARY	chr11	57003757	57003757	C	T	APLNR	exonic	ynonymou	241Q,ENSG	427	112	539	0.207792	134	1	135	0.007407	1	Sub	0.51	2.6			disease_causing	24.8	p.R241Q	Passenger		damaging	Neutral	Tolerated
OVA_378_C1	PRIMARY	chr11	57003757	57003757	C	T	APLNR	exonic	ynonymou	241Q,ENSG	537	9	546	0.016484	134	1	135	0.007407	0.06	Sub	0.59	2.6			disease_causing	24.8	p.R241Q	Passenger		damaging	Neutral	Tolerated
OVA_003_M1	METASTASIS	chr11	57145276	57145276	T	C	PRG3	exonic	ynonymou	000028714	247	93	340	0.273529	46	0	46	0	0.97	Sub	0.57	1.84			polymorphism	22.6	p.S181G	Passenger	SENGER/OT	damaging	Deleterious	Damaging
OVA_003_M2	METASTASIS	chr11	57145276	57145276	T	C	PRG3	exonic	ynonymou	000028714	359	88	447	0.196868	46	0	46	0	0.86	Sub	0.46	1.81			polymorphism	22.6	p.S181G	Passenger	SENGER/OT	damaging	Deleterious	Damaging
OVA_003_M3	METASTASIS	chr11	57145276	57145276	T	C	PRG3	exonic	ynonymou	000028714	450	19	469	0.040512	46	0	46	0	0.43	Sub	0.34	2.67			polymorphism	22.6	p.S181G	Passenger	SENGER/OT	damaging	Deleterious	Damaging
OVA_003_C1	PRIMARY	chr11	57145276	57145276	T	C	PRG3	exonic	ynonymou	000028714	292	162	454	0.356828	46	0	46	0	0.83	Sub	0.86	1.81			polymorphism	22.6	p.S181G	Passenger	SENGER/OT	damaging	Deleterious	Damaging
OVA_003_C2	PRIMARY	chr11	57145276	57145276	T	C	PRG3	exonic	ynonymou	000028714	306	191	497	0.384306	46	0	46	0	1	Sub	0.7	3.61			polymorphism	22.6	p.S181G	Passenger	SENGER/OT	damaging	Deleterious	Damaging
OVA_003_C3	PRIMARY	chr11	57145276	57145276	T	C	PRG3	exonic	ynonymou	000028714	331	178	509	0.349705	46	0	46	0	0.85	Sub	0.82	1.81			polymorphism	22.6	p.S181G	Passenger	SENGER/OT	damaging	Deleterious	Damaging
OVA_378_M1	METASTASIS	chr12	57409651	57409652	AC	-	TAC3	intronic	NA	NA	850	251	1101	0.227975	51	0	51		0.86	Sub	0.72	2.57										
OVA_378_M2	METASTASIS	chr12	57409651	57409652	AC	-	TAC3	intronic	NA	NA	874	288	1162	0.247849	51	0	51		0.83	Sub	0.85	2.57										
OVA_378_M3	METASTASIS	chr12	57409651	57409652	AC	-	TAC3	intronic	NA	NA	816	256	1072	0.238806	51	0	51		0.95	Sub	0.67	2.57										
OVA_378_C1	PRIMARY	chr12	57409651	57409652	AC	-	TAC3	intronic	NA	NA	987	264	1251	0.211031	51	0	51		0.93	Clonal	0.59	2.6										
OVA_378_C2	PRIMARY	chr12	57409651	57409652	AC	-	TAC3	intronic	NA	NA	788	225	1013	0.222113	51	0	51		1	Clonal	0.56	2.59										
OVA_378_M1	METASTASIS	chr6	57498895	57498895	C	T	PRIM2	intronic	NA	NA	481	75	556	0.134892	32	0	32	0	0.37	Sub	0.72	2.57				5.536						
OVA_378_M2	METASTASIS	chr6	57498895	57498895	C	T	PRIM2	intronic	NA	NA	488	71	559	0.127013	32	0	32	0	0.3	Sub	0.85	2.57				5.536						
OVA_378_M3	METASTASIS	chr6	57498895	57498895	C	T	PRIM2	intronic	NA	NA	521	90	611	0.1473	32	0	32	0	0.44	Sub	0.67	2.57				5.536						
OVA_378_C1	PRIMARY	chr6	57498895	57498895	C	T	PRIM2	intronic	NA	NA	498	98	596	0.16443	32	0	32	0	0.56	Sub	0.59	2.6				5.536						
OVA_378_C2	PRIMARY	chr6	57498895	57498895	C	T	PRIM2	intronic	NA	NA	452	110	562	0.19573	32	0	32	0	0.69	Sub	0.56	2.59				5.536						
OVA_378_C3	PRIMARY	chr6	57498895	57498895	C	T	PRIM2	intronic	NA	NA	484	77	561	0.137255	32	0	32	0	0.54	Sub	0.51	2.6				5.536						
OVA_048_M1	METASTASIS	chr11	57583102	57583102	G	T	D1;TMX2-C	UTR3	NA	NA	338	201	539	0.372913	64	1	65	0.015385	1	Clonal	0.56	3.94			polymorphism	8.929						
OVA_048_M2	METASTASIS	chr11	57583102	57583102	G	T	D1;TMX2-C	UTR3	NA	NA	361	142	503	0.282306	64	1	65	0.015385	1	Clonal	0.4	3.87			polymorphism	8.929						
OVA_048_M3	METASTASIS	chr11	57583102	57583102	G	T	D1;TMX2-C	UTR3	NA	NA	339	186	525	0.354286	64	1	65	0.015385	1	Clonal	0.59	3.39			polymorphism	8.929						
OVA_048_C1	PRIMARY	chr11	57583102	57583102	G	T	D1;TMX2-C	UTR3	NA	NA	267	118	385	0.306494	64	1	65	0.015385	0.92	Clonal	0.67	1.82			polymorphism	8.929						
OVA_048_C2	PRIMARY	chr11	57583102	57583102	G	T	D1;TMX2-C	UTR3	NA	NA	342	201	543	0.370166	64	1	65	0.015385	1	Clonal	0.72	1.85			polymorphism	8.929						
OVA_048_C3	PRIMARY	chr11	57583102	57583102	G	T	D1;TMX2-C	UTR3	NA	NA	242	151	393	0.384224	64	1	65	0.015385	1	Clonal	0.74	1.8			polymorphism	8.929						
OVA_003_M1	METASTASIS	chr20	57598760	57598760	G	C	TUBB1	exonic	ynonymou	000002171	311	211	522	0.404215	98	0	98	0	1	Clonal	0.57	1.84			disease_causing	27.6	p.G93A	Passenger	SENGER/OT	damaging	Deleterious	Damaging
OVA_003_M2	METASTASIS	chr20	57598760	57598760	G	C	TUBB1	exonic	ynonymou	000002171	364	198	562	0.352313	98	0	98	0	1	Clonal	0.46	1.81			disease_causing	27.6	p.G93A	Passenger	SENGER/OT	damaging	Deleterious	Damaging
OVA_003_M3	METASTASIS	chr20	57598760	57598760	G	C	TUBB1	exonic	ynonymou	000002171	467	68	535	0.127103	98	0	98	0	1	Clonal	0.34	2.67			disease_causing	27.6	p.G93A	Passenger	SENGER/OT	damaging	Deleterious	Damaging
OVA_003_C1	PRIMARY	chr20	57598760	57598760	G	C	TUBB1	exonic	ynonymou	000002171	259	282	541	0.521257	98	0	98	0	1	Clonal	0.86	1.81			disease_causing	27.6	p.G93A	Passenger	SENGER/OT	damaging	Deleterious	Damaging
OVA_003_C2	PRIMARY	chr20	57598760	57598760	G	C	TUBB1	exonic	ynonymou	000002171	267	314	581	0.540448	98	0	98	0	1	Clonal	0.7	3.61			disease_causing	27.6	p.G93A	Passenger	SENGER/OT	damaging	Deleterious	Damaging
OVA_003_C3	PRIMARY	chr20	57598760	57598760	G	C	TUBB1	exonic	ynonymou	000002171	268	306	574	0.533101	98	0	98	0	1	Clonal	0.82	1.81			disease_causing	27.6	p.G93A	Passenger	SENGER/OT	damaging	Deleterious	Damaging
OVA_048_C2	PRIMARY	chr20	57617873	57617873	C	T	LMO2-ATP5	UTR5	NA	NA	10	5	15	0.333333	21	0	21	0	0.93	Clonal	0.72	1.85			polymorphism	6.696						
OVA_047_C1	PRIMARY	chr17	57746330	57746330	G	T	CLTC	intronic	NA	NA	341	259	600	0.431667	30	0	30	0	1	Clonal	0.6	2.91	Yes		polymorphism	4.663						
OVA_047_C2	PRIMARY	chr17	57746330	57746330	G	T	CLTC	intronic	NA	NA	323	276	599	0.460768	30	0	30	0	0.98	Clonal	0.64	2.76	Yes		polymorphism	4.663						
OVA_047_C3	Contralateral ovary	chr17	57746330	57746330	G	T	CLTC	intronic	NA	NA	338	252	590	0.427119	30	0	30	0	1	Clonal	0.6	2.94	Yes		polymorphism	4.663						
OVA_047_C1	PRIMARY	chr17	57746331	57746331	G	T	CLTC	intronic	NA	NA	341	261	602	0.433555	29	0	29	0	1	Clonal	0.6	2.91	Yes		polymorphism	6.012						
OVA_047_C2	PRIMARY	chr17	57746331	57746331	G	T	CLTC	intronic	NA	NA	326	275	601	0.457571	29	0	29	0	0.97	Clonal	0.64	2.76	Yes		polymorphism	6.012						
OVA_047_C3	Contralateral ovary	chr17	57746331	57746331	G	T	CLTC	intronic	NA	NA	336	249	585	0.425641	29	0	29	0	1	Clonal	0.6	2.94	Yes		polymorphism	6.012						
OVA_003_M1	METASTASIS	chr16	57849581	57849581	C	G	C3;LOC388	intronic	NA	NA	274	194	468	0.41453	64	0	64	0	1	Clonal	0.57	1.84				1.565						
OVA_003_M2	METASTASIS	chr16	57849581	57849581	C	G	C3;LOC388	intronic	NA	NA	345	166	511	0.324853	64	0	64	0	1	Clonal	0.46	1.81				1.565						
OVA_003_M3	METASTASIS	chr16	57849581	57849581	C	G	C3;LOC388	intronic	NA	NA	393	69	462	0.149351	64	0	64	0	1	Clonal	0.34	2.67				1.565						
OVA_003_C1	PRIMARY	chr16	57849581	57849581	C	G	C3;LOC388	intronic	NA	NA	263	236	499	0.472946	64	0	64	0	1	Clonal	0.86	1.81				1.565						
OVA_003_C2	PRIMARY	chr16	57849581	57849581	C	G	C3;LOC388	intronic	NA	NA	271	258	529	0.487713	64	0	64	0	1	Clonal	0.7	3.61				1.565						
OVA_003_C3	PRIMARY	chr16	57849581	57849581	C	G	C3;LOC388	intronic	NA	NA	329	217	546	0.397436	64	0	64	0	0.96	Clonal	0.82	1.81				1.565						
OVA_048_M2	METASTASIS	chr16	57989136	57989136	G	A	CNGB1	UTR3	NA	NA	305	15	320	0.046875	20	0	20	0	0.33	Sub	0.4	3.87			polymorphism	6.021						
OVA_048_M3	METASTASIS	chr16	57989136	57989136	G	A	CNGB1	UTR3	NA	NA	206	44	250	0.176	20	0	20	0	1	Sub	0.59	3.39			polymorphism	6.021						
OVA_048_M2	METASTASIS	chr16	57989148	57989148	G	C	CNGB1	UTR3	NA	NA	312	15	327	0.045872	23	0	23	0	0.32	Sub	0.4	3.87			polymorphism	10.46						
OVA_048_M3	METASTASIS	chr16	57989148	57989148	G	C	CNGB1	UTR3	NA	NA	218	42	260	0.161538	23	0	23	0	1	Sub	0.59	3.39			polymorphism	10.46						
OVA_048_M1	METASTASIS	chr12	58007183	58007183	G	A	BC073932	cRNA_exon	NA	NA	424	8	433	0.018476	101	0	101	0	0.07	Sub	0.59	3.39			polymorphism	10.41						
OVA_048_M3	METASTASIS	chr12	58007183	58007183	G	A	BC073932	cRNA_exon	NA	NA	233	50	283	0.176678	101	0	101	0	1	Sub	0.59	3.39			polymorphism	10.41						
OVA_048_C2	PRIMARY	chr20	58318231	58318231	G	T	PHACTR3	exonic	ynonymou	000003710	114	82	196	0.418367	23	0	23	0	1	Clonal	0.72	1.85			disease_causing	27.1	p.R63M	Passenger	SENGER/OT	damaging	Deleterious	Damaging
OVA_378_C1	PRIMARY	chr20	58348415	58348415	C	A	PHACTR3	exonic	ynonymou	000035992	195	55	250	0.22	62	0	62	0	0.97	Clonal	0.59	2.6			disease_causing	25	p.S278Y	Passenger	SENGER/OT	damaging	Deleterious	Damaging
OVA_013_M1	METASTASIS	chr19	58598786	58598786	A	T	ZSCAN18	intronic	NA	NA	47	26	73	0.356164	18	0	18	0	1	Clonal	0.67	3.06			polymorphism	2.325						
OVA_013_C1	PRIMARY	chr19	58598786	58598786	A	T	ZSCAN18	intronic	NA	NA	60	24	84	0.285714	18	0	18	0	0.79	Sub	0.72	3.1			polymorphism	2.325						
OVA_013_C2	PRIMARY	chr19	58598786	58598786	A	T	ZSCAN18	intronic	NA	NA	65	32	97	0.329897	18	0	18	0	0.76	Sub	0.87	3.05			polymorphism	2.325						
OVA_013_C3	PRIMARY	chr19	58598786	58598786	A	T	ZSCAN18	intronic	NA	NA	62	48	110	0.436364	18	0	18	0	0.95	Sub	0.91	3.01			polymorphism	2.325						
OVA_047_C1	PRIMARY	chr14	58796251	58796251	C	A	ARID4A	exonic	ynonymou	000348476	395	118	513	0.230019	39	0	39	0	0.77	Sub	0.6	2.91			disease_causing	26.7	p.S223Y	Passenger	SENGER/OT	damaging	Deleterious	Damaging
OVA_047_C2	PRIMARY	chr14	58796251	58796251	C	A	ARID4A	exonic	ynonymou	000348476	309	123	432	0.284722	39	0	39	0	0.89	Sub	0.64	2.76			disease_causing	26.7	p.S223Y	Passenger	SENGER/OT	damaging	Deleterious	Damaging
OVA_047_C3	Contralateral ovary	chr14	58796251	58796251	C	A	ARID4A	exonic	ynonymou	000348476	317	114	431	0.264501	39	0	39	0	0.89	Clonal	0.6	2.94			disease_causing	26.7	p.S223Y	Passenger	SENGER/OT	damaging	Deleterious	Damaging
OVA_003_M1	METASTASIS	chr19	59011046	59011046	G	T	SLC27A5	exonic	ynonymou	494M,ENSG	80	35	115	0.304348	79	0	79	0	1	Sub	0.57	1.84			polymorphism	22.8	p.L494M	Passenger	SENGER/OT	damaging	Neutral	Damaging
OVA_003_M2	METASTASIS	chr19	59011046	59011046	G	T	SLC27A5	exonic	ynonymou	494M,ENSG	146	35	181	0.19337	79	0	79	0	0.84	Sub	0.46	1.81			polymorphism	22.8	p.L494M	Passenger	SENGER/OT	damaging	Neutral	Damaging
OVA_003_M3	METASTASIS	chr19	59011046	59011046	G	T	SLC27A5	exonic	ynonymou	494M,ENSG	176	9	185	0.048649	79	0	79	0	0.51	Sub	0.34	2.67			polymorphism	22.8	p.L494M	Passenger	SENGER/OT	damaging	Neutral	Damaging
OVA_003_C1	PRIMARY	chr19	59011046	59011046	G	T	SLC27A5	exonic	ynonymou	494M,ENSG	113	80	193	0.414508	79	0	79	0	0.96	Clonal	0.86	1.81			polymorphism	22.8	p.L494M	Passenger	SENGER/OT	damaging	Neutral	Damaging
OVA_003_C2	PRIMARY	chr19	59011046	59011046	G	T	SLC27A5	exonic	ynonymou	494M,ENSG	126	107	233	0.459227	79	0	79	0	1	Clonal	0.7	3.61			polymorphism	22.8	p.L494M	Passenger	SENGER/OT	damaging	Neutral	Damaging
OVA_003_C3	PRIMARY	chr19	59011046	59011046	G	T	SLC27A5	exonic	ynonymou	494M,ENSG	121	78	199	0.39196	79	0	79	0	0.95	Clonal	0.82	1.81			polymorphism	22.8	p.L494M	Passenger	SENGER/OT	damaging	Neutral	Damaging
OVA_047_C1	PRIMARY	chr11	59210685	59210685	T	C	OR5A1	exonic	ynonymou	T00000302	496	136	632	0.21519	51	0	51	0	0.93	Clonal	0.6	2.91			disease_causing	25.3	p.F15S	Passenger	SENGER/OT	damaging	Deleterious	Damaging
OVA_047_C2	PRIMARY	chr11	59210685	59210685	T	C	OR5A1	exonic	ynonymou	T00000302	504	130	634	0.205047	51	0	51	0	1	Clonal	0.64	2.76			disease_causing	25.3	p.F15S	Passenger	SENGER/OT	damaging	Deleterious	Damaging
OVA_047_C3	Contralateral ovary	chr11	59210685	59210685	T	C	OR5A1	exonic	ynonymou	T00000302	451	148	599	0.247078	51	0	51	0	1	Clonal	0.6	2.94			disease_causing	25.3	p.F15S	Passenger	SENGER/OT	damaging	Deleterious	Damaging
OVA_048_M1	METASTASIS	chr8	59535979	59535979	T	A	NSMAF	exonic	ynonymou	H154L,ENSG	456	123	579	0.212435	40	0	40	0	1	Clonal	0.56	3.94			disease_causing	24.1	p.H185L	Passenger	SENGER/OT	benign	Deleterious	Tolerated
OVA_048_M2	METASTASIS	chr8	59535979	59535979	T	A	NSMAF	exonic	ynonymou	H154L,ENSG	456	118	574	0.205575	40	0	40	0	1	Clonal	0.4	3.87			disease_causing	24.1	p.H185L	Passenger	SENGER/OT	benign	Deleterious	Tolerated
OVA_048_M3	METASTASIS	chr8	59535979	59535979	T	A	NSMAF	exonic	ynonymou	H154L,ENSG	374	182	556	0.327338	40	0	40	0	1	Clonal	0.59	3.39			disease_causing	24.1	p.H185L	Passenger	SENGER/OT	benign	Deleterious	Tolerated
OVA_003_M1	METASTASIS	chr11	60231826	60231826	C	A	MS4A1	intronic	NA	NA	376	110	486	0.226337	32	1	33	0.030303	0.8	Sub	0.57	1.84			polymorphism	11.98						
OVA_003_M2	METASTASIS	chr11	60231826	60231826	C	A	MS4A1	intronic	NA	NA	429	88	517	0.170213	32	1	33	0.030303	0.74	Sub	0.46	1.81			polymorphism	11.98						
OVA_003_M3	METASTASIS	chr11	60231826	60231826	C	A	MS4A1	intronic	NA	NA	554	30	584	0.05137	32	1	33	0.030303	0.54	Sub	0.34	2.67			polymorphism	11.98						
OVA_003_C1	PRIMARY	chr11	60231826	60231826	C	A	MS4A1	intronic	NA	NA	321	225	546	0.412088	32	1	33	0.030303	0.96	Clonal	0.86	1.81			polymorphism	11.98						
OVA_003_C2	PRIMARY	chr11	60231826	60231826	C	A	MS4A1	intronic	NA	NA	345	209	554	0.377256	32	1	33	0.030303	1	Clonal	0.7	3.61			polymorphism	11.98						
OVA_003_C3	PRIMARY	chr11	60231826	60231826	C	A	MS4A1	intronic	NA	NA	324	237	561	0.42246	32	1	33	0.030303	1	Clonal	0.82	1.81			polymorphism	11.98						
OVA_047_M1	METASTASIS	chr1	60280614	60280625	AGGGGGT	-	HOOK1	UTR5	NA	NA	23	38	61	0.622951	27	0	27		1	Sub	0.47	2.01										
OVA_047_M2	METASTASIS	chr1	60280614	60280625	AGGGGGT	-	HOOK1	UTR5	NA	NA	21	48	69	0.695652	27	0	27		1	Sub	0.44	2.01										
OVA_047_M3	METASTASIS	chr1	60280614	60280625	AGGGGGT	-	HOOK1	UTR5	NA	NA	10	14	24	0.583333	27	0	27		1	Sub	0.6	3.02										
OVA_047_M1	METASTASIS	chr11	60468827	60468827	C	A	MS4A8	UTR3	NA	NA	428	156	584	0.267123	33	0	33	0	1	Clonal	0.47	2.01			polymorphism	0.514						
OVA_047_M2	METASTASIS	chr11	60468827	60468827	C	A	MS4A8	UTR3	NA	NA	453	137	590	0.232203	33	0	33	0	1	Clonal	0.44	2.01			polymorphism	0.514						
OVA_047_M3	METASTASIS	chr11	60468827	60468827	C	A	MS4A8	UTR3	NA	NA	190	58	248	0.233871	33	0	33	0	1	Clonal	0.6	3.02			polymorphism	0.514						
OVA_047_C3	Contralateral ovary	chr20	60921254	60921254	C	T	LAMA5	exonic	ynonymou	000370677	396	109	505	0.215842	55	0	55	0	0.94	Clonal	0.6	2.94			polymorphism	8.911	p.D434N	Passenger	SENGER/OT	benign	Neutral	Tolerated

Sample	Type	Chr	Start	End	Ref	Alt	Gene	unc.ensGen	icFunc.ens	hange.ens	t_ref	t_alt	t_total	t_VAF	n_ref	n_alt	n_total	normal_VA	ncer_cell_f	on-based C	purity	ploidy	Cancer Gene	Driver Gene	Mutation Taster	ADD(PHRE	otein_chan	CHASM	ATHMM_Pr	OLYPHEN-	REDICTION	ICTION (cutoff=0.05)
OVA_048_M1	METASTASIS	chr14	60928078	60928078	C	-	C14orf39	exonic	meshift dele	000321731:	118	54	172	0.313953	45	0	45		1	Clonal	0.56	3.94					p.D371Ifs	Passenger			NA	NA
OVA_048_M2	METASTASIS	chr14	60928078	60928078	C	-	C14orf39	exonic	meshift dele	000321731:	132	41	173	0.236994	45	0	45		1	Clonal	0.4	3.87					p.D371Ifs	Passenger			NA	NA
OVA_048_M3	METASTASIS	chr14	60928078	60928078	C	-	C14orf39	exonic	meshift dele	000321731:	268	35	173	0.236994	45	0	45		1	Clonal	0.59	3.39										
OVA_047_M1	METASTASIS	chr14	60933669	60933669	T	G	C14orf39	exonic	nonymous S	000032173	429	190	619	0.306947	47	0	47	0	0.99	Clonal	0.47	2.01			polymorphism	1.227		Passenger			Neutral	Tolerated
OVA_047_M2	METASTASIS	chr14	60933669	60933669	T	G	C14orf39	exonic	nonymous S	000032173	429	188	617	0.3047	47	0	47	0	1	Clonal	0.44	2.01			polymorphism	1.227		Passenger			Neutral	Tolerated
OVA_047_M3	METASTASIS	chr14	60933669	60933669	T	G	C14orf39	exonic	nonymous S	000032173	200	82	282	0.29078	47	0	47	0	0.97	Clonal	0.6	3.02			polymorphism	1.227		Passenger			Neutral	Tolerated
OVA_047_C1	PRIMARY	chr11	61026201	61026201	A	C	VWCE	exonic	nonymous S	403P,ENSG	122	37	159	0.232704	52	1	53	0.018868	1	Sub	0.6	2.91			polymorphism	0.024		Passenger			Neutral	Tolerated
OVA_047_C2	PRIMARY	chr11	61026201	61026201	A	C	VWCE	exonic	nonymous S	403P,ENSG	90	46	136	0.338235	52	1	53	0.018868	1	Sub	0.64	2.76			polymorphism	0.024		Passenger			Neutral	Tolerated
OVA_047_C3	Contralateral ovary	chr11	61026201	61026201	A	C	VWCE	exonic	nonymous S	403P,ENSG	100	20	120	0.166667	52	1	53	0.018868	0.73	Sub	0.6	2.94			polymorphism	0.024		Passenger			Neutral	Tolerated
OVA_048_C2	PRIMARY	chr20	61143781	61143781	G	A	20orf166-A	cRNA_exon	NA	NA	225	13	238	0.054622	43	0	43	0	0.15	Sub	0.72	1.85				2.606				damaging		
OVA_047_M1	METASTASIS	chr18	61328261	61328261	A	T	PINB3;SERP	intronic	NA	NA	444	272	716	0.379888	71	0	71	0	1	Clonal	0.47	2.01			polymorphism	0.477						
OVA_047_M2	METASTASIS	chr18	61328261	61328261	A	T	PINB3;SERP	intronic	NA	NA	495	244	739	0.330176	71	0	71	0	1	Clonal	0.44	2.01			polymorphism	0.477						
OVA_047_M3	METASTASIS	chr18	61328261	61328261	A	T	PINB3;SERP	intronic	NA	NA	407	206	613	0.336052	71	0	71	0	1	Clonal	0.6	3.02			polymorphism	0.477						
OVA_047_C1	PRIMARY	chr18	61328261	61328261	A	T	PINB3;SERP	intronic	NA	NA	284	333	617	0.539708	71	0	71	0	1	Clonal	0.6	2.91			polymorphism	0.477						
OVA_047_C2	PRIMARY	chr18	61328261	61328261	A	T	PINB3;SERP	intronic	NA	NA	252	363	615	0.590244	71	0	71	0	1	Clonal	0.64	2.76			polymorphism	0.477						
OVA_047_C3	Contralateral ovary	chr18	61328261	61328261	A	T	PINB3;SERP	intronic	NA	NA	253	404	657	0.614916	71	0	71	0	1	Clonal	0.6	2.94			polymorphism	0.477						
OVA_047_M1	METASTASIS	chr20	61638072	61638072	C	A	BHLHE23	exonic	stopgain	ST0000037	14	8	22	0.363636	16	0	16	0	1	Clonal	0.47	2.01			disease_causing	36	p.E19X	Passenger			NA	NA
OVA_047_M2	METASTASIS	chr20	61638072	61638072	C	A	BHLHE23	exonic	stopgain	ST0000037	20	6	26	0.230769	16	0	16	0	1	Clonal	0.44	2.01			disease_causing	36	p.E19X	Passenger			NA	NA
OVA_047_M3	METASTASIS	chr20	61638072	61638072	C	A	BHLHE23	exonic	stopgain	ST0000037	7	3	10	0.3	16	0	16	0	1	Clonal	0.6	3.02			disease_causing	36	p.E19X	Passenger			NA	NA
OVA_048_M1	METASTASIS	chr10	61868056	61868056	C	G	ANK3	intronic	NA	NA	490	47	537	0.087523	31	0	31	0	0.49	Sub	0.56	3.94			disease_causing	23						
OVA_365_M1	METASTASIS	chr20	61937264	61937264	G	C	COL20A1	exonic	ynonymous	R123S,ENSG	30	27	57	0.473684	55	0	55	0	1	Clonal	0.85	1.87			polymorphism	14.98	p.R123S	Passenger	SENGER/OT	damaging	Neutral	Damaging
OVA_365_M2	METASTASIS	chr20	61937264	61937264	G	C	COL20A1	exonic	ynonymous	R123S,ENSG	16	10	26	0.384615	55	0	55	0	1	Clonal	0.4	1.81			polymorphism	14.98	p.R123S	Passenger	SENGER/OT	damaging	Neutral	Damaging
OVA_365_M3	METASTASIS	chr20	61937264	61937264	G	C	COL20A1	exonic	ynonymous	R123S,ENSG	28	18	46	0.391304	55	0	55	0	0.96	Clonal	0.82	1.89			polymorphism	14.98	p.R123S	Passenger	SENGER/OT	damaging	Neutral	Damaging
OVA_365_C1	Contralateral ovary	chr20	61937264	61937264	G	C	COL20A1	exonic	ynonymous	R123S,ENSG	23	9	32	0.28125	55	0	55	0	1	Clonal	0.42	1.83			polymorphism	14.98	p.R123S	Passenger	SENGER/OT	damaging	Neutral	Damaging
OVA_365_C2	PRIMARY	chr20	61937264	61937264	G	C	COL20A1	exonic	ynonymous	R123S,ENSG	30	12	42	0.285714	55	0	55	0	0.93	Clonal	0.62	1.92			polymorphism	14.98	p.R123S	Passenger	SENGER/OT	damaging	Neutral	Damaging
OVA_047_C1	PRIMARY	chr20	61939020	61939020	G	T	COL20A1	intronic	NA	NA	88	30	118	0.254237	34	0	34	0	1	Clonal	0.6	2.91			polymorphism	0.563						
OVA_047_C2	PRIMARY	chr20	61939020	61939020	G	T	COL20A1	intronic	NA	NA	89	27	116	0.232759	34	0	34	0	0.96	Clonal	0.64	2.76			polymorphism	0.563						
OVA_047_C3	Contralateral ovary	chr20	61939020	61939020	G	T	COL20A1	intronic	NA	NA	67	26	93	0.27957	34	0	34	0	1	Clonal	0.6	2.94			polymorphism	0.563						
OVA_047_C1	PRIMARY	chr20	62200122	62200122	C	G	HELZ2	exonic	ynonymous	000046714	93	25	118	0.211864	34	0	34	0	0.92	Clonal	0.6	2.91			polymorphism	7.371	p.S440T	Passenger	SENGER/OT	benign	Neutral	Tolerated
OVA_047_C2	PRIMARY	chr20	62200122	62200122	C	G	HELZ2	exonic	ynonymous	000046714	98	35	133	0.263158	34	0	34	0	1	Clonal	0.64	2.76			polymorphism	7.371	p.S440T	Passenger	SENGER/OT	benign	Neutral	Tolerated
OVA_047_C3	Contralateral ovary	chr20	62200122	62200122	C	G	HELZ2	exonic	ynonymous	000046714	84	21	105	0.2	34	0	34	0	0.87	Clonal	0.6	2.94			polymorphism	7.371	p.S440T	Passenger	SENGER/OT	benign	Neutral	Tolerated
OVA_365_M1	METASTASIS	chr11	62458183	62458183	T	G	RNPUL2-BS	exonic	ynonymous	T000004496	188	120	308	0.38961	95	0	95	0	0.92	Clonal	0.85	1.87			polymorphism	0.829					Neutral	Tolerated
OVA_365_M2	METASTASIS	chr11	62458183	62458183	T	G	RNPUL2-BS	exonic	ynonymous	T000004496	161	44	205	0.214634	95	0	95	0	1	Clonal	0.4	1.81			polymorphism	0.829					Neutral	Tolerated
OVA_365_M3	METASTASIS	chr11	62458183	62458183	T	G	RNPUL2-BS	exonic	ynonymous	T000004496	169	92	261	0.35249	95	0	95	0	0.86	Clonal	0.82	1.89			polymorphism	0.829					Neutral	Tolerated
OVA_365_C1	Contralateral ovary	chr11	62458183	62458183	T	G	RNPUL2-BS	exonic	ynonymous	T000004496	201	59	260	0.226923	95	0	95	0	1	Clonal	0.42	1.83			polymorphism	0.829					Neutral	Tolerated
OVA_365_C2	PRIMARY	chr11	62458183	62458183	T	G	RNPUL2-BS	exonic	ynonymous	T000004496	214	86	300	0.286667	95	0	95	0	0.93	Clonal	0.62	1.92			polymorphism	0.829					Neutral	Tolerated
OVA_365_M2	METASTASIS	chr17	62502375	62502375	G	A	DDX5	UTR5	NA	NA	144	49	193	0.253886	91	0	91	0	1	Clonal	0.4	1.81	Yes		polymorphism	7.027						
OVA_365_M1	METASTASIS	chr11	62572875	62572875	G	A	NXF1	UTR5	NA	NA	208	145	353	0.410765	161	1	162	0.006173	0.97	Clonal	0.85	1.87			polymorphism	4.46						
OVA_365_M2	METASTASIS	chr11	62572875	62572875	G	A	NXF1	UTR5	NA	NA	193	61	254	0.240157	161	1	162	0.006173	1	Clonal	0.4	1.81			polymorphism	4.46						
OVA_365_M3	METASTASIS	chr11	62572875	62572875	G	A	NXF1	UTR5	NA	NA	145	130	275	0.472727	161	1	162	0.006173	1	Clonal	0.82	1.89			polymorphism	4.46						
OVA_365_C1	Contralateral ovary	chr11	62572875	62572875	G	A	NXF1	UTR5	NA	NA	212	91	303	0.30033	161	1	162	0.006173	1	Clonal	0.42	1.83			polymorphism	4.46						
OVA_365_C2	PRIMARY	chr11	62572875	62572875	G	A	NXF1	UTR5	NA	NA	277	130	407	0.31941	161	1	162	0.006173	1	Clonal	0.62	1.92			polymorphism	4.46						
OVA_048_M1	METASTASIS	chr20	62699475	62699475	C	A	TCEA2	exonic	ynonymous	T00000440	174	185	359	0.51532	26	0	26	0	1	Clonal	0.56	3.94			polymorphism	13.55	p.A106D	Passenger	CANCER	benign	Neutral	Tolerated
OVA_048_M2	METASTASIS	chr20	62699475	62699475	C	A	TCEA2	exonic	ynonymous	T00000440	218	113	331	0.34139	26	0	26	0	1	Clonal	0.4	3.87			polymorphism	13.55	p.A106D	Passenger	CANCER	benign	Neutral	Tolerated
OVA_048_M3	METASTASIS	chr20	62699475	62699475	C	A	TCEA2	exonic	ynonymous	T00000440	182	160	342	0.467836	26	0	26	0	1	Clonal	0.59	3.39			polymorphism	13.55	p.A106D	Passenger	CANCER	benign	Neutral	Tolerated
OVA_003_M1	METASTASIS	chr12	62729107	62729107	T	G	USP15	UTR3	NA	NA	464	142	606	0.234323	18	0	18	0	0.83	Sub	0.57	1.84			polymorphism	4.682						
OVA_003_M2	METASTASIS	chr12	62729107	62729107	T	G	USP15	UTR3	NA	NA	513	121	634	0.190852	18	0	18	0	0.83	Sub	0.46	1.81			polymorphism	4.682						
OVA_003_M3	METASTASIS	chr12	62729107	62729107	T	G	USP15	UTR3	NA	NA	646	53	699	0.075823	18	0	18	0	0.8	Sub	0.34	2.67			polymorphism	4.682						
OVA_003_C1	PRIMARY	chr12	62729107	62729107	T	G	USP15	UTR3	NA	NA	379	280	659	0.424886	18	0	18	0	0.98	Clonal	0.86	1.81			polymorphism	4.682						
OVA_003_C2	PRIMARY	chr12	62729107	62729107	T	G	USP15	UTR3	NA	NA	363	256	619	0.41357	18	0	18	0	1	Clonal	0.7	3.61			polymorphism	4.682						
OVA_003_C3	PRIMARY	chr12	62729107	62729107	T	G	USP15	UTR3	NA	NA	395	274	669	0.409567	18	0	18	0	0.99	Clonal	0.82	1.81			polymorphism	4.682						
OVA_003_M1	METASTASIS	chr11	62748459	62748459	C	A	SLC22A6	exonic	nonymous S	L345L,ENSG	154	48	202	0.237624	58	0	58	0	0.84	Sub	0.57	1.84			disease_causing	13.07		Passenger			Neutral	Tolerated
OVA_003_M2	METASTASIS	chr11	62748459	62748459	C	A	SLC22A6	exonic	nonymous S	L345L,ENSG	212	47	259	0.181467	58	0	58	0	0.79	Sub	0.46	1.81			disease_causing	13.07		Passenger			Neutral	Tolerated
OVA_003_M3	METASTASIS	chr11	62748459	62748459	C	A	SLC22A6	exonic	nonymous S	L345L,ENSG	264	12	276	0.043478	58	0	58	0	0.46	Sub	0.34	2.67			disease_causing	13.07		Passenger			Neutral	Tolerated
OVA_003_C1	PRIMARY	chr11	62748459	62748459	C	A	SLC22A6	exonic	nonymous S	L345L,ENSG	140	88	228	0.385965	58	0	58	0	0.89	Clonal	0.86	1.81			disease_causing	13.07		Passenger			Neutral	Tolerated
OVA_003_C2	PRIMARY	chr11	62748459	62748459	C	A	SLC22A6	exonic	nonymous S	L345L,ENSG	205	102	307	0.332248	58	0	58	0	1	Clonal	0.7	3.61			disease_causing	13.07		Passenger			Neutral	Tolerated
OVA_003_C3	PRIMARY	chr11	62748459	62748459	C	A	SLC22A6	exonic	nonymous S	L345L,ENSG	213	119	332	0.358434	58	0	58	0	0.87	Clonal	0.82	1.81			disease_causing	13.07		Passenger			Neutral	Tolerated
OVA_003_C1	PRIMARY	chr3	62751570	62751570	C	T	CADPS	exonic	ynonymous	M177I,ENSG	373	247	620	0.398387	47	1	48	0.020833	0.92	Sub	0.86	1.81			disease_causing	21.7	p.M177I	Passenger	CANCER	benign	Neutral	Tolerated
OVA_003_C2	PRIMARY	chr3	62751570	62751570	C	T	CADPS	exonic	ynonymous	M177I,ENSG	478	147	625	0.2352	47	1	48	0.020833	1	Sub	0.7	3.61			disease_causing	21.7	p.M177I	Passenger	CANCER	benign	Neutral	Tolerated
OVA_003_C3	PRIMARY	chr3	62751570	62751570	C	T	CADPS	exonic	ynonymous	M177I,ENSG	428	242	670	0.361194	47	1	48	0.020833	0.88	Sub	0.82	1.81			disease_causing	21.7	p.M177I	Passenger	CANCER	benign	Neutral	Tolerated
OVA_047_C1	PRIMARY	chr20	62837140	62837140	C	A	MYT1	exonic	nonymous S	0000036014	512	134	646	0.20743	80	0	80	0	0.9	Clonal	0.6	2.91			polymorphism	4.795		Passenger			Neutral	Tolerated
OVA_047_C2	PRIMARY	chr20	62837140	62837140	C	A	MYT1	exonic	nonymous S	0000036014	455	152	607	0.250412	80	0	80	0	1	Clonal	0.64	2.76			polymorphism	4.795		Passenger			Neutral	Tolerated
OVA_047_C3	Contralateral ovary	chr20	62837140	62837140	C	A	MYT1	exonic	nonymous S	0000036014	485	125	610	0.204918	80	0	80	0	0.89	Clonal	0.6	2.94			polymorphism	4.795		Passenger			Neutral	Tolerated
OVA_048_C1	PRIMARY	chr20	62854660	62854660	C	A	MYT1	exonic	ynonymous	L826I,ENSG	92	36	128	0.28125	83	0	83	0	0.84	Clonal	0.67	1.82			disease_causing	32	p.L853I	Passenger	SENGER/OT	damaging	Neutral	Damaging
OVA_048_C3	PRIMARY	chr20	62854660	62854660	C	A	MYT1	exonic	ynonymous	L826I,ENSG	126	79	205	0.385366	83	0	83	0	1	Clonal	0.74	1.8			disease_causing	32	p.L853I	Passenger	SENGER/OT	damaging	Neutral	Damaging
OVA_013_M1	METASTASIS	chr11	62984921	62984921	A	C	SLC22A25	exonic	ynonymous	M232R,ENS	398	174	572	0.304196	45	1	46	0.021739	0.91	Clonal	0.67	3.06			polymorphism	18.05	p.M232R	Passenger	SENGER/OT	damaging	Deleterious	Damaging
OVA_013_C1	PRIMARY	chr11	62984921	62984921	A	C	SLC22A25	exonic	ynonymous	M232R,ENS	353	227	580	0.391379	45	1	46	0.021739	1	Clonal	0.72	3.1			polymorphism	18.05	p.M232R	Passenger	SENGER/OT	damaging	Deleterious	Damaging
OVA_013_C2	PRIMARY	chr11	62984921	62984921	A	C	SLC22A25	exonic	ynonymous	M232R,ENS	327	225	552	0.407609	45	1	46	0.021739	0.94	Clonal	0.87	3.05			polymorphism	18.05	p.M232R	Passenger	SENGER/OT	damaging	Deleterious	Damaging
OVA_013_C3	PRIMARY	chr11	62984921	62984921	A	C	SLC22A25	exonic	ynonymous	M232R,ENS	332	268	600	0.446667	45	1	46	0.021739	0.98	Clonal	0.91	3.01			polymorphism	18.05	p.M232R	Passenger	SENGER/OT	damaging	Deleterious	Damaging
OVA_047_C1	PRIMARY	chrX	63412811	63412811	G	C	AMER1	exonic	ynonymous	0000033025	482	138	620	0.222581	45	0	45	0	1	Sub	0.6	2.91	Yes	No	polymorphism	15.46	p.P119R	Passenger	SENGER/OT	benign	Neutral	Damaging
OVA_047_C2	PRIMARY	chrX	63412811	63412811	G	C	AMER1	exonic	ynonymous	0000033025	483	124	607	0.204283	45	0	45	0	0.84	Sub	0.64	2.76	Yes	No	polymorphism	15.46	p.P119R	Passenger	SENGER/OT	benign	Neutral	Damaging
OVA_013_M1	METASTASIS	chr10	63852005	63852005	C	A	ARID5B	exonic	ynonymous	585N,ENSG	171	205	376	0.545213	74	1	75	0.013333	1	Clonal	0.67	3.06			polymorphism	14.25	p.T928N	Passenger	SENGER/OT	benign	Neutral	Damaging
OVA_013_C1	PRIMARY	chr10	63852005	63852005	C	A	ARID5B	exonic	ynonymous	585N,ENSG	150	237	387	0.612403	74	1	75	0.013333	1	Clonal	0.72	3.1			polymorphism	14.25	p.T928N	Passenger	SENGER/OT	benign	Neutral	Damaging
OVA_013_C2	PRIMARY	chr10	63852005	63852005	C	A	ARID5B	exonic	ynonymous	585N,ENSG	168	229	397	0.576826	74	1	75	0.013333	1	Clonal	0.87	3.05			polymorphism	14.25	p.T928N	Passenger	SENGER/OT	benign	Neutral	Damaging
OVA_013_C3	PRIMARY	chr10	63852005	63852005	C	A	ARID5B	exonic	ynonymous	585N,ENSG	178	275	453	0.607064	74	1	75	0.013333	1	Clonal	0.91	3.01			polymorphism	14.25	p.T928N	Passenger	SENGER/OT	benign	Neutral	Damaging
OVA_013_M1	METASTASIS	chr10	64429957	64429957	G	A	ZNF365	intronic	NA	NA	419	168	587	0.286201	41	1	42	0.02381	1	Clonal	0.67	3.06			polymorphism	0.856						
OVA_013_C1	PRIMARY	chr10	64429957	64429957	G	A	ZNF365	intronic	NA	NA	433	158	591	0.267343	41	1	42	0.02381	1	Clonal	0.72	3.1			polymorphism	0.856						
OVA_013_C2	PRIMARY	chr10	64429957	64429957	G	A	ZNF365	intronic	NA	NA	429	160	589	0.271647	41	1	42	0.02381	0.9	Clonal	0.87	3.05			polymorphism	0.856						
OVA_013_C3	PRIMARY	chr10	64429957	64429957	G	A	ZNF365	intronic	NA	NA	441	180	621	0.289855	41	1	42	0.02381	0.92	Clonal	0.91	3.01			polymorphism	0.856						
OVA_365_M1	METASTASIS	chr14	64473851	64473851	C	A	SYNE2	exonic	ynonymous	000344113:	339	93	432	0.215278	34	0	34	0	0.51	Sub	0.85	1.87			polymorphism	0.08	p.H1496Q	Passenger	SENGER/OT	benign	Neutral	Tolerated
OVA_013_C1	PRIMARY	chr11	64514137	64514147	GCCTCATC(	-	PYGM	exonic	meshift dele	750fs,ENSG	245	67	312	0.214744	75	0	75		1	Clonal	0.72	3.1					p.P838Hfs	Passenger			NA	NA
OVA_013_C2	PRIMARY	chr11	64514137	64514147	GCCTCATC(	-	PYGM	exonic	meshift dele	750fs,ENSG	214	62	276	0.224638	75	0	75		0.97	Clonal	0.87	3.05					p.P838Hfs	Passenger			NA	NA
OVA_013_C3	PRIMARY	chr11	64514137	64514147	GCCTCATC(	-	PYGM	exonic	meshift dele	750fs,ENSG	243	75	318	0.235849	75	0	75		0.99	Clonal	0.91	3.01					p.P838Hfs	Passenger			NA	NA
OVA_365_M1	METASTASIS	chr17	64683238	64683238	-	A	PRKCA	exonic	meshift inse	180fs,ENSG	111	551	662	0.832326	19	0	19		1	Clonal	0.85	1.87			disease_causing		p.A180Dfs	Passenger				
OVA_365_M2	METASTASIS	chr17	64683238	64683238	-	A	PRKCA	exonic	meshift inse	180fs,ENSG	120	79	199	0.396985	19	0	19		1	Clonal	0.4	1.81			disease_causing		p.A180Dfs	Passenger				
OVA_365_M3	METASTASIS	chr17	64683238	64683238	-	A	PRKCA	exonic	meshift inse	180fs,ENSG	114	317	431	0.735499	19	0	19		1	Clonal	0.82	1.89			disease_causing		p.A180Dfs	Passenger				
OVA_048_C2	PRIMARY	chr11	64884285	64884285	G	C	L04003;ZNH	exonic	ynonymous	L281V,ENSG	37	18	55	0.327273	64	0	64	0	0.91	Clonal	0.72	1.85			disease_causing	23.8	p.L281V	Passenger	SENGER/OT	damaging	Neutral	Tolerated
OVA_003_M1	METASTASIS	chr5	64920769	64920769	G	A	TRAPPC13	UTR5	NA	NA	390	203	593	0.342327	19	0	19	0	0.87	Sub	0.57	1.84			polymorphism	8.456						
OVA_003_M2	METASTASIS	chr5	64920769	64920769	G	A	TRAPPC13	UTR5	NA	NA	488	147	635	0.231496	19	0	19	0	0.78	Sub	0.46	1.81			polymorphism	8.456						
OVA_003_M3	METASTASIS	chr5	64920769	64920769	G	A	TRAPPC13	UTR5	NA	NA	637	45	682	0.065982	19	0	19	0	0.5	Sub	0.34	2.67			polymorphism	8.456						
OVA_003_C1	PRIMARY	chr5	64920769	64920769	G	A	TRAPPC13	UTR5	NA	NA	127	465	592	0.785473	19	0	19	0	1	Clonal	0.86	1.81			polymorphism	8.456						
OVA_003_C2	PRIMARY	chr5	64920769	64920769	G	A	TRAPPC13	UTR5	NA	NA	167	439	606	0.724422	19	0	19	0	1	Clonal	0.7	3.61			polymorphism	8.456						
OVA_003_C3	PRIMARY	chr5	64920769	64920769	G	A	TRAPPC13	UTR5	NA	NA	186	413	599	0.689482	19	0	19	0	0.98	Clonal	0.82	1.81			polymorphism	8.456						
OVA_048_M1	METASTASIS	chr14	64954541	64954541	G	A	ZBTB25	exonic	nonymous S	T136T,ENSG	351	198	549	0.360656	39	0	39	0	1	Clonal	0.56	3.94			disease_causing	9.445		Passenger			Neutral	Tolerated
OVA_048_M2	METASTASIS	chr14	64954541	64954541	G	A	ZBTB25	exonic	nonymous S	T136T,ENSG	396	143	539	0.265306	39	0	39	0	1	Clonal	0.4	3.87			disease_causing	9.445		Passenger			Neutral	Tolerated
OVA_048_M3	METASTASIS	chr14	64954541	64954541	G	A	ZBTB25	exonic	nonymous S	T136T,ENSG	393	75	468	0.160256	39	0	39	0	1	Clonal	0.59	3.39			disease_causing	9.445		Passenger			Neutral	Tolerated
OVA_013_M1	METASTASIS	chr16	65025929	65025929	A	T	CDH11	intronic	NA	NA	354	156	510	0.305882	40	0	40	0	0.91	Clonal	0.67	3.06	Yes		polymorphism	4.656						
OVA_047_C1	PRIMARY	chr11	65266578	65266578	G	T	MALAT1	cRNA_exon	NA	NA	269	62	331	0.187311	36	0	36	0	0.81	Sub	0.6	2.91	Yes			11.24						
OVA_047_C2	PRIMARY	chr11	65266578	65266578	G	T	MALAT1	cRNA_exon	NA	NA	266	41	307	0.13355	36	0	36	0	0.69	Sub	0.64	2.76	Yes			11.24						
OVA_047_C3	Contralateral ovary	chr11	65266578	65266578	G	T	MALAT1	cRNA_exon	NA	NA	177	48	225	0.213333	36	0	36	0	0.93	Clonal	0.6	2.94	Yes			11.24						

Sample	Type	Chr	Start	End	Ref	Alt	Gene	unc.ensGen	icFunc.ens	hange.ens(	t_ref	t_alt	t_total	t_VAF	n_ref	n_alt	n_total	hormal_VA	ncer_cell_f	on-based C	purity	ploidy	Cancer Gene	Driver Gene	Mutation Taster	ADD(PHRE	otein_chan	CHASM	ATHMM_Pr	OLYPHEN-	REDICTION	ICTION (cutoff=0.05)
OVA_047_C1	PRIMARY	chr11	65266579	65266579	C	T	MALAT1	cRNA_exon	NA	NA	268	61	329	0.18541	36	0	36	0	0.8	Sub	0.6	2.91	Yes			14.11						
OVA_047_C2	PRIMARY	chr11	65266579	65266579	C	T	MALAT1	cRNA_exon	NA	NA	265	41	306	0.133987	36	0	36	0	0.69	Sub	0.64	2.76	Yes			14.11						
OVA_047_C3	Contralateral ovary	chr11	65266579	65266579	C	T	MALAT1	cRNA_exon	NA	NA	175	48	223	0.215247	36	0	36	0	0.94	Clonal	0.6	2.94	Yes			14.11						
OVA_003_M1	METASTASIS	chr6	65301532	65301532	A	T	EYS	exonic	ynonymou:	000370621	323	124	447	0.277405	43	0	43	0	0.98	Sub	0.57	1.84			polymorphism	18.63	p.L1410I	Passenger	SENGER/OT	damaging	Neutral	Tolerated
OVA_003_M2	METASTASIS	chr6	65301532	65301532	A	T	EYS	exonic	ynonymou:	000370621	436	95	531	0.178908	43	0	43	0	0.78	Sub	0.46	1.81			polymorphism	18.63	p.L1410I	Passenger	SENGER/OT	damaging	Neutral	Tolerated
OVA_003_M3	METASTASIS	chr6	65301532	65301532	A	T	EYS	exonic	ynonymou:	000370621	509	29	538	0.053903	43	0	43	0	0.57	Sub	0.34	2.67			polymorphism	18.63	p.L1410I	Passenger	SENGER/OT	damaging	Neutral	Tolerated
OVA_003_C1	PRIMARY	chr6	65301532	65301532	A	T	EYS	exonic	ynonymou:	000370621	236	212	448	0.473214	43	0	43	0	1	Clonal	0.86	1.81			polymorphism	18.63	p.L1410I	Passenger	SENGER/OT	damaging	Neutral	Tolerated
OVA_003_C2	PRIMARY	chr6	65301532	65301532	A	T	EYS	exonic	ynonymou:	000370621	312	193	505	0.382178	43	0	43	0	1	Clonal	0.7	3.61			polymorphism	18.63	p.L1410I	Passenger	SENGER/OT	damaging	Neutral	Tolerated
OVA_003_C3	PRIMARY	chr6	65301532	65301532	A	T	EYS	exonic	ynonymou:	000370621	346	210	556	0.377698	43	0	43	0	0.92	Clonal	0.82	1.81			polymorphism	18.63	p.L1410I	Passenger	SENGER/OT	damaging	Neutral	Tolerated
OVA_047_M1	METASTASIS	chr6	65303164	65303164	C	A	EYS	exonic	ynonymou:	000370621	486	170	656	0.259146	57	0	57	0	1	Sub	0.47	2.01			polymorphism	7.698	p.R1241S	Passenger	SENGER/OT	benign	Neutral	Tolerated
OVA_047_M2	METASTASIS	chr6	65303164	65303164	C	A	EYS	exonic	ynonymou:	000370621	484	157	641	0.24493	57	0	57	0	1	Sub	0.44	2.01			polymorphism	7.698	p.R1241S	Passenger	SENGER/OT	benign	Neutral	Tolerated
OVA_047_M3	METASTASIS	chr6	65303164	65303164	C	A	EYS	exonic	ynonymou:	000370621	357	62	419	0.147971	57	0	57	0	0.64	Sub	0.6	3.02			polymorphism	7.698	p.R1241S	Passenger	SENGER/OT	benign	Neutral	Tolerated
OVA_047_M1	METASTASIS	chr3	65364932	65364932	T	A	AK124433	intronic	NA	NA	474	146	620	0.235484	55	0	55	0	1	Clonal	0.47	2.01			disease_causing	18.62						
OVA_047_M2	METASTASIS	chr3	65364932	65364932	T	A	AK124433	intronic	NA	NA	492	144	636	0.226415	55	0	55	0	1	Clonal	0.44	2.01			disease_causing	18.62						
OVA_047_M3	METASTASIS	chr3	65364932	65364932	T	A	AK124433	intronic	NA	NA	213	53	266	0.199248	55	0	55	0	1	Clonal	0.6	3.02			disease_causing	18.62						
OVA_047_M1	METASTASIS	chr11	65423166	65423166	G	-	RELA	exonic	neshift dele	342fs,ENSG	897	227	1124	0.201957	89	0	89		0.85	Sub	0.47	2.01					p.K343Sfs	Passenger			NA	NA
OVA_047_M2	METASTASIS	chr11	65423166	65423166	G	-	RELA	exonic	neshift dele	342fs,ENSG	547	137	684	0.200292	89	0	89		0.9	Sub	0.44	2.01					p.K343Sfs	Passenger			NA	NA
OVA_047_M3	METASTASIS	chr11	65423166	65423166	G	-	RELA	exonic	neshift dele	342fs,ENSG	204	56	260	0.215385	89	0	89		0.93	Sub	0.6	3.02					p.K343Sfs	Passenger			NA	NA
OVA_048_M1	METASTASIS	chr17	65733398	65733398	G	C	NOL11	intronic	NA	NA	136	605	741	0.816464	42	0	42	0	1	Clonal	0.56	3.94			polymorphism	3.307						
OVA_048_M2	METASTASIS	chr17	65733398	65733398	G	C	NOL11	intronic	NA	NA	264	471	735	0.640816	42	0	42	0	1	Clonal	0.4	3.87			polymorphism	3.307						
OVA_048_M3	METASTASIS	chr17	65733398	65733398	G	C	NOL11	intronic	NA	NA	176	536	712	0.752809	42	0	42	0	1	Clonal	0.59	3.39			polymorphism	3.307						
OVA_048_C1	PRIMARY	chr17	65733398	65733398	G	C	NOL11	intronic	NA	NA	181	307	488	0.629098	42	0	42	0	1	Clonal	0.67	1.82			polymorphism	3.307						
OVA_048_C2	PRIMARY	chr17	65733398	65733398	G	C	NOL11	intronic	NA	NA	218	401	619	0.647819	42	0	42	0	1	Clonal	0.72	1.85			polymorphism	3.307						
OVA_048_C3	PRIMARY	chr17	65733398	65733398	G	C	NOL11	intronic	NA	NA	200	331	531	0.623352	42	0	42	0	1	Clonal	0.74	1.8			polymorphism	3.307						
OVA_003_M1	METASTASIS	chr1	65830404	65830404	C	A	DNAJC6	exonic	ynonymou:	p.L37I,ENS(	174	126	300	0.42	41	0	41	0	1	Sub	0.57	1.84			disease_causing	12.59	p.L94I	Passenger	CANCER	benign	Neutral	Tolerated
OVA_003_M2	METASTASIS	chr1	65830404	65830404	C	A	DNAJC6	exonic	ynonymou:	p.L37I,ENS(	270	123	393	0.312977	41	0	41	0	1	Sub	0.46	1.81			disease_causing	12.59	p.L94I	Passenger	CANCER	benign	Neutral	Tolerated
OVA_003_M3	METASTASIS	chr1	65830404	65830404	C	A	DNAJC6	exonic	ynonymou:	p.L37I,ENS(	531	36	567	0.063492	41	0	41	0	0.48	Sub	0.34	2.67			disease_causing	12.59	p.L94I	Passenger	CANCER	benign	Neutral	Tolerated
OVA_003_C1	PRIMARY	chr1	65830404	65830404	C	A	DNAJC6	exonic	ynonymou:	p.L37I,ENS(	89	309	398	0.776382	41	0	41	0	1	Clonal	0.86	1.81			disease_causing	12.59	p.L94I	Passenger	CANCER	benign	Neutral	Tolerated
OVA_003_C2	PRIMARY	chr1	65830404	65830404	C	A	DNAJC6	exonic	ynonymou:	p.L37I,ENS(	109	431	540	0.798148	41	0	41	0	1	Clonal	0.7	3.61			disease_causing	12.59	p.L94I	Passenger	CANCER	benign	Neutral	Tolerated
OVA_003_C3	PRIMARY	chr1	65830404	65830404	C	A	DNAJC6	exonic	ynonymou:	p.L37I,ENS(	117	409	526	0.777567	41	0	41	0	1	Clonal	0.82	1.81			disease_causing	12.59	p.L94I	Passenger	CANCER	benign	Neutral	Tolerated
OVA_048_C1	PRIMARY	chr15	66208662	66208662	C	T	MEGF11	intronic	NA	NA	117	68	185	0.367568	46	0	46	0	1	Clonal	0.67	1.82			polymorphism	0.359						
OVA_048_C3	PRIMARY	chr15	66208662	66208662	C	T	MEGF11	intronic	NA	NA	148	95	243	0.390947	46	0	46	0	1	Clonal	0.74	1.8			polymorphism	0.359						
OVA_013_M1	METASTASIS	chr11	66260244	66260244	G	C	DPP3	exonic	ynonymou:	349T,ENSG(	198	51	249	0.204819	15	0	15	0	1	Clonal	0.67	3.06			polymorphism	15.49	p.S369T	Passenger	SENGER/OT	benign	Neutral	Tolerated
OVA_003_M1	METASTASIS	chr17	66303587	66303587	G	T	ARSG	UTR5	NA	NA	172	39	211	0.184834	29	0	29	0	0.47	Sub	0.57	1.84			polymorphism	5.688						
OVA_003_M2	METASTASIS	chr17	66303587	66303587	G	T	ARSG	UTR5	NA	NA	318	21	339	0.061947	29	0	29	0	0.21	Sub	0.46	1.81			polymorphism	5.688						
OVA_003_M3	METASTASIS	chr17	66303587	66303587	G	T	ARSG	UTR5	NA	NA	369	6	375	0.016	29	0	29	0	0.12	Sub	0.34	2.67			polymorphism	5.688						
OVA_003_C1	PRIMARY	chr17	66303587	66303587	G	T	ARSG	UTR5	NA	NA	41	171	212	0.806604	29	0	29	0	1	Clonal	0.86	1.81			polymorphism	5.688						
OVA_003_C2	PRIMARY	chr17	66303587	66303587	G	T	ARSG	UTR5	NA	NA	173	92	265	0.34717	29	0	29	0	0.99	Clonal	0.7	3.61			polymorphism	5.688						
OVA_003_C3	PRIMARY	chr17	66303587	66303587	G	T	ARSG	UTR5	NA	NA	81	167	248	0.673387	29	0	29	0	0.96	Clonal	0.82	1.81			polymorphism	5.688						
OVA_378_M1	METASTASIS	chr11	66392015	66392015	G	A	RBM14	exonic	ynonymou:	000031013	93	70	163	0.429448	121	0	121	0	1	Clonal	0.72	2.57			disease_causing	27.3	p.R223Q	Passenger	SENGER/OT	damaging	Neutral	Damaging
OVA_378_M2	METASTASIS	chr11	66392015	66392015	G	A	RBM14	exonic	ynonymou:	000031013	78	68	146	0.465753	121	0	121	0	1	Clonal	0.85	2.57			disease_causing	27.3	p.R223Q	Passenger	SENGER/OT	damaging	Neutral	Damaging
OVA_378_M3	METASTASIS	chr11	66392015	66392015	G	A	RBM14	exonic	ynonymou:	000031013	133	83	216	0.384259	121	0	121	0	1	Clonal	0.67	2.57			disease_causing	27.3	p.R223Q	Passenger	SENGER/OT	damaging	Neutral	Damaging
OVA_378_C1	PRIMARY	chr11	66392015	66392015	G	A	RBM14	exonic	ynonymou:	000031013	139	68	207	0.328502	121	0	121	0	1	Clonal	0.59	2.6			disease_causing	27.3	p.R223Q	Passenger	SENGER/OT	damaging	Neutral	Damaging
OVA_378_C2	PRIMARY	chr11	66392015	66392015	G	A	RBM14	exonic	ynonymou:	000031013	126	49	175	0.28	121	0	121	0	0.99	Clonal	0.56	2.59			disease_causing	27.3	p.R223Q	Passenger	SENGER/OT	damaging	Neutral	Damaging
OVA_378_C3	PRIMARY	chr11	66392015	66392015	G	A	RBM14	exonic	ynonymou:	000031013	143	54	197	0.274112	121	0	121	0	1	Clonal	0.51	2.6			disease_causing	27.3	p.R223Q	Passenger	SENGER/OT	damaging	Neutral	Damaging
OVA_378_M1	METASTASIS	chr5	66449292	66449292	G	A	MAST4	exonic	ynonymou:	00405643:	380	229	609	0.376026	64	1	65	0.015385	1	Clonal	0.72	2.57	Yes	No	disease_causing	32	p.E1178K	Driver	SENGER/OT	damaging	Deleterious	Damaging
OVA_378_M2	METASTASIS	chr5	66449292	66449292	G	A	MAST4	exonic	ynonymou:	00405643:	340	244	584	0.417808	64	1	65	0.015385	0.98	Clonal	0.85	2.57	Yes	No	disease_causing	32	p.E1178K	Driver	SENGER/OT	damaging	Deleterious	Damaging
OVA_378_M3	METASTASIS	chr5	66449292	66449292	G	A	MAST4	exonic	ynonymou:	00405643:	423	225	648	0.347222	64	1	65	0.015385	1	Clonal	0.67	2.57	Yes	No	disease_causing	32	p.E1178K	Driver	SENGER/OT	damaging	Deleterious	Damaging
OVA_378_C1	PRIMARY	chr5	66449292	66449292	G	A	MAST4	exonic	ynonymou:	00405643:	452	183	635	0.288189	64	1	65	0.015385	0.98	Clonal	0.59	2.6	Yes	No	disease_causing	32	p.E1178K	Driver	SENGER/OT	damaging	Deleterious	Damaging
OVA_378_C2	PRIMARY	chr5	66449292	66449292	G	A	MAST4	exonic	ynonymou:	00405643:	460	159	619	0.256866	64	1	65	0.015385	0.91	Clonal	0.56	2.59	Yes	No	disease_causing	32	p.E1178K	Driver	SENGER/OT	damaging	Deleterious	Damaging
OVA_378_C3	PRIMARY	chr5	66449292	66449292	G	A	MAST4	exonic	ynonymou:	00405643:	468	145	613	0.236542	64	1	65	0.015385	0.93	Clonal	0.51	2.6	Yes	No	disease_causing	32	p.E1178K	Driver	SENGER/OT	damaging	Deleterious	Damaging
OVA_048_M1	METASTASIS	chr3	66455764	66455764	C	T	LRIG1	intronic	NA	NA	155	62	217	0.285714	35	0	35	0	1	Sub	0.56	3.94			polymorphism	5.359						
OVA_048_M2	METASTASIS	chr3	66455764	66455764	C	T	LRIG1	intronic	NA	NA	236	26	262	0.099237	35	0	35	0	0.69	Sub	0.4	3.87			polymorphism	5.359						
OVA_048_C1	PRIMARY	chr11	66472920	66472920	C	T	SPTBN2	exonic	nonymous S	000309996	50	34	84	0.404762	27	0	27	0	1	Clonal	0.67	1.82			disease_causing	0.935		Passenger			Neutral	Tolerated
OVA_048_C2	PRIMARY	chr11	66472920	66472920	C	T	SPTBN2	exonic	nonymous S	000309996	132	101	233	0.433476	27	0	27	0	1	Clonal	0.72	1.85			disease_causing	0.935		Passenger			Neutral	Tolerated
OVA_048_C3	PRIMARY	chr11	66472920	66472920	C	T	SPTBN2	exonic	nonymous S	000309996	48	43	91	0.472527	27	0	27	0	1	Clonal	0.74	1.8			disease_causing	0.935		Passenger			Neutral	Tolerated
OVA_003_M1	METASTASIS	chr16	66613542	66613542	C	T	CMTM2	exonic	ynonymou:	T00000379	101	60	161	0.372671	65	0	65	0	0.95	Sub	0.57	1.84			polymorphism	6.337	p.P11L	Passenger	SENGER/OT	benign	Neutral	Damaging
OVA_003_M2	METASTASIS	chr16	66613542	66613542	C	T	CMTM2	exonic	ynonymou:	T00000379	134	74	208	0.355769	65	0	65	0	1	Sub	0.46	1.81			polymorphism	6.337	p.P11L	Passenger	SENGER/OT	benign	Neutral	Damaging
OVA_003_M3	METASTASIS	chr16	66613542	66613542	C	T	CMTM2	exonic	ynonymou:	T00000379	223	12	235	0.051064	65	0	65	0	0.39	Sub	0.34	2.67			polymorphism	6.337	p.P11L	Passenger	SENGER/OT	benign	Neutral	Damaging
OVA_003_C1	PRIMARY	chr16	66613542	66613542	C	T	CMTM2	exonic	ynonymou:	T00000379	38	137	175	0.782857	65	0	65	0	1	Clonal	0.86	1.81			polymorphism	6.337	p.P11L	Passenger	SENGER/OT	benign	Neutral	Damaging
OVA_003_C2	PRIMARY	chr16	66613542	66613542	C	T	CMTM2	exonic	ynonymou:	T00000379	44	134	178	0.752809	65	0	65	0	1	Clonal	0.7	3.61			polymorphism	6.337	p.P11L	Passenger	SENGER/OT	benign	Neutral	Damaging
OVA_003_C3	PRIMARY	chr16	66613542	66613542	C	T	CMTM2	exonic	ynonymou:	T00000379	35	158	193	0.818653	65	0	65	0	1	Clonal	0.82	1.81			polymorphism	6.337	p.P11L	Passenger	SENGER/OT	benign	Neutral	Damaging
OVA_378_M1	METASTASIS	chr16	66969272	66969272	G	T	CES2	UTR5	NA	NA	96	70	166	0.421687	58	0	58	0	1	Clonal	0.72	2.57			polymorphism	1.155						
OVA_378_M2	METASTASIS	chr16	66969272	66969272	G	T	CES2	UTR5	NA	NA	80	70	150	0.466667	58	0	58	0	1	Clonal	0.85	2.57			polymorphism	1.155						
OVA_378_M3	METASTASIS	chr16	66969272	66969272	G	T	CES2	UTR5	NA	NA	180	85	265	0.320755	58	0	58	0	0.96	Clonal	0.67	2.57			polymorphism	1.155						
OVA_378_C1	PRIMARY	chr16	66969272	66969272	G	T	CES2	UTR5	NA	NA	162	71	233	0.304721	58	0	58	0	1	Clonal	0.59	2.6			polymorphism	1.155						
OVA_378_C2	PRIMARY	chr16	66969272	66969272	G	T	CES2	UTR5	NA	NA	130	50	180	0.277778	58	0	58	0	0.99	Clonal	0.56	2.59			polymorphism	1.155						
OVA_378_C3	PRIMARY	chr16	66969272	66969272	G	T	CES2	UTR5	NA	NA	116	44	160	0.275	58	0	58	0	1	Clonal	0.51	2.6			polymorphism	1.155						
OVA_047_C2	PRIMARY	chr17	67028248	67028248	C	A	ABCA9	splicing	NA	NA	581	97	678	0.143068	28	0	28	0	0.59	Sub	0.64	2.76			disease_causing	32		Passenger				
OVA_048_M1	METASTASIS	chr16	67197105	67197105	G	C	FBXL8	exonic	nonymous S	L169L,ENSG	137	80	217	0.368664	125	0	125	0	1	Clonal	0.56	3.94			disease_causing	16.98		Passenger			Neutral	Tolerated
OVA_048_M2	METASTASIS	chr16	67197105	67197105	G	C	FBXL8	exonic	nonymous S	L169L,ENSG	173	79	252	0.313492	125	0	125	0	1	Clonal	0.4	3.87			disease_causing	16.98		Passenger			Neutral	Tolerated
OVA_048_M3	METASTASIS	chr16	67197105	67197105	G	C	FBXL8	exonic	nonymous S	L169L,ENSG	106	74	180	0.411111	125	0	125	0	1	Clonal	0.59	3.39			disease_causing	16.98		Passenger			Neutral	Tolerated
OVA_047_M1	METASTASIS	chr16	67235954	67235954	C	T	ELMO3	exonic	nonymous S	000047789	77	85	162	0.524691	82	0	82	0	NA	Sub	0.47	2.01			disease_causing	17.92		Passenger			Neutral	Tolerated
OVA_047_M2	METASTASIS	chr16	67235954	67235954	C	T	ELMO3	exonic	nonymous S	000047789	119	91	210	0.433333	82	0	82	0	NA	Sub	0.44	2.01			disease_causing	17.92		Passenger			Neutral	Tolerated
OVA_047_M3	METASTASIS	chr16	67235954	67235954	C	T	ELMO3	exonic	nonymous S	000047789	36	36	72	0.5	82	0	82	0	1	Sub	0.6	3.02			disease_causing	17.92		Passenger			Neutral	Tolerated
OVA_365_M1	METASTASIS	chr11	67374399	67374399	G	T	NDUFV1	UTR5	NA	NA	95	89	184	0.483696	128	0	128	0	1	Clonal	0.85	1.87			polymorphism	11.75						
OVA_365_M2	METASTASIS	chr11	67374399	67374399	G	T	NDUFV1	UTR5	NA	NA	94	35	129	0.271318	128	0	128	0	1	Clonal	0.4	1.81			polymorphism	11.75						
OVA_365_M3	METASTASIS	chr11	67374399	67374399	G	T	NDUFV1	UTR5	NA	NA	95	57	152	0.375	128	0	128	0	0.92	Clonal	0.82	1.89			polymorphism	11.75						
OVA_365_C1	Contralateral ovary	chr11	67374399	67374399	G	T	NDUFV1	UTR5	NA	NA	125	42	167	0.251497	128	0	128	0	1	Clonal	0.42	1.83			polymorphism	11.75						
OVA_365_C2	PRIMARY	chr11	67374399	67374399	G	T	NDUFV1	UTR5	NA	NA	132	129	261	0.494253	128	0	128	0	1	Clonal	0.62	1.92			polymorphism	11.75						
OVA_013_C1	PRIMARY	chr16	67440159	67440159	C	T	ZDHHC1	exonic	ynonymou:	000003485	214	81	295	0.274576	156	0	156	0	0.76	Sub	0.72	3.1			disease_causing	24.3	p.G66S	Passenger	CANCER	damaging	Neutral	Tolerated
OVA_378_M1	METASTASIS	chr18	67563165	67563165	T	A	CD226	exonic	stopgain	000028020	471	199	670	0.297015	70	0	70	0	1	Clonal	0.72	2.57			disease_causing_automat	38	p.K167X	Passenger			NA	NA
OVA_378_M2	METASTASIS	chr18	67563165	67563165	T	A	CD226	exonic	stopgain	000028020	423	224	647	0.346213	70	0	70	0	1	Clonal	0.85	2.57			disease_causing_automat	38	p.K167X	Passenger			NA	NA
OVA_378_M3	METASTASIS	chr18	67563165	67563165	T	A	CD226	exonic	stopgain	000028020	524	151	675	0.223704	70	0	70	0	0.89	Clonal	0.67	2.57			disease_causing_automat	38	p.K167X	Passenger			NA	NA
OVA_378_C1	PRIMARY	chr18	67563165	67563165	T	A	CD226	exonic	stopgain	000028020	2852	42	2898	0.014493	70	0	70	0	0.06	Sub	0.59	2.6			disease_causing_automat	38	p.K167X	Passenger			NA	NA
OVA_003_M1	METASTASIS	chr8	67578648	67578648	C	G	VCPIP1	exonic	ynonymou:	000031042	375	179	554	0.323105	211	0	211	0	1	Sub	0.57	1.84			disease_causing	23.3	p.K182N	Passenger	SENGER/OT	damaging	Neutral	Damaging
OVA_003_M2	METASTASIS	chr8	67578648	67578648	C	G	VCPIP1	exonic	ynonymou:	000031042	455	120	575	0.208696	211	0	211	0	0.91	Sub	0.46	1.81			disease_causing	23.3	p.K182N	Passenger	SENGER/OT	damaging	Neutral	Damaging
OVA_003_M3	METASTASIS	chr8	67578648	67578648	C	G	VCPIP1	exonic	ynonymou:	000031042	585	40	625	0.064	211	0	211	0	0.67	Sub	0.34	2.67			disease_causing	23.3	p.K182N	Passenger	SENGER/OT	damaging	Neutral	Damaging
OVA_003_C1	PRIMARY	chr8	67578648	67578648	C	G	VCPIP1	exonic	ynonymou:	000031042	316	276	592	0.466216	211	0	211	0	1	Clonal	0.86	1.81			disease_causing	23.3	p.K182N	Passenger	SENGER/OT	damaging	Neutral	Damaging
OVA_003_C2	PRIMARY	chr8	67578648	67578648	C	G	VCPIP1	exonic	ynonymou:	000031042	345	253	598	0.423077	211	0	211	0	1	Clonal	0.7	3.61			disease_causing	23.3	p.K182N	Passenger	SENGER/OT	damaging	Neutral	Damaging
OVA_003_C3	PRIMARY	chr8	67578648	67578648	C	G	VCPIP1	exonic	ynonymou:	000031042	375	256	631	0.405705	211	0	211	0	0.98	Clonal	0.82	1.81			disease_causing	23.3	p.K182N	Passenger	SENGER/OT	damaging	Neutral	Damaging
OVA_048_M1	METASTASIS	chr16	67688253	67688253	C	T	RLTPR	exonic	nonymous S	.080D,ENSG	70	7	77	0.090909	36	0	36	0	0.5	Sub	0.56	3.94			polymorphism	15.12		Passenger			Neutral	Tolerated
OVA_047_C1	PRIMARY	chr16	67691433	67691433	C	A	ACD;RLTPR	UTR3	NA	NA	105	15	120	0.125	25	0	25	0	0.29	Sub	0.6	2.91			disease_causing	20.6						
OVA_048_M1	METASTASIS	chr8	67705893	67705893	T	-	f44-SGK3;S	UTR5	NA	NA	576	197	773	0.254851	25	0	25		1	Clonal	0.56	3.94										
OVA_048_M2	METASTASIS	chr8	67705893	67705893	T	-	f44-SGK3;S	UTR5	NA	NA	1190	285	1475	0.19322	25	0	25		1	Clonal	0.4	3.87										
OVA_048_M3	METASTASIS	chr8	67705893	67705893	T	-	f44-SGK3;S	UTR5	NA	NA	1104	189	1293	0.146172	25	0	25		1	Clonal	0.59	3.39										
OVA_048_C3	PRIMARY	chr8	67705893	67705893	T	-	f44-SGK3;S	UTR5	NA	NA	471	150	621	0.241546	25	0	25		0.89	Clonal	0.74	1.8										
OVA_048_C1	PRIMARY	chr8	67705893	67705893	T	-	f44-SGK3;S	UTR5	NA	NA	534	188	722	0.260388	25	0	25		1	Clonal	0.67	1.82										
OVA_048_C2	PRIMARY	chr8	67705893	67705893	T	-	f44-SGK3;S	UTR5	NA	NA	582	210	792	0.265152	25	0	25		1	Clonal	0.72	1.85										
OVA_047_M1	METASTASIS	chr17	68172500	68172523	TGTTGGTC	-	KCNJ2	UTR3	NA	NA	829	298	1127	0.264419	67	0	67		1	Clonal	0.47	2.01										

Sample	Type	Chr	Start	End	Ref	Alt	Gene	unc.ensGen	icFunc.ens	hange.ensG	t_ref	t_alt	t_total	t_VAF	n_ref	n_alt	n_total	ormal_VA	ncer_cell_f	on-based C	purity	ploidy	Cancer Gene	Driver Gene	Mutation Taster	ADD(PHRE	otein_chan	CHASM	ATHMM_Pr	OLYPHEN-	REDICTION	ICTION (cutoff=0.05)
OVA_047_M2	METASTASIS	chr17	68172500	68172523	TGTTGGTC	-	KCNJ2	UTR3	NA	NA	927	279	1206	0.231343	67	0	67		1	Clonal	0.44	2.01										
OVA_047_M3	METASTASIS	chr17	68172500	68172523	TGTTGGTC	-	KCNJ2	UTR3	NA	NA	341	109	450	0.242222	67	0	67		1	Clonal	0.6	3.02										
OVA_047_C1	PRIMARY	chrX	68382969	68382969	G	A	PJA1	exonic	ynonymous	T38M,ENSG	527	152	679	0.223859	78	0	78	0	1	Sub	0.6	2.91			polymorphism	12.02	p.T38M	Passenger	SENGER/OT	damaging	Neutral	Tolerated
OVA_047_C2	PRIMARY	chrX	68382969	68382969	G	A	PJA1	exonic	ynonymous	T38M,ENSG	539	144	683	0.210835	78	0	78	0	0.87	Sub	0.64	2.76			polymorphism	12.02	p.T38M	Passenger	SENGER/OT	damaging	Neutral	Tolerated
OVA_047_C3	Contralateral ovary	chrX	68382969	68382969	G	A	PJA1	exonic	ynonymous	T38M,ENSG	546	136	682	0.199413	78	0	78	0	1	Clonal	0.6	2.94			polymorphism	12.02	p.T38M	Passenger	SENGER/OT	damaging	Neutral	Tolerated
OVA_047_M1	METASTASIS	chr3	68802128	68802128	C	T	FAM19A4	exonic	ynonymous	V58M,ENSG	486	133	619	0.214863	44	0	44	0	1	Clonal	0.47	2.01			polymorphism	24.5	p.V58M	Passenger	no weights	damaging	Neutral	Tolerated
OVA_047_M2	METASTASIS	chr3	68802128	68802128	C	T	FAM19A4	exonic	ynonymous	V58M,ENSG	515	150	665	0.225564	44	0	44	0	1	Clonal	0.44	2.01			polymorphism	24.5	p.V58M	Passenger	no weights	damaging	Neutral	Tolerated
OVA_047_M3	METASTASIS	chr3	68802128	68802128	C	T	FAM19A4	exonic	ynonymous	V58M,ENSG	297	90	387	0.232558	44	0	44	0	1	Clonal	0.6	3.02			polymorphism	24.5	p.V58M	Passenger	no weights	damaging	Neutral	Tolerated
OVA_365_M1	METASTASIS	chr11	68825028	68825028	C	T	TPCN2	intronic	NA	NA	116	94	210	0.447619	43	0	43	0	1	Sub	0.85	1.87			polymorphism	1.423						
OVA_365_M3	METASTASIS	chr11	68825028	68825028	C	T	TPCN2	intronic	NA	NA	103	39	142	0.274648	43	0	43	0	0.67	Sub	0.82	1.89			polymorphism	1.423						
OVA_365_M1	METASTASIS	chr1	68903879	68903879	A	G	RPE65	exonic	nonymous S	0000262340	72	278	350	0.794286	32	0	32	0	1	Clonal	0.85	1.87			disease_causing	8.827		Passenger			Neutral	Tolerated
OVA_365_M2	METASTASIS	chr1	68903879	68903879	A	G	RPE65	exonic	nonymous S	0000262340	180	60	240	0.25	32	0	32	0	0.99	Clonal	0.4	1.81			disease_causing	8.827		Passenger			Neutral	Tolerated
OVA_365_M3	METASTASIS	chr1	68903879	68903879	A	G	RPE65	exonic	nonymous S	0000262340	68	232	300	0.773333	32	0	32	0	1	Clonal	0.82	1.89			disease_causing	8.827		Passenger			Neutral	Tolerated
OVA_365_C1	Contralateral ovary	chr1	68903879	68903879	A	G	RPE65	exonic	nonymous S	0000262340	249	70	319	0.219436	32	0	32	0	1	Clonal	0.42	1.83			disease_causing	8.827		Passenger			Neutral	Tolerated
OVA_365_C2	PRIMARY	chr1	68903879	68903879	A	G	RPE65	exonic	nonymous S	0000262340	298	149	447	0.333333	32	0	32	0	1	Clonal	0.62	1.92			disease_causing	8.827		Passenger			Neutral	Tolerated
OVA_365_M1	METASTASIS	chr4	68939712	68939712	G	A	TMPRSS11	exonic	stopgain	0000035629	120	103	223	0.461883	24	0	24	0	1	Clonal	0.85	1.87			disease_causing_automat	36	p.R100X	Passenger			NA	NA
OVA_365_M2	METASTASIS	chr4	68939712	68939712	G	A	TMPRSS11	exonic	stopgain	0000035629	68	11	79	0.139241	24	0	24	0	0.69	Clonal	0.4	1.81			disease_causing_automat	36	p.R100X	Passenger			NA	NA
OVA_365_M3	METASTASIS	chr4	68939712	68939712	G	A	TMPRSS11	exonic	stopgain	0000035629	58	54	112	0.482143	24	0	24	0	1	Clonal	0.82	1.89			disease_causing_automat	36	p.R100X	Passenger			NA	NA
OVA_365_C1	Contralateral ovary	chr4	68939712	68939712	G	A	TMPRSS11	exonic	stopgain	0000035629	60	19	79	0.240506	24	0	24	0	1	Clonal	0.42	1.83			disease_causing_automat	36	p.R100X	Passenger			NA	NA
OVA_365_C2	PRIMARY	chr4	68939712	68939712	G	A	TMPRSS11	exonic	stopgain	0000035629	101	37	138	0.268116	24	0	24	0	0.87	Clonal	0.62	1.92			disease_causing_automat	36	p.R100X	Passenger			NA	NA
OVA_013_M1	METASTASIS	chr12	69107568	69107568	C	A	NUP107	exonic	ynonymous	000022917	310	308	618	0.498382	60	0	60	0	1	Clonal	0.67	3.06			disease_causing	23.6	p.R317S	Passenger	no weights	damaging	Deleterious	Damaging
OVA_013_C1	PRIMARY	chr12	69107568	69107568	C	A	NUP107	exonic	ynonymous	000022917	267	318	585	0.54359	60	0	60	0	1	Clonal	0.72	3.1			disease_causing	23.6	p.R317S	Passenger	no weights	damaging	Deleterious	Damaging
OVA_013_C2	PRIMARY	chr12	69107568	69107568	C	A	NUP107	exonic	ynonymous	000022917	238	325	563	0.577265	60	0	60	0	1	Clonal	0.87	3.05			disease_causing	23.6	p.R317S	Passenger	no weights	damaging	Deleterious	Damaging
OVA_013_C3	PRIMARY	chr12	69107568	69107568	C	A	NUP107	exonic	ynonymous	000022917	235	382	617	0.619125	60	0	60	0	1	Clonal	0.91	3.01			disease_causing	23.6	p.R317S	Passenger	no weights	damaging	Deleterious	Damaging
OVA_047_C1	PRIMARY	chr16	69171746	69171746	C	G	CIRH1A	exonic	ynonymous	0000056309	208	108	316	0.341772	44	0	44	0	0.8	Sub	0.6	2.91			polymorphism	15.07	p.P132A	Passenger	SENGER/OT	benign	Neutral	Tolerated
OVA_047_C2	PRIMARY	chr16	69171746	69171746	C	G	CIRH1A	exonic	ynonymous	0000056309	187	155	342	0.453216	44	0	44	0	0.97	Sub	0.64	2.76			polymorphism	15.07	p.P132A	Passenger	SENGER/OT	benign	Neutral	Tolerated
OVA_047_C3	Contralateral ovary	chr16	69171746	69171746	C	G	CIRH1A	exonic	ynonymous	0000056309	197	132	329	0.401216	44	0	44	0	0.94	Clonal	0.6	2.94			polymorphism	15.07	p.P132A	Passenger	SENGER/OT	benign	Neutral	Tolerated
OVA_013_M1	METASTASIS	chrX	69457085	69457085	C	T	AWAT1	exonic	nonymous S	0000037452	280	84	364	0.230769	26	0	26	0	0.92	Clonal	0.67	3.06			disease_causing	9.774		Passenger			Neutral	Tolerated
OVA_013_C1	PRIMARY	chrX	69457085	69457085	C	T	AWAT1	exonic	nonymous S	0000037452	384	152	536	0.283582	26	0	26	0	1	Clonal	0.72	3.1			disease_causing	9.774		Passenger			Neutral	Tolerated
OVA_013_C2	PRIMARY	chrX	69457085	69457085	C	T	AWAT1	exonic	nonymous S	0000037452	347	134	481	0.278586	26	0	26	0	0.92	Clonal	0.87	3.05			disease_causing	9.774		Passenger			Neutral	Tolerated
OVA_013_C3	PRIMARY	chrX	69457085	69457085	C	T	AWAT1	exonic	nonymous S	0000037452	388	168	556	0.302158	26	0	26	0	0.96	Clonal	0.91	3.01			disease_causing	9.774		Passenger			Neutral	Tolerated
OVA_003_M1	METASTASIS	chr11	69468115	69468115	C	T	AK294004	UTR3	NA	NA	521	47	568	0.082746	43	0	43	0	0.29	Sub	0.57	1.84			disease_causing	19.86						
OVA_003_M2	METASTASIS	chr11	69468115	69468115	C	T	AK294004	UTR3	NA	NA	562	36	598	0.060201	43	0	43	0	0.26	Sub	0.46	1.81			disease_causing	19.86						
OVA_003_M3	METASTASIS	chr11	69468115	69468115	C	T	AK294004	UTR3	NA	NA	567	24	591	0.040609	43	0	43	0	0.43	Sub	0.34	2.67			disease_causing	19.86						
OVA_048_M1	METASTASIS	chr10	69571270	69571270	C	T	DNAJC12	exonic	nonymous S	0000033975	403	197	600	0.328333	92	1	93	0.010753	1	Clonal	0.56	3.94			disease_causing	9.73		Passenger			Neutral	Tolerated
OVA_048_M2	METASTASIS	chr10	69571270	69571270	C	T	DNAJC12	exonic	nonymous S	0000033975	441	163	604	0.269868	92	1	93	0.010753	1	Clonal	0.4	3.87			disease_causing	9.73		Passenger			Neutral	Tolerated
OVA_048_M3	METASTASIS	chr10	69571270	69571270	C	T	DNAJC12	exonic	nonymous S	0000033975	389	149	538	0.276952	92	1	93	0.010753	1	Clonal	0.59	3.39			disease_causing	9.73		Passenger			Neutral	Tolerated
OVA_047_C1	PRIMARY	chr14	69993367	69993367	C	G	PLEKHD1	intronic	NA	NA	337	88	425	0.207059	86	0	86	0	0.69	Sub	0.6	2.91			polymorphism	0.987						
OVA_047_C2	PRIMARY	chr14	69993367	69993367	C	G	PLEKHD1	intronic	NA	NA	291	125	416	0.300481	86	0	86	0	0.94	Sub	0.64	2.76			polymorphism	0.987						
OVA_047_C3	Contralateral ovary	chr14	69993367	69993367	C	G	PLEKHD1	intronic	NA	NA	300	88	388	0.226804	86	0	86	0	0.76	Sub	0.6	2.94			polymorphism	0.987						
OVA_048_C1	PRIMARY	chrX	70281672	70281672	G	T	SNX12	intronic	NA	NA	58	10	68	0.147059	31	0	31	0	0.44	Sub	0.67	1.82			polymorphism	9.808						
OVA_048_C3	PRIMARY	chrX	70281672	70281672	G	T	SNX12	intronic	NA	NA	63	18	81	0.222222	31	0	31	0	0.6	Sub	0.74	1.8			polymorphism	9.808						
OVA_013_M1	METASTASIS	chrX	70340858	70340858	G	A	MED12	exonic	nonymous S	000037408	465	119	584	0.203767	79	1	80	0.0125	0.81	Sub	0.67	3.06	Yes		disease_causing	11.91		Passenger			Neutral	Tolerated
OVA_013_C1	PRIMARY	chrX	70340858	70340858	G	A	MED12	exonic	nonymous S	000037408	459	141	600	0.235	79	1	80	0.0125	0.89	Sub	0.72	3.1	Yes		disease_causing	11.91		Passenger			Neutral	Tolerated
OVA_013_C2	PRIMARY	chrX	70340858	70340858	G	A	MED12	exonic	nonymous S	000037408	452	154	606	0.254125	79	1	80	0.0125	0.84	Sub	0.87	3.05	Yes		disease_causing	11.91		Passenger			Neutral	Tolerated
OVA_013_C3	PRIMARY	chrX	70340858	70340858	G	A	MED12	exonic	nonymous S	000037408	486	161	647	0.248841	79	1	80	0.0125	0.79	Sub	0.91	3.01	Yes		disease_causing	11.91		Passenger			Neutral	Tolerated
OVA_003_M1	METASTASIS	chr14	70490193	70490193	T	G	SMOC1	intronic	NA	NA	244	82	326	0.251534	30	0	30	0	0.89	Sub	0.57	1.84			polymorphism	0.68						
OVA_003_M2	METASTASIS	chr14	70490193	70490193	T	G	SMOC1	intronic	NA	NA	332	60	392	0.153061	30	0	30	0	0.67	Sub	0.46	1.81			polymorphism	0.68						
OVA_003_M3	METASTASIS	chr14	70490193	70490193	T	G	SMOC1	intronic	NA	NA	506	26	532	0.048872	30	0	30	0	0.52	Sub	0.34	2.67			polymorphism	0.68						
OVA_003_C1	PRIMARY	chr14	70490193	70490193	T	G	SMOC1	intronic	NA	NA	276	183	459	0.398693	30	0	30	0	0.92	Clonal	0.86	1.81			polymorphism	0.68						
OVA_003_C2	PRIMARY	chr14	70490193	70490193	T	G	SMOC1	intronic	NA	NA	327	183	510	0.358824	30	0	30	0	1	Clonal	0.7	3.61			polymorphism	0.68						
OVA_003_C3	PRIMARY	chr14	70490193	70490193	T	G	SMOC1	intronic	NA	NA	339	212	551	0.384755	30	0	30	0	0.93	Clonal	0.82	1.81			polymorphism	0.68						
OVA_047_C3	Contralateral ovary	chr4	70504659	70504659	A	C	T2A1;UGT2	exonic	ynonymous	234A,ENSG	561	36	597	0.060302	31	0	31	0	0.26	Sub	0.6	2.94				17.42	p.S435A	Passenger	SENGER/OT	damaging	Neutral	Tolerated
OVA_047_C2	PRIMARY	chrX	70612828	70612828	G	T	TAF1	exonic	ynonymous	011V,ENSG	603	89	692	0.128613	80	0	80	0	0.53	Sub	0.64	2.76			disease_causing	29.6	p.G1032V	Passenger	SENGER/OT	damaging	Deleterious	Damaging
OVA_365_M1	METASTASIS	chr4	70620855	70620855	G	T	SULT1B1	exonic	ynonymous	T00000510	445	123	568	0.216549	45	0	45	0	0.51	Sub	0.85	1.87			disease_causing	12.79	p.N27K	Passenger	SENGER/OT	benign	Deleterious	Tolerated
OVA_048_M1	METASTASIS	chrX	70627565	70627565	C	T	BCYRN1	intronic	NA	NA	390	184	574	0.320557	55	0	55	0	1	Clonal	0.56	3.94			polymorphism	3.266						
OVA_048_M2	METASTASIS	chrX	70627565	70627565	C	T	BCYRN1	intronic	NA	NA	434	141	575	0.245217	55	0	55	0	1	Clonal	0.4	3.87			polymorphism	3.266						
OVA_048_M3	METASTASIS	chrX	70627565	70627565	C	T	BCYRN1	intronic	NA	NA	420	143	563	0.253996	55	0	55	0	1	Clonal	0.59	3.39			polymorphism	3.266						
OVA_048_C1	PRIMARY	chrX	70627565	70627565	C	T	BCYRN1	intronic	NA	NA	338	105	443	0.23702	55	0	55	0	0.71	Sub	0.67	1.82			polymorphism	3.266						
OVA_048_C2	PRIMARY	chrX	70627565	70627565	C	T	BCYRN1	intronic	NA	NA	370	172	542	0.317343	55	0	55	0	0.88	Sub	0.72	1.85			polymorphism	3.266						
OVA_048_C3	PRIMARY	chrX	70627565	70627565	C	T	BCYRN1	intronic	NA	NA	339	151	490	0.308163	55	0	55	0	0.83	Sub	0.74	1.8			polymorphism	3.266						
OVA_378_M1	METASTASIS	chr1	70696167	70696167	T	G	SRSF11	intronic	NA	NA	438	135	573	0.235602	24	0	24	0	1	Clonal	0.72	2.57			disease_causing	16.5						
OVA_378_M2	METASTASIS	chr1	70696167	70696167	T	G	SRSF11	intronic	NA	NA	401	161	562	0.286477	24	0	24	0	1	Clonal	0.85	2.57			disease_causing	16.5						
OVA_378_M3	METASTASIS	chr1	70696167	70696167	T	G	SRSF11	intronic	NA	NA	520	142	662	0.214502	24	0	24	0	1	Clonal	0.67	2.57			disease_causing	16.5						
OVA_378_C1	PRIMARY	chr1	70696167	70696167	T	G	SRSF11	intronic	NA	NA	449	134	583	0.229846	24	0	24	0	1	Clonal	0.59	2.6			disease_causing	16.5						
OVA_378_C2	PRIMARY	chr1	70696167	70696167	T	G	SRSF11	intronic	NA	NA	426	130	556	0.233813	24	0	24	0	1	Clonal	0.56	2.59			disease_causing	16.5						
OVA_378_C3	PRIMARY	chr1	70696167	70696167	T	G	SRSF11	intronic	NA	NA	416	131	547	0.239488	24	0	24	0	1	Clonal	0.51	2.6			disease_causing	16.5						
OVA_047_M3	METASTASIS	chr4	70897665	70897665	T	C	HTN3	intronic	NA	NA	172	3	175	0.017143	40	0	40	0	0.07	Sub	0.6	3.02			polymorphism	2.077						
OVA_047_C1	PRIMARY	chr4	70897665	70897665	T	C	HTN3	intronic	NA	NA	159	58	217	0.267281	40	0	40	0	1	Clonal	0.6	2.91			polymorphism	2.077						
OVA_047_C2	PRIMARY	chr4	70897665	70897665	T	C	HTN3	intronic	NA	NA	116	60	176	0.340909	40	0	40	0	1	Clonal	0.64	2.76			polymorphism	2.077						
OVA_047_C3	Contralateral ovary	chr4	70897665	70897665	T	C	HTN3	intronic	NA	NA	119	58	177	0.327684	40	0	40	0	1	Clonal	0.6	2.94			polymorphism	2.077						
OVA_047_C1	PRIMARY	chr3	71008541	71008541	G	A	FOXP1	exonic	ynonymous	I633Y,ENSG	503	139	642	0.216511	38	0	38	0	0.94	Clonal	0.6	2.91	Yes	No	disease_causing	28.9	p.H647Y	Driver		damaging	Neutral	Damaging
OVA_047_C2	PRIMARY	chr3	71008541	71008541	G	A	FOXP1	exonic	ynonymous	I633Y,ENSG	487	136	623	0.218299	38	0	38	0	0.9	Clonal	0.64	2.76	Yes	No	disease_causing	28.9	p.H647Y	Driver		damaging	Neutral	Damaging
OVA_047_C3	Contralateral ovary	chr3	71008541	71008541	G	A	FOXP1	exonic	ynonymous	I633Y,ENSG	509	118	627	0.188198	38	0	38	0	1	Clonal	0.6	2.94	Yes	No	disease_causing	28.9	p.H647Y	Driver		damaging	Neutral	Damaging
OVA_378_M1	METASTASIS	chr6	71012630	71012630	T	C	COL9A1	UTR5	NA	NA	432	81	513	0.157895	49	0	49	0	0.44	Sub	0.72	2.57			disease_causing	14.98						
OVA_378_M2	METASTASIS	chr6	71012630	71012630	T	C	COL9A1	UTR5	NA	NA	377	47	424	0.110849	49	0	49	0	0.26	Sub	0.85	2.57			disease_causing	14.98						
OVA_378_M3	METASTASIS	chr6	71012630	71012630	T	C	COL9A1	UTR5	NA	NA	471	75	546	0.137363	49	0	49	0	0.41	Sub	0.67	2.57			disease_causing	14.98						
OVA_047_C1	PRIMARY	chr2	71043453	71043453	G	A	CLEC4F	exonic	stopgain	Q354X,ENSG	620	66	686	0.09621	39	0	39	0	0.42	Sub	0.6	2.91			disease_causing_automat	36	p.Q354X	Passenger			NA	NA
OVA_048_M1	METASTASIS	chr11	71152288	71152298	GGGGAAG	-	DHCR7	exonic	neshift dele	00407721:e	833	402	1235	0.325506	38	0	38		1	Clonal	0.56	3.94					p.F201Qfs	Passenger			NA	NA
OVA_048_M2	METASTASIS	chr11	71152288	71152298	GGGGAAG	-	DHCR7	exonic	neshift dele	00407721:e	1047	316	1363	0.231842	38	0	38		1	Clonal	0.4	3.87					p.F201Qfs	Passenger			NA	NA
OVA_048_M3	METASTASIS	chr11	71152288	71152298	GGGGAAG	-	DHCR7	exonic	neshift dele	00407721:e	970	383	1353	0.283075	38	0	38		1	Clonal	0.59	3.39					p.F201Qfs	Passenger			NA	NA
OVA_048_M1	METASTASIS	chr11	71152305	71152305	-	A	DHCR7	exonic	neshift inse	00407721:e	914	788	1702	0.462985	52	0	52		1	Clonal	0.56	3.94			disease_causing		p.G199Wfs	Passenger				
OVA_048_M2	METASTASIS	chr11	71152305	71152305	-	A	DHCR7	exonic	neshift inse	00407721:e	1135	633	1768	0.358032	52	0	52		1	Clonal	0.4	3.87			disease_causing		p.G199Wfs	Passenger				
OVA_048_M3	METASTASIS	chr11	71152305	71152305	-	A	DHCR7	exonic	neshift inse	00407721:e	1070	756	1826	0.41402	52	0	52		1	Clonal	0.59	3.39			disease_causing		p.G199Wfs	Passenger				
OVA_048_C1	PRIMARY	chr16	71772973	71772973	C	T	AP1G1	exonic	ynonymous	717S,ENSG	305	152	457	0.332604	62	0	62	0	0.99	Clonal	0.67	1.82			disease_causing	26.3	p.G717S	Passenger	SENGER/OT	damaging	Deleterious	Tolerated
OVA_048_C2	PRIMARY	chr16	71772973	71772973	C	T	AP1G1	exonic	ynonymous	717S,ENSG	395	220	615	0.357724	62	0	62	0	1	Clonal	0.72	1.85			disease_causing	26.3	p.G717S	Passenger	SENGER/OT	damaging	Deleterious	Tolerated
OVA_048_C3	PRIMARY	chr16	71772973	71772973	C	T	AP1G1	exonic	ynonymous	717S,ENSG	324	188	512	0.367188	62	0	62	0	0.99	Clonal	0.74	1.8			disease_causing	26.3	p.G717S	Passenger	SENGER/OT	damaging	Deleterious	Tolerated
OVA_048_M1	METASTASIS	chr2	71797954	71797954	G	T	DYSF	intronic	NA	NA	398	240	638	0.376176	18	0	18	0	1	Clonal	0.56	3.94			polymorphism	4.876						
OVA_048_M2	METASTASIS	chr2	71797954	71797954	G	T	DYSF	intronic	NA	NA	471	163	634	0.257098	18	0	18	0	1	Clonal	0.4	3.87			polymorphism	4.876						
OVA_048_M3	METASTASIS	chr2	71797954	71797954	G	T	DYSF	intronic	NA	NA	452	152	604	0.251656	18	0	18	0	1	Clonal	0.59	3.39			polymorphism	4.876						
OVA_013_M1	METASTASIS	chr11	71821315	71821315	G	T	LRTOMT	UTR3	NA	NA	465	118	583	0.202401	29	0	29	0	1	Clonal	0.67	3.06			polymorphism	7.987						
OVA_013_C1	PRIMARY	chr11	71821315	71821315	G	T	LRTOMT	UTR3	NA	NA	497	128	625	0.2048	29	0	29	0	1	Clonal	0.72	3.1			polymorphism	7.987						
OVA_013_C2	PRIMARY	chr11	71821315	71821315	G	T	LRTOMT	UTR3	NA	NA	496	131	627	0.208931	29	0	29	0	1	Clonal	0.87	3.05			polymorphism	7.987						
OVA_013_C3	PRIMARY	chr11	71821315	71821315	G	T	LRTOMT	UTR3	NA	NA	521	126	647	0.194745	29	0	29	0	1	Clonal	0.91	3.01			polymorphism	7.987						
OVA_048_M3	METASTASIS	chr3	71834134	71834134	C	T	PROK2	exonic	ynonymous	.A24T,ENSG	35	6	41	0.146341	16	0	16	0	1	Clonal	0.59	3.39			polymorphism	7.124	p.A24T	Passenger	SENGER/OT	damaging	Neutral	Tolerated
OVA_048_M3	METASTASIS	chr3	71834147	71834147	C	G	PROK2	exonic	nonymous S	.L19L,ENSG	36	7	43	0.162791	16	0	16	0	1	Clonal	0.59	3.39			disease_causing	16		Passenger			Neutral	Tolerated
OVA_047_C1	PRIMARY	chr12	71978241	71978241	C	G	LGR5	exonic	nonymous S	0000540815	503	124	627	0.197767	39	0	39	0	0.66	Sub	0.6	2.91			disease_causing	16.51		Passenger			Neutral	Tolerated
OVA_047_C1	PRIMARY	chr16	72130025	72130025	G	C	DHX38	intronic	NA	NA	255	215	470	0.457447	37	0	37	0	1	Clonal	0.6	2.91			polymorphism	10.72						
OVA_047_C2	PRIMARY	chr16	72130025	72130025	G	C	DHX38	intronic	NA	NA	302	236	538	0.438662	37	0	37	0	0.93	Clonal	0.64	2.76			polymorphism	10.72						
OVA_047_C3	Contralateral ovary	chr16	72130025	72130025	G	C	DHX38	intronic	NA	NA	265	208	473	0.439746	37	0	37	0	1	Clonal	0.6	2.94			polymorphism	10.72						

Sample	Type	Chr	Start	End	Ref	Alt	Gene	unc.ensGen	icFunc.ens	hange.ens	t_ref	t_alt	t_total	t_VAF	n_ref	n_alt	n_total	normal_VA	ncer_cell_f	on-based C	purity	ploidy	Cancer Gene	Driver Gene	Mutation Taster	ADD(PHRE	otein_chan	CHASM	ATHMM_Pr	OLYPHEN-	REDICTION	ICTION (cutoff=0.05)
OVA_047_C1	PRIMARY	chr16	72146460	72146460	C	A	DHX38	UTR3	NA	NA	104	84	188	0.446809	18	0	18	0	1	Clonal	0.6	2.91			polymorphism	5.853						
OVA_047_C2	PRIMARY	chr16	72146460	72146460	C	A	DHX38	UTR3	NA	NA	98	95	193	0.492228	18	0	18	0	1	Clonal	0.64	2.76			polymorphism	5.853						
OVA_047_C3	Contralateral ovary	chr16	72146460	72146460	C	A	DHX38	UTR3	NA	NA	81	83	164	0.506098	18	0	18	0	1	Clonal	0.6	2.94			polymorphism	5.853						
OVA_378_C3	PRIMARY	chr11	72289478	72289478	C	A	PDE2A	intronic	NA	NA	355	25	380	0.065789	66	0	66	0	0.32	Sub	0.51	2.6			polymorphism	12.29						
OVA_003_M1	METASTASIS	chr11	72295491	72295491	C	T	PDE2A	exonic	ionymous S	T00000441	135	48	183	0.262295	88	0	88	0	0.93	Sub	0.57	1.84			polymorphism	10.08					Neutral	Damaging
OVA_003_M2	METASTASIS	chr11	72295491	72295491	C	T	PDE2A	exonic	ionymous S	T00000441	189	41	230	0.178261	88	0	88	0	0.78	Sub	0.46	1.81			polymorphism	10.08					Neutral	Damaging
OVA_003_M3	METASTASIS	chr11	72295491	72295491	C	T	PDE2A	exonic	ionymous S	T00000441	232	14	246	0.056911	88	0	88	0	0.6	Sub	0.34	2.67			polymorphism	10.08					Neutral	Damaging
OVA_003_C1	PRIMARY	chr11	72295491	72295491	C	T	PDE2A	exonic	ionymous S	T00000441	127	127	254	0.5	88	0	88	0	1	Clonal	0.86	1.81			polymorphism	10.08					Neutral	Damaging
OVA_003_C2	PRIMARY	chr11	72295491	72295491	C	T	PDE2A	exonic	ionymous S	T00000441	198	131	329	0.398176	88	0	88	0	1	Clonal	0.7	3.61			polymorphism	10.08					Neutral	Damaging
OVA_003_C3	PRIMARY	chr11	72295491	72295491	C	T	PDE2A	exonic	ionymous S	T00000441	169	197	366	0.538251	88	0	88	0	1	Clonal	0.82	1.81			polymorphism	10.08					Neutral	Damaging
OVA_048_M1	METASTASIS	chr5	72337125	72337125	C	T	FCHO2	exonic	ynonymou	000034184	42	61	103	0.592233	46	1	47	0.021277	1	Clonal	0.56	3.94			disease_causing	15.97	p.P308S	Passenger	SENGER/OT	benign	Deleterious	Tolerated
OVA_048_M2	METASTASIS	chr5	72337125	72337125	C	T	FCHO2	exonic	ynonymou	000034184	95	60	155	0.387097	46	1	47	0.021277	1	Clonal	0.4	3.87			disease_causing	15.97	p.P308S	Passenger	SENGER/OT	benign	Deleterious	Tolerated
OVA_048_M3	METASTASIS	chr5	72337125	72337125	C	T	FCHO2	exonic	ynonymou	000034184	56	66	122	0.540984	46	1	47	0.021277	1	Clonal	0.59	3.39			disease_causing	15.97	p.P308S	Passenger	SENGER/OT	benign	Deleterious	Tolerated
OVA_048_C1	PRIMARY	chr5	72337125	72337125	C	T	FCHO2	exonic	ynonymou	000034184	59	92	151	0.609272	46	1	47	0.021277	1	Clonal	0.67	1.82			disease_causing	15.97	p.P308S	Passenger	SENGER/OT	benign	Deleterious	Tolerated
OVA_048_C2	PRIMARY	chr5	72337125	72337125	C	T	FCHO2	exonic	ynonymou	000034184	48	52	100	0.52	46	1	47	0.021277	0.93	Clonal	0.72	1.85			disease_causing	15.97	p.P308S	Passenger	SENGER/OT	benign	Deleterious	Tolerated
OVA_048_C3	PRIMARY	chr5	72337125	72337125	C	T	FCHO2	exonic	ynonymou	000034184	46	65	111	0.585586	46	1	47	0.021277	1	Clonal	0.74	1.8			disease_causing	15.97	p.P308S	Passenger	SENGER/OT	benign	Deleterious	Tolerated
OVA_003_M1	METASTASIS	chr12	72416305	72416305	C	A	TPH2	intronic	NA	NA	361	146	507	0.287968	51	0	51	0	1	Sub	0.57	1.84			polymorphism	8.218						
OVA_003_M2	METASTASIS	chr12	72416305	72416305	C	A	TPH2	intronic	NA	NA	413	133	546	0.24359	51	0	51	0	1	Sub	0.46	1.81			polymorphism	8.218						
OVA_003_M3	METASTASIS	chr12	72416305	72416305	C	A	TPH2	intronic	NA	NA	532	46	578	0.079585	51	0	51	0	0.84	Sub	0.34	2.67			polymorphism	8.218						
OVA_003_C1	PRIMARY	chr12	72416305	72416305	C	A	TPH2	intronic	NA	NA	310	260	570	0.45614	51	0	51	0	1	Clonal	0.86	1.81			polymorphism	8.218						
OVA_003_C2	PRIMARY	chr12	72416305	72416305	C	A	TPH2	intronic	NA	NA	364	218	582	0.37457	51	0	51	0	1	Clonal	0.7	3.61			polymorphism	8.218						
OVA_003_C3	PRIMARY	chr12	72416305	72416305	C	A	TPH2	intronic	NA	NA	349	240	589	0.40747	51	0	51	0	0.99	Clonal	0.82	1.81			polymorphism	8.218						
OVA_048_C1	PRIMARY	chr15	72461917	72461917	G	-	GRAMD2	intronic	NA	NA	290	97	387	0.250646	44	0	44		1	Sub	0.67	1.82										
OVA_048_C3	PRIMARY	chr15	72461917	72461917	G	-	GRAMD2	intronic	NA	NA	210	58	268	0.216418	44	0	44		0.8	Sub	0.74	1.8										
OVA_048_M3	METASTASIS	chr17	72868835	72868835	C	A	FDXR	intronic	NA	NA	388	102	490	0.208163	25	0	25	0	1	Clonal	0.59	3.39			polymorphism	4.78						
OVA_048_C1	PRIMARY	chr14	72944925	72944925	A	G	RGS6	intronic	NA	NA	235	129	364	0.354396	30	0	30	0	1	Clonal	0.67	1.82			polymorphism	1.03						
OVA_048_C2	PRIMARY	chr14	72944925	72944925	A	G	RGS6	intronic	NA	NA	311	195	506	0.385375	30	0	30	0	1	Clonal	0.72	1.85			polymorphism	1.03						
OVA_048_C3	PRIMARY	chr14	72944925	72944925	A	G	RGS6	intronic	NA	NA	244	143	387	0.369509	30	0	30	0	1	Clonal	0.74	1.8			polymorphism	1.03						
OVA_047_C1	PRIMARY	chrX	73069408	73069408	T	C	XIST	cRNA_exon	NA	NA	567	156	723	0.215768	40	0	40	0	1	Sub	0.6	2.91				5.403						
OVA_047_C2	PRIMARY	chrX	73069408	73069408	T	C	XIST	cRNA_exon	NA	NA	575	140	715	0.195804	40	0	40	0	0.81	Sub	0.64	2.76				5.403						
OVA_047_C3	Contralateral ovary	chrX	73069408	73069408	T	C	XIST	cRNA_exon	NA	NA	592	148	740	0.2	40	0	40	0	0.87	Clonal	0.6	2.94				5.403						
OVA_047_C1	PRIMARY	chr9	73296445	73296445	G	T	TRPM3	exonic	ynonymou	3D,ENSG00	590	126	716	0.175978	76	0	76	0	0.76	Sub	0.6	2.91			disease_causing	26.8	p.A443D	Passenger	SENGER/OT	damaging	Deleterious	Damaging
OVA_047_C2	PRIMARY	chr9	73296445	73296445	G	T	TRPM3	exonic	ynonymou	3D,ENSG00	559	133	692	0.192197	76	0	76	0	0.79	Sub	0.64	2.76			disease_causing	26.8	p.A443D	Passenger	SENGER/OT	damaging	Deleterious	Damaging
OVA_047_C3	Contralateral ovary	chr9	73296445	73296445	G	T	TRPM3	exonic	ynonymou	3D,ENSG00	618	95	713	0.13324	76	0	76	0	0.85	Clonal	0.6	2.94			disease_causing	26.8	p.A443D	Passenger	SENGER/OT	damaging	Deleterious	Damaging
OVA_013_M1	METASTASIS	chr15	73418963	73418963	C	T	NEO1	intronic	NA	NA	416	175	591	0.296108	29	0	29	0	1	Clonal	0.67	3.06			polymorphism	4.775						
OVA_013_C1	PRIMARY	chr15	73418963	73418963	C	T	NEO1	intronic	NA	NA	396	177	573	0.308901	29	0	29	0	1	Clonal	0.72	3.1			polymorphism	4.775						
OVA_013_C2	PRIMARY	chr15	73418963	73418963	C	T	NEO1	intronic	NA	NA	391	143	534	0.26779	29	0	29	0	0.88	Clonal	0.87	3.05			polymorphism	4.775						
OVA_013_C3	PRIMARY	chr15	73418963	73418963	C	T	NEO1	intronic	NA	NA	401	178	579	0.307427	29	0	29	0	0.98	Clonal	0.91	3.01			polymorphism	4.775						
OVA_378_M1	METASTASIS	chr7	73474203	73474205	CTC	-	ELN	exonic	ameshift de	74del,ENSG	233	131	364	0.35989	86	0	86		1	Clonal	0.72	2.57	Yes		disease causing		p.L474del	Passenger			Neutral	NA
OVA_378_M2	METASTASIS	chr7	73474203	73474205	CTC	-	ELN	exonic	ameshift de	74del,ENSG	184	146	330	0.442424	86	0	86		1	Clonal	0.85	2.57	Yes		disease causing		p.L474del	Passenger			Neutral	NA
OVA_378_M3	METASTASIS	chr7	73474203	73474205	CTC	-	ELN	exonic	ameshift de	74del,ENSG	341	172	513	0.335283	86	0	86		1	Clonal	0.67	2.57	Yes		disease causing		p.L474del	Passenger			Neutral	NA
OVA_378_C1	PRIMARY	chr7	73474203	73474205	CTC	-	ELN	exonic	ameshift de	74del,ENSG	509	159	668	0.238024	86	0	86		1	Clonal	0.59	2.6	Yes		disease causing		p.L474del	Passenger			Neutral	NA
OVA_378_C2	PRIMARY	chr7	73474203	73474205	CTC	-	ELN	exonic	ameshift de	74del,ENSG	629	162	791	0.204804					0.93	Clonal	0.56	2.59	Yes		disease causing		p.L474del	Passenger			Neutral	NA
OVA_378_C3	PRIMARY	chr7	73474203	73474205	CTC	-	ELN	exonic	ameshift de	74del,ENSG	606	142	748	0.18984					0.93	Clonal	0.51	2.6	Yes		disease causing		p.L474del	Passenger			Neutral	NA
OVA_047_C1	PRIMARY	chr10	73545427	73545427	C	T	CDH23	exonic	ynonymou	00224721:	561	48	609	0.078818	76	0	76	0	0.26	Sub	0.6	2.91			disease_causing	23.3	p.R1923W	Passenger		damaging	Deleterious	Damaging
OVA_048_C1	PRIMARY	chr14	73720550	73720550	G	A	PAPLN	exonic	ynonymou	000555445	42	13	55	0.236364	49	0	49	0	0.71	Sub	0.67	1.82			polymorphism	1.56	p.A395T	Passenger	SENGER/OT	benign	Neutral	Tolerated
OVA_003_C1	PRIMARY	chr7	73752902	73752902	C	G	CLIP2	exonic	ionymous S	000022339	104	11	115	0.095652	69	0	69	0	0.22	Sub	0.86	1.81			disease_causing	10.96		Passenger			Neutral	Tolerated
OVA_003_M1	METASTASIS	chr2	73836705	73836705	A	G	ALMS1	exonic	ynonymou	4115S,ENSG	311	88	399	0.220551	31	0	31	0	0.78	Sub	0.57	1.84			disease_causing	22.9	p.N4158S	Passenger		damaging	Deleterious	Damaging
OVA_003_M2	METASTASIS	chr2	73836705	73836705	A	G	ALMS1	exonic	ynonymou	4115S,ENSG	400	102	502	0.203187	31	0	31	0	0.89	Sub	0.46	1.81			disease_causing	22.9	p.N4158S	Passenger		damaging	Deleterious	Damaging
OVA_003_M3	METASTASIS	chr2	73836705	73836705	A	G	ALMS1	exonic	ynonymou	4115S,ENSG	506	35	541	0.064695	31	0	31	0	0.68	Sub	0.34	2.67			disease_causing	22.9	p.N4158S	Passenger		damaging	Deleterious	Damaging
OVA_003_C1	PRIMARY	chr2	73836705	73836705	A	G	ALMS1	exonic	ynonymou	4115S,ENSG	275	188	463	0.406048	31	0	31	0	0.94	Clonal	0.86	1.81			disease_causing	22.9	p.N4158S	Passenger		damaging	Deleterious	Damaging
OVA_003_C2	PRIMARY	chr2	73836705	73836705	A	G	ALMS1	exonic	ynonymou	4115S,ENSG	330	215	545	0.394495	31	0	31	0	1	Clonal	0.7	3.61			disease_causing	22.9	p.N4158S	Passenger		damaging	Deleterious	Damaging
OVA_003_C3	PRIMARY	chr2	73836705	73836705	A	G	ALMS1	exonic	ynonymou	4115S,ENSG	337	214	551	0.388385	31	0	31	0	0.94	Clonal	0.82	1.81			disease_causing	22.9	p.N4158S	Passenger		damaging	Deleterious	Damaging
OVA_048_M1	METASTASIS	chr6	73951871	73951871	G	C	KHDC1	exonic	ynonymou	R68G,ENSG	207	390	597	0.653266	23	0	23	0	1	Clonal	0.56	3.94			polymorphism	21.3	p.R141G	Passenger	SENGER/OT	damaging	Deleterious	NA
OVA_048_M2	METASTASIS	chr6	73951871	73951871	G	C	KHDC1	exonic	ynonymou	R68G,ENSG	350	306	656	0.466463	23	0	23	0	1	Clonal	0.4	3.87			polymorphism	21.3	p.R141G	Passenger	SENGER/OT	damaging	Deleterious	NA
OVA_048_M3	METASTASIS	chr6	73951871	73951871	G	C	KHDC1	exonic	ynonymou	R68G,ENSG	212	389	601	0.647255	23	0	23	0	1	Clonal	0.59	3.39			polymorphism	21.3	p.R141G	Passenger	SENGER/OT	damaging	Deleterious	NA
OVA_365_M1	METASTASIS	chrX	73961184	73961184	C	G	KIAA2022	exonic	ynonymou	000055682	62	325	387	0.839793	49	0	49	0	1	Clonal	0.85	1.87			disease_causing	26.3	p.E1070Q	Passenger		damaging	Neutral	Damaging
OVA_365_M2	METASTASIS	chrX	73961184	73961184	C	G	KIAA2022	exonic	ynonymou	000055682	210	75	285	0.263158	49	0	49	0	1	Clonal	0.4	1.81			disease_causing	26.3	p.E1070Q	Passenger		damaging	Neutral	Damaging
OVA_365_M3	METASTASIS	chrX	73961184	73961184	C	G	KIAA2022	exonic	ynonymou	000055682	66	239	305	0.783607	49	0	49	0	1	Clonal	0.82	1.89			disease_causing	26.3	p.E1070Q	Passenger		damaging	Neutral	Damaging
OVA_365_C1	Contralateral ovary	chrX	73961184	73961184	C	G	KIAA2022	exonic	ynonymou	000055682	192	81	273	0.296703	49	0	49	0	1	Clonal	0.42	1.83			disease_causing	26.3	p.E1070Q	Passenger		damaging	Neutral	Damaging
OVA_365_C2	PRIMARY	chrX	73961184	73961184	C	G	KIAA2022	exonic	ynonymou	000055682	139	142	281	0.505338	49	0	49	0	1	Clonal	0.62	1.92			disease_causing	26.3	p.E1070Q	Passenger		damaging	Neutral	Damaging
OVA_047_C1	PRIMARY	chr14	73963353	73963353	G	T	HEATR4	exonic	ynonymou	000560393	527	96	623	0.154093	39	0	39	0	0.51	Sub	0.6	2.91			polymorphism	16.4	p.N859K	Passenger	SENGER/OT	benign	Neutral	Tolerated
OVA_047_C3	Contralateral ovary	chr14	73963353	73963353	G	T	HEATR4	exonic	ynonymou	000560393	485	102	587	0.173765	39	0	39	0	0.58	Sub	0.6	2.94			polymorphism	16.4	p.N859K	Passenger	SENGER/OT	benign	Neutral	Tolerated
OVA_003_M1	METASTASIS	chr7	74016744	74016744	G	T	GTF2IRD1	exonic	ynonymou	000424337	383	158	541	0.292052	36	0	36	0	1	Clonal	0.57	1.84			disease_causing	27.1	p.G972V	Passenger	SENGER/OT	damaging	Deleterious	Damaging
OVA_003_M2	METASTASIS	chr7	74016744	74016744	G	T	GTF2IRD1	exonic	ynonymou	000424337	450	113	563	0.20071	36	0	36	0	0.88	Clonal	0.46	1.81			disease_causing	27.1	p.G972V	Passenger	SENGER/OT	damaging	Deleterious	Damaging
OVA_003_M3	METASTASIS	chr7	74016744	74016744	G	T	GTF2IRD1	exonic	ynonymou	000424337	506	57	563	0.101243	36	0	36	0	1	Clonal	0.34	2.67			disease_causing	27.1	p.G972V	Passenger	SENGER/OT	damaging	Deleterious	Damaging
OVA_003_C1	PRIMARY	chr7	74016744	74016744	G	T	GTF2IRD1	exonic	ynonymou	000424337	340	253	593	0.426644	36	0	36	0	0.99	Clonal	0.86	1.81			disease_causing	27.1	p.G972V	Passenger	SENGER/OT	damaging	Deleterious	Damaging
OVA_003_C2	PRIMARY	chr7	74016744	74016744	G	T	GTF2IRD1	exonic	ynonymou	000424337	370	194	564	0.343972	36	0	36	0	1	Clonal	0.7	3.61			disease_causing	27.1	p.G972V	Passenger	SENGER/OT	damaging	Deleterious	Damaging
OVA_003_C3	PRIMARY	chr7	74016744	74016744	G	T	GTF2IRD1	exonic	ynonymou	000424337	330	255	585	0.435897	36	0	36	0	1	Clonal	0.82	1.81			disease_causing	27.1	p.G972V	Passenger	SENGER/OT	damaging	Deleterious	Damaging
OVA_048_M1	METASTASIS	chr2	74042301	74042301	C	T	C2orf78	exonic	ionymous S	000004095	477	74	551	0.134301	26	0	26	0	0.75	Sub	0.56	3.94			disease_causing	5.893		Passenger			Neutral	Tolerated
OVA_048_M2	METASTASIS	chr2	74042301	74042301	C	T	C2orf78	exonic	ionymous S	000004095	509	31	540	0.057407	26	0	26	0	0.4	Sub	0.4	3.87			disease_causing	5.893		Passenger			Neutral	Tolerated
OVA_047_C1	PRIMARY	chr14	74058650	74058650	G	A	ACOT4	UTR5	NA	NA	84	24	108	0.222222	44	0	44	0	0.74	Sub	0.6	2.91			polymorphism	9.227						
OVA_047_C2	PRIMARY	chr14	74058650	74058650	G	A	ACOT4	UTR5	NA	NA	58	34	92	0.369565	44	0	44	0	1	Sub	0.64	2.76			polymorphism	9.227						
OVA_047_C3	Contralateral ovary	chr14	74058650	74058650	G	A	ACOT4	UTR5	NA	NA	79	34	113	0.300885	44	0	44	0	1	Clonal	0.6	2.94			polymorphism	9.227						
OVA_003_C1	PRIMARY	chr6	74189439	74189439	G	-	MTO1	intronic	NA	NA	682	482	1164	0.414089	17	0	17		0.96	Clonal	0.86	1.81										
OVA_003_C2	PRIMARY	chr6	74189439	74189439	G	-	MTO1	intronic	NA	NA	754	530	1284	0.412773	17	0	17		1	Clonal	0.7	3.61										
OVA_003_C3	PRIMARY	chr6	74189439	74189439	G	-	MTO1	intronic	NA	NA	855	573	1428	0.401261	17	0	17		0.97	Clonal	0.82	1.81										
OVA_003_C1	PRIMARY	chr4	74279303	74279303	A	T	ALB	exonic	ynonymou	000503124	532	10	542	0.01845	54	0	54	0	0.02	Sub	0.86	1.81			disease_causing	23.8	p.K337M	Passenger	SENGER/OT	damaging	Deleterious	Damaging
OVA_003_C2	PRIMARY	chr4	74279303	74279303	A	T	ALB	exonic	ynonymou	000503124	474	93	567	0.164021	54	0	54	0	0.47	Sub	0.7	3.61			disease_causing	23.8	p.K337M	Passenger	SENGER/OT	damaging	Deleterious	Damaging
OVA_048_M1	METASTASIS	chrX	74284911	74284911	G	A	ABCB7	exonic	ynonymou	.583F,ENSG	383	220	603	0.364842	51	0	51	0	1	Clonal	0.56	3.94			disease_causing	25.9	p.L610F	Passenger	SENGER/OT	damaging	Deleterious	Damaging
OVA_048_M2	METASTASIS	chrX	74284911	74284911	G	A	ABCB7	exonic	ynonymou	.583F,ENSG	461	153	614	0.249186	51	0	51	0	1	Clonal	0.4	3.87			disease_causing	25.9	p.L610F	Passenger	SENGER/OT	damaging	Deleterious	Damaging
OVA_048_M3	METASTASIS	chrX	74284911	74284911	G	A	ABCB7	exonic	ynonymou	.583F,ENSG	447	130	577	0.225303	51	0	51	0	1	Clonal	0.59	3.39			disease_causing	25.9	p.L610F	Passenger	SENGER/OT	damaging	Deleterious	Damaging
OVA_048_C1	PRIMARY	chrX	74284911	74284911	G	A	ABCB7	exonic	ynonymou	.583F,ENSG	296	131	427	0.306792	51	0	51	0	0.92	Clonal	0.67	1.82			disease_causing	25.9	p.L610F	Passenger	SENGER/OT	damaging	Deleterious	Damaging
OVA_048_C2	PRIMARY	chrX	74284911	74284911	G	A	ABCB7	exonic	ynonymou	.583F,ENSG	377	211	588	0.358844	51	0	51	0	1	Clonal	0.72	1.85			disease_causing	25.9	p.L610F	Passenger	SENGER/OT	damaging	Deleterious	Damaging
OVA_048_C3	PRIMARY	chrX	74284911	74284911	G	A	ABCB7	exonic	ynonymou	.583F,ENSG	299	146	445	0.32809	51	0	51	0	0.89	Clonal	0.74	1.8			disease_causing	25.9	p.L610F	Passenger	SENGER/OT	damaging	Deleterious	Damaging
OVA_003_M1	METASTASIS	chr17	74289943	74289954	GATCCTGA	-	QRICH2	exonic	ameshift de	262765:exo	271	86	357	0.240896	73	0	73		0.85	Sub	0.57	1.84			disease causing		A119_S122	Passenger			Deleterious	NA
OVA_003_M2	METASTASIS	chr17	74289943	74289954	GATCCTGA	-	QRICH2	exonic	ameshift de	262765:exo	648	159	807	0.197026	73	0	73		0.86	Sub	0.46	1.81			disease causing		A119_S122	Passenger			Deleterious	NA
OVA_003_M3	METASTASIS	chr17	74289943	74289954	GATCCTGA	-	QRICH2	exonic	ameshift de	262765:exo	722	58	780	0.074359	73	0	73		0.78	Sub	0.34	2.67			disease causing		A119_S122	Passenger			Deleterious	NA
OVA_003_C1	PRIMARY	chr17	74289943	74289954	GATCCTGA	-	QRICH2	exonic	ameshift de	262765:exo	257	156	413	0.377724	73	0	73		0.88	Sub	0.86	1.81			disease causing		A119_S122	Passenger			Deleterious	NA
OVA_003_C2	PRIMARY	chr17	74289943	74289954	GATCCTGA	-	QRICH2	exonic	ameshift de	262765:exo	229	183	412	0.444175	73	0	73		1	Sub	0.7	3.61			disease causing		A119_S122	Passenger			Deleterious	NA
OVA_003_C3	PRIMARY	chr17	74289943	74289954	GATCCTGA	-	QRICH2	exonic	ameshift de	262765:exo	318	179	497	0.360161	73	0	73		0.87	Sub	0.82	1.81			disease causing		A119_S122	Passenger			Deleterious	NA
OVA_003_M1	METASTASIS	chr3	74349018	74349018	C	T	CNTN3	splicing	NA	NA	290	10	300	0.033333	38	0	38	0	0.12	Sub	0.57	1.84			disease_causing	34		Passenger				
OVA_003_M2	METASTASIS	chr3	74349018	74349018	C	T	CNTN3	splicing	NA	NA	323	14	337	0.041543	38	0	38	0	0.18	Sub	0.46	1.81			disease_causing	34		Passenger				
OVA_003_M3	METASTASIS	chr3	74349018	74349018	C	T	CNTN3	splicing	NA	NA	820	9	829	0.010856	38	0	38	0	0.11	Sub	0.34	2.67			disease_causing	34		Passenger				
OVA_003_C2	PRIMARY	chr3	74349018	74349018	C	T	CNTN3	splicing	NA	NA	876	18	894	0.020134	38	0	38	0	0.1	Sub	0.7	3.61			disease_causing	34		Passenger				
OVA_013_M1	METASTASIS	chr9	74533195	74533195	A	G	C9orf85	intronic	NA	NA	249	86	335	0.256716	12	0	12	0	1	Clonal	0.67	3.06			polymorphism	0.21						
OVA_013_C1	PRIMARY	chr9	74533195	74533195	A	G	C9orf85	intronic	NA	NA	216	54	270	0.2	12	0	12	0	0.95	Clonal	0.72	3.1			polymorphism	0.21						
OVA_013_C2	PRIMARY	chr9	74533195	74533195	A	G	C9orf85	intronic	NA	NA	169	53	222	0.238739	12	0	12	0	1	Clonal	0.87	3.05			polymorphism	0.21						
OVA_013_C3	PRIMARY	chr9	74533195	74533195	A	G	C9orf85	intronic	NA	NA	249	88	337	0.261128	12	0	12	0	1	Clonal	0.91	3.01			polymorphism	0.21						
OVA_047_C1	PRIMARY	chr3	74535619	74535619	G	C	CNTN3	exonic	ynonymou	000026366	600	76	676	0.112426	39	0	39	0	0.49	Sub	0.6	2.91			disease_causing	22.1	p.L116V	Passenger	SENGER/OT	benign	Neutral	Tolerated

Sample	Type	Chr	Start	End	Ref	Alt	Gene	unc.ensGen	icFunc.ens	hange.ens	t_ref	t_alt	t_total	t_VAF	n_ref	n_alt	n_total	normal_VA	ncer_cell_f	on-based C	purity	ploidy	Cancer Gene	Driver Gene	Mutation Taster	ADD(PHRE	otein_chan	CHASM	ATHMM_Pr	OLYPHEN-	REDICTION	ICTION (cutoff=0.05)
OVA_047_C3	Contralateral ovary	chr3	74535619	74535619	G	C	CNTN3	exonic	ynonymou	000026366	586	91	677	0.134417	39	0	39	0	0.72	Sub	0.6	2.94			disease_causing	22.1	p.L116V	Passenger	SENGER/OT	benign	Neutral	Tolerated
OVA_003_M1	METASTASIS	chr5	74647154	74647154	C	G	HMGCR	intronic	NA	NA	418	177	595	0.297479	61	0	61	0	1	Sub	0.57	1.84			polymorphism	1.275						
OVA_003_M2	METASTASIS	chr5	74647154	74647154	C	G	HMGCR	intronic	NA	NA	484	127	611	0.207856	61	0	61	0	0.91	Sub	0.46	1.81			polymorphism	1.275						
OVA_003_M3	METASTASIS	chr5	74647154	74647154	C	G	HMGCR	intronic	NA	NA	585	45	630	0.071429	61	0	61	0	0.75	Sub	0.34	2.67			polymorphism	1.275						
OVA_003_C1	PRIMARY	chr5	74647154	74647154	C	G	HMGCR	intronic	NA	NA	381	269	650	0.413846	61	0	61	0	0.96	Clonal	0.86	1.81			polymorphism	1.275						
OVA_003_C2	PRIMARY	chr5	74647154	74647154	C	G	HMGCR	intronic	NA	NA	365	262	627	0.417863	61	0	61	0	1	Clonal	0.7	3.61			polymorphism	1.275						
OVA_003_C3	PRIMARY	chr5	74647154	74647154	C	G	HMGCR	intronic	NA	NA	381	272	653	0.416539	61	0	61	0	1	Clonal	0.82	1.81			polymorphism	1.275						
OVA_047_M1	METASTASIS	chr2	74656043	74656043	T	C	RTKN	exonic	ynonymou	000027243	549	132	681	0.193833	40	0	40	0	1	Clonal	0.47	2.01			disease_causing	25.2	p.H261R	Passenger	SENGER/OT	damaging	Deleterious	Tolerated
OVA_047_M2	METASTASIS	chr2	74656043	74656043	T	C	RTKN	exonic	ynonymou	000027243	580	132	712	0.185393	40	0	40	0	1	Clonal	0.44	2.01			disease_causing	25.2	p.H261R	Passenger	SENGER/OT	damaging	Deleterious	Tolerated
OVA_047_M3	METASTASIS	chr2	74656043	74656043	T	C	RTKN	exonic	ynonymou	000027243	445	116	561	0.206774	40	0	40	0	1	Clonal	0.6	3.02			disease_causing	25.2	p.H261R	Passenger	SENGER/OT	damaging	Deleterious	Tolerated
OVA_047_C1	PRIMARY	chr2	74750089	74750089	T	C	DQX1	exonic	nonymous S	399P,ENSG	406	66	472	0.139831	23	0	23	0	0.61	Sub	0.6	2.91			polymorphism	15.82		Passenger			Neutral	Tolerated
OVA_365_M3	METASTASIS	chr2	74782367	74782367	C	G	DOK1	exonic	ynonymou	.S49R,ENSG	156	13	169	0.076923	112	0	112	0	0.19	Sub	0.82	1.89			polymorphism	22.8	p.S49R	Passenger	SENGER/OT	benign	Neutral	Tolerated
OVA_013_M1	METASTASIS	chr4	74864333	74864333	T	A	CXCL5	UTR5	NA	NA	253	97	350	0.277143	71	0	71	0	1	Clonal	0.67	3.06			polymorphism	10.03						
OVA_013_C1	PRIMARY	chr4	74864333	74864333	T	A	CXCL5	UTR5	NA	NA	277	116	393	0.295165	71	0	71	0	1	Clonal	0.72	3.1			polymorphism	10.03						
OVA_013_C2	PRIMARY	chr4	74864333	74864333	T	A	CXCL5	UTR5	NA	NA	263	116	379	0.306069	71	0	71	0	1	Clonal	0.87	3.05			polymorphism	10.03						
OVA_013_C3	PRIMARY	chr4	74864333	74864333	T	A	CXCL5	UTR5	NA	NA	311	149	460	0.323913	71	0	71	0	1	Clonal	0.91	3.01			polymorphism	10.03						
OVA_365_C2	PRIMARY	chr1	74930423	74930423	A	G	PGT-TNNI3	exonic	nonymous S	000370899	374	139	513	0.270955	30	0	30	0	0.88	Clonal	0.62	1.92			polymorphism	8.019		Passenger			Neutral	Tolerated
OVA_047_C1	PRIMARY	chr11	74953132	74953132	C	A	TPBGL	exonic	ynonymou	000056219	279	117	396	0.295455	75	0	75	0	1	Clonal	0.6	2.91				26.4	p.D346E	Passenger	SENGER/OT			
OVA_047_C2	PRIMARY	chr11	74953132	74953132	C	A	TPBGL	exonic	ynonymou	000056219	221	149	370	0.402703	75	0	75	0	1	Clonal	0.64	2.76				26.4	p.D346E	Passenger	SENGER/OT			
OVA_047_C3	Contralateral ovary	chr11	74953132	74953132	C	A	TPBGL	exonic	ynonymou	000056219	209	64	273	0.234432	75	0	75	0	1	Clonal	0.6	2.94				26.4	p.D346E	Passenger	SENGER/OT			
OVA_047_C1	PRIMARY	chr14	75016542	75016542	G	A	LTBP2	intronic	NA	NA	262	61	323	0.188854	60	0	60	0	0.63	Sub	0.6	2.91			polymorphism	0.507						
OVA_365_C2	PRIMARY	chr10	75187985	75187985	T	C	MSS51	exonic	ynonymou	T00000299	472	54	526	0.102662	97	0	97	0	0.33	Sub	0.62	1.92			polymorphism	0.599	p.I20V	Passenger	SENGER/OT	benign	Neutral	Tolerated
OVA_048_M1	METASTASIS	chr15	75521934	75521934	G	A	orf39;GOLG	cRNA_exon	NA	NA	64	99	163	0.607362	50	0	50	0	1	Clonal	0.56	3.94				6.467						
OVA_048_M2	METASTASIS	chr15	75521934	75521934	G	A	orf39;GOLG	cRNA_exon	NA	NA	101	61	162	0.376543	50	0	50	0	1	Clonal	0.4	3.87				6.467						
OVA_048_M3	METASTASIS	chr15	75521934	75521934	G	A	orf39;GOLG	cRNA_exon	NA	NA	66	73	139	0.52518	50	0	50	0	1	Clonal	0.59	3.39				6.467						
OVA_048_C1	PRIMARY	chr15	75521934	75521934	G	A	orf39;GOLG	cRNA_exon	NA	NA	29	33	62	0.532258	50	0	50	0	1	Clonal	0.67	1.82				6.467						
OVA_048_C2	PRIMARY	chr15	75521934	75521934	G	A	orf39;GOLG	cRNA_exon	NA	NA	49	78	127	0.614173	50	0	50	0	1	Clonal	0.72	1.85				6.467						
OVA_048_C3	PRIMARY	chr15	75521934	75521934	G	A	orf39;GOLG	cRNA_exon	NA	NA	24	51	75	0.68	50	0	50	0	1	Clonal	0.74	1.8				6.467						
OVA_365_M1	METASTASIS	chr10	75832579	75832579	C	T	VCL	exonic	nonymous S	197T,ENSG	350	230	580	0.396552	32	0	32	0	0.94	Clonal	0.85	1.87			disease_causing	2.055		Passenger			Neutral	Tolerated
OVA_013_M1	METASTASIS	chr6	75898197	75898197	A	G	COL12A1	exonic	ynonymou	00003225	481	167	648	0.257716	45	0	45	0	1	Clonal	0.67	3.06			disease_causing	23.8	p.L293P	Passenger	SENGER/OT	damaging	Neutral	Tolerated
OVA_013_M1	METASTASIS	chr17	76046219	76046219	G	T	TNRC6C	exonic	ynonymou	359V,ENSG	319	253	572	0.442308	59	0	59	0	1	Clonal	0.67	3.06			disease_causing	23.9	p.G359V	Passenger	SENGER/OT	damaging	Neutral	Damaging
OVA_013_C1	PRIMARY	chr17	76046219	76046219	G	T	TNRC6C	exonic	ynonymou	359V,ENSG	286	310	596	0.520134	59	0	59	0	1	Clonal	0.72	3.1			disease_causing	23.9	p.G359V	Passenger	SENGER/OT	damaging	Neutral	Damaging
OVA_013_C2	PRIMARY	chr17	76046219	76046219	G	T	TNRC6C	exonic	ynonymou	359V,ENSG	258	327	585	0.558974	59	0	59	0	1	Clonal	0.87	3.05			disease_causing	23.9	p.G359V	Passenger	SENGER/OT	damaging	Neutral	Damaging
OVA_013_C3	PRIMARY	chr17	76046219	76046219	G	T	TNRC6C	exonic	ynonymou	359V,ENSG	297	330	627	0.526316	59	0	59	0	1	Clonal	0.91	3.01			disease_causing	23.9	p.G359V	Passenger	SENGER/OT	damaging	Neutral	Damaging
OVA_047_C3	Contralateral ovary	chr17	76120655	76120655	G	A	TMC6	exonic	nonymous S	281L,ENSG	184	68	252	0.269841	32	0	32	0	1	Clonal	0.6	2.94			polymorphism	1.952		Passenger			Neutral	Tolerated
OVA_047_C1	PRIMARY	chr17	76120655	76120655	G	A	TMC6	exonic	nonymous S	281L,ENSG	207	46	253	0.181818	32	0	32	0	0.79	Sub	0.6	2.91			polymorphism	1.952		Passenger			Neutral	Tolerated
OVA_047_C1	PRIMARY	chr17	76120676	76120676	G	T	TMC6	exonic	ynonymou	274K,ENSG	235	56	291	0.19244	36	0	36	0	0.83	Clonal	0.6	2.91			disease_causing	23.4	p.Q274K	Passenger	SENGER/OT	damaging	Deleterious	Damaging
OVA_047_C3	Contralateral ovary	chr17	76120676	76120676	G	T	TMC6	exonic	ynonymou	274K,ENSG	197	83	280	0.296429	36	0	36	0	1	Clonal	0.6	2.94			disease_causing	23.4	p.Q274K	Passenger	SENGER/OT	damaging	Deleterious	Damaging
OVA_047_C1	PRIMARY	chr15	76248242	76248242	T	C	NRG4	intronic	NA	NA	346	230	576	0.399306	60	0	60	0	0.93	Clonal	0.6	2.91			polymorphism	4.563						
OVA_047_C2	PRIMARY	chr15	76248242	76248242	T	C	NRG4	intronic	NA	NA	454	188	642	0.292835	60	0	60	0	0.92	Clonal	0.64	2.76			polymorphism	4.563						
OVA_047_C3	Contralateral ovary	chr15	76248242	76248242	T	C	NRG4	intronic	NA	NA	440	211	651	0.324117	60	0	60	0	1	Clonal	0.6	2.94			polymorphism	4.563						
OVA_013_M1	METASTASIS	chr11	76372308	76372308	G	A	LRRC32	exonic	ynonymou	110V,ENSG	230	49	279	0.175627	78	0	78	0	1	Clonal	0.67	3.06			polymorphism	14.09	p.A110V	Passenger	SENGER/OT	damaging	Neutral	Tolerated
OVA_013_C3	PRIMARY	chr11	76372308	76372308	G	A	LRRC32	exonic	ynonymou	110V,ENSG	324	83	407	0.203931	78	0	78	0	1	Clonal	0.91	3.01			polymorphism	14.09	p.A110V	Passenger	SENGER/OT	damaging	Neutral	Tolerated
OVA_003_M1	METASTASIS	chr11	76391244	76391244	T	C	GUCY2EP	RNA_intro	NA	NA	405	140	545	0.256881	86	0	86	0	0.91	Sub	0.57	1.84				0.234						
OVA_003_M2	METASTASIS	chr11	76391244	76391244	T	C	GUCY2EP	RNA_intro	NA	NA	425	130	555	0.234234	86	0	86	0	1	Sub	0.46	1.81				0.234						
OVA_003_M3	METASTASIS	chr11	76391244	76391244	T	C	GUCY2EP	RNA_intro	NA	NA	568	48	616	0.077922	86	0	86	0	0.82	Sub	0.34	2.67				0.234						
OVA_003_C1	PRIMARY	chr11	76391244	76391244	T	C	GUCY2EP	RNA_intro	NA	NA	325	256	581	0.44062	86	0	86	0	1	Sub	0.86	1.81				0.234						
OVA_003_C2	PRIMARY	chr11	76391244	76391244	T	C	GUCY2EP	RNA_intro	NA	NA	380	234	614	0.381107	86	0	86	0	1	Sub	0.7	3.61				0.234						
OVA_003_C3	PRIMARY	chr11	76391244	76391244	T	C	GUCY2EP	RNA_intro	NA	NA	422	245	667	0.367316	86	0	86	0	0.89	Sub	0.82	1.81				0.234						
OVA_378_M1	METASTASIS	chr17	76533455	76533455	C	T	DNAH17	exonic	ynonymou	929K,ENSG	246	146	392	0.372449	65	0	65	0	1	Clonal	0.72	2.57			disease_causing	21.9	p.E929K	Passenger	SENGER/OT		Deleterious	Tolerated
OVA_378_M2	METASTASIS	chr17	76533455	76533455	C	T	DNAH17	exonic	ynonymou	929K,ENSG	186	153	339	0.451327	65	0	65	0	1	Clonal	0.85	2.57			disease_causing	21.9	p.E929K	Passenger	SENGER/OT		Deleterious	Tolerated
OVA_378_M3	METASTASIS	chr17	76533455	76533455	C	T	DNAH17	exonic	ynonymou	929K,ENSG	222	134	356	0.376404	65	0	65	0	1	Clonal	0.67	2.57			disease_causing	21.9	p.E929K	Passenger	SENGER/OT		Deleterious	Tolerated
OVA_378_C1	PRIMARY	chr17	76533455	76533455	C	T	DNAH17	exonic	ynonymou	929K,ENSG	319	148	467	0.316916	65	0	65	0	1	Clonal	0.59	2.6			disease_causing	21.9	p.E929K	Passenger	SENGER/OT		Deleterious	Tolerated
OVA_378_C2	PRIMARY	chr17	76533455	76533455	C	T	DNAH17	exonic	ynonymou	929K,ENSG	258	130	388	0.335052	65	0	65	0	1	Clonal	0.56	2.59			disease_causing	21.9	p.E929K	Passenger	SENGER/OT		Deleterious	Tolerated
OVA_378_C3	PRIMARY	chr17	76533455	76533455	C	T	DNAH17	exonic	ynonymou	929K,ENSG	269	99	368	0.269022	65	0	65	0	1	Clonal	0.51	2.6			disease_causing	21.9	p.E929K	Passenger	SENGER/OT		Deleterious	Tolerated
OVA_378_C1	PRIMARY	chr16	76572141	76572141	A	T	CNTNAP4	exonic	ynonymou	I969F,ENSG	493	50	543	0.092081	48	0	48	0	0.31	Sub	0.59	2.6			disease_causing	22.4	p.I1045F	Passenger	SENGER/OT	damaging	Deleterious	Damaging
OVA_365_M1	METASTASIS	chr16	76574777	76574777	G	A	CNTNAP4	intronic	NA	NA	93	428	521	0.821497	44	0	44	0	1	Sub	0.85	1.87			disease_causing	8.882						
OVA_365_M2	METASTASIS	chr16	76574777	76574777	G	A	CNTNAP4	intronic	NA	NA	447	100	547	0.182815	44	0	44	0	0.72	Sub	0.4	1.81			disease_causing	8.882						
OVA_365_M3	METASTASIS	chr16	76574777	76574777	G	A	CNTNAP4	intronic	NA	NA	184	350	534	0.655431	44	0	44	0	0.95	Sub	0.82	1.89			disease_causing	8.882						
OVA_365_C2	PRIMARY	chr16	76574777	76574777	G	A	CNTNAP4	intronic	NA	NA	417	172	589	0.29202	44	0	44	0	0.95	Clonal	0.62	1.92			disease_causing	8.882						
OVA_047_M1	METASTASIS	chr17	76688532	76688532	A	G	CYTH1	exonic	nonymous S	000044686	292	376	668	0.562874	56	0	56	0	1	Clonal	0.47	2.01			disease_causing	1.7		Passenger			Neutral	Tolerated
OVA_047_M2	METASTASIS	chr17	76688532	76688532	A	G	CYTH1	exonic	nonymous S	000044686	302	368	670	0.549254	56	0	56	0	1	Clonal	0.44	2.01			disease_causing	1.7		Passenger			Neutral	Tolerated
OVA_047_M3	METASTASIS	chr17	76688532	76688532	A	G	CYTH1	exonic	nonymous S	000044686	161	252	413	0.610169	56	0	56	0	1	Clonal	0.6	3.02			disease_causing	1.7		Passenger			Neutral	Tolerated
OVA_047_C1	PRIMARY	chr17	76688532	76688532	A	G	CYTH1	exonic	nonymous S	000044686	336	319	655	0.487023	56	0	56	0	1	Clonal	0.6	2.91			disease_causing	1.7		Passenger			Neutral	Tolerated
OVA_047_C2	PRIMARY	chr17	76688532	76688532	A	G	CYTH1	exonic	nonymous S	000044686	371	277	648	0.427469	56	0	56	0	1	Clonal	0.64	2.76			disease_causing	1.7		Passenger			Neutral	Tolerated
OVA_047_C3	Contralateral ovary	chr17	76688532	76688532	A	G	CYTH1	exonic	nonymous S	000044686	340	280	620	0.451613	56	0	56	0	1	Clonal	0.6	2.94			disease_causing	1.7		Passenger			Neutral	Tolerated
OVA_013_M1	METASTASIS	chr1	76775583	76775583	G	A	T6GALNAC	intronic	NA	NA	479	94	573	0.164049	21	0	21	0	0.82	Sub	0.67	3.06			polymorphism	7.105						
OVA_013_C1	PRIMARY	chr1	76775583	76775583	G	A	T6GALNAC	intronic	NA	NA	466	124	590	0.210169	21	0	21	0	1	Sub	0.72	3.1			polymorphism	7.105						
OVA_013_C2	PRIMARY	chr1	76775583	76775583	G	A	T6GALNAC	intronic	NA	NA	456	109	565	0.19292	21	0	21	0	0.83	Sub	0.87	3.05			polymorphism	7.105						
OVA_013_C3	PRIMARY	chr1	76775583	76775583	G	A	T6GALNAC	intronic	NA	NA	491	127	618	0.205502	21	0	21	0	0.86	Sub	0.91	3.01			polymorphism	7.105						
OVA_047_C2	PRIMARY	chr9	77377255	77377255	C	A	TRPM6	exonic	ynonymou	000376864	602	111	713	0.15568	70	0	70	0	0.64	Sub	0.64	2.76			polymorphism	12.94	p.K1444N	Passenger	SENGER/OT	benign	Neutral	Tolerated
OVA_365_M1	METASTASIS	chr13	77459768	77459768	G	A	KCTD12	exonic	nonymous S	172A,ENSG	5	5	10	0.5	26	0	26	0	1	Sub	0.85	1.87			disease_causing	16.41		Passenger			Neutral	Tolerated
OVA_365_M2	METASTASIS	chr13	77459768	77459768	G	A	KCTD12	exonic	nonymous S	172A,ENSG	4	3	7	0.428571	26	0	26	0	1	Sub	0.4	1.81			disease_causing	16.41		Passenger			Neutral	Tolerated
OVA_365_M3	METASTASIS	chr13	77459768	77459768	G	A	KCTD12	exonic	nonymous S	172A,ENSG	11	3	14	0.214286	26	0	26	0	0.52	Sub	0.82	1.89			disease_causing	16.41		Passenger			Neutral	Tolerated
OVA_013_M1	METASTASIS	chr13	77532116	77532116	C	T	IRG1	exonic	ynonymou	481F,ENSG	334	144	478	0.301255	15	0	15	0	0.9	Clonal	0.67	3.06			polymorphism	14.46	p.S481F	Passenger	no weights		Neutral	Damaging
OVA_013_C1	PRIMARY	chr13	77532116	77532116	C	T	IRG1	exonic	ynonymou	481F,ENSG	319	213	532	0.400376	15	0	15	0	1	Sub	0.72	3.1			polymorphism	14.46	p.S481F	Passenger	no weights		Neutral	Damaging
OVA_013_C2	PRIMARY	chr13	77532116	77532116	C	T	IRG1	exonic	ynonymou	481F,ENSG	294	173	467	0.37045	15	0	15	0	0.85	Sub	0.87	3.05			polymorphism	14.46	p.S481F	Passenger	no weights		Neutral	Damaging
OVA_013_C3	PRIMARY	chr13	77532116	77532116	C	T	IRG1	exonic	ynonymou	481F,ENSG	314	226	540	0.418519	15	0	15	0	0.92	Sub	0.91	3.01			polymorphism	14.46	p.S481F	Passenger	no weights		Neutral	Damaging
OVA_003_C1	PRIMARY	chr13	77692585	77692585	T	C	MYCBP2	exonic	ynonymou	000036008	524	6	530	0.011321	71	0	71	0	0.02	Sub	0.86	1.81			disease_causing	19.58				benign	Neutral	Tolerated
OVA_003_C2	PRIMARY	chr13	77692585	77692585	T	C	MYCBP2	exonic	ynonymou	000036008	463	100	563	0.17762	71	0	71	0	0.51	Sub	0.7	3.61			disease_causing	19.58				benign	Neutral	Tolerated
OVA_048_C3	PRIMARY	chr2	77749348	77749348	A	G	LRRTM4	UTR5	NA	NA	244	188	432	0.435185	17	0	17	0	1	Clonal	0.74	1.8			disease_causing	16.72						
OVA_378_M1	METASTASIS	chr17	77757929	77757929	C	A	CBX2	exonic	nonymous S	000031094	36	19	55	0.345455	117	0	117	0	0.96	Clonal	0.72	2.57			disease_causing	9.267		Passenger			Neutral	Tolerated
OVA_378_M2	METASTASIS	chr17	77757929	77757929	C	A	CBX2	exonic	nonymous S	000031094	21	22	43	0.511628	117	0	117	0	1	Clonal	0.85	2.57			disease_causing	9.267		Passenger			Neutral	Tolerated
OVA_378_M3	METASTASIS	chr17	77757929	77757929	C	A	CBX2	exonic	nonymous S	000031094	61	28	89	0.314607	117	0	117	0	0.94	Clonal	0.67	2.57			disease_causing	9.267		Passenger			Neutral	Tolerated
OVA_378_C1	PRIMARY	chr17	77757929	77757929	C	A	CBX2	exonic	nonymous S	000031094	47	29	76	0.381579	117	0	117	0	1	Sub	0.59	2.6			disease_causing	9.267		Passenger			Neutral	Tolerated
OVA_378_C2	PRIMARY	chr17	77757929	77757929	C	A	CBX2	exonic	nonymous S	000031094	31	17	48	0.354167	117	0	117	0	1	Sub	0.56	2.59			disease_causing	9.267		Passenger			Neutral	Tolerated
OVA_378_C3	PRIMARY	chr17	77757929	77757929	C	A	CBX2	exonic	nonymous S	000031094	36	7	43	0.162791	117	0	117	0	0.64	Sub	0.51	2.6			disease_causing	9.267		Passenger			Neutral	Tolerated
OVA_048_C1	PRIMARY	chr18	77896247	77896247	A	T	ADNP2	exonic	ynonymou	000026219	37	58	95	0.610526	49	0	49	0	1	Clonal	0.67	1.82			polymorphism	10.96	p.H984L	Passenger	no weights	benign	Neutral	Tolerated
OVA_048_C3	PRIMARY	chr18	77896247	77896247	A	T	ADNP2	exonic	ynonymou	000026219	49	55	104	0.528846	49	0	49	0	0.9	Clonal	0.74	1.8			polymorphism	10.96	p.H984L	Passenger	no weights	benign	Neutral	Tolerated
OVA_003_M1	METASTASIS	chr17	78155363	78155363	C	T	CARD14	exonic	nonymous S	.Y42Y,ENSG	148	71	219	0.324201	31	0	31	0	1	Sub	0.57	1.84			disease_causing	11.03		Passenger			Neutral	Tolerated
OVA_003_M2	METASTASIS	chr17	78155363	78155363	C	T	CARD14	exonic	nonymous S	.Y42Y,ENSG	182	59	241	0.244813	31	0	31	0	1	Sub	0.46	1.81			disease_causing	11.03		Passenger			Neutral	Tolerated
OVA_003_M3	METASTASIS	chr17	78155363	78155363	C	T	CARD14	exonic	nonymous S	.Y42Y,ENSG	319	24	343	0.069971	31	0	31	0	0.74	Sub	0.34	2.67			disease_causing	11.03		Passenger			Neutral	Tolerated
OVA_003_C1	PRIMARY	chr17	78155363	78155363	C	T	CARD14	exonic	nonymous S	.Y42Y,ENSG	249	150	399	0.37594	31	0	31	0	1	Clonal	0.86	1.81			disease_causing	11.03		Passenger			Neutral	Tolerated
OVA_003_C2	PRIMARY	chr17	78155363	78155363	C	T	CARD14	exonic	nonymous S	.Y42Y,ENSG	254	180	434	0.414747	31	0	31	0	1	Clonal	0.7	3.61			disease_causing	11.03		Passenger			Neutral	Tolerated
OVA_003_C3	PRIMARY	chr17	78155363	78155363	C	T	CARD14	exonic	nonymous S	.Y42Y,ENSG	276	186	462	0.402597	31	0	31	0	0.98	Clonal	0.82	1.81			disease_causing	11.03		Passenger			Neutral	Tolerated
OVA_013_M1	METASTASIS	chr17	78401505	78401505	G	T	ENDOV	exonic	ynonymou	000005193	237	86	323	0.266254	91	1	92	0.01087	1	Clonal	0.67	3.06			polymorphism	1.2					Neutral	Tolerated
OVA_047_M1	METASTASIS	chr3	78696782	78696782	T	C	ROBO1	exonic	ynonymou	907A,ENSG	557	138	695	0.198561	410	0	410	0	1	Sub	0.47	2.01			disease_causing	19.93	p.T946A	Passenger	SENGER/OT	benign	Neutral	Tolerated
OVA_047_M2	METASTASIS	chr3	78696782	78696782	T	C	ROBO1	exonic	ynonymou	907A,ENSG	596	102	698	0.146132	410	0	410	0	0.81	Sub	0.44	2.01			disease_causing	19.93	p.T946A	Passenger	SENGER/OT	benign	Neutral	Tolerated
OVA_047_M3	METASTASIS	chr3	78696782	78696782	T	C	ROBO1	exonic	ynonymou	907A,ENSG	406	97	503	0.192843	410	0	410	0	1	Sub	0.6	3.02			disease_causing	19.93	p.T946A	Passenger	SENGER/OT	benign	Neutral	Tolerated
OVA_365_M1	METASTASIS	chr17	79278647	79278647	C	A	LINC00482	cRNA_exon	NA	NA	3	79	82	0.963415	71	0	71	0	1	Clonal	0.85	1.87				2.483				damaging	Deleterious	NA

Sample	Type	Chr	Start	End	Ref	Alt	Gene	unc.ensGen	icFunc.ens	hange.ens	t_ref	t_alt	t_total	t_VAF	n_ref	n_alt	n_total	hormal_VA	ncer_cell_f	on-based C	purity	ploidy	Cancer Gene	Driver Gene	Mutation Taster	ADD(PHRE	otein_chan	CHASM	ATHMM_Pr	OLYPHEN-	REDICTION	ICTION (cutoff=0.05)
OVA_365_M2	METASTASIS	chr17	79278647	79278647	C	A	LINC00482	cRNA_exon	NA	NA	16	23	39	0.589744	71	0	71	0	1	Clonal	0.4	1.81				2.483				damaging	Deleterious	NA
OVA_365_M3	METASTASIS	chr17	79278647	79278647	C	A	LINC00482	cRNA_exon	NA	NA	5	56	61	0.918033	71	0	71	0	1	Clonal	0.82	1.89				2.483				damaging	Deleterious	NA
OVA_365_C1	Contralateral ovary	chr17	79278647	79278647	C	A	LINC00482	cRNA_exon	NA	NA	133	2	135	0.014815	71	0	71	0	0.06	Sub	0.42	1.83				2.483				damaging	Deleterious	NA
OVA_365_C2	PRIMARY	chr17	79278647	79278647	C	A	LINC00482	cRNA_exon	NA	NA	28	51	79	0.64557	71	0	71	0	1	Sub	0.62	1.92				2.483				damaging	Deleterious	NA
OVA_003_M1	METASTASIS	chr2	79312388	79312388	T	A	REG1B	exonic	ynonymou	N132Y,ENSG	389	136	525	0.259048	32	0	32	0	0.92	Sub	0.57	1.84			polymorphism	12.24	p.E152V	Passenger	SENGER/OT	benign	Neutral	Tolerated
OVA_003_M2	METASTASIS	chr2	79312388	79312388	T	A	REG1B	exonic	ynonymou	N132Y,ENSG	441	107	548	0.195255	32	0	32	0	0.85	Sub	0.46	1.81			polymorphism	12.24	p.E152V	Passenger	SENGER/OT	benign	Neutral	Tolerated
OVA_003_M3	METASTASIS	chr2	79312388	79312388	T	A	REG1B	exonic	ynonymou	N132Y,ENSG	585	28	613	0.045677	32	0	32	0	0.48	Sub	0.34	2.67			polymorphism	12.24	p.E152V	Passenger	SENGER/OT	benign	Neutral	Tolerated
OVA_003_C1	PRIMARY	chr2	79312388	79312388	T	A	REG1B	exonic	ynonymou	N132Y,ENSG	326	269	595	0.452101	32	0	32	0	1	Clonal	0.86	1.81			polymorphism	12.24	p.E152V	Passenger	SENGER/OT	benign	Neutral	Tolerated
OVA_003_C2	PRIMARY	chr2	79312388	79312388	T	A	REG1B	exonic	ynonymou	N132Y,ENSG	359	253	612	0.413399	32	0	32	0	1	Clonal	0.7	3.61			polymorphism	12.24	p.E152V	Passenger	SENGER/OT	benign	Neutral	Tolerated
OVA_003_C3	PRIMARY	chr2	79312388	79312388	T	A	REG1B	exonic	ynonymou	N132Y,ENSG	371	255	626	0.407348	32	0	32	0	0.99	Clonal	0.82	1.81			polymorphism	12.24	p.E152V	Passenger	SENGER/OT	benign	Neutral	Tolerated
OVA_047_M1	METASTASIS	chr17	79911090	79911090	C	A	NOTUM	exonic	ynonymou	000040967	218	37	255	0.145098	32	0	32	0	0.9	Clonal	0.47	2.01			disease_causing	31	p.C413F	Passenger	no weights	damaging	Deleterious	Damaging
OVA_047_M2	METASTASIS	chr17	79911090	79911090	C	A	NOTUM	exonic	ynonymou	000040967	274	54	328	0.164634	32	0	32	0	1	Clonal	0.44	2.01			disease_causing	31	p.C413F	Passenger	no weights	damaging	Deleterious	Damaging
OVA_047_M3	METASTASIS	chr17	79911090	79911090	C	A	NOTUM	exonic	ynonymou	000040967	110	30	140	0.214286	32	0	32	0	1	Clonal	0.6	3.02			disease_causing	31	p.C413F	Passenger	no weights	damaging	Deleterious	Damaging
OVA_013_M1	METASTASIS	chr12	80083686	80083686	T	C	PAWR	exonic	onymous S	000032882	9	5	14	0.357143	55	0	55	0	1	Clonal	0.67	3.06			disease_causing	10.19		Passenger			Neutral	Tolerated
OVA_013_C1	PRIMARY	chr12	80083686	80083686	T	C	PAWR	exonic	onymous S	000032882	16	4	20	0.357143	55	0	55	0	0.75	Clonal	0.72	3.1			disease_causing	10.19		Passenger			Neutral	Tolerated
OVA_013_C2	PRIMARY	chr12	80083686	80083686	T	C	PAWR	exonic	onymous S	000032882	14	5	19	0.357143	55	0	55	0	0.87	Clonal	0.87	3.05			disease_causing	10.19		Passenger			Neutral	Tolerated
OVA_047_M1	METASTASIS	chr4	80246890	80246890	C	T	NAA11	exonic	ynonymou	000002867	335	313	648	0.483025	81	1	82	0.012195	1	Clonal	0.47	2.01			disease_causing	19.31	p.E48K	Passenger	SENGER/OT	damaging	Deleterious	Tolerated
OVA_047_M2	METASTASIS	chr4	80246890	80246890	C	T	NAA11	exonic	ynonymou	000002867	350	293	643	0.455677	81	1	82	0.012195	1	Clonal	0.44	2.01			disease_causing	19.31	p.E48K	Passenger	SENGER/OT	damaging	Deleterious	Tolerated
OVA_047_M3	METASTASIS	chr4	80246890	80246890	C	T	NAA11	exonic	ynonymou	000002867	185	147	332	0.442771	81	1	82	0.012195	1	Clonal	0.6	3.02			disease_causing	19.31	p.E48K	Passenger	SENGER/OT	damaging	Deleterious	Tolerated
OVA_047_C1	PRIMARY	chr4	80246890	80246890	C	T	NAA11	exonic	ynonymou	000002867	400	193	593	0.325464	81	1	82	0.012195	1	Clonal	0.6	2.91			disease_causing	19.31	p.E48K	Passenger	SENGER/OT	damaging	Deleterious	Tolerated
OVA_047_C2	PRIMARY	chr4	80246890	80246890	C	T	NAA11	exonic	ynonymou	000002867	417	182	599	0.30384	81	1	82	0.012195	0.95	Clonal	0.64	2.76			disease_causing	19.31	p.E48K	Passenger	SENGER/OT	damaging	Deleterious	Tolerated
OVA_047_C3	Contralateral ovary	chr4	80246890	80246890	C	T	NAA11	exonic	ynonymou	000002867	394	194	588	0.329932	81	1	82	0.012195	1	Clonal	0.6	2.94			disease_causing	19.31	p.E48K	Passenger	SENGER/OT	damaging	Deleterious	Tolerated
OVA_003_M1	METASTASIS	chr5	80368995	80368995	C	A	RASGRF2	intronic	NA	NA	426	183	609	0.300493	45	0	45	0	1	Sub	0.57	1.84			polymorphism	2.473						
OVA_003_M2	METASTASIS	chr5	80368995	80368995	C	A	RASGRF2	intronic	NA	NA	474	143	617	0.231767	45	0	45	0	1	Sub	0.46	1.81			polymorphism	2.473						
OVA_003_M3	METASTASIS	chr5	80368995	80368995	C	A	RASGRF2	intronic	NA	NA	616	33	649	0.050847	45	0	45	0	0.54	Sub	0.34	2.67			polymorphism	2.473						
OVA_003_C1	PRIMARY	chr5	80368995	80368995	C	A	RASGRF2	intronic	NA	NA	388	259	647	0.400309	45	0	45	0	0.93	Clonal	0.86	1.81			polymorphism	2.473						
OVA_003_C2	PRIMARY	chr5	80368995	80368995	C	A	RASGRF2	intronic	NA	NA	391	253	644	0.392857	45	0	45	0	1	Clonal	0.7	3.61			polymorphism	2.473						
OVA_003_C3	PRIMARY	chr5	80368995	80368995	C	A	RASGRF2	intronic	NA	NA	384	284	668	0.42515	45	0	45	0	1	Clonal	0.82	1.81			polymorphism	2.473						
OVA_003_M1	METASTASIS	chr7	80535069	80535069	G	A	SEMA3C	intronic	NA	NA	428	162	590	0.274576	49	0	49	0	0.97	Sub	0.57	1.84			polymorphism	2.704						
OVA_003_M2	METASTASIS	chr7	80535069	80535069	G	A	SEMA3C	intronic	NA	NA	470	138	608	0.226974	49	0	49	0	0.99	Sub	0.46	1.81			polymorphism	2.704						
OVA_003_M3	METASTASIS	chr7	80535069	80535069	G	A	SEMA3C	intronic	NA	NA	592	50	642	0.077882	49	0	49	0	0.82	Sub	0.34	2.67			polymorphism	2.704						
OVA_003_C1	PRIMARY	chr7	80535069	80535069	G	A	SEMA3C	intronic	NA	NA	336	273	609	0.448276	49	0	49	0	1	Clonal	0.86	1.81			polymorphism	2.704						
OVA_003_C2	PRIMARY	chr7	80535069	80535069	G	A	SEMA3C	intronic	NA	NA	321	277	598	0.463211	49	0	49	0	1	Clonal	0.7	3.61			polymorphism	2.704						
OVA_003_C3	PRIMARY	chr7	80535069	80535069	G	A	SEMA3C	intronic	NA	NA	371	272	643	0.423017	49	0	49	0	1	Clonal	0.82	1.81			polymorphism	2.704						
OVA_003_M1	METASTASIS	chr15	80767480	80767480	G	C	ARNT2	exonic	ynonymou	000052777	314	97	411	0.23601	58	0	58	0	0.84	Sub	0.57	1.84			disease_causing	28.7	p.G180R	Passenger	SENGER/OT	damaging	Deleterious	Damaging
OVA_003_M2	METASTASIS	chr15	80767480	80767480	G	C	ARNT2	exonic	ynonymou	000052777	420	82	502	0.163347	58	0	58	0	0.71	Sub	0.46	1.81			disease_causing	28.7	p.G180R	Passenger	SENGER/OT	damaging	Deleterious	Damaging
OVA_003_M3	METASTASIS	chr15	80767480	80767480	G	C	ARNT2	exonic	ynonymou	000052777	526	24	550	0.043636	58	0	58	0	0.42	Sub	0.34	2.67			disease_causing	28.7	p.G180R	Passenger	SENGER/OT	damaging	Deleterious	Damaging
OVA_003_C1	PRIMARY	chr15	80767480	80767480	G	C	ARNT2	exonic	ynonymou	000052777	300	177	477	0.371069	58	0	58	0	0.86	Sub	0.86	1.81			disease_causing	28.7	p.G180R	Passenger	SENGER/OT	damaging	Deleterious	Damaging
OVA_003_C2	PRIMARY	chr15	80767480	80767480	G	C	ARNT2	exonic	ynonymou	000052777	328	139	467	0.297645	58	0	58	0	1	Sub	0.7	3.61			disease_causing	28.7	p.G180R	Passenger	SENGER/OT	damaging	Deleterious	Damaging
OVA_003_C3	PRIMARY	chr15	80767480	80767480	G	C	ARNT2	exonic	ynonymou	000052777	347	233	580	0.401724	58	0	58	0	0.98	Sub	0.82	1.81			disease_causing	28.7	p.G180R	Passenger	SENGER/OT	damaging	Deleterious	Damaging
OVA_365_M1	METASTASIS	chr13	80911119	80911119	G	-	SPRY2	exonic	eshift dele	000037710	1246	923	2169	0.425542	82	0	82		1	Clonal	0.85	1.87	Yes	Yes			p.P241Hfs	Passenger			NA	NA
OVA_365_M2	METASTASIS	chr13	80911119	80911119	G	-	SPRY2	exonic	eshift dele	000037710	1370	424	1794	0.236343	82	0	82		1	Clonal	0.4	1.81	Yes	Yes			p.P241Hfs	Passenger			NA	NA
OVA_365_M3	METASTASIS	chr13	80911119	80911119	G	-	SPRY2	exonic	eshift dele	000037710	1180	894	2074	0.431051	82	0	82		1	Clonal	0.82	1.89	Yes	Yes			p.P241Hfs	Passenger			NA	NA
OVA_365_C1	Contralateral ovary	chr13	80911119	80911119	G	-	SPRY2	exonic	eshift dele	000037710	1262	453	1715	0.26414	82	0	82		1	Clonal	0.42	1.83	Yes	Yes			p.P241Hfs	Passenger			NA	NA
OVA_365_C2	PRIMARY	chr13	80911119	80911119	G	-	SPRY2	exonic	eshift dele	000037710	1223	660	1883	0.350505	82	0	82		1	Clonal	0.62	1.92	Yes	Yes			p.P241Hfs	Passenger			NA	NA
OVA_013_C2	PRIMARY	chr7	82545359	82545359	A	T	PCLO	exonic	onymous S	00333891:	619	59	678	0.087021	55	0	55	0	0.37	Sub	0.87	3.05			disease_causing	8.35		Passenger			Neutral	Tolerated
OVA_048_M1	METASTASIS	chrX	83442803	83442803	G	A	RPS6KA6	intronic	NA	NA	142	9	151	0.059603	45	1	46	0.021739	0.39	Sub	0.56	3.94			polymorphism	7.772						
OVA_013_M1	METASTASIS	chr6	84302770	84302770	A	T	SNAP91	intronic	NA	NA	328	203	531	0.382298	24	0	24	0	1	Clonal	0.67	3.06			disease_causing	14.86						
OVA_013_C1	PRIMARY	chr6	84302770	84302770	A	T	SNAP91	intronic	NA	NA	348	236	584	0.40411	24	0	24	0	1	Clonal	0.72	3.1			disease_causing	14.86						
OVA_013_C2	PRIMARY	chr6	84302770	84302770	A	T	SNAP91	intronic	NA	NA	294	259	553	0.468354	24	0	24	0	1	Clonal	0.87	3.05			disease_causing	14.86						
OVA_013_C3	PRIMARY	chr6	84302770	84302770	A	T	SNAP91	intronic	NA	NA	347	227	574	0.39547	24	0	24	0	1	Clonal	0.91	3.01			disease_causing	14.86						
OVA_013_M1	METASTASIS	chrX	84347445	84347445	G	A	.POOL;SATL	exonic	onymous S	000039540	424	186	610	0.304918	47	0	47	0	1	Clonal	0.67	3.06			disease_causing	10.87		Passenger			Neutral	Tolerated
OVA_013_C1	PRIMARY	chrX	84347445	84347445	G	A	.POOL;SATL	exonic	onymous S	000039540	425	179	604	0.296358	47	0	47	0	1	Sub	0.72	3.1			disease_causing	10.87		Passenger			Neutral	Tolerated
OVA_013_C2	PRIMARY	chrX	84347445	84347445	G	A	.POOL;SATL	exonic	onymous S	000039540	444	161	605	0.266116	47	0	47	0	0.88	Sub	0.87	3.05			disease_causing	10.87		Passenger			Neutral	Tolerated
OVA_013_C3	PRIMARY	chrX	84347445	84347445	G	A	.POOL;SATL	exonic	onymous S	000039540	460	185	645	0.286822	47	0	47	0	0.91	Sub	0.91	3.01			disease_causing	10.87		Passenger			Neutral	Tolerated
OVA_048_M1	METASTASIS	chr16	84600434	84600434	T	C	COTL1	UTR3	NA	NA	88	24	112	0.214286	36	0	36	0	1	Clonal	0.56	3.94			polymorphism	5.51						
OVA_048_M2	METASTASIS	chr16	84600434	84600434	T	C	COTL1	UTR3	NA	NA	108	16	124	0.129032	36	0	36	0	0.9	Clonal	0.4	3.87			polymorphism	5.51						
OVA_013_C3	PRIMARY	chr1	84955266	84955266	C	T	RPF1	intronic	NA	NA	635	45	680	0.066176	108	0	108	0	0.28	Sub	0.91	3.01			polymorphism	5.548						
OVA_048_M1	METASTASIS	chr1	84991595	84991595	C	G	SPATA1	cRNA_exon	NA	NA	446	124	570	0.217544	37	0	37	0	1	Clonal	0.56	3.94				10.27				benign		
OVA_048_M2	METASTASIS	chr1	84991595	84991595	C	G	SPATA1	cRNA_exon	NA	NA	499	75	574	0.130662	37	0	37	0	0.91	Clonal	0.4	3.87				10.27				benign		
OVA_378_C1	PRIMARY	chr8	85618208	85618208	T	A	RALYL	UTR5	NA	NA	500	98	598	0.16388	33	0	33	0	0.89	Clonal	0.59	2.6			disease_causing	20.6						
OVA_378_C2	PRIMARY	chr8	85618208	85618208	T	A	RALYL	UTR5	NA	NA	456	107	563	0.190053	33	0	33	0	1	Clonal	0.56	2.59			disease_causing	20.6						
OVA_378_C3	PRIMARY	chr8	85618208	85618208	T	A	RALYL	UTR5	NA	NA	455	91	546	0.166667	33	0	33	0	1	Clonal	0.51	2.6			disease_causing	20.6						
OVA_047_M1	METASTASIS	chr3	85851368	85851368	T	C	CADM2-AS:	RNA_intro	NA	NA	436	111	547	0.202925	19	0	19	0	1	Clonal	0.47	2.01			disease_causing	17.46						
OVA_047_M2	METASTASIS	chr3	85851368	85851368	T	C	CADM2-AS:	RNA_intro	NA	NA	415	122	537	0.227188	19	0	19	0	1	Clonal	0.44	2.01			disease_causing	17.46						
OVA_047_M3	METASTASIS	chr3	85851368	85851368	T	C	CADM2-AS:	RNA_intro	NA	NA	155	36	191	0.188482	19	0	19	0	1	Clonal	0.6	3.02			disease_causing	17.46						
OVA_047_C3	Contralateral ovary	chr10	85944549	85944549	C	T	C10orf99	UTR3	NA	NA	242	81	323	0.250774	29	0	29	0	0.84	Clonal	0.6	2.94			polymorphism	1.939						
OVA_047_M1	METASTASIS	chr11	86519121	86519121	C	T	PRSS23	exonic	ynonymou	000005275	572	143	715	0.2	110	0	110	0	1	Clonal	0.47	2.01			disease_causing	24.8	p.L146F	Passenger	SENGER/OT	damaging	Neutral	Tolerated
OVA_047_M2	METASTASIS	chr11	86519121	86519121	C	T	PRSS23	exonic	ynonymou	000005275	599	123	722	0.17036	110	0	110	0	0.94	Clonal	0.44	2.01			disease_causing	24.8	p.L146F	Passenger	SENGER/OT	damaging	Neutral	Tolerated
OVA_047_M3	METASTASIS	chr11	86519121	86519121	C	T	PRSS23	exonic	ynonymou	000005275	446	93	539	0.172542	110	0	110	0	0.92	Clonal	0.6	3.02			disease_causing	24.8	p.L146F	Passenger	SENGER/OT	damaging	Neutral	Tolerated
OVA_047_M1	METASTASIS	chr7	86781654	86781654	T	A	035377;DM	cRNA_exon	NA	NA	395	170	565	0.300885	187	1	188	0.005319	0.97	Clonal	0.47	2.01				6.335						
OVA_047_M2	METASTASIS	chr7	86781654	86781654	T	A	035377;DM	cRNA_exon	NA	NA	430	169	599	0.282137	187	1	188	0.005319	0.99	Clonal	0.44	2.01				6.335						
OVA_047_M3	METASTASIS	chr7	86781654	86781654	T	A	035377;DM	cRNA_exon	NA	NA	137	54	191	0.282723	187	1	188	0.005319	0.94	Clonal	0.6	3.02				6.335						
OVA_378_M1	METASTASIS	chr4	86916496	86916496	C	G	ARHGAP24	exonic	onymous S	S468S,ENSG	123	427	550	0.776364	76	0	76	0	1	Clonal	0.72	2.57			disease_causing	18.66		Passenger			Neutral	Tolerated
OVA_378_M2	METASTASIS	chr4	86916496	86916496	C	G	ARHGAP24	exonic	onymous S	S468S,ENSG	61	488	549	0.888889	76	0	76	0	1	Clonal	0.85	2.57			disease_causing	18.66		Passenger			Neutral	Tolerated
OVA_378_M3	METASTASIS	chr4	86916496	86916496	C	G	ARHGAP24	exonic	onymous S	S468S,ENSG	161	424	585	0.724786	76	0	76	0	1	Clonal	0.67	2.57			disease_causing	18.66		Passenger			Neutral	Tolerated
OVA_378_C1	PRIMARY	chr4	86916496	86916496	C	G	ARHGAP24	exonic	onymous S	S468S,ENSG	211	381	592	0.643581	76	0	76	0	1	Clonal	0.59	2.6			disease_causing	18.66		Passenger			Neutral	Tolerated
OVA_378_C2	PRIMARY	chr4	86916496	86916496	C	G	ARHGAP24	exonic	onymous S	S468S,ENSG	264	298	562	0.530249	76	0	76	0	1	Clonal	0.56	2.59			disease_causing	18.66		Passenger			Neutral	Tolerated
OVA_378_C3	PRIMARY	chr4	86916496	86916496	C	G	ARHGAP24	exonic	onymous S	S468S,ENSG	295	267	562	0.475089	76	0	76	0	1	Clonal	0.51	2.6			disease_causing	18.66		Passenger			Neutral	Tolerated
OVA_047_C1	PRIMARY	chrX	86924357	86924357	A	G	KLHL4	exonic	ynonymou	000373114	576	97	673	0.144131	42	0	42	0	0.77	Sub	0.6	2.91			polymorphism	15.78	p.Y709C	Passenger	SENGER/OT	damaging	Neutral	Damaging
OVA_047_C2	PRIMARY	chrX	86924357	86924357	A	G	KLHL4	exonic	ynonymou	000373114	549	107	656	0.16311	42	0	42	0	0.67	Sub	0.64	2.76			polymorphism	15.78	p.Y709C	Passenger	SENGER/OT	damaging	Neutral	Damaging
OVA_047_C3	Contralateral ovary	chrX	86924357	86924357	A	G	KLHL4	exonic	ynonymou	000373114	548	105	653	0.160796	42	0	42	0	0.7	Sub	0.6	2.94			polymorphism	15.78	p.Y709C	Passenger	SENGER/OT	damaging	Neutral	Damaging
OVA_047_M1	METASTASIS	chr9	87563385	87563385	G	A	NTRK2	exonic	onymous S	591K,ENSG	581	138	719	0.191933	97	0	97	0	1	Clonal	0.47	2.01			disease_causing	10.14		Passenger			Neutral	Tolerated
OVA_047_M2	METASTASIS	chr9	87563385	87563385	G	A	NTRK2	exonic	onymous S	591K,ENSG	576	136	712	0.191011	97	0	97	0	1	Clonal	0.44	2.01			disease_causing	10.14		Passenger			Neutral	Tolerated
OVA_047_M3	METASTASIS	chr9	87563385	87563385	G	A	NTRK2	exonic	onymous S	591K,ENSG	487	124	611	0.202946	97	0	97	0	1	Clonal	0.6	3.02			disease_causing	10.14		Passenger			Neutral	Tolerated
OVA_003_M1	METASTASIS	chr9	87570458	87570458	C	A	NTRK2	intronic	NA	NA	265	153	418	0.366029	71	0	71	0	1	Clonal	0.57	1.84			polymorphism	1.331				benign		
OVA_003_M2	METASTASIS	chr9	87570458	87570458	C	A	NTRK2	intronic	NA	NA	315	146	461	0.316703	71	0	71	0	1	Clonal	0.46	1.81			polymorphism	1.331				benign		
OVA_003_M3	METASTASIS	chr9	87570458	87570458	C	A	NTRK2	intronic	NA	NA	429	52	481	0.108108	71	0	71	0	1	Clonal	0.34	2.67			polymorphism	1.331				benign		
OVA_003_C1	PRIMARY	chr9	87570458	87570458	C	A	NTRK2	intronic	NA	NA	255	255	510	0.5	71	0	71	0	1	Clonal	0.86	1.81			polymorphism	1.331				benign		
OVA_003_C2	PRIMARY	chr9	87570458	87570458	C	A	NTRK2	intronic	NA	NA	266	280	546	0.512821	71	0	71	0	1	Clonal	0.7	3.61			polymorphism	1.331				benign		
OVA_003_C3	PRIMARY	chr9	87570458	87570458	C	A	NTRK2	intronic	NA	NA	309	232	541	0.428835	71	0	71	0	1	Clonal	0.82	1.81			polymorphism	1.331				benign		
OVA_013_M1	METASTASIS	chr11	88258569	88258569	G	T	GRM5	exonic	stopgain	000039329	504	126	630	0.2	31	0	31	0	1	Clonal	0.67	3.06			disease_causing_automat	44	p.Y878X	Passenger			NA	NA
OVA_013_C1	PRIMARY	chr11	88258569	88258569	G	T	GRM5	exonic	stopgain	000039329	547	121	668	0.181138	31	0	31	0	1	Clonal	0.72	3.1			disease_causing_automat	44	p.Y878X	Passenger			NA	NA
OVA_013_C2	PRIMARY	chr11	88258569	88258569	G	T	GRM5	exonic	stopgain	000039329	521	119	640	0.185938	31	0	31	0	0.99	Clonal	0.87	3.05			disease_causing_automat	44	p.Y878X	Passenger			NA	NA
OVA_013_C3	PRIMARY	chr11	88258569	88258569	G	T	GRM5	exonic	stopgain	000039329	553	131	684	0.19152	31	0	31	0	0.99	Clonal	0.91	3.01			disease_causing_automat	44	p.Y878X	Passenger			NA	NA
OVA_003_M1	METASTASIS	chr2	88382213	88382213	T	C	SMYD1	intronic	NA	NA	363	131	494	0.265182	49	0	49	0	0.94	Sub	0.57	1.84			polymorphism	3.581						
OVA_003_M2	METASTASIS	chr2	88382213	88382213	T	C	SMYD1	intronic	NA	NA	423	121	544	0.222426	49	0	49	0	0.97	Sub	0.46	1.81			polymorphism	3.581						
OVA_003_M3	METASTASIS	chr2	88382213	88382213	T	C	SMYD1	intronic	NA	NA	535	37	572	0.064685	49	0	49	0	0.68	Sub	0.34	2.67			polymorphism	3.581						
OVA_003_C1	PRIMARY	chr2	88382213	88382213	T	C	SMYD1	intronic	NA	NA	324	251	575	0.436522	49	0	49	0	1	Clonal	0.86	1.81			polymorphism	3.581						
OVA_003_C2	PRIMARY	chr2	88382213	88382213	T	C	SMYD1	intronic	NA	NA	330	244	574	0.425087	49	0	49	0	1	Clonal	0.7	3.61			polymorphism	3.581						

Sample	Type	Chr	Start	End	Ref	Alt	Gene	unc.ensGen	icFunc.ens	hange.ens	t_ref	t_alt	t_total	t_VAF	n_ref	n_alt	n_total	ormal_VA	ncer_cell_f	on-based C	purity	ploidy	Cancer Gene	Driver Gene	Mutation Taster	ADD(PHRE	otein_chan	CHASM	THMM_Pr	OLYPHEN-	REDICTION	ICTION (cutoff=0.05)
OVA_003_C3	PRIMARY	chr2	88382213	88382213	T	C	SMYD1	intronic	NA	NA	342	263	605	0.434711	49	0	49	0	1	Clonal	0.82	1.81			polymorphism	3.581						
OVA_365_M3	METASTASIS	chr16	88496002	88496002	G	T	ZNF469	exonic	onymous S	708A,ENSG	36	4	40	0.1	86	0	86	0	0.14	Sub	0.82	1.89			disease_causing	5.664		Passenger			Neutral	Tolerated
OVA_365_C1	Contralateral ovary	chr16	88496002	88496002	G	T	ZNF469	exonic	onymous S	708A,ENSG	41	18	59	0.305085	86	0	86	0	1	Clonal	0.42	1.83			disease_causing	5.664		Passenger			Neutral	Tolerated
OVA_365_C2	PRIMARY	chr16	88496002	88496002	G	T	ZNF469	exonic	onymous S	708A,ENSG	49	32	81	0.395062	86	0	86	0	1	Clonal	0.62	1.92			disease_causing	5.664		Passenger			Neutral	Tolerated
OVA_365_M3	METASTASIS	chr16	88496003	88496003	C	T	ZNF469	exonic	ynonymou	709S,ENSG	36	4	40	0.1	85	0	85	0	0.14	Sub	0.82	1.89			polymorphism	21	p.P709S	Passenger	SENGER/OT	damaging	Deleterious	Damaging
OVA_365_C1	Contralateral ovary	chr16	88496003	88496003	C	T	ZNF469	exonic	ynonymou	709S,ENSG	41	18	59	0.305085	85	0	85	0	1	Clonal	0.42	1.83			polymorphism	21	p.P709S	Passenger	SENGER/OT	damaging	Deleterious	Damaging
OVA_365_C2	PRIMARY	chr16	88496003	88496003	C	T	ZNF469	exonic	ynonymou	709S,ENSG	49	32	81	0.395062	85	0	85	0	1	Clonal	0.62	1.92			polymorphism	21	p.P709S	Passenger	SENGER/OT	damaging	Deleterious	Damaging
OVA_013_M1	METASTASIS	chr16	88500684	88500684	C	G	ZNF469	exonic	ynonymou	2269S,ENSG	62	24	86	0.27907	40	1	41	0.02439	1	Clonal	0.67	3.06			polymorphism	0.859	p.T2269S	Passenger	SENGER/OT	benign	Neutral	Tolerated
OVA_013_C1	PRIMARY	chr16	88500684	88500684	C	G	ZNF469	exonic	ynonymou	2269S,ENSG	52	22	74	0.297297	40	1	41	0.02439	1	Clonal	0.72	3.1			polymorphism	0.859	p.T2269S	Passenger	SENGER/OT	benign	Neutral	Tolerated
OVA_013_C2	PRIMARY	chr16	88500684	88500684	C	G	ZNF469	exonic	ynonymou	2269S,ENSG	48	31	79	0.392405	40	1	41	0.02439	0.9	Clonal	0.87	3.05			polymorphism	0.859	p.T2269S	Passenger	SENGER/OT	benign	Neutral	Tolerated
OVA_013_C3	PRIMARY	chr16	88500684	88500684	C	G	ZNF469	exonic	ynonymou	2269S,ENSG	44	25	69	0.362319	40	1	41	0.02439	0.79	Clonal	0.91	3.01			polymorphism	0.859	p.T2269S	Passenger	SENGER/OT	benign	Neutral	Tolerated
OVA_365_C1	Contralateral ovary	chr16	88600958	88600958	G	A	ZFPM1	exonic	onymous S	000319555	2	5	7	0.714286	20	0	20	0	1	Clonal	0.42	1.83			polymorphism	7.926		Passenger			Neutral	Tolerated
OVA_365_C2	PRIMARY	chr16	88600958	88600958	G	A	ZFPM1	exonic	onymous S	000319555	10	14	24	0.583333	20	0	20	0	1	Clonal	0.62	1.92			polymorphism	7.926		Passenger			Neutral	Tolerated
OVA_047_M1	METASTASIS	chr16	88932088	88932088	C	A	PABPN1L	intronic	NA	NA	173	90	263	0.342205	70	0	70	0	1	Sub	0.47	2.01			polymorphism	3.609						
OVA_047_M2	METASTASIS	chr16	88932088	88932088	C	A	PABPN1L	intronic	NA	NA	273	10	283	0.035336	70	0	70	0	0.12	Sub	0.44	2.01			polymorphism	3.609						
OVA_047_M3	METASTASIS	chr16	88932088	88932088	C	A	PABPN1L	intronic	NA	NA	117	6	123	0.04878	70	0	70	0	0.16	Sub	0.6	3.02			polymorphism	3.609						
OVA_003_M1	METASTASIS	chr9	88938700	88938700	T	C	ZCCHC6	exonic	onymous S	000375961:	485	122	607	0.200988	60	0	60	0	0.91	Sub	0.57	1.84			disease_causing	10.94		Passenger			Neutral	Tolerated
OVA_003_M2	METASTASIS	chr9	88938700	88938700	T	C	ZCCHC6	exonic	onymous S	000375961:	506	100	606	0.165017	60	0	60	0	0.89	Sub	0.46	1.81			disease_causing	10.94		Passenger			Neutral	Tolerated
OVA_003_M3	METASTASIS	chr9	88938700	88938700	T	C	ZCCHC6	exonic	onymous S	000375961:	607	40	647	0.061824	60	0	60	0	0.9	Sub	0.34	2.67			disease_causing	10.94		Passenger			Neutral	Tolerated
OVA_003_C1	PRIMARY	chr9	88938700	88938700	T	C	ZCCHC6	exonic	onymous S	000375961:	351	257	608	0.422697	60	0	60	0	0.98	Clonal	0.86	1.81			disease_causing	10.94		Passenger			Neutral	Tolerated
OVA_003_C2	PRIMARY	chr9	88938700	88938700	T	C	ZCCHC6	exonic	onymous S	000375961:	410	218	628	0.347134	60	0	60	0	1	Clonal	0.7	3.61			disease_causing	10.94		Passenger			Neutral	Tolerated
OVA_003_C3	PRIMARY	chr9	88938700	88938700	T	C	ZCCHC6	exonic	onymous S	000375961:	383	246	629	0.391097	60	0	60	0	0.95	Clonal	0.82	1.81			disease_causing	10.94		Passenger			Neutral	Tolerated
OVA_003_M1	METASTASIS	chr10	89468827	89468827	T	G	PAPSS2	intronic	NA	NA	190	124	314	0.394904	27	0	27	0	1	Sub	0.57	1.84			polymorphism	0.177						
OVA_003_M2	METASTASIS	chr10	89468827	89468827	T	G	PAPSS2	intronic	NA	NA	293	137	430	0.318605	27	0	27	0	1	Sub	0.46	1.81			polymorphism	0.177						
OVA_003_M3	METASTASIS	chr10	89468827	89468827	T	G	PAPSS2	intronic	NA	NA	316	39	355	0.109859	27	0	27	0	0.83	Sub	0.34	2.67			polymorphism	0.177						
OVA_003_C1	PRIMARY	chr10	89468827	89468827	T	G	PAPSS2	intronic	NA	NA	57	219	276	0.793478	27	0	27	0	1	Clonal	0.86	1.81			polymorphism	0.177						
OVA_003_C2	PRIMARY	chr10	89468827	89468827	T	G	PAPSS2	intronic	NA	NA	77	213	290	0.734483	27	0	27	0	1	Clonal	0.7	3.61			polymorphism	0.177						
OVA_003_C3	PRIMARY	chr10	89468827	89468827	T	G	PAPSS2	intronic	NA	NA	87	213	300	0.71	27	0	27	0	1	Clonal	0.82	1.81			polymorphism	0.177						
OVA_365_M1	METASTASIS	chr4	89601253	89601253	G	C	HERC3	exonic	ynonymou	0H,ENSG00	157	123	280	0.439286	31	0	31	0	1	Clonal	0.85	1.87			disease_causing	24.2	p.D736H	Passenger	SENGER/OT	damaging	Neutral	Tolerated
OVA_365_M2	METASTASIS	chr4	89601253	89601253	G	C	HERC3	exonic	ynonymou	0H,ENSG00	90	15	105	0.142857	31	0	31	0	0.71	Clonal	0.4	1.81			disease_causing	24.2	p.D736H	Passenger	SENGER/OT	damaging	Neutral	Tolerated
OVA_365_M3	METASTASIS	chr4	89601253	89601253	G	C	HERC3	exonic	ynonymou	0H,ENSG00	116	74	190	0.389474	31	0	31	0	0.95	Clonal	0.82	1.89			disease_causing	24.2	p.D736H	Passenger	SENGER/OT	damaging	Neutral	Tolerated
OVA_365_C1	Contralateral ovary	chr4	89601253	89601253	G	C	HERC3	exonic	ynonymou	0H,ENSG00	112	23	135	0.17037	31	0	31	0	0.82	Clonal	0.42	1.83			disease_causing	24.2	p.D736H	Passenger	SENGER/OT	damaging	Neutral	Tolerated
OVA_365_C2	PRIMARY	chr4	89601253	89601253	G	C	HERC3	exonic	ynonymou	0H,ENSG00	128	61	189	0.322751	31	0	31	0	1	Clonal	0.62	1.92			disease_causing	24.2	p.D736H	Passenger	SENGER/OT	damaging	Neutral	Tolerated
OVA_048_M3	METASTASIS	chr6	89790893	89790893	C	A	PNRC1	exonic	ynonymou	000003360	31	11	42	0.261905	82	0	82	0	1	Clonal	0.59	3.39			disease_causing	23	p.P94T	Passenger	SENGER/OT	damaging	Neutral	Tolerated
OVA_378_M1	METASTASIS	chr5	89979692	89979692	A	G	GPR98	exonic	ynonymou	000405460:	476	26	502	0.051793	40	0	40	0	0.14	Sub	0.72	2.57			polymorphism	15.1	p.N1985S	Passenger	SENGER/OT	benign	Neutral	Tolerated
OVA_378_M2	METASTASIS	chr5	89979692	89979692	A	G	GPR98	exonic	ynonymou	000405460:	485	38	523	0.072658	40	0	40	0	0.17	Sub	0.85	2.57			polymorphism	15.1	p.N1985S	Passenger	SENGER/OT	benign	Neutral	Tolerated
OVA_378_M3	METASTASIS	chr5	89979692	89979692	A	G	GPR98	exonic	ynonymou	000405460:	533	40	573	0.069808	40	0	40	0	0.21	Sub	0.67	2.57			polymorphism	15.1	p.N1985S	Passenger	SENGER/OT	benign	Neutral	Tolerated
OVA_048_M1	METASTASIS	chr1	90152010	90152010	G	C	LRRC8C	intronic	NA	NA	518	44	562	0.078292	67	0	67	0	0.43	Sub	0.56	3.94			polymorphism	3.895						
OVA_365_M1	METASTASIS	chr15	90220736	90220736	T	A	PLIN1	splicing	NA	NA	219	164	383	0.428198	42	0	42	0	1	Clonal	0.85	1.87			disease_causing	32						
OVA_365_M2	METASTASIS	chr15	90220736	90220736	T	A	PLIN1	splicing	NA	NA	179	48	227	0.211454	42	0	42	0	1	Clonal	0.4	1.81			disease_causing	32						
OVA_365_M3	METASTASIS	chr15	90220736	90220736	T	A	PLIN1	splicing	NA	NA	180	144	324	0.444444	42	0	42	0	1	Clonal	0.82	1.89			disease_causing	32						
OVA_365_C1	Contralateral ovary	chr15	90220736	90220736	T	A	PLIN1	splicing	NA	NA	232	74	306	0.24183	42	0	42	0	1	Clonal	0.42	1.83			disease_causing	32						
OVA_365_C2	PRIMARY	chr15	90220736	90220736	T	A	PLIN1	splicing	NA	NA	236	102	338	0.301775	42	0	42	0	0.98	Clonal	0.62	1.92			disease_causing	32						
OVA_003_M1	METASTASIS	chr6	90576648	90576648	A	G	CASP8AP2	cRNA_exon	NA	NA	188	79	267	0.29588	47	0	47	0	1	Clonal	0.57	1.84				10.36						
OVA_003_M2	METASTASIS	chr6	90576648	90576648	A	G	CASP8AP2	cRNA_exon	NA	NA	279	97	376	0.257979	47	0	47	0	1	Clonal	0.46	1.81				10.36						
OVA_003_M3	METASTASIS	chr6	90576648	90576648	A	G	CASP8AP2	cRNA_exon	NA	NA	248	28	276	0.101449	47	0	47	0	1	Clonal	0.34	2.67				10.36						
OVA_003_C1	PRIMARY	chr6	90576648	90576648	A	G	CASP8AP2	cRNA_exon	NA	NA	163	130	293	0.443686	47	0	47	0	1	Clonal	0.86	1.81				10.36						
OVA_003_C2	PRIMARY	chr6	90576648	90576648	A	G	CASP8AP2	cRNA_exon	NA	NA	180	143	323	0.442724	47	0	47	0	1	Clonal	0.7	3.61				10.36						
OVA_003_C3	PRIMARY	chr6	90576648	90576648	A	G	CASP8AP2	cRNA_exon	NA	NA	196	145	341	0.42522	47	0	47	0	1	Clonal	0.82	1.81				10.36						
OVA_003_M1	METASTASIS	chr6	90660860	90660861	GC	AA	BACH2	exonic	ynonymou	000053798	149	65	214	0.303738	86	0	86	0	1	Sub	0.57	1.84			polymorphism	11.4	p.A322V	Passenger	SENGER/OT	benign	Neutral	Tolerated
OVA_003_M2	METASTASIS	chr6	90660860	90660861	GC	AA	BACH2	exonic	ynonymou	000053798	191	37	228	0.162281	86	0	86	0	0.71	Sub	0.46	1.81			polymorphism	11.4	p.A322V	Passenger	SENGER/OT	benign	Neutral	Tolerated
OVA_003_M3	METASTASIS	chr6	90660860	90660861	GC	AA	BACH2	exonic	ynonymou	000053798	203	14	217	0.064516	86	0	86	0	0.68	Sub	0.34	2.67			polymorphism	11.4	p.A322V	Passenger	SENGER/OT	benign	Neutral	Tolerated
OVA_003_C1	PRIMARY	chr6	90660860	90660861	GC	AA	BACH2	exonic	ynonymou	000053798	127	110	237	0.468	86	0	86	0	1	Clonal	0.86	1.81			polymorphism	11.4	p.A322V	Passenger	SENGER/OT	benign	Neutral	Tolerated
OVA_003_C2	PRIMARY	chr6	90660860	90660861	GC	AA	BACH2	exonic	ynonymou	000053798	167	102	269	0.38	86	0	86	0	1	Clonal	0.7	3.61			polymorphism	11.4	p.A322V	Passenger	SENGER/OT	benign	Neutral	Tolerated
OVA_003_C3	PRIMARY	chr6	90660860	90660861	GC	AA	BACH2	exonic	ynonymou	000053798	170	141	311	0.4555	86	0	86	0	1	Clonal	0.82	1.81			polymorphism	11.4	p.A322V	Passenger	SENGER/OT	benign	Neutral	Tolerated
OVA_013_M1	METASTASIS	chr14	90723066	90723066	C	A	PSMC1	intronic	NA	NA	17	5	22	0.227273	43	0	43	0	0.91	Clonal	0.67	3.06			polymorphism	6.725						
OVA_013_C1	PRIMARY	chr14	90723066	90723066	C	A	PSMC1	intronic	NA	NA	17	11	28	0.392857	43	0	43	0	1	Clonal	0.72	3.1			polymorphism	6.725						
OVA_013_C2	PRIMARY	chr14	90723066	90723066	C	A	PSMC1	intronic	NA	NA	16	16	32	0.5	43	0	43	0	1	Clonal	0.87	3.05			polymorphism	6.725						
OVA_013_C3	PRIMARY	chr14	90723066	90723066	C	A	PSMC1	intronic	NA	NA	20	19	39	0.487179	43	0	43	0	1	Clonal	0.91	3.01			polymorphism	6.725						
OVA_047_C1	PRIMARY	chr10	90966665	90966665	G	C	CH25H	exonic	ynonymou	000037185	544	57	601	0.094842	218	0	218	0	0.32	Sub	0.6	2.91			polymorphism	0.051	p.L129V	Passenger	SENGER/OT	benign	Neutral	Tolerated
OVA_048_M1	METASTASIS	chr14	91142836	91142836	C	A	TTC7B	intronic	NA	NA	381	239	620	0.385484	35	1	36	0.027778	1	Clonal	0.56	3.94			polymorphism	7.417						
OVA_048_M2	METASTASIS	chr14	91142836	91142836	C	A	TTC7B	intronic	NA	NA	413	203	616	0.329545	35	1	36	0.027778	1	Clonal	0.4	3.87			polymorphism	7.417						
OVA_048_M3	METASTASIS	chr14	91142836	91142836	C	A	TTC7B	intronic	NA	NA	312	269	581	0.462995	35	1	36	0.027778	1	Clonal	0.59	3.39			polymorphism	7.417						
OVA_048_C1	PRIMARY	chr14	91142836	91142836	C	A	TTC7B	intronic	NA	NA	269	108	377	0.286472	35	1	36	0.027778	0.86	Clonal	0.67	1.82			polymorphism	7.417						
OVA_048_C2	PRIMARY	chr14	91142836	91142836	C	A	TTC7B	intronic	NA	NA	415	172	587	0.293015	35	1	36	0.027778	1	Clonal	0.72	1.85			polymorphism	7.417						
OVA_048_C3	PRIMARY	chr14	91142836	91142836	C	A	TTC7B	intronic	NA	NA	268	155	423	0.36643	35	1	36	0.027778	0.99	Clonal	0.74	1.8			polymorphism	7.417						
OVA_378_M1	METASTASIS	chr10	91162751	91162751	T	C	IFIT1	exonic	ynonymou	I240T,ENSG	102	330	432	0.763889	41	0	41	0	1	Clonal	0.72	2.57			polymorphism	22.4	p.I240T	Passenger	SENGER/OT	damaging	Deleterious	Damaging
OVA_378_M2	METASTASIS	chr10	91162751	91162751	T	C	IFIT1	exonic	ynonymou	I240T,ENSG	63	346	409	0.845966	41	0	41	0	1	Clonal	0.85	2.57			polymorphism	22.4	p.I240T	Passenger	SENGER/OT	damaging	Deleterious	Damaging
OVA_378_M3	METASTASIS	chr10	91162751	91162751	T	C	IFIT1	exonic	ynonymou	I240T,ENSG	157	399	556	0.717626	41	0	41	0	1	Clonal	0.67	2.57			polymorphism	22.4	p.I240T	Passenger	SENGER/OT	damaging	Deleterious	Damaging
OVA_378_C1	PRIMARY	chr10	91162751	91162751	T	C	IFIT1	exonic	ynonymou	I240T,ENSG	195	327	522	0.626437	41	0	41	0	1	Clonal	0.59	2.6			polymorphism	22.4	p.I240T	Passenger	SENGER/OT	damaging	Deleterious	Damaging
OVA_378_C2	PRIMARY	chr10	91162751	91162751	T	C	IFIT1	exonic	ynonymou	I240T,ENSG	226	295	521	0.566219	41	0	41	0	1	Clonal	0.56	2.59			polymorphism	22.4	p.I240T	Passenger	SENGER/OT	damaging	Deleterious	Damaging
OVA_378_C3	PRIMARY	chr10	91162751	91162751	T	C	IFIT1	exonic	ynonymou	I240T,ENSG	204	250	454	0.550661	41	0	41	0	1	Clonal	0.51	2.6			polymorphism	22.4	p.I240T	Passenger	SENGER/OT	damaging	Deleterious	Damaging
OVA_365_M1	METASTASIS	chr11	92087724	92087724	C	G	FAT3	exonic	ynonymou	.816V,ENSG	318	273	591	0.461929	61	0	61	0	1	Sub	0.85	1.87	Yes	Yes	disease_causing	14.05	p.L816V	Passenger	SENGER/OT	damaging	Neutral	Damaging
OVA_365_M2	METASTASIS	chr11	92087724	92087724	C	G	FAT3	exonic	ynonymou	.816V,ENSG	461	88	549	0.160291	61	0	61	0	0.8	Sub	0.4	1.81	Yes	Yes	disease_causing	14.05	p.L816V	Passenger	SENGER/OT	damaging	Neutral	Damaging
OVA_365_M3	METASTASIS	chr11	92087724	92087724	C	G	FAT3	exonic	ynonymou	.816V,ENSG	347	252	599	0.420701	61	0	61	0	1	Sub	0.82	1.89	Yes	Yes	disease_causing	14.05	p.L816V	Passenger	SENGER/OT	damaging	Neutral	Damaging
OVA_365_C1	Contralateral ovary	chr11	92087724	92087724	C	G	FAT3	exonic	ynonymou	.816V,ENSG	410	123	533	0.230769	61	0	61	0	1	Clonal	0.42	1.83	Yes	Yes	disease_causing	14.05	p.L816V	Passenger	SENGER/OT	damaging	Neutral	Damaging
OVA_365_C2	PRIMARY	chr11	92087724	92087724	C	G	FAT3	exonic	ynonymou	.816V,ENSG	484	120	604	0.198675	61	0	61	0	0.84	Sub	0.62	1.92	Yes	Yes	disease_causing	14.05	p.L816V	Passenger	SENGER/OT	damaging	Neutral	Damaging
OVA_365_M1	METASTASIS	chr7	92120733	92120733	A	G	PEX1	exonic	onymous S	000428214:	230	353	583	0.605489	42	0	42	0	1	Clonal	0.85	1.87			disease_causing	13.82		Passenger			Neutral	Tolerated
OVA_365_M2	METASTASIS	chr7	92120733	92120733	A	G	PEX1	exonic	onymous S	000428214:	307	155	462	0.335498	42	0	42	0	1	Clonal	0.4	1.81			disease_causing	13.82		Passenger			Neutral	Tolerated
OVA_365_M3	METASTASIS	chr7	92120733	92120733	A	G	PEX1	exonic	onymous S	000428214:	271	297	568	0.522887	42	0	42	0	1	Clonal	0.82	1.89			disease_causing	13.82		Passenger			Neutral	Tolerated
OVA_365_C1	Contralateral ovary	chr7	92120733	92120733	A	G	PEX1	exonic	onymous S	000428214:	308	153	461	0.331887	42	0	42	0	1	Clonal	0.42	1.83			disease_causing	13.82		Passenger			Neutral	Tolerated
OVA_365_C2	PRIMARY	chr7	92120733	92120733	A	G	PEX1	exonic	onymous S	000428214:	288	244	532	0.458647	42	0	42	0	1	Clonal	0.62	1.92			disease_causing	13.82		Passenger			Neutral	Tolerated
OVA_048_M1	METASTASIS	chr11	92577560	92577560	C	T	FAT3	exonic	ynonymou	V,ENSG000	544	57	601	0.094842	27	0	27	0	0.53	Sub	0.56	3.94	Yes	No	disease_causing	21.9	p.A3676V	Passenger	SENGER/OT	benign	Neutral	Tolerated
OVA_365_C2	PRIMARY	chr7	92730943	92730943	C	G	SAMD9	exonic	ynonymou	000379958	409	114	523	0.217973	92	0	92	0	0.93	Clonal	0.62	1.92			polymorphism	8.112	p.E1490Q	Passenger	SENGER/OT	benign	Neutral	Tolerated
OVA_047_C1	PRIMARY	chr5	92923759	92923759	G	A	NR2F1	exonic	onymous S	0000032711	375	166	541	0.306839	114	0	114	0	1	Clonal	0.6	2.91			disease_causing	14.8		Passenger			Neutral	Tolerated
OVA_047_C2	PRIMARY	chr5	92923759	92923759	G	A	NR2F1	exonic	onymous S	0000032711	372	188	560	0.335714	114	0	114	0	1	Clonal	0.64	2.76			disease_causing	14.8		Passenger			Neutral	Tolerated
OVA_047_C3	Contralateral ovary	chr5	92923759	92923759	G	A	NR2F1	exonic	onymous S	0000032711	342	151	493	0.306288	114	0	114	0	1	Clonal	0.6	2.94			disease_causing	14.8		Passenger			Neutral	Tolerated
OVA_048_C1	PRIMARY	chrX	92964392	92964392	A	T	FAM133A	UTR5	NA	NA	149	73	222	0.328829	37	0	37	0	0.98	Clonal	0.67	1.82			polymorphism	8.303						
OVA_048_C2	PRIMARY	chrX	92964392	92964392	A	T	FAM133A	UTR5	NA	NA	284	184	468	0.393162	37	0	37	0	1	Clonal	0.72	1.85			polymorphism	8.303						
OVA_048_C3	PRIMARY	chrX	92964392	92964392	A	T	FAM133A	UTR5	NA	NA	177	115	292	0.393836	37	0	37	0	1	Clonal	0.74	1.8			polymorphism	8.303						
OVA_013_M1	METASTASIS	chr15	93558119	93558119	A	G	CHD2	exonic	ynonymou	0394196:e:	410	159	569	0.279438	111	0	111	0	1	Clonal	0.67	3.06	Yes	Yes	disease_causing	24.9	p.K1629R	Passenger	SENGER/OT	damaging	Neutral	Tolerated
OVA_013_C1	PRIMARY	chr15	93558119	93558119	A	G	CHD2	exonic	ynonymou	0394196:e:	460	145	605	0.239669	111	0	111	0	1	Sub	0.72	3.1	Yes	Yes	disease_causing	24.9	p.K1629R	Passenger	SENGER/OT	damaging	Neutral	Tolerated
OVA_013_C2	PRIMARY	chr15	93558119	93558119	A	G	CHD2	exonic	ynonymou	0394196:e:	442	133	575	0.231304	111	0	111	0	0.99	Sub	0.87	3.05	Yes	Yes	disease_causing	24.9	p.K1629R	Passenger	SENGER/OT	damaging	Neutral	Tolerated
OVA_013_C3	PRIMARY	chr15	93558119	93558119	A	G	CHD2	exonic	ynonymou	0394196:e:	484	143	627	0.22807	111	0	111	0	0.73	Sub	0.91	3.01	Yes	Yes	disease_causing	24.9	p.K1629R	Passenger	SENGER/OT	damaging	Neutral	Tolerated
OVA_365_M1	METASTASIS	chr1	93584899	93584899	G	T	MTF2	exonic	onymous S	246T,ENSG	36	14	50	0.28	21	0	21	0	0.66	Sub	0.85	1.87			disease_causing	10.69		Passenger			Neutral	Tolerated
OVA_365_M2	METASTASIS	chr1	93584899	93584899	G	T	MTF2	exonic	onymous S	246T,ENSG	35	6	41	0.146341	21	0	21	0	0.73	Sub	0.4	1.81			disease_causing	10.69		Passenger			Neutral	Tolerated
OVA_365_M3	METASTASIS	chr1	93584899	93584899	G	T	MTF2	exonic	onymous S	246T,ENSG	22	17	39	0.435897	21	0	21	0	1	Sub	0.82	1.89			disease_causing	10.69		Passenger			Neutral	Tolerated
OVA_365_C1	Contralateral ovary	chr1	93584899	93584899	G	T	MTF2	exonic	onymous S	246T,ENSG	28	9	37	0.243243	21	0	21	0	1	Clonal	0.42	1.83			disease_causing	10.69		Passenger			Neutral	Tolerated
OVA_365_C2	PRIMARY	chr1	93584899	93584899	G	T	MTF2	exonic	onymous S	246T,ENSG	38	12	50	0.24	21	0	21	0	0.78	Clonal	0.62	1.92			disease_causing	10.69		Passenger			Neutral	Tolerated
OVA_047_M1	METASTASIS	chr10	93756861	93756861	G	A	BTAF1	UTR5	NA	NA	501	141	642	0.219626	67	0	67	0	0.71	Sub	0.47	2.01			polymorphism	14.31						

Sample	Type	Chr	Start	End	Ref	Alt	Gene	unc.ensGen	icFunc.ens	hange.ens(	t_ref	t_alt	t_total	t_VAF	n_ref	n_alt	n_total	ormal_VA	ncer_cell_f	on-based C	purity	ploidy	Cancer Gene	Driver Gene	Mutation Taster	ADD(PHRE	otein_chan	CHASM	THMM_Pr	OLYPHEN-	REDICTION	ICTION (cutoff=0.05)
OVA_048_C2	PRIMARY	chr14	93995611	93995611	G	C	UNC79	intronic	NA	NA	360	204	564	0.361702	23	0	23	0	1	Clonal	0.72	1.85			polymorphism	10.62						
OVA_047_M1	METASTASIS	chr9	94486599	94486599	A	G	ROR2	exonic	ynonymou	I726T,ENSG	281	63	344	0.18314	137	0	137	0	0.96	Clonal	0.47	2.01			polymorphism	15.68	p.I726T	Passenger	CANCER	benign	Neutral	Tolerated
OVA_047_M2	METASTASIS	chr9	94486599	94486599	A	G	ROR2	exonic	ynonymou	I726T,ENSG	300	56	356	0.157303	137	0	137	0	0.87	Clonal	0.44	2.01			polymorphism	15.68	p.I726T	Passenger	CANCER	benign	Neutral	Tolerated
OVA_047_M3	METASTASIS	chr9	94486599	94486599	A	G	ROR2	exonic	ynonymou	I726T,ENSG	96	23	119	0.193277	137	0	137	0	1	Clonal	0.6	3.02			polymorphism	15.68	p.I726T	Passenger	CANCER	benign	Neutral	Tolerated
OVA_003_C3	PRIMARY	chr11	94532634	94532634	C	G	AMOTL1	exonic	ynonymou	A93G,ENSG	651	25	676	0.036982	105	0	105	0	0.09	Sub	0.82	1.81			polymorphism	16.3	p.A93G	Passenger	SENGER/OT	benign	Neutral	Tolerated
OVA_378_C2	PRIMARY	chr5	94800403	94800403	-	AGG	TTC37	intronic	NA	NA	484	152	636	0.238994	53	1	54		0.85	Sub	0.56	2.59										
OVA_003_M1	METASTASIS	chr7	94898800	94898800	G	C	PPP1R9A	exonic	ynonymou	013N,ENSG	389	131	520	0.251923	19	0	19	0	0.89	Sub	0.57	1.84			polymorphism	16.54	p.K1035N	Passenger	SENGER/OT	benign	Neutral	Damaging
OVA_003_M2	METASTASIS	chr7	94898800	94898800	G	C	PPP1R9A	exonic	ynonymou	013N,ENSG	410	124	534	0.23221	19	0	19	0	1	Sub	0.46	1.81			polymorphism	16.54	p.K1035N	Passenger	SENGER/OT	benign	Neutral	Damaging
OVA_003_M3	METASTASIS	chr7	94898800	94898800	G	C	PPP1R9A	exonic	ynonymou	013N,ENSG	521	26	547	0.047532	19	0	19	0	0.5	Sub	0.34	2.67			polymorphism	16.54	p.K1035N	Passenger	SENGER/OT	benign	Neutral	Damaging
OVA_003_C1	PRIMARY	chr7	94898800	94898800	G	C	PPP1R9A	exonic	ynonymou	013N,ENSG	305	233	538	0.433086	19	0	19	0	1	Clonal	0.86	1.81			polymorphism	16.54	p.K1035N	Passenger	SENGER/OT	benign	Neutral	Damaging
OVA_003_C2	PRIMARY	chr7	94898800	94898800	G	C	PPP1R9A	exonic	ynonymou	013N,ENSG	346	186	532	0.349624	19	0	19	0	1	Clonal	0.7	3.61			polymorphism	16.54	p.K1035N	Passenger	SENGER/OT	benign	Neutral	Damaging
OVA_003_C3	PRIMARY	chr7	94898800	94898800	G	C	PPP1R9A	exonic	ynonymou	013N,ENSG	313	259	572	0.452797	19	0	19	0	1	Clonal	0.82	1.81			polymorphism	16.54	p.K1035N	Passenger	SENGER/OT	benign	Neutral	Damaging
OVA_365_M1	METASTASIS	chr14	94955970	94955970	A	G	SERPINA12	exonic	ynonymou	347P,ENSG	195	106	301	0.352159	46	0	46	0	0.83	Sub	0.85	1.87			disease_causing	24.4	p.L347P	Passenger	SENGER/OT	damaging	Deleterious	Damaging
OVA_365_M2	METASTASIS	chr14	94955970	94955970	A	G	SERPINA12	exonic	ynonymou	347P,ENSG	187	61	248	0.245968	46	0	46	0	1	Sub	0.4	1.81			disease_causing	24.4	p.L347P	Passenger	SENGER/OT	damaging	Deleterious	Damaging
OVA_365_M3	METASTASIS	chr14	94955970	94955970	A	G	SERPINA12	exonic	ynonymou	347P,ENSG	181	119	300	0.396667	46	0	46	0	0.97	Sub	0.82	1.89			disease_causing	24.4	p.L347P	Passenger	SENGER/OT	damaging	Deleterious	Damaging
OVA_365_C1	Contralateral ovary	chr14	94955970	94955970	A	G	SERPINA12	exonic	ynonymou	347P,ENSG	198	49	247	0.198381	46	0	46	0	0.95	Clonal	0.42	1.83			disease_causing	24.4	p.L347P	Passenger	SENGER/OT	damaging	Deleterious	Damaging
OVA_365_C2	PRIMARY	chr14	94955970	94955970	A	G	SERPINA12	exonic	ynonymou	347P,ENSG	203	92	295	0.311864	46	0	46	0	1	Clonal	0.62	1.92			disease_causing	24.4	p.L347P	Passenger	SENGER/OT	damaging	Deleterious	Damaging
OVA_048_M1	METASTASIS	chr10	95445024	95445024	T	C	FRA10AC1	exonic	ynonymou	202G,ENSG	122	41	163	0.251534	53	0	53	0	1	Clonal	0.56	3.94			disease_causing	27.9	p.R202G	Passenger	SENGER/OT	damaging	Deleterious	Damaging
OVA_048_M2	METASTASIS	chr10	95445024	95445024	T	C	FRA10AC1	exonic	ynonymou	202G,ENSG	139	33	172	0.19186	53	0	53	0	1	Clonal	0.4	3.87			disease_causing	27.9	p.R202G	Passenger	SENGER/OT	damaging	Deleterious	Damaging
OVA_048_M3	METASTASIS	chr10	95445024	95445024	T	C	FRA10AC1	exonic	ynonymou	202G,ENSG	92	42	134	0.313433	53	0	53	0	1	Clonal	0.59	3.39			disease_causing	27.9	p.R202G	Passenger	SENGER/OT	damaging	Deleterious	Damaging
OVA_048_C1	PRIMARY	chr10	95445024	95445024	T	C	FRA10AC1	exonic	ynonymou	202G,ENSG	113	63	176	0.357955	53	0	53	0	1	Clonal	0.67	1.82			disease_causing	27.9	p.R202G	Passenger	SENGER/OT	damaging	Deleterious	Damaging
OVA_048_C2	PRIMARY	chr10	95445024	95445024	T	C	FRA10AC1	exonic	ynonymou	202G,ENSG	110	63	173	0.364162	53	0	53	0	1	Clonal	0.72	1.85			disease_causing	27.9	p.R202G	Passenger	SENGER/OT	damaging	Deleterious	Damaging
OVA_048_C3	PRIMARY	chr10	95445024	95445024	T	C	FRA10AC1	exonic	ynonymou	202G,ENSG	140	80	220	0.363636	53	0	53	0	0.98	Clonal	0.74	1.8			disease_causing	27.9	p.R202G	Passenger	SENGER/OT	damaging	Deleterious	Damaging
OVA_003_C1	PRIMARY	chr1	95538441	95538441	A	G	ALG14	exonic	ynonymou	T00000370	552	52	604	0.086093	211	0	211	0	0.2	Sub	0.86	1.81			polymorphism	23.1	p.L5P	Passenger	SENGER/OT	damaging	Deleterious	Damaging
OVA_013_M1	METASTASIS	chr8	95565611	95565611	A	G	KIAA1429	splicing	NA	NA	533	91	624	0.145833	147	0	147	0	0.73	Sub	0.67	3.06			disease_causing	34		Passenger				
OVA_013_C1	PRIMARY	chr8	95565611	95565611	A	G	KIAA1429	splicing	NA	NA	500	90	590	0.152542	147	0	147	0	0.58	Sub	0.72	3.1			disease_causing	34		Passenger				
OVA_013_C2	PRIMARY	chr8	95565611	95565611	A	G	KIAA1429	splicing	NA	NA	487	79	566	0.139576	147	0	147	0	0.46	Sub	0.87	3.05			disease_causing	34		Passenger				
OVA_013_C3	PRIMARY	chr8	95565611	95565611	A	G	KIAA1429	splicing	NA	NA	515	100	615	0.162602	147	0	147	0	0.52	Sub	0.91	3.01			disease_causing	34		Passenger				
OVA_047_M1	METASTASIS	chr5	95735615	95735615	G	C	PCSK1	RNA_intro	NA	NA	377	223	600	0.371667	19	0	19	0	1	Clonal	0.47	2.01			polymorphism	2.594						
OVA_047_M2	METASTASIS	chr5	95735615	95735615	G	C	PCSK1	RNA_intro	NA	NA	463	154	617	0.249595	19	0	19	0	0.88	Clonal	0.44	2.01			polymorphism	2.594						
OVA_047_M3	METASTASIS	chr5	95735615	95735615	G	C	PCSK1	RNA_intro	NA	NA	136	94	230	0.408696	19	0	19	0	1	Clonal	0.6	3.02			polymorphism	2.594						
OVA_048_C1	PRIMARY	chr10	95993931	95993931	A	C	PLCE1	exonic	onymous S	692T,ENSG	259	123	382	0.32199	42	0	42	0	0.96	Sub	0.67	1.82			disease_causing	0.489		Passenger			Neutral	Tolerated
OVA_048_C2	PRIMARY	chr10	95993931	95993931	A	C	PLCE1	exonic	onymous S	692T,ENSG	362	160	522	0.306513	42	0	42	0	0.85	Sub	0.72	1.85			disease_causing	0.489		Passenger			Neutral	Tolerated
OVA_048_C3	PRIMARY	chr10	95993931	95993931	A	C	PLCE1	exonic	onymous S	692T,ENSG	316	143	459	0.311547	42	0	42	0	0.84	Sub	0.74	1.8			disease_causing	0.489		Passenger			Neutral	Tolerated
OVA_048_M1	METASTASIS	chr6	96044626	96044626	C	G	MANEA	exonic	onymous S	000035881	247	195	442	0.441176	65	0	65	0	1	Clonal	0.56	3.94			disease_causing	3.968		Passenger			Neutral	Tolerated
OVA_048_M2	METASTASIS	chr6	96044626	96044626	C	G	MANEA	exonic	onymous S	000035881	360	97	457	0.212254	65	0	65	0	1	Clonal	0.4	3.87			disease_causing	3.968		Passenger			Neutral	Tolerated
OVA_003_M1	METASTASIS	chr5	96215732	96215732	C	T	ERAP2	exonic	ynonymou	000043704	425	184	609	0.302135	57	0	57	0	1	Sub	0.57	1.84			disease_causing	23.4	p.L115F	Passenger	SENGER/OT	damaging	Deleterious	Damaging
OVA_003_M2	METASTASIS	chr5	96215732	96215732	C	T	ERAP2	exonic	ynonymou	000043704	493	114	607	0.187809	57	0	57	0	0.82	Sub	0.46	1.81			disease_causing	23.4	p.L115F	Passenger	SENGER/OT	damaging	Deleterious	Damaging
OVA_003_M3	METASTASIS	chr5	96215732	96215732	C	T	ERAP2	exonic	ynonymou	000043704	645	42	687	0.061135	57	0	57	0	0.64	Sub	0.34	2.67			disease_causing	23.4	p.L115F	Passenger	SENGER/OT	damaging	Deleterious	Damaging
OVA_003_C1	PRIMARY	chr5	96215732	96215732	C	T	ERAP2	exonic	ynonymou	000043704	350	307	657	0.467275	57	0	57	0	1	Clonal	0.86	1.81			disease_causing	23.4	p.L115F	Passenger	SENGER/OT	damaging	Deleterious	Damaging
OVA_003_C2	PRIMARY	chr5	96215732	96215732	C	T	ERAP2	exonic	ynonymou	000043704	346	281	627	0.448166	57	0	57	0	1	Clonal	0.7	3.61			disease_causing	23.4	p.L115F	Passenger	SENGER/OT	damaging	Deleterious	Damaging
OVA_003_C3	PRIMARY	chr5	96215732	96215732	C	T	ERAP2	exonic	ynonymou	000043704	391	280	671	0.417288	57	0	57	0	1	Clonal	0.82	1.81			disease_causing	23.4	p.L115F	Passenger	SENGER/OT	damaging	Deleterious	Damaging
OVA_003_C3	PRIMARY	chr6	97058437	97058437	G	C	FHL5	intronic	NA	NA	568	116	684	0.169591	31	0	31	0	0.41	Sub	0.82	1.81			polymorphism	4.777						
OVA_048_C3	PRIMARY	chr8	97247651	97247651	T	C	UQCRB	intronic	NA	NA	341	146	487	0.299795	140	0	140	0	1	Clonal	0.74	1.8			polymorphism	8.289						
OVA_048_M1	METASTASIS	chr3	97454908	97454908	A	C	EPHA6	exonic	ynonymou	349A,ENSG	354	239	593	0.403035	38	0	38	0	1	Clonal	0.56	3.94			disease_causing	30	p.D1025A	Passenger	CANCER	damaging	Deleterious	Damaging
OVA_048_M2	METASTASIS	chr3	97454908	97454908	A	C	EPHA6	exonic	ynonymou	349A,ENSG	388	191	579	0.329879	38	0	38	0	1	Clonal	0.4	3.87			disease_causing	30	p.D1025A	Passenger	CANCER	damaging	Deleterious	Damaging
OVA_048_M3	METASTASIS	chr3	97454908	97454908	A	C	EPHA6	exonic	ynonymou	349A,ENSG	338	233	571	0.408056	38	0	38	0	1	Clonal	0.59	3.39			disease_causing	30	p.D1025A	Passenger	CANCER	damaging	Deleterious	Damaging
OVA_047_M1	METASTASIS	chr8	98711981	98711981	G	T	MTDH	splicing	NA	NA	428	260	688	0.377907	35	0	35	0	1	Clonal	0.47	2.01			disease_causing	33		Passenger				
OVA_047_M2	METASTASIS	chr8	98711981	98711981	G	T	MTDH	splicing	NA	NA	445	260	705	0.368794	35	0	35	0	1	Clonal	0.44	2.01			disease_causing	33		Passenger				
OVA_047_M3	METASTASIS	chr8	98711981	98711981	G	T	MTDH	splicing	NA	NA	340	189	529	0.357278	35	0	35	0	1	Clonal	0.6	3.02			disease_causing	33		Passenger				
OVA_378_M1	METASTASIS	chr13	99042233	99042233	A	-	FARP1	exonic	eshift dele	000376586	274	161	435	0.370115	50	0	50		1	Clonal	0.72	2.57					p.E293Dfs	Passenger			NA	NA
OVA_378_M2	METASTASIS	chr13	99042233	99042233	A	-	FARP1	exonic	eshift dele	000376586	257	181	438	0.413242	50	0	50		0.97	Clonal	0.85	2.57					p.E293Dfs	Passenger			NA	NA
OVA_378_M3	METASTASIS	chr13	99042233	99042233	A	-	FARP1	exonic	eshift dele	000376586	457	227	684	0.331871	50	0	50		0.99	Clonal	0.67	2.57					p.E293Dfs	Passenger			NA	NA
OVA_378_C1	PRIMARY	chr13	99042233	99042233	A	-	FARP1	exonic	eshift dele	000376586	399	202	601	0.336106	50	0	50		1	Clonal	0.59	2.6					p.E293Dfs	Passenger			NA	NA
OVA_378_C2	PRIMARY	chr13	99042233	99042233	A	-	FARP1	exonic	eshift dele	000376586	376	146	522	0.279693	50	0	50		0.99	Clonal	0.56	2.59					p.E293Dfs	Passenger			NA	NA
OVA_378_C3	PRIMARY	chr13	99042233	99042233	A	-	FARP1	exonic	eshift dele	000376586	342	131	473	0.276956	50	0	50		1	Clonal	0.51	2.6					p.E293Dfs	Passenger			NA	NA
OVA_048_M1	METASTASIS	chr8	99042813	99042813	G	C	MATN2	exonic	ynonymou	000524308	487	136	623	0.218299	42	0	42	0	1	Clonal	0.56	3.94	Yes	No	polymorphism	16.41	p.S827T	Passenger	SENGER/OT	benign	Neutral	Tolerated
OVA_048_M2	METASTASIS	chr8	99042813	99042813	G	C	MATN2	exonic	ynonymou	000524308	510	102	612	0.166667	42	0	42	0	1	Clonal	0.4	3.87	Yes	No	polymorphism	16.41	p.S827T	Passenger	SENGER/OT	benign	Neutral	Tolerated
OVA_048_M3	METASTASIS	chr8	99042813	99042813	G	C	MATN2	exonic	ynonymou	000524308	418	174	592	0.293919	42	0	42	0	1	Clonal	0.59	3.39	Yes	No	polymorphism	16.41	p.S827T	Passenger	SENGER/OT	benign	Neutral	Tolerated
OVA_365_M1	METASTASIS	chr8	99101547	99101547	C	T	C8orf47	exonic	ynonymou	000003185	317	87	404	0.215347	72	0	72	0	0.94	Clonal	0.85	1.87			polymorphism	16.49	p.T101I	Passenger	SENGER/OT	damaging	Deleterious	Damaging
OVA_365_M2	METASTASIS	chr8	99101547	99101547	C	T	C8orf47	exonic	ynonymou	000003185	181	28	209	0.133971	72	0	72	0	0.93	Clonal	0.4	1.81			polymorphism	16.49	p.T101I	Passenger	SENGER/OT	damaging	Deleterious	Damaging
OVA_365_M3	METASTASIS	chr8	99101547	99101547	C	T	C8orf47	exonic	ynonymou	000003185	302	86	388	0.221649	72	0	72	0	0.99	Clonal	0.82	1.89			polymorphism	16.49	p.T101I	Passenger	SENGER/OT	damaging	Deleterious	Damaging
OVA_365_C1	Contralateral ovary	chr8	99101547	99101547	C	T	C8orf47	exonic	ynonymou	000003185	150	29	179	0.162011	72	0	72	0	0.94	Clonal	0.42	1.83			polymorphism	16.49	p.T101I	Passenger	SENGER/OT	damaging	Deleterious	Damaging
OVA_365_C2	PRIMARY	chr8	99101547	99101547	C	T	C8orf47	exonic	ynonymou	000003185	226	82	308	0.266234	72	0	72	0	1	Clonal	0.62	1.92			polymorphism	16.49	p.T101I	Passenger	SENGER/OT	damaging	Deleterious	Damaging
OVA_365_M1	METASTASIS	chr7	99474086	99474086	C	T	OR2AE1	exonic	ynonymou	000031636	473	162	635	0.255118	48	0	48	0	0.86	Sub	0.85	1.87			polymorphism	17.89	p.D191N	Passenger	SENGER/OT	damaging	Deleterious	Tolerated
OVA_365_M2	METASTASIS	chr7	99474086	99474086	C	T	OR2AE1	exonic	ynonymou	000031636	476	79	555	0.142342	48	0	48	0	0.85	Sub	0.4	1.81			polymorphism	17.89	p.D191N	Passenger	SENGER/OT	damaging	Deleterious	Tolerated
OVA_365_M3	METASTASIS	chr7	99474086	99474086	C	T	OR2AE1	exonic	ynonymou	000031636	464	182	646	0.281734	48	0	48	0	0.97	Sub	0.82	1.89			polymorphism	17.89	p.D191N	Passenger	SENGER/OT	damaging	Deleterious	Tolerated
OVA_048_M1	METASTASIS	chr3	99514530	99514530	G	T	COL8A1	exonic	onymous S	595V,ENSG	365	117	482	0.242739	56	0	56	0	1	Clonal	0.56	3.94			disease_causing	6.784		Passenger			Neutral	Tolerated
OVA_048_M2	METASTASIS	chr3	99514530	99514530	G	T	COL8A1	exonic	onymous S	595V,ENSG	444	75	519	0.144509	56	0	56	0	1	Clonal	0.4	3.87			disease_causing	6.784		Passenger			Neutral	Tolerated
OVA_047_M1	METASTASIS	chr9	99581474	99581474	T	A	ZNF782	exonic	onymous S	145G,ENSG	612	108	720	0.15	48	0	48	0	0.78	Sub	0.47	2.01			polymorphism	0.83		Passenger			Neutral	Tolerated
OVA_047_M2	METASTASIS	chr9	99581474	99581474	T	A	ZNF782	exonic	onymous S	145G,ENSG	601	93	694	0.134006	48	0	48	0	0.74	Sub	0.44	2.01			polymorphism	0.83		Passenger			Neutral	Tolerated
OVA_047_M3	METASTASIS	chr9	99581474	99581474	T	A	ZNF782	exonic	onymous S	145G,ENSG	492	92	584	0.157534	48	0	48	0	0.84	Sub	0.6	3.02			polymorphism	0.83		Passenger			Neutral	Tolerated
OVA_047_M1	METASTASIS	chr15	99671814	99671814	T	A	SYNM	exonic	onymous S	000328642	413	114	527	0.216319	71	0	71	0	1	Clonal	0.47	2.01			polymorphism	1.288		Passenger			Neutral	Tolerated
OVA_047_M2	METASTASIS	chr15	99671814	99671814	T	A	SYNM	exonic	onymous S	000328642	490	94	584	0.160959	71	0	71	0	0.89	Clonal	0.44	2.01			polymorphism	1.288		Passenger			Neutral	Tolerated
OVA_047_M3	METASTASIS	chr15	99671814	99671814	T	A	SYNM	exonic	onymous S	000328642	197	48	245	0.195918	71	0	71	0	1	Clonal	0.6	3.02			polymorphism	1.288		Passenger			Neutral	Tolerated
OVA_013_M1	METASTASIS	chr7	1E+08	1E+08	C	T	TSC22D4	exonic	unknown	UNKNOWN	65	64	129	0.496124	70	1	71	0.014085	1	Clonal	0.67	3.06			polymorphism	11.27					Neutral	Tolerated
OVA_013_C1	PRIMARY	chr7	1E+08	1E+08	C	T	TSC22D4	exonic	unknown	UNKNOWN	92	70	162	0.432099	70	1	71	0.014085	1	Clonal	0.72	3.1			polymorphism	11.27					Neutral	Tolerated
OVA_013_C2	PRIMARY	chr7	1E+08	1E+08	C	T	TSC22D4	exonic	unknown	UNKNOWN	83	75	158	0.474684	70	1	71	0.014085	1	Clonal	0.87	3.05			polymorphism	11.27					Neutral	Tolerated
OVA_013_C3	PRIMARY	chr7	1E+08	1E+08	C	T	TSC22D4	exonic	unknown	UNKNOWN	88	105	193	0.544041	70	1	71	0.014085	1	Clonal	0.91	3.01			polymorphism	11.27					Neutral	Tolerated
OVA_365_M1	METASTASIS	chr7	1E+08	1E+08	C	T	LRCH4	intronic	NA	NA	32	6	38	0.157895	86	0	86	0	0.53	Sub	0.85	1.87			polymorphism	0.152						
OVA_365_M2	METASTASIS	chr7	1E+08	1E+08	C	T	LRCH4	intronic	NA	NA	16	2	18	0.111111	86	0	86	0	0.66	Sub	0.4	1.81			polymorphism	0.152						
OVA_365_M3	METASTASIS	chr7	1E+08	1E+08	C	T	LRCH4	intronic	NA	NA	11	6	17	0.352941	86	0	86	0	1	Sub	0.82	1.89			polymorphism	0.152						
OVA_365_C1	Contralateral ovary	chr7	1E+08	1E+08	C	T	LRCH4	intronic	NA	NA	13	3	16	0.1875	86	0	86	0	1	Clonal	0.42	1.83			polymorphism	0.152						
OVA_365_C2	PRIMARY	chr7	1E+08	1E+08	C	T	LRCH4	intronic	NA	NA	22	11	33	0.333333	86	0	86	0	1	Clonal	0.62	1.92			polymorphism	0.152						
OVA_365_M3	METASTASIS	chr7	1E+08	1E+08	C	T	PCOLCE	exonic	onymous S	000022306	477	29	506	0.057312	65	0	65	0	0.2	Sub	0.82	1.89			polymorphism	10.12		Passenger			Neutral	Tolerated
OVA_365_M3	METASTASIS	chrX	1E+08	1E+08	G	C	ARL13A	intronic	NA	NA	509	34	543	0.062615	126	0	126	0	0.09	Sub	0.82	1.89			polymorphism	3.688						
OVA_048_C1	PRIMARY	chr7	1.01E+08	1.01E+08	T	A	MUC17	exonic	onymous S	3866I,ENSG	346	84	430	0.195349	42	0	42	0	0.58	Sub	0.67	1.82			polymorphism	0.118		Passenger			Neutral	Tolerated
OVA_048_M1	METASTASIS	chr7	1.01E+08	1.01E+08	G	T	AP1S1	exonic	unknown	UNKNOWN	4	9	13	0.692308	21	0	21	0	1	Clonal	0.56	3.94			disease_causing	20.2					Neutral	Tolerated
OVA_048_M3	METASTASIS	chr7	1.01E+08	1.01E+08	G	T	AP1S1	exonic	unknown	UNKNOWN	10	3	13	0.230769	21	0	21	0	1	Clonal	0.59	3.39			disease_causing	20.2					Neutral	Tolerated
OVA_365_M1	METASTASIS	chr8	1.01E+08	1.01E+08	G	C	VPS13B	intronic	NA	NA	526	134	660	0.20303	36	0	36	0	0.89	Sub	0.85	1.87			disease_causing	18.2						
OVA_365_M2	METASTASIS	chr8	1.01E+08	1.01E+08	G	C	VPS13B	intronic	NA	NA	411	82	493	0.166329	36	0	36	0	1	Clonal	0.4	1.81			disease_causing	18.2						
OVA_365_M3	METASTASIS	chr8	1.01E+08	1.01E+08	G	C	VPS13B	intronic	NA	NA	505	126	631	0.199683	36	0	36	0	0.89	Sub	0.82	1.89			disease_causing	18.2						
OVA_013_C2	PRIMARY	chr11	1.01E+08	1.01E+08	G	A	ARHGAP42	exonic	ynonymou	785H,ENSG	616	61	677	0.090103	52	0	52	0	0.48	Sub	0.87	3.05			disease_causing	34	p.R819H	Passenger	SENGER/OT	damaging	Deleterious	Damaging
OVA_048_M1	METASTASIS	chr1	1.01E+08	1.01E+08	T	C	CDC14A	intronic	NA	NA	309	232	541	0.428835	29	0	29	0	1	Clonal	0.56	3.94			polymorphism	7.427						
OVA_048_M2	METASTASIS	chr1	1.01E+08	1.01E+08	T	C	CDC14A	intronic	NA	NA	338	213	551	0.38657	29	0	29	0	1	Clonal	0.4	3.87			polymorphism	7.427						
OVA_048_M3	METASTASIS	chr1	1.01E+08	1.01E+08	T	C	CDC14A	intronic	NA	NA	275	254	529	0.480151	29	0	29	0	1	Clonal	0.59	3.39			polymorphism	7.427						
OVA_378_C3	PRIMARY	chr6	1.01E+08	1.01E+08	A	G	SIM1	exonic	ynonymou	360P,ENSG	221	62	283	0.219081	72	0	72	0	0.86	Sub	0.51	2.6			disease_causing	21.6	p.S360P	Passenger	SENGER/OT	benign	Neutral	Tolerated
OVA_048_M1	METASTASIS	chrX	1.01E+08	1.01E+08	T	G	ARMCX2	exonic	ynonymou	000032876	326	172	498	0.345382	95	2	97	0.020619	1	Clonal	0.56	3.94			polymorphism	16.66	p.E303D	Passenger	SENGER/OT	benign	Neutral	Tolerated
OVA_048_M2	METASTASIS	chrX	1.01E+08	1.01E+08	T	G	ARMCX2	exonic	ynonymou	000032876	378	148	526	0.281369	95	2	97	0.020619	1	Clonal	0.4	3.87			polymorphism	16.66	p.E303D	Passenger	SENGER/OT	benign	Neutral	Tolerated
OVA_048_M3	METASTASIS	chrX	1.01E+08	1.01E+08	T	G	ARMCX2	exonic	ynonymou	000032876	381	128	509	0.251473	95	2	97	0.020619	1	Clonal	0.59	3.39			polymorphism	16.66	p.E303D	Passenger	SENGER/OT	benign	Neutral	Tolerated

Sample	Type	Chr	Start	End	Ref	Alt	Gene	unc.ensGen	icFunc.ens	hange.ens(	t_ref	t_alt	t_total	t_VAF	n_ref	n_alt	n_total	normal_VA	ncer_cell_f	on-based C	purity	ploidy	Cancer Gene	Driver Gene	Mutation Taster	ADD(PHRE	otein_chan	CHASM	ATHMM_Pr	OLYPHEN-	REDICTION	ICTION (cutoff=0.05)
OVA_048_M1	METASTASIS	chr14	1.01E+08	1.01E+08	T	A	BEGAIN	exonic	ynonymou	000044307	9	3	12	0.25	11	0	11	0	1	Clonal	0.56	3.94			disease_causing	22.6	p.D543V	Passenger	no weights	benign	Deleteriou	Damaging
OVA_048_M2	METASTASIS	chr14	1.01E+08	1.01E+08	T	A	BEGAIN	exonic	ynonymou	000044307	22	7	29	0.241379	11	0	11	0	1	Clonal	0.4	3.87			disease_causing	22.6	p.D543V	Passenger	no weights	benign	Deleteriou	Damaging
OVA_048_C2	PRIMARY	chr14	1.01E+08	1.01E+08	T	A	BEGAIN	exonic	ynonymou	000044307	8	3	11	0.272727	11	0	11	0	0.76	Clonal	0.72	1.85			disease_causing	22.6	p.D543V	Passenger	no weights	benign	Deleteriou	Damaging
OVA_048_C3	PRIMARY	chr14	1.01E+08	1.01E+08	T	A	BEGAIN	exonic	ynonymou	000044307	9	4	13	0.307692	11	0	11	0	0.83	Clonal	0.74	1.8			disease_causing	22.6	p.D543V	Passenger	no weights	benign	Deleteriou	Damaging
OVA_048_C2	PRIMARY	chr12	1.01E+08	1.01E+08	A	T	GAS2L3	exonic	ionymous S	240G,ENSG	394	122	516	0.236434	33	0	33	0	0.89	Clonal	0.72	1.85			disease_causing	8.029		Passenger			Neutral	Tolerated
OVA_365_M1	METASTASIS	chr10	1.01E+08	1.01E+08	C	G	CNNM1	intronic	NA	NA	211	173	384	0.450521	11	0	11	0	1	Clonal	0.85	1.87			polymorphism	1.538						
OVA_365_M2	METASTASIS	chr10	1.01E+08	1.01E+08	C	G	CNNM1	intronic	NA	NA	126	26	152	0.171053	11	0	11	0	0.85	Clonal	0.4	1.81			polymorphism	1.538						
OVA_365_M3	METASTASIS	chr10	1.01E+08	1.01E+08	C	G	CNNM1	intronic	NA	NA	168	129	297	0.434343	11	0	11	0	1	Clonal	0.82	1.89			polymorphism	1.538						
OVA_365_C2	PRIMARY	chr10	1.01E+08	1.01E+08	C	G	CNNM1	intronic	NA	NA	206	84	290	0.289655	11	0	11	0	0.94	Clonal	0.62	1.92			polymorphism	1.538						
OVA_378_M1	METASTASIS	chr15	1.02E+08	1.02E+08	C	T	LRRK1	:RNA_intro	NA	NA	324	244	568	0.429577	43	0	43	0	1	Clonal	0.72	2.57			polymorphism	6.547						
OVA_378_M2	METASTASIS	chr15	1.02E+08	1.02E+08	C	T	LRRK1	:RNA_intro	NA	NA	300	248	548	0.452555	43	0	43	0	1	Clonal	0.85	2.57			polymorphism	6.547						
OVA_378_M3	METASTASIS	chr15	1.02E+08	1.02E+08	C	T	LRRK1	:RNA_intro	NA	NA	358	248	606	0.409241	43	0	43	0	1	Clonal	0.67	2.57			polymorphism	6.547						
OVA_378_C1	PRIMARY	chr15	1.02E+08	1.02E+08	C	T	LRRK1	:RNA_intro	NA	NA	1460	30	1491	0.020121	43	0	43	0	0.07	Sub	0.72	2.57			polymorphism	6.547						
OVA_047_M1	METASTASIS	chr10	1.02E+08	1.02E+08	G	T	ABCC2	UTR3	NA	NA	161	65	226	0.287611	57	0	57	0	0.92	Clonal	0.47	2.01			polymorphism	0.61						
OVA_047_M2	METASTASIS	chr10	1.02E+08	1.02E+08	G	T	ABCC2	UTR3	NA	NA	164	79	243	0.325103	57	0	57	0	1	Clonal	0.44	2.01			polymorphism	0.61						
OVA_047_M3	METASTASIS	chr10	1.02E+08	1.02E+08	G	T	ABCC2	UTR3	NA	NA	87	48	135	0.355556	57	0	57	0	1	Clonal	0.6	3.02			polymorphism	0.61						
OVA_003_C2	PRIMARY	chr15	1.02E+08	1.02E+08	T	G	SNRPA1	intronic	NA	NA	489	50	539	0.092764	38	0	38	0	0.36	Sub	0.7	3.61			disease_causing	7.233						
OVA_378_M1	METASTASIS	chr13	1.02E+08	1.02E+08	T	C	NALCN	exonic	ynonymou	I486V,ENSG	252	42	294	0.142857	34	0	34	0	0.4	Sub	0.72	2.57			disease_causing	23.7	p.I486V	Passenger	SENGER/OT	damaging	Neutral	Tolerated
OVA_378_M2	METASTASIS	chr13	1.02E+08	1.02E+08	T	C	NALCN	exonic	ynonymou	I486V,ENSG	254	18	272	0.066176	34	0	34	0	0.16	Sub	0.85	2.57			disease_causing	23.7	p.I486V	Passenger	SENGER/OT	damaging	Neutral	Tolerated
OVA_378_M3	METASTASIS	chr13	1.02E+08	1.02E+08	T	C	NALCN	exonic	ynonymou	I486V,ENSG	426	39	465	0.083871	34	0	34	0	0.25	Sub	0.67	2.57			disease_causing	23.7	p.I486V	Passenger	SENGER/OT	damaging	Neutral	Tolerated
OVA_013_C1	PRIMARY	chr15	1.02E+08	1.02E+08	C	T	TARSL2	exonic	ionymous S	000335968	527	136	663	0.205128	107	0	107	0	0.98	Sub	0.72	3.1			disease_causing	19.38		Passenger			Neutral	Tolerated
OVA_013_C2	PRIMARY	chr15	1.02E+08	1.02E+08	C	T	TARSL2	exonic	ionymous S	000335968	514	123	637	0.193093	107	0	107	0	0.83	Sub	0.87	3.05			disease_causing	19.38		Passenger			Neutral	Tolerated
OVA_013_C3	PRIMARY	chr15	1.02E+08	1.02E+08	C	T	TARSL2	exonic	ionymous S	000335968	544	137	681	0.201175	107	0	107	0	0.64	Sub	0.91	3.01			disease_causing	19.38		Passenger			Neutral	Tolerated
OVA_048_C1	PRIMARY	chr12	1.03E+08	1.03E+08	G	C	PARPBP	exonic	ynonymou	222Q,ENSG	323	116	439	0.264237	64	0	64	0	1	Clonal	0.67	1.82			disease_causing	26.8	p.E380Q	Passenger	SENGER/OT	damaging	Neutral	Damaging
OVA_048_C3	PRIMARY	chr12	1.03E+08	1.03E+08	G	C	PARPBP	exonic	ynonymou	222Q,ENSG	386	135	521	0.259117	64	0	64	0	0.96	Clonal	0.74	1.8			disease_causing	26.8	p.E380Q	Passenger	SENGER/OT	damaging	Neutral	Damaging
OVA_013_M1	METASTASIS	chr11	1.03E+08	1.03E+08	T	A	MMP12	cRNA_exon	NA	NA	521	96	617	0.155592	41	0	41	0	0.93	Clonal	0.67	3.06				1.629	p.H112L	Passenger	SENGER/OT	benign		
OVA_048_M1	METASTASIS	chr10	1.03E+08	1.03E+08	A	C	MRPL43	exonic	ynonymou	110W,ENSG	403	147	550	0.267273	167	1	168	0.005952	1	Clonal	0.56	3.94			disease_causing	26.2	p.L110W	Passenger	no weights	damaging	Deleteriou	Damaging
OVA_048_M2	METASTASIS	chr10	1.03E+08	1.03E+08	A	C	MRPL43	exonic	ynonymou	110W,ENSG	431	108	539	0.200371	167	1	168	0.005952	1	Clonal	0.4	3.87			disease_causing	26.2	p.L110W	Passenger	no weights	damaging	Deleteriou	Damaging
OVA_048_M3	METASTASIS	chr10	1.03E+08	1.03E+08	A	C	MRPL43	exonic	ynonymou	110W,ENSG	373	157	530	0.296226	167	1	168	0.005952	1	Clonal	0.59	3.39			disease_causing	26.2	p.L110W	Passenger	no weights	damaging	Deleteriou	Damaging
OVA_013_M1	METASTASIS	chr11	1.03E+08	1.03E+08	C	G	DYNC2H1	exonic	ynonymou	0375735:ex	340	315	655	0.480916	48	0	48	0	1	Clonal	0.67	3.06			polymorphism	14.95	p.T3811S	Passenger	SENGER/OT	benign	Neutral	Tolerated
OVA_013_C1	PRIMARY	chr11	1.03E+08	1.03E+08	C	G	DYNC2H1	exonic	ynonymou	0375735:ex	292	369	661	0.558245	48	0	48	0	1	Clonal	0.72	3.1			polymorphism	14.95	p.T3811S	Passenger	SENGER/OT	benign	Neutral	Tolerated
OVA_013_C2	PRIMARY	chr11	1.03E+08	1.03E+08	C	G	DYNC2H1	exonic	ynonymou	0375735:ex	268	350	618	0.566343	48	0	48	0	1	Clonal	0.87	3.05			polymorphism	14.95	p.T3811S	Passenger	SENGER/OT	benign	Neutral	Tolerated
OVA_013_C3	PRIMARY	chr11	1.03E+08	1.03E+08	C	G	DYNC2H1	exonic	ynonymou	0375735:ex	304	383	687	0.557496	48	0	48	0	1	Clonal	0.91	3.01			polymorphism	14.95	p.T3811S	Passenger	SENGER/OT	benign	Neutral	Tolerated
OVA_048_M1	METASTASIS	chr12	1.03E+08	1.03E+08	G	A	PAH	exonic	ionymous S	.L86L,ENSG	366	322	688	0.468023	28	0	28	0	1	Clonal	0.56	3.94			disease_causing	9.584		Passenger			Neutral	Tolerated
OVA_048_M2	METASTASIS	chr12	1.03E+08	1.03E+08	G	A	PAH	exonic	ionymous S	.L86L,ENSG	398	265	663	0.399698	28	0	28	0	1	Clonal	0.4	3.87			disease_causing	9.584		Passenger			Neutral	Tolerated
OVA_048_M3	METASTASIS	chr12	1.03E+08	1.03E+08	G	A	PAH	exonic	ionymous S	.L86L,ENSG	286	380	666	0.570571	28	0	28	0	1	Clonal	0.59	3.39			disease_causing	9.584		Passenger			Neutral	Tolerated
OVA_048_C1	PRIMARY	chr12	1.03E+08	1.03E+08	G	A	PAH	exonic	ionymous S	.L86L,ENSG	208	258	466	0.553648	28	0	28	0	1	Clonal	0.67	1.82			disease_causing	9.584		Passenger			Neutral	Tolerated
OVA_048_C2	PRIMARY	chr12	1.03E+08	1.03E+08	G	A	PAH	exonic	ionymous S	.L86L,ENSG	278	346	624	0.554487	28	0	28	0	1	Clonal	0.72	1.85			disease_causing	9.584		Passenger			Neutral	Tolerated
OVA_048_C3	PRIMARY	chr12	1.03E+08	1.03E+08	G	A	PAH	exonic	ionymous S	.L86L,ENSG	230	239	469	0.509595	28	0	28	0	1	Clonal	0.74	1.8			disease_causing	9.584		Passenger			Neutral	Tolerated
OVA_003_M1	METASTASIS	chr13	1.03E+08	1.03E+08	C	G	TPP2	exonic	ynonymou	S915C,ENSG	438	167	605	0.276033	65	0	65	0	0.98	Sub	0.57	1.84			disease_causing	29.2	p.S915C	Passenger	no weights	damaging	Deleteriou	Damaging
OVA_003_M2	METASTASIS	chr13	1.03E+08	1.03E+08	C	G	TPP2	exonic	ynonymou	S915C,ENSG	495	139	634	0.219243	65	0	65	0	0.96	Sub	0.46	1.81			disease_causing	29.2	p.S915C	Passenger	no weights	damaging	Deleteriou	Damaging
OVA_003_M3	METASTASIS	chr13	1.03E+08	1.03E+08	C	G	TPP2	exonic	ynonymou	S915C,ENSG	672	48	720	0.066667	65	0	65	0	0.64	Sub	0.34	2.67			disease_causing	29.2	p.S915C	Passenger	no weights	damaging	Deleteriou	Damaging
OVA_003_C1	PRIMARY	chr13	1.03E+08	1.03E+08	C	G	TPP2	exonic	ynonymou	S915C,ENSG	386	284	670	0.423881	65	0	65	0	0.98	Clonal	0.86	1.81			disease_causing	29.2	p.S915C	Passenger	no weights	damaging	Deleteriou	Damaging
OVA_003_C2	PRIMARY	chr13	1.03E+08	1.03E+08	C	G	TPP2	exonic	ynonymou	S915C,ENSG	380	295	675	0.437037	65	0	65	0	1	Clonal	0.7	3.61			disease_causing	29.2	p.S915C	Passenger	no weights	damaging	Deleteriou	Damaging
OVA_003_C3	PRIMARY	chr13	1.03E+08	1.03E+08	C	G	TPP2	exonic	ynonymou	S915C,ENSG	405	273	678	0.402655	65	0	65	0	0.98	Clonal	0.82	1.81			disease_causing	29.2	p.S915C	Passenger	no weights	damaging	Deleteriou	Damaging
OVA_048_M1	METASTASIS	chrX	1.03E+08	1.03E+08	T	A	ESX1	exonic	ynonymou	000037258	189	109	298	0.365772	25	0	25	0	1	Clonal	0.56	3.94			disease_causing	24.2	p.N189Y	Passenger	CANCER	damaging	Deleteriou	Damaging
OVA_048_M2	METASTASIS	chrX	1.03E+08	1.03E+08	T	A	ESX1	exonic	ynonymou	000037258	202	82	284	0.288732	25	0	25	0	1	Clonal	0.4	3.87			disease_causing	24.2	p.N189Y	Passenger	CANCER	damaging	Deleteriou	Damaging
OVA_048_M3	METASTASIS	chrX	1.03E+08	1.03E+08	T	A	ESX1	exonic	ynonymou	000037258	197	106	303	0.349835	25	0	25	0	1	Clonal	0.59	3.39			disease_causing	24.2	p.N189Y	Passenger	CANCER	damaging	Deleteriou	Damaging
OVA_048_C1	PRIMARY	chrX	1.03E+08	1.03E+08	T	A	ESX1	exonic	ynonymou	000037258	70	42	112	0.375	25	0	25	0	1	Clonal	0.67	1.82			disease_causing	24.2	p.N189Y	Passenger	CANCER	damaging	Deleteriou	Damaging
OVA_048_C2	PRIMARY	chrX	1.03E+08	1.03E+08	T	A	ESX1	exonic	ynonymou	000037258	180	116	296	0.391892	25	0	25	0	1	Clonal	0.72	1.85			disease_causing	24.2	p.N189Y	Passenger	CANCER	damaging	Deleteriou	Damaging
OVA_048_C3	PRIMARY	chrX	1.03E+08	1.03E+08	T	A	ESX1	exonic	ynonymou	000037258	96	54	150	0.36	25	0	25	0	0.97	Clonal	0.74	1.8			disease_causing	24.2	p.N189Y	Passenger	CANCER	damaging	Deleteriou	Damaging
OVA_048_M3	METASTASIS	chr14	1.04E+08	1.04E+08	C	A	TNFAIP2	intronic	NA	NA	431	36	467	0.077088	70	0	70	0	0.68	Sub	0.59	3.39			disease_causing	6.661						
OVA_047_M1	METASTASIS	chr7	1.04E+08	1.04E+08	C	T	ORC5	exonic	ynonymou	000044745	417	185	602	0.307309	68	0	68	0	0.99	Clonal	0.47	2.01			disease_causing	26.3	p.R166H	Passenger	SENGER/OT	damaging	Neutral	Tolerated
OVA_047_M2	METASTASIS	chr7	1.04E+08	1.04E+08	C	T	ORC5	exonic	ynonymou	000044745	451	171	622	0.27492	68	0	68	0	0.96	Clonal	0.44	2.01			disease_causing	26.3	p.R166H	Passenger	SENGER/OT	damaging	Neutral	Tolerated
OVA_047_M3	METASTASIS	chr7	1.04E+08	1.04E+08	C	T	ORC5	exonic	ynonymou	000044745	239	101	340	0.297059	68	0	68	0	0.99	Clonal	0.6	3.02			disease_causing	26.3	p.R166H	Passenger	SENGER/OT	damaging	Neutral	Tolerated
OVA_047_C1	PRIMARY	chr7	1.04E+08	1.04E+08	C	T	ORC5	exonic	ynonymou	000044745	548	85	633	0.134281	68	0	68	0	0.45	Sub	0.6	2.91			disease_causing	26.3	p.R166H	Passenger	SENGER/OT	damaging	Neutral	Tolerated
OVA_047_C2	PRIMARY	chr7	1.04E+08	1.04E+08	C	T	ORC5	exonic	ynonymou	000044745	503	139	642	0.216511	68	0	68	0	0.68	Sub	0.64	2.76			disease_causing	26.3	p.R166H	Passenger	SENGER/OT	damaging	Neutral	Tolerated
OVA_047_C3	Contralateral ovary	chr7	1.04E+08	1.04E+08	C	T	ORC5	exonic	ynonymou	000044745	485	130	615	0.211382	68	0	68	0	0.92	Clonal	0.6	2.94			disease_causing	26.3	p.R166H	Passenger	SENGER/OT	damaging	Neutral	Tolerated
OVA_048_M1	METASTASIS	chr8	1.04E+08	1.04E+08	A	G	ATP6V1C1	exonic	ionymous S	.92A,ENSG(	423	152	575	0.264348	47	1	48	0.020833	1	Clonal	0.56	3.94			disease_causing	11.17		Passenger			Neutral	Tolerated
OVA_048_M2	METASTASIS	chr8	1.04E+08	1.04E+08	A	G	ATP6V1C1	exonic	ionymous S	.92A,ENSG(	450	121	571	0.211909	47	1	48	0.020833	1	Clonal	0.4	3.87			disease_causing	11.17		Passenger			Neutral	Tolerated
OVA_048_M3	METASTASIS	chr8	1.04E+08	1.04E+08	A	G	ATP6V1C1	exonic	ionymous S	.92A,ENSG(	482	75	557	0.13465	47	1	48	0.020833	1	Clonal	0.59	3.39			disease_causing	11.17		Passenger			Neutral	Tolerated
OVA_048_C1	PRIMARY	chr8	1.04E+08	1.04E+08	A	G	ATP6V1C1	exonic	ionymous S	.92A,ENSG(	338	92	430	0.213953	47	1	48	0.020833	0.85	Clonal	0.67	1.82			disease_causing	11.17		Passenger			Neutral	Tolerated
OVA_048_C2	PRIMARY	chr8	1.04E+08	1.04E+08	A	G	ATP6V1C1	exonic	ionymous S	.92A,ENSG(	384	165	549	0.300546	47	1	48	0.020833	1	Clonal	0.72	1.85			disease_causing	11.17		Passenger			Neutral	Tolerated
OVA_048_C3	PRIMARY	chr8	1.04E+08	1.04E+08	A	G	ATP6V1C1	exonic	ionymous S	.92A,ENSG(	372	121	493	0.245436	47	1	48	0.020833	0.91	Clonal	0.74	1.8			disease_causing	11.17		Passenger			Neutral	Tolerated
OVA_378_C1	PRIMARY	chr10	1.04E+08	1.04E+08	C	A	PSD	exonic	ynonymou	/228F,ENSG	244	78	322	0.242236	63	0	63	0	0.82	Sub	0.59	2.6			polymorphism	22.2	p.V228F	Passenger	SENGER/OT	damaging	Neutral	Damaging
OVA_378_C2	PRIMARY	chr10	1.04E+08	1.04E+08	C	A	PSD	exonic	ynonymou	/228F,ENSG	159	66	225	0.293333	63	0	63	0	1	Sub	0.56	2.59			polymorphism	22.2	p.V228F	Passenger	SENGER/OT	damaging	Neutral	Damaging
OVA_378_C3	PRIMARY	chr10	1.04E+08	1.04E+08	C	A	PSD	exonic	ynonymou	/228F,ENSG	173	41	214	0.191589	63	0	63	0	0.75	Sub	0.51	2.6			polymorphism	22.2	p.V228F	Passenger	SENGER/OT	damaging	Neutral	Damaging
OVA_013_M1	METASTASIS	chr9	1.04E+08	1.04E+08	G	T	TMEM246	exonic	ynonymou	000037484	267	75	342	0.219298	99	0	99	0	0.87	Clonal	0.67	3.06			polymorphism	17.59	p.H357Q	Passenger	no weights	benign	Neutral	Tolerated
OVA_013_C1	PRIMARY	chr9	1.04E+08	1.04E+08	G	T	TMEM246	exonic	ynonymou	000037484	315	126	441	0.285714	99	0	99	0	1	Clonal	0.72	3.1			polymorphism	17.59	p.H357Q	Passenger	no weights	benign	Neutral	Tolerated
OVA_013_C2	PRIMARY	chr9	1.04E+08	1.04E+08	G	T	TMEM246	exonic	ynonymou	000037484	338	129	467	0.276231	99	0	99	0	0.91	Clonal	0.87	3.05			polymorphism	17.59	p.H357Q	Passenger	no weights	benign	Neutral	Tolerated
OVA_013_C3	PRIMARY	chr9	1.04E+08	1.04E+08	G	T	TMEM246	exonic	ynonymou	000037484	380	168	548	0.306569	99	0	99	0	0.98	Clonal	0.91	3.01			polymorphism	17.59	p.H357Q	Passenger	no weights	benign	Neutral	Tolerated
OVA_047_M1	METASTASIS	chr12	1.04E+08	1.04E+08	G	T	GNN	cRNA_exon	NA	NA	395	198	593	0.333895	50	0	50	0	1	Clonal	0.47	2.01				2.939						
OVA_047_M2	METASTASIS	chr12	1.04E+08	1.04E+08	G	T	GNN	cRNA_exon	NA	NA	406	179	585	0.305983	50	0	50	0	1	Clonal	0.44	2.01				2.939						
OVA_047_M3	METASTASIS	chr12	1.04E+08	1.04E+08	G	T	GNN	cRNA_exon	NA	NA	234	101	335	0.301493	50	0	50	0	1	Clonal	0.6	3.02				2.939						
OVA_013_M1	METASTASIS	chr7	1.05E+08	1.05E+08	C	T	KMT2E	exonic	ynonymou	000311117	552	133	685	0.194161	138	1	139	0.007194	0.97	Clonal	0.67	3.06			polymorphism	20.8	p.P908S	Passenger	SENGER/OT	benign	Deleteriou	Damaging
OVA_013_C1	PRIMARY	chr7	1.05E+08	1.05E+08	C	T	KMT2E	exonic	ynonymou	000311117	529	128	657	0.194825	138	1	139	0.007194	0.93	Sub	0.72	3.1			polymorphism	20.8	p.P908S	Passenger	SENGER/OT	benign	Deleteriou	Damaging
OVA_013_C2	PRIMARY	chr7	1.05E+08	1.05E+08	C	T	KMT2E	exonic	ynonymou	000311117	503	126	629	0.200318	138	1	139	0.007194	0.86	Sub	0.87	3.05			polymorphism	20.8	p.P908S	Passenger	SENGER/OT	benign	Deleteriou	Damaging
OVA_013_C3	PRIMARY	chr7	1.05E+08	1.05E+08	C	T	KMT2E	exonic	ynonymou	000311117	524	140	664	0.210843	138	1	139	0.007194	0.88	Sub	0.91	3.01			polymorphism	20.8	p.P908S	Passenger	SENGER/OT	benign	Deleteriou	Damaging
OVA_047_M1	METASTASIS	chr14	1.05E+08	1.05E+08	G	C	C14orf180	UTR3	NA	NA	238	65	303	0.214521	35	0	35	0	1	Clonal	0.47	2.01			polymorphism	0.22						
OVA_047_M2	METASTASIS	chr14	1.05E+08	1.05E+08	G	C	C14orf180	UTR3	NA	NA	279	62	341	0.181818	35	0	35	0	1	Clonal	0.44	2.01			polymorphism	0.22						
OVA_047_M3	METASTASIS	chr14	1.05E+08	1.05E+08	G	C	C14orf180	UTR3	NA	NA	134	28	162	0.17284	35	0	35	0	0.92	Clonal	0.6	3.02			polymorphism	0.22						
OVA_048_M1	METASTASIS	chr10	1.05E+08	1.05E+08	G	C	PDCD11	intronic	NA	NA	588	13	601	0.021631	50	0	50	0	0.1	Sub	0.56	3.94			polymorphism	8.387						
OVA_048_M3	METASTASIS	chr10	1.05E+08	1.05E+08	G	C	PDCD11	intronic	NA	NA	439	121	560	0.216071	50	0	50	0	1	Sub	0.59	3.39			polymorphism	8.387						
OVA_365_C2	PRIMARY	chr14	1.06E+08	1.06E+08	C	A	BTBD6	exonic	ynonymou	000039255	266	96	362	0.265193	96	0	96	0	0.86	Clonal	0.62	1.92			polymorphism	22	p.P266Q	Passenger	SENGER/OT	benign	Neutral	Tolerated
OVA_047_M1	METASTASIS	chr10	1.06E+08	1.06E+08	A	G	COL17A1	intronic	NA	NA	159	51	210	0.242857	38	0	38	0	0.78	Sub	0.47	2.01			polymorphism	7						
OVA_047_M2	METASTASIS	chr10	1.06E+08	1.06E+08	A	G	COL17A1	intronic	NA	NA	197	72	269	0.267658	38	0	38	0	0.94	Sub	0.44	2.01			polymorphism	7						
OVA_047_M3	METASTASIS	chr10	1.06E+08	1.06E+08	A	G	COL17A1	intronic	NA	NA	78	27	105	0.257143	38	0	38	0	0.86	Sub	0.6	3.02			polymorphism	7						
OVA_048_C1	PRIMARY	chr13	1.06E+08	1.06E+08	A	C	DAOA	exonic	ynonymou	.R17S,ENSG	178	78	256	0.304688	25	0	25	0	0.91	Clonal	0.67	1.82			polymorphism	22	p.R17S	Passenger		damaging	Neutral	Damaging
OVA_048_C2	PRIMARY	chr13	1.06E+08	1.06E+08	A	C	DAOA	exonic	ynonymou	.R17S,ENSG	200	132	332	0.39759	25	0	25	0	1	Clonal	0.72	1.85			polymorphism	22	p.R17S	Passenger		damaging	Neutral	Damaging
OVA_048_C3	PRIMARY	chr13	1.06E+08	1.06E+08	A	C	DAOA	exonic	ynonymou	.R17S,ENSG	164	109	273	0.399267	25	0	25	0	1	Clonal	0.74	1.8			polymorphism	22	p.R17S	Passenger		damaging	Neutral	Damaging
OVA_047_C2	PRIMARY	chr14	1.06E+08	1.06E+08	G	A	abParts	exonic	ionymous S	000039055	269	152	421	0.361045	56	0	56	0	1	Clonal	0.64	2.76				4.482						
OVA_378_C2	PRIMARY	chr7	1.07E+08	1.07E+08	T	G	HBP1	exonic	ynonymou	000468410	468	143	611	0.234043	54	0	54	0	1	Clonal	0.56	2.59			disease_causing	22.7	p.S511A	Passenger	CANCER	benign	Neutral	Damaging
OVA_048_M1	METASTASIS	chr10	1.07E+08	1.07E+08	C	A	SORCS3	intronic	NA	NA	192	154	346	0.445087	9	0	9	0	1	Clonal	0.56	3.94			polymorphism	6.996						
OVA_048_M2	METASTASIS	chr10	1.07E+08	1.07E+08	C	A	SORCS3	intronic	NA	NA	256	111	367	0.302452	9	0	9	0	1	Clonal	0.4	3.87			polymorphism	6.996						
OVA_048_M3	METASTASIS	chr10	1.07E+08	1.07E+08	C	A	SORCS3	intronic	NA	NA	184	83	267	0.310861	9	0	9	0	1	Clonal	0.59	3.39			polymorphism	6.996						
OVA_003_M1	METASTASIS	chr14	1.07E+08	1.07E+08	TG	-	abParts	downstrean	NA	NA	992	237	1229	0.19284	47	0	47		0.88	Sub	0.57	1.84										
OVA_003_M2	METASTASIS	chr14	1.07E+08	1.07E+08	TG	-	abParts	downstrean	NA	NA	1358	272	1630	0.166871	47	0	47		0.9	Sub	0.46	1.81										
OVA_003_M3	METASTASIS	chr14	1.07E+08	1.07E+08	TG	-	abParts	downstrean	NA	NA	1478	71	1549	0.045836	47	0	47		0.53	Sub	0.34	2.67										
OVA_003_C1	PRIMARY	chr14	1.07E+08	1.07E+08	TG	-	abParts	downstrean	NA	NA	409	309	718	0.430362	47	0	47		1	Clonal	0.86	1.81										
OVA_003_C2	PRIMARY	chr14	1.07E+08	1.07E+08	TG	-	abParts	downstrean	NA	NA	456	332	788	0.42132	47	0	47		1	Clonal	0.7	3.61										

Sample	Type	Chr	Start	End	Ref	Alt	Gene	unc.ensGen	icFunc.ens	hange.ens	t_ref	t_alt	t_total	t_VAF	n_ref	n_alt	n_total	normal_VA	ncer_cell_f	on-based C	purity	ploidy	Cancer Gene	Driver Gene	Mutation Taster	ADD(PHRE	otein_chan	CHASM	ATHMM_Pr	OLYPHEN-	REDICTION	ICTION (cutoff=0.05)
OVA_003_C3	PRIMARY	chr14	1.07E+08	1.07E+08	TG	-	abParts	downstrean	NA	NA	564	346	910	0.38022	47	0	47		0.92	Clonal	0.82	1.81										
OVA_003_M1	METASTASIS	chr7	1.07E+08	1.07E+08	A	T	COG5	exonic	ynonymou	T000003470	37	12	49	0.244898	27	0	27	0	0.87	Clonal	0.57	1.84			polymorphism	14.92	p.S11T	Passenger	SENGER/OT	benign	Neutral	Tolerated
OVA_003_M2	METASTASIS	chr7	1.07E+08	1.07E+08	A	T	COG5	exonic	ynonymou	T000003470	62	15	77	0.194805	27	0	27	0	0.85	Clonal	0.46	1.81			polymorphism	14.92	p.S11T	Passenger	SENGER/OT	benign	Neutral	Tolerated
OVA_003_M3	METASTASIS	chr7	1.07E+08	1.07E+08	A	T	COG5	exonic	ynonymou	T000003470	65	8	73	0.109589	27	0	27	0	1	Clonal	0.34	2.67			polymorphism	14.92	p.S11T	Passenger	SENGER/OT	benign	Neutral	Tolerated
OVA_003_C1	PRIMARY	chr7	1.07E+08	1.07E+08	A	T	COG5	exonic	ynonymou	T000003470	37	29	66	0.439394	27	0	27	0	1	Clonal	0.86	1.81			polymorphism	14.92	p.S11T	Passenger	SENGER/OT	benign	Neutral	Tolerated
OVA_003_C2	PRIMARY	chr7	1.07E+08	1.07E+08	A	T	COG5	exonic	ynonymou	T000003470	39	27	66	0.409091	27	0	27	0	1	Clonal	0.7	3.61			polymorphism	14.92	p.S11T	Passenger	SENGER/OT	benign	Neutral	Tolerated
OVA_003_C3	PRIMARY	chr7	1.07E+08	1.07E+08	A	T	COG5	exonic	ynonymou	T000003470	42	19	61	0.311475	27	0	27	0	0.76	Clonal	0.82	1.81			polymorphism	14.92	p.S11T	Passenger	SENGER/OT	benign	Neutral	Tolerated
OVA_013_C2	PRIMARY	chr7	1.07E+08	1.07E+08	C	A	SLC26A3	exonic	ynonymou	/384F,ENSG	611	44	655	0.067176	48	0	48	0	0.29	Sub	0.87	3.05			polymorphism	0.117	p.V384F	Passenger	SENGER/OT	benign	Neutral	Tolerated
OVA_013_M1	METASTASIS	chr7	1.07E+08	1.07E+08	G	C	SLC26A3	intronic	NA	NA	372	250	622	0.401929	53	0	53	0	1	Clonal	0.67	3.06			disease_causing	8.436						
OVA_013_C1	PRIMARY	chr7	1.07E+08	1.07E+08	G	C	SLC26A3	intronic	NA	NA	343	293	636	0.460692	53	0	53	0	1	Clonal	0.72	3.1			disease_causing	8.436						
OVA_013_C2	PRIMARY	chr7	1.07E+08	1.07E+08	G	C	SLC26A3	intronic	NA	NA	343	263	606	0.433993	53	0	53	0	1	Clonal	0.87	3.05			disease_causing	8.436						
OVA_013_C3	PRIMARY	chr7	1.07E+08	1.07E+08	G	C	SLC26A3	intronic	NA	NA	353	307	660	0.465152	53	0	53	0	1	Clonal	0.91	3.01			disease_causing	8.436						
OVA_003_C1	PRIMARY	chr11	1.08E+08	1.08E+08	G	A	ELMOD1	exonic	nonymous S	000026584	569	51	620	0.082258	49	0	49	0	0.19	Sub	0.86	1.81			disease_causing	9.937		Passenger			Neutral	Tolerated
OVA_003_C2	PRIMARY	chr11	1.08E+08	1.08E+08	G	A	ELMOD1	exonic	nonymous S	000026584	572	48	620	0.077419	49	0	49	0	0.38	Sub	0.7	3.61			disease_causing	9.937		Passenger			Neutral	Tolerated
OVA_003_C3	PRIMARY	chr11	1.08E+08	1.08E+08	G	A	ELMOD1	exonic	nonymous S	000026584	657	40	697	0.057389	49	0	49	0	0.14	Sub	0.82	1.81			disease_causing	9.937		Passenger			Neutral	Tolerated
OVA_047_C1	PRIMARY	chr9	1.08E+08	1.08E+08	A	C	ABCA1	intronic	NA	NA	362	180	542	0.332103	38	0	38	0	1	Clonal	0.6	2.91			polymorphism	5.857						
OVA_047_C2	PRIMARY	chr9	1.08E+08	1.08E+08	A	C	ABCA1	intronic	NA	NA	393	176	569	0.309315	38	0	38	0	0.97	Clonal	0.64	2.76			polymorphism	5.857						
OVA_047_C3	Contralateral ovary	chr9	1.08E+08	1.08E+08	A	C	ABCA1	intronic	NA	NA	354	172	526	0.326996	38	0	38	0	1	Clonal	0.6	2.94			polymorphism	5.857						
OVA_047_C1	PRIMARY	chr7	1.08E+08	1.08E+08	ACCAG	-	LAMB1	exonic	neshift dele	0222399:ex	658	222	880	0.252273	26	0	26		0.84	Sub	0.6	2.91					p.L508Dfs	Passenger			NA	NA
OVA_047_C2	PRIMARY	chr7	1.08E+08	1.08E+08	ACCAG	-	LAMB1	exonic	neshift dele	0222399:ex	1527	320	1847	0.173254	26	0	26		0.54	Sub	0.64	2.76					p.L508Dfs	Passenger			NA	NA
OVA_047_C3	Contralateral ovary	chr7	1.08E+08	1.08E+08	ACCAG	-	LAMB1	exonic	neshift dele	0222399:ex	1472	300	1772	0.1693	26	0	26		0.74	Sub	0.6	2.94					p.L508Dfs	Passenger			NA	NA
OVA_047_C3	Contralateral ovary	chr3	1.08E+08	1.08E+08	C	G	LINC00636	cRNA_exon	NA	NA	695	94	789	0.119138	95	0	95	0	1	Clonal	0.6	2.94			polymorphism	8.548					Neutral	NA
OVA_378_C1	PRIMARY	chr1	1.08E+08	1.08E+08	G	T	VAV3	exonic	ynonymou	3I,ENSG00	542	103	645	0.15969	58	0	58	0	0.86	Sub	0.59	2.6			polymorphism	11.28	p.L619I	Passenger	SENGER/OT	benign	Neutral	Tolerated
OVA_378_C2	PRIMARY	chr1	1.08E+08	1.08E+08	G	T	VAV3	exonic	ynonymou	3I,ENSG00	462	115	577	0.199307	58	0	58	0	1	Sub	0.56	2.59			polymorphism	11.28	p.L619I	Passenger	SENGER/OT	benign	Neutral	Tolerated
OVA_378_C3	PRIMARY	chr1	1.08E+08	1.08E+08	G	T	VAV3	exonic	ynonymou	3I,ENSG00	474	73	547	0.133455	58	0	58	0	0.66	Sub	0.51	2.6			polymorphism	11.28	p.L619I	Passenger	SENGER/OT	benign	Neutral	Tolerated
OVA_003_M1	METASTASIS	chr6	1.08E+08	1.08E+08	T	A	SEC63	intronic	NA	NA	53	17	70	0.242857	24	0	24	0	0.86	Clonal	0.57	1.84			polymorphism	5.798						
OVA_003_M2	METASTASIS	chr6	1.08E+08	1.08E+08	T	A	SEC63	intronic	NA	NA	75	16	91	0.175824	24	0	24	0	0.77	Clonal	0.46	1.81			polymorphism	5.798						
OVA_003_C1	PRIMARY	chr6	1.08E+08	1.08E+08	T	A	SEC63	intronic	NA	NA	45	20	65	0.307692	24	0	24	0	0.71	Sub	0.86	1.81			polymorphism	5.798						
OVA_003_C2	PRIMARY	chr6	1.08E+08	1.08E+08	T	A	SEC63	intronic	NA	NA	60	32	92	0.347826	24	0	24	0	1	Sub	0.7	3.61			polymorphism	5.798						
OVA_003_C3	PRIMARY	chr6	1.08E+08	1.08E+08	T	A	SEC63	intronic	NA	NA	48	35	83	0.421687	24	0	24	0	1	Sub	0.82	1.81			polymorphism	5.798						
OVA_047_M1	METASTASIS	chr11	1.08E+08	1.08E+08	G	A	C11orf65	exonic	nonymous S	241N,ENSG	448	212	660	0.321212	41	0	41	0	1	Clonal	0.47	2.01			disease_causing	10.62		Passenger			Neutral	Tolerated
OVA_047_M2	METASTASIS	chr11	1.08E+08	1.08E+08	G	A	C11orf65	exonic	nonymous S	241N,ENSG	476	201	677	0.296898	41	0	41	0	1	Clonal	0.44	2.01			disease_causing	10.62		Passenger			Neutral	Tolerated
OVA_047_M3	METASTASIS	chr11	1.08E+08	1.08E+08	G	A	C11orf65	exonic	nonymous S	241N,ENSG	308	144	452	0.318584	41	0	41	0	1	Clonal	0.6	3.02			disease_causing	10.62		Passenger			Neutral	Tolerated
OVA_378_M1	METASTASIS	chr13	1.09E+08	1.09E+08	A	G	TNFSF13B	exonic	ynonymou	000004305	320	204	524	0.389313	62	0	62	0	1	Sub	0.72	2.57			polymorphism	17.13	p.S36G	Passenger	SENGER/OT	benign	Neutral	Tolerated
OVA_378_M2	METASTASIS	chr13	1.09E+08	1.09E+08	A	G	TNFSF13B	exonic	ynonymou	000004305	249	215	464	0.463362	62	0	62	0	1	Sub	0.85	2.57			polymorphism	17.13	p.S36G	Passenger	SENGER/OT	benign	Neutral	Tolerated
OVA_378_M3	METASTASIS	chr13	1.09E+08	1.09E+08	A	G	TNFSF13B	exonic	ynonymou	000004305	407	163	570	0.285965	62	0	62	0	0.86	Sub	0.67	2.57			polymorphism	17.13	p.S36G	Passenger	SENGER/OT	benign	Neutral	Tolerated
OVA_378_C1	PRIMARY	chr13	1.09E+08	1.09E+08	A	G	TNFSF13B	exonic	ynonymou	000004305	394	161	555	0.29009	62	0	62	0	0.99	Sub	0.59	2.6			polymorphism	17.13	p.S36G	Passenger	SENGER/OT	benign	Neutral	Tolerated
OVA_378_C2	PRIMARY	chr13	1.09E+08	1.09E+08	A	G	TNFSF13B	exonic	ynonymou	000004305	374	147	521	0.28215	62	0	62	0	1	Sub	0.56	2.59			polymorphism	17.13	p.S36G	Passenger	SENGER/OT	benign	Neutral	Tolerated
OVA_378_C3	PRIMARY	chr13	1.09E+08	1.09E+08	A	G	TNFSF13B	exonic	ynonymou	000004305	427	102	529	0.192817	62	0	62	0	0.76	Sub	0.51	2.6			polymorphism	17.13	p.S36G	Passenger	SENGER/OT	benign	Neutral	Tolerated
OVA_013_M1	METASTASIS	chr12	1.09E+08	1.09E+08	C	G	ISCU	UTR3	NA	NA	378	195	573	0.340314	89	0	89	0	1	Clonal	0.67	3.06			polymorphism	6.866						
OVA_013_C1	PRIMARY	chr12	1.09E+08	1.09E+08	C	G	ISCU	UTR3	NA	NA	324	227	551	0.411978	89	0	89	0	1	Sub	0.72	3.1			polymorphism	6.866						
OVA_013_C2	PRIMARY	chr12	1.09E+08	1.09E+08	C	G	ISCU	UTR3	NA	NA	286	224	510	0.439216	89	0	89	0	1	Sub	0.87	3.05			polymorphism	6.866						
OVA_013_C3	PRIMARY	chr12	1.09E+08	1.09E+08	C	G	ISCU	UTR3	NA	NA	352	227	579	0.392055	89	0	89	0	0.86	Sub	0.91	3.01			polymorphism	6.866						
OVA_048_M1	METASTASIS	chr12	1.09E+08	1.09E+08	C	T	TMEM119	exonic	ynonymou	000039280	94	2	96	0.020833	16	0	16	0	0.18	Sub	0.4	3.87			polymorphism	1.307	p.G193S	Passenger	SENGER/OT	benign	Neutral	Tolerated
OVA_048_M2	METASTASIS	chr12	1.09E+08	1.09E+08	C	T	TMEM119	exonic	ynonymou	000039280	33	4	37	0.108108	16	0	16	0	1	Sub	0.4	3.87			polymorphism	1.307	p.G193S	Passenger	SENGER/OT	benign	Neutral	Tolerated
OVA_048_M3	METASTASIS	chr12	1.09E+08	1.09E+08	C	T	TMEM119	exonic	ynonymou	000039280	52	9	61	0.147541	16	0	16	0	1	Sub	0.59	3.39			polymorphism	1.307	p.G193S	Passenger	SENGER/OT	benign	Neutral	Tolerated
OVA_047_C1	PRIMARY	chr8	1.09E+08	1.09E+08	A	G	RSPO2	UTR5	NA	NA	665	69	734	0.094005	42	0	42	0	0.5	Sub	0.6	2.91	Yes		polymorphism	7.23						
OVA_047_C3	Contralateral ovary	chr8	1.09E+08	1.09E+08	A	G	RSPO2	UTR5	NA	NA	677	35	712	0.049157	42	0	42	0	0.31	Sub	0.6	2.94	Yes		polymorphism	7.23						
OVA_003_M1	METASTASIS	chr13	1.09E+08	1.09E+08	G	T	MYO16	intronic	NA	NA	936	15	951	0.015773	55	1	56	0.017857	0.06	Sub	0.57	1.84			polymorphism	5.133						
OVA_003_C1	PRIMARY	chr13	1.09E+08	1.09E+08	G	T	MYO16	intronic	NA	NA	347	201	548	0.366788	55	1	56	0.017857	0.85	Sub	0.86	1.81			polymorphism	5.133						
OVA_003_C2	PRIMARY	chr13	1.09E+08	1.09E+08	G	T	MYO16	intronic	NA	NA	477	96	573	0.167539	55	1	56	0.017857	0.98	Sub	0.7	3.61			polymorphism	5.133						
OVA_003_C3	PRIMARY	chr13	1.09E+08	1.09E+08	G	T	MYO16	intronic	NA	NA	374	203	577	0.35182	55	1	56	0.017857	0.85	Sub	0.82	1.81			polymorphism	5.133						
OVA_365_M1	METASTASIS	chr6	1.1E+08	1.1E+08	C	A	ZBTB24	UTR3	NA	NA	249	232	481	0.482328	10	0	10	0	1	Clonal	0.85	1.87			polymorphism	3.086						
OVA_365_M2	METASTASIS	chr6	1.1E+08	1.1E+08	C	A	ZBTB24	UTR3	NA	NA	148	46	194	0.237113	10	0	10	0	1	Clonal	0.4	1.81			polymorphism	3.086						
OVA_365_M3	METASTASIS	chr6	1.1E+08	1.1E+08	C	A	ZBTB24	UTR3	NA	NA	187	177	364	0.486264	10	0	10	0	1	Clonal	0.82	1.89			polymorphism	3.086						
OVA_365_C2	PRIMARY	chr6	1.1E+08	1.1E+08	C	A	ZBTB24	UTR3	NA	NA	257	107	364	0.293956	10	0	10	0	0.95	Clonal	0.62	1.92			polymorphism	3.086						
OVA_013_M1	METASTASIS	chr6	1.1E+08	1.1E+08	C	T	ZBTB24	exonic	ynonymou	000023012	384	192	576	0.333333	140	0	140	0	0.99	Clonal	0.67	3.06			disease_causing	26.3	p.G284E	Passenger	SENGER/OT	damaging	Neutral	Tolerated
OVA_013_C1	PRIMARY	chr6	1.1E+08	1.1E+08	C	T	ZBTB24	exonic	ynonymou	000023012	342	230	572	0.402098	140	0	140	0	1	Clonal	0.72	3.1			disease_causing	26.3	p.G284E	Passenger	SENGER/OT	damaging	Neutral	Tolerated
OVA_013_C2	PRIMARY	chr6	1.1E+08	1.1E+08	C	T	ZBTB24	exonic	ynonymou	000023012	313	238	551	0.431942	140	0	140	0	0.99	Clonal	0.87	3.05			disease_causing	26.3	p.G284E	Passenger	SENGER/OT	damaging	Neutral	Tolerated
OVA_013_C3	PRIMARY	chr6	1.1E+08	1.1E+08	C	T	ZBTB24	exonic	ynonymou	000023012	340	239	579	0.412781	140	0	140	0	0.9	Clonal	0.91	3.01			disease_causing	26.3	p.G284E	Passenger	SENGER/OT	damaging	Neutral	Tolerated
OVA_003_C3	PRIMARY	chr4	1.1E+08	1.1E+08	C	G	COL25A1	exonic	ynonymou	156A,ENSG	504	55	559	0.09839	34	0	34	0	0.14	Sub	0.82	1.81			disease_causing	30	p.G156A	Passenger	SENGER/OT	damaging	Deleterious	Damaging
OVA_003_M1	METASTASIS	chr1	1.1E+08	1.1E+08	C	A	GSTM3	exonic	ynonymou	000036106	432	157	589	0.266553	43	0	43	0	0.94	Sub	0.57	1.84			disease_causing	26.7	p.D110Y	Passenger	SENGER/OT	damaging	Deleterious	Damaging
OVA_003_M2	METASTASIS	chr1	1.1E+08	1.1E+08	C	A	GSTM3	exonic	ynonymou	000036106	477	133	610	0.218033	43	0	43	0	0.95	Sub	0.46	1.81			disease_causing	26.7	p.D110Y	Passenger	SENGER/OT	damaging	Deleterious	Damaging
OVA_003_M3	METASTASIS	chr1	1.1E+08	1.1E+08	C	A	GSTM3	exonic	ynonymou	000036106	615	43	658	0.06535	43	0	43	0	0.69	Sub	0.34	2.67			disease_causing	26.7	p.D110Y	Passenger	SENGER/OT	damaging	Deleterious	Damaging
OVA_003_C1	PRIMARY	chr1	1.1E+08	1.1E+08	C	A	GSTM3	exonic	ynonymou	000036106	392	276	668	0.413174	43	0	43	0	0.96	Clonal	0.86	1.81			disease_causing	26.7	p.D110Y	Passenger	SENGER/OT	damaging	Deleterious	Damaging
OVA_003_C2	PRIMARY	chr1	1.1E+08	1.1E+08	C	A	GSTM3	exonic	ynonymou	000036106	377	276	653	0.422665	43	0	43	0	1	Clonal	0.7	3.61			disease_causing	26.7	p.D110Y	Passenger	SENGER/OT	damaging	Deleterious	Damaging
OVA_003_C3	PRIMARY	chr1	1.1E+08	1.1E+08	C	A	GSTM3	exonic	ynonymou	000036106	410	278	688	0.40407	43	0	43	0	0.98	Clonal	0.82	1.81			disease_causing	26.7	p.D110Y	Passenger	SENGER/OT	damaging	Deleterious	Damaging
OVA_003_M1	METASTASIS	chr1	1.1E+08	1.1E+08	CCTCGGC	-	EPS8L3	RNA_intro	NA	NA	565	184	749	0.245661	26	0	26		0.87	Sub	0.57	1.84										
OVA_003_M2	METASTASIS	chr1	1.1E+08	1.1E+08	CCTCGGC	-	EPS8L3	RNA_intro	NA	NA	714	172	886	0.194131	26	0	26		0.85	Sub	0.57	1.84										
OVA_003_M3	METASTASIS	chr1	1.1E+08	1.1E+08	CCTCGGC	-	EPS8L3	RNA_intro	NA	NA	881	53	934	0.056745	26	0	26		0.6	Sub	0.57	1.84										
OVA_003_C1	PRIMARY	chr1	1.1E+08	1.1E+08	CCTCGGC	-	EPS8L3	RNA_intro	NA	NA	321	200	521	0.383877	26	0	26		0.89	Sub	0.86	1.81										
OVA_003_C2	PRIMARY	chr1	1.1E+08	1.1E+08	CCTCGGC	-	EPS8L3	RNA_intro	NA	NA	385	182	567	0.320988	26	0	26		1	Sub	0.7	3.61										
OVA_003_C3	PRIMARY	chr1	1.1E+08	1.1E+08	CCTCGGC	-	EPS8L3	RNA_intro	NA	NA	434	245	679	0.360825	26	0	26		0.88	Sub	0.82	1.81										
OVA_365_M1	METASTASIS	chr7	1.1E+08	1.1E+08	C	G	IMMP2L	exonic	ynonymou	000040570	405	116	521	0.222649	53	0	53	0	0.75	Sub	0.85	1.87			disease_causing	33	p.W156C	Passenger	no weights	damaging	Deleterious	Damaging
OVA_365_M2	METASTASIS	chr7	1.1E+08	1.1E+08	C	G	IMMP2L	exonic	ynonymou	000040570	320	51	371	0.137466	53	0	53	0	0.82	Sub	0.4	1.81			disease_causing	33	p.W156C	Passenger	no weights	damaging	Deleterious	Damaging
OVA_365_M3	METASTASIS	chr7	1.1E+08	1.1E+08	C	G	IMMP2L	exonic	ynonymou	000040570	394	122	516	0.236434	53	0	53	0	0.81	Sub	0.82	1.89			disease_causing	33	p.W156C	Passenger	no weights	damaging	Deleterious	Damaging
OVA_365_C1	Contralateral ovary	chr7	1.1E+08	1.1E+08	C	G	IMMP2L	exonic	ynonymou	000040570	312	78	390	0.2	53	0	53	0	1	Clonal	0.42	1.83			disease_causing	33	p.W156C	Passenger	no weights	damaging	Deleterious	Damaging
OVA_365_C2	PRIMARY	chr7	1.1E+08	1.1E+08	C	G	IMMP2L	exonic	ynonymou	000040570	373	102	475	0.214737	53	0	53	0	0.91	Clonal	0.62	1.92			disease_causing	33	p.W156C	Passenger	no weights	damaging	Deleterious	Damaging
OVA_047_M1	METASTASIS	chr1	1.1E+08	1.1E+08	G	A	CSF1	intronic	NA	NA	635	27	662	0.040785	72	0	72	0	0.21	Sub	0.47	2.01			polymorphism	13.76						
OVA_048_C1	PRIMARY	chr12	1.11E+08	1.11E+08	A	G	ATP2A2	UTR3	NA	NA	80	189	269	0.702602	13	0	13	0	1	Clonal	0.67	1.82			disease_causing	15.98						
OVA_048_C2	PRIMARY	chr12	1.11E+08	1.11E+08	A	G	ATP2A2	UTR3	NA	NA	82	225	307	0.732899	13	0	13	0	1	Clonal	0.72	1.85			disease_causing	15.98						
OVA_048_C3	PRIMARY	chr12	1.11E+08	1.11E+08	A	G	ATP2A2	UTR3	NA	NA	59	139	198	0.70202	13	0	13	0	1	Clonal	0.74	1.8			disease_causing	15.98						
OVA_047_M1	METASTASIS	chr6	1.11E+08	1.11E+08	C	A	CDK19	exonic	ynonymou	000003238	467	153	620	0.246774	39	0	39	0	1	Clonal	0.47	2.01			disease_causing	26	p.Q134H	Driver	SENGER/OT	damaging	Deleterious	Damaging
OVA_047_M2	METASTASIS	chr6	1.11E+08	1.11E+08	C	A	CDK19	exonic	ynonymou	000003238	491	135	626	0.215655	39	0	39	0	0.97	Clonal	0.44	2.01			disease_causing	26	p.Q134H	Driver	SENGER/OT	damaging	Deleterious	Damaging
OVA_047_M3	METASTASIS	chr6	1.11E+08	1.11E+08	C	A	CDK19	exonic	ynonymou	000003238	333	106	439	0.241458	39	0	39	0	1	Clonal	0.6	3.02			disease_causing	26	p.Q134H	Driver	SENGER/OT	damaging	Deleterious	Damaging
OVA_365_M1	METASTASIS	chr13	1.11E+08	1.11E+08	G	A	ING1	exonic	ynonymou	000037577	3	19	22	0.863636	91	0	91	0	1	Clonal	0.85	1.87			polymorphism	6.272	p.G101S	Passenger	CANCER	benign	Neutral	Tolerated
OVA_365_M2	METASTASIS	chr13	1.11E+08	1.11E+08	G	A	ING1	exonic	ynonymou	000037577	8	5	13	0.384615	91	0	91	0	1	Clonal	0.4	1.81			polymorphism	6.272	p.G101S	Passenger	CANCER	benign	Neutral	Tolerated
OVA_365_M3	METASTASIS	chr13	1.11E+08	1.11E+08	G	A	ING1	exonic	ynonymou	000037577	3	10	13	0.769231	91	0	91	0	1	Clonal	0.82	1.89			polymorphism	6.272	p.G101S	Passenger	CANCER	benign	Neutral	Tolerated
OVA_048_M1	METASTASIS	chr9	1.12E+08	1.12E+08	-	A	TMEM245	intronic	NA	NA	285	111	396	0.280303	27	0	27		1	Clonal	0.56	3.94			polymorphism							
OVA_048_M2	METASTASIS	chr9	1.12E+08	1.12E+08	-	A	TMEM245	intronic	NA	NA	264	126	390	0.323077	27	0	27		1	Clonal	0.4	3.87			polymorphism							
OVA_048_M3	METASTASIS	chr9	1.12E+08	1.12E+08	-	A	TMEM245	intronic	NA	NA	216	170	386	0.440415	27	0	27		1	Clonal	0.59	3.39			polymorphism							
OVA_003_M1	METASTASIS	chr7	1.12E+08	1.12E+08	G	C	ZNF277	splicing	NA	NA	127	67	194	0.345361	26	1	27	0.037037	1	Sub	0.57	1.84			disease_causing	33		Passenger				
OVA_003_M2	METASTASIS	chr7	1.12E+08	1.12E+08	G	C	ZNF277	splicing	NA	NA	184	54	238	0.226891	26	1	27	0.037037	0.99	Sub	0.46	1.81			disease_causing	33		Passenger				
OVA_003_M3	METASTASIS	chr7	1.12E+08	1.12E+08	G	C	ZNF277	splicing	NA	NA	324	16	340	0.047059	26	1	27	0.037037	0.5	Sub	0.34	2.67			disease_causing	33		Passenger				
OVA_003_C1	PRIMARY	chr7	1.12E+08	1.12E+08	G	C	ZNF277	splicing	NA	NA	184	157	341	0.460411	26	1	27	0.037037	1	Clonal	0.86	1.81			disease_causing	33		Passenger				
OVA_003_C2	PRIMARY	chr7	1.12E+08	1.12E+08	G	C	ZNF277	splicing	NA	NA	231	160	391	0.409207	26	1	27	0.037037	1	Clonal	0.7	3.61			disease_causing	33		Passenger				
OVA_003_C3	PRIMARY	chr7	1.12E+08	1.12E+08	G	C	ZNF277	splicing	NA	NA	244	145	389	0.372751	26	1	27	0.037037	0.9	Clonal	0.82	1.81			disease_causing	33		Passenger				
OVA_047_C1	PRIMARY	chr13	1.12E+08	1.12E+08	A	G	HGEF7;TEX	upstream	NA	NA	329	141	470	0.3	18	0	18	0	1	Sub	0.6	2.91				2.414						
OVA_047_C2	PRIMARY	chr13	1.12E+08	1.12E+08	A	G	HGEF7;TEX	upstream	NA	NA	388	164	552	0.297101	18	0	18	0	0.63	Sub	0.64	2.76				2.414						
OVA_047_C3	Contralateral ovary	chr13	1.12E+08	1.12E+08	A	G	HGEF7;TEX	upstream	NA	NA	335	125	460	0.271739	18	0	18	0	0.91	Clonal	0.6	2.94				2.414						

Sample	Type	Chr	Start	End	Ref	Alt	Gene	unc.ensGen	icFunc.ens	hange.ens	t_ref	t_alt	t_total	t_VAF	n_ref	n_alt	n_total	normal_VA	ncer_cell_f	on-based C	purity	ploidy	Cancer Gene	Driver Gene	Mutation Taster	ADD(PHRE	otein_chan	CHASM	ATHMM_Pr	OLYPHEN-	REDICTION	ICTION (cutoff=0.05)
OVA_047_M1	METASTASIS	chr1	1.12E+08	1.12E+08	T	A	C1orf162	intronic	NA	NA	586	127	713	0.178121	58	0	58	0	0.93	Clonal	0.47	2.01			disease_causing	10.72						
OVA_047_M2	METASTASIS	chr1	1.12E+08	1.12E+08	T	A	C1orf162	intronic	NA	NA	570	149	719	0.207232	58	0	58	0	1	Clonal	0.44	2.01			disease_causing	10.72						
OVA_047_M3	METASTASIS	chr1	1.12E+08	1.12E+08	T	A	C1orf162	intronic	NA	NA	371	87	458	0.189956	58	0	58	0	1	Clonal	0.6	3.02			disease_causing	10.72						
OVA_378_M1	METASTASIS	chr11	1.12E+08	1.12E+08	G	T	BCO2	exonic	ynonymou	T00000527	363	169	532	0.317669	32	0	32	0	0.88	Sub	0.72	2.57			polymorphism	4.51	p.S12I	Passenger	SENGER/OT	benign	Neutral	Tolerated
OVA_378_M2	METASTASIS	chr11	1.12E+08	1.12E+08	G	T	BCO2	exonic	ynonymou	T00000527	316	178	494	0.360324	32	0	32	0	0.84	Sub	0.85	2.57			polymorphism	4.51	p.S12I	Passenger	SENGER/OT	benign	Neutral	Tolerated
OVA_378_M3	METASTASIS	chr11	1.12E+08	1.12E+08	G	T	BCO2	exonic	ynonymou	T00000527	430	203	633	0.320695	32	0	32	0	0.96	Sub	0.67	2.57			polymorphism	4.51	p.S12I	Passenger	SENGER/OT	benign	Neutral	Tolerated
OVA_378_C1	PRIMARY	chr11	1.12E+08	1.12E+08	G	T	BCO2	exonic	ynonymou	T00000527	417	190	607	0.313015	32	0	32	0	1	Clonal	0.59	2.6			polymorphism	4.51	p.S12I	Passenger	SENGER/OT	benign	Neutral	Tolerated
OVA_378_C2	PRIMARY	chr11	1.12E+08	1.12E+08	G	T	BCO2	exonic	ynonymou	T00000527	383	136	519	0.262042	32	0	32	0	0.93	Clonal	0.56	2.59			polymorphism	4.51	p.S12I	Passenger	SENGER/OT	benign	Neutral	Tolerated
OVA_378_C3	PRIMARY	chr11	1.12E+08	1.12E+08	G	T	BCO2	exonic	ynonymou	T00000527	473	100	573	0.17452	32	0	32	0	0.86	Clonal	0.51	2.6			polymorphism	4.51	p.S12I	Passenger	SENGER/OT	benign	Neutral	Tolerated
OVA_047_M1	METASTASIS	chr7	1.12E+08	1.12E+08	A	G	TMEM168	exonic	nonymous S	278L,ENSG	537	178	715	0.248951	58	0	58	0	1	Clonal	0.47	2.01			disease_causing	10.27		Passenger			Neutral	Tolerated
OVA_047_M2	METASTASIS	chr7	1.12E+08	1.12E+08	A	G	TMEM168	exonic	nonymous S	278L,ENSG	540	188	728	0.258242	58	0	58	0	1	Clonal	0.44	2.01			disease_causing	10.27		Passenger			Neutral	Tolerated
OVA_047_M3	METASTASIS	chr7	1.12E+08	1.12E+08	A	G	TMEM168	exonic	nonymous S	278L,ENSG	344	137	481	0.284823	58	0	58	0	1	Clonal	0.6	3.02			disease_causing	10.27		Passenger			Neutral	Tolerated
OVA_003_M1	METASTASIS	chr1	1.13E+08	1.13E+08	C	T	WNT2B	exonic	ynonymou	000036968	231	103	334	0.308383	52	0	52	0	1	Sub	0.57	1.84			disease_causing	30	p.R197C	Passenger	SENGER/OT	damaging	Deleterious	Damaging
OVA_003_M2	METASTASIS	chr1	1.13E+08	1.13E+08	C	T	WNT2B	exonic	ynonymou	000036968	306	90	396	0.227273	52	0	52	0	0.99	Sub	0.46	1.81			disease_causing	30	p.R197C	Passenger	SENGER/OT	damaging	Deleterious	Damaging
OVA_003_M3	METASTASIS	chr1	1.13E+08	1.13E+08	C	T	WNT2B	exonic	ynonymou	000036968	445	27	472	0.057203	52	0	52	0	0.6	Sub	0.34	2.67			disease_causing	30	p.R197C	Passenger	SENGER/OT	damaging	Deleterious	Damaging
OVA_003_C1	PRIMARY	chr1	1.13E+08	1.13E+08	C	T	WNT2B	exonic	ynonymou	000036968	245	171	416	0.411058	52	0	52	0	0.95	Clonal	0.86	1.81			disease_causing	30	p.R197C	Passenger	SENGER/OT	damaging	Deleterious	Damaging
OVA_003_C2	PRIMARY	chr1	1.13E+08	1.13E+08	C	T	WNT2B	exonic	ynonymou	000036968	267	207	474	0.436709	52	0	52	0	1	Clonal	0.7	3.61			disease_causing	30	p.R197C	Passenger	SENGER/OT	damaging	Deleterious	Damaging
OVA_003_C3	PRIMARY	chr1	1.13E+08	1.13E+08	C	T	WNT2B	exonic	ynonymou	000036968	351	213	564	0.37766	52	0	52	0	0.92	Clonal	0.82	1.81			disease_causing	30	p.R197C	Passenger	SENGER/OT	damaging	Deleterious	Damaging
OVA_048_M1	METASTASIS	chr11	1.13E+08	1.13E+08	G	A	NCAM1	intronic	NA	NA	344	167	511	0.32681	39	0	39	0	1	Clonal	0.56	3.94			polymorphism	0.91						
OVA_048_M2	METASTASIS	chr11	1.13E+08	1.13E+08	G	A	NCAM1	intronic	NA	NA	382	127	509	0.249509	39	0	39	0	1	Clonal	0.4	3.87			polymorphism	0.91						
OVA_048_M3	METASTASIS	chr11	1.13E+08	1.13E+08	G	A	NCAM1	intronic	NA	NA	347	163	510	0.319608	39	0	39	0	1	Clonal	0.59	3.39			polymorphism	0.91						
OVA_048_C1	PRIMARY	chr11	1.13E+08	1.13E+08	G	A	NCAM1	intronic	NA	NA	156	82	238	0.344538	39	0	39	0	1	Clonal	0.67	1.82			polymorphism	0.91						
OVA_048_C2	PRIMARY	chr11	1.13E+08	1.13E+08	G	A	NCAM1	intronic	NA	NA	304	150	454	0.330396	39	0	39	0	0.92	Clonal	0.72	1.85			polymorphism	0.91						
OVA_048_C3	PRIMARY	chr11	1.13E+08	1.13E+08	G	A	NCAM1	intronic	NA	NA	200	102	302	0.337748	39	0	39	0	0.91	Clonal	0.74	1.8			polymorphism	0.91						
OVA_378_M1	METASTASIS	chr3	1.13E+08	1.13E+08	T	G	SPICE1	exonic	ynonymou	000295872	557	109	666	0.163664	49	0	49	0	0.94	Sub	0.72	2.57			disease_causing	17.6	p.E423D	Passenger	SENGER/OT	benign	Neutral	Damaging
OVA_378_M2	METASTASIS	chr3	1.13E+08	1.13E+08	T	G	SPICE1	exonic	ynonymou	000295872	559	131	690	0.189855	49	0	49	0	1	Sub	0.85	2.57			disease_causing	17.6	p.E423D	Passenger	SENGER/OT	benign	Neutral	Damaging
OVA_378_M3	METASTASIS	chr3	1.13E+08	1.13E+08	T	G	SPICE1	exonic	ynonymou	000295872	639	101	740	0.136486	49	0	49	0	0.82	Sub	0.67	2.57			disease_causing	17.6	p.E423D	Passenger	SENGER/OT	benign	Neutral	Damaging
OVA_378_C1	PRIMARY	chr3	1.13E+08	1.13E+08	T	G	SPICE1	exonic	ynonymou	000295872	570	128	698	0.183381	49	0	49	0	1	Clonal	0.59	2.6			disease_causing	17.6	p.E423D	Passenger	SENGER/OT	benign	Neutral	Damaging
OVA_378_C2	PRIMARY	chr3	1.13E+08	1.13E+08	T	G	SPICE1	exonic	ynonymou	000295872	584	99	683	0.144949	49	0	49	0	1	Clonal	0.56	2.59			disease_causing	17.6	p.E423D	Passenger	SENGER/OT	benign	Neutral	Damaging
OVA_378_C3	PRIMARY	chr3	1.13E+08	1.13E+08	T	G	SPICE1	exonic	ynonymou	000295872	556	87	643	0.135303	49	0	49	0	1	Clonal	0.51	2.6			disease_causing	17.6	p.E423D	Passenger	SENGER/OT	benign	Neutral	Damaging
OVA_013_M1	METASTASIS	chr1	1.13E+08	1.13E+08	C	G	RHOC	exonic	ynonymou	000036963	352	100	452	0.221239	55	0	55	0	0.88	Clonal	0.67	3.06			disease_causing	22.6	p.M82I	Passenger	CANCER	benign	Neutral	Tolerated
OVA_048_C1	PRIMARY	chr12	1.13E+08	1.13E+08	C	T	OAS3	RNA_intro	NA	NA	38	23	61	0.377049	17	0	17	0	0.75	Sub	0.67	1.82			polymorphism	7.928						
OVA_048_C2	PRIMARY	chr12	1.13E+08	1.13E+08	C	T	OAS3	RNA_intro	NA	NA	80	106	186	0.569892	17	0	17	0	1	Sub	0.72	1.85			polymorphism	7.928						
OVA_048_C3	PRIMARY	chr12	1.13E+08	1.13E+08	C	T	OAS3	RNA_intro	NA	NA	37	64	101	0.633663	17	0	17	0	1	Sub	0.74	1.8			polymorphism	7.928						
OVA_047_M1	METASTASIS	chr9	1.14E+08	1.14E+08	C	T	MUSK	exonic	ynonymou	000189978	541	152	693	0.219336	83	0	83	0	0.92	Sub	0.47	2.01			disease_causing	28.4	p.P824S	Passenger	CANCER	damaging	Deleterious	Damaging
OVA_047_M2	METASTASIS	chr9	1.14E+08	1.14E+08	C	T	MUSK	exonic	ynonymou	000189978	573	124	697	0.177905	83	0	83	0	0.8	Sub	0.44	2.01			disease_causing	28.4	p.P824S	Passenger	CANCER	damaging	Deleterious	Damaging
OVA_047_M3	METASTASIS	chr9	1.14E+08	1.14E+08	C	T	MUSK	exonic	ynonymou	000189978	433	117	550	0.212727	83	0	83	0	0.92	Sub	0.6	3.02			disease_causing	28.4	p.P824S	Passenger	CANCER	damaging	Deleterious	Damaging
OVA_365_C2	PRIMARY	chr3	1.14E+08	1.14E+08	T	A	TIGIT	UTR5	NA	NA	332	128	460	0.278261	49	0	49	0	0.9	Clonal	0.62	1.92			polymorphism	9.349						
OVA_048_M1	METASTASIS	chr13	1.14E+08	1.14E+08	G	A	TMCO3	exonic	ynonymou	.A19T,ENSG	224	153	377	0.405836	44	0	44	0	1	Clonal	0.56	3.94			polymorphism	2.862	p.A19T	Passenger	SENGER/OT	benign	Neutral	Tolerated
OVA_048_M2	METASTASIS	chr13	1.14E+08	1.14E+08	G	A	TMCO3	exonic	ynonymou	.A19T,ENSG	247	122	369	0.330623	44	0	44	0	1	Clonal	0.4	3.87			polymorphism	2.862	p.A19T	Passenger	SENGER/OT	benign	Neutral	Tolerated
OVA_048_M3	METASTASIS	chr13	1.14E+08	1.14E+08	G	A	TMCO3	exonic	ynonymou	.A19T,ENSG	171	202	373	0.541555	44	0	44	0	1	Clonal	0.59	3.39			polymorphism	2.862	p.A19T	Passenger	SENGER/OT	benign	Neutral	Tolerated
OVA_047_C1	PRIMARY	chr4	1.14E+08	1.14E+08	G	T	ANK2	exonic	ynonymou	000005032	460	196	656	0.29878	61	1	62	0.016129	1	Clonal	0.6	2.91			disease_causing	22.7	p.A339S	Passenger	CANCER	damaging	Neutral	Tolerated
OVA_047_C2	PRIMARY	chr4	1.14E+08	1.14E+08	G	T	ANK2	exonic	ynonymou	000005032	448	191	639	0.298905	61	1	62	0.016129	0.94	Clonal	0.64	2.76			disease_causing	22.7	p.A339S	Passenger	CANCER	damaging	Neutral	Tolerated
OVA_047_C3	Contralateral ovary	chr4	1.14E+08	1.14E+08	G	T	ANK2	exonic	ynonymou	000005032	427	186	613	0.303426	61	1	62	0.016129	1	Clonal	0.6	2.94			disease_causing	22.7	p.A339S	Passenger	CANCER	damaging	Neutral	Tolerated
OVA_003_M1	METASTASIS	chr4	1.14E+08	1.14E+08	A	T	ANK2	exonic	ynonymou	0264366:e	337	150	487	0.308008	49	0	49	0	0.78	Sub	0.57	1.84			polymorphism	18.96	p.M3292L	Passenger	CANCER	benign	Neutral	Damaging
OVA_003_M2	METASTASIS	chr4	1.14E+08	1.14E+08	A	T	ANK2	exonic	ynonymou	0264366:e	427	103	530	0.19434	49	0	49	0	0.65	Sub	0.46	1.81			polymorphism	18.96	p.M3292L	Passenger	CANCER	benign	Neutral	Damaging
OVA_003_M3	METASTASIS	chr4	1.14E+08	1.14E+08	A	T	ANK2	exonic	ynonymou	0264366:e	576	37	613	0.060359	49	0	49	0	0.46	Sub	0.34	2.67			polymorphism	18.96	p.M3292L	Passenger	CANCER	benign	Neutral	Damaging
OVA_003_C1	PRIMARY	chr4	1.14E+08	1.14E+08	A	T	ANK2	exonic	ynonymou	0264366:e	90	373	463	0.805616	49	0	49	0	1	Clonal	0.86	1.81			polymorphism	18.96	p.M3292L	Passenger	CANCER	benign	Neutral	Damaging
OVA_003_C2	PRIMARY	chr4	1.14E+08	1.14E+08	A	T	ANK2	exonic	ynonymou	0264366:e	160	357	517	0.690522	49	0	49	0	1	Clonal	0.7	3.61			polymorphism	18.96	p.M3292L	Passenger	CANCER	benign	Neutral	Damaging
OVA_003_C3	PRIMARY	chr4	1.14E+08	1.14E+08	A	T	ANK2	exonic	ynonymou	0264366:e	155	392	547	0.716636	49	0	49	0	1	Clonal	0.82	1.81			polymorphism	18.96	p.M3292L	Passenger	CANCER	benign	Neutral	Damaging
OVA_048_M1	METASTASIS	chr1	1.14E+08	1.14E+08	A	G	B1-AS1;PTF	RNA_intro	NA	NA	354	174	528	0.329545	23	0	23	0	1	Clonal	0.56	3.94			disease_causing	14.71						
OVA_048_M2	METASTASIS	chr1	1.14E+08	1.14E+08	A	G	B1-AS1;PTF	RNA_intro	NA	NA	463	59	522	0.113027	23	0	23	0	0.79	Clonal	0.4	3.87			disease_causing	14.71						
OVA_048_C2	PRIMARY	chrX	1.14E+08	1.14E+08	G	A	RBMXL3	exonic	nonymous S	000042477	57	35	92	0.380435	62	0	62	0	1	Clonal	0.72	1.85			polymorphism	2.718		Passenger			Neutral	Tolerated
OVA_048_C2	PRIMARY	chrX	1.14E+08	1.14E+08	C	A	RBMXL3	exonic	ynonymou	000042477	59	33	92	0.358696	63	0	63	0	1	Clonal	0.72	1.85			polymorphism	10.55	p.P363T	Passenger	SENGER/OT	damaging	Neutral	Damaging
OVA_048_M1	METASTASIS	chr1	1.14E+08	1.14E+08	C	T	4B1;AP4B1-	exonic	ynonymou	000036956	517	43	560	0.076786	69	0	69	0	0.43	Sub	0.56	3.94			polymorphism	11.37				benign	Neutral	Tolerated
OVA_047_M1	METASTASIS	chrX	1.15E+08	1.15E+08	C	T	LUZP4	exonic	stopgain	208X,ENSG	483	155	638	0.242947	37	0	37	0	1	Clonal	0.47	2.01			disease_causing	35	p.Q290X	Passenger			NA	NA
OVA_047_M2	METASTASIS	chrX	1.15E+08	1.15E+08	C	T	LUZP4	exonic	stopgain	208X,ENSG	484	140	624	0.224359	37	0	37	0	1	Clonal	0.44	2.01			disease_causing	35	p.Q290X	Passenger			NA	NA
OVA_047_M3	METASTASIS	chrX	1.15E+08	1.15E+08	C	T	LUZP4	exonic	stopgain	208X,ENSG	230	65	295	0.220339	37	0	37	0	0.96	Clonal	0.6	3.02			disease_causing	35	p.Q290X	Passenger			NA	NA
OVA_048_M1	METASTASIS	chr12	1.15E+08	1.15E+08	A	T	TBX5	exonic	nonymous S	203T,ENSG	463	34	497	0.06841	20	0	20	0	0.31	Sub	0.56	3.94			disease_causing	9.972		Passenger			Neutral	Tolerated
OVA_047_C1	PRIMARY	chrX	1.16E+08	1.16E+08	T	C	SLC6A14	exonic	ynonymou	T00000371	540	68	608	0.111842	82	0	82	0	0.49	Sub	0.6	2.91			polymorphism	15.76	p.F10L	Passenger	SENGER/OT	benign	Neutral	Tolerated
OVA_047_C2	PRIMARY	chrX	1.16E+08	1.16E+08	T	C	SLC6A14	exonic	ynonymou	T00000371	483	100	583	0.171527	82	0	82	0	0.71	Sub	0.64	2.76			polymorphism	15.76	p.F10L	Passenger	SENGER/OT	benign	Neutral	Tolerated
OVA_047_C3	Contralateral ovary	chrX	1.16E+08	1.16E+08	T	C	SLC6A14	exonic	ynonymou	T00000371	488	116	604	0.192053	82	0	82	0	0.84	Sub	0.6	2.94			polymorphism	15.76	p.F10L	Passenger	SENGER/OT	benign	Neutral	Tolerated
OVA_048_M1	METASTASIS	chr10	1.16E+08	1.16E+08	T	C	AFAP1L2	exonic	ynonymou	000369271	934	11	945	0.011615	48	0	48	0	0.05	Sub	0.4	3.87			disease_causing	31	p.E734G	Passenger	SENGER/OT	damaging	Deleterious	Damaging
OVA_048_M2	METASTASIS	chr10	1.16E+08	1.16E+08	T	C	AFAP1L2	exonic	ynonymou	000369271	468	23	491	0.046843	48	0	48	0	0.23	Sub	0.4	3.87			disease_causing	31	p.E734G	Passenger	SENGER/OT	damaging	Deleterious	Damaging
OVA_048_M3	METASTASIS	chr10	1.16E+08	1.16E+08	T	C	AFAP1L2	exonic	ynonymou	000369271	281	105	386	0.272021	48	0	48	0	1	Sub	0.59	3.39			disease_causing	31	p.E734G	Passenger	SENGER/OT	damaging	Deleterious	Damaging
OVA_013_M1	METASTASIS	chr6	1.17E+08	1.17E+08	G	A	TSPYL1	UTR5	NA	NA	237	142	379	0.37467	98	1	99	0.010101	1	Clonal	0.67	3.06			polymorphism	11.14						
OVA_013_C1	PRIMARY	chr6	1.17E+08	1.17E+08	G	A	TSPYL1	UTR5	NA	NA	232	131	363	0.360882	98	1	99	0.010101	1	Clonal	0.72	3.1			polymorphism	11.14						
OVA_013_C2	PRIMARY	chr6	1.17E+08	1.17E+08	G	A	TSPYL1	UTR5	NA	NA	194	124	318	0.389937	98	1	99	0.010101	0.9	Clonal	0.87	3.05			polymorphism	11.14						
OVA_013_C3	PRIMARY	chr6	1.17E+08	1.17E+08	G	A	TSPYL1	UTR5	NA	NA	246	186	432	0.430556	98	1	99	0.010101	0.94	Clonal	0.91	3.01			polymorphism	11.14						
OVA_048_M1	METASTASIS	chr9	1.17E+08	1.17E+08	C	G	ZNF618	exonic	ynonymou	.585G,ENSG	336	120	456	0.263158	47	0	47	0	1	Clonal	0.56	3.94			disease_causing	22.4	p.A678G	Passenger	SENGER/OT	benign	Neutral	Tolerated
OVA_048_M2	METASTASIS	chr9	1.17E+08	1.17E+08	C	G	ZNF618	exonic	ynonymou	.585G,ENSG	355	76	431	0.176334	47	0	47	0	1	Clonal	0.4	3.87			disease_causing	22.4	p.A678G	Passenger	SENGER/OT	benign	Neutral	Tolerated
OVA_048_C1	PRIMARY	chr9	1.17E+08	1.17E+08	G	C	COL27A1	intronic	NA	NA	152	24	176	0.136364	11	0	11	0	0.41	Sub	0.67	1.82			polymorphism	7.464						
OVA_378_C1	PRIMARY	chr7	1.17E+08	1.17E+08	C	A	CFTR	intronic	NA	NA	418	97	515	0.18835	49	0	49	0	0.83	Sub	0.59	2.6			disease_causing	12.51						
OVA_047_C2	PRIMARY	chr11	1.17E+08	1.17E+08	G	A	DSCAML1	exonic	ynonymou	39W,ENSG	533	73	606	0.120462	130	0	130	0	0.38	Sub	0.64	2.76			disease_causing	29.4	p.R1209W	Passenger	SENGER/OT	damaging	Deleterious	Damaging
OVA_003_M1	METASTASIS	chr11	1.17E+08	1.17E+08	G	C	DSCAML1	exonic	ynonymou	000032132	804	12	816	0.014706	72	0	72	0	0.05	Sub	0.57	1.84			disease_causing	25.5	p.R257G	Passenger	SENGER/OT	damaging	Deleterious	Damaging
OVA_003_C1	PRIMARY	chr11	1.17E+08	1.17E+08	G	C	DSCAML1	exonic	ynonymou	000032132	365	203	568	0.357394	72	0	72	0	0.83	Sub	0.86	1.81			disease_causing	25.5	p.R257G	Passenger	SENGER/OT	damaging	Deleterious	Damaging
OVA_003_C2	PRIMARY	chr11	1.17E+08	1.17E+08	G	C	DSCAML1	exonic	ynonymou	000032132	447	101	548	0.184307	72	0	72	0	0.9	Sub	0.7	3.61			disease_causing	25.5	p.R257G	Passenger	SENGER/OT	damaging	Deleterious	Damaging
OVA_003_C3	PRIMARY	chr11	1.17E+08	1.17E+08	G	C	DSCAML1	exonic	ynonymou	000032132	417	166	583	0.284734	72	0	72	0	0.69	Sub	0.82	1.81			disease_causing	25.5	p.R257G	Passenger	SENGER/OT	damaging	Deleterious	Damaging
OVA_047_C1	PRIMARY	chrX	1.18E+08	1.18E+08	A	G	DOCK11	intronic	NA	NA	580	85	665	0.12782	13	0	13	0	0.55	Sub	0.6	2.91			polymorphism	6.755						
OVA_047_C2	PRIMARY	chrX	1.18E+08	1.18E+08	A	G	DOCK11	intronic	NA	NA	558	104	662	0.1571	13	0	13	0	0.65	Sub	0.64	2.76			polymorphism	6.755						
OVA_047_C3	Contralateral ovary	chrX	1.18E+08	1.18E+08	A	G	DOCK11	intronic	NA	NA	542	124	666	0.186186	13	0	13	0	0.81	Sub	0.6	2.94			polymorphism	6.755						
OVA_003_M1	METASTASIS	chr2	1.19E+08	1.19E+08	C	T	DDX18	intronic	NA	NA	450	153	603	0.253731	86	0	86	0	0.9	Sub	0.57	1.84			polymorphism	2.842						
OVA_003_M2	METASTASIS	chr2	1.19E+08	1.19E+08	C	T	DDX18	intronic	NA	NA	484	114	598	0.190635	86	0	86	0	0.83	Sub	0.46	1.81			polymorphism	2.842						
OVA_003_M3	METASTASIS	chr2	1.19E+08	1.19E+08	C	T	DDX18	intronic	NA	NA	620	38	658	0.057751	86	0	86	0	0.61	Sub	0.34	2.67			polymorphism	2.842						
OVA_003_C1	PRIMARY	chr2	1.19E+08	1.19E+08	C	T	DDX18	intronic	NA	NA	371	247	618	0.399676	86	0	86	0	0.93	Sub	0.86	1.81			polymorphism	2.842						
OVA_003_C2	PRIMARY	chr2	1.19E+08	1.19E+08	C	T	DDX18	intronic	NA	NA	369	243	612	0.397059	86	0	86	0	1	Sub	0.7	3.61			polymorphism	2.842						
OVA_003_C3	PRIMARY	chr2	1.19E+08	1.19E+08	C	T	DDX18	intronic	NA	NA	426	212	638	0.332288	86	0	86	0	0.81	Sub	0.82	1.81			polymorphism	2.842						
OVA_048_C1	PRIMARY	chr10	1.19E+08	1.19E+08	G	T	ENO4	exonic	stopgain	.E25X,ENSG	74	63	137	0.459854	101	0	101	0	1	Clonal	0.67	1.82			disease_causing_automat	35	p.E25X	Passenger			NA	NA
OVA_048_C3	PRIMARY	chr10	1.19E+08	1.19E+08	G	T	ENO4	exonic	stopgain	.E25X,ENSG	86	58	144	0.402778	101	0	101	0	1	Clonal	0.74	1.8			disease_causing_automat	35	p.E25X	Passenger			NA	NA
OVA_378_M1	METASTASIS	chr5	1.19E+08	1.19E+08	G	T	HSD17B4	UTR5	NA	NA	98	73	171	0.426901	142	0	142	0	1	Clonal	0.72	2.57			polymorphism	8.53						
OVA_378_M2	METASTASIS	chr5	1.19E+08	1.19E+08	G	T	HSD17B4	UTR5	NA	NA	96	80	176	0.454545	142	0	142	0	1	Clonal	0.85	2.57			polymorphism	8.53						
OVA_378_M3	METASTASIS	chr5	1.19E+08	1.19E+08	G	T	HSD17B4	UTR5	NA	NA	193	83	276	0.300725	142	0	142	0	0.9	Clonal	0.67	2.57			polymorphism	8.53						
OVA_378_C1	PRIMARY	chr5	1.19E+08	1.19E+08	G	T	HSD17B4	UTR5	NA	NA	150	80	230	0.347826	142	0	142	0	1	Clonal	0.59	2.6			polymorphism	8.53						
OVA_378_C2	PRIMARY	chr5	1.19E+08	1.19E+08	G	T	HSD17B4	UTR5	NA	NA	136	69	205	0.336585	142	0	142	0	1	Clonal	0.56	2.59			polymorphism	8.53						
OVA_378_C3	PRIMARY	chr5	1.19E+08	1.19E+08	G	T	HSD17B4	UTR5	NA	NA	133	40	173	0.231214	142	0	142	0	0.91	Clonal	0.51	2.6			polymorphism	8.53						
OVA_003_M1	METASTASIS	chr3	1.19E+08	1.19E+08	G	C	C3orf30	exonic	ynonymou	122P,ENSG	446	162	608	0.266447	47	0	47	0	0.94	Sub	0.57	1.84			polymorphism	3.45	p.A122P	Passenger	SENGER/OT	benign	Neutral	Tolerated
OVA_003_M2	METASTASIS	chr3	1.19E+08	1.19E+08	G	C	C3orf30	exonic	ynonymou	122P,ENSG	514	140	654	0.214067	47	0	47	0	0.93	Sub	0.46	1.81			polymorphism	3.45	p.A122P	Passenger	SENGER/OT	benign	Neutral	Tolerated
OVA_003_M3	METASTASIS	chr3	1.19E+08	1.19E+08	G	C	C3orf30	exonic	ynonymou	122P,ENSG	607	45	652	0.069018	47	0	47	0	0.73	Sub	0.34	2.67			polymorphism	3.45	p.A122P	Passenger	SENGER/OT	benign	Neutral	Tolerated
OVA_003_C1	PRIMARY	chr3	1.19E+08	1.19E+08	G	C	C3orf30	exonic	ynonymou	122P,ENSG	341	310	651	0.47619	47	0	47	0	1	Clonal	0.86	1.81			polymorphism	3.45	p.A122P	Passenger	SENGER/OT	benign	Neutral	Tolerated
OVA_003_C2	PRIMARY	chr3	1.19E+08	1.19E+08	G	C	C3orf30	exonic	ynonymou	122P,ENSG	399	265	664	0.399096	47	0	47	0	1	Clonal	0.7	3.61			polymorphism	3.45	p.A122P	Passenger	SENGER/OT	benign	Neutral	Tolerated

Sample	Type	Chr	Start	End	Ref	Alt	Gene	unc.ensGen	icFunc.ens	hange.ens	t_ref	t_alt	t_total	t_VAF	n_ref	n_alt	n_total	normal_VA	ncer_cell_f	on-based C	purity	ploidy	Cancer Gene	Driver Gene	Mutation Taster	ADD(PHRE	otein_chan	CHASM	THMM_Pr	OLYPHEN-	REDICTION	ICTION (cutoff=0.05)
OVA_003_C3	PRIMARY	chr3	1.19E+08	1.19E+08	G	C	C3orf30	exonic	ynonymou	122P,ENSG	378	282	660	0.427273	47	0	47	0	1	Clonal	0.82	1.81			polymorphism	3.45	p.A122P	Passenger	SENGER/OT	benign	Neutral	Tolerated
OVA_048_M1	METASTASIS	chrX	1.19E+08	1.19E+08	C	T	UPF3B	exonic	ynonymou	341Q,ENSG	286	125	411	0.304136	15	0	15	0	1	Sub	0.56	3.94			disease_causing	24.7	p.R354Q	Passenger	SENGER/OT	damaging	Neutral	Damaging
OVA_048_M2	METASTASIS	chrX	1.19E+08	1.19E+08	C	T	UPF3B	exonic	ynonymou	341Q,ENSG	390	128	518	0.247104	15	0	15	0	1	Sub	0.4	3.87			disease_causing	24.7	p.R354Q	Passenger	SENGER/OT	damaging	Neutral	Damaging
OVA_048_M3	METASTASIS	chrX	1.19E+08	1.19E+08	C	T	UPF3B	exonic	ynonymou	341Q,ENSG	314	13	327	0.039755	15	0	15	0	0.35	Sub	0.59	3.39			disease_causing	24.7	p.R354Q	Passenger	SENGER/OT	damaging	Neutral	Damaging
OVA_048_C1	PRIMARY	chrX	1.19E+08	1.19E+08	C	T	UPF3B	exonic	ynonymou	341Q,ENSG	204	80	284	0.28169	15	0	15	0	0.84	Sub	0.67	1.82			disease_causing	24.7	p.R354Q	Passenger	SENGER/OT	damaging	Neutral	Damaging
OVA_048_C2	PRIMARY	chrX	1.19E+08	1.19E+08	C	T	UPF3B	exonic	ynonymou	341Q,ENSG	285	143	428	0.334112	15	0	15	0	0.93	Sub	0.72	1.85			disease_causing	24.7	p.R354Q	Passenger	SENGER/OT	damaging	Neutral	Damaging
OVA_048_C3	PRIMARY	chrX	1.19E+08	1.19E+08	C	T	UPF3B	exonic	ynonymou	341Q,ENSG	261	103	364	0.282967	15	0	15	0	0.76	Sub	0.74	1.8			disease_causing	24.7	p.R354Q	Passenger	SENGER/OT	damaging	Neutral	Damaging
OVA_047_C3	Contralateral ovary	chr3	1.19E+08	1.19E+08	T	A	POGLUT1	exonic	nonymous S	000004866	522	158	680	0.232353	61	0	61	0	1	Clonal	0.6	2.94			disease_causing	13.15		Passenger			Neutral	Tolerated
OVA_048_C3	PRIMARY	chr11	1.19E+08	1.19E+08	C	T	RNF26	cRNA_exon	NA	NA	231	14	245	0.057143	28	0	28	0	0.15	Sub	0.74	1.8			polymorphism	8.562						
OVA_047_M1	METASTASIS	chrX	1.19E+08	1.19E+08	G	T	RHOXF1	exonic	stopgain	T00000217	483	110	593	0.185497	58	0	58	0	0.78	Sub	0.47	2.01			disease_causing_automat	22.9	p.S31X	Passenger			NA	NA
OVA_047_M2	METASTASIS	chrX	1.19E+08	1.19E+08	G	T	RHOXF1	exonic	stopgain	T00000217	505	113	618	0.182848	58	0	58	0	0.82	Sub	0.44	2.01			disease_causing_automat	22.9	p.S31X	Passenger			NA	NA
OVA_047_M3	METASTASIS	chrX	1.19E+08	1.19E+08	G	T	RHOXF1	exonic	stopgain	T00000217	234	48	282	0.170213	58	0	58	0	0.74	Sub	0.6	3.02			disease_causing_automat	22.9	p.S31X	Passenger			NA	NA
OVA_047_C1	PRIMARY	chrX	1.19E+08	1.19E+08	G	T	RHOXF1	exonic	stopgain	T00000217	417	244	661	0.369138	58	0	58	0	1	Clonal	0.6	2.91			disease_causing_automat	22.9	p.S31X	Passenger			NA	NA
OVA_047_C2	PRIMARY	chrX	1.19E+08	1.19E+08	G	T	RHOXF1	exonic	stopgain	T00000217	404	239	643	0.371695	58	0	58	0	1	Clonal	0.64	2.76			disease_causing_automat	22.9	p.S31X	Passenger			NA	NA
OVA_047_C3	Contralateral ovary	chrX	1.19E+08	1.19E+08	G	T	RHOXF1	exonic	stopgain	T00000217	396	257	653	0.393568	58	0	58	0	1	Clonal	0.6	2.94			disease_causing_automat	22.9	p.S31X	Passenger			NA	NA
OVA_048_M1	METASTASIS	chr3	1.19E+08	1.19E+08	C	T	MAATS1	intronic	NA	NA	122	4	126	0.031746	20	0	20	0	0.18	Sub	0.56	3.94			polymorphism	0.411				damaging		
OVA_048_M2	METASTASIS	chr3	1.19E+08	1.19E+08	C	T	MAATS1	intronic	NA	NA	154	8	162	0.049383	20	0	20	0	0.34	Sub	0.4	3.87			polymorphism	0.411				damaging		
OVA_048_M3	METASTASIS	chr3	1.19E+08	1.19E+08	C	T	MAATS1	intronic	NA	NA	152	39	191	0.204188	20	0	20	0	1	Sub	0.59	3.39			polymorphism	0.411				damaging		
OVA_013_C3	PRIMARY	chr3	1.2E+08	1.2E+08	A	G	GSK3B	UTR3	NA	NA	105	45	150	0.3	26	0	26	0	0.96	Clonal	0.91	3.01			disease_causing	18.48						
OVA_378_M1	METASTASIS	chr12	1.2E+08	1.2E+08	-	AGGC	SRRM4	intronic	NA	NA	11	26	37	0.702703	13	0	13		1	Clonal	0.72	2.57										
OVA_378_M2	METASTASIS	chr12	1.2E+08	1.2E+08	-	AGGC	SRRM4	intronic	NA	NA	2	20	22	0.909091	13	0	13		1	Clonal	0.85	2.57										
OVA_378_M3	METASTASIS	chr12	1.2E+08	1.2E+08	-	AGGC	SRRM4	intronic	NA	NA	8	43	51	0.843137	13	0	13		1	Clonal	0.67	2.57										
OVA_378_C1	PRIMARY	chr12	1.2E+08	1.2E+08	-	AGGC	SRRM4	intronic	NA	NA	6	39	45	0.866667	13	0	13		1	Sub	0.59	2.6										
OVA_378_C2	PRIMARY	chr12	1.2E+08	1.2E+08	-	AGGC	SRRM4	intronic	NA	NA	7	23	30	0.766667	13	0	13		1	Sub	0.56	2.59										
OVA_378_C3	PRIMARY	chr12	1.2E+08	1.2E+08	-	AGGC	SRRM4	intronic	NA	NA	6	11	17	0.647059	13	0	13		1	Sub	0.51	2.6										
OVA_047_M1	METASTASIS	chr2	1.2E+08	1.2E+08	G	T	EN1	exonic	nonymous S	000029520	33	15	48	0.3125	21	0	21	0	1	Clonal	0.47	2.01			polymorphism	9.915		Passenger			Neutral	Tolerated
OVA_047_M2	METASTASIS	chr2	1.2E+08	1.2E+08	G	T	EN1	exonic	nonymous S	000029520	49	19	68	0.279412	21	0	21	0	1	Clonal	0.44	2.01			polymorphism	9.915		Passenger			Neutral	Tolerated
OVA_047_M3	METASTASIS	chr2	1.2E+08	1.2E+08	G	T	EN1	exonic	nonymous S	000029520	23	7	30	0.233333	21	0	21	0	1	Clonal	0.6	3.02			polymorphism	9.915		Passenger			Neutral	Tolerated
OVA_378_M1	METASTASIS	chr1	1.2E+08	1.2E+08	G	T	HSD3B2	exonic	ynonymou	000043374	503	118	621	0.190016	109	1	110	0.009091	0.91	Sub	0.72	2.57			disease_causing	17.93	p.L179F	Passenger	SENGER/OT	damaging	Deleterious	Tolerated
OVA_378_M2	METASTASIS	chr1	1.2E+08	1.2E+08	G	T	HSD3B2	exonic	ynonymou	000043374	471	144	615	0.234146	109	1	110	0.009091	1	Sub	0.85	2.57			disease_causing	17.93	p.L179F	Passenger	SENGER/OT	damaging	Deleterious	Tolerated
OVA_378_M3	METASTASIS	chr1	1.2E+08	1.2E+08	G	T	HSD3B2	exonic	ynonymou	000043374	560	113	673	0.167905	109	1	110	0.009091	0.84	Sub	0.67	2.57			disease_causing	17.93	p.L179F	Passenger	SENGER/OT	damaging	Deleterious	Tolerated
OVA_378_C1	PRIMARY	chr1	1.2E+08	1.2E+08	G	T	HSD3B2	exonic	ynonymou	000043374	540	109	649	0.167951	109	1	110	0.009091	0.91	Clonal	0.59	2.6			disease_causing	17.93	p.L179F	Passenger	SENGER/OT	damaging	Deleterious	Tolerated
OVA_378_C2	PRIMARY	chr1	1.2E+08	1.2E+08	G	T	HSD3B2	exonic	ynonymou	000043374	502	106	608	0.174342	109	1	110	0.009091	0.97	Clonal	0.56	2.59			disease_causing	17.93	p.L179F	Passenger	SENGER/OT	damaging	Deleterious	Tolerated
OVA_378_C3	PRIMARY	chr1	1.2E+08	1.2E+08	G	T	HSD3B2	exonic	ynonymou	000043374	492	120	612	0.196078	109	1	110	0.009091	1	Clonal	0.51	2.6			disease_causing	17.93	p.L179F	Passenger	SENGER/OT	damaging	Deleterious	Tolerated
OVA_048_M1	METASTASIS	chr2	1.2E+08	1.2E+08	C	T	STEAP3	exonic	nonymous S	L282L,ENSG	300	134	434	0.308756	52	0	52	0	1	Clonal	0.56	3.94			disease_causing	18.75		Passenger			Neutral	Tolerated
OVA_048_M2	METASTASIS	chr2	1.2E+08	1.2E+08	C	T	STEAP3	exonic	nonymous S	L282L,ENSG	345	61	406	0.150246	52	0	52	0	1	Clonal	0.4	3.87			disease_causing	18.75		Passenger			Neutral	Tolerated
OVA_048_C2	PRIMARY	chr2	1.21E+08	1.21E+08	A	G	EPB41L5	intronic	NA	NA	431	160	591	0.270728	24	0	24	0	0.75	Sub	0.72	1.85			polymorphism	7.381						
OVA_047_C1	PRIMARY	chr11	1.21E+08	1.21E+08	G	A	TECTA	exonic	ynonymou	532N,ENSG	526	135	661	0.204236	104	0	104	0	0.68	Sub	0.6	2.91			disease_causing	26.7	p.D1532N	Passenger	SENGER/OT	damaging	Neutral	Tolerated
OVA_047_C2	PRIMARY	chr11	1.21E+08	1.21E+08	G	A	TECTA	exonic	ynonymou	532N,ENSG	549	113	662	0.170695	104	0	104	0	0.53	Sub	0.64	2.76			disease_causing	26.7	p.D1532N	Passenger	SENGER/OT	damaging	Neutral	Tolerated
OVA_047_C3	Contralateral ovary	chr11	1.21E+08	1.21E+08	G	A	TECTA	exonic	ynonymou	532N,ENSG	498	153	651	0.235023	104	0	104	0	0.79	Sub	0.6	2.94			disease_causing	26.7	p.D1532N	Passenger	SENGER/OT	damaging	Neutral	Tolerated
OVA_365_M1	METASTASIS	chr3	1.21E+08	1.21E+08	C	T	FBXO40	exonic	nonymous S	000033804	312	201	513	0.391813	64	0	64	0	0.93	Clonal	0.85	1.87			disease_causing	11.27		Passenger			Neutral	Tolerated
OVA_365_M2	METASTASIS	chr3	1.21E+08	1.21E+08	C	T	FBXO40	exonic	nonymous S	000033804	318	77	395	0.194937	64	0	64	0	0.97	Clonal	0.4	1.81			disease_causing	11.27		Passenger			Neutral	Tolerated
OVA_365_M3	METASTASIS	chr3	1.21E+08	1.21E+08	C	T	FBXO40	exonic	nonymous S	000033804	315	206	521	0.395393	64	0	64	0	0.97	Clonal	0.82	1.89			disease_causing	11.27		Passenger			Neutral	Tolerated
OVA_365_C1	Contralateral ovary	chr3	1.21E+08	1.21E+08	C	T	FBXO40	exonic	nonymous S	000033804	303	91	394	0.230964	64	0	64	0	1	Clonal	0.42	1.83			disease_causing	11.27		Passenger			Neutral	Tolerated
OVA_365_C2	PRIMARY	chr3	1.21E+08	1.21E+08	C	T	FBXO40	exonic	nonymous S	000033804	352	125	477	0.262055	64	0	64	0	0.85	Sub	0.62	1.92			disease_causing	11.27		Passenger			Neutral	Tolerated
OVA_047_C1	PRIMARY	chr8	1.21E+08	1.21E+08	G	T	COL14A1	intronic	NA	NA	606	48	654	0.073394	50	0	50	0	0.61	Sub	0.6	2.91			disease_causing	16.83						
OVA_013_M1	METASTASIS	chr10	1.22E+08	1.22E+08	T	C	INPP5F	exonic	ynonymou	V51A,ENSG	490	151	641	0.235569	77	1	78	0.012821	0.94	Clonal	0.67	3.06			disease_causing	23.1	p.V51A	Passenger	SENGER/OT	benign	Deleterious	Damaging
OVA_013_C1	PRIMARY	chr10	1.22E+08	1.22E+08	T	C	INPP5F	exonic	ynonymou	V51A,ENSG	419	185	604	0.306291	77	1	78	0.012821	1	Sub	0.72	3.1			disease_causing	23.1	p.V51A	Passenger	SENGER/OT	benign	Deleterious	Damaging
OVA_013_C2	PRIMARY	chr10	1.22E+08	1.22E+08	T	C	INPP5F	exonic	ynonymou	V51A,ENSG	442	155	597	0.259631	77	1	78	0.012821	0.86	Sub	0.87	3.05			disease_causing	23.1	p.V51A	Passenger	SENGER/OT	benign	Deleterious	Damaging
OVA_013_C3	PRIMARY	chr10	1.22E+08	1.22E+08	T	C	INPP5F	exonic	ynonymou	V51A,ENSG	459	174	633	0.274882	77	1	78	0.012821	0.88	Sub	0.91	3.01			disease_causing	23.1	p.V51A	Passenger	SENGER/OT	benign	Deleterious	Damaging
OVA_003_M1	METASTASIS	chr3	1.22E+08	1.22E+08	G	A	ILDR1	exonic	nonymous S	G85G,ENSG	200	89	289	0.307958	71	0	71	0	1	Sub	0.57	1.84			disease_causing	12.42		Passenger			Neutral	Tolerated
OVA_003_M2	METASTASIS	chr3	1.22E+08	1.22E+08	G	A	ILDR1	exonic	nonymous S	G85G,ENSG	302	80	382	0.209424	71	0	71	0	0.91	Sub	0.46	1.81			disease_causing	12.42		Passenger			Neutral	Tolerated
OVA_003_M3	METASTASIS	chr3	1.22E+08	1.22E+08	G	A	ILDR1	exonic	nonymous S	G85G,ENSG	345	29	374	0.07754	71	0	71	0	0.82	Sub	0.34	2.67			disease_causing	12.42		Passenger			Neutral	Tolerated
OVA_003_C1	PRIMARY	chr3	1.22E+08	1.22E+08	G	A	ILDR1	exonic	nonymous S	G85G,ENSG	199	178	377	0.472149	71	0	71	0	1	Clonal	0.86	1.81			disease_causing	12.42		Passenger			Neutral	Tolerated
OVA_003_C2	PRIMARY	chr3	1.22E+08	1.22E+08	G	A	ILDR1	exonic	nonymous S	G85G,ENSG	241	180	421	0.427553	71	0	71	0	1	Clonal	0.7	3.61			disease_causing	12.42		Passenger			Neutral	Tolerated
OVA_003_C3	PRIMARY	chr3	1.22E+08	1.22E+08	G	A	ILDR1	exonic	nonymous S	G85G,ENSG	269	217	486	0.446502	71	0	71	0	1	Clonal	0.82	1.81			disease_causing	12.42		Passenger			Neutral	Tolerated
OVA_013_C1	PRIMARY	chr5	1.22E+08	1.22E+08	C	T	SNCAIP	exonic	nonymous S	000379533	1120	762	1883	0.404673	56	0	56	0	1	Sub	0.72	3.1			disease_causing	10.93		Passenger			Neutral	Tolerated
OVA_013_C2	PRIMARY	chr5	1.22E+08	1.22E+08	C	T	SNCAIP	exonic	nonymous S	000379533	352	251	603	0.416252	56	0	56	0	0.96	Sub	0.87	3.05			disease_causing	10.93		Passenger			Neutral	Tolerated
OVA_013_C3	PRIMARY	chr5	1.22E+08	1.22E+08	C	T	SNCAIP	exonic	nonymous S	000379533	388	261	649	0.402157	56	0	56	0	0.88	Sub	0.91	3.01			disease_causing	10.93		Passenger			Neutral	Tolerated
OVA_048_M1	METASTASIS	chr2	1.22E+08	1.22E+08	G	A	CLASP1	intronic	NA	NA	225	97	322	0.301242	46	0	46	0	1	Clonal	0.56	3.94			disease_causing	16.53						
OVA_048_M2	METASTASIS	chr2	1.22E+08	1.22E+08	G	A	CLASP1	intronic	NA	NA	262	73	335	0.21791	46	0	46	0	1	Clonal	0.4	3.87			disease_causing	16.53						
OVA_048_M3	METASTASIS	chr2	1.22E+08	1.22E+08	G	A	CLASP1	intronic	NA	NA	184	117	301	0.388704	46	0	46	0	1	Clonal	0.59	3.39			disease_causing	16.53						
OVA_048_C1	PRIMARY	chr2	1.22E+08	1.22E+08	G	A	CLASP1	intronic	NA	NA	100	50	150	0.333333	46	0	46	0	1	Clonal	0.67	1.82			disease_causing	16.53						
OVA_048_C2	PRIMARY	chr2	1.22E+08	1.22E+08	G	A	CLASP1	intronic	NA	NA	239	124	363	0.341598	46	0	46	0	0.95	Clonal	0.72	1.85			disease_causing	16.53						
OVA_048_C3	PRIMARY	chr2	1.22E+08	1.22E+08	G	A	CLASP1	intronic	NA	NA	133	73	206	0.354369	46	0	46	0	0.96	Clonal	0.74	1.8			disease_causing	16.53						
OVA_047_M1	METASTASIS	chr12	1.22E+08	1.22E+08	G	C	SETD1B	splicing	NA	NA	430	117	547	0.213894	63	0	63	0	0.9	Clonal	0.47	2.01	Yes		disease_causing	34		Passenger				
OVA_047_M2	METASTASIS	chr12	1.22E+08	1.22E+08	G	C	SETD1B	splicing	NA	NA	432	119	551	0.215971	63	0	63	0	0.97	Clonal	0.44	2.01	Yes		disease_causing	34		Passenger				
OVA_047_M3	METASTASIS	chr12	1.22E+08	1.22E+08	G	C	SETD1B	splicing	NA	NA	137	60	197	0.304569	63	0	63	0	1	Clonal	0.6	3.02	Yes		disease_causing	34		Passenger				
OVA_378_M1	METASTASIS	chr3	1.22E+08	1.22E+08	C	T	DTX3L	exonic	nonymous S	181R,ENSG	567	109	676	0.161243	47	1	48	0.020833	0.93	Clonal	0.72	2.57			polymorphism	1.13		Passenger			Neutral	Tolerated
OVA_378_M2	METASTASIS	chr3	1.22E+08	1.22E+08	C	T	DTX3L	exonic	nonymous S	181R,ENSG	543	132	675	0.195556	47	1	48	0.020833	1	Clonal	0.85	2.57			polymorphism	1.13		Passenger			Neutral	Tolerated
OVA_378_M3	METASTASIS	chr3	1.22E+08	1.22E+08	C	T	DTX3L	exonic	nonymous S	181R,ENSG	623	140	763	0.183486	47	1	48	0.020833	1	Clonal	0.67	2.57			polymorphism	1.13		Passenger			Neutral	Tolerated
OVA_378_C1	PRIMARY	chr3	1.22E+08	1.22E+08	C	T	DTX3L	exonic	nonymous S	181R,ENSG	593	119	712	0.167135	47	1	48	0.020833	1	Clonal	0.59	2.6			polymorphism	1.13		Passenger			Neutral	Tolerated
OVA_378_C2	PRIMARY	chr3	1.22E+08	1.22E+08	C	T	DTX3L	exonic	nonymous S	181R,ENSG	601	80	681	0.117474	47	1	48	0.020833	0.89	Clonal	0.56	2.59			polymorphism	1.13		Passenger			Neutral	Tolerated
OVA_378_C3	PRIMARY	chr3	1.22E+08	1.22E+08	C	T	DTX3L	exonic	nonymous S	181R,ENSG	596	73	669	0.109118	47	1	48	0.020833	0.86	Clonal	0.51	2.6			polymorphism	1.13		Passenger			Neutral	Tolerated
OVA_365_M1	METASTASIS	chr3	1.23E+08	1.23E+08	G	T	SEMA5B	exonic	ynonymou	Q96K,ENSG	174	154	328	0.469512	35	0	35	0	1	Clonal	0.85	1.87			polymorphism	17.84	p.Q150K	Passenger	SENGER/OT	benign	Neutral	Tolerated
OVA_365_M2	METASTASIS	chr3	1.23E+08	1.23E+08	G	T	SEMA5B	exonic	ynonymou	Q96K,ENSG	278	65	343	0.189504	35	0	35	0	0.94	Clonal	0.4	1.81			polymorphism	17.84	p.Q150K	Passenger	SENGER/OT	benign	Neutral	Tolerated
OVA_365_M3	METASTASIS	chr3	1.23E+08	1.23E+08	G	T	SEMA5B	exonic	ynonymou	Q96K,ENSG	270	153	423	0.361702	35	0	35	0	0.88	Clonal	0.82	1.89			polymorphism	17.84	p.Q150K	Passenger	SENGER/OT	benign	Neutral	Tolerated
OVA_365_C1	Contralateral ovary	chr3	1.23E+08	1.23E+08	G	T	SEMA5B	exonic	ynonymou	Q96K,ENSG	298	88	386	0.227979	35	0	35	0	1	Clonal	0.42	1.83			polymorphism	17.84	p.Q150K	Passenger	SENGER/OT	benign	Neutral	Tolerated
OVA_365_C2	PRIMARY	chr3	1.23E+08	1.23E+08	G	T	SEMA5B	exonic	ynonymou	Q96K,ENSG	282	126	408	0.308824	35	0	35	0	1	Clonal	0.62	1.92			polymorphism	17.84	p.Q150K	Passenger	SENGER/OT	benign	Neutral	Tolerated
OVA_365_M1	METASTASIS	chr11	1.23E+08	1.23E+08	A	T	UBASH3B	exonic	ynonymou	000284273	347	234	581	0.402754	49	0	49	0	0.95	Clonal	0.85	1.87			disease_causing	23.8	p.H479L	Passenger	SENGER/OT	benign	Deleterious	Tolerated
OVA_365_M2	METASTASIS	chr11	1.23E+08	1.23E+08	A	T	UBASH3B	exonic	ynonymou	000284273	386	88	474	0.185654	49	0	49	0	0.92	Clonal	0.4	1.81			disease_causing	23.8	p.H479L	Passenger	SENGER/OT	benign	Deleterious	Tolerated
OVA_365_M3	METASTASIS	chr11	1.23E+08	1.23E+08	A	T	UBASH3B	exonic	ynonymou	000284273	346	206	552	0.373188	49	0	49	0	0.91	Clonal	0.82	1.89			disease_causing	23.8	p.H479L	Passenger	SENGER/OT	benign	Deleterious	Tolerated
OVA_365_C1	Contralateral ovary	chr11	1.23E+08	1.23E+08	A	T	UBASH3B	exonic	ynonymou	000284273	381	106	487	0.217659	49	0	49	0	1	Clonal	0.42	1.83			disease_causing	23.8	p.H479L	Passenger	SENGER/OT	benign	Deleterious	Tolerated
OVA_365_C2	PRIMARY	chr11	1.23E+08	1.23E+08	A	T	UBASH3B	exonic	ynonymou	000284273	444	119	563	0.211368	49	0	49	0	0.9	Clonal	0.62	1.92			disease_causing	23.8	p.H479L	Passenger	SENGER/OT	benign	Deleterious	Tolerated
OVA_003_M1	METASTASIS	chr3	1.23E+08	1.23E+08	T	A	ADCY5	exonic	ynonymou	Y909F,ENSG	169	64	233	0.274678	39	0	39	0	0.97	Sub	0.57	1.84			disease_causing	33	p.Y909F	Passenger	SENGER/OT	damaging	Deleterious	Damaging
OVA_003_M2	METASTASIS	chr3	1.23E+08	1.23E+08	T	A	ADCY5	exonic	ynonymou	Y909F,ENSG	219	52	271	0.191882	39	0	39	0	0.84	Sub	0.46	1.81			disease_causing	33	p.Y909F	Passenger	SENGER/OT	damaging	Deleterious	Damaging
OVA_003_M3	METASTASIS	chr3	1.23E+08	1.23E+08	T	A	ADCY5	exonic	ynonymou	Y909F,ENSG	290	24	314	0.076433	39	0	39	0	0.81	Sub	0.34	2.67			disease_causing	33	p.Y909F	Passenger	SENGER/OT	damaging	Deleterious	Damaging
OVA_003_C1	PRIMARY	chr3	1.23E+08	1.23E+08	T	A	ADCY5	exonic	ynonymou	Y909F,ENSG	185	153	338	0.452663	39	0	39	0	1	Sub	0.86	1.81			disease_causing	33	p.Y909F	Passenger	SENGER/OT	damaging	Deleterious	Damaging
OVA_003_C2	PRIMARY	chr3	1.23E+08	1.23E+08	T	A	ADCY5	exonic	ynonymou	Y909F,ENSG	210	164	374	0.438503	39	0	39	0	1	Sub	0.7	3.61			disease_causing	33	p.Y909F	Passenger	SENGER/OT	damaging	Deleterious	Damaging
OVA_003_C3	PRIMARY	chr3	1.23E+08	1.23E+08	T	A	ADCY5	exonic	ynonymou	Y909F,ENSG	280	140	420	0.333333	39	0	39	0	0.81	Sub	0.82	1.81			disease_causing	33	p.Y909F	Passenger	SENGER/OT	damaging	Deleterious	Damaging
OVA_013_M1	METASTASIS	chrX	1.23E+08	1.23E+08	A	T	STAG2	UTR5	NA	NA	250	322	572	0.562937	22	0	22	0	1	Clonal	0.67	3.06	Yes		disease_causing	13.27						
OVA_013_C1	PRIMARY	chrX	1.23E+08	1.23E+08	A	T	STAG2	UTR5	NA	NA	206	355	561	0.632799	22	0	22	0	1	Clonal	0.72	3.1	Yes		disease_causing	13.27						
OVA_013_C2	PRIMARY	chrX	1.23E+08	1.23E+08	A	T	STAG2	UTR5	NA	NA	184	277	461	0.600868	22	0	22	0	1	Clonal	0.87	3.05	Yes		disease_causing	13.27						
OVA_013_C3	PRIMARY	chrX	1.23E+08	1.23E+08	A	T	STAG2	UTR5	NA	NA	215	345	560	0.616071	22	0	22	0	1	Clonal	0.91	3.01	Yes		disease_causing	13.27						
OVA_378_M1	METASTASIS	chr7	1.23E+08	1.23E+08	T	C	WASL	exonic	ynonymou	T00000223	37	130	167	0.778443	172	0	172	0	1	Clonal	0.72	2.57			disease_causing	24	p.N16S	Passenger	no weights	damaging	Deleterious	Damaging
OVA_378_M2	METASTASIS	chr7	1.23E+08	1.23E+08	T	C	WASL	exonic	ynonymou	T00000223	14	143	157	0.910828	172	0	172	0	1	Clonal	0.85	2.57			disease_causing	24	p.N16S	Passenger	no weights	damaging	Deleterious	Damaging
OVA_378_M3	METASTASIS	chr7	1.23E+08	1.23E+08	T	C	WASL	exonic	ynonymou	T00000223	78	203	281	0.72242	172	0	172	0	1	Clonal	0.67	2.57			disease_causing	24	p.N16S	Passenger	no weights	damaging	Deleterious	Damaging
OVA_378_C1	PRIMARY	chr7	1.23E+08	1.23E+08	T	C	WASL	exonic	ynonymou	T00000223	149	143	292	0.489726	172	0	172	0	1	Clonal	0.59	2.6			disease_causing	24	p.N16S	Passenger	no weights	damaging	Deleterious	Damaging
OVA_378_C2	PRIMARY	chr7	1.23E+08	1.23E+08	T	C	WASL	exonic	ynonymou	T00000223	165	134	299	0.448161	172	0	172	0	1	Clonal	0.56	2.59			disease_causing	24	p.N16S	Passenger	no weights	damaging	Deleterious	Damaging
OVA_378_C3	PRIMARY	chr7	1.23E+08	1.23E+08	T	C	WASL	exonic	ynonymou	T00000223	147	99	246	0.402439	172	0	172	0	1	Clonal	0.51	2.6			disease_causing	24	p.N16S	Passenger	no weights	damaging	Deleterious	Damaging

Sample	Type	Chr	Start	End	Ref	Alt	Gene	unc.ensGer	icFunc.ens	hange.ens(	t_ref	t_alt	t_total	t_VAF	n_ref	n_alt	n_total	ormal_VA	ncer_cell_f	on-based C	purity	ploidy	Cancer Gene	Driver Gene	Mutation Taster	ADD(PHRE	otein_chan	CHASM	ATHMM_Pr	OLYPHEN-	REDICTION	ICTION (cutoff=0.05)
OVA_048_M1	METASTASIS	chr11	1.23E+08	1.23E+08	G	A	GRAMD1B	UTR5	NA	NA	339	260	599	0.434057	27	0	27	0	1	Clonal	0.56	3.94			polymorphism	2.699						
OVA_048_M2	METASTASIS	chr11	1.23E+08	1.23E+08	G	A	GRAMD1B	UTR5	NA	NA	383	214	597	0.358459	27	0	27	0	1	Clonal	0.4	3.87			polymorphism	2.699						
OVA_048_M3	METASTASIS	chr11	1.23E+08	1.23E+08	G	A	GRAMD1B	UTR5	NA	NA	352	255	607	0.420099	27	0	27	0	1	Clonal	0.59	3.39			polymorphism	2.699						
OVA_378_M1	METASTASIS	chr6	1.24E+08	1.24E+08	A	G	TRDN	intronic	NA	NA	75	48	123	0.390244	54	0	54	0	1	Clonal	0.72	2.57			polymorphism	1.266						
OVA_378_M2	METASTASIS	chr6	1.24E+08	1.24E+08	A	G	TRDN	intronic	NA	NA	56	44	100	0.44	54	0	54	0	1	Clonal	0.85	2.57			polymorphism	1.266						
OVA_378_M3	METASTASIS	chr6	1.24E+08	1.24E+08	A	G	TRDN	intronic	NA	NA	152	78	230	0.33913	54	0	54	0	1	Clonal	0.67	2.57			polymorphism	1.266						
OVA_378_C1	PRIMARY	chr6	1.24E+08	1.24E+08	A	G	TRDN	intronic	NA	NA	112	42	154	0.272727	54	0	54	0	0.93	Sub	0.59	2.6			polymorphism	1.266						
OVA_378_C2	PRIMARY	chr6	1.24E+08	1.24E+08	A	G	TRDN	intronic	NA	NA	95	36	131	0.274809	54	0	54	0	0.97	Sub	0.56	2.59			polymorphism	1.266						
OVA_378_C3	PRIMARY	chr6	1.24E+08	1.24E+08	A	G	TRDN	intronic	NA	NA	110	26	136	0.191176	54	0	54	0	0.75	Sub	0.51	2.6			polymorphism	1.266						
OVA_048_C3	PRIMARY	chrX	1.24E+08	1.24E+08	T	C	TENM1	exonic	onymous S	513G,ENSG	449	52	501	0.103792	38	0	38	0	0.28	Sub	0.74	1.8			disease_causing	11.07		Passenger			Neutral	Tolerated
OVA_047_C1	PRIMARY	chr8	1.24E+08	1.24E+08	C	A	ZHX2	exonic	stopgain	000031439	559	70	629	0.111288	95	1	96	0.010417	0.93	Sub	0.6	2.91			disease_causing_automat	38	p.C390X	Passenger			NA	NA
OVA_047_C2	PRIMARY	chr8	1.24E+08	1.24E+08	C	A	ZHX2	exonic	stopgain	000031439	530	84	614	0.136808	95	1	96	0.010417	0.84	Sub	0.64	2.76			disease_causing_automat	38	p.C390X	Passenger			NA	NA
OVA_047_C3	Contralateral ovary	chr8	1.24E+08	1.24E+08	C	A	ZHX2	exonic	stopgain	000031439	539	61	600	0.101667	95	1	96	0.010417	0.75	Sub	0.6	2.94			disease_causing_automat	38	p.C390X	Passenger			NA	NA
OVA_048_C1	PRIMARY	chr10	1.24E+08	1.24E+08	G	A	TACC2	UTR3	NA	NA	388	21	409	0.051345	82	1	83	0.012048	0.15	Sub	0.67	1.82			disease_causing	14.18						
OVA_013_M1	METASTASIS	chr11	1.24E+08	1.24E+08	T	C	VWA5A	intronic	NA	NA	440	128	568	0.225352	48	0	48	0	0.9	Clonal	0.67	3.06			polymorphism	2.462						
OVA_013_C1	PRIMARY	chr11	1.24E+08	1.24E+08	T	C	VWA5A	intronic	NA	NA	411	169	580	0.291379	48	0	48	0	1	Clonal	0.72	3.1			polymorphism	2.462						
OVA_013_C2	PRIMARY	chr11	1.24E+08	1.24E+08	T	C	VWA5A	intronic	NA	NA	431	159	590	0.269492	48	0	48	0	0.89	Clonal	0.87	3.05			polymorphism	2.462						
OVA_013_C3	PRIMARY	chr11	1.24E+08	1.24E+08	T	C	VWA5A	intronic	NA	NA	425	180	605	0.297521	48	0	48	0	0.95	Clonal	0.91	3.01			polymorphism	2.462						
OVA_047_M1	METASTASIS	chr5	1.24E+08	1.24E+08	C	G	ZNF608	exonic	ynonymous	189Q,ENSG	191	91	282	0.322695	154	0	154	0	1	Sub	0.47	2.01			polymorphism	23.3	p.E189Q	Passenger	CANCER	benign	Neutral	Damaging
OVA_047_M2	METASTASIS	chr5	1.24E+08	1.24E+08	C	G	ZNF608	exonic	ynonymous	189Q,ENSG	290	89	379	0.234828	154	0	154	0	0.82	Sub	0.44	2.01			polymorphism	23.3	p.E189Q	Passenger	CANCER	benign	Neutral	Damaging
OVA_047_M3	METASTASIS	chr5	1.24E+08	1.24E+08	C	G	ZNF608	exonic	ynonymous	189Q,ENSG	108	29	137	0.211679	154	0	154	0	0.71	Sub	0.6	3.02			polymorphism	23.3	p.E189Q	Passenger	CANCER	benign	Neutral	Damaging
OVA_047_M2	METASTASIS	chr12	1.24E+08	1.24E+08	C	G	TCTN2	intronic	NA	NA	592	91	683	0.133236	37	0	37	0	0.6	Sub	0.44	2.01			polymorphism	13.02						
OVA_047_M3	METASTASIS	chr12	1.24E+08	1.24E+08	C	G	TCTN2	intronic	NA	NA	473	43	516	0.083333	37	0	37	0	0.44	Sub	0.6	3.02			polymorphism	13.02						
OVA_003_M1	METASTASIS	chr8	1.24E+08	1.24E+08	C	A	FAM83A	cRNA_exon	NA	NA	394	79	473	0.167019	74	0	74	0	0.93	Sub	0.57	1.84			polymorphism	1.212					Neutral	NA
OVA_003_M2	METASTASIS	chr8	1.24E+08	1.24E+08	C	A	FAM83A	cRNA_exon	NA	NA	446	80	526	0.152091	74	0	74	0	0.82	Sub	0.46	1.81			polymorphism	1.212					Neutral	NA
OVA_003_M3	METASTASIS	chr8	1.24E+08	1.24E+08	C	A	FAM83A	cRNA_exon	NA	NA	337	24	361	0.066482	74	0	74	0	1	Sub	0.34	2.67			polymorphism	1.212					Neutral	NA
OVA_003_C1	PRIMARY	chr8	1.24E+08	1.24E+08	C	A	FAM83A	cRNA_exon	NA	NA	434	134	568	0.235915	74	0	74	0	1	Sub	0.86	1.81			polymorphism	1.212					Neutral	NA
OVA_003_C2	PRIMARY	chr8	1.24E+08	1.24E+08	C	A	FAM83A	cRNA_exon	NA	NA	460	143	603	0.237148	74	0	74	0	1	Sub	0.7	3.61			polymorphism	1.212					Neutral	NA
OVA_003_C3	PRIMARY	chr8	1.24E+08	1.24E+08	C	A	FAM83A	cRNA_exon	NA	NA	486	109	595	0.183193	74	0	74	0	0.63	Sub	0.82	1.81			polymorphism	1.212					Neutral	NA
OVA_047_C1	PRIMARY	chr8	1.24E+08	1.24E+08	C	G	ATAD2	intronic	NA	NA	705	85	790	0.107595	35	0	35	0	0.9	Sub	0.6	2.91			polymorphism	1.275						
OVA_047_C2	PRIMARY	chr8	1.24E+08	1.24E+08	C	G	ATAD2	intronic	NA	NA	662	104	766	0.13577	35	0	35	0	0.83	Sub	0.64	2.76			polymorphism	1.275						
OVA_047_C3	Contralateral ovary	chr8	1.24E+08	1.24E+08	C	G	ATAD2	intronic	NA	NA	678	82	760	0.107895	35	0	35	0	0.79	Sub	0.6	2.94			polymorphism	1.275						
OVA_047_C1	PRIMARY	chr3	1.25E+08	1.25E+08	G	T	ITGB5	UTR5	NA	NA	168	16	184	0.086957	82	0	82	0	0.29	Sub	0.6	2.91				7.8					Deleterious	NA
OVA_365_C1	Contralateral ovary	chr11	1.25E+08	1.25E+08	A	T	ESAM	exonic	ynonymous	125R,ENSG	191	98	289	0.3391	76	0	76	0	1	Clonal	0.42	1.83			polymorphism	7.406				benign	Neutral	Tolerated
OVA_013_M1	METASTASIS	chr8	1.25E+08	1.25E+08	C	G	FER1L6	exonic	ynonymous	921R,ENSG	523	87	610	0.142623	47	0	47	0	0.85	Clonal	0.67	3.06			disease_causing	25.5	p.P921R	Passenger		damaging	Deleterious	Damaging
OVA_013_C1	PRIMARY	chr8	1.25E+08	1.25E+08	C	G	FER1L6	exonic	ynonymous	921R,ENSG	530	99	629	0.157393	47	0	47	0	1	Clonal	0.72	3.1			disease_causing	25.5	p.P921R	Passenger		damaging	Deleterious	Damaging
OVA_013_C2	PRIMARY	chr8	1.25E+08	1.25E+08	C	G	FER1L6	exonic	ynonymous	921R,ENSG	492	111	603	0.18408	47	0	47	0	0.98	Clonal	0.87	3.05			disease_causing	25.5	p.P921R	Passenger		damaging	Deleterious	Damaging
OVA_013_C3	PRIMARY	chr8	1.25E+08	1.25E+08	C	G	FER1L6	exonic	ynonymous	921R,ENSG	523	103	626	0.164537	47	0	47	0	0.85	Clonal	0.91	3.01			disease_causing	25.5	p.P921R	Passenger		damaging	Deleterious	Damaging
OVA_013_M1	METASTASIS	chr11	1.25E+08	1.25E+08	A	T	PKNOX2	exonic	ynonymous	270L,ENSG	145	80	225	0.355556	57	0	57	0	1	Clonal	0.67	3.06			disease_causing	32	p.Q334L	Passenger	SENGER/OT	damaging	Deleterious	Damaging
OVA_013_C1	PRIMARY	chr11	1.25E+08	1.25E+08	A	T	PKNOX2	exonic	ynonymous	270L,ENSG	219	128	347	0.368876	57	0	57	0	1	Clonal	0.72	3.1			disease_causing	32	p.Q334L	Passenger	SENGER/OT	damaging	Deleterious	Damaging
OVA_013_C2	PRIMARY	chr11	1.25E+08	1.25E+08	A	T	PKNOX2	exonic	ynonymous	270L,ENSG	161	129	290	0.444828	57	0	57	0	1	Clonal	0.87	3.05			disease_causing	32	p.Q334L	Passenger	SENGER/OT	damaging	Deleterious	Damaging
OVA_013_C3	PRIMARY	chr11	1.25E+08	1.25E+08	A	T	PKNOX2	exonic	ynonymous	270L,ENSG	202	156	358	0.435754	57	0	57	0	1	Clonal	0.91	3.01			disease_causing	32	p.Q334L	Passenger	SENGER/OT	damaging	Deleterious	Damaging
OVA_047_M1	METASTASIS	chrX	1.25E+08	1.25E+08	G	T	DCAF12L2	exonic	ynonymous	.Q4K,ENSG	68	20	88	0.227273	19	0	19	0	0.96	Clonal	0.47	2.01	Yes	No	polymorphism	15.76	p.Q4K	Passenger	SENGER/OT	benign	Neutral	Damaging
OVA_047_M2	METASTASIS	chrX	1.25E+08	1.25E+08	G	T	DCAF12L2	exonic	ynonymous	.Q4K,ENSG	78	21	99	0.212121	19	0	19	0	0.96	Clonal	0.44	2.01	Yes	No	polymorphism	15.76	p.Q4K	Passenger	SENGER/OT	benign	Neutral	Damaging
OVA_047_M3	METASTASIS	chrX	1.25E+08	1.25E+08	G	T	DCAF12L2	exonic	ynonymous	.Q4K,ENSG	43	17	60	0.283333	19	0	19	0	1	Clonal	0.6	3.02	Yes	No	polymorphism	15.76	p.Q4K	Passenger	SENGER/OT	benign	Neutral	Damaging
OVA_013_C2	PRIMARY	chr6	1.26E+08	1.26E+08	C	G	HDDC2	exonic	ynonymous	101Q,ENSG	493	81	574	0.141115	47	0	47	0	0.32	Sub	0.87	3.05			disease_causing	29.1	p.E135Q	Passenger	SENGER/OT	damaging	Deleterious	Damaging
OVA_047_C1	PRIMARY	chr5	1.26E+08	1.26E+08	C	T	GRAMD3	exonic	ynonymous	000005423	571	54	625	0.0864	39	0	39	0	0.29	Sub	0.6	2.91			polymorphism	14.28	p.A62V	Passenger	SENGER/OT	damaging	Neutral	Damaging
OVA_365_M1	METASTASIS	chr3	1.26E+08	1.26E+08	C	T	ALDH1L1	exonic	onymous S	L519L,ENSG	89	110	199	0.552764	32	0	32	0	1	Clonal	0.85	1.87			disease_causing	8.106		Passenger			Neutral	Tolerated
OVA_365_M2	METASTASIS	chr3	1.26E+08	1.26E+08	C	T	ALDH1L1	exonic	onymous S	L519L,ENSG	128	30	158	0.189873	32	0	32	0	0.94	Clonal	0.4	1.81			disease_causing	8.106		Passenger			Neutral	Tolerated
OVA_365_M3	METASTASIS	chr3	1.26E+08	1.26E+08	C	T	ALDH1L1	exonic	onymous S	L519L,ENSG	112	75	187	0.40107	32	0	32	0	0.98	Clonal	0.82	1.89			disease_causing	8.106		Passenger			Neutral	Tolerated
OVA_365_C1	Contralateral ovary	chr3	1.26E+08	1.26E+08	C	T	ALDH1L1	exonic	onymous S	L519L,ENSG	152	62	214	0.28972	32	0	32	0	1	Clonal	0.42	1.83			disease_causing	8.106		Passenger			Neutral	Tolerated
OVA_365_C2	PRIMARY	chr3	1.26E+08	1.26E+08	C	T	ALDH1L1	exonic	onymous S	L519L,ENSG	153	74	227	0.325991	32	0	32	0	1	Clonal	0.62	1.92			disease_causing	8.106		Passenger			Neutral	Tolerated
OVA_003_M1	METASTASIS	chrX	1.26E+08	1.26E+08	G	A	CXorf64	exonic	stopgain	000037112	254	109	363	0.300275	73	1	74	0.013514	1	Sub	0.57	1.84			disease_causing	32	p.W72X	Passenger			NA	NA
OVA_003_M2	METASTASIS	chrX	1.26E+08	1.26E+08	G	A	CXorf64	exonic	stopgain	000037112	352	111	463	0.239741	73	1	74	0.013514	1	Sub	0.46	1.81			disease_causing	32	p.W72X	Passenger			NA	NA
OVA_003_M3	METASTASIS	chrX	1.26E+08	1.26E+08	G	A	CXorf64	exonic	stopgain	000037112	525	32	557	0.057451	73	1	74	0.013514	0.55	Sub	0.34	2.67			disease_causing	32	p.W72X	Passenger			NA	NA
OVA_003_C1	PRIMARY	chrX	1.26E+08	1.26E+08	G	A	CXorf64	exonic	stopgain	000037112	278	266	544	0.488971	73	1	74	0.013514	1	Clonal	0.86	1.81			disease_causing	32	p.W72X	Passenger			NA	NA
OVA_003_C2	PRIMARY	chrX	1.26E+08	1.26E+08	G	A	CXorf64	exonic	stopgain	000037112	275	258	533	0.484053	73	1	74	0.013514	1	Clonal	0.7	3.61			disease_causing	32	p.W72X	Passenger			NA	NA
OVA_003_C3	PRIMARY	chrX	1.26E+08	1.26E+08	G	A	CXorf64	exonic	stopgain	000037112	218	330	548	0.60219	73	1	74	0.013514	1	Clonal	0.82	1.81			disease_causing	32	p.W72X	Passenger			NA	NA
OVA_365_M1	METASTASIS	chr8	1.26E+08	1.26E+08	C	T	KIAA0196	intronic	NA	NA	478	140	618	0.226537	55	0	55	0	0.99	Clonal	0.85	1.87			disease_causing	11.25						
OVA_365_M2	METASTASIS	chr8	1.26E+08	1.26E+08	C	T	KIAA0196	intronic	NA	NA	286	41	327	0.125382	55	0	55	0	0.87	Clonal	0.4	1.81			disease_causing	11.25						
OVA_365_M3	METASTASIS	chr8	1.26E+08	1.26E+08	C	T	KIAA0196	intronic	NA	NA	436	113	549	0.205829	55	0	55	0	0.91	Clonal	0.82	1.89			disease_causing	11.25						
OVA_365_C1	Contralateral ovary	chr8	1.26E+08	1.26E+08	C	T	KIAA0196	intronic	NA	NA	294	41	335	0.122388	55	0	55	0	0.71	Sub	0.42	1.83			disease_causing	11.25						
OVA_365_C2	PRIMARY	chr8	1.26E+08	1.26E+08	C	T	KIAA0196	intronic	NA	NA	406	118	524	0.225191	55	0	55	0	0.96	Sub	0.62	1.92			disease_causing	11.25						
OVA_047_M1	METASTASIS	chr10	1.26E+08	1.26E+08	G	T	LHPP	exonic	ynonymous	000003688	22	8	30	0.266667	30	0	30	0	1	Clonal	0.47	2.01			polymorphism	11.78	p.A32S	Passenger	SENGER/OT	benign	Neutral	Tolerated
OVA_047_M2	METASTASIS	chr10	1.26E+08	1.26E+08	G	T	LHPP	exonic	ynonymous	000003688	37	5	42	0.119048	30	0	30	0	0.66	Clonal	0.44	2.01			polymorphism	11.78	p.A32S	Passenger	SENGER/OT	benign	Neutral	Tolerated
OVA_048_C2	PRIMARY	chr3	1.26E+08	1.26E+08	G	T	CHST13	exonic	ynonymous	000031934	100	73	173	0.421965	15	0	15	0	1	Clonal	0.72	1.85			disease_causing	21.9	p.G129V	Passenger	SENGER/OT	damaging	Deleterious	Damaging
OVA_378_C1	PRIMARY	chr3	1.27E+08	1.27E+08	C	A	PLXNA1	exonic	ynonymous	H61Q,ENSG	580	82	662	0.123867	154	0	154	0	0.79	Sub	0.59	2.6	Yes	No	disease_causing	14.34	p.H84Q	Passenger	SENGER/OT	damaging	Deleterious	Tolerated
OVA_365_M1	METASTASIS	chr3	1.27E+08	1.27E+08	G	T	PLXNA1	exonic	stopgain	.321X,ENSG	129	99	228	0.434211	93	0	93	0	1	Clonal	0.85	1.87	Yes	No	isease_causing_automat	48	p.E1344X	Passenger	SENGER/OT		NA	NA
OVA_365_M2	METASTASIS	chr3	1.27E+08	1.27E+08	G	T	PLXNA1	exonic	stopgain	.321X,ENSG	128	46	174	0.264368	93	0	93	0	1	Clonal	0.4	1.81	Yes	No	isease_causing_automat	48	p.E1344X	Passenger	SENGER/OT		NA	NA
OVA_365_M3	METASTASIS	chr3	1.27E+08	1.27E+08	G	T	PLXNA1	exonic	stopgain	.321X,ENSG	140	97	237	0.409283	93	0	93	0	1	Clonal	0.82	1.89	Yes	No	isease_causing_automat	48	p.E1344X	Passenger	SENGER/OT		NA	NA
OVA_365_C1	Contralateral ovary	chr3	1.27E+08	1.27E+08	G	T	PLXNA1	exonic	stopgain	.321X,ENSG	131	61	192	0.317708	93	0	93	0	1	Clonal	0.42	1.83	Yes	No	isease_causing_automat	48	p.E1344X	Passenger	SENGER/OT		NA	NA
OVA_365_C2	PRIMARY	chr3	1.27E+08	1.27E+08	G	T	PLXNA1	exonic	stopgain	.321X,ENSG	158	69	227	0.303965	93	0	93	0	0.99	Clonal	0.62	1.92	Yes	No	isease_causing_automat	48	p.E1344X	Passenger	SENGER/OT		NA	NA
OVA_003_C1	PRIMARY	chr5	1.27E+08	1.27E+08	C	G	PRRC1	exonic	onymous S	000004421	524	84	608	0.138158	53	0	53	0	0.32	Sub	0.86	1.81			polymorphism	0.42		Passenger			Neutral	Tolerated
OVA_003_C2	PRIMARY	chr5	1.27E+08	1.27E+08	C	G	PRRC1	exonic	onymous S	000004421	563	57	620	0.091935	53	0	53	0	0.45	Sub	0.7	3.61			polymorphism	0.42		Passenger			Neutral	Tolerated
OVA_003_C3	PRIMARY	chr5	1.27E+08	1.27E+08	C	G	PRRC1	exonic	onymous S	000004421	602	36	638	0.056426	53	0	53	0	0.14	Sub	0.82	1.81			polymorphism	0.42		Passenger			Neutral	Tolerated
OVA_003_M1	METASTASIS	chr3	1.27E+08	1.27E+08	A	T	BC015846	RNA_intro	NA	NA	271	82	353	0.232295	23	0	23	0	0.82	Sub	0.57	1.84				1.947						
OVA_003_M2	METASTASIS	chr3	1.27E+08	1.27E+08	A	T	BC015846	RNA_intro	NA	NA	327	74	401	0.184539	23	0	23	0	0.81	Sub	0.46	1.81				1.947						
OVA_003_M3	METASTASIS	chr3	1.27E+08	1.27E+08	A	T	BC015846	RNA_intro	NA	NA	348	18	366	0.04918	23	0	23	0	0.52	Sub	0.34	2.67				1.947						
OVA_003_C1	PRIMARY	chr3	1.27E+08	1.27E+08	A	T	BC015846	RNA_intro	NA	NA	243	202	445	0.453933	23	0	23	0	1	Clonal	0.86	1.81				1.947						
OVA_003_C2	PRIMARY	chr3	1.27E+08	1.27E+08	A	T	BC015846	RNA_intro	NA	NA	289	194	483	0.401656	23	0	23	0	1	Clonal	0.7	3.61				1.947						
OVA_003_C3	PRIMARY	chr3	1.27E+08	1.27E+08	A	T	BC015846	RNA_intro	NA	NA	298	219	517	0.423598	23	0	23	0	1	Clonal	0.82	1.81				1.947						
OVA_378_M1	METASTASIS	chr5	1.27E+08	1.27E+08	C	A	SLC12A2	exonic	onymous S	000003432	302	182	484	0.376033	28	0	28	0	1	Clonal	0.72	2.57			disease_causing	16.6		Passenger			Neutral	Tolerated
OVA_378_M2	METASTASIS	chr5	1.27E+08	1.27E+08	C	A	SLC12A2	exonic	onymous S	000003432	279	169	448	0.377232	28	0	28	0	0.88	Clonal	0.85	2.57			disease_causing	16.6		Passenger			Neutral	Tolerated
OVA_378_M3	METASTASIS	chr5	1.27E+08	1.27E+08	C	A	SLC12A2	exonic	onymous S	000003432	395	191	586	0.325939	28	0	28	0	0.98	Clonal	0.67	2.57			disease_causing	16.6		Passenger			Neutral	Tolerated
OVA_047_C1	PRIMARY	chr5	1.28E+08	1.28E+08	G	C	FBN2	exonic	ynonymous	877V,ENSG	434	170	604	0.281457	51	0	51	0	0.94	Sub	0.6	2.91			disease_causing	21.8	p.L2877V	Passenger	SENGER/OT	damaging	Neutral	Tolerated
OVA_047_C2	PRIMARY	chr5	1.28E+08	1.28E+08	G	C	FBN2	exonic	ynonymous	877V,ENSG	426	164	590	0.277966	51	0	51	0	0.87	Sub	0.64	2.76			disease_causing	21.8	p.L2877V	Passenger	SENGER/OT	damaging	Neutral	Tolerated
OVA_047_C3	Contralateral ovary	chr5	1.28E+08	1.28E+08	G	C	FBN2	exonic	ynonymous	877V,ENSG	420	152	572	0.265734	51	0	51	0	0.89	Clonal	0.6	2.94			disease_causing	21.8	p.L2877V	Passenger	SENGER/OT	damaging	Neutral	Tolerated
OVA_003_M1	METASTASIS	chr2	1.29E+08	1.29E+08	T	A	AMMECR1L	exonic	onymous S	.G2G,ENSG	369	152	521	0.291747	75	0	75	0	1	Sub	0.57	1.84			disease_causing	12.67		Passenger			Neutral	Tolerated
OVA_003_M2	METASTASIS	chr2	1.29E+08	1.29E+08	T	A	AMMECR1L	exonic	onymous S	.G2G,ENSG	432	113	545	0.207339	75	0	75	0	0.9	Sub	0.46	1.81			disease_causing	12.67		Passenger			Neutral	Tolerated
OVA_003_M3	METASTASIS	chr2	1.29E+08	1.29E+08	T	A	AMMECR1L	exonic	onymous S	.G2G,ENSG	595	30	625	0.048	75	0	75	0	0.51	Sub	0.34	2.67			disease_causing	12.67		Passenger			Neutral	Tolerated
OVA_003_C1	PRIMARY	chr2	1.29E+08	1.29E+08	T	A	AMMECR1L	exonic	onymous S	.G2G,ENSG	337	243	580	0.418966	75	0	75	0	0.97	Clonal	0.86	1.81			disease_causing	12.67		Passenger			Neutral	Tolerated
OVA_003_C2	PRIMARY	chr2	1.29E+08	1.29E+08	T	A	AMMECR1L	exonic	onymous S	.G2G,ENSG	336	250	586	0.426621	75	0	75	0	1	Clonal	0.7	3.61			disease_causing	12.67		Passenger			Neutral	Tolerated
OVA_003_C3	PRIMARY	chr2	1.29E+08	1.29E+08	T	A	AMMECR1L	exonic	onymous S	.G2G,ENSG	360	269	629	0.427663	75	0	75	0	1	Clonal	0.82	1.81			disease_causing	12.67		Passenger			Neutral	Tolerated
OVA_013_M1	METASTASIS	chr3	1.29E+08	1.29E+08	G	T	EFCAB12	exonic	ynonymous	P24H,ENSG	294	280	574	0.487805	70	0	70	0	1	Clonal	0.67	3.06			polymorphism	4.084	p.P24H	Passenger	SENGER/OT	benign	Neutral	Tolerated
OVA_013_C1	PRIMARY	chr3	1.29E+08	1.29E+08	G	T	EFCAB12	exonic	ynonymous	P24H,ENSG	251	323	574	0.562718	70	0	70	0	1	Clonal	0.72	3.1			polymorphism	4.084	p.P24H	Passenger	SENGER/OT	benign	Neutral	Tolerated
OVA_013_C2	PRIMARY	chr3	1.29E+08	1.29E+08	G	T	EFCAB12	exonic	ynonymous	P24H,ENSG	253	318	571	0.556918	70	0	70	0	1	Clonal	0.87	3.05			polymorphism	4.084	p.P24H	Passenger	SENGER/OT	benign	Neutral	Tolerated
OVA_013_C3	PRIMARY	chr3	1.29E+08	1.29E+08	G	T	EFCAB12	exonic	ynonymous	P24H,ENSG	246	345	591	0.583756	70	0	70	0	1	Clonal	0.91	3.01			polymorphism	4.084	p.P24H	Passenger	SENGER/OT	benign	Neutral	Tolerated
OVA_003_M1	METASTASIS	chrX	1.29E+08	1.29E+08	G	T	BCORL1	exonic	ynonymous	000218147	146	49	195	0.251282	78	0	78	0	0.89	Sub	0.57	1.84	Yes	Yes	disease_causing	23.5	p.C1148F	Passenger	SENGER/OT	damaging	Neutral	NA
OVA_003_M2	METASTASIS	chrX	1.29E+08	1.29E+08	G	T	BCORL1	exonic	ynonymous	000218147	152	53	205	0.258537	78	0	78	0	1	Sub	0.46	1.81	Yes	Yes	disease_causing	23.5	p.C1148F	Passenger	SENGER/OT	damaging	Neutral	NA
OVA_003_M3	METASTASIS	chrX	1.29E+08	1.29E+08	G	T	BCORL1	exonic	ynonymous	000218147	233	14	247	0.05668	78	0	78	0	0.54	Sub	0.34	2.67	Yes	Yes	disease_causing	23.5	p.C1148F	Passenger	SENGER/OT	damaging	Neutral	NA

Sample	Type	Chr	Start	End	Ref	Alt	Gene	unc.ensGer	icFunc.ens	hange.ens(	t_ref	t_alt	t_total	t_VAF	n_ref	n_alt	n_total	normal_VA	ncer_cell_f	on-based C	purity	ploidy	Cancer Gene	Driver Gene	Mutation Taster	ADD(PHRE	otein_chan	CHASM	THMM_Pr	OLYPHEN-	REDICTION	ICTION (cutoff=0.05)
OVA_003_C1	PRIMARY	chrX	1.29E+08	1.29E+08	G	T	BCORL1	exonic	ynonymou	000218147	157	127	284	0.447183	78	0	78	0	1	Clonal	0.86	1.81	Yes	Yes	disease_causing	23.5	p.C1148F	Passenger	SENGER/OT	damaging	Neutral	NA
OVA_003_C2	PRIMARY	chrX	1.29E+08	1.29E+08	G	T	BCORL1	exonic	ynonymou	000218147	133	154	287	0.536585	78	0	78	0	1	Clonal	0.7	3.61	Yes	Yes	disease_causing	23.5	p.C1148F	Passenger	SENGER/OT	damaging	Neutral	NA
OVA_003_C3	PRIMARY	chrX	1.29E+08	1.29E+08	G	T	BCORL1	exonic	ynonymou	000218147	81	150	231	0.649351	78	0	78	0	1	Clonal	0.82	1.81	Yes	Yes	disease_causing	23.5	p.C1148F	Passenger	SENGER/OT	damaging	Neutral	NA
OVA_048_M1	METASTASIS	chr12	1.29E+08	1.29E+08	G	T	TMEM132(	exonic	ynonymou	000315208	242	7	249	0.028112	20	0	20	0	0.1	Sub	0.56	3.94			disease_causing	14.61	p.Q749H	Passenger	SENGER/OT	benign	Deleterious	Tolerated
OVA_048_M2	METASTASIS	chr12	1.29E+08	1.29E+08	G	T	TMEM132(	exonic	ynonymou	000315208	267	18	285	0.063158	20	0	20	0	0.38	Sub	0.4	3.87			disease_causing	14.61	p.Q749H	Passenger	SENGER/OT	benign	Deleterious	Tolerated
OVA_048_M3	METASTASIS	chr12	1.29E+08	1.29E+08	G	T	TMEM132(	exonic	ynonymou	000315208	164	76	240	0.316667	20	0	20	0	1	Sub	0.59	3.39			disease_causing	14.61	p.Q749H	Passenger	SENGER/OT	benign	Deleterious	Tolerated
OVA_013_M1	METASTASIS	chrX	1.3E+08	1.3E+08	C	A	GPR119	exonic	ynonymou	000027621	281	72	353	0.203966	42	0	42	0	0.81	Sub	0.67	3.06			polymorphism	13.16	p.G211V	Passenger	SENGER/OT	benign	Neutral	Tolerated
OVA_013_C1	PRIMARY	chrX	1.3E+08	1.3E+08	C	A	GPR119	exonic	ynonymou	000027621	367	133	500	0.266	42	0	42	0	1	Sub	0.72	3.1			polymorphism	13.16	p.G211V	Passenger	SENGER/OT	benign	Neutral	Tolerated
OVA_013_C2	PRIMARY	chrX	1.3E+08	1.3E+08	C	A	GPR119	exonic	ynonymou	000027621	392	110	502	0.219124	42	0	42	0	0.72	Sub	0.87	3.05			polymorphism	13.16	p.G211V	Passenger	SENGER/OT	benign	Neutral	Tolerated
OVA_013_C3	PRIMARY	chrX	1.3E+08	1.3E+08	C	A	GPR119	exonic	ynonymou	000027621	387	153	540	0.283333	42	0	42	0	0.9	Sub	0.91	3.01			polymorphism	13.16	p.G211V	Passenger	SENGER/OT	benign	Neutral	Tolerated
OVA_013_M1	METASTASIS	chr7	1.3E+08	1.3E+08	G	T	CPA1	exonic	ynonymou	239Y,ENSG	470	116	586	0.197952	19	0	19	0	0.99	Clonal	0.67	3.06			polymorphism	9.154	p.D327Y	Passenger	SENGER/OT	benign	Deleterious	Damaging
OVA_013_C1	PRIMARY	chr7	1.3E+08	1.3E+08	G	T	CPA1	exonic	ynonymou	239Y,ENSG	496	113	609	0.18555	19	0	19	0	0.89	Clonal	0.72	3.1			polymorphism	9.154	p.D327Y	Passenger	SENGER/OT	benign	Deleterious	Damaging
OVA_013_C2	PRIMARY	chr7	1.3E+08	1.3E+08	G	T	CPA1	exonic	ynonymou	239Y,ENSG	478	121	599	0.202003	19	0	19	0	0.87	Clonal	0.87	3.05			polymorphism	9.154	p.D327Y	Passenger	SENGER/OT	benign	Deleterious	Damaging
OVA_013_C3	PRIMARY	chr7	1.3E+08	1.3E+08	G	T	CPA1	exonic	ynonymou	239Y,ENSG	494	140	634	0.22082	19	0	19	0	0.92	Clonal	0.91	3.01			polymorphism	9.154	p.D327Y	Passenger	SENGER/OT	benign	Deleterious	Damaging
OVA_365_M1	METASTASIS	chr6	1.3E+08	1.3E+08	C	A	L3MBTL3	exonic	ynonymou	26D,ENSG0	459	90	549	0.163934	43	0	43	0	0.39	Sub	0.85	1.87			disease_causing	27.6	p.A351D	Passenger	CANCER	damaging	Deleterious	Damaging
OVA_048_M1	METASTASIS	chr9	1.31E+08	1.31E+08	G	-	SLC25A25	exonic	heshift dele	K158fs,ENS	279	158	437	0.361556	55	0	55		1	Clonal	0.56	3.94					p.R159Efs	Passenger			NA	NA
OVA_048_M2	METASTASIS	chr9	1.31E+08	1.31E+08	G	-	SLC25A25	exonic	heshift dele	K158fs,ENS	304	114	418	0.272727	55	0	55		1	Clonal	0.4	3.87					p.R159Efs	Passenger			NA	NA
OVA_048_M3	METASTASIS	chr9	1.31E+08	1.31E+08	G	-	SLC25A25	exonic	heshift dele	K158fs,ENS	281	82	363	0.225895	55	0	55		1	Clonal	0.59	3.39					p.R159Efs	Passenger			NA	NA
OVA_048_C1	PRIMARY	chr9	1.31E+08	1.31E+08	G	-	SLC25A25	exonic	heshift dele	K158fs,ENS	234	123	357	0.344538	55	0	55		1	Clonal	0.67	1.82					p.R159Efs	Passenger			NA	NA
OVA_048_C2	PRIMARY	chr9	1.31E+08	1.31E+08	G	-	SLC25A25	exonic	heshift dele	K158fs,ENS	340	177	517	0.34236	55	0	55		0.95	Clonal	0.72	1.85					p.R159Efs	Passenger			NA	NA
OVA_048_C3	PRIMARY	chr9	1.31E+08	1.31E+08	G	-	SLC25A25	exonic	heshift dele	K158fs,ENS	195	121	316	0.382911	55	0	55		1	Clonal	0.74	1.8					p.R159Efs	Passenger			NA	NA
OVA_365_M1	METASTASIS	chr8	1.31E+08	1.31E+08	C	T	ASAP1	intronic	NA	NA	385	148	533	0.277674	25	0	25	0	1	Clonal	0.85	1.87			polymorphism	3.384						
OVA_365_M2	METASTASIS	chr8	1.31E+08	1.31E+08	C	T	ASAP1	intronic	NA	NA	213	48	261	0.183908	25	0	25	0	1	Clonal	0.4	1.81			polymorphism	3.384						
OVA_365_M3	METASTASIS	chr8	1.31E+08	1.31E+08	C	T	ASAP1	intronic	NA	NA	364	139	503	0.276342	25	0	25	0	1	Clonal	0.82	1.89			polymorphism	3.384						
OVA_365_C1	Contralateral ovary	chr8	1.31E+08	1.31E+08	C	T	ASAP1	intronic	NA	NA	214	55	269	0.204461	25	0	25	0	1	Clonal	0.42	1.83			polymorphism	3.384						
OVA_365_C2	PRIMARY	chr8	1.31E+08	1.31E+08	C	T	ASAP1	intronic	NA	NA	321	93	414	0.224638	25	0	25	0	0.95	Clonal	0.62	1.92			polymorphism	3.384						
OVA_003_M1	METASTASIS	chrX	1.32E+08	1.32E+08	C	T	AK095439	RNA_intro	NA	NA	460	104	564	0.184397	34	0	34	0	0.65	Sub	0.57	1.84			polymorphism	2.182						
OVA_003_M2	METASTASIS	chrX	1.32E+08	1.32E+08	C	T	AK095439	RNA_intro	NA	NA	479	74	553	0.133816	34	0	34	0	0.58	Sub	0.46	1.81			polymorphism	2.182						
OVA_003_M3	METASTASIS	chrX	1.32E+08	1.32E+08	C	T	AK095439	RNA_intro	NA	NA	575	24	599	0.040067	34	0	34	0	0.38	Sub	0.34	2.67			polymorphism	2.182						
OVA_003_C1	PRIMARY	chrX	1.32E+08	1.32E+08	C	T	AK095439	RNA_intro	NA	NA	358	208	566	0.367491	34	0	34	0	0.85	Sub	0.86	1.81			polymorphism	2.182						
OVA_003_C2	PRIMARY	chrX	1.32E+08	1.32E+08	C	T	AK095439	RNA_intro	NA	NA	401	166	567	0.292769	34	0	34	0	1	Sub	0.7	3.61			polymorphism	2.182						
OVA_003_C3	PRIMARY	chrX	1.32E+08	1.32E+08	C	T	AK095439	RNA_intro	NA	NA	462	114	576	0.197917	34	0	34	0	0.48	Sub	0.82	1.81			polymorphism	2.182						
OVA_048_C1	PRIMARY	chr9	1.32E+08	1.32E+08	G	A	ZER1	exonic	nonymous S	L157L,ENSG	48	25	73	0.342466	28	0	28	0	1	Clonal	0.67	1.82			disease_causing	15.59		Passenger			Neutral	Tolerated
OVA_047_C1	PRIMARY	chr5	1.33E+08	1.33E+08	T	G	FSTL4	exonic	ynonymou	0000265342	436	171	607	0.281713	54	0	54	0	0.94	Clonal	0.6	2.91			disease_causing	27.4	p.Y549S	Passenger	SENGER/OT	damaging	Deleterious	Damaging
OVA_047_C2	PRIMARY	chr5	1.33E+08	1.33E+08	T	G	FSTL4	exonic	ynonymou	0000265342	413	183	596	0.307047	54	0	54	0	0.96	Clonal	0.64	2.76			disease_causing	27.4	p.Y549S	Passenger	SENGER/OT	damaging	Deleterious	Damaging
OVA_047_C3	Contralateral ovary	chr5	1.33E+08	1.33E+08	T	G	FSTL4	exonic	ynonymou	0000265342	412	188	600	0.313333	54	0	54	0	1	Clonal	0.6	2.94			disease_causing	27.4	p.Y549S	Passenger	SENGER/OT	damaging	Deleterious	Damaging
OVA_365_C1	Contralateral ovary	chr10	1.33E+08	1.33E+08	C	T	TCERG1L	exonic	ynonymou	0000036864	52	12	64	0.1875	59	0	59	0	0.9	Clonal	0.42	1.83			disease_causing	12.57	p.A299T	Passenger	SENGER/OT	benign	Neutral	Tolerated
OVA_378_M1	METASTASIS	chr9	1.33E+08	1.33E+08	C	A	;DKFZp434	intronic	NA	NA	286	104	390	0.266667	42	0	42	0	0.74	Sub	0.72	2.57				0.723						
OVA_378_M2	METASTASIS	chr9	1.33E+08	1.33E+08	C	A	;DKFZp434	intronic	NA	NA	230	158	388	0.407216	42	0	42	0	0.95	Sub	0.85	2.57				0.723						
OVA_378_M3	METASTASIS	chr9	1.33E+08	1.33E+08	C	A	;DKFZp434	intronic	NA	NA	211	117	328	0.356707	42	0	42	0	1	Sub	0.67	2.57				0.723						
OVA_378_C1	PRIMARY	chr9	1.33E+08	1.33E+08	C	A	;DKFZp434	intronic	NA	NA	295	134	429	0.312354	42	0	42	0	1	Clonal	0.59	2.6				0.723						
OVA_378_C2	PRIMARY	chr9	1.33E+08	1.33E+08	C	A	;DKFZp434	intronic	NA	NA	304	119	423	0.281324	42	0	42	0	1	Clonal	0.56	2.59				0.723						
OVA_378_C3	PRIMARY	chr9	1.33E+08	1.33E+08	C	A	;DKFZp434	intronic	NA	NA	334	96	430	0.223256	42	0	42	0	0.88	Clonal	0.51	2.6				0.723						
OVA_047_M1	METASTASIS	chr3	1.33E+08	1.33E+08	G	A	BFSP2	UTR5	NA	NA	303	114	417	0.273381	57	0	57	0	1	Clonal	0.47	2.01			polymorphism	11.68						
OVA_047_M2	METASTASIS	chr3	1.33E+08	1.33E+08	G	A	BFSP2	UTR5	NA	NA	376	160	536	0.298507	57	0	57	0	1	Clonal	0.44	2.01			polymorphism	11.68						
OVA_047_M3	METASTASIS	chr3	1.33E+08	1.33E+08	G	A	BFSP2	UTR5	NA	NA	122	40	162	0.246914	57	0	57	0	1	Clonal	0.6	3.02			polymorphism	11.68						
OVA_365_M1	METASTASIS	chr12	1.33E+08	1.33E+08	C	T	FBRSL1	exonic	stopgain	Q370X,ENS	8	56	64	0.875	28	0	28	0	1	Sub	0.85	1.87			disease_causing_automat	47	p.Q370X	Passenger			NA	NA
OVA_365_M2	METASTASIS	chr12	1.33E+08	1.33E+08	C	T	FBRSL1	exonic	stopgain	Q370X,ENS	9	22	31	0.709677	28	0	28	0	1	Sub	0.4	1.81			disease_causing_automat	47	p.Q370X	Passenger			NA	NA
OVA_365_M3	METASTASIS	chr12	1.33E+08	1.33E+08	C	T	FBRSL1	exonic	stopgain	Q370X,ENS	5	28	33	0.848485	28	0	28	0	1	Sub	0.82	1.89			disease_causing_automat	47	p.Q370X	Passenger			NA	NA
OVA_365_C1	Contralateral ovary	chr12	1.33E+08	1.33E+08	C	T	FBRSL1	exonic	stopgain	Q370X,ENS	12	27	39	0.692308	28	0	28	0	1	Sub	0.42	1.83			disease_causing_automat	47	p.Q370X	Passenger			NA	NA
OVA_365_C2	PRIMARY	chr12	1.33E+08	1.33E+08	C	T	FBRSL1	exonic	stopgain	Q370X,ENS	12	45	57	0.789474	28	0	28	0	1	Sub	0.62	1.92			disease_causing_automat	47	p.Q370X	Passenger			NA	NA
OVA_047_M1	METASTASIS	chr9	1.33E+08	1.33E+08	G	A	ASS1	intronic	NA	NA	191	44	235	0.187234	57	1	58	0.017241	0.79	Clonal	0.47	2.01			polymorphism	2.858						
OVA_047_M2	METASTASIS	chr9	1.33E+08	1.33E+08	G	A	ASS1	intronic	NA	NA	230	64	294	0.217687	57	1	58	0.017241	0.98	Clonal	0.44	2.01			polymorphism	2.858						
OVA_047_M3	METASTASIS	chr9	1.33E+08	1.33E+08	G	A	ASS1	intronic	NA	NA	90	20	110	0.181818	57	1	58	0.017241	0.79	Clonal	0.6	3.02			polymorphism	2.858						
OVA_013_M1	METASTASIS	chr4	1.33E+08	1.33E+08	A	T	31768;BC04	intronic	NA	NA	121	56	177	0.316384	13	0	13	0	0.94	Clonal	0.67	3.06				1.547						
OVA_013_C1	PRIMARY	chr4	1.33E+08	1.33E+08	A	T	31768;BC04	intronic	NA	NA	95	87	182	0.478022	13	0	13	0	1	Clonal	0.72	3.1				1.547						
OVA_013_C2	PRIMARY	chr4	1.33E+08	1.33E+08	A	T	31768;BC04	intronic	NA	NA	78	54	132	0.409091	13	0	13	0	0.94	Clonal	0.87	3.05				1.547						
OVA_013_C3	PRIMARY	chr4	1.33E+08	1.33E+08	A	T	31768;BC04	intronic	NA	NA	82	78	160	0.4875	13	0	13	0	1	Clonal	0.91	3.01				1.547						
OVA_047_M1	METASTASIS	chr9	1.33E+08	1.33E+08	G	T	FUBP3	intronic	NA	NA	40	9	49	0.183673	28	0	28	0	0.77	Clonal	0.47	2.01			disease_causing	15.9						
OVA_048_M1	METASTASIS	chr5	1.34E+08	1.34E+08	C	A	PPP2CA	exonic	ynonymou	0000048119	181	372	553	0.672694	46	0	46	0	1	Clonal	0.56	3.94			disease_causing	28	p.A216S	Passenger	SENGER/OT	damaging	Deleterious	Damaging
OVA_048_M2	METASTASIS	chr5	1.34E+08	1.34E+08	C	A	PPP2CA	exonic	ynonymou	0000048119	282	264	546	0.483516	46	0	46	0	1	Clonal	0.4	3.87			disease_causing	28	p.A216S	Passenger	SENGER/OT	damaging	Deleterious	Damaging
OVA_048_M3	METASTASIS	chr5	1.34E+08	1.34E+08	C	A	PPP2CA	exonic	ynonymou	0000048119	213	337	550	0.612727	46	0	46	0	1	Clonal	0.59	3.39			disease_causing	28	p.A216S	Passenger	SENGER/OT	damaging	Deleterious	Damaging
OVA_365_M1	METASTASIS	chr8	1.34E+08	1.34E+08	C	T	LRRC6	UTR3	NA	NA	454	140	594	0.23569	30	0	30	0	1	Clonal	0.85	1.87			polymorphism	5.987						
OVA_013_M1	METASTASIS	chr9	1.34E+08	1.34E+08	C	T	FIBCD1	intronic	NA	NA	16	52	68	0.764706	14	0	14	0	1	Clonal	0.67	3.06			polymorphism	4.401						
OVA_013_C1	PRIMARY	chr9	1.34E+08	1.34E+08	C	T	FIBCD1	intronic	NA	NA	13	64	77	0.831169	14	0	14	0	1	Clonal	0.72	3.1			polymorphism	4.401						
OVA_013_C2	PRIMARY	chr9	1.34E+08	1.34E+08	C	T	FIBCD1	intronic	NA	NA	11	60	71	0.84507	14	0	14	0	1	Clonal	0.87	3.05			polymorphism	4.401						
OVA_013_C3	PRIMARY	chr9	1.34E+08	1.34E+08	C	T	FIBCD1	intronic	NA	NA	7	55	62	0.887097	14	0	14	0	1	Clonal	0.91	3.01			polymorphism	4.401						
OVA_003_M1	METASTASIS	chr8	1.34E+08	1.34E+08	G	A	PHF20L1	UTR3	NA	NA	572	34	606	0.056106	77	1	78	0.012821	0.31	Sub	0.57	1.84			disease_causing	17.92						
OVA_003_M2	METASTASIS	chr8	1.34E+08	1.34E+08	G	A	PHF20L1	UTR3	NA	NA	563	26	589	0.044143	77	1	78	0.012821	0.24	Sub	0.46	1.81			disease_causing	17.92						
OVA_003_C1	PRIMARY	chr8	1.34E+08	1.34E+08	G	A	PHF20L1	UTR3	NA	NA	512	132	644	0.204969	77	1	78	0.012821	0.88	Sub	0.86	1.81			disease_causing	17.92						
OVA_003_C2	PRIMARY	chr8	1.34E+08	1.34E+08	G	A	PHF20L1	UTR3	NA	NA	588	68	656	0.103659	77	1	78	0.012821	0.81	Sub	0.7	3.61			disease_causing	17.92						
OVA_003_C3	PRIMARY	chr8	1.34E+08	1.34E+08	G	A	PHF20L1	UTR3	NA	NA	505	147	652	0.22546	77	1	78	0.012821	0.77	Sub	0.82	1.81			disease_causing	17.92						
OVA_048_C2	PRIMARY	chr9	1.34E+08	1.34E+08	G	T	LAMC3	splicing	NA	NA	187	76	263	0.288973	43	0	43	0	0.8	Sub	0.72	1.85			disease_causing	27.8		Passenger				
OVA_013_M1	METASTASIS	chr8	1.34E+08	1.34E+08	C	G	SLA	exonic	nonymous S	0000052211	364	187	551	0.339383	181	0	181	0	1	Clonal	0.67	3.06			disease_causing	14.8		Passenger			Neutral	Tolerated
OVA_013_C1	PRIMARY	chr8	1.34E+08	1.34E+08	C	G	SLA	exonic	nonymous S	0000052211	340	214	554	0.386282	181	0	181	0	1	Clonal	0.72	3.1			disease_causing	14.8		Passenger			Neutral	Tolerated
OVA_013_C2	PRIMARY	chr8	1.34E+08	1.34E+08	C	G	SLA	exonic	nonymous S	0000052211	315	209	524	0.398855	181	0	181	0	0.92	Clonal	0.87	3.05			disease_causing	14.8		Passenger			Neutral	Tolerated
OVA_013_C3	PRIMARY	chr8	1.34E+08	1.34E+08	C	G	SLA	exonic	nonymous S	0000052211	334	245	579	0.423143	181	0	181	0	0.93	Clonal	0.91	3.01			disease_causing	14.8		Passenger			Neutral	Tolerated
OVA_013_M1	METASTASIS	chr11	1.34E+08	1.34E+08	G	T	NCAPD3	intronic	NA	NA	338	169	507	0.333333	35	0	35	0	0.99	Clonal	0.67	3.06			polymorphism	12.07						
OVA_013_C1	PRIMARY	chr11	1.34E+08	1.34E+08	G	T	NCAPD3	intronic	NA	NA	306	196	502	0.390438	35	0	35	0	1	Sub	0.72	3.1			polymorphism	12.07						
OVA_013_C2	PRIMARY	chr11	1.34E+08	1.34E+08	G	T	NCAPD3	intronic	NA	NA	234	156	390	0.4	35	0	35	0	0.92	Sub	0.87	3.05			polymorphism	12.07						
OVA_013_C3	PRIMARY	chr11	1.34E+08	1.34E+08	G	T	NCAPD3	intronic	NA	NA	328	208	536	0.38806	35	0	35	0	0.85	Sub	0.91	3.01			polymorphism	12.07						
OVA_013_M1	METASTASIS	chr8	1.34E+08	1.34E+08	C	A	TG	exonic	ynonymou	0542445:ex	93	154	247	0.623482	26	0	26	0	1	Clonal	0.67	3.06			polymorphism	13.27	p.P2761Q	Passenger	SENGER/OT	damaging	Deleterious	Damaging
OVA_013_C1	PRIMARY	chr8	1.34E+08	1.34E+08	C	A	TG	exonic	ynonymou	0542445:ex	83	263	346	0.760116	26	0	26	0	1	Clonal	0.72	3.1			polymorphism	13.27	p.P2761Q	Passenger	SENGER/OT	damaging	Deleterious	Damaging
OVA_013_C2	PRIMARY	chr8	1.34E+08	1.34E+08	C	A	TG	exonic	ynonymou	0542445:ex	47	242	289	0.83737	26	0	26	0	1	Clonal	0.87	3.05			polymorphism	13.27	p.P2761Q	Passenger	SENGER/OT	damaging	Deleterious	Damaging
OVA_013_C3	PRIMARY	chr8	1.34E+08	1.34E+08	C	A	TG	exonic	ynonymou	0542445:ex	38	268	306	0.875817	26	0	26	0	1	Clonal	0.91	3.01			polymorphism	13.27	p.P2761Q	Passenger	SENGER/OT	damaging	Deleterious	Damaging
OVA_048_M1	METASTASIS	chr3	1.34E+08	1.34E+08	C	T	CEP63	exonic	ynonymou	R229C,ENSG	309	220	529	0.415879	44	0	44	0	1	Clonal	0.56	3.94			disease_causing	29.9	p.R229C	Passenger	SENGER/OT	damaging	Deleterious	Damaging
OVA_048_M2	METASTASIS	chr3	1.34E+08	1.34E+08	C	T	CEP63	exonic	ynonymou	R229C,ENSG	388	147	535	0.274766	44	0	44	0	1	Clonal	0.4	3.87			disease_causing	29.9	p.R229C	Passenger	SENGER/OT	damaging	Deleterious	Damaging
OVA_048_M3	METASTASIS	chr3	1.34E+08	1.34E+08	C	T	CEP63	exonic	ynonymou	R229C,ENSG	329	194	523	0.370937	44	0	44	0	1	Clonal	0.59	3.39			disease_causing	29.9	p.R229C	Passenger	SENGER/OT	damaging	Deleterious	Damaging
OVA_047_M1	METASTASIS	chr3	1.34E+08	1.34E+08	C	T	KY	intronic	NA	NA	488	146	634	0.230284	52	1	53	0.018868	0.97	Sub	0.47	2.01			polymorphism	2.752						
OVA_047_M2	METASTASIS	chr3	1.34E+08	1.34E+08	C	T	KY	intronic	NA	NA	537	119	656	0.181402	52	1	53	0.018868	0.82	Sub	0.44	2.01			polymorphism	2.752						
OVA_047_M3	METASTASIS	chr3	1.34E+08	1.34E+08	C	T	KY	intronic	NA	NA	297	86	383	0.224543	52	1	53	0.018868	0.97	Sub	0.6	3.02			polymorphism	2.752						
OVA_048_M3	METASTASIS	chr9	1.34E+08	1.34E+08	C	A	POMT1	RNA_intro	NA	NA	336	96	432	0.222222	49	0	49	0	1	Clonal	0.59	3.39			polymorphism	1.175						
OVA_047_C1	PRIMARY	chr6	1.34E+08	1.34E+08	A	T	SGK1	exonic	ynonymou	/136E,ENSG	470	172	642	0.267913	81	0	81	0	0.89	Sub	0.6	2.91	Yes	No	polymorphism	14.02	p.V217E	Passenger	SENGER/OT	benign	Neutral	Tolerated
OVA_047_C2	PRIMARY	chr6	1.34E+08	1.34E+08	A	T	SGK1	exonic	ynonymou	/136E,ENSG	453	173	626	0.276358	81	0	81	0	0.86	Sub	0.64	2.76	Yes	No	polymorphism	14.02	p.V217E	Passenger	SENGER/OT	benign	Neutral	Tolerated
OVA_047_C3	Contralateral ovary	chr6	1.34E+08	1.34E+08	A	T	SGK1	exonic	ynonymou	/136E,ENSG	460	167	627	0.266348	81	0	81	0	0.89	Clonal	0.6	2.94	Yes	No	polymorphism	14.02	p.V217E	Passenger	SENGER/OT	benign	Neutral	Tolerated
OVA_013_M1	METASTASIS	chr10	1.35E+08	1.35E+08	C	A	INPP5A	intronic	NA	NA	187	75	262	0.28626	45	0	45	0	0.85	Clonal	0.67	3.06			disease_causing	1.696						
OVA_013_C1	PRIMARY	chr10	1.35E+08	1.35E+08	C	A	INPP5A	intronic	NA	NA	223	133	356	0.373596	45	0	45	0	1	Clonal	0.72	3.1			disease_causing	1.696						
OVA_013_C2	PRIMARY	chr10	1.35E+08	1.35E+08	C	A	INPP5A	intronic	NA	NA	209	139	348	0.399425	45	0	45	0	1	Clonal	0.87	3.05			disease_causing	1.696						
OVA_013_C3	PRIMARY	chr10	1.35E+08	1.35E+08	C	A	INPP5A	intronic	NA	NA	267	156	423	0.368794	45	0	45	0	1	Clonal	0.91	3.01			disease_causing	1.696						
OVA_048_C1	PRIMARY	chr10	1.35E+08	1.35E+08	G	T	KNDC1	exonic	ynonymou	000030461	62	32	94	0.340426	62	0	62	0	1	Clonal	0.67	1.82			polymorphism	5.532	p.S687I	Passenger	SENGER/OT	benign	Neutral	Damaging

Sample	Type	Chr	Start	End	Ref	Alt	Gene	unc.ensGen	icFunc.ens	hange.ens	t_ref	t_alt	t_total	t_VAF	n_ref	n_alt	n_total	ormal_VA	ncer_cell_f	on-based C	purity	ploidy	Cancer Gene	Driver Gene	Mutation Taster	ADD(PHRE	otein_chan	CHASM	ATHMM_Pr	OLYPHEN-	REDICTION	ICTION (cutoff=0.05)
OVA_048_C3	PRIMARY	chr10	1.35E+08	1.35E+08	G	T	KNDC1	exonic	ynonymou	000030461	83	54	137	0.394161	62	0	62	0	1	Clonal	0.74	1.8			polymorphism	5.532	p.S687I	Passenger	SENGER/OT	benign	Neutral	Damaging
OVA_048_M1	METASTASIS	chr9	1.35E+08	1.35E+08	-	A	SETX	intronic	NA	NA	306	174	480	0.3625	23	0	23		1	Clonal	0.56	3.94			polymorphism							
OVA_048_M2	METASTASIS	chr9	1.35E+08	1.35E+08	-	A	SETX	intronic	NA	NA	381	117	498	0.23494	23	0	23		1	Clonal	0.4	3.87			polymorphism							
OVA_048_C1	PRIMARY	chr9	1.35E+08	1.35E+08	-	A	SETX	intronic	NA	NA	156	115	271	0.424354	23	0	23		0.84	Sub	0.67	1.82			polymorphism							
OVA_048_C2	PRIMARY	chr9	1.35E+08	1.35E+08	-	A	SETX	intronic	NA	NA	381	180	561	0.320856	23	0	23		0.89	Sub	0.72	1.85			polymorphism							
OVA_048_C3	PRIMARY	chr9	1.35E+08	1.35E+08	-	A	SETX	intronic	NA	NA	167	97	264	0.367424	23	0	23		0.99	Sub	0.74	1.8			polymorphism							
OVA_048_M1	METASTASIS	chrX	1.35E+08	1.35E+08	C	A	FHL1	intronic	NA	NA	383	161	544	0.295956	42	0	42	0	1	Sub	0.56	3.94			polymorphism	0.038						
OVA_048_M2	METASTASIS	chrX	1.35E+08	1.35E+08	C	A	FHL1	intronic	NA	NA	414	114	528	0.215909	42	0	42	0	1	Sub	0.4	3.87			polymorphism	0.038						
OVA_048_M3	METASTASIS	chrX	1.35E+08	1.35E+08	C	A	FHL1	intronic	NA	NA	500	15	515	0.029126	42	0	42	0	0.26	Sub	0.59	3.39			polymorphism	0.038						
OVA_048_C1	PRIMARY	chrX	1.35E+08	1.35E+08	C	A	FHL1	intronic	NA	NA	245	59	304	0.194079	42	0	42	0	0.58	Sub	0.67	1.82			polymorphism	0.038						
OVA_048_C2	PRIMARY	chrX	1.35E+08	1.35E+08	C	A	FHL1	intronic	NA	NA	379	142	521	0.272553	42	0	42	0	0.76	Sub	0.72	1.85			polymorphism	0.038						
OVA_048_C3	PRIMARY	chrX	1.35E+08	1.35E+08	C	A	FHL1	intronic	NA	NA	261	108	369	0.292683	42	0	42	0	0.79	Sub	0.74	1.8			polymorphism	0.038						
OVA_047_C3	Contralateral ovary	chr10	1.35E+08	1.35E+08	A	C	CYP2E1	intronic	NA	NA	197	75	272	0.275735	27	0	27	0	0.92	Clonal	0.6	2.94			polymorphism	2.973						
OVA_365_M1	METASTASIS	chrX	1.36E+08	1.36E+08	G	C	CD40LG	exonic	ynonymou	/154L,ENSG	301	197	498	0.395582	91	0	91	0	0.93	Clonal	0.85	1.87			disease_causing	26.3	p.V175L	Passenger	SENGER/OT	damaging	Neutral	Damaging
OVA_013_M1	METASTASIS	chr9	1.36E+08	1.36E+08	C	T	C9orf96	intronic	NA	NA	84	106	190	0.557895	23	0	23	0	1	Clonal	0.67	3.06			polymorphism	0.109						
OVA_013_C1	PRIMARY	chr9	1.36E+08	1.36E+08	C	T	C9orf96	intronic	NA	NA	98	144	242	0.595041	23	0	23	0	1	Clonal	0.72	3.1			polymorphism	0.109						
OVA_013_C2	PRIMARY	chr9	1.36E+08	1.36E+08	C	T	C9orf96	intronic	NA	NA	123	164	287	0.571429	23	0	23	0	1	Clonal	0.87	3.05			polymorphism	0.109						
OVA_013_C3	PRIMARY	chr9	1.36E+08	1.36E+08	C	T	C9orf96	intronic	NA	NA	151	188	339	0.554572	23	0	23	0	1	Clonal	0.91	3.01			polymorphism	0.109						
OVA_047_M1	METASTASIS	chr5	1.36E+08	1.36E+08	G	C	SPOCK1	exonic	stopgain	343X,ENSG	374	202	576	0.350694	83	0	83	0	1	Clonal	0.47	2.01			disease_causing_automat	37	p.Y343X	Passenger			NA	NA
OVA_047_M2	METASTASIS	chr5	1.36E+08	1.36E+08	G	C	SPOCK1	exonic	stopgain	343X,ENSG	427	174	601	0.289517	83	0	83	0	1	Clonal	0.44	2.01			disease_causing_automat	37	p.Y343X	Passenger			NA	NA
OVA_047_M3	METASTASIS	chr5	1.36E+08	1.36E+08	G	C	SPOCK1	exonic	stopgain	343X,ENSG	178	86	264	0.325758	83	0	83	0	1	Clonal	0.6	3.02			disease_causing_automat	37	p.Y343X	Passenger			NA	NA
OVA_013_M1	METASTASIS	chr2	1.37E+08	1.37E+08	G	T	LCT	exonic	ynonymou	377E,ENSG	346	102	448	0.227679	47	0	47	0	0.91	Clonal	0.67	3.06			polymorphism	13.33	p.D945E	Passenger	SENGER/OT	damaging	Neutral	Tolerated
OVA_013_C1	PRIMARY	chr2	1.37E+08	1.37E+08	G	T	LCT	exonic	ynonymou	377E,ENSG	431	131	562	0.233096	47	0	47	0	0.88	Sub	0.72	3.1			polymorphism	13.33	p.D945E	Passenger	SENGER/OT	damaging	Neutral	Tolerated
OVA_013_C2	PRIMARY	chr2	1.37E+08	1.37E+08	G	T	LCT	exonic	ynonymou	377E,ENSG	392	152	544	0.279412	47	0	47	0	0.92	Sub	0.87	3.05			polymorphism	13.33	p.D945E	Passenger	SENGER/OT	damaging	Neutral	Tolerated
OVA_013_C3	PRIMARY	chr2	1.37E+08	1.37E+08	G	T	LCT	exonic	ynonymou	377E,ENSG	425	143	568	0.251761	47	0	47	0	0.8	Sub	0.91	3.01			polymorphism	13.33	p.D945E	Passenger	SENGER/OT	damaging	Neutral	Tolerated
OVA_003_M1	METASTASIS	chr2	1.37E+08	1.37E+08	A	G	DARS	exonic	unknown	UNKNOWN	315	81	396	0.204545	154	0	154	0	0.72	Sub	0.57	1.84			polymorphism	16.15					Neutral	Tolerated
OVA_003_M2	METASTASIS	chr2	1.37E+08	1.37E+08	A	G	DARS	exonic	unknown	UNKNOWN	415	88	503	0.17495	154	0	154	0	0.76	Sub	0.46	1.81			polymorphism	16.15					Neutral	Tolerated
OVA_003_M3	METASTASIS	chr2	1.37E+08	1.37E+08	A	G	DARS	exonic	unknown	UNKNOWN	414	23	437	0.052632	154	0	154	0	0.55	Sub	0.34	2.67			polymorphism	16.15					Neutral	Tolerated
OVA_003_C1	PRIMARY	chr2	1.37E+08	1.37E+08	A	G	DARS	exonic	unknown	UNKNOWN	216	138	354	0.389831	154	0	154	0	0.9	Clonal	0.86	1.81			polymorphism	16.15					Neutral	Tolerated
OVA_003_C2	PRIMARY	chr2	1.37E+08	1.37E+08	A	G	DARS	exonic	unknown	UNKNOWN	228	126	354	0.355932	154	0	154	0	1	Clonal	0.7	3.61			polymorphism	16.15					Neutral	Tolerated
OVA_003_C3	PRIMARY	chr2	1.37E+08	1.37E+08	A	G	DARS	exonic	unknown	UNKNOWN	262	152	414	0.36715	154	0	154	0	0.89	Clonal	0.82	1.81			polymorphism	16.15					Neutral	Tolerated
OVA_047_C1	PRIMARY	chr6	1.37E+08	1.37E+08	C	A	MAP3K5	exonic	stopgain	152X,ENSG	514	130	644	0.201863	46	0	46	0	0.67	Sub	0.6	2.91			disease_causing_automat	41	p.E905X	Passenger			NA	NA
OVA_047_M1	METASTASIS	chr6	1.38E+08	1.38E+08	TCAGTGAG	-	IFNGR1	exonic	eshift dele	00458076:	738	202	940	0.214894	79	1	80		0.69	Sub	0.47	2.01					p.P205Lfs	Passenger			NA	NA
OVA_047_M2	METASTASIS	chr6	1.38E+08	1.38E+08	TCAGTGAG	-	IFNGR1	exonic	eshift dele	00458076:	1523	338	1861	0.181623	79	1	80		0.64	Sub	0.44	2.01					p.P205Lfs	Passenger			NA	NA
OVA_047_M3	METASTASIS	chr6	1.38E+08	1.38E+08	TCAGTGAG	-	IFNGR1	exonic	eshift dele	00458076:	282	74	356	0.207865	79	1	80		0.69	Sub	0.6	3.02					p.P205Lfs	Passenger			NA	NA
OVA_013_C2	PRIMARY	chr7	1.38E+08	1.38E+08	G	A	CREB3L2	exonic	ynonymou	000004524	567	33	600	0.055	73	0	73	0	0.29	Sub	0.87	3.05	Yes	No	disease_causing	25.9	p.S191F	Passenger	SENGER/OT	damaging	Neutral	Damaging
OVA_048_M1	METASTASIS	chr3	1.38E+08	1.38E+08	CCA	-	ESYT3	intronic	NA	NA	483	226	709	0.318759	23	0	23		1	Clonal	0.56	3.94										
OVA_048_M2	METASTASIS	chr3	1.38E+08	1.38E+08	CCA	-	ESYT3	intronic	NA	NA	530	225	755	0.298013	23	0	23		1	Clonal	0.4	3.87										
OVA_048_M3	METASTASIS	chr3	1.38E+08	1.38E+08	CCA	-	ESYT3	intronic	NA	NA	512	256	768	0.333333	23	0	23		1	Clonal	0.59	3.39										
OVA_048_C1	PRIMARY	chr3	1.38E+08	1.38E+08	CCA	-	ESYT3	intronic	NA	NA	249	135	384	0.351563	23	0	23		1	Clonal	0.67	1.82										
OVA_048_C2	PRIMARY	chr3	1.38E+08	1.38E+08	CCA	-	ESYT3	intronic	NA	NA	374	206	580	0.355172	23	0	23		0.99	Clonal	0.72	1.85										
OVA_048_C3	PRIMARY	chr3	1.38E+08	1.38E+08	CCA	-	ESYT3	intronic	NA	NA	267	155	422	0.367299	23	0	23		0.99	Clonal	0.74	1.8										
OVA_047_M1	METASTASIS	chrX	1.39E+08	1.39E+08	T	G	MCF2	exonic	ynonymou	162H,ENSG	524	141	665	0.21203	46	0	46	0	0.89	Sub	0.47	2.01			polymorphism	23.1	p.N222H	Passenger	SENGER/OT	damaging	Neutral	Damaging
OVA_047_M2	METASTASIS	chrX	1.39E+08	1.39E+08	T	G	MCF2	exonic	ynonymou	162H,ENSG	526	138	664	0.207831	46	0	46	0	0.94	Sub	0.44	2.01			polymorphism	23.1	p.N222H	Passenger	SENGER/OT	damaging	Neutral	Damaging
OVA_047_M3	METASTASIS	chrX	1.39E+08	1.39E+08	T	G	MCF2	exonic	ynonymou	162H,ENSG	310	42	352	0.119318	46	0	46	0	0.52	Sub	0.6	3.02			polymorphism	23.1	p.N222H	Passenger	SENGER/OT	damaging	Neutral	Damaging
OVA_047_C1	PRIMARY	chrX	1.39E+08	1.39E+08	T	G	MCF2	exonic	ynonymou	162H,ENSG	428	277	705	0.392908	46	0	46	0	1	Clonal	0.6	2.91			polymorphism	23.1	p.N222H	Passenger	SENGER/OT	damaging	Neutral	Damaging
OVA_047_C2	PRIMARY	chrX	1.39E+08	1.39E+08	T	G	MCF2	exonic	ynonymou	162H,ENSG	413	263	676	0.389053	46	0	46	0	1	Clonal	0.64	2.76			polymorphism	23.1	p.N222H	Passenger	SENGER/OT	damaging	Neutral	Damaging
OVA_047_C3	Contralateral ovary	chrX	1.39E+08	1.39E+08	T	G	MCF2	exonic	ynonymou	162H,ENSG	350	319	669	0.476831	46	0	46	0	1	Clonal	0.6	2.94			polymorphism	23.1	p.N222H	Passenger	SENGER/OT	damaging	Neutral	Damaging
OVA_003_M1	METASTASIS	chr6	1.39E+08	1.39E+08	C	G	NHSL1	exonic	ynonymou	.532R,ENSG	123	77	200	0.385	61	0	61	0	0.98	Sub	0.57	1.84			disease_causing	25	p.G1532R	Passenger	SENGER/OT	damaging	Deleterious	Tolerated
OVA_003_M2	METASTASIS	chr6	1.39E+08	1.39E+08	C	G	NHSL1	exonic	ynonymou	.532R,ENSG	192	86	278	0.309353	61	0	61	0	1	Sub	0.46	1.81			disease_causing	25	p.G1532R	Passenger	SENGER/OT	damaging	Deleterious	Tolerated
OVA_003_M3	METASTASIS	chr6	1.39E+08	1.39E+08	C	G	NHSL1	exonic	ynonymou	.532R,ENSG	300	24	324	0.074074	61	0	61	0	0.56	Sub	0.34	2.67			disease_causing	25	p.G1532R	Passenger	SENGER/OT	damaging	Deleterious	Tolerated
OVA_003_C1	PRIMARY	chr6	1.39E+08	1.39E+08	C	G	NHSL1	exonic	ynonymou	.532R,ENSG	42	159	201	0.791045	61	0	61	0	1	Clonal	0.86	1.81			disease_causing	25	p.G1532R	Passenger	SENGER/OT	damaging	Deleterious	Tolerated
OVA_003_C2	PRIMARY	chr6	1.39E+08	1.39E+08	C	G	NHSL1	exonic	ynonymou	.532R,ENSG	69	151	220	0.686364	61	0	61	0	1	Clonal	0.7	3.61			disease_causing	25	p.G1532R	Passenger	SENGER/OT	damaging	Deleterious	Tolerated
OVA_003_C3	PRIMARY	chr6	1.39E+08	1.39E+08	C	G	NHSL1	exonic	ynonymou	.532R,ENSG	61	206	267	0.771536	61	0	61	0	1	Clonal	0.82	1.81			disease_causing	25	p.G1532R	Passenger	SENGER/OT	damaging	Deleterious	Tolerated
OVA_047_C1	PRIMARY	chr9	1.39E+08	1.39E+08	G	A	UBAC1	exonic	onymous S	000371756	209	186	395	0.470886	29	0	29	0	1	Clonal	0.6	2.91			disease_causing	9.039		Passenger			Neutral	Tolerated
OVA_047_C2	PRIMARY	chr9	1.39E+08	1.39E+08	G	A	UBAC1	exonic	onymous S	000371756	202	186	388	0.479381	29	0	29	0	1	Clonal	0.64	2.76			disease_causing	9.039		Passenger			Neutral	Tolerated
OVA_047_C3	Contralateral ovary	chr9	1.39E+08	1.39E+08	G	A	UBAC1	exonic	onymous S	000371756	209	163	372	0.438172	29	0	29	0	1	Clonal	0.6	2.94			disease_causing	9.039		Passenger			Neutral	Tolerated
OVA_048_M1	METASTASIS	chrX	1.39E+08	1.39E+08	T	A	ATP11C	exonic	ynonymou	000361648	443	221	664	0.332831	46	0	46	0	1	Sub	0.56	3.94			disease_causing	23.1	p.Y434F	Passenger	SENGER/OT	damaging	Deleterious	Damaging
OVA_048_M2	METASTASIS	chrX	1.39E+08	1.39E+08	T	A	ATP11C	exonic	ynonymou	000361648	492	140	632	0.221519	46	0	46	0	1	Sub	0.4	3.87			disease_causing	23.1	p.Y434F	Passenger	SENGER/OT	damaging	Deleterious	Damaging
OVA_048_M3	METASTASIS	chrX	1.39E+08	1.39E+08	T	A	ATP11C	exonic	ynonymou	000361648	558	18	576	0.03125	46	0	46	0	0.27	Sub	0.59	3.39			disease_causing	23.1	p.Y434F	Passenger	SENGER/OT	damaging	Deleterious	Damaging
OVA_047_M1	METASTASIS	chr9	1.39E+08	1.39E+08	G	C	QSOX2	exonic	ynonymou	000003587	196	84	280	0.3	35	0	35	0	0.96	Clonal	0.47	2.01			disease_causing	11.64	p.L278V	Passenger	SENGER/OT	damaging	Neutral	Tolerated
OVA_047_M2	METASTASIS	chr9	1.39E+08	1.39E+08	G	C	QSOX2	exonic	ynonymou	000003587	214	111	325	0.341538	35	0	35	0	1	Clonal	0.44	2.01			disease_causing	11.64	p.L278V	Passenger	SENGER/OT	damaging	Neutral	Tolerated
OVA_047_M3	METASTASIS	chr9	1.39E+08	1.39E+08	G	C	QSOX2	exonic	ynonymou	000003587	97	63	160	0.39375	35	0	35	0	1	Clonal	0.6	3.02			disease_causing	11.64	p.L278V	Passenger	SENGER/OT	damaging	Neutral	Tolerated
OVA_047_C1	PRIMARY	chr9	1.39E+08	1.39E+08	G	C	QSOX2	exonic	ynonymou	000003587	126	312	438	0.712329	35	0	35	0	1	Clonal	0.6	2.91			disease_causing	11.64	p.L278V	Passenger	SENGER/OT	damaging	Neutral	Tolerated
OVA_047_C2	PRIMARY	chr9	1.39E+08	1.39E+08	G	C	QSOX2	exonic	ynonymou	000003587	104	251	355	0.707042	35	0	35	0	1	Clonal	0.64	2.76			disease_causing	11.64	p.L278V	Passenger	SENGER/OT	damaging	Neutral	Tolerated
OVA_047_C3	Contralateral ovary	chr9	1.39E+08	1.39E+08	G	C	QSOX2	exonic	ynonymou	000003587	100	241	341	0.706745	35	0	35	0	1	Clonal	0.6	2.94			disease_causing	11.64	p.L278V	Passenger	SENGER/OT	damaging	Neutral	Tolerated
OVA_378_C2	PRIMARY	chr8	1.39E+08	1.39E+08	T	G	FAM135B	intronic	NA	NA	337	73	410	0.178049	47	0	47	0	0.81	Sub	0.56	2.59	Yes		polymorphism	5.007						
OVA_047_C1	PRIMARY	chr9	1.39E+08	1.39E+08	T	A	SNAPC4	exonic	ynonymou	000298532	121	40	161	0.248447	45	0	45	0	1	Clonal	0.6	2.91			polymorphism	5.865	p.T1373S	Passenger	SENGER/OT	benign	Neutral	Tolerated
OVA_047_C2	PRIMARY	chr9	1.39E+08	1.39E+08	T	A	SNAPC4	exonic	ynonymou	000298532	117	41	158	0.259494	45	0	45	0	1	Clonal	0.64	2.76			polymorphism	5.865	p.T1373S	Passenger	SENGER/OT	benign	Neutral	Tolerated
OVA_047_C3	Contralateral ovary	chr9	1.39E+08	1.39E+08	T	A	SNAPC4	exonic	ynonymou	000298532	86	23	109	0.211009	45	0	45	0	0.92	Clonal	0.6	2.94			polymorphism	5.865	p.T1373S	Passenger	SENGER/OT	benign	Neutral	Tolerated
OVA_013_M1	METASTASIS	chr9	1.39E+08	1.39E+08	A	C	SEC16A	intronic	NA	NA	245	69	314	0.219745	13	0	13	0	1	Clonal	0.67	3.06			polymorphism	0.409						
OVA_013_C1	PRIMARY	chr9	1.39E+08	1.39E+08	A	C	SEC16A	intronic	NA	NA	278	91	369	0.246612	13	0	13	0	1	Sub	0.72	3.1			polymorphism	0.409						
OVA_013_C2	PRIMARY	chr9	1.39E+08	1.39E+08	A	C	SEC16A	intronic	NA	NA	261	94	355	0.264789	13	0	13	0	0.87	Sub	0.87	3.05			polymorphism	0.409						
OVA_013_C3	PRIMARY	chr9	1.39E+08	1.39E+08	A	C	SEC16A	intronic	NA	NA	313	106	419	0.252983	13	0	13	0	0.81	Sub	0.91	3.01			polymorphism	0.409						
OVA_013_M1	METASTASIS	chr7	1.4E+08	1.4E+08	G	A	TBXAS1	exonic	ynonymou	000416849	396	93	489	0.190184	44	0	44	0	1	Clonal	0.67	3.06			disease_causing	21.2	p.D247N	Passenger	SENGER/OT	benign	Deleterious	Tolerated
OVA_048_M1	METASTASIS	chr9	1.4E+08	1.4E+08	C	T	FUT7	exonic	ynonymou	000031441	56	28	84	0.333333	66	1	67	0.014925	1	Clonal	0.56	3.94			polymorphism	16.64	p.R145H	Passenger	SENGER/OT	damaging	Deleterious	Damaging
OVA_048_M2	METASTASIS	chr9	1.4E+08	1.4E+08	C	T	FUT7	exonic	ynonymou	000031441	70	27	97	0.278351	66	1	67	0.014925	1	Clonal	0.4	3.87			polymorphism	16.64	p.R145H	Passenger	SENGER/OT	damaging	Deleterious	Damaging
OVA_048_M3	METASTASIS	chr9	1.4E+08	1.4E+08	C	T	FUT7	exonic	ynonymou	000031441	71	23	94	0.244681	66	1	67	0.014925	1	Clonal	0.59	3.39			polymorphism	16.64	p.R145H	Passenger	SENGER/OT	damaging	Deleterious	Damaging
OVA_048_C1	PRIMARY	chr9	1.4E+08	1.4E+08	C	T	FUT7	exonic	ynonymou	000031441	29	10	39	0.25641	66	1	67	0.014925	0.51	Sub	0.67	1.82			polymorphism	16.64	p.R145H	Passenger	SENGER/OT	damaging	Deleterious	Damaging
OVA_048_C2	PRIMARY	chr9	1.4E+08	1.4E+08	C	T	FUT7	exonic	ynonymou	000031441	66	34	100	0.34	66	1	67	0.014925	0.95	Sub	0.72	1.85			polymorphism	16.64	p.R145H	Passenger	SENGER/OT	damaging	Deleterious	Damaging
OVA_048_C3	PRIMARY	chr9	1.4E+08	1.4E+08	C	T	FUT7	exonic	ynonymou	000031441	42	25	67	0.373134	66	1	67	0.014925	1	Sub	0.74	1.8			polymorphism	16.64	p.R145H	Passenger	SENGER/OT	damaging	Deleterious	Damaging
OVA_378_C1	PRIMARY	chr7	1.4E+08	1.4E+08	G	A	RAB19	exonic	ynonymou	R88H,ENSG	418	133	551	0.241379	67	0	67	0	1	Clonal	0.59	2.6			disease_causing	27.9	p.R88H	Passenger	CANCER	damaging	Deleterious	Damaging
OVA_378_C2	PRIMARY	chr7	1.4E+08	1.4E+08	G	A	RAB19	exonic	ynonymou	R88H,ENSG	368	105	473	0.221987	67	0	67	0	1	Clonal	0.56	2.59			disease_causing	27.9	p.R88H	Passenger	CANCER	damaging	Deleterious	Damaging
OVA_378_C3	PRIMARY	chr7	1.4E+08	1.4E+08	G	A	RAB19	exonic	ynonymou	R88H,ENSG	389	87	476	0.182773	67	0	67	0	0.9	Clonal	0.51	2.6			disease_causing	27.9	p.R88H	Passenger	CANCER	damaging	Deleterious	Damaging
OVA_048_C3	PRIMARY	chr9	1.4E+08	1.4E+08	C	-	TOR4A	exonic	eshift dele	000357503	205	67	272	0.246324	80	0	80		0.67	Sub	0.74	1.8					p.P205Lfs	Passenger			NA	NA
OVA_003_M1	METASTASIS	chr5	1.4E+08	1.4E+08	G	C	PCDHA13	exonic	ynonymou	552H,ENSG	287	112	399	0.280702	168	1	169	0.005917	0.99	Sub	0.57	1.84			polymorphism	23.1	p.Q552H	Passenger	SENGER/OT	benign	Deleterious	Tolerated
OVA_003_M2	METASTASIS	chr5	1.4E+08	1.4E+08	G	C	PCDHA13	exonic	ynonymou	552H,ENSG	594	263	587	0.448041	168	1	169	0.005917	1	Sub	0.46	1.81			polymorphism	23.1	p.Q552H	Passenger	SENGER/OT	benign	Deleterious	Tolerated
OVA_003_M3	METASTASIS	chr5	1.4E+08	1.4E+08	G	C	PCDHA13	exonic	ynonymou	552H,ENSG	475	36	511	0.07045	168	1	169	0.005917	0.81	Sub	0.34	2.67			polymorphism	23.1	p.Q552H	Passenger	SENGER/OT	benign	Deleterious	Tolerated
OVA_003_C1	PRIMARY	chr5	1.4E+08	1.4E+08	G	C	PCDHA13	exonic	ynonymou	552H,ENSG	331	232	563	0.412078	168	1	169	0.005917	0.96	Clonal	0.86	1.81			polymorphism	23.1	p.Q552H	Passenger	SENGER/OT	benign	Deleterious	Tolerated
OVA_003_C2	PRIMARY	chr5	1.4E+08	1.4E+08	G	C	PCDHA13	exonic	ynonymou	552H,ENSG	323	252	575	0.438261	168	1	169	0.005917	1	Clonal	0.7	3.61			polymorphism	23.1	p.Q552H	Passenger	SENGER/OT	benign	Deleterious	Tolerated
OVA_003_C3	PRIMARY	chr5	1.4E+08	1.4E+08	G	C	PCDHA13	exonic	ynonymou	552H,ENSG	349	245	594	0.412458	168	1	169	0.005917	1	Clonal	0.82	1.81			polymorphism	23.1	p.Q552H	Passenger	SENGER/OT	benign	Deleterious	Tolerated
OVA_378_M1	METASTASIS	chr5	1.41E+08	1.41E+08	G	A	PCDHB17	exonic	ynonymou	000053953	241	194	435	0.445977	46	0	46	0	1	Clonal	0.72	2.57				13.22				benign	Neutral	Tolerated
OVA_378_M2	METASTASIS	chr5	1.41E+08	1.41E+08	G	A	PCDHB17	exonic	ynonymou	000053953	266	211	477	0.442348	46	0	46	0	1	Clonal	0.85	2.57				13.22				benign	Neutral	Tolerated
OVA_378_M3	METASTASIS	chr5	1.41E+08	1.41E+08	G	A	PCDHB17	exonic	ynonymou	000053953	336	251	587	0.427598	46	0	46	0	1	Clonal	0.67	2.57				13.22				benign	Neutral	Tolerated
OVA_378_C1	PRIMARY	chr5	1.41E+08	1.41E+08	G	A	PCDHB17	exonic	ynonymou	000053953	348	206	554	0.371841	46	0	46	0	1	Clonal	0.59	2.6				13.22				benign	Neutral	Tolerated
OVA_378_C2	PRIMARY	chr5	1.41E+08	1.41E+08	G	A	PCDHB17	exonic	ynonymou	000053953	298	133	431	0.308585	46	0	46	0	1	Clonal	0.56	2.59				13.22				benign	Neutral	Tolerated
OVA_378_C3	PRIMARY	chr5	1.41E+08	1.41E+08	G	A	PCDHB17	exonic	ynonymou	000053953	300	124	424	0.292453	46	0	46	0	1	Clonal	0.51	2.6				13.22				benign	Neutral	Tolerated
OVA_047_C1	PRIMARY	chr5	1.41E+08	1.41E+08	C	T	PCDHB17	exonic	onymous S	000053953	443	178	621	0.286634	33	0	33	0	1	Clonal	0.6	2.91				11.98					Neutral	Tolerated
OVA_047_C2	PRIMARY	chr5	1.41E+08	1.41E+08	C	T	PCDHB17	exonic	onymous S	000053953	406	178	584	0.304795	33	0	33	0	1	Clonal	0.64	2.76				11.98					Neutral	Tolerated
OVA_047_C3	Contralateral ovary	chr5	1.41E+08	1.41E+08	C	T	PCDHB17	exonic	onymous S	000053953	423	197	620	0.317742	33	0	33	0	1	Clonal	0.6	2.94				11.98					Neutral	Tolerated

Sample	Type	Chr	Start	End	Ref	Alt	Gene	unc.ensGen	icFunc.ens	hange.ens	t_ref	t_alt	t_total	t_VAF	n_ref	n_alt	n_total	normal_VA	ncer_cell_f	on-based C	purity	ploidy	Cancer Gene	Driver Gene	Mutation Taster	ADD(PHRE	otein_chan	CHASM	THMM_Pr	OLYPHEN-	REDICTION	ICTION (cutoff=0.05)
OVA_048_M1	METASTASIS	chr5	1.41E+08	1.41E+08	G	T	PCDHGA4	exonic	ynonymou	000057125	456	65	521	0.12476	52	1	53	0.018868	0.57	Sub	0.56	3.94			disease_causing	24.7	p.G543V	Passenger		damaging		
OVA_013_M1	METASTASIS	chr5	1.41E+08	1.41E+08	C	A	PCDHGB5	intronic	NA	NA	423	168	591	0.284264	35	0	35	0	0.85	Sub	0.67	3.06			polymorphism	15.61	p.T794N	Passenger		benign		
OVA_013_C1	PRIMARY	chr5	1.41E+08	1.41E+08	C	A	PCDHGB5	intronic	NA	NA	378	204	582	0.350515	35	0	35	0	1	Sub	0.72	3.1			polymorphism	15.61	p.T794N	Passenger		benign		
OVA_013_C2	PRIMARY	chr5	1.41E+08	1.41E+08	C	A	PCDHGB5	intronic	NA	NA	350	221	571	0.38704	35	0	35	0	0.89	Sub	0.87	3.05			polymorphism	15.61	p.T794N	Passenger		benign		
OVA_013_C3	PRIMARY	chr5	1.41E+08	1.41E+08	C	A	PCDHGB5	intronic	NA	NA	366	232	598	0.38796	35	0	35	0	1	Sub	0.91	3.01			polymorphism	15.61	p.T794N	Passenger		benign		
OVA_048_M1	METASTASIS	chrX	1.41E+08	1.41E+08	G	A	MAGEC1	UTR3	NA	NA	313	205	518	0.395753	19	0	19	0	1	Clonal	0.56	3.94			polymorphism	0.537						
OVA_048_M2	METASTASIS	chrX	1.41E+08	1.41E+08	G	A	MAGEC1	UTR3	NA	NA	352	160	512	0.3125	19	0	19	0	1	Clonal	0.4	3.87			polymorphism	0.537						
OVA_048_M3	METASTASIS	chrX	1.41E+08	1.41E+08	G	A	MAGEC1	UTR3	NA	NA	146	223	369	0.604336	19	0	19	0	1	Clonal	0.59	3.39			polymorphism	0.537						
OVA_048_C1	PRIMARY	chrX	1.41E+08	1.41E+08	G	A	MAGEC1	UTR3	NA	NA	186	151	337	0.448071	19	0	19	0	1	Clonal	0.67	1.82			polymorphism	0.537						
OVA_048_C2	PRIMARY	chrX	1.41E+08	1.41E+08	G	A	MAGEC1	UTR3	NA	NA	315	175	490	0.357143	19	0	19	0	0.99	Clonal	0.72	1.85			polymorphism	0.537						
OVA_048_C3	PRIMARY	chrX	1.41E+08	1.41E+08	G	A	MAGEC1	UTR3	NA	NA	247	154	401	0.38404	19	0	19	0	1	Clonal	0.74	1.8			polymorphism	0.537						
OVA_003_M1	METASTASIS	chr5	1.41E+08	1.41E+08	C	G	FCHSD1	intronic	NA	NA	138	42	180	0.233333	64	0	64	0	0.83	Sub	0.57	1.84			polymorphism	6.925						
OVA_003_M2	METASTASIS	chr5	1.41E+08	1.41E+08	C	G	FCHSD1	intronic	NA	NA	162	52	214	0.242991	64	0	64	0	1	Sub	0.46	1.81			polymorphism	6.925						
OVA_003_M3	METASTASIS	chr5	1.41E+08	1.41E+08	C	G	FCHSD1	intronic	NA	NA	188	18	206	0.087379	64	0	64	0	0.92	Sub	0.34	2.67			polymorphism	6.925						
OVA_003_C1	PRIMARY	chr5	1.41E+08	1.41E+08	C	G	FCHSD1	intronic	NA	NA	115	93	208	0.447115	64	0	64	0	1	Clonal	0.86	1.81			polymorphism	6.925						
OVA_003_C2	PRIMARY	chr5	1.41E+08	1.41E+08	C	G	FCHSD1	intronic	NA	NA	148	94	242	0.38843	64	0	64	0	1	Clonal	0.7	3.61			polymorphism	6.925						
OVA_003_C3	PRIMARY	chr5	1.41E+08	1.41E+08	C	G	FCHSD1	intronic	NA	NA	142	117	259	0.451737	64	0	64	0	1	Clonal	0.82	1.81			polymorphism	6.925						
OVA_365_C1	Contralateral ovary	chr7	1.42E+08	1.42E+08	C	A	2S1;TCRBV	intronic	NA	NA	160	26	186	0.139785	52	0	52	0	0.81	Clonal	0.42	1.83				4.065						
OVA_047_C1	PRIMARY	chr4	1.43E+08	1.43E+08	C	A	IL15	intronic	NA	NA	458	222	680	0.326471	36	0	36	0	1	Clonal	0.6	2.91			polymorphism	0.068						
OVA_047_C2	PRIMARY	chr4	1.43E+08	1.43E+08	C	A	IL15	intronic	NA	NA	450	225	675	0.333333	36	0	36	0	1	Clonal	0.64	2.76			polymorphism	0.068						
OVA_047_C3	Contralateral ovary	chr4	1.43E+08	1.43E+08	C	A	IL15	intronic	NA	NA	476	205	681	0.301028	36	0	36	0	1	Clonal	0.6	2.94			polymorphism	0.068						
OVA_013_M1	METASTASIS	chr3	1.43E+08	1.43E+08	G	A	SLC9A9	exonic	nonymous S	000316549	422	140	562	0.24911	42	0	42	0	0.99	Clonal	0.67	3.06			disease_causing	16.22		Passenger			Neutral	Tolerated
OVA_013_C1	PRIMARY	chr3	1.43E+08	1.43E+08	G	A	SLC9A9	exonic	nonymous S	000316549	413	174	587	0.296422	42	0	42	0	1	Sub	0.72	3.1			disease_causing	16.22		Passenger			Neutral	Tolerated
OVA_013_C2	PRIMARY	chr3	1.43E+08	1.43E+08	G	A	SLC9A9	exonic	nonymous S	000316549	370	182	552	0.32971	42	0	42	0	1	Sub	0.87	3.05			disease_causing	16.22		Passenger			Neutral	Tolerated
OVA_013_C3	PRIMARY	chr3	1.43E+08	1.43E+08	G	A	SLC9A9	exonic	nonymous S	000316549	453	162	615	0.263415	42	0	42	0	0.84	Sub	0.91	3.01			disease_causing	16.22		Passenger			Neutral	Tolerated
OVA_048_C1	PRIMARY	chr7	1.44E+08	1.44E+08	A	G	OR6B1	exonic	ynonymou	000040892	405	32	437	0.073227	37	0	37	0	0.15	Sub	0.67	1.82			disease_causing	25.3	p.Y218C	Passenger	SENGER/OT	damaging	Deleterious	Damaging
OVA_047_M1	METASTASIS	chr8	1.44E+08	1.44E+08	C	G	JRK	cRNA_exon	NA	NA	371	53	424	0.125	47	0	47	0	1	Clonal	0.47	2.01				0.989	p.V492L	Passenger		benign		
OVA_047_M2	METASTASIS	chr8	1.44E+08	1.44E+08	C	G	JRK	cRNA_exon	NA	NA	362	44	406	0.108374	47	0	47	0	1	Clonal	0.44	2.01				0.989	p.V492L	Passenger		benign		
OVA_047_M3	METASTASIS	chr8	1.44E+08	1.44E+08	C	G	JRK	cRNA_exon	NA	NA	111	15	126	0.119048	47	0	47	0	1	Clonal	0.6	3.02				0.989	p.V492L	Passenger		benign		
OVA_365_M1	METASTASIS	chr8	1.44E+08	1.44E+08	A	G	GML	exonic	nonymous S	.T57T,ENSG	200	376	576	0.652778	33	0	33	0	1	Clonal	0.85	1.87			polymorphism	0.05		Passenger			Neutral	Tolerated
OVA_365_M2	METASTASIS	chr8	1.44E+08	1.44E+08	A	G	GML	exonic	nonymous S	.T57T,ENSG	268	197	465	0.423656	33	0	33	0	1	Clonal	0.4	1.81			polymorphism	0.05		Passenger			Neutral	Tolerated
OVA_365_M3	METASTASIS	chr8	1.44E+08	1.44E+08	A	G	GML	exonic	nonymous S	.T57T,ENSG	195	389	584	0.666096	33	0	33	0	1	Clonal	0.82	1.89			polymorphism	0.05		Passenger			Neutral	Tolerated
OVA_365_C1	Contralateral ovary	chr8	1.44E+08	1.44E+08	A	G	GML	exonic	nonymous S	.T57T,ENSG	302	174	476	0.365546	33	0	33	0	1	Clonal	0.42	1.83			polymorphism	0.05		Passenger			Neutral	Tolerated
OVA_365_C2	PRIMARY	chr8	1.44E+08	1.44E+08	A	G	GML	exonic	nonymous S	.T57T,ENSG	246	283	529	0.534972	33	0	33	0	1	Clonal	0.62	1.92			polymorphism	0.05		Passenger			Neutral	Tolerated
OVA_013_M1	METASTASIS	chr8	1.45E+08	1.45E+08	C	T	SCRIB	exonic	nonymous S	V17V,ENSG	72	40	112	0.357143	85	0	85	0	1	Clonal	0.67	3.06			disease_causing	22.3		Passenger			Neutral	Tolerated
OVA_013_C1	PRIMARY	chr8	1.45E+08	1.45E+08	C	T	SCRIB	exonic	nonymous S	V17V,ENSG	87	49	136	0.360294	85	0	85	0	1	Sub	0.72	3.1			disease_causing	22.3		Passenger			Neutral	Tolerated
OVA_013_C2	PRIMARY	chr8	1.45E+08	1.45E+08	C	T	SCRIB	exonic	nonymous S	V17V,ENSG	70	33	103	0.320388	85	0	85	0	0.74	Sub	0.87	3.05			disease_causing	22.3		Passenger			Neutral	Tolerated
OVA_013_C3	PRIMARY	chr8	1.45E+08	1.45E+08	C	T	SCRIB	exonic	nonymous S	V17V,ENSG	97	73	170	0.429412	85	0	85	0	0.94	Sub	0.91	3.01			disease_causing	22.3		Passenger			Neutral	Tolerated
OVA_047_C1	PRIMARY	chr2	1.45E+08	1.45E+08	G	T	ZEB2	exonic	nonymous S	000040948	549	167	716	0.23324	36	0	36	0	1	Sub	0.6	2.91			disease_causing	10.21		Passenger			Neutral	Tolerated
OVA_047_C2	PRIMARY	chr2	1.45E+08	1.45E+08	G	T	ZEB2	exonic	nonymous S	000040948	602	89	691	0.128799	36	0	36	0	0.66	Sub	0.64	2.76			disease_causing	10.21		Passenger			Neutral	Tolerated
OVA_047_C3	Contralateral ovary	chr2	1.45E+08	1.45E+08	G	T	ZEB2	exonic	nonymous S	000040948	584	102	686	0.148688	36	0	36	0	0.8	Sub	0.6	2.94			disease_causing	10.21		Passenger			Neutral	Tolerated
OVA_013_M1	METASTASIS	chr1	1.45E+08	1.45E+08	T	A	TXNIP	UTR5	NA	NA	478	152	630	0.24127	76	0	76	0	0.96	Clonal	0.67	3.06			polymorphism	11.58						
OVA_013_C1	PRIMARY	chr1	1.45E+08	1.45E+08	T	A	TXNIP	UTR5	NA	NA	406	202	608	0.332237	76	0	76	0	1	Clonal	0.72	3.1			polymorphism	11.58						
OVA_013_C2	PRIMARY	chr1	1.45E+08	1.45E+08	T	A	TXNIP	UTR5	NA	NA	367	196	563	0.348135	76	0	76	0	1	Clonal	0.87	3.05			polymorphism	11.58						
OVA_013_C3	PRIMARY	chr1	1.45E+08	1.45E+08	T	A	TXNIP	UTR5	NA	NA	444	196	640	0.30625	76	0	76	0	0.98	Clonal	0.91	3.01			polymorphism	11.58						
OVA_378_C1	PRIMARY	chr1	1.46E+08	1.46E+08	C	T	ITGA10	exonic	ynonymou	000053881	506	103	609	0.16913	49	0	49	0	0.91	Clonal	0.59	2.6			disease_causing	18.68				benign	Neutral	Damaging
OVA_378_C2	PRIMARY	chr1	1.46E+08	1.46E+08	C	T	ITGA10	exonic	ynonymou	000053881	460	94	554	0.169675	49	0	49	0	0.94	Clonal	0.56	2.59			disease_causing	18.68				benign	Neutral	Damaging
OVA_378_C3	PRIMARY	chr1	1.46E+08	1.46E+08	C	T	ITGA10	exonic	ynonymou	000053881	476	86	562	0.153025	49	0	49	0	0.91	Clonal	0.51	2.6			disease_causing	18.68				benign	Neutral	Damaging
OVA_013_M1	METASTASIS	chr8	1.46E+08	1.46E+08	C	T	ADCK5	intronic	NA	NA	24	7	31	0.225806	40	0	40	0	0.67	Clonal	0.67	3.06			disease_causing	13.73						
OVA_013_C1	PRIMARY	chr8	1.46E+08	1.46E+08	C	T	ADCK5	intronic	NA	NA	15	7	22	0.318182	40	0	40	0	0.88	Sub	0.72	3.1			disease_causing	13.73						
OVA_013_C2	PRIMARY	chr8	1.46E+08	1.46E+08	C	T	ADCK5	intronic	NA	NA	17	10	27	0.37037	40	0	40	0	0.85	Sub	0.87	3.05			disease_causing	13.73						
OVA_013_C3	PRIMARY	chr8	1.46E+08	1.46E+08	C	T	ADCK5	intronic	NA	NA	33	12	45	0.266667	40	0	40	0	0.58	Sub	0.91	3.01			disease_causing	13.73						
OVA_047_C1	PRIMARY	chr8	1.46E+08	1.46E+08	G	C	TONSL	exonic	ynonymou	000040937	653	84	737	0.113976	102	0	102	0	1	Clonal	0.6	2.91			disease_causing	22.5	p.I211M	Passenger	SENGER/OT	damaging	Neutral	Damaging
OVA_047_C2	PRIMARY	chr8	1.46E+08	1.46E+08	G	C	TONSL	exonic	ynonymou	000040937	567	123	690	0.178261	102	0	102	0	1	Clonal	0.64	2.76			disease_causing	22.5	p.I211M	Passenger	SENGER/OT	damaging	Neutral	Damaging
OVA_047_C3	Contralateral ovary	chr8	1.46E+08	1.46E+08	G	C	TONSL	exonic	ynonymou	000040937	617	70	687	0.101892	102	0	102	0	0.95	Clonal	0.6	2.94			disease_causing	22.5	p.I211M	Passenger	SENGER/OT	damaging	Neutral	Damaging
OVA_003_C1	PRIMARY	chr3	1.47E+08	1.47E+08	A	C	ZIC1	exonic	ynonymou	000002829	377	14	391	0.035806	52	0	52	0	0.08	Sub	0.86	1.81			disease_causing	23.7	p.D27A	Passenger	SENGER/OT	benign	Neutral	Damaging
OVA_013_M1	METASTASIS	chr5	1.48E+08	1.48E+08	C	T	SH3TC2	exonic	stopgain	W120X,ENS	276	101	377	0.267905	38	1	39	0.025641	0.8	Sub	0.67	3.06	Yes	No	isease_causing_automat	38	p.W120X	Passenger			NA	NA
OVA_003_M1	METASTASIS	chr7	1.49E+08	1.49E+08	G	A	KRBA1	exonic	ynonymou	000319551	136	56	192	0.291667	72	0	72	0	1	Clonal	0.57	1.84			polymorphism	13.05	p.D735N	Passenger		benign	Neutral	Tolerated
OVA_003_M2	METASTASIS	chr7	1.49E+08	1.49E+08	G	A	KRBA1	exonic	ynonymou	000319551	168	50	218	0.229358	72	0	72	0	1	Clonal	0.46	1.81			polymorphism	13.05	p.D735N	Passenger		benign	Neutral	Tolerated
OVA_003_C1	PRIMARY	chr7	1.49E+08	1.49E+08	G	A	KRBA1	exonic	ynonymou	000319551	137	113	250	0.452	72	0	72	0	1	Clonal	0.86	1.81			polymorphism	13.05	p.D735N	Passenger		benign	Neutral	Tolerated
OVA_003_C2	PRIMARY	chr7	1.49E+08	1.49E+08	G	A	KRBA1	exonic	ynonymou	000319551	191	120	311	0.385852	72	0	72	0	1	Clonal	0.7	3.61			polymorphism	13.05	p.D735N	Passenger		benign	Neutral	Tolerated
OVA_003_C3	PRIMARY	chr7	1.49E+08	1.49E+08	G	A	KRBA1	exonic	ynonymou	000319551	164	124	288	0.430556	72	0	72	0	1	Clonal	0.82	1.81			polymorphism	13.05	p.D735N	Passenger		benign	Neutral	Tolerated
OVA_378_C3	PRIMARY	chr7	1.5E+08	1.5E+08	C	G	SSPO	RNA_intro	NA	NA	193	34	227	0.14978	71	0	71	0	0.74	Sub	0.51	2.6				3.946						
OVA_048_M1	METASTASIS	chr1	1.5E+08	1.5E+08	A	T	SV2A	exonic	ynonymou	420R,ENSG	136	7	143	0.048951	26	0	26	0	0.27	Sub	0.56	3.94			disease_causing	26	p.W420R	Passenger	SENGER/OT	damaging	Deleterious	Tolerated
OVA_048_M3	METASTASIS	chr1	1.5E+08	1.5E+08	A	T	SV2A	exonic	ynonymou	420R,ENSG	104	20	124	0.16129	26	0	26	0	1	Sub	0.59	3.39			disease_causing	26	p.W420R	Passenger	SENGER/OT	damaging	Deleterious	Tolerated
OVA_365_M1	METASTASIS	chr5	1.5E+08	1.5E+08	G	T	RBM22	UTR3	NA	NA	178	143	321	0.445483	71	1	72	0.013889	1	Clonal	0.85	1.87			polymorphism	5.406						
OVA_365_M2	METASTASIS	chr5	1.5E+08	1.5E+08	G	T	RBM22	UTR3	NA	NA	82	29	111	0.261261	71	1	72	0.013889	1	Clonal	0.4	1.81			polymorphism	5.406						
OVA_365_M3	METASTASIS	chr5	1.5E+08	1.5E+08	G	T	RBM22	UTR3	NA	NA	120	93	213	0.43662	71	1	72	0.013889	1	Clonal	0.82	1.89			polymorphism	5.406						
OVA_048_M1	METASTASIS	chr5	1.5E+08	1.5E+08	C	T	RBM22	exonic	ynonymou	000001998	238	339	577	0.587522	42	0	42	0	1	Clonal	0.56	3.94			disease_causing	23.9	p.C58Y	Passenger	SENGER/OT	damaging	Deleterious	Tolerated
OVA_048_M2	METASTASIS	chr5	1.5E+08	1.5E+08	C	T	RBM22	exonic	ynonymou	000001998	344	220	564	0.390071	42	0	42	0	1	Clonal	0.4	3.87			disease_causing	23.9	p.C58Y	Passenger	SENGER/OT	damaging	Deleterious	Tolerated
OVA_048_M3	METASTASIS	chr5	1.5E+08	1.5E+08	C	T	RBM22	exonic	ynonymou	000001998	230	336	566	0.59364	42	0	42	0	1	Clonal	0.59	3.39			disease_causing	23.9	p.C58Y	Passenger	SENGER/OT	damaging	Deleterious	Tolerated
OVA_048_C1	PRIMARY	chr5	1.5E+08	1.5E+08	C	T	RBM22	exonic	ynonymou	000001998	193	226	419	0.539379	42	0	42	0	1	Clonal	0.67	1.82			disease_causing	23.9	p.C58Y	Passenger	SENGER/OT	damaging	Deleterious	Tolerated
OVA_048_C2	PRIMARY	chr5	1.5E+08	1.5E+08	C	T	RBM22	exonic	ynonymou	000001998	234	308	542	0.568266	42	0	42	0	1	Clonal	0.72	1.85			disease_causing	23.9	p.C58Y	Passenger	SENGER/OT	damaging	Deleterious	Tolerated
OVA_048_C3	PRIMARY	chr5	1.5E+08	1.5E+08	C	T	RBM22	exonic	ynonymou	000001998	172	229	401	0.571072	42	0	42	0	0.97	Clonal	0.74	1.8			disease_causing	23.9	p.C58Y	Passenger	SENGER/OT	damaging	Deleterious	Tolerated
OVA_047_M1	METASTASIS	chr7	1.5E+08	1.5E+08	A	T	GIMAP7	exonic	ynonymou	000031354	624	94	718	0.130919	64	0	64	0	0.81	Sub	0.47	2.01			polymorphism	0.45	p.K191N	Passenger	SENGER/OT	benign	Neutral	Tolerated
OVA_047_M2	METASTASIS	chr7	1.5E+08	1.5E+08	A	T	GIMAP7	exonic	ynonymou	000031354	579	135	714	0.189076	64	0	64	0	1	Sub	0.44	2.01			polymorphism	0.45	p.K191N	Passenger	SENGER/OT	benign	Neutral	Tolerated
OVA_047_M3	METASTASIS	chr7	1.5E+08	1.5E+08	A	T	GIMAP7	exonic	ynonymou	000031354	486	133	619	0.214863	64	0	64	0	1	Sub	0.6	3.02			polymorphism	0.45	p.K191N	Passenger	SENGER/OT	benign	Neutral	Tolerated
OVA_365_M1	METASTASIS	chr7	1.51E+08	1.51E+08	G	A	NOS3	UTR3	NA	NA	279	261	540	0.483333	49	0	49	0	1	Clonal	0.85	1.87			polymorphism	0.281						
OVA_365_M2	METASTASIS	chr7	1.51E+08	1.51E+08	G	A	NOS3	UTR3	NA	NA	308	140	448	0.3125	49	0	49	0	1	Clonal	0.4	1.81			polymorphism	0.281						
OVA_365_M3	METASTASIS	chr7	1.51E+08	1.51E+08	G	A	NOS3	UTR3	NA	NA	298	238	536	0.44403	49	0	49	0	1	Clonal	0.82	1.89			polymorphism	0.281						
OVA_365_C1	Contralateral ovary	chr7	1.51E+08	1.51E+08	G	A	NOS3	UTR3	NA	NA	380	105	485	0.216495	49	0	49	0	1	Clonal	0.42	1.83			polymorphism	0.281						
OVA_365_C2	PRIMARY	chr7	1.51E+08	1.51E+08	G	A	NOS3	UTR3	NA	NA	397	151	548	0.275547	49	0	49	0	1	Clonal	0.62	1.92			polymorphism	0.281						
OVA_003_M1	METASTASIS	chr7	1.51E+08	1.51E+08	C	-	FASTK	UTR3	NA	NA	92	33	125	0.264	56	0	56		0.93	Clonal	0.57	1.84										
OVA_003_M2	METASTASIS	chr7	1.51E+08	1.51E+08	C	-	FASTK	UTR3	NA	NA	123	39	162	0.240741	56	0	56		1	Clonal	0.46	1.81										
OVA_003_C1	PRIMARY	chr7	1.51E+08	1.51E+08	C	-	FASTK	UTR3	NA	NA	128	69	197	0.350254	56	0	56		0.81	Sub	0.86	1.81										
OVA_003_C2	PRIMARY	chr7	1.51E+08	1.51E+08	C	-	FASTK	UTR3	NA	NA	164	81	245	0.330612	56	0	56		1	Sub	0.7	3.61										
OVA_003_C3	PRIMARY	chr7	1.51E+08	1.51E+08	C	-	FASTK	UTR3	NA	NA	160	95	255	0.372549	56	0	56		0.9	Sub	0.82	1.81										
OVA_047_M1	METASTASIS	chr1	1.51E+08	1.51E+08	G	T	ARNT	exonic	ynonymou	000035859	593	101	694	0.145533	31	0	31	0	0.9	Clonal	0.47	2.01	Yes	Yes	disease_causing	27.3	p.Q201K	Passenger	SENGER/OT	damaging	Deleterious	Damaging
OVA_047_M2	METASTASIS	chr1	1.51E+08	1.51E+08	G	T	ARNT	exonic	ynonymou	000035859	608	112	720	0.155556	31	0	31	0	1	Clonal	0.44	2.01	Yes	Yes	disease_causing	27.3	p.Q201K	Passenger	SENGER/OT	damaging	Deleterious	Damaging
OVA_047_M3	METASTASIS	chr1	1.51E+08	1.51E+08	G	T	ARNT	exonic	ynonymou	000035859	501	106	607	0.174629	31	0	31	0	1	Clonal	0.6	3.02	Yes	Yes	disease_causing	27.3	p.Q201K	Passenger	SENGER/OT	damaging	Deleterious	Damaging
OVA_047_M1	METASTASIS	chr3	1.51E+08	1.51E+08	T	C	P2RY14	exonic	ynonymou	277R,ENSG	541	182	723	0.251729	60	0	60	0	1	Clonal	0.47	2.01			disease_causing	26.6	p.K277R	Passenger	SENGER/OT	damaging	Deleterious	Damaging
OVA_047_M2	METASTASIS	chr3	1.51E+08	1.51E+08	T	C	P2RY14	exonic	ynonymou	277R,ENSG	532	222	754	0.29443	60	0	60	0	1	Clonal	0.44	2.01			disease_causing	26.6	p.K277R	Passenger	SENGER/OT	damaging	Deleterious	Damaging
OVA_047_M3	METASTASIS	chr3	1.51E+08	1.51E+08	T	C	P2RY14	exonic	ynonymou	277R,ENSG	452	128	580	0.22069	60	0	60	0	0.96	Clonal	0.6	3.02			disease_causing	26.6	p.K277R	Passenger	SENGER/OT	damaging	Deleterious	Damaging
OVA_047_M1	METASTASIS	chr3	1.51E+08	1.51E+08	T	A	MED12L	exonic	ynonymou	.015K,ENSG	516	157	673	0.233284	43	0	43	0	0.98	Sub	0.47	2.01			disease_causing	29.8	p.I1155K	Passenger	SENGER/OT	damaging	Deleterious	Damaging
OVA_047_M2	METASTASIS	chr3	1.51E+08	1.51E+08	T	A	MED12L	exonic	ynonymou	.015K,ENSG	482	210	692	0.303468	43	0	43	0	1	Sub	0.44	2.01			disease_causing	29.8	p.I1155K	Passenger	SENGER/OT	damaging	Deleterious	Damaging
OVA_047_M3	METASTASIS	chr3	1.51E+08	1.51E+08	T	A	MED12L	exonic	ynonymou	.015K,ENSG	434	85	519	0.163776	43	0	43	0	0.71	Sub	0.6	3.02			disease_causing	29.8	p.I1155K	Passenger	SENGER/OT	damaging	Deleterious	Damaging
OVA_365_M1	METASTASIS	chr2	1.52E+08	1.52E+08	T	C	NEB	exonic	ynonymou	857R,ENSG	301	247	548	0.45073	104	0	104	0	1	Clonal	0.85	1.87			disease_causing	22.1	p.Q3857R	Passenger		benign	Neutral	Tolerated
OVA_365_M2	METASTASIS	chr2	1.52E+08	1.52E+08	T	C	NEB	exonic	ynonymou	857R,ENSG	403	92	495	0.185859	104	0	104	0	0.92	Clonal	0.4	1.81			disease_causing	22.1	p.Q3857R	Passenger		benign	Neutral	Tolerated
OVA_365_M3	METASTASIS	chr2	1.52E+08	1.52E+08	T	C	NEB	exonic	ynonymou	857R,ENSG	337	242	579	0.417962	104	0	104	0	1	Clonal	0.82	1.89			disease_causing	22.1	p.Q3857R	Passenger		benign	Neutral	Tolerated
OVA_365_C1	Contralateral ovary	chr2	1.52E+08	1.52E+08	T	C	NEB	exonic	ynonymou	857R,ENSG	398	108	506	0.213439	104	0	104	0	1	Clonal	0.42	1.83			disease_causing	22.1	p.Q3857R	Passenger		benign	Neutral	Tolerated

Sample	Type	Chr	Start	End	Ref	Alt	Gene	unc.ensGen	icFunc.ens	hange.ens(	t_ref	t_alt	t_total	t_VAF	n_ref	n_alt	n_total	normal_VA	ncer_cell_f	on-based C	purity	ploidy	Cancer Gene	Driver Gene	Mutation Taster	ADD(PHRE	otein_chan	CHASM	ATHMM_Pr	OLYPHEN-	REDICTION	ICTION (cutoff=0.05)
OVA_365_C2	PRIMARY	chr2	1.52E+08	1.52E+08	T	C	NEB	exonic	ynonymous	3857R,ENSG	387	159	546	0.291209	104	0	104	0	0.94	Clonal	0.62	1.92			disease_causing	22.1	p.Q3857R	Passenger		benign	Neutral	Tolerated
OVA_047_M2	METASTASIS	chr4	1.53E+08	1.53E+08	T	C	PET112	exonic	ynonymous	000005123(	550	90	640	0.140625	44	0	44	0	0.49	Sub	0.44	2.01			disease_causing	0.437	p.I185V	Passenger	SENGER/OT	benign	Neutral	Tolerated
OVA_048_C1	PRIMARY	chr6	1.53E+08	1.53E+08	C	T	SYNE1	exonic	ynonymous	000423061:	156	81	237	0.341772	52	0	52	0	1	Clonal	0.67	1.82			disease_causing	15.3	p.A1952T	Passenger	SENGER/OT	benign	Neutral	Tolerated
OVA_048_C2	PRIMARY	chr6	1.53E+08	1.53E+08	C	T	SYNE1	exonic	ynonymous	000423061:	378	166	544	0.305147	52	0	52	0	1	Clonal	0.72	1.85			disease_causing	15.3	p.A1952T	Passenger	SENGER/OT	benign	Neutral	Tolerated
OVA_048_C3	PRIMARY	chr6	1.53E+08	1.53E+08	C	T	SYNE1	exonic	ynonymous	000423061:	194	122	316	0.386076	52	0	52	0	1	Clonal	0.74	1.8			disease_causing	15.3	p.A1952T	Passenger	SENGER/OT	benign	Neutral	Tolerated
OVA_047_C1	PRIMARY	chrX	1.53E+08	1.53E+08	G	A	SRPK3	intronic	NA	NA	162	26	188	0.138298	15	0	15	0	0.88	Sub	0.6	2.91			polymorphism	9.169						
OVA_047_C2	PRIMARY	chrX	1.53E+08	1.53E+08	G	A	SRPK3	intronic	NA	NA	145	25	170	0.147059	15	0	15	0	0.75	Sub	0.64	2.76			polymorphism	9.169						
OVA_047_C3	Contralateral ovary	chrX	1.53E+08	1.53E+08	G	A	SRPK3	intronic	NA	NA	126	18	144	0.125	15	0	15	0	0.79	Clonal	0.6	2.94			polymorphism	9.169						
OVA_047_C1	PRIMARY	chrX	1.53E+08	1.53E+08	T	A	SRPK3	intronic	NA	NA	259	68	327	0.207951	26	0	26	0	1	Clonal	0.6	2.91			polymorphism	0.432						
OVA_047_C2	PRIMARY	chrX	1.53E+08	1.53E+08	T	A	SRPK3	intronic	NA	NA	252	76	328	0.231707	26	0	26	0	1	Clonal	0.64	2.76			polymorphism	0.432						
OVA_047_C3	Contralateral ovary	chrX	1.53E+08	1.53E+08	T	A	SRPK3	intronic	NA	NA	210	46	256	0.179688	26	0	26	0	1	Clonal	0.6	2.94			polymorphism	0.432						
OVA_047_C1	PRIMARY	chrX	1.53E+08	1.53E+08	G	A	IDH3G	exonic	ynonymous	.08C,ENSG(	504	118	622	0.189711	67	1	68	0.014706	1	Clonal	0.6	2.91			disease_causing	23.6	p.R108C	Driver	SENGER/OT	damaging	Deleterious	Damaging
OVA_047_C2	PRIMARY	chrX	1.53E+08	1.53E+08	G	A	IDH3G	exonic	ynonymous	.08C,ENSG(	464	150	614	0.2443	67	1	68	0.014706	1	Clonal	0.64	2.76			disease_causing	23.6	p.R108C	Driver	SENGER/OT	damaging	Deleterious	Damaging
OVA_047_C3	Contralateral ovary	chrX	1.53E+08	1.53E+08	G	A	IDH3G	exonic	ynonymous	.08C,ENSG(	448	165	613	0.269168	67	1	68	0.014706	1	Clonal	0.6	2.94			disease_causing	23.6	p.R108C	Driver	SENGER/OT	damaging	Deleterious	Damaging
OVA_378_M1	METASTASIS	chrX	1.54E+08	1.54E+08	-	G	FLNA	exonic	neshift inse	0360319:ex	27	31	58	0.534483	163	0	163		0.95	Clonal	0.72	2.57	Yes	Yes	polymorphism		p.Q2546Hfs	Passenger				
OVA_378_M2	METASTASIS	chrX	1.54E+08	1.54E+08	-	G	FLNA	exonic	neshift inse	0360319:ex	9	29	38	0.763158	163	0	163		1	Clonal	0.85	2.57	Yes	Yes	polymorphism		p.Q2546Hfs	Passenger				
OVA_378_M3	METASTASIS	chrX	1.54E+08	1.54E+08	-	G	FLNA	exonic	neshift inse	0360319:ex	32	55	87	0.632184	163	0	163		1	Clonal	0.67	2.57	Yes	Yes	polymorphism		p.Q2546Hfs	Passenger				
OVA_378_C2	PRIMARY	chrX	1.54E+08	1.54E+08	C	G	FLNA	exonic	ynonymous	1692L,ENSG	335	205	540	0.37963	162	0	162	0	0.97	Clonal	0.56	2.59	Yes	Yes	disease_causing	25.5	p.V1700L	Passenger		damaging	Deleterious	Damaging
OVA_048_M1	METASTASIS	chr1	1.54E+08	1.54E+08	C	T	NPR1	exonic	nonymous S	000368680	589	8	597	0.0134	71	0	71	0	0.09	Sub	0.56	3.94			disease_causing	11.71		Passenger			Neutral	Tolerated
OVA_048_M2	METASTASIS	chr1	1.54E+08	1.54E+08	C	T	NPR1	exonic	nonymous S	000368680	564	18	582	0.030928	71	0	71	0	0.25	Sub	0.4	3.87			disease_causing	11.71		Passenger			Neutral	Tolerated
OVA_048_M3	METASTASIS	chr1	1.54E+08	1.54E+08	C	T	NPR1	exonic	nonymous S	000368680	471	106	577	0.183709	71	0	71	0	1	Sub	0.59	3.39			disease_causing	11.71		Passenger			Neutral	Tolerated
OVA_048_M1	METASTASIS	chr4	1.54E+08	1.54E+08	G	C	FHDC1	exonic	ynonymous	S879T,ENSG	21	4	25	0.16	26	0	26	0	0.57	Clonal	0.56	3.94			polymorphism	5.833	p.S879T	Passenger	SENGER/OT	benign	Neutral	Tolerated
OVA_365_C2	PRIMARY	chr7	1.54E+08	1.54E+08	G	A	DPP6	exonic	nonymous S	.S10S,ENSG	174	66	240	0.275	101	0	101	0	1	Clonal	0.62	1.92			disease_causing	14.07		Passenger			Neutral	Tolerated
OVA_365_M1	METASTASIS	chr1	1.54E+08	1.54E+08	G	-	UBAP2L	exonic	neshift dele	530fs,ENSG	388	281	669	0.42003	53	1	54		0.99	Clonal	0.85	1.87					p.A520Hfs	Passenger			NA	NA
OVA_365_M2	METASTASIS	chr1	1.54E+08	1.54E+08	G	-	UBAP2L	exonic	neshift dele	530fs,ENSG	394	100	494	0.202429	53	1	54		1	Clonal	0.4	1.81					p.A520Hfs	Passenger			NA	NA
OVA_365_M3	METASTASIS	chr1	1.54E+08	1.54E+08	G	-	UBAP2L	exonic	neshift dele	530fs,ENSG	277	230	507	0.453649	53	1	54		1	Clonal	0.82	1.89					p.A520Hfs	Passenger			NA	NA
OVA_365_C1	Contralateral ovary	chr1	1.54E+08	1.54E+08	G	-	UBAP2L	exonic	neshift dele	530fs,ENSG	328	108	436	0.247706	53	1	54		1	Clonal	0.42	1.83					p.A520Hfs	Passenger			NA	NA
OVA_365_C2	PRIMARY	chr1	1.54E+08	1.54E+08	G	-	UBAP2L	exonic	neshift dele	530fs,ENSG	385	156	541	0.288355	53	1	54		0.94	Clonal	0.62	1.92					p.A520Hfs	Passenger			NA	NA
OVA_047_C3	Contralateral ovary	chr2	1.55E+08	1.55E+08	A	C	GALNT13	exonic	nonymous S	P115P,ENSG	604	72	676	0.106509	55	0	55	0	0.57	Sub	0.6	2.94			disease_causing	11.48		Passenger			Neutral	Tolerated
OVA_378_M1	METASTASIS	chr1	1.55E+08	1.55E+08	G	A	CLK2	intronic	NA	NA	402	111	513	0.216374	31	0	31	0	0.82	Sub	0.72	2.57	Yes		polymorphism	6.918						
OVA_378_M2	METASTASIS	chr1	1.55E+08	1.55E+08	G	A	CLK2	intronic	NA	NA	418	117	535	0.218692	31	0	31	0	0.73	Sub	0.85	2.57	Yes		polymorphism	6.918						
OVA_378_M3	METASTASIS	chr1	1.55E+08	1.55E+08	G	A	CLK2	intronic	NA	NA	455	145	600	0.241667	31	0	31	0	0.97	Sub	0.67	2.57	Yes		polymorphism	6.918						
OVA_378_C1	PRIMARY	chr1	1.55E+08	1.55E+08	G	A	CLK2	intronic	NA	NA	450	135	585	0.230769	31	0	31	0	1	Sub	0.59	2.6	Yes		polymorphism	6.918						
OVA_378_C2	PRIMARY	chr1	1.55E+08	1.55E+08	G	A	CLK2	intronic	NA	NA	449	107	556	0.192446	31	0	31	0	0.87	Sub	0.56	2.59	Yes		polymorphism	6.918						
OVA_378_C3	PRIMARY	chr1	1.55E+08	1.55E+08	G	A	CLK2	intronic	NA	NA	461	80	541	0.147874	31	0	31	0	0.73	Sub	0.51	2.6	Yes		polymorphism	6.918						
OVA_365_M1	METASTASIS	chr1	1.55E+08	1.55E+08	G	C	PKLR	exonic	ynonymous	F191L,ENSG	104	129	233	0.553648	82	0	82	0	1	Clonal	0.85	1.87			disease_causing	14.87	p.F191L	Passenger	SENGER/OT	benign	Deleterious	Damaging
OVA_365_M2	METASTASIS	chr1	1.55E+08	1.55E+08	G	C	PKLR	exonic	ynonymous	F191L,ENSG	52	20	72	0.277778	82	0	82	0	1	Clonal	0.4	1.81			disease_causing	14.87	p.F191L	Passenger	SENGER/OT	benign	Deleterious	Damaging
OVA_365_M3	METASTASIS	chr1	1.55E+08	1.55E+08	G	C	PKLR	exonic	ynonymous	F191L,ENSG	67	83	150	0.553333	82	0	82	0	1	Clonal	0.82	1.89			disease_causing	14.87	p.F191L	Passenger	SENGER/OT	benign	Deleterious	Damaging
OVA_365_C1	Contralateral ovary	chr1	1.55E+08	1.55E+08	G	C	PKLR	exonic	ynonymous	F191L,ENSG	67	39	106	0.367925	82	0	82	0	1	Clonal	0.42	1.83			disease_causing	14.87	p.F191L	Passenger	SENGER/OT	benign	Deleterious	Damaging
OVA_365_C2	PRIMARY	chr1	1.55E+08	1.55E+08	G	C	PKLR	exonic	ynonymous	F191L,ENSG	92	74	166	0.445783	82	0	82	0	1	Clonal	0.62	1.92			disease_causing	14.87	p.F191L	Passenger	SENGER/OT	benign	Deleterious	Damaging
OVA_378_C1	PRIMARY	chr4	1.55E+08	1.55E+08	G	C	DCHS2	exonic	ynonymous	000044350	31	5	36	0.138889	71	0	71	0	0.47	Sub	0.59	2.6			polymorphism	13.33	p.F330L	Passenger	SENGER/OT	benign	Neutral	Tolerated
OVA_378_C2	PRIMARY	chr4	1.55E+08	1.55E+08	G	C	DCHS2	exonic	ynonymous	000044350	21	6	27	0.222222	71	0	71	0	0.79	Sub	0.56	2.59			polymorphism	13.33	p.F330L	Passenger	SENGER/OT	benign	Neutral	Tolerated
OVA_378_C3	PRIMARY	chr4	1.55E+08	1.55E+08	G	C	DCHS2	exonic	ynonymous	000044350	17	3	20	0.15	71	0	71	0	0.59	Sub	0.51	2.6			polymorphism	13.33	p.F330L	Passenger	SENGER/OT	benign	Neutral	Tolerated
OVA_048_M1	METASTASIS	chr4	1.55E+08	1.55E+08	G	T	FGB	UTR5	NA	NA	117	170	287	0.592334	44	1	45	0.022222	1	Clonal	0.56	3.94			polymorphism	12.47						
OVA_048_M2	METASTASIS	chr4	1.55E+08	1.55E+08	G	T	FGB	UTR5	NA	NA	178	128	306	0.418301	44	1	45	0.022222	1	Clonal	0.4	3.87			polymorphism	12.47						
OVA_048_M3	METASTASIS	chr4	1.55E+08	1.55E+08	G	T	FGB	UTR5	NA	NA	122	182	304	0.598684	44	1	45	0.022222	1	Clonal	0.59	3.39			polymorphism	12.47						
OVA_048_C1	PRIMARY	chr4	1.55E+08	1.55E+08	G	T	FGB	UTR5	NA	NA	83	95	178	0.533708	44	1	45	0.022222	1	Clonal	0.67	1.82			polymorphism	12.47						
OVA_048_C2	PRIMARY	chr4	1.55E+08	1.55E+08	G	T	FGB	UTR5	NA	NA	121	156	277	0.563177	44	1	45	0.022222	1	Clonal	0.72	1.85			polymorphism	12.47						
OVA_048_C3	PRIMARY	chr4	1.55E+08	1.55E+08	G	T	FGB	UTR5	NA	NA	50	93	143	0.65035	44	1	45	0.022222	1	Clonal	0.74	1.8			polymorphism	12.47						
OVA_365_M1	METASTASIS	chr1	1.57E+08	1.57E+08	A	G	SH2D2A	intronic	NA	NA	26	15	41	0.365854	26	0	26	0	0.86	Clonal	0.85	1.87			polymorphism	3.451						
OVA_365_M2	METASTASIS	chr1	1.57E+08	1.57E+08	A	G	SH2D2A	intronic	NA	NA	18	6	24	0.25	26	0	26	0	1	Clonal	0.4	1.81			polymorphism	3.451						
OVA_365_M3	METASTASIS	chr1	1.57E+08	1.57E+08	A	G	SH2D2A	intronic	NA	NA	14	14	28	0.5	26	0	26	0	1	Clonal	0.82	1.89			polymorphism	3.451						
OVA_365_C1	Contralateral ovary	chr1	1.57E+08	1.57E+08	A	G	SH2D2A	intronic	NA	NA	21	8	29	0.275862	26	0	26	0	1	Clonal	0.42	1.83			polymorphism	3.451						
OVA_365_C2	PRIMARY	chr1	1.57E+08	1.57E+08	A	G	SH2D2A	intronic	NA	NA	30	12	42	0.285714	26	0	26	0	0.93	Clonal	0.62	1.92			polymorphism	3.451						
OVA_047_M1	METASTASIS	chr7	1.57E+08	1.57E+08	G	T	MNX1	exonic	nonymous S	000002529	30	9	39	0.230769	14	0	14	0	1	Clonal	0.47	2.01	Yes		polymorphism	14.48		Passenger			Neutral	Tolerated
OVA_047_M2	METASTASIS	chr7	1.57E+08	1.57E+08	G	T	MNX1	exonic	nonymous S	000002529	31	10	41	0.243902	14	0	14	0	1	Clonal	0.44	2.01	Yes		polymorphism	14.48		Passenger			Neutral	Tolerated
OVA_047_M3	METASTASIS	chr7	1.57E+08	1.57E+08	G	T	MNX1	exonic	nonymous S	000002529	32	10	42	0.238095	14	0	14	0	1	Clonal	0.6	3.02	Yes		polymorphism	14.48		Passenger			Neutral	Tolerated
OVA_048_M1	METASTASIS	chr1	1.57E+08	1.57E+08	A	G	NTRK1	exonic	ynonymous	000005243	139	164	303	0.541254	52	1	53	0.018868	1	Clonal	0.56	3.94	Yes	Yes	disease_causing	23.8	p.N121S	Passenger	SENGER/OT	damaging	Deleterious	Damaging
OVA_048_M2	METASTASIS	chr1	1.57E+08	1.57E+08	A	G	NTRK1	exonic	ynonymous	000005243	203	124	327	0.379205	52	1	53	0.018868	1	Clonal	0.4	3.87	Yes	Yes	disease_causing	23.8	p.N121S	Passenger	SENGER/OT	damaging	Deleterious	Damaging
OVA_048_M3	METASTASIS	chr1	1.57E+08	1.57E+08	A	G	NTRK1	exonic	ynonymous	000005243	155	106	261	0.40613	52	1	53	0.018868	1	Clonal	0.59	3.39	Yes	Yes	disease_causing	23.8	p.N121S	Passenger	SENGER/OT	damaging	Deleterious	Damaging
OVA_365_M1	METASTASIS	chr5	1.57E+08	1.57E+08	G	A	NIPAL4	exonic	stopgain	W13X,ENSG	3	11	14	0.785714	29	0	29	0	1	Clonal	0.85	1.87			disease_causing_automat	35	p.W13X	Passenger			NA	NA
OVA_365_M3	METASTASIS	chr5	1.57E+08	1.57E+08	G	A	NIPAL4	exonic	stopgain	W13X,ENSG	1	8	9	0.888889	29	0	29	0	1	Clonal	0.82	1.89			disease_causing_automat	35	p.W13X	Passenger			NA	NA
OVA_365_C1	Contralateral ovary	chr5	1.57E+08	1.57E+08	G	A	NIPAL4	exonic	stopgain	W13X,ENSG	5	4	9	0.444444	29	0	29	0	1	Clonal	0.42	1.83			disease_causing_automat	35	p.W13X	Passenger			NA	NA
OVA_365_C2	PRIMARY	chr5	1.57E+08	1.57E+08	G	A	NIPAL4	exonic	stopgain	W13X,ENSG	1	6	7	0.857143	29	0	29	0	1	Clonal	0.62	1.92			disease_causing_automat	35	p.W13X	Passenger			NA	NA
OVA_003_M1	METASTASIS	chr1	1.57E+08	1.57E+08	C	T	LRRC71	intronic	NA	NA	95	30	125	0.24	59	0	59	0	0.85	Sub	0.57	1.84			polymorphism	1.701						
OVA_003_M2	METASTASIS	chr1	1.57E+08	1.57E+08	C	T	LRRC71	intronic	NA	NA	180	34	214	0.158879	59	0	59	0	0.69	Sub	0.46	1.81			polymorphism	1.701						
OVA_003_M3	METASTASIS	chr1	1.57E+08	1.57E+08	C	T	LRRC71	intronic	NA	NA	167	15	182	0.082418	59	0	59	0	0.87	Sub	0.34	2.67			polymorphism	1.701						
OVA_003_C1	PRIMARY	chr1	1.57E+08	1.57E+08	C	T	LRRC71	intronic	NA	NA	149	87	236	0.368644	59	0	59	0	0.85	Sub	0.86	1.81			polymorphism	1.701						
OVA_003_C2	PRIMARY	chr1	1.57E+08	1.57E+08	C	T	LRRC71	intronic	NA	NA	160	68	228	0.298246	59	0	59	0	1	Sub	0.7	3.61			polymorphism	1.701						
OVA_003_C3	PRIMARY	chr1	1.57E+08	1.57E+08	C	T	LRRC71	intronic	NA	NA	155	103	258	0.399225	59	0	59	0	0.97	Sub	0.82	1.81			polymorphism	1.701						
OVA_047_C3	Contralateral ovary	chr1	1.57E+08	1.57E+08	A	C	ARHGEF11	exonic	ynonymous	00361409:	539	80	619	0.129241	47	1	48	0.020833	0.95	Clonal	0.6	2.94			disease_causing	13.23	p.V1283G	Passenger	SENGER/OT	benign	Neutral	Tolerated
OVA_047_C1	PRIMARY	chr2	1.57E+08	1.57E+08	G	A	GPD2	exonic	ynonymous	00409674:	595	117	712	0.164326	71	0	71	0	0.88	Clonal	0.6	2.91			disease_causing	23.6	p.G392E	Passenger	SENGER/OT	benign	Deleterious	Tolerated
OVA_047_C2	PRIMARY	chr2	1.57E+08	1.57E+08	G	A	GPD2	exonic	ynonymous	00409674:	592	126	718	0.175487	71	0	71	0	0.9	Clonal	0.64	2.76			disease_causing	23.6	p.G392E	Passenger	SENGER/OT	benign	Deleterious	Tolerated
OVA_047_C3	Contralateral ovary	chr2	1.57E+08	1.57E+08	G	A	GPD2	exonic	ynonymous	00409674:	545	144	689	0.208999	71	0	71	0	1	Clonal	0.6	2.94			disease_causing	23.6	p.G392E	Passenger	SENGER/OT	benign	Deleterious	Tolerated
OVA_003_C1	PRIMARY	chr6	1.58E+08	1.58E+08	C	G	SNX9	cRNA_exon	NA	NA	529	94	623	0.150883	44	0	44	0	0.35	Sub	0.86	1.81			polymorphism	4.415						
OVA_048_M1	METASTASIS	chr6	1.59E+08	1.59E+08	T	G	RSPH3	intronic	NA	NA	161	104	265	0.392453	20	0	20	0	1	Clonal	0.56	3.94			polymorphism	5.878						
OVA_048_M2	METASTASIS	chr6	1.59E+08	1.59E+08	T	G	RSPH3	intronic	NA	NA	201	108	309	0.349515	20	0	20	0	1	Clonal	0.4	3.87			polymorphism	5.878						
OVA_048_M3	METASTASIS	chr6	1.59E+08	1.59E+08	T	G	RSPH3	intronic	NA	NA	163	139	302	0.460265	20	0	20	0	1	Clonal	0.59	3.39			polymorphism	5.878						
OVA_365_M1	METASTASIS	chr4	1.6E+08	1.6E+08	C	T	PPID	exonic	ynonymous	000030772	328	265	593	0.44688	37	0	37	0	1	Clonal	0.85	1.87			polymorphism	19.08	p.G190R	Passenger	SENGER/OT	benign	Neutral	Tolerated
OVA_365_M2	METASTASIS	chr4	1.6E+08	1.6E+08	C	T	PPID	exonic	ynonymous	000030772	389	88	477	0.184486	37	0	37	0	0.92	Clonal	0.4	1.81			polymorphism	19.08	p.G190R	Passenger	SENGER/OT	benign	Neutral	Tolerated
OVA_365_M3	METASTASIS	chr4	1.6E+08	1.6E+08	C	T	PPID	exonic	ynonymous	000030772	331	239	570	0.419298	37	0	37	0	1	Clonal	0.82	1.89			polymorphism	19.08	p.G190R	Passenger	SENGER/OT	benign	Neutral	Tolerated
OVA_365_C1	Contralateral ovary	chr4	1.6E+08	1.6E+08	C	T	PPID	exonic	ynonymous	000030772	358	114	472	0.241525	37	0	37	0	0.92	Clonal	0.42	1.83			polymorphism	19.08	p.G190R	Passenger	SENGER/OT	benign	Neutral	Tolerated
OVA_365_C2	PRIMARY	chr4	1.6E+08	1.6E+08	C	T	PPID	exonic	ynonymous	000030772	381	149	530	0.281132	37	0	37	0	0.91	Clonal	0.62	1.92			polymorphism	19.08	p.G190R	Passenger	SENGER/OT	benign	Neutral	Tolerated
OVA_365_M3	METASTASIS	chr3	1.6E+08	1.6E+08	C	T	SMC4	exonic	ynonymous	000004659	432	39	471	0.082803	54	0	54	0	0.2	Sub	0.82	1.89			disease_causing	22.8	p.P66S	Passenger	SENGER/OT	benign	Deleterious	Tolerated
OVA_378_C3	PRIMARY	chr6	1.6E+08	1.6E+08	G	C	PNLDC1	intronic	NA	NA	357	31	388	0.079897	73	0	73	0	0.31	Sub	0.51	2.6			disease_causing	21.8						
OVA_003_M1	METASTASIS	chr1	1.6E+08	1.6E+08	C	G	COPA	intronic	NA	NA	411	152	563	0.269982	58	0	58	0	0.96	Sub	0.57	1.84			polymorphism	1.701						
OVA_003_M2	METASTASIS	chr1	1.6E+08	1.6E+08	C	G	COPA	intronic	NA	NA	468	119	587	0.202726	58	0	58	0	0.88	Sub	0.46	1.81			polymorphism	1.701						
OVA_003_M3	METASTASIS	chr1	1.6E+08	1.6E+08	C	G	COPA	intronic	NA	NA	607	32	639	0.050078	58	0	58	0	0.53	Sub	0.34	2.67			polymorphism	1.701						
OVA_003_C1	PRIMARY	chr1	1.6E+08	1.6E+08	C	G	COPA	intronic	NA	NA	364	262	626	0.41853	58	0	58	0	0.97	Clonal	0.86	1.81			polymorphism	1.701						
OVA_003_C2	PRIMARY	chr1	1.6E+08	1.6E+08	C	G	COPA	intronic	NA	NA	363	247	610	0.404918	58	0	58	0	1	Clonal	0.7	3.61			polymorphism	1.701						
OVA_003_C3	PRIMARY	chr1	1.6E+08	1.6E+08	C	G	COPA	intronic	NA	NA	403	237	640	0.370313	58	0	58	0	0.9	Clonal	0.82	1.81			polymorphism	1.701						
OVA_047_M1	METASTASIS	chr6	1.6E+08	1.6E+08	C	T	IGF2R	exonic	ynonymous	000356956	429	229	658	0.348024	90	0	90	0	1	Clonal	0.47	2.01			disease_causing	27.5	p.T1470I	Passenger	SENGER/OT	damaging	Deleterious	Damaging
OVA_047_M2	METASTASIS	chr6	1.6E+08	1.6E+08	C	T	IGF2R	exonic	ynonymous	000356956	483	185	668	0.276946	90	0	90	0	0.97	Clonal	0.44	2.01			disease_causing	27.5	p.T1470I	Passenger	SENGER/OT	damaging	Deleterious	Damaging
OVA_047_M3	METASTASIS	chr6	1.6E+08	1.6E+08	C	T	IGF2R	exonic	ynonymous	000356956	278	124	402	0.308458	90	0	90	0	1	Clonal	0.6	3.02			disease_causing	27.5	p.T1470I	Passenger	SENGER/OT	damaging	Deleterious	Damaging
OVA_047_C1	PRIMARY	chr1	1.61E+08	1.61E+08	G	C	CD84	:RNA_intro	NA	NA	651	68	719	0.094576	35	0	35	0	0.69	Sub	0.6	2.91			disease_causing	14.73						
OVA_047_C2	PRIMARY	chr1	1.61E+08	1.61E+08	G	C	CD84	:RNA_intro	NA	NA	641	80	721	0.110957	35	0	35	0	0.79	Sub	0.64	2.76			disease_causing	14.73						
OVA_047_C3	Contralateral ovary	chr1	1.61E+08	1.61E+08	G	C	CD84	:RNA_intro	NA	NA	641	74	715	0.103497	35	0	35	0	0.76	Sub	0.6	2.94			disease_causing	14.73						
OVA_365_C2	PRIMARY	chr2	1.61E+08	1.61E+08	T	C	75;LY75-CD	intronic	NA	NA	431	170	601	0.282862	52	0	52	0	0.92	Clonal	0.62	1.92			polymorphism	9.042						
OVA_047_C1	PRIMARY	chr1	1.61E+08	1.61E+08	G	A	CD244	exonic	ynonymous	000036803	674	89	763	0.116645	42	0	42	0	0.86	Sub	0.6	2.91			polymorphism	0.01	p.T46M	Passenger	SENGER/OT	benign	Neutral	Tolerated
OVA_047_C2	PRIMARY	chr1	1.61E+08	1.61E+08	G	A	CD244	exonic	ynonymous	000036803	679	90	769	0.117035	42	0	42	0	0.83	Sub	0.64	2.76			polymorphism	0.01	p.T46M	Passenger	SENGER/OT	benign	Neutral	Tolerated

Sample	Type	Chr	Start	End	Ref	Alt	Gene	unc.ensGen	icFunc.ens	hange.ens	t_ref	t_alt	t_total	t_VAF	n_ref	n_alt	n_total	normal_VA	ncer_cell_f	on-based C	purity	ploidy	Cancer Gene	Driver Gene	Mutation Taster	ADD(PHRE	otein_chan	CHASM	ATHMM_Pr	OLYPHEN-	REDICTION	ICTION (cutoff=0.05)
OVA_047_C3	Contralateral ovary	chr1	1.61E+08	1.61E+08	G	A	CD244	exonic	ynonymous	000036803	663	104	767	0.135593	42	0	42	0	1	Clonal	0.6	2.94			polymorphism	0.01	p.T46M	Passenger	SENGER/OT	benign	Neutral	Tolerated
OVA_013_M1	METASTASIS	chr1	1.61E+08	1.61E+08	A	T	ITLN2	intronic	NA	NA	477	112	589	0.190153	21	0	21	0	0.95	Clonal	0.67	3.06			polymorphism	2.009						
OVA_047_M2	METASTASIS	chr6	1.61E+08	1.61E+08	G	T	LPA	intronic	NA	NA	535	94	629	0.149444	31	0	31	0	0.52	Sub	0.44	2.01			polymorphism	0.096						
OVA_047_M2	METASTASIS	chr6	1.61E+08	1.61E+08	T	C	LPA	intronic	NA	NA	538	90	628	0.143312	31	0	31	0	0.5	Sub	0.44	2.01			polymorphism	1.08						
OVA_013_C1	PRIMARY	chr5	1.61E+08	1.61E+08	A	T	GABRA1	intronic	NA	NA	445	84	529	0.15879	55	0	55	0	0.44	Sub	0.72	3.1			polymorphism	4.283						
OVA_048_M1	METASTASIS	chr4	1.62E+08	1.62E+08	CA	GT	FSTL5	exonic	nonymous S	345V,ENSG	54	73	127	0.574803	22	0	22	0	1	Clonal	0.56	3.94			disease_causing	9.388		Passenger			Neutral	Tolerated
OVA_048_M2	METASTASIS	chr4	1.62E+08	1.62E+08	CA	GT	FSTL5	exonic	nonymous S	345V,ENSG	78	39	117	0.333333	22	0	22	0	1	Clonal	0.4	3.87			disease_causing	9.388		Passenger			Neutral	Tolerated
OVA_048_M3	METASTASIS	chr4	1.62E+08	1.62E+08	CA	GT	FSTL5	exonic	nonymous S	345V,ENSG	39	74	113	0.654867	22	0	22	0	1	Clonal	0.59	3.39			disease_causing	9.388		Passenger			Neutral	Tolerated
OVA_048_C1	PRIMARY	chr4	1.62E+08	1.62E+08	CA	GT	FSTL5	exonic	nonymous S	345V,ENSG	25	23	48	0.479167	22	0	22	0	0.95	Clonal	0.67	1.82			disease_causing	9.388		Passenger			Neutral	Tolerated
OVA_048_C2	PRIMARY	chr4	1.62E+08	1.62E+08	CA	GT	FSTL5	exonic	nonymous S	345V,ENSG	45	54	99	0.545455	22	0	22	0	0.97	Clonal	0.72	1.85			disease_causing	9.388		Passenger			Neutral	Tolerated
OVA_048_C3	PRIMARY	chr4	1.62E+08	1.62E+08	CA	GT	FSTL5	exonic	nonymous S	345V,ENSG	30	39	69	0.565217	22	0	22	0	0.96	Clonal	0.74	1.8			disease_causing	9.388		Passenger			Neutral	Tolerated
OVA_378_C1	PRIMARY	chr2	1.63E+08	1.63E+08	A	T	DPP4	splicing	NA	NA	568	92	660	0.139394	90	0	90	0	0.75	Sub	0.59	2.6			disease_causing	34		Passenger				
OVA_378_C2	PRIMARY	chr2	1.63E+08	1.63E+08	A	T	DPP4	splicing	NA	NA	499	104	603	0.172471	90	0	90	0	0.78	Sub	0.56	2.59			disease_causing	34		Passenger				
OVA_378_C3	PRIMARY	chr2	1.63E+08	1.63E+08	A	T	DPP4	splicing	NA	NA	508	104	612	0.169935	90	0	90	0	0.84	Sub	0.51	2.6			disease_causing	34		Passenger				
OVA_048_C2	PRIMARY	chr4	1.64E+08	1.64E+08	C	T	TMA16	intronic	NA	NA	210	299	509	0.587426	42	0	42	0	1	Clonal	0.72	1.85			polymorphism	2.243						
OVA_013_M1	METASTASIS	chr3	1.65E+08	1.65E+08	C	A	SI	exonic	ynonymous	000264382	452	162	614	0.263844	62	0	62	0	1	Clonal	0.67	3.06			disease_causing	23.6	p.A826S	Passenger	SENGER/OT	damaging	Deleterious	Damaging
OVA_013_C1	PRIMARY	chr3	1.65E+08	1.65E+08	C	A	SI	exonic	ynonymous	000264382	428	170	598	0.284281	62	0	62	0	1	Clonal	0.72	3.1			disease_causing	23.6	p.A826S	Passenger	SENGER/OT	damaging	Deleterious	Damaging
OVA_013_C2	PRIMARY	chr3	1.65E+08	1.65E+08	C	A	SI	exonic	ynonymous	000264382	395	172	567	0.303351	62	0	62	0	1	Clonal	0.87	3.05			disease_causing	23.6	p.A826S	Passenger	SENGER/OT	damaging	Deleterious	Damaging
OVA_013_C3	PRIMARY	chr3	1.65E+08	1.65E+08	C	A	SI	exonic	ynonymous	000264382	430	185	615	0.300813	62	0	62	0	0.96	Clonal	0.91	3.01			disease_causing	23.6	p.A826S	Passenger	SENGER/OT	damaging	Deleterious	Damaging
OVA_003_M1	METASTASIS	chr1	1.67E+08	1.67E+08	G	A	FMO9P	cRNA_exon	NA	NA	361	139	500	0.278	34	0	34	0	0.98	Sub	0.57	1.84				15.34						
OVA_003_M2	METASTASIS	chr1	1.67E+08	1.67E+08	G	A	FMO9P	cRNA_exon	NA	NA	455	108	563	0.191829	34	0	34	0	0.84	Sub	0.46	1.81				15.34						
OVA_003_M3	METASTASIS	chr1	1.67E+08	1.67E+08	G	A	FMO9P	cRNA_exon	NA	NA	536	29	565	0.051327	34	0	34	0	0.54	Sub	0.34	2.67				15.34						
OVA_003_C1	PRIMARY	chr1	1.67E+08	1.67E+08	G	A	FMO9P	cRNA_exon	NA	NA	298	243	541	0.449168	34	0	34	0	1	Clonal	0.86	1.81				15.34						
OVA_003_C2	PRIMARY	chr1	1.67E+08	1.67E+08	G	A	FMO9P	cRNA_exon	NA	NA	319	241	560	0.430357	34	0	34	0	1	Clonal	0.7	3.61				15.34						
OVA_003_C3	PRIMARY	chr1	1.67E+08	1.67E+08	G	A	FMO9P	cRNA_exon	NA	NA	313	256	569	0.449912	34	0	34	0	1	Clonal	0.82	1.81				15.34						
OVA_365_M2	METASTASIS	chr5	1.68E+08	1.68E+08	A	C	TENM2	exonic	nonymous S	00519204:e	130	66	196	0.336735	29	0	29	0	1	Sub	0.4	1.81			disease_causing	11.15		Passenger			Neutral	Tolerated
OVA_365_M3	METASTASIS	chr5	1.68E+08	1.68E+08	A	C	TENM2	exonic	nonymous S	00519204:e	345	34	379	0.08971	29	0	29	0	0.22	Sub	0.82	1.89			disease_causing	11.15		Passenger			Neutral	Tolerated
OVA_003_M1	METASTASIS	chr6	1.68E+08	1.68E+08	C	T	MLLT4	exonic	stopgain	000400822	149	172	321	0.535826	84	1	85	0.011765	1	Clonal	0.57	1.84	Yes	No	isease_causing_automat	43	p.Q1415X	Passenger			NA	NA
OVA_003_M2	METASTASIS	chr6	1.68E+08	1.68E+08	C	T	MLLT4	exonic	stopgain	000400822	239	199	438	0.454338	84	1	85	0.011765	1	Clonal	0.46	1.81	Yes	No	isease_causing_automat	43	p.Q1415X	Passenger			NA	NA
OVA_003_M3	METASTASIS	chr6	1.68E+08	1.68E+08	C	T	MLLT4	exonic	stopgain	000400822	343	49	392	0.125	84	1	85	0.011765	1	Clonal	0.34	2.67	Yes	No	isease_causing_automat	43	p.Q1415X	Passenger			NA	NA
OVA_003_C1	PRIMARY	chr6	1.68E+08	1.68E+08	C	T	MLLT4	exonic	stopgain	000400822	51	323	374	0.863636	84	1	85	0.011765	1	Clonal	0.86	1.81	Yes	No	isease_causing_automat	43	p.Q1415X	Passenger			NA	NA
OVA_003_C2	PRIMARY	chr6	1.68E+08	1.68E+08	C	T	MLLT4	exonic	stopgain	000400822	81	316	397	0.79597	84	1	85	0.011765	1	Clonal	0.7	3.61	Yes	No	isease_causing_automat	43	p.Q1415X	Passenger			NA	NA
OVA_003_C3	PRIMARY	chr6	1.68E+08	1.68E+08	C	T	MLLT4	exonic	stopgain	000400822	72	415	487	0.852156	84	1	85	0.011765	1	Clonal	0.82	1.81	Yes	No	isease_causing_automat	43	p.Q1415X	Passenger			NA	NA
OVA_048_C3	PRIMARY	chr6	1.68E+08	1.68E+08	G	A	MLLT4	exonic	ynonymous	000344191:	162	89	251	0.354582	56	0	56	0	0.96	Clonal	0.74	1.8	Yes	No	disease_causing	24.7	p.A1656T	Passenger		damaging	Neutral	Damaging
OVA_047_C1	PRIMARY	chr4	1.69E+08	1.69E+08	A	G	ANXA10	UTR3	NA	NA	356	132	488	0.270492	67	0	67	0	0.9	Sub	0.6	2.91			polymorphism	4.043						
OVA_047_C2	PRIMARY	chr4	1.69E+08	1.69E+08	A	G	ANXA10	UTR3	NA	NA	390	153	543	0.281768	67	0	67	0	0.88	Sub	0.64	2.76			polymorphism	4.043						
OVA_047_C3	Contralateral ovary	chr4	1.69E+08	1.69E+08	A	G	ANXA10	UTR3	NA	NA	309	140	449	0.311804	67	0	67	0	1	Clonal	0.6	2.94			polymorphism	4.043						
OVA_378_M1	METASTASIS	chr4	1.69E+08	1.69E+08	A	C	DDX60L	exonic	ynonymous	.404R,ENSG	164	130	294	0.442177	24	0	24	0	1	Clonal	0.72	2.57			polymorphism	23.2	p.L1404R	Passenger	SENGER/OT	damaging	Deleterious	Damaging
OVA_378_M2	METASTASIS	chr4	1.69E+08	1.69E+08	A	C	DDX60L	exonic	ynonymous	.404R,ENSG	126	127	253	0.501976	24	0	24	0	1	Clonal	0.85	2.57			polymorphism	23.2	p.L1404R	Passenger	SENGER/OT	damaging	Deleterious	Damaging
OVA_378_M3	METASTASIS	chr4	1.69E+08	1.69E+08	A	C	DDX60L	exonic	ynonymous	.404R,ENSG	303	191	494	0.38664	24	0	24	0	1	Clonal	0.67	2.57			polymorphism	23.2	p.L1404R	Passenger	SENGER/OT	damaging	Deleterious	Damaging
OVA_378_C1	PRIMARY	chr4	1.69E+08	1.69E+08	A	C	DDX60L	exonic	ynonymous	.404R,ENSG	235	122	357	0.341737	24	0	24	0	1	Clonal	0.59	2.6			polymorphism	23.2	p.L1404R	Passenger	SENGER/OT	damaging	Deleterious	Damaging
OVA_378_C2	PRIMARY	chr4	1.69E+08	1.69E+08	A	C	DDX60L	exonic	ynonymous	.404R,ENSG	249	94	343	0.274052	24	0	24	0	0.97	Clonal	0.56	2.59			polymorphism	23.2	p.L1404R	Passenger	SENGER/OT	damaging	Deleterious	Damaging
OVA_378_C3	PRIMARY	chr4	1.69E+08	1.69E+08	A	C	DDX60L	exonic	ynonymous	.404R,ENSG	219	85	304	0.279605	24	0	24	0	1	Clonal	0.51	2.6			polymorphism	23.2	p.L1404R	Passenger	SENGER/OT	damaging	Deleterious	Damaging
OVA_378_M1	METASTASIS	chr3	1.7E+08	1.7E+08	A	T	SAMD7	exonic	ynonymous	396N,ENSG	510	82	592	0.138514	38	0	38	0	0.8	Sub	0.72	2.57			polymorphism	22.4	p.K396N	Passenger	SENGER/OT	damaging	Neutral	Tolerated
OVA_378_M2	METASTASIS	chr3	1.7E+08	1.7E+08	A	T	SAMD7	exonic	ynonymous	396N,ENSG	483	105	588	0.178571	38	0	38	0	0.95	Sub	0.85	2.57			polymorphism	22.4	p.K396N	Passenger	SENGER/OT	damaging	Neutral	Tolerated
OVA_378_M3	METASTASIS	chr3	1.7E+08	1.7E+08	A	T	SAMD7	exonic	ynonymous	396N,ENSG	574	81	655	0.123664	38	0	38	0	0.74	Sub	0.67	2.57			polymorphism	22.4	p.K396N	Passenger	SENGER/OT	damaging	Neutral	Tolerated
OVA_048_M1	METASTASIS	chr2	1.7E+08	1.7E+08	T	A	ABCB11	intronic	NA	NA	602	10	612	0.01634	20	0	20	0	0.09	Sub	0.56	3.94			polymorphism	0.117						
OVA_048_M2	METASTASIS	chr2	1.7E+08	1.7E+08	T	A	ABCB11	intronic	NA	NA	597	14	611	0.022913	20	0	20	0	0.18	Sub	0.4	3.87			polymorphism	0.117						
OVA_048_M3	METASTASIS	chr2	1.7E+08	1.7E+08	T	A	ABCB11	intronic	NA	NA	479	107	586	0.182594	20	0	20	0	1	Sub	0.59	3.39			polymorphism	0.117						
OVA_378_C2	PRIMARY	chr2	1.7E+08	1.7E+08	C	A	LRP2	exonic	ynonymous	00263816:	497	128	625	0.2048	31	0	31	0	0.93	Clonal	0.56	2.59			polymorphism	9.975	p.K4606N	Passenger	SENGER/OT	damaging	Neutral	Damaging
OVA_378_C2	PRIMARY	chr2	1.7E+08	1.7E+08	T	G	LRP2	exonic	ynonymous	00263816:	493	123	616	0.199675	30	0	30	0	0.91	Clonal	0.56	2.59			polymorphism	14.69	p.K4606Q	Passenger	SENGER/OT	benign	Neutral	Tolerated
OVA_048_M1	METASTASIS	chr2	1.7E+08	1.7E+08	-	G	LRP2	exonic	neshift inse	0263816:e	1041	464	1505	0.308306	32	1	33		1	Clonal	0.56	3.94			disease_causing		p.C4381Lfs	Passenger	SENGER/OT			
OVA_048_M2	METASTASIS	chr2	1.7E+08	1.7E+08	-	G	LRP2	exonic	neshift inse	0263816:e	1416	369	1785	0.206723	32	1	33		1	Clonal	0.4	3.87			disease_causing		p.C4381Lfs	Passenger	SENGER/OT			
OVA_048_M3	METASTASIS	chr6	1.7E+08	1.7E+08	A	T	WDR27	intronic	NA	NA	465	52	517	0.10058	37	0	37	0	1	Clonal	0.59	3.39			polymorphism	3.307						
OVA_013_M1	METASTASIS	chr6	1.7E+08	1.7E+08	C	G	WDR27	exonic	ynonymous	000033357	341	173	514	0.336576	63	1	64	0.015625	1	Clonal	0.67	3.06			polymorphism	4.838	p.G315A	Passenger	SENGER/OT	benign	Neutral	Tolerated
OVA_013_C1	PRIMARY	chr6	1.7E+08	1.7E+08	C	G	WDR27	exonic	ynonymous	000033357	348	235	583	0.403087	63	1	64	0.015625	1	Sub	0.72	3.1			polymorphism	4.838	p.G315A	Passenger	SENGER/OT	benign	Neutral	Tolerated
OVA_013_C2	PRIMARY	chr6	1.7E+08	1.7E+08	C	G	WDR27	exonic	ynonymous	000033357	352	219	571	0.383538	63	1	64	0.015625	0.88	Sub	0.87	3.05			polymorphism	4.838	p.G315A	Passenger	SENGER/OT	benign	Neutral	Tolerated
OVA_013_C3	PRIMARY	chr6	1.7E+08	1.7E+08	C	G	WDR27	exonic	ynonymous	000033357	351	249	600	0.415	63	1	64	0.015625	0.91	Sub	0.91	3.01			polymorphism	4.838	p.G315A	Passenger	SENGER/OT	benign	Neutral	Tolerated
OVA_013_M1	METASTASIS	chr1	1.71E+08	1.71E+08	A	T	PRRC2C	exonic	ynonymous	Q236L,ENSG	545	107	652	0.16411	43	0	43	0	0.82	Sub	0.67	3.06			disease_causing	24.5	p.Q236L	Passenger	SENGER/OT	damaging	Deleterious	Damaging
OVA_013_C1	PRIMARY	chr1	1.71E+08	1.71E+08	A	T	PRRC2C	exonic	ynonymous	Q236L,ENSG	530	131	661	0.198185	43	0	43	0	0.95	Sub	0.72	3.1			disease_causing	24.5	p.Q236L	Passenger	SENGER/OT	damaging	Deleterious	Damaging
OVA_013_C2	PRIMARY	chr1	1.71E+08	1.71E+08	A	T	PRRC2C	exonic	ynonymous	Q236L,ENSG	509	125	634	0.197161	43	0	43	0	0.85	Sub	0.87	3.05			disease_causing	24.5	p.Q236L	Passenger	SENGER/OT	damaging	Deleterious	Damaging
OVA_013_C3	PRIMARY	chr1	1.71E+08	1.71E+08	A	T	PRRC2C	exonic	ynonymous	Q236L,ENSG	523	159	682	0.233138	43	0	43	0	0.98	Sub	0.91	3.01			disease_causing	24.5	p.Q236L	Passenger	SENGER/OT	damaging	Deleterious	Damaging
OVA_048_M2	METASTASIS	chr3	1.72E+08	1.72E+08	C	A	FNDC3B	UTR5	NA	NA	599	17	616	0.027597	48	1	49	0.020408	0.28	Sub	0.4	3.87			polymorphism	6.949						
OVA_048_M3	METASTASIS	chr3	1.72E+08	1.72E+08	C	A	FNDC3B	UTR5	NA	NA	545	71	616	0.11526	48	1	49	0.020408	1	Sub	0.59	3.39			polymorphism	6.949						
OVA_003_M1	METASTASIS	chr2	1.72E+08	1.72E+08	G	-	TLK1	exonic	neshift dele	000442919:	349	127	476	0.266807	26	0	26		0.94	Sub	0.57	1.84					p.Q509Sfs	Passenger			NA	NA
OVA_003_M2	METASTASIS	chr2	1.72E+08	1.72E+08	G	-	TLK1	exonic	neshift dele	000442919:	1010	262	1272	0.205975	26	0	26		0.9	Sub	0.46	1.81					p.Q509Sfs	Passenger			NA	NA
OVA_003_M3	METASTASIS	chr2	1.72E+08	1.72E+08	G	-	TLK1	exonic	neshift dele	000442919:	1072	50	1122	0.044563	26	0	26		0.47	Sub	0.34	2.67					p.Q509Sfs	Passenger			NA	NA
OVA_003_C1	PRIMARY	chr2	1.72E+08	1.72E+08	G	-	TLK1	exonic	neshift dele	000442919:	256	194	450	0.431111	26	0	26		1	Sub	0.86	1.81					p.Q509Sfs	Passenger			NA	NA
OVA_003_C2	PRIMARY	chr2	1.72E+08	1.72E+08	G	-	TLK1	exonic	neshift dele	000442919:	307	109	416	0.262019	26	0	26		1	Sub	0.7	3.61					p.Q509Sfs	Passenger			NA	NA
OVA_003_C3	PRIMARY	chr2	1.72E+08	1.72E+08	G	-	TLK1	exonic	neshift dele	000442919:	379	200	579	0.345423	26	0	26		0.84	Sub	0.82	1.81					p.Q509Sfs	Passenger			NA	NA
OVA_365_M1	METASTASIS	chr2	1.75E+08	1.75E+08	T	C	SP3	exonic	ynonymous	000045578	397	199	596	0.333893	47	0	47	0	1	Clonal	0.85	1.87			disease_causing	26.9	p.K706E	Passenger	SENGER/OT	damaging	Deleterious	Damaging
OVA_048_C1	PRIMARY	chr3	1.75E+08	1.75E+08	G	T	NAALADL2	:RNA_intro	NA	NA	394	131	525	0.249524	34	0	34	0	1	Clonal	0.67	1.82			polymorphism	7.833						
OVA_048_C3	PRIMARY	chr3	1.75E+08	1.75E+08	G	T	NAALADL2	:RNA_intro	NA	NA	394	146	540	0.27037	34	0	34	0	1	Clonal	0.74	1.8			polymorphism	7.833						
OVA_003_M1	METASTASIS	chr5	1.76E+08	1.76E+08	GAAAAGAA	-	UIMC1	intronic	NA	NA	90	7	97	0.072165	41	0	41		0.18	Sub	0.57	1.84										
OVA_003_M2	METASTASIS	chr5	1.76E+08	1.76E+08	GAAAAGAA	-	UIMC1	intronic	NA	NA	4	9	13	0.692308	41	0	41		1	Sub	0.46	1.81										
OVA_003_M3	METASTASIS	chr5	1.76E+08	1.76E+08	GAAAAGAA	-	UIMC1	intronic	NA	NA	10	5	15	0.333333	41	0	41		1	Sub	0.34	2.67										
OVA_003_C1	PRIMARY	chr5	1.76E+08	1.76E+08	GAAAAGAA	-	UIMC1	intronic	NA	NA	4	4	8	0.5	41	0	41		0.66	Clonal	0.86	1.81										
OVA_003_C2	PRIMARY	chr5	1.76E+08	1.76E+08	GAAAAGAA	-	UIMC1	intronic	NA	NA	8	4	12	0.333333	41	0	41		0.95	Clonal	0.7	3.61										
OVA_003_C3	PRIMARY	chr5	1.76E+08	1.76E+08	GAAAAGAA	-	UIMC1	intronic	NA	NA	3	82	85	0.964706	41	0	41		1	Clonal	0.82	1.81										
OVA_013_M1	METASTASIS	chr5	1.77E+08	1.77E+08	G	C	MXD3	exonic	ynonymous	000004279	76	35	111	0.315315	41	0	41	0	0.94	Clonal	0.67	3.06			disease_causing	24.7	p.L80V	Passenger	CANCER	damaging	Deleterious	Damaging
OVA_013_C1	PRIMARY	chr5	1.77E+08	1.77E+08	G	C	MXD3	exonic	ynonymous	000004279	59	38	97	0.391753	41	0	41	0	1	Clonal	0.72	3.1			disease_causing	24.7	p.L80V	Passenger	CANCER	damaging	Deleterious	Damaging
OVA_013_C2	PRIMARY	chr5	1.77E+08	1.77E+08	G	C	MXD3	exonic	ynonymous	000004279	72	44	116	0.37931	41	0	41	0	0.87	Clonal	0.87	3.05			disease_causing	24.7	p.L80V	Passenger	CANCER	damaging	Deleterious	Damaging
OVA_013_C3	PRIMARY	chr5	1.77E+08	1.77E+08	G	C	MXD3	exonic	ynonymous	000004279	82	73	155	0.470968	41	0	41	0	1	Clonal	0.91	3.01			disease_causing	24.7	p.L80V	Passenger	CANCER	damaging	Deleterious	Damaging
OVA_365_M1	METASTASIS	chr4	1.77E+08	1.77E+08	C	A	WDR17	intronic	NA	NA	343	271	614	0.441368	37	0	37	0	1	Clonal	0.85	1.87			polymorphism	3.701						
OVA_365_M2	METASTASIS	chr4	1.77E+08	1.77E+08	C	A	WDR17	intronic	NA	NA	437	94	531	0.177024	37	0	37	0	0.88	Clonal	0.4	1.81			polymorphism	3.701						
OVA_365_M3	METASTASIS	chr4	1.77E+08	1.77E+08	C	A	WDR17	intronic	NA	NA	326	267	593	0.450253	37	0	37	0	1	Clonal	0.82	1.89			polymorphism	3.701						
OVA_365_C1	Contralateral ovary	chr4	1.77E+08	1.77E+08	C	A	WDR17	intronic	NA	NA	398	101	499	0.202405	37	0	37	0	0.77	Sub	0.42	1.83			polymorphism	3.701						
OVA_365_C2	PRIMARY	chr4	1.77E+08	1.77E+08	C	A	WDR17	intronic	NA	NA	403	161	564	0.285461	37	0	37	0	0.93	Sub	0.62	1.92			polymorphism	3.701						
OVA_003_M1	METASTASIS	chr3	1.79E+08	1.79E+08	G	T	PIK3CA	exonic	ynonymous	.R88L,ENSG	405	225	630	0.357143	69	0	69	0	1	Clonal	0.57	1.84	Yes	No	disease_causing	27.1	p.R88L	Driver	CANCER	damaging	Deleterious	Damaging
OVA_003_M2	METASTASIS	chr3	1.79E+08	1.79E+08	G	T	PIK3CA	exonic	ynonymous	.R88L,ENSG	431	184	615	0.299187	69	0	69	0	1	Clonal	0.46	1.81	Yes	No	disease_causing	27.1	p.R88L	Driver	CANCER	damaging	Deleterious	Damaging
OVA_003_M3	METASTASIS	chr3	1.79E+08	1.79E+08	G	T	PIK3CA	exonic	ynonymous	.R88L,ENSG	579	84	663	0.126697	69	0	69	0	1	Clonal	0.34	2.67	Yes	No	disease_causing	27.1	p.R88L	Driver	CANCER	damaging	Deleterious	Damaging
OVA_003_C1	PRIMARY	chr3	1.79E+08	1.79E+08	G	T	PIK3CA	exonic	ynonymous	.R88L,ENSG	390	311	701	0.443652	69	0	69	0	1	Clonal	0.86	1.81	Yes	No	disease_causing	27.1	p.R88L	Driver	CANCER	damaging	Deleterious	Damaging
OVA_003_C2	PRIMARY	chr3	1.79E+08	1.79E+08	G	T	PIK3CA	exonic	ynonymous	.R88L,ENSG	397	321	718	0.447075	69	0	69	0	1	Clonal	0.7	3.61	Yes	No	disease_causing	27.1	p.R88L	Driver	CANCER	damaging	Deleterious	Damaging
OVA_003_C3	PRIMARY	chr3	1.79E+08	1.79E+08	G	T	PIK3CA	exonic	ynonymous	.R88L,ENSG	396	317	713	0.4446	69	0	69	0	1	Clonal	0.82	1.81	Yes	No	disease_causing	27.1	p.R88L	Driver	CANCER	damaging	Deleterious	Damaging
OVA_048_C3	PRIMARY	chr5	1.79E+08	1.79E+08	G	A	HNRNPH1	intronic	NA	NA	456	47	503	0.093439	54	0	54	0	0.25	Sub	0.74	1.8			polymorphism	0.669						
OVA_047_M1	METASTASIS	chr2	1.79E+08	1.79E+08	C	T	TTN	exonic	ynonymous	4786I,ENSG	607	145	752	0.192819	37	0	37	0	1	Clonal	0.47	2.01			polymorphism	16.38	p.V33726I	Passenger	SENGER/OT	benign	Neutral	Tolerated
OVA_047_M2	METASTASIS	chr2	1.79E+08	1.79E+08	C	T	TTN	exonic	ynonymous	4786I,ENSG	618	150	768	0.195313	37	0	37	0	1	Clonal	0.44	2.01			polymorphism	16.38	p.V33726I	Passenger	SENGER/OT	benign	Neutral	Tolerated
OVA_047_M3	METASTASIS	chr2	1.79E+08	1.79E+08	C	T	TTN	exonic	ynonymous	4786I,ENSG	514	110	624	0.176282	37	0	37	0	0.94	Clonal	0.6	3.02			polymorphism	16.38	p.V33726I	Passenger	SENGER/OT	benign	Neutral	Tolerated
OVA_048_M1	METASTASIS	chr1	1.79E+08	1.79E+08	C	A	AXDND1	intronic	NA	NA	249	139	388	0.358247	21	0	21	0	1	Clonal	0.56	3.94			polymorphism	2.534						
OVA_048_M2	METASTASIS	chr1	1.79E+08	1.79E+08	C	A	AXDND1	intronic	NA	NA	326	119	445	0.267416	21	0	21	0	1	Clonal	0.4	3.87			polymorphism	2.534						

Sample	Type	Chr	Start	End	Ref	Alt	Gene	unc.ensGen	icFunc.ens	hange.ensG	t_ref	t_alt	t_total	t_VAF	n_ref	n_alt	n_total	ormal_VA	ncer_cell_f	on-based C	purity	ploidy	Cancer Gene	Driver Gene	Mutation Taster	ADD(PHRE	otein_chan	CHASM	ATHMM_Pr	OLYPHEN-	REDICTION	ICTION (cutoff=0.05)
OVA_048_M3	METASTASIS	chr1	1.79E+08	1.79E+08	C	A	AXDND1	intronic	NA	NA	362	85	447	0.190157	21	0	21	0	1	Clonal	0.59	3.39			polymorphism	2.534						
OVA_047_M1	METASTASIS	chr2	1.79E+08	1.79E+08	C	T	TTN	exonic	ynonymous	372N,ENSG	566	160	726	0.220386	60	0	60	0	1	Sub	0.47	2.01			disease_causing	24.7	p.D27312N	Driver	SENGER/OT	damaging	Deleterious	Tolerated
OVA_047_M2	METASTASIS	chr2	1.79E+08	1.79E+08	C	T	TTN	exonic	ynonymous	372N,ENSG	642	138	780	0.176923	60	0	60	0	0.97	Sub	0.44	2.01			disease_causing	24.7	p.D27312N	Driver	SENGER/OT	damaging	Deleterious	Tolerated
OVA_047_M3	METASTASIS	chr2	1.79E+08	1.79E+08	C	T	TTN	exonic	ynonymous	372N,ENSG	558	99	657	0.150685	60	0	60	0	0.8	Sub	0.6	3.02			disease_causing	24.7	p.D27312N	Driver	SENGER/OT	damaging	Deleterious	Tolerated
OVA_378_M1	METASTASIS	chr2	1.79E+08	1.79E+08	C	T	TTN	exonic	ynonymous	2410I,ENSG	543	145	688	0.210756	121	0	121	0	1	Clonal	0.72	2.57			disease_causing	23.2	p.V21350I	Passenger	SENGER/OT	damaging	Neutral	Tolerated
OVA_378_M2	METASTASIS	chr2	1.79E+08	1.79E+08	C	T	TTN	exonic	ynonymous	2410I,ENSG	563	140	703	0.199147	121	0	121	0	0.86	Clonal	0.85	2.57			disease_causing	23.2	p.V21350I	Passenger	SENGER/OT	damaging	Neutral	Tolerated
OVA_378_M3	METASTASIS	chr2	1.79E+08	1.79E+08	C	T	TTN	exonic	ynonymous	2410I,ENSG	594	136	730	0.186301	121	0	121	0	0.93	Clonal	0.67	2.57			disease_causing	23.2	p.V21350I	Passenger	SENGER/OT	damaging	Neutral	Tolerated
OVA_378_C1	PRIMARY	chr2	1.79E+08	1.79E+08	C	T	TTN	exonic	ynonymous	2410I,ENSG	605	129	734	0.175749	121	0	121	0	0.95	Sub	0.59	2.6			disease_causing	23.2	p.V21350I	Passenger	SENGER/OT	damaging	Neutral	Tolerated
OVA_378_C2	PRIMARY	chr2	1.79E+08	1.79E+08	C	T	TTN	exonic	ynonymous	2410I,ENSG	638	25	663	0.037707	121	0	121	0	0.17	Sub	0.56	2.59			disease_causing	23.2	p.V21350I	Passenger	SENGER/OT	damaging	Neutral	Tolerated
OVA_378_M1	METASTASIS	chr1	1.8E+08	1.8E+08	G	T	ACBD6	intronic	NA	NA	61	667	728	0.916209	28	0	28	0	1	Clonal	0.72	2.57			polymorphism	0.521						
OVA_378_M2	METASTASIS	chr1	1.8E+08	1.8E+08	G	T	ACBD6	intronic	NA	NA	65	635	700	0.907143	28	0	28	0	1	Clonal	0.85	2.57			polymorphism	0.521						
OVA_378_M3	METASTASIS	chr1	1.8E+08	1.8E+08	G	T	ACBD6	intronic	NA	NA	83	706	789	0.894804	28	0	28	0	1	Clonal	0.67	2.57			polymorphism	0.521						
OVA_378_C1	PRIMARY	chr1	1.8E+08	1.8E+08	G	T	ACBD6	intronic	NA	NA	58	717	775	0.925161	28	0	28	0	1	Clonal	0.59	2.6			polymorphism	0.521						
OVA_378_C2	PRIMARY	chr1	1.8E+08	1.8E+08	G	T	ACBD6	intronic	NA	NA	55	697	752	0.926862	28	0	28	0	1	Clonal	0.56	2.59			polymorphism	0.521						
OVA_378_C3	PRIMARY	chr1	1.8E+08	1.8E+08	G	T	ACBD6	intronic	NA	NA	79	654	733	0.892224	28	0	28	0	1	Clonal	0.51	2.6			polymorphism	0.521						
OVA_048_M1	METASTASIS	chr1	1.81E+08	1.81E+08	C	T	KIAA1614	exonic	nonymous S	245D,ENSG	199	22	221	0.099548	26	0	26	0	0.55	Sub	0.56	3.94			polymorphism	6.304		Passenger			Neutral	Tolerated
OVA_048_M2	METASTASIS	chr1	1.81E+08	1.81E+08	C	T	KIAA1614	exonic	nonymous S	245D,ENSG	205	14	219	0.063927	26	0	26	0	0.45	Sub	0.4	3.87			polymorphism	6.304		Passenger			Neutral	Tolerated
OVA_048_M3	METASTASIS	chr1	1.81E+08	1.81E+08	C	T	KIAA1614	exonic	nonymous S	245D,ENSG	195	36	231	0.155844	26	0	26	0	1	Sub	0.59	3.39			polymorphism	6.304		Passenger			Neutral	Tolerated
OVA_048_M1	METASTASIS	chr1	1.82E+08	1.82E+08	C	G	CACNA1E	exonic	ynonymous	000367570	386	215	601	0.357737	12	0	12	0	1	Clonal	0.56	3.94			disease_causing	27.6	p.F340L	Passenger		damaging	Deleterious	Damaging
OVA_048_M2	METASTASIS	chr1	1.82E+08	1.82E+08	C	G	CACNA1E	exonic	ynonymous	000367570	389	207	596	0.347315	12	0	12	0	1	Clonal	0.4	3.87			disease_causing	27.6	p.F340L	Passenger		damaging	Deleterious	Damaging
OVA_048_M3	METASTASIS	chr1	1.82E+08	1.82E+08	C	G	CACNA1E	exonic	ynonymous	000367570	337	242	579	0.417962	12	0	12	0	1	Clonal	0.59	3.39			disease_causing	27.6	p.F340L	Passenger		damaging	Deleterious	Damaging
OVA_365_M1	METASTASIS	chr1	1.82E+08	1.82E+08	C	T	CACNA1E	intronic	NA	NA	273	225	498	0.451807	48	0	48	0	1	Sub	0.85	1.87			polymorphism	10.97						
OVA_365_M2	METASTASIS	chr1	1.82E+08	1.82E+08	C	T	CACNA1E	intronic	NA	NA	339	52	391	0.132992	48	0	48	0	0.66	Sub	0.4	1.81			polymorphism	10.97						
OVA_365_M3	METASTASIS	chr1	1.82E+08	1.82E+08	C	T	CACNA1E	intronic	NA	NA	282	180	462	0.38961	48	0	48	0	0.95	Sub	0.82	1.89			polymorphism	10.97						
OVA_365_C1	Contralateral ovary	chr1	1.82E+08	1.82E+08	C	T	CACNA1E	intronic	NA	NA	269	88	357	0.246499	48	0	48	0	1	Clonal	0.42	1.83			polymorphism	10.97						
OVA_365_C2	PRIMARY	chr1	1.82E+08	1.82E+08	C	T	CACNA1E	intronic	NA	NA	319	140	459	0.305011	48	0	48	0	0.99	Clonal	0.62	1.92			polymorphism	10.97						
OVA_048_C2	PRIMARY	chr2	1.83E+08	1.83E+08	A	G	SSFA2	exonic	ynonymous	461A,ENSG	259	131	390	0.335897	27	0	27	0	0.93	Clonal	0.72	1.85			polymorphism	12.77	p.T461A	Passenger	SENGER/OT	benign	Neutral	Tolerated
OVA_013_M1	METASTASIS	chr3	1.83E+08	1.83E+08	G	A	MCCC1	UTR5	NA	NA	354	108	462	0.233766	61	0	61	0	0.93	Clonal	0.67	3.06			polymorphism	1.698						
OVA_013_C1	PRIMARY	chr3	1.83E+08	1.83E+08	G	A	MCCC1	UTR5	NA	NA	372	130	502	0.258964	61	0	61	0	0.98	Sub	0.72	3.1			polymorphism	1.698						
OVA_013_C2	PRIMARY	chr3	1.83E+08	1.83E+08	G	A	MCCC1	UTR5	NA	NA	301	104	405	0.25679	61	0	61	0	0.85	Sub	0.87	3.05			polymorphism	1.698						
OVA_013_C3	PRIMARY	chr3	1.83E+08	1.83E+08	G	A	MCCC1	UTR5	NA	NA	415	133	548	0.242701	61	0	61	0	0.77	Sub	0.91	3.01			polymorphism	1.698						
OVA_003_M1	METASTASIS	chr3	1.84E+08	1.84E+08	G	C	HTR3C	intronic	NA	NA	405	194	599	0.323873	19	0	19	0	1	Clonal	0.57	1.84			polymorphism	5.504						
OVA_003_M2	METASTASIS	chr3	1.84E+08	1.84E+08	G	C	HTR3C	intronic	NA	NA	417	180	597	0.301508	19	0	19	0	1	Clonal	0.46	1.81			polymorphism	5.504						
OVA_003_M3	METASTASIS	chr3	1.84E+08	1.84E+08	G	C	HTR3C	intronic	NA	NA	544	53	597	0.088777	19	0	19	0	1	Clonal	0.34	2.67			polymorphism	5.504						
OVA_003_C1	PRIMARY	chr3	1.84E+08	1.84E+08	G	C	HTR3C	intronic	NA	NA	373	264	637	0.414443	19	0	19	0	1	Clonal	0.86	1.81			polymorphism	5.504						
OVA_003_C2	PRIMARY	chr3	1.84E+08	1.84E+08	G	C	HTR3C	intronic	NA	NA	381	256	637	0.401884	19	0	19	0	1	Clonal	0.7	3.61			polymorphism	5.504						
OVA_003_C3	PRIMARY	chr3	1.84E+08	1.84E+08	G	C	HTR3C	intronic	NA	NA	386	306	692	0.442197	19	0	19	0	1	Clonal	0.82	1.81			polymorphism	5.504						
OVA_048_M1	METASTASIS	chr3	1.84E+08	1.84E+08	C	G	DVL3	exonic	ynonymous	000004233	373	183	556	0.329137	87	0	87	0	1	Clonal	0.56	3.94			disease_causing	24.8	p.S177C	Passenger	SENGER/OT	damaging	Deleterious	Damaging
OVA_048_M2	METASTASIS	chr3	1.84E+08	1.84E+08	C	G	DVL3	exonic	ynonymous	000004233	374	173	547	0.316271	87	0	87	0	1	Clonal	0.4	3.87			disease_causing	24.8	p.S177C	Passenger	SENGER/OT	damaging	Deleterious	Damaging
OVA_048_M3	METASTASIS	chr3	1.84E+08	1.84E+08	C	G	DVL3	exonic	ynonymous	000004233	338	191	529	0.361059	87	0	87	0	1	Clonal	0.59	3.39			disease_causing	24.8	p.S177C	Passenger	SENGER/OT	damaging	Deleterious	Damaging
OVA_048_C1	PRIMARY	chr3	1.84E+08	1.84E+08	C	G	DVL3	exonic	ynonymous	000004233	225	63	288	0.21875	87	0	87	0	0.87	Clonal	0.67	1.82			disease_causing	24.8	p.S177C	Passenger	SENGER/OT	damaging	Deleterious	Damaging
OVA_048_C2	PRIMARY	chr3	1.84E+08	1.84E+08	C	G	DVL3	exonic	ynonymous	000004233	354	143	497	0.287726	87	0	87	0	1	Clonal	0.72	1.85			disease_causing	24.8	p.S177C	Passenger	SENGER/OT	damaging	Deleterious	Damaging
OVA_048_C3	PRIMARY	chr3	1.84E+08	1.84E+08	C	G	DVL3	exonic	ynonymous	000004233	261	77	338	0.227811	87	0	87	0	0.84	Clonal	0.74	1.8			disease_causing	24.8	p.S177C	Passenger	SENGER/OT	damaging	Deleterious	Damaging
OVA_365_C1	Contralateral ovary	chr3	1.84E+08	1.84E+08	C	T	ABCF3	intronic	NA	NA	289	78	367	0.212534	83	0	83	0	1	Clonal	0.42	1.83			polymorphism	6.944						
OVA_365_M1	METASTASIS	chr3	1.84E+08	1.84E+08	A	G	CHRD	intronic	NA	NA	25	29	54	0.537037	100	0	100	0	1	Clonal	0.85	1.87			polymorphism	7.275						
OVA_365_M2	METASTASIS	chr3	1.84E+08	1.84E+08	A	G	CHRD	intronic	NA	NA	25	6	31	0.193548	100	0	100	0	0.96	Clonal	0.4	1.81			polymorphism	7.275						
OVA_365_M3	METASTASIS	chr3	1.84E+08	1.84E+08	A	G	CHRD	intronic	NA	NA	22	19	41	0.463415	100	0	100	0	1	Clonal	0.82	1.89			polymorphism	7.275						
OVA_365_C1	Contralateral ovary	chr3	1.84E+08	1.84E+08	A	G	CHRD	intronic	NA	NA	27	4	31	0.129032	100	0	100	0	0.75	Clonal	0.42	1.83			polymorphism	7.275						
OVA_365_C2	PRIMARY	chr3	1.84E+08	1.84E+08	A	G	CHRD	intronic	NA	NA	33	14	47	0.297872	100	0	100	0	0.97	Clonal	0.62	1.92			polymorphism	7.275						
OVA_013_C2	PRIMARY	chr3	1.84E+08	1.84E+08	A	T	CHRD;EPHB	UTR3	NA	NA	485	99	584	0.169521	42	0	42	0	0.56	Sub	0.87	3.05			polymorphism	2.379						
OVA_047_C1	PRIMARY	chr4	1.86E+08	1.86E+08	C	T	CASP3	exonic	ynonymous	A72T,ENSG	421	206	627	0.328549	55	0	55	0	1	Sub	0.6	2.91	Yes	Yes	disease_causing	25	p.A72T	Passenger	SENGER/OT	damaging	Neutral	Tolerated
OVA_047_C2	PRIMARY	chr4	1.86E+08	1.86E+08	C	T	CASP3	exonic	ynonymous	A72T,ENSG	448	161	609	0.264368	55	0	55	0	0.83	Sub	0.64	2.76	Yes	Yes	disease_causing	25	p.A72T	Passenger	SENGER/OT	damaging	Neutral	Tolerated
OVA_047_C3	Contralateral ovary	chr4	1.86E+08	1.86E+08	C	T	CASP3	exonic	ynonymous	A72T,ENSG	436	192	628	0.305732	55	0	55	0	1	Clonal	0.6	2.94	Yes	Yes	disease_causing	25	p.A72T	Passenger	SENGER/OT	damaging	Neutral	Tolerated
OVA_047_C1	PRIMARY	chr1	1.86E+08	1.86E+08	C	A	HMCN1	exonic	ynonymous	4062K,ENSG	555	101	656	0.153963	50	0	50	0	0.82	Sub	0.6	2.91			disease_causing	17.3	p.N4062K	Passenger	SENGER/OT	damaging	Deleterious	Damaging
OVA_047_C2	PRIMARY	chr1	1.86E+08	1.86E+08	C	A	HMCN1	exonic	ynonymous	4062K,ENSG	522	102	624	0.163462	50	0	50	0	0.84	Sub	0.64	2.76			disease_causing	17.3	p.N4062K	Passenger	SENGER/OT	damaging	Deleterious	Damaging
OVA_047_C3	Contralateral ovary	chr1	1.86E+08	1.86E+08	C	A	HMCN1	exonic	ynonymous	4062K,ENSG	528	101	629	0.160572	50	0	50	0	0.86	Clonal	0.6	2.94			disease_causing	17.3	p.N4062K	Passenger	SENGER/OT	damaging	Deleterious	Damaging
OVA_047_M1	METASTASIS	chr2	1.88E+08	1.88E+08	C	G	ITGAV	exonic	ynonymous	000261023	595	136	731	0.186047	66	1	67	0.014925	0.97	Sub	0.47	2.01	Yes	Yes	disease_causing	27.7	p.I647M	Passenger	SENGER/OT	damaging	Neutral	Damaging
OVA_047_M2	METASTASIS	chr2	1.88E+08	1.88E+08	C	G	ITGAV	exonic	ynonymous	000261023	610	121	731	0.165527	66	1	67	0.014925	0.91	Sub	0.44	2.01	Yes	Yes	disease_causing	27.7	p.I647M	Passenger	SENGER/OT	damaging	Neutral	Damaging
OVA_047_M3	METASTASIS	chr2	1.88E+08	1.88E+08	C	G	ITGAV	exonic	ynonymous	000261023	486	62	548	0.113139	66	1	67	0.014925	0.6	Sub	0.6	3.02	Yes	Yes	disease_causing	27.7	p.I647M	Passenger	SENGER/OT	damaging	Neutral	Damaging
OVA_048_C1	PRIMARY	chr4	1.88E+08	1.88E+08	T	-	FAT1	exonic	neshift dele	00441802:	180	73	253	0.288538	41	0	41		0.57	Sub	0.67	1.82	Yes	Yes			p.K1335Sfs	Passenger			NA	NA
OVA_365_M1	METASTASIS	chr2	1.88E+08	1.88E+08	C	T	ZSWIM2	exonic	ynonymous	000002951	240	296	536	0.552239	83	0	83	0	1	Sub	0.85	1.87			disease_causing	24.2	p.A96T	Passenger	SENGER/OT	damaging	Neutral	Tolerated
OVA_365_M2	METASTASIS	chr2	1.88E+08	1.88E+08	C	T	ZSWIM2	exonic	ynonymous	000002951	221	38	259	0.146718	83	0	83	0	0.73	Sub	0.4	1.81			disease_causing	24.2	p.A96T	Passenger	SENGER/OT	damaging	Neutral	Tolerated
OVA_365_M3	METASTASIS	chr2	1.88E+08	1.88E+08	C	T	ZSWIM2	exonic	ynonymous	000002951	259	248	507	0.489152	83	0	83	0	1	Sub	0.82	1.89			disease_causing	24.2	p.A96T	Passenger	SENGER/OT	damaging	Neutral	Tolerated
OVA_365_C1	Contralateral ovary	chr2	1.88E+08	1.88E+08	C	T	ZSWIM2	exonic	ynonymous	000002951	230	83	313	0.265176	83	0	83	0	1	Clonal	0.42	1.83			disease_causing	24.2	p.A96T	Passenger	SENGER/OT	damaging	Neutral	Tolerated
OVA_365_C2	PRIMARY	chr2	1.88E+08	1.88E+08	C	T	ZSWIM2	exonic	ynonymous	000002951	339	125	464	0.269397	83	0	83	0	0.87	Clonal	0.62	1.92			disease_causing	24.2	p.A96T	Passenger	SENGER/OT	damaging	Neutral	Tolerated
OVA_048_M1	METASTASIS	chr4	1.89E+08	1.89E+08	C	G	ZFP42	exonic	ynonymous	D70E,ENSG	220	308	528	0.583333	33	0	33	0	1	Clonal	0.56	3.94			polymorphism	15.2	p.D70E	Passenger		damaging	Neutral	Damaging
OVA_048_M2	METASTASIS	chr4	1.89E+08	1.89E+08	C	G	ZFP42	exonic	ynonymous	D70E,ENSG	319	206	525	0.392381	33	0	33	0	1	Clonal	0.4	3.87			polymorphism	15.2	p.D70E	Passenger		damaging	Neutral	Damaging
OVA_048_M3	METASTASIS	chr4	1.89E+08	1.89E+08	C	G	ZFP42	exonic	ynonymous	D70E,ENSG	264	227	491	0.462322	33	0	33	0	1	Clonal	0.59	3.39			polymorphism	15.2	p.D70E	Passenger		damaging	Neutral	Damaging
OVA_048_C1	PRIMARY	chr4	1.89E+08	1.89E+08	C	G	ZFP42	exonic	ynonymous	D70E,ENSG	105	189	294	0.642857	33	0	33	0	1	Clonal	0.67	1.82			polymorphism	15.2	p.D70E	Passenger		damaging	Neutral	Damaging
OVA_048_C2	PRIMARY	chr4	1.89E+08	1.89E+08	C	G	ZFP42	exonic	ynonymous	D70E,ENSG	179	253	432	0.585648	33	0	33	0	1	Clonal	0.72	1.85			polymorphism	15.2	p.D70E	Passenger		damaging	Neutral	Damaging
OVA_048_C3	PRIMARY	chr4	1.89E+08	1.89E+08	C	G	ZFP42	exonic	ynonymous	D70E,ENSG	105	203	308	0.659091	33	0	33	0	1	Clonal	0.74	1.8			polymorphism	15.2	p.D70E	Passenger		damaging	Neutral	Damaging
OVA_047_C1	PRIMARY	chr2	1.9E+08	1.9E+08	C	A	COL3A1	intronic	NA	NA	528	74	602	0.122924	28	0	28	0	0.66	Sub	0.6	2.91	Yes		polymorphism	2.898						
OVA_047_C2	PRIMARY	chr2	1.9E+08	1.9E+08	C	A	COL3A1	intronic	NA	NA	499	75	574	0.130662	28	0	28	0	0.67	Sub	0.64	2.76	Yes		polymorphism	2.898						
OVA_047_C3	Contralateral ovary	chr2	1.9E+08	1.9E+08	C	A	COL3A1	intronic	NA	NA	420	73	493	0.148073	28	0	28	0	0.79	Sub	0.6	2.94	Yes		polymorphism	2.898						
OVA_047_M1	METASTASIS	chr2	1.9E+08	1.9E+08	G	T	COL3A1	splicing	NA	NA	563	126	689	0.182874	54	0	54	0	0.95	Sub	0.47	2.01	Yes		disease_causing_automat	34		Passenger				
OVA_047_M2	METASTASIS	chr2	1.9E+08	1.9E+08	G	T	COL3A1	splicing	NA	NA	607	95	702	0.135328	54	0	54	0	0.75	Sub	0.44	2.01	Yes		disease_causing_automat	34		Passenger				
OVA_047_M3	METASTASIS	chr2	1.9E+08	1.9E+08	G	T	COL3A1	splicing	NA	NA	392	35	427	0.081967	54	0	54	0	0.44	Sub	0.6	3.02	Yes		disease_causing_automat	34		Passenger				
OVA_048_C1	PRIMARY	chr2	1.92E+08	1.92E+08	C	G	MYO1B	intronic	NA	NA	219	237	456	0.519737	22	0	22	0	1	Clonal	0.67	1.82			polymorphism	8.001						
OVA_048_C3	PRIMARY	chr2	1.92E+08	1.92E+08	C	G	MYO1B	intronic	NA	NA	221	104	325	0.32	22	0	22	0	0.86	Clonal	0.74	1.8			polymorphism	8.001						
OVA_003_M1	METASTASIS	chr2	1.93E+08	1.93E+08	T	G	NABP1	intronic	NA	NA	547	94	641	0.146646	42	0	42	0	0.81	Sub	0.57	1.84			polymorphism	12.39						
OVA_003_M2	METASTASIS	chr2	1.93E+08	1.93E+08	T	G	NABP1	intronic	NA	NA	589	78	667	0.116942	42	0	42	0	0.63	Sub	0.46	1.81			polymorphism	12.39						
OVA_003_M3	METASTASIS	chr2	1.93E+08	1.93E+08	T	G	NABP1	intronic	NA	NA	653	34	687	0.049491	42	0	42	0	0.77	Sub	0.34	2.67			polymorphism	12.39						
OVA_003_C1	PRIMARY	chr2	1.93E+08	1.93E+08	T	G	NABP1	intronic	NA	NA	551	170	721	0.235784	42	0	42	0	0.78	Sub	0.86	1.81			polymorphism	12.39						
OVA_003_C2	PRIMARY	chr2	1.93E+08	1.93E+08	T	G	NABP1	intronic	NA	NA	534	149	683	0.218155	42	0	42	0	1	Sub	0.7	3.61			polymorphism	12.39						
OVA_003_C3	PRIMARY	chr2	1.93E+08	1.93E+08	T	G	NABP1	intronic	NA	NA	575	168	743	0.22611	42	0	42	0	0.77	Sub	0.82	1.81			polymorphism	12.39						
OVA_048_M3	METASTASIS	chr2	1.93E+08	1.93E+08	G	C	TMEFF2	exonic	ynonymous	000003923	357	29	386	0.07513	41	0	41	0	0.74	Clonal	0.59	3.39			disease_causing	15.42	p.D61E	Passenger	SENGER/OT	benign	Neutral	Tolerated
OVA_365_C2	PRIMARY	chr3	1.93E+08	1.93E+08	T	A	ATP13A4	exonic	ynonymous	00342695:e	468	34	502	0.067729	65	0	65	0	0.22	Sub	0.62	1.92			polymorphism	18.96	p.H1172L	Passenger	SENGER/OT	benign	Neutral	Tolerated
OVA_047_M1	METASTASIS	chr1	1.96E+08	1.96E+08	A	G	KCNT2	intronic	NA	NA	568	72	640	0.1125	43	1	44	0.022727	0.7	Sub	0.47	2.01			polymorphism	0.39						
OVA_365_M1	METASTASIS	chr1	1.97E+08	1.97E+08	T	C	CRB1	intronic	NA	NA	296	216	512	0.421875	56	0	56	0	1	Clonal	0.85	1.87			polymorphism	9.306						
OVA_365_M2	METASTASIS	chr1	1.97E+08	1.97E+08	T	C	CRB1	intronic	NA	NA	298	63	361	0.174515	56	0	56	0	0.87	Clonal	0.4	1.81			polymorphism	9.306						
OVA_365_M3	METASTASIS	chr1	1.97E+08	1.97E+08	T	C	CRB1	intronic	NA	NA	282	211	493	0.427992	56	0	56	0	1	Clonal	0.82	1.89			polymorphism	9.306						
OVA_365_C2	PRIMARY	chr1	1.97E+08	1.97E+08	T	C	CRB1	intronic	NA	NA	332	143	475	0.301053	56	0	56	0	0.98	Clonal	0.62	1.92			polymorphism	9.306						
OVA_013_M1	METASTASIS	chr1	1.97E+08	1.97E+08	C	T	CRB1	intronic	NA	NA	520	133	653	0.203675	41	0	41	0	1	Clonal	0.67	3.06			polymorphism	1.425						
OVA_013_C1	PRIMARY	chr1	1.97E+08	1.97E+08	C	T	CRB1	intronic	NA	NA	518	130	648	0.200617	41	0	41	0	0.96	Sub	0.72	3.1			polymorphism	1.425						
OVA_013_C2	PRIMARY	chr1	1.97E+08	1.97E+08	C	T	CRB1	intronic	NA	NA	538	119	657	0.181126	41	0	41	0	0.78	Sub	0.87	3.05			polymorphism	1.425						
OVA_013_C3	PRIMARY	chr1	1.97E+08	1.97E+08	C	T	CRB1	intronic	NA	NA	548	138	686	0.201166	41	0	41	0	0.84	Sub	0.91	3.01			polymorphism	1.425						
OVA_003_M1	METASTASIS	chr2	1.98E+08	1.98E+08	G	A	PGAP1	intronic	NA	NA	518	106	624	0.169872	31	0	31	0	0.94	Sub	0.57	1.84			polymorphism	0.408						
OVA_003_M2	METASTASIS	chr2	1.98E+08	1.98E+08	G	A	PGAP1	intronic	NA	NA	548	94	642	0.146417	31	0	31	0	0.79	Sub	0.46	1.81			polymorphism	0.408						
OVA_003_M3	METASTASIS	chr2	1.98E+08	1.98E+08	G	A	PGAP1	intronic	NA	NA	615	29	644	0.045031	31	0	31	0	0.7	Sub	0.34	2.67			polymorphism	0.408						
OVA_003_C1	PRIMARY	chr2	1.98E+08	1.98E+08	G	A	PGAP1	intronic	NA	NA	506	143	649	0.220339	31	0	31	0	0.73	Sub	0.86	1.81			polymorphism	0.408						

Sample	Type	Chr	Start	End	Ref	Alt	Gene	unc.ensGen	icFunc.ens	hange.ens(	t_ref	t_alt	t_total	t_VAF	n_ref	n_alt	n_total	normal_VA	ncer_cell_f	on-based C	purity	ploidy	Cancer Gene	Driver Gene	Mutation Taster	ADD(PHRE	otein_chan	CHASM	ATHMM_Pr	OLYPHEN-	REDICTION	ICTION (cutoff=0.05)
OVA_003_C2	PRIMARY	chr2	1.98E+08	1.98E+08	G	A	PGAP1	intronic	NA	NA	509	149	658	0.226444	31	0	31	0	1	Sub	0.7	3.61			polymorphism	0.408						
OVA_003_C3	PRIMARY	chr2	1.98E+08	1.98E+08	G	A	PGAP1	intronic	NA	NA	547	137	684	0.200292	31	0	31	0	0.69	Sub	0.82	1.81			polymorphism	0.408						
OVA_013_M1	METASTASIS	chr2	2E+08	2E+08	C	A	SATB2	exonic	ynonymou	000417098	326	85	411	0.206813	86	1	87	0.011494	0.82	Clonal	0.67	3.06			disease_causing	25.7	p.D609Y	Passenger	CANCER	damaging	Deleterious	Damaging
OVA_013_C1	PRIMARY	chr2	2E+08	2E+08	C	A	SATB2	exonic	ynonymou	000417098	356	148	504	0.293651	86	1	87	0.011494	1	Sub	0.72	3.1			disease_causing	25.7	p.D609Y	Passenger	CANCER	damaging	Deleterious	Damaging
OVA_013_C2	PRIMARY	chr2	2E+08	2E+08	C	A	SATB2	exonic	ynonymou	000417098	280	124	404	0.306931	86	1	87	0.011494	0.71	Sub	0.87	3.05			disease_causing	25.7	p.D609Y	Passenger	CANCER	damaging	Deleterious	Damaging
OVA_013_C3	PRIMARY	chr2	2E+08	2E+08	C	A	SATB2	exonic	ynonymou	000417098	386	169	555	0.304505	86	1	87	0.011494	0.97	Sub	0.91	3.01			disease_causing	25.7	p.D609Y	Passenger	CANCER	damaging	Deleterious	Damaging
OVA_047_M1	METASTASIS	chr1	2.01E+08	2.01E+08	G	A	KIF21B	exonic	nonymous S	1408S,ENSG	297	80	377	0.212202	74	0	74	0	1	Clonal	0.47	2.01			disease_causing	7.125		Passenger			Neutral	Tolerated
OVA_047_M2	METASTASIS	chr1	2.01E+08	2.01E+08	G	A	KIF21B	exonic	nonymous S	1408S,ENSG	374	88	462	0.190476	74	0	74	0	1	Clonal	0.44	2.01			disease_causing	7.125		Passenger			Neutral	Tolerated
OVA_047_M3	METASTASIS	chr1	2.01E+08	2.01E+08	G	A	KIF21B	exonic	nonymous S	1408S,ENSG	100	35	135	0.259259	74	0	74	0	1	Clonal	0.6	3.02			disease_causing	7.125		Passenger			Neutral	Tolerated
OVA_048_M1	METASTASIS	chr1	2.02E+08	2.02E+08	G	A	IPO9	intronic	NA	NA	318	91	409	0.222494	9	0	9	0	1	Clonal	0.56	3.94			polymorphism	9.404						
OVA_048_M2	METASTASIS	chr1	2.02E+08	2.02E+08	G	A	IPO9	intronic	NA	NA	407	59	466	0.126609	9	0	9	0	0.88	Clonal	0.4	3.87			polymorphism	9.404						
OVA_048_M1	METASTASIS	chr2	2.02E+08	2.02E+08	G	T	FAM126B	exonic	ynonymou	S131Y,ENSG	105	58	163	0.355828	39	1	40	0.025	1	Clonal	0.56	3.94			disease_causing	28.3	p.S131Y	Passenger	no weights	damaging	Deleterious	Damaging
OVA_048_M2	METASTASIS	chr2	2.02E+08	2.02E+08	G	T	FAM126B	exonic	ynonymou	S131Y,ENSG	141	73	214	0.341121	39	1	40	0.025	1	Clonal	0.4	3.87			disease_causing	28.3	p.S131Y	Passenger	no weights	damaging	Deleterious	Damaging
OVA_048_M3	METASTASIS	chr2	2.02E+08	2.02E+08	G	T	FAM126B	exonic	ynonymou	S131Y,ENSG	93	95	188	0.505319	39	1	40	0.025	1	Clonal	0.59	3.39			disease_causing	28.3	p.S131Y	Passenger	no weights	damaging	Deleterious	Damaging
OVA_048_C1	PRIMARY	chr2	2.02E+08	2.02E+08	G	T	FAM126B	exonic	ynonymou	S131Y,ENSG	89	50	139	0.359712	39	1	40	0.025	1	Sub	0.67	1.82			disease_causing	28.3	p.S131Y	Passenger	no weights	damaging	Deleterious	Damaging
OVA_048_C2	PRIMARY	chr2	2.02E+08	2.02E+08	G	T	FAM126B	exonic	ynonymou	S131Y,ENSG	131	63	194	0.324742	39	1	40	0.025	0.9	Sub	0.72	1.85			disease_causing	28.3	p.S131Y	Passenger	no weights	damaging	Deleterious	Damaging
OVA_048_C3	PRIMARY	chr2	2.02E+08	2.02E+08	G	T	FAM126B	exonic	ynonymou	S131Y,ENSG	102	4	106	0.037736	39	1	40	0.025	0.06	Sub	0.74	1.8			disease_causing	28.3	p.S131Y	Passenger	no weights	damaging	Deleterious	Damaging
OVA_378_C2	PRIMARY	chr2	2.02E+08	2.02E+08	C	T	ALS2CR11	exonic	ynonymou	586N,ENSG	580	31	611	0.050736	73	0	73	0	0.18	Sub	0.56	2.59			polymorphism	0.129	p.S1783N	Passenger	SENGER/OT	benign	Neutral	Tolerated
OVA_048_M1	METASTASIS	chr1	2.05E+08	2.05E+08	T	G	NFASC	exonic	ynonymou	0539706:ex	519	69	588	0.117347	74	0	74	0	0.65	Sub	0.56	3.94			disease_causing	15.2	p.N1203K	Passenger	CANCER	damaging	Deleterious	Damaging
OVA_048_M2	METASTASIS	chr1	2.05E+08	2.05E+08	T	G	NFASC	exonic	ynonymou	0539706:ex	550	20	570	0.035088	74	0	74	0	0.24	Sub	0.4	3.87			disease_causing	15.2	p.N1203K	Passenger	CANCER	damaging	Deleterious	Damaging
OVA_048_M3	METASTASIS	chr1	2.05E+08	2.05E+08	T	G	NFASC	exonic	ynonymou	0539706:ex	457	115	572	0.201049	74	0	74	0	1	Sub	0.59	3.39			disease_causing	15.2	p.N1203K	Passenger	CANCER	damaging	Deleterious	Damaging
OVA_013_C2	PRIMARY	chr1	2.05E+08	2.05E+08	G	A	CNTN2	exonic	ynonymou	000331830	393	59	452	0.130531	63	0	63	0	0.56	Sub	0.87	3.05			disease_causing	29.3	p.G391D	Driver	SENGER/OT	damaging	Deleterious	Damaging
OVA_003_C2	PRIMARY	chr1	2.06E+08	2.06E+08	G	T	SLC41A1	exonic	ynonymou	000036713	362	72	434	0.165899	58	0	58	0	0.81	Sub	0.7	3.61			disease_causing	27.8	p.N148K	Passenger	SENGER/OT	damaging	Deleterious	Damaging
OVA_003_C1	PRIMARY	chr1	2.06E+08	2.06E+08	G	T	SLC41A1	exonic	ynonymou	000036713	393	7	400	0.0175	58	0	58	0	0.04	Sub	0.86	1.81			disease_causing	27.8	p.N148K	Passenger	SENGER/OT	damaging	Deleterious	Damaging
OVA_003_M1	METASTASIS	chr2	2.06E+08	2.06E+08	A	G	PARD3B	exonic	nonymous S	815R,ENSG	493	104	597	0.174204	61	0	61	0	0.97	Clonal	0.57	1.84			disease_causing	9.517		Passenger			Neutral	Tolerated
OVA_003_M2	METASTASIS	chr2	2.06E+08	2.06E+08	A	G	PARD3B	exonic	nonymous S	815R,ENSG	504	98	602	0.162791	61	0	61	0	0.87	Clonal	0.46	1.81			disease_causing	9.517		Passenger			Neutral	Tolerated
OVA_003_M3	METASTASIS	chr2	2.06E+08	2.06E+08	A	G	PARD3B	exonic	nonymous S	815R,ENSG	571	44	615	0.071545	61	0	61	0	1	Clonal	0.34	2.67			disease_causing	9.517		Passenger			Neutral	Tolerated
OVA_003_C1	PRIMARY	chr2	2.06E+08	2.06E+08	A	G	PARD3B	exonic	nonymous S	815R,ENSG	508	155	663	0.233786	61	0	61	0	0.78	Sub	0.86	1.81			disease_causing	9.517		Passenger			Neutral	Tolerated
OVA_003_C2	PRIMARY	chr2	2.06E+08	2.06E+08	A	G	PARD3B	exonic	nonymous S	815R,ENSG	535	128	663	0.193062	61	0	61	0	1	Sub	0.7	3.61			disease_causing	9.517		Passenger			Neutral	Tolerated
OVA_003_C3	PRIMARY	chr2	2.06E+08	2.06E+08	A	G	PARD3B	exonic	nonymous S	815R,ENSG	513	169	682	0.247801	61	0	61	0	0.85	Sub	0.82	1.81			disease_causing	9.517		Passenger			Neutral	Tolerated
OVA_365_M3	METASTASIS	chr2	2.08E+08	2.08E+08	A	C	CREB1	intronic	NA	NA	459	66	525	0.125714	60	0	60	0	0.43	Sub	0.82	1.89	Yes		disease_causing	21.2						
OVA_047_C3	Contralateral ovary	chr2	2.09E+08	2.09E+08	C	T	CRYGD	exonic	nonymous S	000026437	314	102	416	0.245192	71	0	71	0	1	Clonal	0.6	2.94			polymorphism	3.187		Passenger			Neutral	Tolerated
OVA_378_M1	METASTASIS	chr2	2.09E+08	2.09E+08	G	C	CRYGD	exonic	ynonymou	000026437	331	104	435	0.23908	88	0	88	0	0.9	Sub	0.72	2.57			disease_causing	23.9	p.C109W	Passenger	SENGER/OT	damaging	Deleterious	Damaging
OVA_378_M2	METASTASIS	chr2	2.09E+08	2.09E+08	G	C	CRYGD	exonic	ynonymou	000026437	332	109	441	0.247166	88	0	88	0	0.83	Sub	0.85	2.57			disease_causing	23.9	p.C109W	Passenger	SENGER/OT	damaging	Deleterious	Damaging
OVA_378_M3	METASTASIS	chr2	2.09E+08	2.09E+08	G	C	CRYGD	exonic	ynonymou	000026437	424	120	544	0.220588	88	0	88	0	0.88	Sub	0.67	2.57			disease_causing	23.9	p.C109W	Passenger	SENGER/OT	damaging	Deleterious	Damaging
OVA_378_C1	PRIMARY	chr2	2.09E+08	2.09E+08	G	C	CRYGD	exonic	ynonymou	000026437	423	94	517	0.181818	88	0	88	0	0.8	Sub	0.59	2.6			disease_causing	23.9	p.C109W	Passenger	SENGER/OT	damaging	Deleterious	Damaging
OVA_378_C2	PRIMARY	chr2	2.09E+08	2.09E+08	G	C	CRYGD	exonic	ynonymou	000026437	256	93	349	0.266476	88	0	88	0	0.94	Sub	0.56	2.59			disease_causing	23.9	p.C109W	Passenger	SENGER/OT	damaging	Deleterious	Damaging
OVA_378_C3	PRIMARY	chr2	2.09E+08	2.09E+08	G	C	CRYGD	exonic	ynonymou	000026437	243	64	307	0.208469	88	0	88	0	0.82	Sub	0.51	2.6			disease_causing	23.9	p.C109W	Passenger	SENGER/OT	damaging	Deleterious	Damaging
OVA_047_M1	METASTASIS	chr1	2.1E+08	2.1E+08	G	T	HSD11B1	RNA_intro	NA	NA	372	215	587	0.366269	37	0	37	0	1	Clonal	0.47	2.01			polymorphism	10.54						
OVA_047_M2	METASTASIS	chr1	2.1E+08	2.1E+08	G	T	HSD11B1	RNA_intro	NA	NA	406	197	603	0.3267	37	0	37	0	1	Clonal	0.44	2.01			polymorphism	10.54						
OVA_047_M3	METASTASIS	chr1	2.1E+08	2.1E+08	G	T	HSD11B1	RNA_intro	NA	NA	166	78	244	0.319672	37	0	37	0	1	Clonal	0.6	3.02			polymorphism	10.54						
OVA_013_M1	METASTASIS	chr1	2.12E+08	2.12E+08	C	T	INTS7	exonic	ynonymou	A444T,ENSG	505	132	637	0.207221	77	1	78	0.012821	1	Clonal	0.67	3.06			polymorphism	13.16	p.A444T	Passenger	SENGER/OT	benign	Neutral	Tolerated
OVA_013_C1	PRIMARY	chr1	2.12E+08	2.12E+08	C	T	INTS7	exonic	ynonymou	A444T,ENSG	541	132	673	0.196137	77	1	78	0.012821	0.94	Clonal	0.72	3.1			polymorphism	13.16	p.A444T	Passenger	SENGER/OT	benign	Neutral	Tolerated
OVA_013_C2	PRIMARY	chr1	2.12E+08	2.12E+08	C	T	INTS7	exonic	ynonymou	A444T,ENSG	517	132	649	0.20339	77	1	78	0.012821	0.87	Clonal	0.87	3.05			polymorphism	13.16	p.A444T	Passenger	SENGER/OT	benign	Neutral	Tolerated
OVA_013_C3	PRIMARY	chr1	2.12E+08	2.12E+08	C	T	INTS7	exonic	ynonymou	A444T,ENSG	526	157	683	0.229868	77	1	78	0.012821	0.96	Clonal	0.91	3.01			polymorphism	13.16	p.A444T	Passenger	SENGER/OT	benign	Neutral	Tolerated
OVA_013_C1	PRIMARY	chr1	2.12E+08	2.12E+08	G	C	DTL	exonic	ynonymou	G76R,ENSG	619	73	692	0.105491	50	0	50	0	0.5	Sub	0.72	3.1			disease_causing	29.7	p.G118R	Passenger	SENGER/OT	damaging	Deleterious	Damaging
OVA_047_M1	METASTASIS	chr1	2.13E+08	2.13E+08	C	G	RPS6KC1	exonic	ynonymou	890R,ENSG	413	274	687	0.398836	46	0	46	0	1	Clonal	0.47	2.01			disease_causing	28.7	p.P902R	Passenger	SENGER/OT	damaging	Deleterious	Damaging
OVA_047_M2	METASTASIS	chr1	2.13E+08	2.13E+08	C	G	RPS6KC1	exonic	ynonymou	890R,ENSG	459	225	684	0.328947	46	0	46	0	1	Clonal	0.44	2.01			disease_causing	28.7	p.P902R	Passenger	SENGER/OT	damaging	Deleterious	Damaging
OVA_047_M3	METASTASIS	chr1	2.13E+08	2.13E+08	C	G	RPS6KC1	exonic	ynonymou	890R,ENSG	252	153	405	0.377778	46	0	46	0	1	Clonal	0.6	3.02			disease_causing	28.7	p.P902R	Passenger	SENGER/OT	damaging	Deleterious	Damaging
OVA_048_M1	METASTASIS	chr2	2.16E+08	2.16E+08	TGTTCTGC/	-	ABCA12	exonic	ameshift de	04del,ENSG	717	276	993	0.277946	43	0	43		1	Clonal	0.56	3.94			polymorphism		1317_T132	Passenger			Deleterious	NA
OVA_048_M2	METASTASIS	chr2	2.16E+08	2.16E+08	TGTTCTGC/	-	ABCA12	exonic	ameshift de	04del,ENSG	902	256	1158	0.221071	43	0	43		1	Clonal	0.4	3.87			polymorphism		1317_T132	Passenger			Deleterious	NA
OVA_048_M3	METASTASIS	chr2	2.16E+08	2.16E+08	TGTTCTGC/	-	ABCA12	exonic	ameshift de	04del,ENSG	688	344	1032	0.333333	43	0	43		1	Clonal	0.59	3.39			polymorphism		1317_T132	Passenger			Deleterious	NA
OVA_365_C1	Contralateral ovary	chr2	2.17E+08	2.17E+08	C	G	38047	exonic	ynonymou	000027306	201	30	231	0.12987	101	0	101	0	0.62	Sub	0.42	1.83			disease_causing	27.7	p.K211N	Passenger	SENGER/OT	damaging	Deleterious	Damaging
OVA_013_C3	PRIMARY	chr2	2.19E+08	2.19E+08	C	T	TNS1	exonic	ynonymou	3M,ENSG0	315	23	338	0.068047	59	0	59	0	0.22	Sub	0.91	3.01			disease_causing	22.1				damaging	Neutral	Damaging
OVA_003_M1	METASTASIS	chr2	2.19E+08	2.19E+08	C	T	AAMP	intronic	NA	NA	104	33	137	0.240876	39	0	39	0	1	Sub	0.57	1.84			polymorphism	0.905						
OVA_003_M2	METASTASIS	chr2	2.19E+08	2.19E+08	C	T	AAMP	intronic	NA	NA	147	35	182	0.192308	39	0	39	0	1	Sub	0.46	1.81			polymorphism	0.905						
OVA_003_M3	METASTASIS	chr2	2.19E+08	2.19E+08	C	T	AAMP	intronic	NA	NA	137	11	148	0.074324	39	0	39	0	1	Sub	0.34	2.67			polymorphism	0.905						
OVA_003_C1	PRIMARY	chr2	2.19E+08	2.19E+08	C	T	AAMP	intronic	NA	NA	148	78	226	0.345133	39	0	39	0	1	Clonal	0.86	1.81			polymorphism	0.905						
OVA_003_C2	PRIMARY	chr2	2.19E+08	2.19E+08	C	T	AAMP	intronic	NA	NA	165	45	210	0.214286	39	0	39	0	1	Clonal	0.7	3.61			polymorphism	0.905						
OVA_003_C3	PRIMARY	chr2	2.19E+08	2.19E+08	C	T	AAMP	intronic	NA	NA	180	91	271	0.335793	39	0	39	0	1	Clonal	0.82	1.81			polymorphism	0.905						
OVA_047_M1	METASTASIS	chr2	2.19E+08	2.19E+08	GCTCGGGA	-	PLCD4	UTR5	NA	NA	279	90	369	0.243902	56	0	56		0.78	Sub	0.47	2.01										
OVA_047_M2	METASTASIS	chr2	2.19E+08	2.19E+08	GCTCGGGA	-	PLCD4	UTR5	NA	NA	378	100	478	0.209205	56	0	56		0.73	Sub	0.44	2.01										
OVA_047_M3	METASTASIS	chr2	2.19E+08	2.19E+08	GCTCGGGA	-	PLCD4	UTR5	NA	NA	182	52	234	0.222222	56	0	56		0.74	Sub	0.6	3.02										
OVA_378_C3	PRIMARY	chr1	2.2E+08	2.2E+08	G	C	SLC30A10	exonic	nonymous S	000036692	291	28	319	0.087774	90	0	90	0	0.43	Sub	0.51	2.6			disease_causing	17.65		Passenger			Neutral	Tolerated
OVA_003_M1	METASTASIS	chr2	2.2E+08	2.2E+08	C	G	GLB1L	intronic	NA	NA	454	115	569	0.202109	27	0	27	0	0.92	Clonal	0.57	1.84			polymorphism	10.65						
OVA_003_M2	METASTASIS	chr2	2.2E+08	2.2E+08	C	G	GLB1L	intronic	NA	NA	466	97	563	0.172291	27	0	27	0	0.92	Clonal	0.46	1.81			polymorphism	10.65						
OVA_003_M3	METASTASIS	chr2	2.2E+08	2.2E+08	C	G	GLB1L	intronic	NA	NA	543	46	589	0.078098	27	0	27	0	1	Clonal	0.34	2.67			polymorphism	10.65						
OVA_003_C1	PRIMARY	chr2	2.2E+08	2.2E+08	C	G	GLB1L	intronic	NA	NA	427	185	612	0.302288	27	0	27	0	1	Clonal	0.86	1.81			polymorphism	10.65						
OVA_003_C2	PRIMARY	chr2	2.2E+08	2.2E+08	C	G	GLB1L	intronic	NA	NA	436	155	591	0.262267	27	0	27	0	1	Clonal	0.7	3.61			polymorphism	10.65						
OVA_003_C3	PRIMARY	chr2	2.2E+08	2.2E+08	C	G	GLB1L	intronic	NA	NA	444	195	639	0.305164	27	0	27	0	1	Clonal	0.82	1.81			polymorphism	10.65						
OVA_003_M1	METASTASIS	chr2	2.2E+08	2.2E+08	CCCGGC	-	SPEG	intronic	NA	NA	76	51	127	0.401575	26	0	26		1	Clonal	0.57	1.84										
OVA_003_M2	METASTASIS	chr2	2.2E+08	2.2E+08	CCCGGC	-	SPEG	intronic	NA	NA	88	60	148	0.405405	26	0	26		1	Clonal	0.46	1.81										
OVA_003_M3	METASTASIS	chr2	2.2E+08	2.2E+08	CCCGGC	-	SPEG	intronic	NA	NA	167	22	189	0.116402	26	0	26		1	Clonal	0.34	2.67										
OVA_003_C1	PRIMARY	chr2	2.2E+08	2.2E+08	CCCGGC	-	SPEG	intronic	NA	NA	77	94	171	0.549708	26	0	26		1	Clonal	0.86	1.81										
OVA_003_C2	PRIMARY	chr2	2.2E+08	2.2E+08	CCCGGC	-	SPEG	intronic	NA	NA	73	95	168	0.565476	26	0	26		1	Clonal	0.7	3.61										
OVA_003_C3	PRIMARY	chr2	2.2E+08	2.2E+08	CCCGGC	-	SPEG	intronic	NA	NA	111	96	207	0.463768	26	0	26		1	Clonal	0.82	1.81										
OVA_013_M1	METASTASIS	chr2	2.2E+08	2.2E+08	G	T	INHA	exonic	nonymous S	000002437	72	76	148	0.513514	57	0	57	0	1	Clonal	0.67	3.06			polymorphism	6.439		Passenger			Neutral	Tolerated
OVA_013_C1	PRIMARY	chr2	2.2E+08	2.2E+08	G	T	INHA	exonic	nonymous S	000002437	70	75	145	0.517241	57	0	57	0	1	Clonal	0.72	3.1			polymorphism	6.439		Passenger			Neutral	Tolerated
OVA_013_C2	PRIMARY	chr2	2.2E+08	2.2E+08	G	T	INHA	exonic	nonymous S	000002437	56	57	113	0.504425	57	0	57	0	1	Clonal	0.87	3.05			polymorphism	6.439		Passenger			Neutral	Tolerated
OVA_013_C3	PRIMARY	chr2	2.2E+08	2.2E+08	G	T	INHA	exonic	nonymous S	000002437	65	91	156	0.583333	57	0	57	0	1	Clonal	0.91	3.01			polymorphism	6.439		Passenger			Neutral	Tolerated
OVA_013_C2	PRIMARY	chr1	2.21E+08	2.21E+08	G	T	36951	exonic	ynonymou	000036691	596	40	636	0.062893	72	0	72	0	0.27	Sub	0.87	3.05			polymorphism	23.1	p.Q299H	Passenger	SENGER/OT	benign	Neutral	Tolerated
OVA_365_M1	METASTASIS	chr1	2.28E+08	2.28E+08	G	T	OBSCN	exonic	ynonymou	000042212	146	9	155	0.058065	180	0	180	0	0.14	Sub	0.85	1.87			polymorphism	22.8	p.A445S	Passenger		benign	Neutral	Tolerated
OVA_003_C1	PRIMARY	chr2	2.29E+08	2.29E+08	G	T	SPHKAP	exonic	ynonymou	535R,ENSG	551	33	584	0.056507	56	0	56	0	0.19	Sub	0.86	1.81			disease_causing	24.7	p.S1535R	Passenger	SENGER/OT	damaging	Deleterious	Damaging
OVA_003_C2	PRIMARY	chr2	2.29E+08	2.29E+08	G	T	SPHKAP	exonic	ynonymou	535R,ENSG	558	44	602	0.07309	56	0	56	0	0.36	Sub	0.7	3.61			disease_causing	24.7	p.S1535R	Passenger	SENGER/OT	damaging	Deleterious	Damaging
OVA_003_C1	PRIMARY	chr2	2.3E+08	2.3E+08	T	C	DNER	exonic	ynonymou	000341772	419	40	459	0.087146	56	0	56	0	0.29	Sub	0.86	1.81			disease_causing	29.2	p.E719G	Passenger	SENGER/OT	damaging	Neutral	Damaging
OVA_003_C2	PRIMARY	chr2	2.3E+08	2.3E+08	T	C	DNER	exonic	ynonymou	000341772	489	17	506	0.033597	56	0	56	0	0.16	Sub	0.7	3.61			disease_causing	29.2	p.E719G	Passenger	SENGER/OT	damaging	Neutral	Damaging
OVA_047_C1	PRIMARY	chr1	2.31E+08	2.31E+08	G	A	ARV1	exonic	ynonymou	p.R6Q,ENSG	419	168	587	0.286201	39	0	39	0	0.96	Clonal	0.6	2.91			polymorphism	17.06	p.R6Q	Passenger	SENGER/OT	benign	Neutral	Tolerated
OVA_047_C2	PRIMARY	chr1	2.31E+08	2.31E+08	G	A	ARV1	exonic	ynonymou	p.R6Q,ENSG	391	167	558	0.299283	39	0	39	0	0.94	Clonal	0.64	2.76			polymorphism	17.06	p.R6Q	Passenger	SENGER/OT	benign	Neutral	Tolerated
OVA_047_C3	Contralateral ovary	chr1	2.31E+08	2.31E+08	G	A	ARV1	exonic	ynonymou	p.R6Q,ENSG	346	140	486	0.288066	39	0	39	0	0.97	Clonal	0.6	2.94			polymorphism	17.06	p.R6Q	Passenger	SENGER/OT	benign	Neutral	Tolerated
OVA_047_C1	PRIMARY	chr2	2.33E+08	2.33E+08	A	T	COPS7B	exonic	ynonymou	215N,ENSG	481	168	649	0.25886	52	0	52	0	1	Clonal	0.6	2.91			polymorphism	2.059	p.K220N	Passenger	SENGER/OT	benign	Neutral	Tolerated
OVA_047_C2	PRIMARY	chr2	2.33E+08	2.33E+08	A	T	COPS7B	exonic	ynonymou	215N,ENSG	487	128	615	0.20813	52	0	52	0	1	Clonal	0.64	2.76			polymorphism	2.059	p.K220N	Passenger	SENGER/OT	benign	Neutral	Tolerated
OVA_047_C3	Contralateral ovary	chr2	2.33E+08	2.33E+08	A	T	COPS7B	exonic	ynonymou	215N,ENSG	449	142	591	0.240271	52	0	52	0	1	Clonal	0.6	2.94			polymorphism	2.059	p.K220N	Passenger	SENGER/OT	benign	Neutral	Tolerated
OVA_378_M1	METASTASIS	chr1	2.33E+08	2.33E+08	ATTA	-	KIAA1804	intronic	NA	NA	904	287	1191	0.240974	48	0	48		0.91	Sub	0.72	2.57										
OVA_378_M2	METASTASIS	chr1	2.33E+08	2.33E+08	ATTA	-	KIAA1804	intronic	NA	NA	959	332	1291	0.257165	48	0	48		0.86	Sub	0.85	2.57										
OVA_378_M3	METASTASIS	chr1	2.33E+08	2.33E+08	ATTA	-	KIAA1804	intronic	NA	NA	1096	331	1427	0.231955	48	0	48		0.93	Sub	0.67	2.57										
OVA_378_C1	PRIMARY	chr1	2.33E+08	2.33E+08	ATTA	-	KIAA1804	intronic	NA	NA	989	332	1321	0.251325	48	0	48		1	Clonal	0.59	2.6										
OVA_378_C2	PRIMARY	chr1	2.33E+08	2.33E+08	ATTA	-	KIAA1804	intronic	NA	NA	946	298	1244	0.23955	48	0	48		1	Clonal	0.56	2.59										
OVA_378_C3	PRIMARY	chr1	2.33E+08	2.33E+08	ATTA	-	KIAA1804	intronic	NA	NA	1960	600	2560	0.234375					1	Clonal	0.51	2.6										
OVA_003_M1	METASTASIS	chr2	2.34E+08	2.34E+08	G	C	INPP5D	exonic	ynonymou	R54T,ENSG	474	134	608	0.220395	50	0	50	0	1	Sub	0.57	1.84			disease_causing	25.5	p.R54T	Passenger		damaging	Deleterious	Damaging
OVA_003_M2	METASTASIS	chr2	2.34E+08	2.34E+08	G	C	INPP5D	exonic	ynonymou	R54T,ENSG	527	98	625	0.1568	50	0	50	0	0.84	Sub	0.46	1.81			disease_causing	25.5	p.R54T	Passenger		damaging	Deleterious	Damaging

Sample	Type	Chr	Start	End	Ref	Alt	Gene	unc.ensGer	icFunc.ens	hange.ens(	t_ref	t_alt	t_total	t_VAF	n_ref	n_alt	n_total	normal_VA	ncer_cell_f	on-based C	purity	ploidy	Cancer Gene	Driver Gene	Mutation Taster	ADD(PHRE	otein_chan	CHASM	THMM_Pr	OLYPHEN-	REDICTION	ICTION (cutoff=0.05)
OVA_003_M3	METASTASIS	chr2	2.34E+08	2.34E+08	G	C	INPP5D	exonic	ynonymou	.R54T,ENSG	611	29	640	0.045313	50	0	50	0	0.61	Sub	0.34	2.67			disease_causing	25.5	p.R54T	Passenger		damaging	Deleterious	Damaging
OVA_003_C1	PRIMARY	chr2	2.34E+08	2.34E+08	G	C	INPP5D	exonic	ynonymou	.R54T,ENSG	465	190	655	0.290076	50	0	50	0	0.96	Clonal	0.86	1.81			disease_causing	25.5	p.R54T	Passenger		damaging	Deleterious	Damaging
OVA_003_C2	PRIMARY	chr2	2.34E+08	2.34E+08	G	C	INPP5D	exonic	ynonymou	.R54T,ENSG	478	147	625	0.2352	50	0	50	0	1	Clonal	0.7	3.61			disease_causing	25.5	p.R54T	Passenger		damaging	Deleterious	Damaging
OVA_003_C3	PRIMARY	chr2	2.34E+08	2.34E+08	G	C	INPP5D	exonic	ynonymou	.R54T,ENSG	471	196	667	0.293853	50	0	50	0	1	Clonal	0.82	1.81			disease_causing	25.5	p.R54T	Passenger		damaging	Deleterious	Damaging
OVA_047_C1	PRIMARY	chr2	2.34E+08	2.34E+08	A	G	USP40	exonic	ynonymou	000045096(	514	161	675	0.238519	77	0	77	0	1	Clonal	0.6	2.91			disease_causing	26.1	p.L658P	Passenger	SENGER/OT	damaging	Deleterious	Damaging
OVA_047_C2	PRIMARY	chr2	2.34E+08	2.34E+08	A	G	USP40	exonic	ynonymou	000045096(	546	128	674	0.189911	77	0	77	0	0.97	Clonal	0.64	2.76			disease_causing	26.1	p.L658P	Passenger	SENGER/OT	damaging	Deleterious	Damaging
OVA_047_C3	Contralateral ovary	chr2	2.34E+08	2.34E+08	A	G	USP40	exonic	ynonymou	000045096(	496	141	637	0.22135	77	0	77	0	0.96	Clonal	0.6	2.94			disease_causing	26.1	p.L658P	Passenger	SENGER/OT	damaging	Deleterious	Damaging
OVA_378_M1	METASTASIS	chr1	2.37E+08	2.37E+08	C	T	HEATR1	intronic	NA	NA	411	148	559	0.264758	35	0	35	0	1	Clonal	0.72	2.57			polymorphism	9.195						
OVA_378_M2	METASTASIS	chr1	2.37E+08	2.37E+08	C	T	HEATR1	intronic	NA	NA	402	159	561	0.283422	35	0	35	0	0.95	Clonal	0.85	2.57			polymorphism	9.195						
OVA_378_M3	METASTASIS	chr1	2.37E+08	2.37E+08	C	T	HEATR1	intronic	NA	NA	470	160	630	0.253968	35	0	35	0	1	Clonal	0.67	2.57			polymorphism	9.195						
OVA_378_C1	PRIMARY	chr1	2.37E+08	2.37E+08	C	T	HEATR1	intronic	NA	NA	469	149	618	0.2411	35	0	35	0	1	Clonal	0.59	2.6			polymorphism	9.195						
OVA_378_C2	PRIMARY	chr1	2.37E+08	2.37E+08	C	T	HEATR1	intronic	NA	NA	438	112	550	0.203636	35	0	35	0	0.93	Clonal	0.56	2.59			polymorphism	9.195						
OVA_378_C3	PRIMARY	chr1	2.37E+08	2.37E+08	C	T	HEATR1	intronic	NA	NA	437	122	559	0.218247	35	0	35	0	1	Clonal	0.51	2.6			polymorphism	9.195						
OVA_048_M1	METASTASIS	chr1	2.38E+08	2.38E+08	AATA	-	RYR2	exonic	neshift dele	0366574:ex	385	141	526	0.268061	46	0	46		1	Clonal	0.56	3.94					p.N3308Kfs	Passenger			NA	NA
OVA_378_M1	METASTASIS	chr1	2.38E+08	2.38E+08	C	T	RYR2	exonic	ynonymou	000366574:	457	123	580	0.212069	57	0	57	0	0.8	Sub	0.72	2.57			disease_causing	32	p.R3454C	Passenger		damaging	Deleterious	Damaging
OVA_378_M2	METASTASIS	chr1	2.38E+08	2.38E+08	C	T	RYR2	exonic	ynonymou	000366574:	407	151	558	0.270609	57	0	57	0	0.9	Sub	0.85	2.57			disease_causing	32	p.R3454C	Passenger		damaging	Deleterious	Damaging
OVA_378_M3	METASTASIS	chr1	2.38E+08	2.38E+08	C	T	RYR2	exonic	ynonymou	000366574:	501	155	656	0.23628	57	0	57	0	0.94	Sub	0.67	2.57			disease_causing	32	p.R3454C	Passenger		damaging	Deleterious	Damaging
OVA_378_C1	PRIMARY	chr1	2.38E+08	2.38E+08	C	T	RYR2	exonic	ynonymou	000366574:	486	148	634	0.233438	57	0	57	0	1	Sub	0.59	2.6			disease_causing	32	p.R3454C	Passenger		damaging	Deleterious	Damaging
OVA_378_C2	PRIMARY	chr1	2.38E+08	2.38E+08	C	T	RYR2	exonic	ynonymou	000366574:	444	139	583	0.238422	57	0	57	0	1	Sub	0.56	2.59			disease_causing	32	p.R3454C	Passenger		damaging	Deleterious	Damaging
OVA_378_C3	PRIMARY	chr1	2.38E+08	2.38E+08	C	T	RYR2	exonic	ynonymou	000366574:	476	98	574	0.170732	57	0	57	0	0.84	Sub	0.51	2.6			disease_causing	32	p.R3454C	Passenger		damaging	Deleterious	Damaging
OVA_048_C3	PRIMARY	chr1	2.38E+08	2.38E+08	G	A	RYR2	exonic	ynonymou	0366574:ex	394	119	513	0.231969	39	0	39	0	0.86	Clonal	0.74	1.8			disease_causing	33	p.R4790Q	Passenger		damaging	Deleterious	Damaging
OVA_047_C3	Contralateral ovary	chr2	2.38E+08	2.38E+08	C	A	COL6A3	exonic	ynonymou	000392004	361	80	441	0.181406	74	1	75	0.013333	0.79	Sub	0.6	2.94			disease_causing	24.9	p.V305F	Passenger	SENGER/OT	damaging	Neutral	Damaging
OVA_013_M1	METASTASIS	chr2	2.39E+08	2.39E+08	G	A	ESPNL	intronic	NA	NA	106	33	139	0.23741	16	0	16	0	0.95	Clonal	0.67	3.06			polymorphism	7.702						
OVA_013_C1	PRIMARY	chr2	2.39E+08	2.39E+08	G	A	ESPNL	intronic	NA	NA	108	37	145	0.255172	16	0	16	0	0.96	Sub	0.72	3.1			polymorphism	7.702						
OVA_013_C2	PRIMARY	chr2	2.39E+08	2.39E+08	G	A	ESPNL	intronic	NA	NA	96	43	139	0.309353	16	0	16	0	0.71	Sub	0.87	3.05			polymorphism	7.702						
OVA_013_C3	PRIMARY	chr2	2.39E+08	2.39E+08	G	A	ESPNL	intronic	NA	NA	117	41	158	0.259494	16	0	16	0	0.83	Sub	0.91	3.01			polymorphism	7.702						
OVA_047_C1	PRIMARY	chr1	2.41E+08	2.41E+08	T	A	RGS7	exonic	ynonymou	)1W,ENSG(	408	220	628	0.350318	15	0	15	0	1	Clonal	0.6	2.91	Yes	Yes	disease_causing	26.5	p.R201W	Driver	SENGER/OT	damaging	Deleterious	Damaging
OVA_047_C2	PRIMARY	chr1	2.41E+08	2.41E+08	T	A	RGS7	exonic	ynonymou	)1W,ENSG(	438	191	629	0.303657	15	0	15	0	0.95	Clonal	0.64	2.76	Yes	Yes	disease_causing	26.5	p.R201W	Driver	SENGER/OT	damaging	Deleterious	Damaging
OVA_047_C3	Contralateral ovary	chr1	2.41E+08	2.41E+08	T	A	RGS7	exonic	ynonymou	)1W,ENSG(	440	173	613	0.282219	15	0	15	0	0.95	Clonal	0.6	2.94	Yes	Yes	disease_causing	26.5	p.R201W	Driver	SENGER/OT	damaging	Deleterious	Damaging
OVA_047_C2	PRIMARY	chr1	2.42E+08	2.42E+08	G	C	EXO1	intronic	NA	NA	439	163	602	0.270764	10	0	10	0	0.85	Sub	0.64	2.76			polymorphism	0.817						
OVA_048_C1	PRIMARY	chr2	2.42E+08	2.42E+08	G	T	ANO7	intronic	NA	NA	36	12	48	0.25	21	0	21	0	1	Sub	0.67	1.82			polymorphism	5.332						
OVA_048_C2	PRIMARY	chr2	2.42E+08	2.42E+08	G	T	ANO7	intronic	NA	NA	62	37	99	0.373737	21	0	21	0	1	Sub	0.72	1.85			polymorphism	5.332						
OVA_048_C3	PRIMARY	chr2	2.42E+08	2.42E+08	G	T	ANO7	intronic	NA	NA	56	12	68	0.176471	21	0	21	0	0.48	Sub	0.74	1.8			polymorphism	5.332						
OVA_013_M1	METASTASIS	chr2	2.42E+08	2.42E+08	C	T	HDLBP	intronic	NA	NA	409	161	570	0.282456	12	0	12	0	1	Clonal	0.67	3.06			polymorphism	0.469						
OVA_013_C3	PRIMARY	chr2	2.42E+08	2.42E+08	C	T	HDLBP	intronic	NA	NA	418	167	585	0.28547	12	0	12	0	0.91	Sub	0.91	3.01			polymorphism	0.469						
OVA_013_C2	PRIMARY	chr2	2.42E+08	2.42E+08	C	T	HDLBP	intronic	NA	NA	335	213	548	0.388686	12	0	12	0	0.89	Sub	0.87	3.05			polymorphism	0.469						

**Supplementary Table S3: Copy number variations predicted by using Facets algorithm**											
##Abbreviations of column headers											
##Sample: Unique tissue sample identifier											
##Tissue type: Type of tissue, e.g Primary, metastasis											
##chrom: Chromosome											
##nhet: number of heterozygous markers											
##num.mark: Total markers											
##start: hg19 start coordinate											
##end: hg19 end coordinate											
##Classification: classification of copynumber variation											
##Purity: Tumor purity											
##Ploidy: Tumor ploidy											
##cyto: Cytoband											
##Size_Mb: copynumber variation size in Mb											
**Sample**	**Tissue type**	**chrom**	**num.mark**	**nhet**	**start**	**end**	**Classificati**	**Purity**	**Ploidy**	**Cyto**	**Size_Mb**
OVA_378_M1	Metastasis	chr11	4097	384	134550	60230531	Loss	0.753235	2.555202	p11.11-q12.2	60.09598
OVA_378_M2	Metastasis	chr11	4094	384	134550	60231260	Loss	0.840932	2.531102	p11.11-q12.2	60.09671
OVA_378_M3	Metastasis	chr11	4087	383	134550	60231260	Loss	0.692693	2.569487	p11.11-q12.2	60.09671
OVA_013_M1	Metastasis	chr11	902	76	44640477	60786711	Loss	0.621947	2.883383	p11.11-q12.2	16.14623
OVA_013_C1	Primary	chr11	894	73	44919327	60714276	Loss	0.701164	3.125184	p11.11-q12.2	15.79495
OVA_013_C3	Primary	chr11	895	74	44919331	60718792	Loss	0.75175	3.001491	p11.11-q12.2	15.79946
OVA_013_C2	Primary	chr11	894	74	44919366	60718792	Loss	0.755564	3.14838	p11.11-q12.2	15.79943
OVA_048_C3	Primary	chr11	432	50	51459044	61076509	Gain	0.715645	1.786574	p11.11-q12.2	9.617465
OVA_047_C1	Primary	chr11	3232	331	47447924	76506888	Loss	0.576016	2.862818	p11.11-q13.5	29.05896
OVA_047_CO	Contralateral ovary	chr11	3238	330	47447924	76798961	Loss	0.55613	2.930893	p11.11-q13.5	29.35104
OVA_047_M3	Metastasis	chr11	6010	569	2325239	85397109	Loss	0.605088	2.815208	p11.11-q14.1	83.07187
OVA_047_M1	Metastasis	chr11	5957	567	2797237	85409211	Loss	0.624347	2.86771	p11.11-q14.1	82.61197
OVA_047_M2	Metastasis	chr11	5953	567	2797237	85397109	Loss	0.599725	2.678568	p11.11-q14.1	82.59987
OVA_378_C1	Primary	chr11	9918	895	134550	1.35E+08	Loss	0.616455	2.593914	p11.11-q25	134.7221
OVA_378_C2	Primary	chr11	9943	895	134550	1.35E+08	Loss	0.607575	2.601752	p11.11-q25	134.722
OVA_003_C2	Primary	chr11	7029	626	26725149	1.35E+08	Del	0.709607	3.665802	p11.11-q25	108.1314
OVA_048_M2	Metastasis	chr11	4719	463	49372839	1.35E+08	Del	0.405007	3.72502	p11.11-q25	85.48382
OVA_048_M1	Metastasis	chr11	4707	462	50003627	1.35E+08	Del	0.625824	3.962486	p11.11-q25	84.85303
OVA_048_M3	Metastasis	chr11	4706	462	51459044	1.35E+08	Del	0.59385	3.387584	p11.11-q25	83.39762
OVA_047_C2	Primary	chr11	668	68	34533002	51411832	Del	0.575254	2.829045	p11.12-p13	16.87883
OVA_048_M1	Metastasis	chr11	2391	287	193190	49898083	Del	0.625824	3.962486	p11.12-p15.5	49.70489
OVA_048_M2	Metastasis	chr11	2053	235	1272954	49329552	Del	0.405007	3.72502	p11.12-p15.5	48.0566
OVA_048_M3	Metastasis	chr11	2068	236	1272954	50003627	Del	0.59385	3.387584	p11.12-p15.5	48.73067
OVA_047_C2	Primary	chr2	25	2	91944085	92321754	Amp	0.575254	2.829045	p11.1-p11.1	0.377669
OVA_048_M1	Metastasis	chr12	1630	160	90681	34179746	Del	0.625824	3.962486	p11.1-p13.33	34.08907
OVA_048_M2	Metastasis	chr12	1630	160	90681	34179648	Del	0.405007	3.72502	p11.1-p13.33	34.08897
OVA_047_C2	Primary	chr10	1400	181	93387	38654829	Loss	0.575254	2.829045	p11.1-p15.3	38.56144
OVA_378_C2	Primary	chr3	4632	361	239347	89521725	Loss	0.607575	2.601752	p11.1-p26.3	89.28238
OVA_378_C1	Primary	chr3	4618	361	239350	89521663	Loss	0.616455	2.593914	p11.1-p26.3	89.28231
OVA_378_M1	Metastasis	chr3	4634	361	239350	89521663	Loss	0.753235	2.555202	p11.1-p26.3	89.28231
OVA_378_M2	Metastasis	chr3	4630	361	239350	89521725	Loss	0.840932	2.531102	p11.1-p26.3	89.28238
OVA_378_M3	Metastasis	chr3	4614	357	239350	89521725	Loss	0.692693	2.569487	p11.1-p26.3	89.28238
OVA_378_C3	Primary	chr3	4633	361	239588	89521663	Loss	0.517221	2.617793	p11.1-p26.3	89.28208
OVA_378_M1	Metastasis	chr17	3026	313	6157	25308930	Loss	0.753235	2.555202	p11.1-q11.1	25.30277
OVA_378_M2	Metastasis	chr17	3022	312	6157	25290357	Loss	0.840932	2.531102	p11.1-q11.1	25.2842
OVA_378_M3	Metastasis	chr17	3019	312	6157	25309292	Loss	0.692693	2.569487	p11.1-q11.1	25.30314
OVA_047_M1	Metastasis	chr18	655	55	12863	18534806	Loss	0.624347	2.86771	p11.1-q11.1	18.52194
OVA_378_C3	Primary	chr18	718	59	12863	18520278	Loss	0.517221	2.617793	p11.1-q11.1	18.50742
OVA_047_M2	Metastasis	chr18	657	55	12905	18534806	Loss	0.599725	2.678568	p11.1-q11.1	18.5219
OVA_047_M3	Metastasis	chr18	656	55	12905	18534806	Loss	0.605088	2.815208	p11.1-q11.1	18.5219
OVA_047_C1	Primary	chr8	241	17	39018507	47759746	Loss	0.576016	2.862818	p11.1-q11.1	8.741239
OVA_047_C1	Primary	chr3	754	78	53886912	93699334	Loss	0.576016	2.862818	p11.1-q11.1	39.81242
OVA_047_C1	Primary	chr18	878	68	12863	23603751	Del	0.576016	2.862818	p11.1-q11.2	23.59089
OVA_047_CO	Contralateral ovary	chr18	834	66	658025	22807642	Del	0.55613	2.930893	p11.1-q11.2	22.14962
OVA_047_C2	Primary	chr17	1235	114	9143141	27085808	Del	0.575254	2.829045	p11.1-q11.2	17.94267
OVA_047_CO	Contralateral ovary	chr17	1228	113	9143141	27086200	Del	0.55613	2.930893	p11.1-q11.2	17.94306
OVA_013_C3	Primary	chr21	33	6	9826872	14982614	Loss	0.75175	3.001491	p11.1-q11.2	5.155742
OVA_047_C1	Primary	chr17	475	44	18483578	27033752	Del	0.576016	2.862818	p11.1-q11.2	8.550174
OVA_047_CO	Contralateral ovary	chr16	315	25	30975492	46394927	Loss	0.55613	2.930893	p11.1-q11.2	15.41944
OVA_003_C2	Primary	chr3	652	60	57484251	96713004	Del	0.709607	3.665802	p11.1-q11.2	39.22875
OVA_047_CO	Contralateral ovary	chr2	378	35	90458455	98277049	Loss	0.55613	2.930893	p11.1-q11.2	7.818594
OVA_048_C3	Primary	chr20	1076	112	76915	31827643	Gain	0.715645	1.786574	p11.1-q11.21	31.75073

Sample	Tissue type	chrom	num.mark	nhet	start	end	Classificati	Purity	Ploidy	Cyto	Size_Mb
OVA_048_M1	Metastasis	chr20	1072	112	76915	31761919	Del	0.625824	3.962486	p11.1-q11.21	31.685
OVA_048_M3	Metastasis	chr20	1072	112	76920	31761919	Del	0.59385	3.387584	p11.1-q11.21	31.685
OVA_048_M2	Metastasis	chr20	1078	112	76962	31878815	Del	0.405007	3.72502	p11.1-q11.21	31.80185
OVA_013_C1	Primary	chr10	1394	164	93257	43088670	Loss	0.701164	3.125184	p11.1-q11.21	42.99541
OVA_013_C2	Primary	chr10	1397	164	93257	43090003	Loss	0.755564	3.14838	p11.1-q11.21	42.99675
OVA_013_C3	Primary	chr10	1395	164	93257	43078228	Loss	0.75175	3.001491	p11.1-q11.21	42.98497
OVA_047_C1	Primary	chr10	1527	189	93257	44345134	Loss	0.576016	2.862818	p11.1-q11.21	44.25188
OVA_047_CO	Contralateral ovary	chr10	1527	189	93257	44345134	Loss	0.55613	2.930893	p11.1-q11.21	44.25188
OVA_378_C3	Primary	chr7	2763	321	193222	62694427	Loss	0.517221	2.617793	p11.1-q11.21	62.50121
OVA_378_C1	Primary	chr7	2796	322	193245	63720665	Loss	0.616455	2.593914	p11.1-q11.21	63.52742
OVA_378_C2	Primary	chr7	2785	322	193245	63250377	Loss	0.607575	2.601752	p11.1-q11.21	63.05713
OVA_048_M2	Metastasis	chr8	1344	105	11565708	49832861	Del	0.405007	3.72502	p11.1-q11.21	38.26715
OVA_047_M1	Metastasis	chr10	953	103	13386754	43600689	Loss	0.624347	2.86771	p11.1-q11.21	30.21394
OVA_047_M3	Metastasis	chr10	995	105	13386846	44345175	Loss	0.605088	2.815208	p11.1-q11.21	30.95833
OVA_047_M2	Metastasis	chr10	993	105	13481427	44345175	Loss	0.599725	2.678568	p11.1-q11.21	30.86375
OVA_047_C2	Primary	chr10	51	5	38658075	42600238	Amp	0.575254	2.829045	p11.1-q11.21	3.942163
OVA_013_M1	Metastasis	chr10	1626	183	93257	49917753	Loss	0.621947	2.883383	p11.1-q11.23	49.8245
OVA_013_C2	Primary	chr7	2478	238	193222	72418992	Del	0.755564	3.14838	p11.1-q11.23	72.22577
OVA_013_C3	Primary	chr7	2476	237	193222	72409197	Del	0.75175	3.001491	p11.1-q11.23	72.21598
OVA_013_C1	Primary	chr7	2486	238	193245	72419548	Del	0.701164	3.125184	p11.1-q11.23	72.2263
OVA_047_M3	Metastasis	chr7	2309	208	6630103	75034255	Loss	0.605088	2.815208	p11.1-q11.23	68.40415
OVA_003_C2	Primary	chr7	1643	161	25163744	73021328	Del	0.709607	3.665802	p11.1-q11.23	47.85758
OVA_013_M1	Metastasis	chr7	1289	130	27797660	72399011	Loss	0.621947	2.883383	p11.1-q11.23	44.60135
OVA_047_C2	Primary	chr7	932	87	45717711	76631590	Loss	0.575254	2.829045	p11.1-q11.23	30.91388
OVA_047_M1	Metastasis	chr7	397	34	55713506	73280020	Loss	0.624347	2.86771	p11.1-q11.23	17.56651
OVA_047_M2	Metastasis	chr7	514	36	55874726	75068514	Loss	0.599725	2.678568	p11.1-q11.23	19.19379
OVA_048_M3	Metastasis	chr12	1637	160	90681	39087609	Del	0.59385	3.387584	p11.1-q12	38.99693
OVA_047_C2	Primary	chr12	1781	152	5687097	40110582	Loss	0.575254	2.829045	p11.1-q12	34.42349
OVA_047_CO	Contralateral ovary	chr12	1768	151	5961102	40114744	Loss	0.55613	2.930893	p11.1-q12	34.15364
OVA_047_C1	Primary	chr12	1733	147	6161904	40114744	Loss	0.576016	2.862818	p11.1-q12	33.95284
OVA_047_M2	Metastasis	chr12	1733	147	6161954	40110582	Loss	0.599725	2.678568	p11.1-q12	33.94863
OVA_047_M3	Metastasis	chr12	1733	147	6161954	40158511	Loss	0.605088	2.815208	p11.1-q12	33.99656
OVA_013_C2	Primary	chr12	647	74	26208428	44162013	Loss	0.755564	3.14838	p11.1-q12	17.95359
OVA_047_M1	Metastasis	chr12	483	31	27800667	40951902	Del	0.624347	2.86771	p11.1-q12	13.15124
OVA_048_M1	Metastasis	chr18	776	72	47632	28654818	Del	0.625824	3.962486	p11.1-q12.1	28.60719
OVA_048_M1	Metastasis	chr8	1683	142	182793	56725718	Del	0.625824	3.962486	p11.1-q12.1	56.54293
OVA_013_M1	Metastasis	chr16	2480	172	2259393	50813629	Loss	0.621947	2.883383	p11.1-q12.1	48.55424
OVA_013_C3	Primary	chr18	658	60	9195598	30050356	Del	0.75175	3.001491	p11.1-q12.1	20.85476
OVA_013_C2	Primary	chr18	653	60	9195607	30050356	Del	0.755564	3.14838	p11.1-q12.1	20.85475
OVA_013_C1	Primary	chr18	657	60	9221883	30050356	Del	0.701164	3.125184	p11.1-q12.1	20.82847
OVA_047_M1	Metastasis	chr16	1316	99	22486554	51173589	Loss	0.624347	2.86771	p11.1-q12.1	28.68704
OVA_047_M2	Metastasis	chr16	1312	99	22545285	51172677	Loss	0.599725	2.678568	p11.1-q12.1	28.62739
OVA_047_M3	Metastasis	chr16	1301	99	23096952	51174255	Loss	0.605088	2.815208	p11.1-q12.1	28.0773
OVA_048_M3	Metastasis	chr8	859	47	25042515	56860153	Del	0.59385	3.387584	p11.1-q12.1	31.81764
OVA_048_C3	Primary	chr8	594	38	30694758	56986758	Gain	0.715645	1.786574	p11.1-q12.1	26.292
OVA_003_C2	Primary	chr18	1206	103	47524	33077673	Del	0.709607	3.665802	p11.1-q12.2	33.03015
OVA_003_C1	Primary	chr18	1207	103	47632	33077971	Gain	0.852911	1.812048	p11.1-q12.2	33.03034
OVA_003_C2	Primary	chr16	3887	238	85697	55730124	Del	0.709607	3.665802	p11.1-q12.2	55.64443
OVA_013_C1	Primary	chr16	3470	287	62965	57113168	Del	0.701164	3.125184	p11.1-q13	57.0502
OVA_013_C2	Primary	chr16	3467	285	62965	57113168	Del	0.755564	3.14838	p11.1-q13	57.0502
OVA_013_C3	Primary	chr16	2812	198	2226243	57113168	Loss	0.75175	3.001491	p11.1-q13	54.88693
OVA_048_M3	Metastasis	chr6	74	2	57246847	70042918	Del	0.59385	3.387584	p11.1-q13	12.79607
OVA_048_C3	Primary	chr6	72	2	57393203	70411770	Gain	0.715645	1.786574	p11.1-q13	13.01857
OVA_048_M1	Metastasis	chr6	71	2	57393203	70042918	Del	0.625824	3.962486	p11.1-q13	12.64972
OVA_048_M2	Metastasis	chr6	72	2	57393203	70411770	Del	0.405007	3.72502	p11.1-q13	13.01857
OVA_013_C1	Primary	chr12	771	83	26208428	47471439	Loss	0.701164	3.125184	p11.1-q13.11	21.26301
OVA_047_C2	Primary	chr3	2845	224	42226151	1.05E+08	Loss	0.575254	2.829045	p11.1-q13.11	63.01275
OVA_047_CO	Contralateral ovary	chr3	1086	94	53916081	1.06E+08	Loss	0.55613	2.930893	p11.1-q13.11	51.67199
OVA_003_C2	Primary	chr20	1982	135	4228781	44144021	Del	0.709607	3.665802	p11.1-q13.12	39.91524
OVA_013_C1	Primary	chr20	2525	206	76700	49212798	Del	0.701164	3.125184	p11.1-q13.13	49.1361
OVA_013_C3	Primary	chr20	2405	200	76920	47538388	Loss	0.75175	3.001491	p11.1-q13.13	47.46147
OVA_013_C2	Primary	chr20	2432	203	76934	47843146	Del	0.755564	3.14838	p11.1-q13.13	47.76621
OVA_013_M1	Metastasis	chr20	2404	200	76934	47538388	Loss	0.621947	2.883383	p11.1-q13.13	47.46145
OVA_003_C2	Primary	chr12	2878	250	6428669	52470983	Del	0.709607	3.665802	p11.1-q13.13	46.04231
OVA_003_C2	Primary	chr8	2606	203	182785	68965500	Del	0.709607	3.665802	p11.1-q13.2	68.78272
OVA_047_M1	Metastasis	chr3	1076	88	58318751	1.12E+08	Loss	0.624347	2.86771	p11.1-q13.2	53.74786

Sample	Tissue type	chrom	num.mark	nhet	start	end	Classificati	Purity	Ploidy	Cyto	Size_Mb
OVA_047_M3	Metastasis	chr3	1068	86	58383275	1.12E+08	Loss	0.605088	2.815208	p11.1-q13.2	53.6133
OVA_013_M1	Metastasis	chr6	943	74	44355399	79595097	Loss	0.621947	2.883383	p11.1-q14.1	35.2397
OVA_013_C1	Primary	chr6	940	73	44355414	79577544	Del	0.701164	3.125184	p11.1-q14.1	35.22213
OVA_013_C3	Primary	chr6	518	29	55381369	76608251	Del	0.75175	3.001491	p11.1-q14.1	21.22688
OVA_013_C2	Primary	chr6	512	29	55431435	76608251	Del	0.755564	3.14838	p11.1-q14.1	21.17682
OVA_013_C2	Primary	chr2	3125	207	42472748	1.28E+08	Del	0.755564	3.14838	p11.1-q14.3	85.34549
OVA_013_C1	Primary	chr2	3127	208	42472842	1.28E+08	Del	0.701164	3.125184	p11.1-q14.3	85.34536
OVA_013_C3	Primary	chr2	3126	208	42472842	1.28E+08	Loss	0.75175	3.001491	p11.1-q14.3	85.34386
OVA_013_M1	Metastasis	chr2	2389	168	64069497	1.28E+08	Loss	0.621947	2.883383	p11.1-q14.3	63.74878
OVA_003_C2	Primary	chr6	2672	177	38843426	1.11E+08	Del	0.709607	3.665802	p11.1-q21	72.14849
OVA_047_M2	Metastasis	chr6	2512	173	41019978	1.12E+08	Loss	0.599725	2.678568	p11.1-q21	70.56767
OVA_047_M3	Metastasis	chr6	2463	172	41658889	1.12E+08	Loss	0.605088	2.815208	p11.1-q21	69.96228
OVA_048_M3	Metastasis	chr18	1136	101	47524	44656650	Del	0.59385	3.387584	p11.1-q21.1	44.60913
OVA_048_M2	Metastasis	chr18	1152	101	47615	45567131	Del	0.405007	3.72502	p11.1-q21.1	45.51952
OVA_048_C3	Primary	chr21	117	16	9826193	22849672	Gain	0.715645	1.786574	p11.1-q21.1	13.02348
OVA_048_M3	Metastasis	chr2	1910	172	70679005	1.31E+08	Del	0.59385	3.387584	p11.1-q21.1	60.45313
OVA_048_M2	Metastasis	chr9	1289	103	121432	71080033	Del	0.405007	3.72502	p11.1-q21.11	70.9586
OVA_048_M3	Metastasis	chr9	1289	103	121432	71080033	Loss	0.59385	3.387584	p11.1-q21.11	70.9586
OVA_047_C1	Primary	chr7	1216	108	45717711	87043230	Loss	0.576016	2.862818	p11.1-q21.12	41.32552
OVA_048_M2	Metastasis	chr17	3549	340	5994	40028491	Del	0.405007	3.72502	p11.1-q21.2	40.0225
OVA_048_M3	Metastasis	chr17	3358	309	5994	39017995	Del	0.59385	3.387584	p11.1-q21.2	39.012
OVA_048_C2	Primary	chr17	3374	314	5996	39140138	Loss	0.736255	1.845483	p11.1-q21.2	39.13414
OVA_048_M1	Metastasis	chr1	6364	560	2082303	1.5E+08	Del	0.625824	3.962486	p11.1-q21.2	148.1982
OVA_048_M1	Metastasis	chr17	1258	96	19349980	39041052	Del	0.625824	3.962486	p11.1-q21.2	19.69107
OVA_048_M2	Metastasis	chr10	525	45	35858030	62551890	Del	0.405007	3.72502	p11.1-q21.2	26.69386
OVA_047_M1	Metastasis	chr8	1280	68	37422150	85618214	Loss	0.624347	2.86771	p11.1-q21.2	48.19606
OVA_048_M2	Metastasis	chr1	6376	560	2076055	1.5E+08	Del	0.405007	3.72502	p11.1-q21.3	148.3427
OVA_013_C2	Primary	chr21	164	21	9826872	26969703	Loss	0.755564	3.14838	p11.1-q21.3	17.14283
OVA_048_M3	Metastasis	chr1	5536	474	13910618	1.5E+08	Del	0.59385	3.387584	p11.1-q21.3	136.5082
OVA_013_C1	Primary	chr8	1149	42	38987070	89128907	Loss	0.701164	3.125184	p11.1-q21.3	50.14184
OVA_003_C2	Primary	chr1	2049	147	94528363	1.52E+08	Del	0.709607	3.665802	p11.1-q21.3	57.52421
OVA_013_C3	Primary	chr1	1757	122	96461726	1.52E+08	Del	0.75175	3.001491	p11.1-q21.3	55.54766
OVA_013_C2	Primary	chr1	1758	121	97187338	1.52E+08	Del	0.755564	3.14838	p11.1-q21.3	54.86926
OVA_013_C1	Primary	chr1	1772	122	97187340	1.52E+08	Del	0.701164	3.125184	p11.1-q21.3	54.89791
OVA_365_M1	Metastasis	chr17	5055	471	6157	43473935	Loss	0.854003	1.907418	p11.1-q21.31	43.46778
OVA_365_M3	Metastasis	chr17	5231	510	6188	43474198	Loss	0.827915	1.890476	p11.1-q21.31	43.46801
OVA_013_C2	Primary	chr9	557	20	40500070	86617489	Loss	0.755564	3.14838	p11.1-q21.32	46.11742
OVA_013_C1	Primary	chr9	541	20	40501731	86559794	Loss	0.701164	3.125184	p11.1-q21.32	46.05806
OVA_047_M1	Metastasis	chr17	5426	488	436105	47875826	Del	0.624347	2.86771	p11.1-q21.33	47.43972
OVA_047_M3	Metastasis	chr17	5418	484	436105	47788710	Del	0.605088	2.815208	p11.1-q21.33	47.35261
OVA_047_M2	Metastasis	chr17	5427	487	436121	47866244	Del	0.599725	2.678568	p11.1-q21.33	47.43012
OVA_365_C2	Primary	chr17	5997	615	6188	51902431	Loss	0.629033	1.960121	p11.1-q22	51.89624
OVA_013_M1	Metastasis	chr1	2443	172	96461726	1.56E+08	Loss	0.621947	2.883383	p11.1-q22	59.72453
OVA_048_M3	Metastasis	chr10	1887	192	93257	72511377	Del	0.59385	3.387584	p11.1-q22.1	72.41812
OVA_048_M1	Metastasis	chr10	1183	109	18963038	71211368	Del	0.625824	3.962486	p11.1-q22.1	52.24833
OVA_048_M3	Metastasis	chr7	2298	198	19184647	99030889	Del	0.59385	3.387584	p11.1-q22.1	79.84624
OVA_047_M1	Metastasis	chr6	2717	182	41659215	1.18E+08	Loss	0.624347	2.86771	p11.1-q22.1	76.23746
OVA_047_C1	Primary	chr2	2083	217	90260191	1.42E+08	Loss	0.576016	2.862818	p11.1-q22.1	51.68599
OVA_013_M1	Metastasis	chr21	375	46	9826872	33083799	Del	0.621947	2.883383	p11.1-q22.11	23.25693
OVA_003_C2	Primary	chr10	2896	296	93257	75479125	Del	0.709607	3.665802	p11.1-q22.2	75.38587
OVA_003_C2	Primary	chr2	6085	522	1982850	1.44E+08	Del	0.709607	3.665802	p11.1-q22.2	141.8152
OVA_047_M2	Metastasis	chr8	1801	105	33416189	1.01E+08	Loss	0.599725	2.678568	p11.1-q22.2	67.60456
OVA_047_M3	Metastasis	chr8	1799	105	35425629	1.01E+08	Loss	0.605088	2.815208	p11.1-q22.2	65.6254
OVA_047_C2	Primary	chr9	895	79	37770086	93225996	Loss	0.575254	2.829045	p11.1-q22.2	55.45591
OVA_047_C1	Primary	chr9	895	79	37780831	93267970	Loss	0.576016	2.862818	p11.1-q22.2	55.48714
OVA_047_M2	Metastasis	chr21	1721	208	9696141	48084304	Loss	0.599725	2.678568	p11.1-q22.3	38.38816
OVA_365_M1	Metastasis	chr21	1678	202	9696141	48084304	Loss	0.854003	1.907418	p11.1-q22.3	38.38816
OVA_378_C1	Primary	chr21	1875	233	9696141	48084177	Loss	0.616455	2.593914	p11.1-q22.3	38.38804
OVA_378_M1	Metastasis	chr21	1881	233	9696141	48084304	Loss	0.753235	2.555202	p11.1-q22.3	38.38816
OVA_378_M3	Metastasis	chr21	1871	231	9696141	48084304	Loss	0.692693	2.569487	p11.1-q22.3	38.38816
OVA_047_C1	Primary	chr21	1721	208	9696158	48084177	Loss	0.576016	2.862818	p11.1-q22.3	38.38802
OVA_047_CO	Contralateral ovary	chr21	1719	207	9696158	48084177	Loss	0.55613	2.930893	p11.1-q22.3	38.38802
OVA_047_M1	Metastasis	chr21	1721	208	9696158	48084282	Loss	0.624347	2.86771	p11.1-q22.3	38.38812
OVA_378_C2	Primary	chr21	1878	233	9696158	48084304	Loss	0.607575	2.601752	p11.1-q22.3	38.38815
OVA_378_C3	Primary	chr21	1881	233	9696158	48084282	Loss	0.517221	2.617793	p11.1-q22.3	38.38812
OVA_378_M2	Metastasis	chr21	1878	232	9696158	48084340	Loss	0.840932	2.531102	p11.1-q22.3	38.38818

Sample	Tissue type	chrom	num.mark	nhet	start	end	Classificati	Purity	Ploidy	Cyto	Size_Mb
OVA_048_M2	Metastasis	chr21	1318	162	9826193	48084304	Del	0.405007	3.72502	p11.1-q22.3	38.25811
OVA_048_M3	Metastasis	chr21	1319	162	9826194	48084282	Del	0.59385	3.387584	p11.1-q22.3	38.25809
OVA_048_M1	Metastasis	chr21	1319	162	9826201	48084304	Del	0.625824	3.962486	p11.1-q22.3	38.2581
OVA_003_C1	Primary	chr21	1741	189	9826761	48084282	Loss	0.852911	1.812048	p11.1-q22.3	38.25752
OVA_003_C2	Primary	chr21	1727	188	9826761	48084340	Del	0.709607	3.665802	p11.1-q22.3	38.25758
OVA_003_C3	Primary	chr21	1748	189	9826761	48084282	Loss	0.820093	1.828085	p11.1-q22.3	38.25752
OVA_013_C1	Primary	chr21	1176	125	9826872	45725199	Loss	0.701164	3.125184	p11.1-q22.3	35.89833
OVA_048_M3	Metastasis	chrX	2383	112	2942251	1.1E+08	Del	0.59385	3.387584	p11.1-q23	107.4933
OVA_013_M1	Metastasis	chr18	1853	163	9195607	77933813	Del	0.621947	2.883383	p11.1-q23	68.73821
OVA_013_C2	Primary	chr8	1739	88	38834035	1.06E+08	Loss	0.755564	3.14838	p11.1-q23.1	67.59664
OVA_013_M1	Metastasis	chr8	1740	88	38840075	1.06E+08	Loss	0.621947	2.883383	p11.1-q23.1	67.61643
OVA_013_C3	Primary	chr8	1716	82	38987070	1.06E+08	Loss	0.75175	3.001491	p11.1-q23.1	67.34418
OVA_048_C3	Primary	chr2	1723	152	86712820	1.53E+08	Gain	0.715645	1.786574	p11.1-q23.3	65.87146
OVA_013_C2	Primary	chr3	4616	307	39225585	1.43E+08	Del	0.755564	3.14838	p11.1-q24	104.1457
OVA_013_C1	Primary	chr3	4608	307	39230930	1.44E+08	Del	0.701164	3.125184	p11.1-q24	104.2849
OVA_013_C3	Primary	chr3	4603	307	39230930	1.43E+08	Del	0.75175	3.001491	p11.1-q24	104.1403
OVA_013_M1	Metastasis	chr3	4605	307	39307256	1.44E+08	Loss	0.621947	2.883383	p11.1-q24	104.2437
OVA_048_M1	Metastasis	chr16	2086	191	33951036	88495575	Del	0.625824	3.962486	p11.1-q24.2	54.54454
OVA_365_M1	Metastasis	chr16	6572	598	67062	90232838	Loss	0.854003	1.907418	p11.1-q24.3	90.16578
OVA_365_M3	Metastasis	chr16	6810	653	67062	90232838	Loss	0.827915	1.890476	p11.1-q24.3	90.16578
OVA_378_M1	Metastasis	chr16	7383	647	67062	90233312	Loss	0.753235	2.555202	p11.1-q24.3	90.16625
OVA_378_M2	Metastasis	chr16	7374	646	67062	90233312	Loss	0.840932	2.531102	p11.1-q24.3	90.16625
OVA_378_M3	Metastasis	chr16	7351	642	67062	90233312	Loss	0.692693	2.569487	p11.1-q24.3	90.16625
OVA_378_C1	Primary	chr16	3912	356	30582888	90233312	Loss	0.616455	2.593914	p11.1-q24.3	59.65042
OVA_378_C3	Primary	chr16	3923	359	30582888	90233312	Loss	0.517221	2.617793	p11.1-q24.3	59.65042
OVA_378_C2	Primary	chr16	3917	358	30596879	90233312	Loss	0.607575	2.601752	p11.1-q24.3	59.63643
OVA_013_C2	Primary	chr17	6339	496	6157	72240168	Del	0.755564	3.14838	p11.1-q25.1	72.23401
OVA_013_C3	Primary	chr17	6317	487	6157	71364631	Del	0.75175	3.001491	p11.1-q25.1	71.35847
OVA_013_M1	Metastasis	chr17	6364	497	6157	72277879	Del	0.621947	2.883383	p11.1-q25.1	72.27172
OVA_003_C1	Primary	chr17	7249	643	6188	72200447	Loss	0.852911	1.812048	p11.1-q25.1	72.19426
OVA_003_C2	Primary	chr17	7231	638	6188	71757108	Del	0.709607	3.665802	p11.1-q25.1	71.75092
OVA_003_C3	Primary	chr17	7295	649	6188	71757108	Loss	0.820093	1.828085	p11.1-q25.1	71.75092
OVA_013_C1	Primary	chr17	5474	423	6900076	72281367	Del	0.701164	3.125184	p11.1-q25.1	65.38129
OVA_378_C1	Primary	chr17	9781	995	6157	81083660	Loss	0.616455	2.593914	p11.1-q25.3	81.0775
OVA_378_C2	Primary	chr17	9800	995	6157	81187975	Loss	0.607575	2.601752	p11.1-q25.3	81.18182
OVA_378_C3	Primary	chr17	9810	996	6157	81187975	Loss	0.517221	2.617793	p11.1-q25.3	81.18182
OVA_048_M1	Metastasis	chr3	5719	500	239350	1.61E+08	Del	0.625824	3.962486	p11.1-q25.33	160.4403
OVA_048_M2	Metastasis	chr3	5724	500	239347	1.61E+08	Del	0.405007	3.72502	p11.1-q26.1	160.7004
OVA_048_M3	Metastasis	chr3	2845	274	53882661	1.61E+08	Del	0.59385	3.387584	p11.1-q26.1	107.0846
OVA_013_C3	Primary	chrX	1683	71	57620760	1.32E+08	Loss	0.75175	3.001491	p11.1-q26.2	74.47155
OVA_378_C1	Primary	chr10	6402	581	93257	1.36E+08	Loss	0.616455	2.593914	p11.1-q26.3	135.4075
OVA_378_C2	Primary	chr10	6426	581	93257	1.36E+08	Loss	0.607575	2.601752	p11.1-q26.3	135.4075
OVA_378_M3	Metastasis	chr10	6388	575	93257	1.36E+08	Loss	0.692693	2.569487	p11.1-q26.3	135.4075
OVA_378_C3	Primary	chr10	6421	579	93387	1.36E+08	Loss	0.517221	2.617793	p11.1-q26.3	135.4074
OVA_378_M1	Metastasis	chr10	6425	580	93387	1.36E+08	Loss	0.753235	2.555202	p11.1-q26.3	135.4074
OVA_378_M2	Metastasis	chr10	6416	581	93387	1.36E+08	Loss	0.840932	2.531102	p11.1-q26.3	135.4074
OVA_365_M1	Metastasis	chrX	3957	181	2700202	1.35E+08	Loss	0.854003	1.907418	p11.1-q26.3	132.7878
OVA_365_M3	Metastasis	chrX	4013	186	2700202	1.35E+08	Loss	0.827915	1.890476	p11.1-q26.3	132.7878
OVA_365_C2	Primary	chrX	4009	184	2798969	1.35E+08	Loss	0.629033	1.960121	p11.1-q26.3	132.689
OVA_013_C2	Primary	chrX	1817	80	55187103	1.35E+08	Loss	0.755564	3.14838	p11.1-q26.3	80.10506
OVA_013_C1	Primary	chrX	1778	79	55187222	1.34E+08	Loss	0.701164	3.125184	p11.1-q26.3	78.99902
OVA_013_M1	Metastasis	chrX	1816	80	55187421	1.35E+08	Loss	0.621947	2.883383	p11.1-q26.3	80.10474
OVA_378_C2	Primary	chr6	8391	895	203619	1.71E+08	Loss	0.607575	2.601752	p11.1-q27	170.6898
OVA_378_M2	Metastasis	chr6	8389	896	203619	1.71E+08	Loss	0.840932	2.531102	p11.1-q27	170.6898
OVA_378_C3	Primary	chr6	8390	896	203666	1.71E+08	Loss	0.517221	2.617793	p11.1-q27	170.6898
OVA_378_M1	Metastasis	chr6	8397	896	203699	1.71E+08	Loss	0.753235	2.555202	p11.1-q27	170.6898
OVA_378_C1	Primary	chr6	8367	886	203722	1.71E+08	Loss	0.616455	2.593914	p11.1-q27	170.6897
OVA_378_M3	Metastasis	chr6	8349	892	203722	1.71E+08	Loss	0.692693	2.569487	p11.1-q27	170.6897
OVA_378_C1	Primary	chrX	5180	307	2700027	1.55E+08	Del	0.616455	2.593914	p11.1-q28	152.0747
OVA_378_C2	Primary	chrX	5257	313	2700027	1.55E+08	Del	0.607575	2.601752	p11.1-q28	152.0747
OVA_048_C3	Primary	chrX	3482	183	2700089	1.55E+08	Gain	0.715645	1.786574	p11.1-q28	152.0542
OVA_048_M1	Metastasis	chrX	3482	183	2700194	1.55E+08	Del	0.625824	3.962486	p11.1-q28	152.0541
OVA_048_M2	Metastasis	chrX	3482	183	2700194	1.55E+08	Del	0.405007	3.72502	p11.1-q28	152.0541
OVA_378_C3	Primary	chrX	5273	313	2700202	1.55E+08	Del	0.517221	2.617793	p11.1-q28	152.0745
OVA_378_M1	Metastasis	chrX	5281	315	2700202	1.55E+08	Del	0.753235	2.555202	p11.1-q28	152.0745
OVA_378_M2	Metastasis	chrX	5147	299	2700202	1.55E+08	Del	0.840932	2.531102	p11.1-q28	152.0745

Sample	Tissue type	chrom	num.mark	nhet	start	end	Classificati	Purity	Ploidy	Cyto	Size_Mb
OVA_378_M3	Metastasis	chrX	5048	296	2700202	1.55E+08	Del	0.692693	2.569487	p11.1-q28	152.0745
OVA_003_C2	Primary	chrX	3937	160	32053662	1.55E+08	Del	0.709607	3.665802	p11.1-q28	122.721
OVA_047_M2	Metastasis	chrX	3486	172	46466495	1.55E+08	Loss	0.599725	2.678568	p11.1-q28	108.3082
OVA_047_M1	Metastasis	chrX	3476	170	46696492	1.55E+08	Loss	0.624347	2.86771	p11.1-q28	108.0782
OVA_047_M3	Metastasis	chrX	3476	170	46696492	1.55E+08	Loss	0.605088	2.815208	p11.1-q28	108.0782
OVA_047_CO	Contralateral ovary	chr7	3208	245	45717711	1.27E+08	Loss	0.55613	2.930893	p11.1-q32.1	81.51784
OVA_048_M1	Metastasis	chr2	3521	279	70679005	1.87E+08	Del	0.625824	3.962486	p11.1-q32.1	115.9998
OVA_048_M2	Metastasis	chr2	2897	224	86712820	1.87E+08	Del	0.405007	3.72502	p11.1-q32.1	99.97637
OVA_048_M1	Metastasis	chr7	4128	338	19184647	1.42E+08	Del	0.625824	3.962486	p11.1-q34	122.3527
OVA_048_M2	Metastasis	chr7	4105	337	20444402	1.41E+08	Del	0.405007	3.72502	p11.1-q34	121.0341
OVA_378_C1	Primary	chr9	6845	588	116986	1.41E+08	Loss	0.616455	2.593914	p11.1-q34.3	140.9547
OVA_378_C2	Primary	chr9	6867	589	116986	1.41E+08	Loss	0.607575	2.601752	p11.1-q34.3	140.9547
OVA_378_C3	Primary	chr9	6867	589	116986	1.41E+08	Loss	0.517221	2.617793	p11.1-q34.3	140.9547
OVA_378_M3	Metastasis	chr9	6827	585	116986	1.41E+08	Loss	0.692693	2.569487	p11.1-q34.3	140.9547
OVA_378_M1	Metastasis	chr9	6868	590	117158	1.41E+08	Loss	0.753235	2.555202	p11.1-q34.3	140.9546
OVA_378_M2	Metastasis	chr9	6863	590	117158	1.41E+08	Loss	0.840932	2.531102	p11.1-q34.3	140.9546
OVA_048_M1	Metastasis	chr9	4371	416	32418237	1.41E+08	Del	0.625824	3.962486	p11.1-q34.3	108.6533
OVA_378_M1	Metastasis	chr7	8061	698	193222	1.59E+08	Loss	0.753235	2.555202	p11.1-q36.3	158.8321
OVA_378_M3	Metastasis	chr7	8025	690	193222	1.59E+08	Loss	0.692693	2.569487	p11.1-q36.3	158.8321
OVA_378_M2	Metastasis	chr7	8059	698	193245	1.59E+08	Loss	0.840932	2.531102	p11.1-q36.3	158.8321
OVA_378_M1	Metastasis	chr8	9	1	43092798	43097021	Del	0.753235	2.555202	p11.21-p11.21	0.004223
OVA_378_C2	Primary	chr8	9	1	43092812	43097021	Del	0.607575	2.601752	p11.21-p11.21	0.004209
OVA_378_M2	Metastasis	chr8	9	1	43092859	43097021	Del	0.840932	2.531102	p11.21-p11.21	0.004162
OVA_047_C2	Primary	chr8	427	18	36661686	43054563	Loss	0.575254	2.829045	p11.21-p11.23	6.392877
OVA_047_C2	Primary	chr18	599	50	658064	15006052	Del	0.575254	2.829045	p11.21-p11.32	14.34799
OVA_048_M2	Metastasis	chr10	1066	120	93257	35842247	Del	0.405007	3.72502	p11.21-p15.3	35.74899
OVA_013_C3	Primary	chr8	195	10	36703323	38975542	Del	0.75175	3.001491	p11.22-p11.23	2.272219
OVA_047_CO	Contralateral ovary	chrX	520	11	45241305	53117072	Loss	0.55613	2.930893	p11.22-p11.3	7.875767
OVA_013_C3	Primary	chr18	335	47	47610	9134071	Del	0.75175	3.001491	p11.22-p11.32	9.086461
OVA_013_M1	Metastasis	chr18	340	47	47615	9134071	Del	0.621947	2.883383	p11.22-p11.32	9.086456
OVA_013_C1	Primary	chr18	339	48	47632	9204535	Del	0.701164	3.125184	p11.22-p11.32	9.156903
OVA_013_C2	Primary	chr18	325	44	47632	9124948	Del	0.755564	3.14838	p11.22-p11.32	9.077316
OVA_047_C1	Primary	chr8	219	3	33416189	38991720	Loss	0.576016	2.862818	p11.22-p12	5.575531
OVA_013_C2	Primary	chr8	190	8	33416222	38832610	Del	0.755564	3.14838	p11.22-p12	5.416388
OVA_013_M1	Metastasis	chr8	190	7	33451309	38835561	Del	0.621947	2.883383	p11.22-p12	5.384252
OVA_047_CO	Contralateral ovary	chr8	211	2	35453058	38965271	Del	0.55613	2.930893	p11.22-p12	3.512213
OVA_013_C1	Primary	chr8	206	13	35583815	38975542	Del	0.701164	3.125184	p11.22-p12	3.391727
OVA_013_C3	Primary	chr8	15	3	35453057	36692176	Loss	0.75175	3.001491	p11.23-p12	1.239119
OVA_047_M1	Metastasis	chr12	1461	121	5021840	27799113	Del	0.624347	2.86771	p11.23-p13.32	22.77727
OVA_047_M1	Metastasis	chrX	1199	66	2700202	46618076	Del	0.624347	2.86771	p11.23-p22.33	43.91787
OVA_047_M3	Metastasis	chrX	1197	66	2700202	46618076	Del	0.605088	2.815208	p11.23-p22.33	43.91787
OVA_047_M2	Metastasis	chrX	1179	64	2816095	46434300	Del	0.599725	2.678568	p11.23-p22.33	43.61821
OVA_047_C2	Primary	chr8	1494	181	163553	36644980	Del	0.575254	2.829045	p11.23-p23.3	36.48143
OVA_047_C1	Primary	chr17	8	1	18380052	18454959	Del	0.576016	2.862818	p11.2-p11.2	0.074907
OVA_378_C3	Primary	chr16	10	0	28354003	28495341	Loss	0.517221	2.617793	p11.2-p11.2	0.141338
OVA_378_C1	Primary	chr16	23	3	28426094	28509188	Loss	0.616455	2.593914	p11.2-p11.2	0.083094
OVA_048_M3	Metastasis	chr16	11	1	31724836	31963719	Del	0.59385	3.387584	p11.2-p11.2	0.238883
OVA_047_C2	Primary	chr2	384	41	84517897	90259898	Del	0.575254	2.829045	p11.2-p11.2	5.742001
OVA_047_CO	Contralateral ovary	chr2	381	40	81424784	90260191	Del	0.55613	2.930893	p11.2-p12	8.835407
OVA_047_C1	Primary	chr2	384	41	83083971	90259979	Del	0.576016	2.862818	p11.2-p12	7.176008
OVA_047_C2	Primary	chr16	587	52	25268368	30967493	Del	0.575254	2.829045	p11.2-p12.1	5.699125
OVA_047_CO	Contralateral ovary	chr16	572	49	27245629	30974744	Del	0.55613	2.930893	p11.2-p12.1	3.729115
OVA_047_C1	Primary	chr16	560	47	27363944	30965231	Del	0.576016	2.862818	p11.2-p12.1	3.601287
OVA_047_C1	Primary	chr6	232	27	53159249	57055354	Loss	0.576016	2.862818	p11.2-p12.1	3.896105
OVA_048_M1	Metastasis	chr6	126	17	56047400	57372263	Del	0.625824	3.962486	p11.2-p12.1	1.324863
OVA_047_M2	Metastasis	chr7	113	9	50057768	55780845	Del	0.599725	2.678568	p11.2-p12.2	5.723077
OVA_047_M1	Metastasis	chr7	98	8	50143938	55629781	Del	0.624347	2.86771	p11.2-p12.2	5.485843
OVA_365_M1	Metastasis	chr6	417	33	47754498	57393203	Amp	0.854003	1.907418	p11.2-p12.3	9.638705
OVA_365_M3	Metastasis	chr6	414	33	47762949	57372263	Amp	0.827915	1.890476	p11.2-p12.3	9.609314
OVA_047_C1	Primary	chr17	724	65	8926221	18314947	Del	0.576016	2.862818	p11.2-p13.1	9.388726
OVA_378_C2	Primary	chr16	1046	82	12620413	28332182	Del	0.607575	2.601752	p11.2-p13.12	15.71177
OVA_378_C3	Primary	chr16	1051	82	12662395	28332182	Del	0.517221	2.617793	p11.2-p13.12	15.66979
OVA_378_C1	Primary	chr16	1034	77	12758836	28354496	Del	0.616455	2.593914	p11.2-p13.12	15.59566
OVA_048_M1	Metastasis	chr17	2100	215	5994	19319211	Del	0.625824	3.962486	p11.2-p13.3	19.31322
OVA_048_M3	Metastasis	chr16	2509	223	684579	31613480	Del	0.59385	3.387584	p11.2-p13.3	30.9289
OVA_048_M2	Metastasis	chr16	2520	224	684590	31963719	Del	0.405007	3.72502	p11.2-p13.3	31.27913

Sample	Tissue type	chrom	num.mark	nhet	start	end	Classificati	Purity	Ploidy	Cyto	Size_Mb
OVA_048_M1	Metastasis	chr16	2517	224	684682	31963719	Del	0.625824	3.962486	p11.2-p13.3	31.27904
OVA_013_M1	Metastasis	chr11	2117	220	1247458	44640291	Del	0.621947	2.883383	p11.2-p15.5	43.39283
OVA_013_C1	Primary	chr11	2098	219	1267971	44640519	Del	0.701164	3.125184	p11.2-p15.5	43.37255
OVA_013_C2	Primary	chr11	2033	217	1776114	44640519	Del	0.755564	3.14838	p11.2-p15.5	42.86441
OVA_013_C3	Primary	chr11	2042	217	1780694	44640477	Del	0.75175	3.001491	p11.2-p15.5	42.85978
OVA_048_M3	Metastasis	chr6	875	97	41743258	57185316	Del	0.59385	3.387584	p11.2-p21.1	15.44206
OVA_003_C2	Primary	chr9	795	71	27286259	44175598	Del	0.709607	3.665802	p11.2-p21.2	16.88934
OVA_048_M2	Metastasis	chr6	940	100	39873309	57372263	Del	0.405007	3.72502	p11.2-p21.2	17.49895
OVA_365_C2	Primary	chr9	1535	110	117666	45362386	Loss	0.629033	1.960121	p11.2-p24.3	45.24472
OVA_365_M1	Metastasis	chr9	1494	106	117666	44175088	Loss	0.854003	1.907418	p11.2-p24.3	44.05742
OVA_365_M3	Metastasis	chr9	1529	110	117666	44876257	Loss	0.827915	1.890476	p11.2-p24.3	44.75859
OVA_048_M2	Metastasis	chr2	3047	275	45521	86709704	Del	0.405007	3.72502	p11.2-p25.3	86.66418
OVA_365_C2	Primary	chr6	4380	587	549073	58287632	Gain	0.629033	1.960121	p11.2-p25.3	57.73856
OVA_047_C1	Primary	chr1	2165	194	67390416	1.21E+08	Del	0.576016	2.862818	p11.2-p31.3	53.53967
OVA_047_C2	Primary	chr1	2557	234	55548934	1.21E+08	Del	0.575254	2.829045	p11.2-p32.3	65.3797
OVA_047_CO	Contralateral ovary	chr1	5017	437	27312407	1.21E+08	Del	0.55613	2.930893	p11.2-p36.11	93.61768
OVA_047_C2	Primary	chr18	40	2	12863	649804	Loss	0.575254	2.829045	p11.32-p11.32	0.636941
OVA_047_CO	Contralateral ovary	chr18	40	2	12863	649804	Loss	0.55613	2.930893	p11.32-p11.32	0.636941
OVA_378_C2	Primary	chr18	13	2	12863	118446	Loss	0.607575	2.601752	p11.32-p11.32	0.105583
OVA_378_M3	Metastasis	chr18	11	2	12863	109192	Loss	0.692693	2.569487	p11.32-p11.32	0.096329
OVA_378_M2	Metastasis	chr18	15	2	12905	166819	Loss	0.840932	2.531102	p11.32-p11.32	0.153914
OVA_378_C1	Primary	chr18	11	2	12916	109178	Del	0.616455	2.593914	p11.32-p11.32	0.096262
OVA_378_M1	Metastasis	chr18	12	2	12916	111413	Loss	0.753235	2.555202	p11.32-p11.32	0.098497
OVA_048_C3	Primary	chr18	13	1	47615	203073	Gain	0.715645	1.786574	p11.32-p11.32	0.155458
OVA_047_C2	Primary	chrX	1040	57	8522064	44970788	Del	0.575254	2.829045	p11.3-p22.31	36.44872
OVA_047_C1	Primary	chrX	1162	61	2700194	44935937	Del	0.576016	2.862818	p11.3-p22.33	42.23574
OVA_047_CO	Contralateral ovary	chrX	1167	61	2700194	45059862	Del	0.55613	2.930893	p11.3-p22.33	42.35967
OVA_047_C2	Primary	chr4	24	1	49034709	49637620	Amp	0.575254	2.829045	p11-p11	0.602911
OVA_047_C2	Primary	chr4	198	16	42069098	49019311	Loss	0.575254	2.829045	p11-p13	6.950213
OVA_047_C2	Primary	chr5	32	0	43503698	49928997	Loss	0.575254	2.829045	p11-q11.1	6.425299
OVA_013_C1	Primary	chr5	596	42	32444286	52370174	Del	0.701164	3.125184	p11-q11.2	19.92589
OVA_013_M1	Metastasis	chr5	614	45	32523114	53183268	Del	0.621947	2.883383	p11-q11.2	20.66015
OVA_013_C3	Primary	chr5	595	41	32523181	52370174	Del	0.75175	3.001491	p11-q11.2	19.84699
OVA_013_C2	Primary	chr5	595	42	33441128	52942197	Del	0.755564	3.14838	p11-q11.2	19.50107
OVA_378_C1	Primary	chr19	5228	621	156855	30019402	Loss	0.616455	2.593914	p11-q12	29.86255
OVA_378_C3	Primary	chr19	5243	625	156855	30097188	Loss	0.517221	2.617793	p11-q12	29.94033
OVA_378_M1	Metastasis	chr19	5253	627	156855	30106297	Loss	0.753235	2.555202	p11-q12	29.94944
OVA_378_M2	Metastasis	chr19	5243	624	156855	30102856	Loss	0.840932	2.531102	p11-q12	29.946
OVA_378_C2	Primary	chr19	5238	624	156928	30097214	Loss	0.607575	2.601752	p11-q12	29.94029
OVA_378_M3	Metastasis	chr19	5224	624	156928	30097214	Loss	0.692693	2.569487	p11-q12	29.94029
OVA_013_C1	Primary	chr19	3003	327	5273496	33954954	Loss	0.701164	3.125184	p11-q13.11	28.68146
OVA_013_C3	Primary	chr19	2999	327	5455735	33902652	Loss	0.75175	3.001491	p11-q13.11	28.44692
OVA_013_C2	Primary	chr19	3003	327	5455800	34180194	Loss	0.755564	3.14838	p11-q13.11	28.72439
OVA_013_M1	Metastasis	chr19	2986	327	5455800	33703206	Loss	0.621947	2.883383	p11-q13.11	28.24741
OVA_003_C2	Primary	chr5	730	64	43644318	72354329	Del	0.709607	3.665802	p11-q13.2	28.71001
OVA_013_C2	Primary	chr4	1452	143	13370308	74364838	Loss	0.755564	3.14838	p11-q13.3	60.99453
OVA_013_C1	Primary	chr4	1388	133	15482322	74319538	Loss	0.701164	3.125184	p11-q13.3	58.83722
OVA_003_C2	Primary	chr4	717	62	47575086	73280525	Del	0.709607	3.665802	p11-q13.3	25.70544
OVA_047_M3	Metastasis	chr19	3918	373	13443770	46248501	Loss	0.605088	2.815208	p11-q13.32	32.80473
OVA_047_M1	Metastasis	chr19	3888	371	13470522	46141845	Loss	0.624347	2.86771	p11-q13.32	32.67132
OVA_047_M2	Metastasis	chr19	3918	372	13470522	46248501	Loss	0.599725	2.678568	p11-q13.32	32.77798
OVA_048_M3	Metastasis	chr19	2291	286	18375236	45899767	Del	0.59385	3.387584	p11-q13.32	27.52453
OVA_048_M1	Metastasis	chr19	1953	233	20844361	45899731	Del	0.625824	3.962486	p11-q13.32	25.05537
OVA_048_M2	Metastasis	chr19	2718	324	18368810	48815357	Del	0.405007	3.72502	p11-q13.33	30.44655
OVA_003_C2	Primary	chr19	7673	751	9251220	59094003	Del	0.709607	3.665802	p11-q13.43	49.84278
OVA_047_CO	Contralateral ovary	chr19	5381	546	19959497	59009949	Loss	0.55613	2.930893	p11-q13.43	39.05045
OVA_047_C1	Primary	chr19	5400	552	20012070	59065307	Loss	0.576016	2.862818	p11-q13.43	39.05324
OVA_047_C2	Primary	chr19	5363	545	20215030	58992080	Loss	0.575254	2.829045	p11-q13.43	38.77705
OVA_047_C1	Primary	chr4	1742	158	13340010	77089557	Loss	0.576016	2.862818	p11-q21.1	63.74955
OVA_047_CO	Contralateral ovary	chr4	1739	160	13370308	77105952	Loss	0.55613	2.930893	p11-q21.1	63.73564
OVA_048_M2	Metastasis	chr4	1119	89	30498953	82366788	Del	0.405007	3.72502	p11-q21.21	51.86784
OVA_048_M1	Metastasis	chr4	1119	89	30723851	82366788	Del	0.625824	3.962486	p11-q21.21	51.64294
OVA_048_M3	Metastasis	chr4	1115	89	30921913	82366788	Loss	0.59385	3.387584	p11-q21.21	51.44488
OVA_048_C3	Primary	chr4	917	69	41266151	82366788	Gain	0.715645	1.786574	p11-q21.21	41.10064
OVA_047_M2	Metastasis	chr4	3130	279	8393989	1.14E+08	Loss	0.599725	2.678568	p11-q26	105.9086
OVA_047_M1	Metastasis	chr4	3144	279	8394168	1.15E+08	Loss	0.624347	2.86771	p11-q26	106.1887

Sample	Tissue type	chrom	num.mark	nhet	start	end	Classificati	Purity	Ploidy	Cyto	Size_Mb
OVA_047_M3	Metastasis	chr4	3069	274	9909926	1.14E+08	Loss	0.605088	2.815208	p11-q26	104.5164
OVA_378_M1	Metastasis	chr5	2271	194	44813282	1.31E+08	Loss	0.753235	2.555202	p11-q31.1	86.45596
OVA_378_M3	Metastasis	chr5	2545	216	45696056	1.35E+08	Loss	0.692693	2.569487	p11-q31.1	88.99459
OVA_048_M3	Metastasis	chr5	2523	227	36953687	1.4E+08	Del	0.59385	3.387584	p11-q31.3	103.1316
OVA_048_M1	Metastasis	chr5	2483	225	37239884	1.4E+08	Del	0.625824	3.962486	p11-q31.3	102.8406
OVA_048_M2	Metastasis	chr5	2483	225	37247684	1.4E+08	Del	0.405007	3.72502	p11-q31.3	102.8376
OVA_378_M2	Metastasis	chr5	2982	259	45695935	1.4E+08	Loss	0.840932	2.531102	p11-q31.3	94.4858
OVA_378_C1	Primary	chr4	5909	547	53218	1.91E+08	Loss	0.616455	2.593914	p11-q35.2	190.8943
OVA_378_C2	Primary	chr4	5918	549	53218	1.91E+08	Loss	0.607575	2.601752	p11-q35.2	190.8942
OVA_378_C3	Primary	chr4	5915	550	53218	1.91E+08	Loss	0.517221	2.617793	p11-q35.2	190.8942
OVA_378_M2	Metastasis	chr4	5908	548	53218	1.91E+08	Loss	0.840932	2.531102	p11-q35.2	190.8943
OVA_378_M1	Metastasis	chr4	5915	550	53428	1.91E+08	Loss	0.753235	2.555202	p11-q35.2	190.8941
OVA_378_M3	Metastasis	chr4	5892	542	53428	1.91E+08	Loss	0.692693	2.569487	p11-q35.2	190.894
OVA_378_C2	Primary	chr5	5680	511	45696056	1.81E+08	Loss	0.607575	2.601752	p11-q35.3	134.9912
OVA_013_M1	Metastasis	chr12	44	1	23737481	25679137	Amp	0.621947	2.883383	p12.1-p12.1	1.941656
OVA_013_C3	Primary	chr12	18	1	26208428	26383959	Del	0.75175	3.001491	p12.1-p12.1	0.175531
OVA_048_M1	Metastasis	chr6	265	26	46656082	56044578	Del	0.625824	3.962486	p12.1-p12.3	9.388496
OVA_013_M1	Metastasis	chr12	608	43	10205224	23728815	Gain	0.621947	2.883383	p12.1-p13.2	13.52359
OVA_047_CO	Contralateral ovary	chr16	1252	101	4412112	27238110	Loss	0.55613	2.930893	p12.1-p13.3	22.826
OVA_047_C2	Primary	chr16	1238	98	4414304	25266658	Loss	0.575254	2.829045	p12.1-p13.3	20.85235
OVA_047_C1	Primary	chr16	1261	100	4431360	27357927	Loss	0.576016	2.862818	p12.1-p13.3	22.92657
OVA_013_C1	Primary	chr12	1673	149	90681	25801489	Gain	0.701164	3.125184	p12.1-p13.33	25.71081
OVA_013_C2	Primary	chr12	1673	149	90681	25801489	Gain	0.755564	3.14838	p12.1-p13.33	25.71081
OVA_013_C3	Primary	chr12	1673	149	90681	25801489	Gain	0.75175	3.001491	p12.1-p13.33	25.71081
OVA_013_C3	Primary	chr6	521	48	43593289	55360350	Loss	0.75175	3.001491	p12.1-p21.1	11.76706
OVA_013_C2	Primary	chr6	521	48	43594720	55428658	Loss	0.755564	3.14838	p12.1-p21.1	11.83394
OVA_048_C2	Primary	chr6	1031	101	36566832	56035654	Gain	0.736255	1.845483	p12.1-p21.31	19.46882
OVA_047_M1	Metastasis	chr16	1352	106	2988469	22448295	Del	0.624347	2.86771	p12.2-p13.3	19.45983
OVA_047_M2	Metastasis	chr16	1351	107	3014446	22492409	Del	0.599725	2.678568	p12.2-p13.3	19.47796
OVA_047_M3	Metastasis	chr16	1363	107	3016843	23085035	Del	0.605088	2.815208	p12.2-p13.3	20.06819
OVA_047_M2	Metastasis	chr7	1702	165	6628242	49842440	Loss	0.599725	2.678568	p12.2-p22.1	43.2142
OVA_047_M1	Metastasis	chr7	2322	208	193245	50135931	Loss	0.624347	2.86771	p12.2-p22.3	49.94269
OVA_048_M1	Metastasis	chr10	633	76	93257	18948721	Del	0.625824	3.962486	p12.31-p15.3	18.85546
OVA_047_C1	Primary	chr7	426	36	37243832	45703835	Del	0.576016	2.862818	p12.3-p14.1	8.460003
OVA_047_C2	Primary	chr7	425	36	37243832	45703835	Del	0.575254	2.829045	p12.3-p14.1	8.460003
OVA_047_CO	Contralateral ovary	chr7	408	35	37947207	45703835	Del	0.55613	2.930893	p12.3-p14.1	7.756628
OVA_048_M1	Metastasis	chr6	559	61	39851818	46649231	Del	0.625824	3.962486	p12.3-p21.2	6.797413
OVA_365_CO	Contralateral ovary	chr6	1185	112	35473738	47799844	Gain	0.451025	1.890627	p12.3-p21.31	12.32611
OVA_365_M3	Metastasis	chr6	3960	551	404973	47759433	Amp	0.827915	1.890476	p12.3-p25.3	47.35446
OVA_365_M1	Metastasis	chr6	3942	548	630033	47749806	Amp	0.854003	1.907418	p12.3-p25.3	47.11977
OVA_365_M2	Metastasis	chr6	3945	546	656539	47791726	Gain	0.387458	1.893344	p12.3-p25.3	47.13519
OVA_047_M1	Metastasis	chr1	14	1	119923433	1.2E+08	Loss	0.624347	2.86771	p12-p12	0.126913
OVA_003_C3	Primary	chr4	42	4	42618036	47163325	Loss	0.820093	1.828085	p12-p13	4.545289
OVA_047_C2	Primary	chr5	225	14	38418311	43502528	Loss	0.575254	2.829045	p12-p13.1	5.084217
OVA_048_C2	Primary	chr19	333	52	18332993	20656048	Gain	0.736255	1.845483	p12-p13.11	2.323055
OVA_048_M1	Metastasis	chr19	339	55	18368988	20828391	Del	0.625824	3.962486	p12-p13.11	2.459403
OVA_048_C1	Primary	chr19	317	46	18420645	20728156	Gain	0.682521	1.843999	p12-p13.11	2.307511
OVA_048_C3	Primary	chr19	326	49	18420645	20844122	Gain	0.715645	1.786574	p12-p13.11	2.423477
OVA_048_C1	Primary	chr2	316	29	73053104	81423752	Amp	0.682521	1.843999	p12-p13.2	8.370648
OVA_047_M1	Metastasis	chr1	299	26	114301335	1.2E+08	Loss	0.624347	2.86771	p12-p13.2	5.382021
OVA_047_C2	Primary	chr19	3821	308	1358263	20188835	Del	0.575254	2.829045	p12-p13.3	18.83057
OVA_047_C1	Primary	chr19	3809	306	1358726	20011767	Del	0.576016	2.862818	p12-p13.3	18.65304
OVA_003_C2	Primary	chr4	570	59	21764761	47574826	Del	0.709607	3.665802	p12-p15.2	25.81007
OVA_378_C2	Primary	chr5	1417	108	140403	45396783	Loss	0.607575	2.601752	p12-p15.33	45.25638
OVA_003_C2	Primary	chr5	1408	123	140636	43628251	Del	0.709607	3.665802	p12-p15.33	43.48762
OVA_013_C3	Primary	chr4	723	82	13383237	44724158	Loss	0.75175	3.001491	p12-p15.33	31.34092
OVA_003_C1	Primary	chr4	1036	118	6718124	47163325	Loss	0.852911	1.812048	p12-p16.1	40.4452
OVA_047_C1	Primary	chr2	1471	119	44053761	81424784	Loss	0.576016	2.862818	p12-p21	37.37102
OVA_013_M1	Metastasis	chr8	1190	113	6264130	33449690	Del	0.621947	2.883383	p12-p23.1	27.18556
OVA_013_C1	Primary	chr8	1163	111	6264139	35579762	Del	0.701164	3.125184	p12-p23.1	29.31562
OVA_013_C2	Primary	chr8	1142	105	6264280	33371196	Del	0.755564	3.14838	p12-p23.1	27.10692
OVA_013_C3	Primary	chr8	1148	106	6264280	35425629	Del	0.75175	3.001491	p12-p23.1	29.16135
OVA_378_M1	Metastasis	chr8	1432	140	4852032	33451309	Loss	0.753235	2.555202	p12-p23.2	28.59928
OVA_047_CO	Contralateral ovary	chr8	1422	165	163421	35425629	Del	0.55613	2.930893	p12-p23.3	35.26221
OVA_047_M2	Metastasis	chr8	1498	180	163461	33408680	Del	0.599725	2.678568	p12-p23.3	33.24522
OVA_047_C1	Primary	chr8	1475	178	163553	33408680	Del	0.576016	2.862818	p12-p23.3	33.24513

Sample	Tissue type	chrom	num.mark	nhet	start	end	Classificati	Purity	Ploidy	Cyto	Size_Mb
OVA_047_M1	Metastasis	chr8	1497	181	163553	33449690	Del	0.624347	2.86771	p12-p23.3	33.28614
OVA_047_M3	Metastasis	chr8	1498	181	163553	33451309	Del	0.605088	2.815208	p12-p23.3	33.28776
OVA_378_M3	Metastasis	chr8	1599	164	328513	35453058	Loss	0.692693	2.569487	p12-p23.3	35.12455
OVA_378_M2	Metastasis	chr8	1513	152	2024697	33416222	Loss	0.840932	2.531102	p12-p23.3	31.39153
OVA_047_CO	Contralateral ovary	chr2	2404	198	27428238	81424273	Loss	0.55613	2.930893	p12-p23.3	53.99604
OVA_047_C2	Primary	chr2	2316	189	27599132	83083971	Loss	0.575254	2.829045	p12-p23.3	55.48484
OVA_047_M3	Metastasis	chr1	1798	158	78601415	1.2E+08	Loss	0.605088	2.815208	p12-p31.1	41.6785
OVA_048_C3	Primary	chr19	14	6	18368988	18418428	Gain	0.715645	1.786574	p13.11-p13.11	0.04944
OVA_048_C3	Primary	chr19	435	54	16178600	18368529	Amp	0.715645	1.786574	p13.11-p13.12	2.189929
OVA_048_C2	Primary	chr19	411	52	16296282	18331190	Amp	0.736255	1.845483	p13.11-p13.12	2.034908
OVA_048_C1	Primary	chr19	1608	167	9203994	18368533	Amp	0.682521	1.843999	p13.11-p13.2	9.164539
OVA_047_CO	Contralateral ovary	chr19	3796	305	1357082	19944387	Del	0.55613	2.930893	p13.11-p13.3	18.58731
OVA_048_M1	Metastasis	chr19	345	29	13910518	16146711	Amp	0.625824	3.962486	p13.12-p13.13	2.236193
OVA_048_M3	Metastasis	chr19	344	29	13915578	16146711	Gain	0.59385	3.387584	p13.12-p13.13	2.231133
OVA_048_C2	Primary	chr19	381	34	13868573	16284186	Amp	0.736255	1.845483	p13.12-p13.2	2.415613
OVA_048_C3	Primary	chr19	362	32	13868573	16146711	Amp	0.715645	1.786574	p13.12-p13.2	2.278138
OVA_378_C1	Primary	chr16	1968	168	67062	12662448	Loss	0.616455	2.593914	p13.12-p13.3	12.59539
OVA_378_C2	Primary	chr16	1971	171	67062	12618598	Loss	0.607575	2.601752	p13.12-p13.3	12.55154
OVA_378_C3	Primary	chr16	1974	171	67062	12621026	Loss	0.517221	2.617793	p13.12-p13.3	12.55396
OVA_013_C1	Primary	chr17	899	77	6157	6733473	Del	0.701164	3.125184	p13.1-p13.3	6.727316
OVA_047_C1	Primary	chr17	1662	182	6157	8925689	Loss	0.576016	2.862818	p13.1-p13.3	8.919532
OVA_047_C2	Primary	chr17	1668	182	6157	9124602	Loss	0.575254	2.829045	p13.1-p13.3	9.118445
OVA_047_CO	Contralateral ovary	chr17	1667	182	6157	9124602	Loss	0.55613	2.930893	p13.1-p13.3	9.118445
OVA_013_C3	Primary	chr9	794	62	21077371	39177373	Gain	0.75175	3.001491	p13.1-p21.3	18.1
OVA_013_M1	Metastasis	chr9	794	62	21077716	39178260	Gain	0.621947	2.883383	p13.1-p21.3	18.10054
OVA_003_C2	Primary	chr19	144	22	8807850	9237337	Loss	0.709607	3.665802	p13.2-p13.2	0.429487
OVA_003_C3	Primary	chr19	139	20	8808942	9225973	Amp	0.820093	1.828085	p13.2-p13.2	0.417031
OVA_003_C1	Primary	chr19	129	19	8976713	9237337	Amp	0.852911	1.812048	p13.2-p13.2	0.260624
OVA_048_M2	Metastasis	chr19	849	86	9028373	13387970	Loss	0.405007	3.72502	p13.2-p13.2	4.359597
OVA_048_C2	Primary	chr19	811	81	9203994	13868171	Amp	0.736255	1.845483	p13.2-p13.2	4.664177
OVA_048_C3	Primary	chr19	811	81	9203994	13868171	Amp	0.715645	1.786574	p13.2-p13.2	4.664177
OVA_047_M1	Metastasis	chr19	2884	240	156855	13446573	Del	0.624347	2.86771	p13.2-p13.3	13.28972
OVA_047_M2	Metastasis	chr19	2889	240	156855	13446573	Loss	0.599725	2.678568	p13.2-p13.3	13.28972
OVA_047_M3	Metastasis	chr19	2886	239	156855	13418707	Del	0.605088	2.815208	p13.2-p13.3	13.26185
OVA_365_M1	Metastasis	chr19	2750	271	156855	12359265	Loss	0.854003	1.907418	p13.2-p13.3	12.20241
OVA_048_M1	Metastasis	chr19	1578	156	243011	9071464	Del	0.625824	3.962486	p13.2-p13.3	8.828453
OVA_048_M2	Metastasis	chr19	1549	154	243011	9026318	Del	0.405007	3.72502	p13.2-p13.3	8.783307
OVA_048_M3	Metastasis	chr19	621	72	4844785	9028373	Del	0.59385	3.387584	p13.2-p13.3	4.183588
OVA_003_C1	Primary	chr19	687	88	5561075	8973462	Gain	0.852911	1.812048	p13.2-p13.3	3.412387
OVA_003_C2	Primary	chr19	672	86	5561075	8670528	Del	0.709607	3.665802	p13.2-p13.3	3.109453
OVA_003_C3	Primary	chr19	675	87	5561075	8808373	Gain	0.820093	1.828085	p13.2-p13.3	3.247298
OVA_013_M1	Metastasis	chr12	1018	105	90681	10191746	Amp	0.621947	2.883383	p13.2-p13.33	10.10107
OVA_048_M2	Metastasis	chr5	812	79	143197	37239884	Del	0.405007	3.72502	p13.2-p15.33	37.09669
OVA_048_C3	Primary	chr5	813	79	143235	37247684	Gain	0.715645	1.786574	p13.2-p15.33	37.10445
OVA_048_M1	Metastasis	chr5	811	79	143235	37227678	Del	0.625824	3.962486	p13.2-p15.33	37.08444
OVA_048_M3	Metastasis	chr5	777	77	143235	36684018	Loss	0.59385	3.387584	p13.2-p15.33	36.54078
OVA_048_C2	Primary	chr5	813	79	143239	37247684	Amp	0.736255	1.845483	p13.2-p15.33	37.10445
OVA_047_CO	Contralateral ovary	chr9	1511	120	117720	37769944	Gain	0.55613	2.930893	p13.2-p24.3	37.65222
OVA_047_M1	Metastasis	chr1	1432	124	82302600	1.14E+08	Loss	0.624347	2.86771	p13.2-p31.1	31.95332
OVA_003_C2	Primary	chr12	481	38	90681	6428110	Del	0.709607	3.665802	p13.31-p13.33	6.337429
OVA_047_M1	Metastasis	chr12	383	43	93432	5021198	Loss	0.624347	2.86771	p13.32-p13.33	4.927766
OVA_047_M1	Metastasis	chr17	22	3	6157	426133	Loss	0.624347	2.86771	p13.3-p13.3	0.419976
OVA_047_M3	Metastasis	chr17	22	3	6157	426045	Loss	0.605088	2.815208	p13.3-p13.3	0.419888
OVA_047_C2	Primary	chr16	1278	136	62417	4412598	Del	0.575254	2.829045	p13.3-p13.3	4.350181
OVA_047_CO	Contralateral ovary	chr16	1275	136	62417	4412008	Del	0.55613	2.930893	p13.3-p13.3	4.349591
OVA_047_M2	Metastasis	chr16	265	36	62417	767453	Del	0.599725	2.678568	p13.3-p13.3	0.705036
OVA_047_M3	Metastasis	chr16	278	41	62417	776239	Del	0.605088	2.815208	p13.3-p13.3	0.713822
OVA_047_C1	Primary	chr16	1284	139	62444	4431202	Del	0.576016	2.862818	p13.3-p13.3	4.368758
OVA_047_M1	Metastasis	chr16	266	35	62464	767456	Del	0.624347	2.86771	p13.3-p13.3	0.704992
OVA_013_C3	Primary	chr16	657	89	62965	2225971	Del	0.75175	3.001491	p13.3-p13.3	2.163006
OVA_013_M1	Metastasis	chr16	671	89	62965	2258752	Del	0.621947	2.883383	p13.3-p13.3	2.195787
OVA_048_M1	Metastasis	chr16	155	17	85561	683909	Del	0.625824	3.962486	p13.3-p13.3	0.598348
OVA_048_M2	Metastasis	chr16	155	17	85561	683909	Del	0.405007	3.72502	p13.3-p13.3	0.598348
OVA_048_M3	Metastasis	chr16	155	17	85561	683909	Del	0.59385	3.387584	p13.3-p13.3	0.598348
OVA_003_C2	Primary	chr19	1223	124	156855	5456450	Del	0.709607	3.665802	p13.3-p13.3	5.299595
OVA_003_C3	Primary	chr19	1233	125	156855	5456450	Loss	0.820093	1.828085	p13.3-p13.3	5.299595

Sample	Tissue type	chrom	num.mark	nhet	start	end	Classificati	Purity	Ploidy	Cyto	Size_Mb
OVA_013_C1	Primary	chr19	13	0	156855	336102	Del	0.701164	3.125184	p13.3-p13.3	0.179247
OVA_013_C2	Primary	chr19	972	109	156855	5286040	Del	0.755564	3.14838	p13.3-p13.3	5.129185
OVA_013_M1	Metastasis	chr19	969	108	156855	5286119	Del	0.621947	2.883383	p13.3-p13.3	5.129264
OVA_013_C3	Primary	chr19	971	109	156928	5286040	Del	0.75175	3.001491	p13.3-p13.3	5.129112
OVA_047_C1	Primary	chr19	349	57	156928	1358263	Del	0.576016	2.862818	p13.3-p13.3	1.201335
OVA_047_C2	Primary	chr19	349	56	156928	1357082	Del	0.575254	2.829045	p13.3-p13.3	1.200154
OVA_047_CO	Contralateral ovary	chr19	346	56	156928	1356485	Del	0.55613	2.930893	p13.3-p13.3	1.199557
OVA_048_M3	Metastasis	chr19	380	34	243011	1796096	Del	0.59385	3.387584	p13.3-p13.3	1.553085
OVA_013_C1	Primary	chr19	961	110	362283	5260933	Del	0.701164	3.125184	p13.3-p13.3	4.89865
OVA_047_M1	Metastasis	chr16	725	76	771750	2987315	Loss	0.624347	2.86771	p13.3-p13.3	2.215565
OVA_047_M2	Metastasis	chr16	727	76	771750	2998702	Loss	0.599725	2.678568	p13.3-p13.3	2.226952
OVA_047_M3	Metastasis	chr16	716	71	776399	3016177	Loss	0.605088	2.815208	p13.3-p13.3	2.239778
OVA_048_M3	Metastasis	chr19	118	5	1796883	2255185	Loss	0.59385	3.387584	p13.3-p13.3	0.458302
OVA_048_M3	Metastasis	chr19	296	29	2271587	4182622	Del	0.59385	3.387584	p13.3-p13.3	1.911035
OVA_048_M3	Metastasis	chr19	135	15	4199839	4839274	Loss	0.59385	3.387584	p13.3-p13.3	0.639435
OVA_013_C3	Primary	chr5	713	52	140636	32444286	Gain	0.75175	3.001491	p13.3-p15.33	32.30365
OVA_013_C2	Primary	chr5	729	55	140714	33162462	Gain	0.755564	3.14838	p13.3-p15.33	33.02175
OVA_013_M1	Metastasis	chr5	713	52	140714	32444286	Gain	0.621947	2.883383	p13.3-p15.33	32.30357
OVA_013_C1	Primary	chr5	712	51	140716	32417784	Gain	0.701164	3.125184	p13.3-p15.33	32.27707
OVA_048_M3	Metastasis	chr2	1193	101	32698159	70528827	Del	0.59385	3.387584	p13.3-p22.3	37.83067
OVA_048_C1	Primary	chr9	941	67	3271018	35737707	Gain	0.682521	1.843999	p13.3-p24.2	32.46669
OVA_047_M2	Metastasis	chr9	975	64	117720	33566287	Loss	0.599725	2.678568	p13.3-p24.3	33.44857
OVA_047_M3	Metastasis	chr9	972	64	117720	33548175	Loss	0.605088	2.815208	p13.3-p24.3	33.43046
OVA_048_M1	Metastasis	chr2	2423	220	45611	70528827	Del	0.625824	3.962486	p13.3-p25.3	70.48322
OVA_003_C1	Primary	chr20	474	46	76700	4167497	Amp	0.852911	1.812048	p13-p13	4.090797
OVA_003_M1	Metastasis	chr20	476	46	76915	4202649	Gain	0.552447	1.852543	p13-p13	4.125734
OVA_003_C2	Primary	chr20	477	46	76920	4228473	Loss	0.709607	3.665802	p13-p13	4.151553
OVA_003_M2	Metastasis	chr20	460	46	76920	3928844	Gain	0.439877	1.867794	p13-p13	3.851924
OVA_003_C3	Primary	chr20	476	46	76934	4202574	Gain	0.820093	1.828085	p13-p13	4.12564
OVA_003_M3	Metastasis	chr20	472	46	76934	4163513	Gain	0.574876	1.865189	p13-p13	4.086579
OVA_047_C2	Primary	chr11	370	24	20181741	34527181	Loss	0.575254	2.829045	p13-p15.1	14.34544
OVA_047_C2	Primary	chr4	740	52	10085300	42067235	Del	0.575254	2.829045	p13-p16.1	31.98194
OVA_047_CO	Contralateral ovary	chr7	570	53	23854821	37924888	Loss	0.55613	2.930893	p14.1-p15.3	14.07007
OVA_003_C2	Primary	chr11	380	42	17482175	26705310	Del	0.709607	3.665802	p14.2-p15.1	9.223135
OVA_047_C1	Primary	chr7	510	51	26217669	37172863	Loss	0.576016	2.862818	p14.2-p15.2	10.95519
OVA_047_C2	Primary	chr7	556	52	23826216	37172727	Loss	0.575254	2.829045	p14.2-p15.3	13.34651
OVA_003_C3	Primary	chr11	2374	293	179361	27016087	Loss	0.820093	1.828085	p14.2-p15.5	26.83673
OVA_003_C2	Primary	chr3	27	2	57312639	57475381	Del	0.709607	3.665802	p14.3-p14.3	0.162742
OVA_003_C2	Primary	chr3	2022	181	40498904	57311841	Del	0.709607	3.665802	p14.3-p22.1	16.81294
OVA_047_M2	Metastasis	chr3	2046	161	40503506	58318751	Loss	0.599725	2.678568	p14.3-p22.1	17.81525
OVA_047_M1	Metastasis	chr3	2200	173	38592928	58303069	Loss	0.624347	2.86771	p14.3-p22.2	19.71014
OVA_047_M3	Metastasis	chr3	2205	175	38593015	58382846	Loss	0.605088	2.815208	p14.3-p22.2	19.78983
OVA_003_C1	Primary	chr3	3400	308	10219508	57484251	Loss	0.852911	1.812048	p14.3-p25.3	47.26474
OVA_003_C3	Primary	chr3	3410	308	10219508	57484251	Loss	0.820093	1.828085	p14.3-p25.3	47.26474
OVA_047_C1	Primary	chr11	331	36	17333527	20181605	Gain	0.576016	2.862818	p15.1-p15.1	2.848078
OVA_047_C2	Primary	chr11	330	36	17333527	20180896	Gain	0.575254	2.829045	p15.1-p15.1	2.847369
OVA_047_CO	Contralateral ovary	chr11	331	36	17333527	20181605	Gain	0.55613	2.930893	p15.1-p15.1	2.848078
OVA_048_M1	Metastasis	chr4	106	9	22389632	30498953	Del	0.625824	3.962486	p15.1-p15.2	8.109321
OVA_048_M2	Metastasis	chr4	104	9	22389632	30279417	Del	0.405007	3.72502	p15.1-p15.2	7.889785
OVA_048_M3	Metastasis	chr4	110	9	22389632	30725272	Del	0.59385	3.387584	p15.1-p15.2	8.33564
OVA_047_C2	Primary	chr11	202	13	11974262	17191019	Loss	0.575254	2.829045	p15.1-p15.3	5.216757
OVA_047_CO	Contralateral ovary	chr11	197	13	12023773	17191019	Loss	0.55613	2.930893	p15.1-p15.3	5.167246
OVA_003_C2	Primary	chr11	906	107	6232336	17464758	Del	0.709607	3.665802	p15.1-p15.4	11.23242
OVA_365_C2	Primary	chr5	576	65	140532	14397238	Gain	0.629033	1.960121	p15.2-p15.33	14.25671
OVA_003_C1	Primary	chr11	760	95	6340416	14865551	Loss	0.852911	1.812048	p15.2-p15.4	8.525135
OVA_003_C2	Primary	chr4	489	61	6718124	21466300	Del	0.709607	3.665802	p15.2-p16.1	14.74818
OVA_003_C3	Primary	chr4	554	67	6858935	25162082	Loss	0.820093	1.828085	p15.2-p16.1	18.30315
OVA_003_M3	Metastasis	chr7	604	59	7484105	27186982	Gain	0.574876	1.865189	p15.2-p21.3	19.70288
OVA_003_M2	Metastasis	chr7	587	59	7491908	27135338	Gain	0.439877	1.867794	p15.2-p21.3	19.64343
OVA_047_C1	Primary	chr7	1206	101	193254	26192667	Loss	0.576016	2.862818	p15.2-p22.3	25.99941
OVA_013_M1	Metastasis	chr7	1168	105	331086	27702304	Del	0.621947	2.883383	p15.2-p22.3	27.37122
OVA_048_M2	Metastasis	chr4	893	105	53218	20760520	Del	0.405007	3.72502	p15.31-p16.3	20.7073
OVA_048_C3	Primary	chr4	893	105	53430	20760520	Gain	0.715645	1.786574	p15.31-p16.3	20.70709
OVA_048_M1	Metastasis	chr4	893	105	53430	20760520	Del	0.625824	3.962486	p15.31-p16.3	20.70709
OVA_048_M3	Metastasis	chr4	893	105	53430	20760520	Loss	0.59385	3.387584	p15.31-p16.3	20.70709
OVA_013_C1	Primary	chr4	35	3	13604954	15480785	Del	0.701164	3.125184	p15.32-p15.33	1.875831

Sample	Tissue type	chrom	num.mark	nhet	start	end	Classificati	Purity	Ploidy	Cyto	Size_Mb
OVA_013_M1	Metastasis	chr4	39	4	13605290	15518271	Loss	0.621947	2.883383	p15.32-p15.33	1.912981
OVA_047_M3	Metastasis	chr5	175	17	732554	3610430	Gain	0.605088	2.815208	p15.33-p15.33	2.877876
OVA_047_CO	Contralateral ovary	chr11	14	0	11941964	12020270	Loss	0.55613	2.930893	p15.3-p15.3	0.078306
OVA_047_C1	Primary	chr11	1694	121	134550	11924337	Del	0.576016	2.862818	p15.3-p15.5	11.78979
OVA_047_C2	Primary	chr11	1704	121	134550	11971853	Del	0.575254	2.829045	p15.3-p15.5	11.8373
OVA_047_CO	Contralateral ovary	chr11	1690	120	134550	11924337	Del	0.55613	2.930893	p15.3-p15.5	11.78979
OVA_003_C1	Primary	chr7	539	57	7480382	24905654	Amp	0.852911	1.812048	p15.3-p21.3	17.42527
OVA_003_C2	Primary	chr7	539	57	7480382	24905654	Loss	0.709607	3.665802	p15.3-p21.3	17.42527
OVA_003_C3	Primary	chr7	539	57	7480382	24905654	Amp	0.820093	1.828085	p15.3-p21.3	17.42527
OVA_003_M1	Metastasis	chr7	543	57	7484105	25181912	Gain	0.552447	1.852543	p15.3-p21.3	17.69781
OVA_047_C2	Primary	chr7	1157	100	193245	23810759	Del	0.575254	2.829045	p15.3-p22.3	23.61751
OVA_047_CO	Contralateral ovary	chr7	1145	100	538345	23827618	Del	0.55613	2.930893	p15.3-p22.3	23.28927
OVA_003_C2	Primary	chr11	1074	144	179460	6231731	Del	0.709607	3.665802	p15.4-p15.5	6.052271
OVA_365_C2	Primary	chr11	132	21	134550	502115	Loss	0.629033	1.960121	p15.5-p15.5	0.367565
OVA_013_C1	Primary	chr11	59	8	179361	320848	Del	0.701164	3.125184	p15.5-p15.5	0.141487
OVA_013_C2	Primary	chr11	61	8	179361	373282	Del	0.755564	3.14838	p15.5-p15.5	0.193921
OVA_013_C3	Primary	chr11	61	8	179361	373220	Del	0.75175	3.001491	p15.5-p15.5	0.193859
OVA_013_M1	Metastasis	chr11	58	8	179361	320772	Del	0.621947	2.883383	p15.5-p15.5	0.141411
OVA_048_M2	Metastasis	chr11	247	40	193016	1009881	Del	0.405007	3.72502	p15.5-p15.5	0.816865
OVA_048_M3	Metastasis	chr11	248	40	193045	1011577	Del	0.59385	3.387584	p15.5-p15.5	0.818532
OVA_013_M1	Metastasis	chr11	285	36	372157	1246801	Loss	0.621947	2.883383	p15.5-p15.5	0.874644
OVA_013_C1	Primary	chr11	312	38	372225	1267502	Del	0.701164	3.125184	p15.5-p15.5	0.895277
OVA_013_C3	Primary	chr11	367	40	373492	1780168	Del	0.75175	3.001491	p15.5-p15.5	1.406676
OVA_013_C2	Primary	chr11	365	40	373499	1775095	Del	0.755564	3.14838	p15.5-p15.5	1.401596
OVA_048_M2	Metastasis	chr11	83	11	1011490	1271214	Del	0.405007	3.72502	p15.5-p15.5	0.259724
OVA_048_M3	Metastasis	chr11	82	11	1013922	1271181	Del	0.59385	3.387584	p15.5-p15.5	0.257259
OVA_013_M1	Metastasis	chr2	643	37	44021556	63849806	Loss	0.621947	2.883383	p15-p21	19.82825
OVA_047_C2	Primary	chr4	904	96	53430	10083157	Del	0.575254	2.829045	p16.1-p16.3	10.02973
OVA_047_M1	Metastasis	chr4	791	90	502741	8391525	Loss	0.624347	2.86771	p16.1-p16.3	7.888784
OVA_047_M3	Metastasis	chr4	849	94	517461	9892424	Loss	0.605088	2.815208	p16.1-p16.3	9.374963
OVA_003_C2	Primary	chr4	395	41	2230858	6711290	Del	0.709607	3.665802	p16.1-p16.3	4.480432
OVA_003_C1	Primary	chr4	269	35	53218	1804813	Amp	0.852911	1.812048	p16.3-p16.3	1.751595
OVA_003_C3	Primary	chr4	263	35	53218	1795796	Amp	0.820093	1.828085	p16.3-p16.3	1.742578
OVA_013_C1	Primary	chr4	30	3	53218	494188	Del	0.701164	3.125184	p16.3-p16.3	0.44097
OVA_013_C2	Primary	chr4	30	3	53218	494188	Del	0.755564	3.14838	p16.3-p16.3	0.44097
OVA_047_CO	Contralateral ovary	chr4	35	0	53218	500609	Del	0.55613	2.930893	p16.3-p16.3	0.447391
OVA_047_M3	Metastasis	chr4	38	0	53218	515697	Loss	0.605088	2.815208	p16.3-p16.3	0.462479
OVA_003_C2	Primary	chr4	324	36	53428	2210075	Loss	0.709607	3.665802	p16.3-p16.3	2.156647
OVA_013_M1	Metastasis	chr4	30	3	53428	494188	Loss	0.621947	2.883383	p16.3-p16.3	0.44076
OVA_047_C1	Primary	chr4	18	0	53428	337598	Del	0.576016	2.862818	p16.3-p16.3	0.28417
OVA_013_C3	Primary	chr4	30	3	53430	494188	Del	0.75175	3.001491	p16.3-p16.3	0.440758
OVA_047_M1	Metastasis	chr4	36	0	53430	501134	Loss	0.624347	2.86771	p16.3-p16.3	0.447704
OVA_047_M2	Metastasis	chr4	41	0	53430	520853	Loss	0.599725	2.678568	p16.3-p16.3	0.467423
OVA_047_C1	Primary	chr4	89	6	343826	891983	Loss	0.576016	2.862818	p16.3-p16.3	0.548157
OVA_013_M1	Metastasis	chr4	414	17	494832	4249924	Loss	0.621947	2.883383	p16.3-p16.3	3.755092
OVA_013_C1	Primary	chr4	413	17	494998	4249855	Loss	0.701164	3.125184	p16.3-p16.3	3.754857
OVA_013_C2	Primary	chr4	412	17	494998	4249855	Loss	0.755564	3.14838	p16.3-p16.3	3.754857
OVA_013_C3	Primary	chr4	413	17	494998	4249855	Loss	0.75175	3.001491	p16.3-p16.3	3.754857
OVA_047_CO	Contralateral ovary	chr4	67	5	501115	875887	Loss	0.55613	2.930893	p16.3-p16.3	0.374772
OVA_365_CO	Contralateral ovary	chr2	2123	197	41509	48033514	Gain	0.451025	1.890627	p16.3-p25.3	47.99201
OVA_365_M1	Metastasis	chr2	2125	198	41509	48059836	Amp	0.854003	1.907418	p16.3-p25.3	48.01833
OVA_365_M2	Metastasis	chr2	2138	199	41509	48707153	Gain	0.387458	1.893344	p16.3-p25.3	48.66564
OVA_365_C2	Primary	chr2	2121	197	41602	48030838	Gain	0.629033	1.960121	p16.3-p25.3	47.98924
OVA_365_M3	Metastasis	chr2	2127	198	41602	48065989	Amp	0.827915	1.890476	p16.3-p25.3	48.02439
OVA_013_C3	Primary	chr6	541	24	37622308	43592372	Gain	0.75175	3.001491	p21.1-p21.2	5.970064
OVA_013_M1	Metastasis	chr6	528	23	38560449	43591622	Gain	0.621947	2.883383	p21.1-p21.2	5.031173
OVA_013_C1	Primary	chr6	503	19	38746263	43555183	Gain	0.701164	3.125184	p21.1-p21.2	4.80892
OVA_047_C1	Primary	chr6	1224	143	32606824	43154064	Loss	0.576016	2.862818	p21.1-p21.32	10.54724
OVA_048_M2	Metastasis	chr7	726	64	193222	20441504	Del	0.405007	3.72502	p21.1-p22.3	20.24828
OVA_048_M1	Metastasis	chr7	702	63	193254	19015508	Del	0.625824	3.962486	p21.1-p22.3	18.82225
OVA_048_M3	Metastasis	chr7	704	63	193254	19015508	Del	0.59385	3.387584	p21.1-p22.3	18.82225
OVA_047_M3	Metastasis	chr6	2598	408	18399837	41658566	Loss	0.605088	2.815208	p21.1-p22.3	23.25873
OVA_047_M1	Metastasis	chr6	2575	410	20546697	41658889	Loss	0.624347	2.86771	p21.1-p22.3	21.11219
OVA_003_C2	Primary	chrX	875	58	2700027	31986534	Del	0.709607	3.665802	p21.1-p22.33	29.28651
OVA_013_C1	Primary	chrX	832	42	2700027	33229437	Loss	0.701164	3.125184	p21.1-p22.33	30.52941
OVA_013_C2	Primary	chrX	836	43	2700027	34150150	Loss	0.755564	3.14838	p21.1-p22.33	31.45012

Sample	Tissue type	chrom	num.mark	nhet	start	end	Classificati	Purity	Ploidy	Cyto	Size_Mb
OVA_013_C3	Primary	chrX	832	42	2700027	33229437	Loss	0.75175	3.001491	p21.1-p22.33	30.52941
OVA_013_M1	Metastasis	chrX	830	42	2700027	32827566	Loss	0.621947	2.883383	p21.1-p22.33	30.12754
OVA_048_M1	Metastasis	chr9	537	31	2838419	32408521	Del	0.625824	3.962486	p21.1-p24.2	29.5701
OVA_047_M1	Metastasis	chr9	914	64	117720	33167141	Loss	0.624347	2.86771	p21.1-p24.3	33.04942
OVA_048_M3	Metastasis	chr6	2636	383	203548	41739642	Del	0.59385	3.387584	p21.1-p25.3	41.53609
OVA_048_M3	Metastasis	chr3	2777	216	5216113	53856593	Loss	0.59385	3.387584	p21.1-p26.1	48.64048
OVA_048_C1	Primary	chr6	745	88	32373064	39869783	Gain	0.682521	1.843999	p21.2-p21.32	7.496719
OVA_048_M2	Metastasis	chr6	508	41	33399656	39869783	Del	0.405007	3.72502	p21.2-p21.32	6.470127
OVA_013_C1	Primary	chr6	1404	215	30529209	37614135	Loss	0.701164	3.125184	p21.2-p21.33	7.084926
OVA_013_C2	Primary	chr6	1401	215	30529209	37606305	Loss	0.755564	3.14838	p21.2-p21.33	7.077096
OVA_013_C3	Primary	chr6	1407	215	30529209	37619896	Loss	0.75175	3.001491	p21.2-p21.33	7.090687
OVA_003_C2	Primary	chr6	1555	256	30688427	38841101	Del	0.709607	3.665802	p21.2-p21.33	8.152674
OVA_048_C3	Primary	chr6	1172	138	31524507	39869783	Gain	0.715645	1.786574	p21.2-p21.33	8.345276
OVA_048_M3	Metastasis	chr8	579	65	11422045	24813751	Loss	0.59385	3.387584	p21.2-p23.1	13.39171
OVA_003_C3	Primary	chr9	483	42	8319957	27229141	Loss	0.820093	1.828085	p21.2-p24.1	18.90918
OVA_003_C1	Primary	chr9	478	42	8331644	27229141	Loss	0.852911	1.812048	p21.2-p24.1	18.8975
OVA_003_C2	Primary	chr9	471	41	8437141	27229141	Del	0.709607	3.665802	p21.2-p24.1	18.792
OVA_048_M1	Metastasis	chr6	2564	376	203548	39847270	Del	0.625824	3.962486	p21.2-p25.3	39.64372
OVA_048_C2	Primary	chr3	217	13	48956257	49833418	Gain	0.736255	1.845483	p21.31-p21.31	0.877161
OVA_048_C3	Primary	chr3	215	12	48999189	49833418	Amp	0.715645	1.786574	p21.31-p21.31	0.834229
OVA_048_C1	Primary	chr3	204	12	49012469	49764879	Gain	0.682521	1.843999	p21.31-p21.31	0.75241
OVA_365_CO	Contralateral ovary	chr6	1351	246	30545627	35473370	Gain	0.451025	1.890627	p21.31-p21.33	4.927743
OVA_048_M2	Metastasis	chr6	1405	282	25973271	33393525	Del	0.405007	3.72502	p21.32-p22.2	7.420254
OVA_048_C1	Primary	chr6	210	17	31624371	31940298	Gain	0.682521	1.843999	p21.33-p21.33	0.315927
OVA_013_M1	Metastasis	chr6	160	62	29640729	30529209	Del	0.621947	2.883383	p21.33-p22.1	0.88848
OVA_013_C1	Primary	chr6	156	63	29644668	30525107	Del	0.701164	3.125184	p21.33-p22.1	0.880439
OVA_013_C2	Primary	chr6	157	62	29644668	30525107	Del	0.755564	3.14838	p21.33-p22.1	0.880439
OVA_013_C3	Primary	chr6	155	62	29691385	30525137	Del	0.75175	3.001491	p21.33-p22.1	0.833752
OVA_003_C2	Primary	chr6	206	28	29911727	30682872	Loss	0.709607	3.665802	p21.33-p22.1	0.771145
OVA_003_M3	Metastasis	chr6	923	129	17850618	30682872	Gain	0.574876	1.865189	p21.33-p22.3	12.83225
OVA_003_C1	Primary	chr6	919	129	17856244	30682872	Amp	0.852911	1.812048	p21.33-p22.3	12.82663
OVA_003_C3	Primary	chr6	914	129	18121964	30682872	Gain	0.820093	1.828085	p21.33-p22.3	12.56091
OVA_003_M1	Metastasis	chr6	901	128	18197915	30682872	Gain	0.552447	1.852543	p21.33-p22.3	12.48496
OVA_365_CO	Contralateral ovary	chr6	1427	193	532572	30545297	Gain	0.451025	1.890627	p21.33-p25.3	30.01273
OVA_003_C2	Primary	chr7	711	75	193222	7476178	Del	0.709607	3.665802	p21.3-p22.3	7.282956
OVA_013_C2	Primary	chr1	765	67	75172001	96488135	Loss	0.755564	3.14838	p21.3-p31.1	21.31613
OVA_013_C3	Primary	chr1	752	66	75602922	95712458	Loss	0.75175	3.001491	p21.3-p31.1	20.10954
OVA_013_C1	Primary	chr1	753	66	75622616	96488206	Loss	0.701164	3.125184	p21.3-p31.1	20.86559
OVA_013_M1	Metastasis	chr2	94	3	42396934	44019930	Loss	0.621947	2.883383	p21-p21	1.622996
OVA_047_C1	Primary	chr2	943	82	27372075	44052035	Del	0.576016	2.862818	p21-p23.3	16.67996
OVA_047_M1	Metastasis	chr2	1708	141	10799319	44933375	Loss	0.624347	2.86771	p21-p25.1	34.13406
OVA_047_M2	Metastasis	chr2	2019	169	45521	44933375	Loss	0.599725	2.678568	p21-p25.3	44.88785
OVA_047_M3	Metastasis	chr2	2023	170	45611	44999281	Loss	0.605088	2.815208	p21-p25.3	44.95367
OVA_013_M1	Metastasis	chr2	1693	114	1982850	42284843	Loss	0.621947	2.883383	p21-p25.3	40.30199
OVA_013_C1	Primary	chr2	1690	114	1982877	42396722	Del	0.701164	3.125184	p21-p25.3	40.41385
OVA_013_C2	Primary	chr2	1690	114	1982877	42396722	Del	0.755564	3.14838	p21-p25.3	40.41385
OVA_013_C3	Primary	chr2	1693	114	1982877	42396934	Del	0.75175	3.001491	p21-p25.3	40.41406
OVA_003_C2	Primary	chr6	17	6	29855425	29911360	Del	0.709607	3.665802	p22.1-p22.1	0.055935
OVA_013_C2	Primary	chr6	371	45	25925839	29641156	Loss	0.755564	3.14838	p22.1-p22.2	3.715317
OVA_047_M3	Metastasis	chr7	617	44	193245	6628514	Loss	0.605088	2.815208	p22.1-p22.3	6.435269
OVA_003_C2	Primary	chr6	689	95	18122157	29798419	Loss	0.709607	3.665802	p22.1-p22.3	11.67626
OVA_047_M2	Metastasis	chr6	484	45	18122416	27861682	Loss	0.599725	2.678568	p22.1-p22.3	9.739266
OVA_047_M2	Metastasis	chr3	416	22	35730750	40500231	Del	0.599725	2.678568	p22.1-p22.3	4.769481
OVA_047_CO	Contralateral ovary	chr3	899	59	15628040	41288537	Loss	0.55613	2.930893	p22.1-p25.1	25.6605
OVA_048_C2	Primary	chr2	1486	142	45521	39108547	Loss	0.736255	1.845483	p22.1-p25.3	39.06303
OVA_047_CO	Contralateral ovary	chr6	1117	106	203666	28104878	Loss	0.55613	2.930893	p22.1-p25.3	27.90121
OVA_003_C2	Primary	chr3	1351	126	10219508	40468896	Del	0.709607	3.665802	p22.1-p25.3	30.24939
OVA_047_C1	Primary	chr3	1563	116	8556617	41278039	Loss	0.576016	2.862818	p22.1-p26.1	32.72142
OVA_047_C2	Primary	chr3	1581	120	8607124	42218299	Del	0.575254	2.829045	p22.1-p26.1	33.61118
OVA_003_C2	Primary	chr1	1031	96	66827527	94520708	Del	0.709607	3.665802	p22.1-p31.3	27.69318
OVA_013_C3	Primary	chr3	15	0	39225767	39230559	Loss	0.75175	3.001491	p22.2-p22.2	0.004792
OVA_013_C1	Primary	chr3	13	0	39226171	39230691	Loss	0.701164	3.125184	p22.2-p22.2	0.00452
OVA_047_M3	Metastasis	chr3	276	12	33853496	38592406	Del	0.605088	2.815208	p22.2-p22.3	4.73891
OVA_047_M1	Metastasis	chr3	263	10	35730650	38592406	Del	0.624347	2.86771	p22.2-p22.3	2.861756
OVA_013_C3	Primary	chr3	708	25	16926766	39188182	Del	0.75175	3.001491	p22.2-p24.3	22.26142
OVA_013_M1	Metastasis	chr3	710	24	16926766	39225585	Del	0.621947	2.883383	p22.2-p24.3	22.29882

Sample	Tissue type	chrom	num.mark	nhet	start	end	Classificati	Purity	Ploidy	Cyto	Size_Mb
OVA_013_C2	Primary	chr3	704	24	16926790	39188182	Del	0.755564	3.14838	p22.2-p24.3	22.26139
OVA_013_C1	Primary	chr3	776	28	15631024	39225835	Del	0.701164	3.125184	p22.2-p25.1	23.59481
OVA_048_M2	Metastasis	chr6	661	57	203548	25972179	Del	0.405007	3.72502	p22.2-p25.3	25.76863
OVA_047_C2	Primary	chrX	126	5	2700027	8507773	Del	0.575254	2.829045	p22.31-p22.33	5.807746
OVA_047_M2	Metastasis	chrX	12	0	2700089	2798969	Loss	0.599725	2.678568	p22.33-p22.33	0.09888
OVA_048_M3	Metastasis	chrX	30	1	2700089	2936622	Del	0.59385	3.387584	p22.33-p22.33	0.236533
OVA_013_M1	Metastasis	chr7	13	0	193222	330786	Del	0.621947	2.883383	p22.3-p22.3	0.137564
OVA_365_M2	Metastasis	chr7	285	34	193245	2515613	Gain	0.387458	1.893344	p22.3-p22.3	2.322368
OVA_047_CO	Contralateral ovary	chr7	16	0	193254	331427	Loss	0.55613	2.930893	p22.3-p22.3	0.138173
OVA_365_CO	Contralateral ovary	chr7	75	10	193254	923935	Gain	0.451025	1.890627	p22.3-p22.3	0.730681
OVA_047_M3	Metastasis	chr6	57	9	17669216	18387953	Loss	0.605088	2.815208	p22.3-p22.3	0.718737
OVA_048_M3	Metastasis	chr2	1230	119	45495	32696048	Del	0.59385	3.387584	p22.3-p25.3	32.65055
OVA_047_M2	Metastasis	chr6	620	62	203548	18122157	Loss	0.599725	2.678568	p22.3-p25.3	17.91861
OVA_047_C1	Primary	chr6	595	59	203666	17706484	Loss	0.576016	2.862818	p22.3-p25.3	17.50282
OVA_047_M3	Metastasis	chr6	589	58	203666	17665479	Del	0.605088	2.815208	p22.3-p25.3	17.46181
OVA_047_M1	Metastasis	chr6	671	68	203699	20490390	Loss	0.624347	2.86771	p22.3-p25.3	20.28669
OVA_003_C2	Primary	chr6	609	84	1961203	17987562	Del	0.709607	3.665802	p22.3-p25.3	16.02636
OVA_047_M3	Metastasis	chr3	1144	91	5024771	33840375	Loss	0.605088	2.815208	p22.3-p26.1	28.8156
OVA_047_M2	Metastasis	chr3	1157	93	5164135	33908106	Loss	0.599725	2.678568	p22.3-p26.1	28.74397
OVA_047_M1	Metastasis	chr3	1156	93	5212088	33908020	Loss	0.624347	2.86771	p22.3-p26.1	28.69593
OVA_365_M3	Metastasis	chr1	499	42	67786050	87814455	Loss	0.827915	1.890476	p22.3-p31.3	20.02841
OVA_365_M1	Metastasis	chr1	494	41	67786088	87814481	Loss	0.854003	1.907418	p22.3-p31.3	20.02839
OVA_365_C2	Primary	chr8	477	54	163553	15095224	Loss	0.629033	1.960121	p22-p23.3	14.93167
OVA_048_C2	Primary	chr8	245	30	182785	11418766	Gain	0.736255	1.845483	p23.1-p23.3	11.23598
OVA_048_M2	Metastasis	chr8	248	31	182785	11422045	Del	0.405007	3.72502	p23.1-p23.3	11.23926
OVA_013_C1	Primary	chr8	142	19	163421	4495043	Del	0.701164	3.125184	p23.2-p23.3	4.331622
OVA_013_C3	Primary	chr8	142	19	163421	4495043	Del	0.75175	3.001491	p23.2-p23.3	4.331622
OVA_013_M1	Metastasis	chr8	141	19	163438	4494943	Del	0.621947	2.883383	p23.2-p23.3	4.331505
OVA_013_C2	Primary	chr8	142	19	163461	4494943	Del	0.755564	3.14838	p23.2-p23.3	4.331482
OVA_047_C2	Primary	chr2	19	0	27587724	27598615	Del	0.575254	2.829045	p23.3-p23.3	0.010891
OVA_047_C2	Primary	chr2	1091	97	45521	27587447	Loss	0.575254	2.829045	p23.3-p25.3	27.54193
OVA_047_C1	Primary	chr2	1013	85	45611	27360983	Loss	0.576016	2.862818	p23.3-p25.3	27.31537
OVA_013_C1	Primary	chr6	186	12	7727525	13612078	Gain	0.701164	3.125184	p23-p24.3	5.884553
OVA_013_M1	Metastasis	chr6	192	12	7727525	13632738	Gain	0.621947	2.883383	p23-p24.3	5.905213
OVA_013_C3	Primary	chr6	186	12	7727668	13612078	Gain	0.75175	3.001491	p23-p24.3	5.88441
OVA_003_C2	Primary	chr9	141	9	5126795	8436703	Del	0.709607	3.665802	p24.1-p24.1	3.309908
OVA_003_C2	Primary	chr9	227	20	117666	5126443	Del	0.709607	3.665802	p24.1-p24.3	5.008777
OVA_003_C1	Primary	chr9	231	20	117720	5126795	Loss	0.852911	1.812048	p24.1-p24.3	5.009075
OVA_003_C3	Primary	chr9	230	20	117720	5090612	Loss	0.820093	1.828085	p24.1-p24.3	4.972892
OVA_047_CO	Contralateral ovary	chr2	491	53	1946646	20822031	Del	0.55613	2.930893	p24.1-p25.3	18.87539
OVA_048_M1	Metastasis	chr9	109	10	121432	2837211	Del	0.625824	3.962486	p24.2-p24.3	2.715779
OVA_013_C1	Primary	chr6	280	30	203548	7606255	Loss	0.701164	3.125184	p24.3-p25.3	7.402707
OVA_013_C2	Primary	chr6	280	30	203548	7606255	Loss	0.755564	3.14838	p24.3-p25.3	7.402707
OVA_013_C3	Primary	chr6	280	30	203548	7606255	Loss	0.75175	3.001491	p24.3-p25.3	7.402707
OVA_013_C2	Primary	chr3	816	52	239347	16679432	Del	0.755564	3.14838	p24.3-p26.3	16.44009
OVA_013_C3	Primary	chr3	818	52	239347	16679432	Del	0.75175	3.001491	p24.3-p26.3	16.44009
OVA_013_M1	Metastasis	chr3	816	52	239347	16679432	Loss	0.621947	2.883383	p24.3-p26.3	16.44009
OVA_013_C1	Primary	chr3	756	49	239347	15628114	Del	0.701164	3.125184	p25.1-p26.3	15.38877
OVA_003_C2	Primary	chr2	85	8	41509	1946968	Del	0.709607	3.665802	p25.3-p25.3	1.905459
OVA_003_C3	Primary	chr2	85	8	41509	1946968	Gain	0.820093	1.828085	p25.3-p25.3	1.905459
OVA_013_C3	Primary	chr2	75	5	41509	1947030	Loss	0.75175	3.001491	p25.3-p25.3	1.905521
OVA_047_CO	Contralateral ovary	chr2	83	2	45521	1926724	Del	0.55613	2.930893	p25.3-p25.3	1.881203
OVA_365_M3	Metastasis	chr6	15	1	203548	401710	Gain	0.827915	1.890476	p25.3-p25.3	0.198162
OVA_003_C2	Primary	chr6	51	8	203666	1930299	Loss	0.709607	3.665802	p25.3-p25.3	1.726633
OVA_365_M1	Metastasis	chr6	14	3	532572	619293	Amp	0.854003	1.907418	p25.3-p25.3	0.086721
OVA_003_C2	Primary	chr3	427	17	239350	10191638	Del	0.709607	3.665802	p25.3-p26.3	9.952288
OVA_003_C1	Primary	chr3	427	17	239588	10191638	Gain	0.852911	1.812048	p25.3-p26.3	9.95205
OVA_047_C1	Primary	chr3	174	11	239347	8543762	Del	0.576016	2.862818	p26.1-p26.3	8.304415
OVA_048_M3	Metastasis	chr3	106	10	239350	5212088	Del	0.59385	3.387584	p26.1-p26.3	4.972738
OVA_047_C2	Primary	chr3	175	9	239588	8590574	Del	0.575254	2.829045	p26.1-p26.3	8.350986
OVA_047_CO	Contralateral ovary	chr3	174	11	239588	7721997	Del	0.55613	2.930893	p26.1-p26.3	7.482409
OVA_047_M1	Metastasis	chr3	150	10	239588	5164135	Del	0.624347	2.86771	p26.1-p26.3	4.924547
OVA_047_M2	Metastasis	chr3	148	11	239588	5024771	Del	0.599725	2.678568	p26.1-p26.3	4.785183
OVA_047_M3	Metastasis	chr3	146	10	239588	5023172	Del	0.605088	2.815208	p26.1-p26.3	4.783584
OVA_013_C1	Primary	chr1	954	95	50917964	75608854	Del	0.701164	3.125184	p31.1-p32.3	24.69089
OVA_013_C2	Primary	chr1	940	94	50917964	75139139	Del	0.755564	3.14838	p31.1-p32.3	24.22118

Sample	Tissue type	chrom	num.mark	nhet	start	end	Classificati	Purity	Ploidy	Cyto	Size_Mb
OVA_013_M1	Metastasis	chr1	992	98	48699239	75608854	Loss	0.621947	2.883383	p31.1-p33	26.90962
OVA_013_C3	Primary	chr1	972	97	49201984	75596548	Loss	0.75175	3.001491	p31.1-p33	26.39456
OVA_365_M2	Metastasis	chr1	1160	94	44877961	65897557	Gain	0.387458	1.893344	p31.3-p34.1	21.0196
OVA_365_M3	Metastasis	chr1	1073	90	45292173	65339124	Amp	0.827915	1.890476	p31.3-p34.1	20.04695
OVA_365_M1	Metastasis	chr1	1081	90	45292710	65831686	Amp	0.854003	1.907418	p31.3-p34.1	20.53898
OVA_365_C2	Primary	chr1	2401	163	32827082	67672663	Gain	0.629033	1.960121	p31.3-p35.1	34.84558
OVA_365_CO	Contralateral ovary	chr1	2384	160	32827165	67401630	Gain	0.451025	1.890627	p31.3-p35.1	34.57447
OVA_003_C2	Primary	chr1	3186	265	25554029	66821195	Del	0.709607	3.665802	p31.3-p36.11	41.26717
OVA_047_C1	Primary	chr1	3827	329	19200908	67370946	Del	0.576016	2.862818	p31.3-p36.13	48.17004
OVA_047_C2	Primary	chr1	3433	288	19186129	55547068	Del	0.575254	2.829045	p32.3-p36.13	36.36094
OVA_365_M1	Metastasis	chr1	395	35	42693529	45292181	Amp	0.854003	1.907418	p34.1-p34.2	2.598652
OVA_365_M2	Metastasis	chr1	1178	61	32827084	44820469	Amp	0.387458	1.893344	p34.1-p35.1	11.99339
OVA_365_M3	Metastasis	chr1	1244	61	32827165	45291908	Amp	0.827915	1.890476	p34.1-p35.1	12.46474
OVA_365_M1	Metastasis	chr1	848	26	32827084	42660684	Amp	0.854003	1.907418	p34.2-p35.1	9.8336
OVA_013_C1	Primary	chr1	225	18	33116150	36028277	Del	0.701164	3.125184	p34.3-p35.1	2.912127
OVA_013_C3	Primary	chr1	353	20	33116150	36932135	Del	0.75175	3.001491	p34.3-p35.1	3.815985
OVA_013_C2	Primary	chr1	353	20	33116170	36932062	Del	0.755564	3.14838	p34.3-p35.1	3.815892
OVA_013_M1	Metastasis	chr1	2067	171	12941803	36379911	Del	0.621947	2.883383	p34.3-p36.21	23.43811
OVA_013_C1	Primary	chr1	267	13	29563375	33100323	Loss	0.701164	3.125184	p35.1-p35.3	3.536948
OVA_013_C2	Primary	chr1	266	13	29581937	33100323	Loss	0.755564	3.14838	p35.1-p35.3	3.518386
OVA_013_C3	Primary	chr1	266	13	29581937	33100323	Loss	0.75175	3.001491	p35.1-p35.3	3.518386
OVA_365_C2	Primary	chr1	3707	356	13273	32800640	Loss	0.629033	1.960121	p35.1-p36.33	32.78737
OVA_365_M1	Metastasis	chr1	3545	324	13273	32800640	Loss	0.854003	1.907418	p35.1-p36.33	32.78737
OVA_365_M3	Metastasis	chr1	3678	354	13273	32800706	Loss	0.827915	1.890476	p35.1-p36.33	32.78743
OVA_047_M2	Metastasis	chr1	3234	185	13380	31206357	Del	0.599725	2.678568	p35.2-p36.33	31.19298
OVA_047_M3	Metastasis	chr1	3225	181	13453	31186387	Del	0.605088	2.815208	p35.2-p36.33	31.17293
OVA_013_C3	Primary	chr1	1538	140	12919012	29563375	Del	0.75175	3.001491	p35.3-p36.21	16.64436
OVA_013_C1	Primary	chr1	1537	139	12941803	29557224	Del	0.701164	3.125184	p35.3-p36.21	16.61542
OVA_013_C2	Primary	chr1	1624	149	12186157	29563318	Del	0.755564	3.14838	p35.3-p36.22	17.37716
OVA_047_M1	Metastasis	chr1	3220	182	13453	29842928	Del	0.624347	2.86771	p35.3-p36.33	29.82948
OVA_003_C3	Primary	chr1	905	65	17248563	25291010	Gain	0.820093	1.828085	p36.11-p36.13	8.042447
OVA_003_C2	Primary	chr1	906	65	17248607	25551505	Del	0.709607	3.665802	p36.11-p36.13	8.302898
OVA_003_C1	Primary	chr1	2254	193	2938924	25291010	Gain	0.852911	1.812048	p36.11-p36.32	22.35209
OVA_047_CO	Contralateral ovary	chr1	2919	164	905813	27278810	Del	0.55613	2.930893	p36.11-p36.33	26.373
OVA_003_C2	Primary	chr1	64	13	16862502	17198381	Del	0.709607	3.665802	p36.13-p36.13	0.335879
OVA_003_M2	Metastasis	chr1	9	3	19775246	19948453	Loss	0.439877	1.867794	p36.13-p36.13	0.173207
OVA_047_C2	Primary	chr1	560	19	12726169	19184077	Loss	0.575254	2.829045	p36.13-p36.21	6.457908
OVA_047_C1	Primary	chr1	780	21	11561257	19199473	Loss	0.576016	2.862818	p36.13-p36.22	7.638216
OVA_003_C3	Primary	chr1	1247	113	3416294	16817189	Gain	0.820093	1.828085	p36.13-p36.32	13.4009
OVA_003_C2	Primary	chr1	1204	108	3662309	16817189	Del	0.709607	3.665802	p36.13-p36.32	13.15488
OVA_048_C2	Primary	chr1	89	10	12202473	13839735	Amp	0.736255	1.845483	p36.21-p36.22	1.637262
OVA_047_C2	Primary	chr1	1434	64	887471	12711267	Loss	0.575254	2.829045	p36.21-p36.33	11.8238
OVA_047_C1	Primary	chr1	1171	63	976629	11354924	Loss	0.576016	2.862818	p36.22-p36.33	10.3783
OVA_048_M3	Metastasis	chr1	828	96	1580738	12186205	Del	0.59385	3.387584	p36.22-p36.33	10.60547
OVA_003_C1	Primary	chr1	578	25	880466	2938506	Amp	0.852911	1.812048	p36.32-p36.33	2.05804
OVA_003_C3	Primary	chr1	610	26	883918	3415357	Amp	0.820093	1.828085	p36.32-p36.33	2.531439
OVA_003_C2	Primary	chr1	653	31	884094	3659657	Loss	0.709607	3.665802	p36.32-p36.33	2.775563
OVA_047_C1	Primary	chr1	78	1	13380	976135	Del	0.576016	2.862818	p36.33-p36.33	0.962755
OVA_047_C2	Primary	chr1	30	1	13380	886719	Del	0.575254	2.829045	p36.33-p36.33	0.873339
OVA_048_C3	Primary	chr1	124	14	13380	1239368	Gain	0.715645	1.786574	p36.33-p36.33	1.225988
OVA_048_M1	Metastasis	chr1	337	48	13380	2080395	Loss	0.625824	3.962486	p36.33-p36.33	2.067015
OVA_048_M2	Metastasis	chr1	335	48	13380	1991014	Loss	0.405007	3.72502	p36.33-p36.33	1.977634
OVA_003_C2	Primary	chr1	30	4	13453	883699	Del	0.709607	3.665802	p36.33-p36.33	0.870246
OVA_003_C3	Primary	chr1	30	4	13453	883666	Gain	0.820093	1.828085	p36.33-p36.33	0.870213
OVA_047_CO	Contralateral ovary	chr1	52	1	13453	901923	Del	0.55613	2.930893	p36.33-p36.33	0.88847
OVA_048_M3	Metastasis	chr1	257	28	13453	1575731	Loss	0.59385	3.387584	p36.33-p36.33	1.562278
OVA_047_C2	Primary	chr18	16	3	18511438	18534806	Amp	0.575254	2.829045	q11.1-q11.1	0.023368
OVA_048_C2	Primary	chr15	20	4	20170011	20613868	Gain	0.736255	1.845483	q11.1-q11.1	0.443857
OVA_047_C2	Primary	chr18	220	13	18550353	22902087	Del	0.575254	2.829045	q11.1-q11.2	4.351734
OVA_047_M1	Metastasis	chr15	47	4	20169854	21937592	Del	0.624347	2.86771	q11.1-q11.2	1.767738
OVA_047_M3	Metastasis	chr15	44	3	20169854	21902968	Loss	0.605088	2.815208	q11.1-q11.2	1.733114
OVA_047_C2	Primary	chr15	282	23	20170011	25494501	Loss	0.575254	2.829045	q11.1-q11.2	5.32449
OVA_047_M2	Metastasis	chr15	47	4	20170011	21937592	Loss	0.599725	2.678568	q11.1-q11.2	1.767581
OVA_047_C2	Primary	chr2	378	36	95480969	98797446	Loss	0.575254	2.829045	q11.1-q11.2	3.316477
OVA_378_M2	Metastasis	chr22	732	102	16123252	21797171	Del	0.840932	2.531102	q11.1-q11.21	5.673919
OVA_378_M3	Metastasis	chr22	717	102	16123252	21435186	Del	0.692693	2.569487	q11.1-q11.21	5.311934

Sample	Tissue type	chrom	num.mark	nhet	start	end	Classificati	Purity	Ploidy	Cyto	Size_Mb
OVA_378_M1	Metastasis	chr22	742	103	16123428	21823523	Del	0.753235	2.555202	q11.1-q11.21	5.700095
OVA_003_C2	Primary	chr22	668	64	16123252	22221680	Del	0.709607	3.665802	q11.1-q11.22	6.098428
OVA_003_C3	Primary	chr22	668	64	16123252	22221680	Gain	0.820093	1.828085	q11.1-q11.22	6.098428
OVA_013_C1	Primary	chr22	968	101	16123252	26854490	Loss	0.701164	3.125184	q11.1-q12.1	10.73124
OVA_013_C2	Primary	chr22	968	101	16123252	26854490	Loss	0.755564	3.14838	q11.1-q12.1	10.73124
OVA_013_M1	Metastasis	chr22	950	99	16123252	26688831	Loss	0.621947	2.883383	q11.1-q12.1	10.56558
OVA_048_M3	Metastasis	chr22	887	97	16123252	27113823	Del	0.59385	3.387584	q11.1-q12.1	10.99057
OVA_047_C1	Primary	chr5	585	58	49699034	71015314	Del	0.576016	2.862818	q11.1-q13.2	21.31628
OVA_047_CO	Contralateral ovary	chr5	553	53	49706799	71488999	Del	0.55613	2.930893	q11.1-q13.2	21.7822
OVA_047_C2	Primary	chr5	578	58	49963235	70952573	Del	0.575254	2.829045	q11.1-q13.2	20.98934
OVA_048_C3	Primary	chr15	450	52	20170150	33446610	Gain	0.715645	1.786574	q11.1-q13.3	13.27646
OVA_048_C2	Primary	chr15	431	49	20643885	33446947	Gain	0.736255	1.845483	q11.1-q13.3	12.80306
OVA_365_C2	Primary	chr22	3459	300	16123252	48025183	Loss	0.629033	1.960121	q11.1-q13.31	31.90193
OVA_013_C3	Primary	chr22	3076	350	16123252	51237364	Loss	0.75175	3.001491	q11.1-q13.33	35.11411
OVA_047_C1	Primary	chr22	3584	358	16123252	51237410	Del	0.576016	2.862818	q11.1-q13.33	35.11416
OVA_047_C2	Primary	chr22	3581	358	16123252	51237410	Del	0.575254	2.829045	q11.1-q13.33	35.11416
OVA_047_CO	Contralateral ovary	chr22	3578	356	16123252	51237410	Del	0.55613	2.930893	q11.1-q13.33	35.11416
OVA_047_M1	Metastasis	chr22	3576	360	16123252	51237486	Del	0.624347	2.86771	q11.1-q13.33	35.11423
OVA_047_M2	Metastasis	chr22	3584	360	16123252	51237486	Del	0.599725	2.678568	q11.1-q13.33	35.11423
OVA_047_M3	Metastasis	chr22	3573	356	16123252	51237410	Del	0.605088	2.815208	q11.1-q13.33	35.11416
OVA_048_M1	Metastasis	chr22	2841	287	16123252	51237364	Del	0.625824	3.962486	q11.1-q13.33	35.11411
OVA_048_M2	Metastasis	chr22	2842	288	16123252	51237364	Del	0.405007	3.72502	q11.1-q13.33	35.11411
OVA_365_M1	Metastasis	chr22	3650	324	16123252	51237364	Loss	0.854003	1.907418	q11.1-q13.33	35.11411
OVA_365_M3	Metastasis	chr22	3811	355	16123252	51237486	Loss	0.827915	1.890476	q11.1-q13.33	35.11423
OVA_378_C2	Primary	chr22	4102	476	16123252	51237410	Del	0.607575	2.601752	q11.1-q13.33	35.11416
OVA_378_C3	Primary	chr22	4137	490	16123252	51237410	Del	0.517221	2.617793	q11.1-q13.33	35.11416
OVA_378_C1	Primary	chr22	4042	465	16123428	51237364	Del	0.616455	2.593914	q11.1-q13.33	35.11394
OVA_003_C2	Primary	chr15	824	76	20170011	38258835	Del	0.709607	3.665802	q11.1-q14	18.08882
OVA_013_M1	Metastasis	chr15	726	48	20170011	38258772	Loss	0.621947	2.883383	q11.1-q14	18.08876
OVA_048_M1	Metastasis	chr15	496	54	20170011	34355903	Del	0.625824	3.962486	q11.1-q14	14.18589
OVA_013_C1	Primary	chr15	734	49	20170150	38643232	Loss	0.701164	3.125184	q11.1-q14	18.47308
OVA_013_C2	Primary	chr15	727	49	20170150	38258772	Del	0.755564	3.14838	q11.1-q14	18.08862
OVA_013_C3	Primary	chr15	727	49	20170150	38258772	Loss	0.75175	3.001491	q11.1-q14	18.08862
OVA_048_M3	Metastasis	chr15	494	54	20170150	34355263	Del	0.59385	3.387584	q11.1-q14	14.18511
OVA_048_M2	Metastasis	chr15	1667	162	20170150	51829812	Del	0.405007	3.72502	q11.1-q21.2	31.65966
OVA_047_M1	Metastasis	chr18	1189	76	18550353	58310809	Del	0.624347	2.86771	q11.1-q21.32	39.76046
OVA_047_M2	Metastasis	chr18	1190	76	18550477	58310776	Del	0.599725	2.678568	q11.1-q21.32	39.7603
OVA_047_M3	Metastasis	chr18	1187	76	18550477	59166523	Del	0.605088	2.815208	q11.1-q21.33	40.61605
OVA_378_C1	Primary	chr15	5789	529	20169854	1.03E+08	Loss	0.616455	2.593914	q11.1-q26.3	82.3464
OVA_378_C2	Primary	chr15	5809	534	20169854	1.03E+08	Loss	0.607575	2.601752	q11.1-q26.3	82.3464
OVA_378_M3	Metastasis	chr15	5789	534	20169854	1.03E+08	Loss	0.692693	2.569487	q11.1-q26.3	82.3464
OVA_378_C3	Primary	chr15	5807	534	20170011	1.03E+08	Loss	0.517221	2.617793	q11.1-q26.3	82.34625
OVA_378_M1	Metastasis	chr15	5812	535	20170011	1.03E+08	Loss	0.753235	2.555202	q11.1-q26.3	82.34625
OVA_378_M2	Metastasis	chr15	5809	533	20170011	1.03E+08	Loss	0.840932	2.531102	q11.1-q26.3	82.34625
OVA_378_C1	Primary	chr3	4536	408	93595967	1.98E+08	Gain	0.616455	2.593914	q11.1-q29	104.2516
OVA_378_C2	Primary	chr3	4536	408	93595967	1.98E+08	Gain	0.607575	2.601752	q11.1-q29	104.2516
OVA_378_C3	Primary	chr3	4536	408	93595967	1.98E+08	Gain	0.517221	2.617793	q11.1-q29	104.2516
OVA_378_M1	Metastasis	chr3	4536	408	93595967	1.98E+08	Gain	0.753235	2.555202	q11.1-q29	104.2516
OVA_378_M2	Metastasis	chr3	4536	408	93595967	1.98E+08	Amp	0.840932	2.531102	q11.1-q29	104.2516
OVA_378_M3	Metastasis	chr3	4536	408	93595967	1.98E+08	Gain	0.692693	2.569487	q11.1-q29	104.2516
OVA_047_M1	Metastasis	chr5	2888	247	49928997	1.4E+08	Del	0.624347	2.86771	q11.1-q31.3	90.15102
OVA_047_M2	Metastasis	chr5	2885	246	49963235	1.4E+08	Del	0.599725	2.678568	q11.1-q31.3	90.11669
OVA_378_C1	Primary	chr5	5663	510	49438314	1.81E+08	Loss	0.616455	2.593914	q11.1-q35.3	131.2489
OVA_378_C3	Primary	chr5	5684	511	49438314	1.81E+08	Loss	0.517221	2.617793	q11.1-q35.3	131.2489
OVA_047_M3	Metastasis	chr5	5352	525	49928993	1.81E+08	Del	0.605088	2.815208	q11.1-q35.3	130.7582
OVA_047_M1	Metastasis	chr10	42	2	43604557	44345134	Loss	0.624347	2.86771	q11.21-q11.21	0.740577
OVA_047_C2	Primary	chr10	29	1	43657728	44345134	Loss	0.575254	2.829045	q11.21-q11.21	0.687406
OVA_047_M3	Metastasis	chr10	241	33	44788714	50373858	Del	0.605088	2.815208	q11.21-q11.23	5.585144
OVA_047_M1	Metastasis	chr10	250	33	44788826	50532542	Del	0.624347	2.86771	q11.21-q11.23	5.743716
OVA_047_M2	Metastasis	chr10	257	35	44788826	50533755	Del	0.599725	2.678568	q11.21-q11.23	5.744929
OVA_048_M1	Metastasis	chr20	973	81	31805366	47242504	Del	0.625824	3.962486	q11.21-q13.13	15.43714
OVA_048_M3	Metastasis	chr20	974	81	31805408	47242539	Del	0.59385	3.387584	q11.21-q13.13	15.43713
OVA_048_C2	Primary	chr20	967	81	31946946	47242539	Loss	0.736255	1.845483	q11.21-q13.13	15.29559
OVA_048_M2	Metastasis	chr20	967	81	31946946	47242539	Del	0.405007	3.72502	q11.21-q13.13	15.29559
OVA_378_M3	Metastasis	chr22	3291	369	21480456	51237486	Del	0.692693	2.569487	q11.21-q13.33	29.75703
OVA_378_M2	Metastasis	chr22	3310	370	21799325	51237364	Del	0.840932	2.531102	q11.21-q13.33	29.43804

Sample	Tissue type	chrom	num.mark	nhet	start	end	Classificati	Purity	Ploidy	Cyto	Size_Mb
OVA_378_M1	Metastasis	chr22	3404	387	21829039	51237486	Del	0.753235	2.555202	q11.21-q13.33	29.40845
OVA_013_C3	Primary	chr10	1561	140	43088670	82300706	Del	0.75175	3.001491	q11.21-q23.1	39.21204
OVA_013_C1	Primary	chr10	1576	141	43089331	85910518	Del	0.701164	3.125184	q11.21-q23.1	42.82119
OVA_013_C2	Primary	chr10	1565	141	43127339	84733493	Del	0.755564	3.14838	q11.21-q23.1	41.60615
OVA_048_M2	Metastasis	chr8	2364	196	49985395	1.46E+08	Del	0.405007	3.72502	q11.21-q24.3	96.29408
OVA_047_C1	Primary	chr10	3159	262	44788717	1.12E+08	Del	0.576016	2.862818	q11.21-q25.1	66.8363
OVA_047_CO	Contralateral ovary	chr10	3137	259	44788826	1.12E+08	Del	0.55613	2.930893	q11.21-q25.1	66.83619
OVA_047_C2	Primary	chr10	3160	262	44788837	1.09E+08	Del	0.575254	2.829045	q11.21-q25.1	64.13487
OVA_003_C2	Primary	chr22	2999	293	22277571	51237410	Del	0.709607	3.665802	q11.22-q13.33	28.95984
OVA_003_C3	Primary	chr22	3013	297	22277675	51237486	Loss	0.820093	1.828085	q11.22-q13.33	28.95981
OVA_378_M1	Metastasis	chr8	154	3	52762770	59416418	Amp	0.753235	2.555202	q11.23-q12.1	6.653648
OVA_378_M2	Metastasis	chr8	154	3	52762770	59416418	Amp	0.840932	2.531102	q11.23-q12.1	6.653648
OVA_378_M3	Metastasis	chr8	154	3	52762778	59416418	Amp	0.692693	2.569487	q11.23-q12.1	6.65364
OVA_378_C3	Primary	chr8	80	1	55370971	57358556	Amp	0.517221	2.617793	q11.23-q12.1	1.987585
OVA_378_C1	Primary	chr8	84	1	55371777	59059228	Amp	0.616455	2.593914	q11.23-q12.1	3.687451
OVA_378_C2	Primary	chr8	78	1	55372516	57358535	Amp	0.607575	2.601752	q11.23-q12.1	1.986019
OVA_003_C2	Primary	chr7	1054	83	73022089	96765730	Del	0.709607	3.665802	q11.23-q21.3	23.74364
OVA_047_C2	Primary	chr7	697	51	76632709	96747237	Del	0.575254	2.829045	q11.23-q21.3	20.11453
OVA_047_M1	Metastasis	chr7	1920	133	73449755	1.06E+08	Del	0.624347	2.86771	q11.23-q22.3	32.15406
OVA_047_M3	Metastasis	chr7	1813	131	75044238	1.06E+08	Del	0.605088	2.815208	q11.23-q22.3	30.61151
OVA_047_M2	Metastasis	chr7	1821	132	75070377	1.07E+08	Del	0.599725	2.678568	q11.23-q22.3	31.43763
OVA_013_M1	Metastasis	chr10	1350	122	49918021	85961556	Loss	0.621947	2.883383	q11.23-q23.1	36.04354
OVA_047_M3	Metastasis	chr10	2898	226	50374819	1.12E+08	Del	0.605088	2.815208	q11.23-q25.1	61.2502
OVA_047_M1	Metastasis	chr10	2895	227	50532822	1.09E+08	Del	0.624347	2.86771	q11.23-q25.1	58.39088
OVA_047_M2	Metastasis	chr10	2895	227	50534149	1.09E+08	Del	0.599725	2.678568	q11.23-q25.1	58.38956
OVA_013_C2	Primary	chr7	2785	163	72419438	1.31E+08	Loss	0.755564	3.14838	q11.23-q32.3	58.59335
OVA_013_C1	Primary	chr7	2798	165	72420066	1.31E+08	Loss	0.701164	3.125184	q11.23-q32.3	58.77367
OVA_013_C1	Primary	chr14	669	72	19114472	24029038	Del	0.701164	3.125184	q11.2-q11.2	4.914566
OVA_013_C2	Primary	chr14	665	71	19114472	24004070	Del	0.755564	3.14838	q11.2-q11.2	4.889598
OVA_013_C3	Primary	chr14	665	71	19114472	24004124	Loss	0.75175	3.001491	q11.2-q11.2	4.889652
OVA_013_M1	Metastasis	chr14	665	71	19114472	24004070	Loss	0.621947	2.883383	q11.2-q11.2	4.889598
OVA_048_M2	Metastasis	chr14	63	11	19114472	20757838	Loss	0.405007	3.72502	q11.2-q11.2	1.643366
OVA_378_C1	Primary	chr14	474	75	19114472	22749282	Loss	0.616455	2.593914	q11.2-q11.2	3.63481
OVA_378_C2	Primary	chr14	474	72	19114472	22739862	Loss	0.607575	2.601752	q11.2-q11.2	3.62539
OVA_378_C3	Primary	chr14	475	73	19114472	22740245	Loss	0.517221	2.617793	q11.2-q11.2	3.625773
OVA_378_M1	Metastasis	chr14	473	71	19114472	22733655	Loss	0.753235	2.555202	q11.2-q11.2	3.619183
OVA_378_M2	Metastasis	chr14	475	73	19114472	22740270	Loss	0.840932	2.531102	q11.2-q11.2	3.625798
OVA_378_M3	Metastasis	chr14	468	71	19114472	22689901	Loss	0.692693	2.569487	q11.2-q11.2	3.575429
OVA_047_C1	Primary	chr17	365	27	27037826	30689814	Del	0.576016	2.862818	q11.2-q11.2	3.651988
OVA_047_C2	Primary	chr17	332	25	27086200	30678740	Del	0.575254	2.829045	q11.2-q11.2	3.59254
OVA_047_CO	Contralateral ovary	chr17	315	23	27088955	30611885	Del	0.55613	2.930893	q11.2-q11.2	3.52293
OVA_013_C1	Primary	chr5	19	4	52370944	53183268	Del	0.701164	3.125184	q11.2-q11.2	0.812324
OVA_013_C3	Primary	chr5	19	4	52370944	53183268	Del	0.75175	3.001491	q11.2-q11.2	0.812324
OVA_003_C2	Primary	chr14	1167	123	19114472	31404314	Del	0.709607	3.665802	q11.2-q12	12.28984
OVA_047_M1	Metastasis	chr14	1192	150	19114472	31897386	Loss	0.624347	2.86771	q11.2-q12	12.78291
OVA_047_M2	Metastasis	chr14	1193	150	19114472	31897390	Loss	0.599725	2.678568	q11.2-q12	12.78292
OVA_047_M3	Metastasis	chr14	1193	150	19114472	31897390	Loss	0.605088	2.815208	q11.2-q12	12.78292
OVA_047_CO	Contralateral ovary	chr18	189	21	22902087	29848436	Loss	0.55613	2.930893	q11.2-q12.1	6.946349
OVA_047_C2	Primary	chr18	199	22	23598286	30321928	Loss	0.575254	2.829045	q11.2-q12.1	6.723642
OVA_047_C1	Primary	chr18	191	21	23612269	29867972	Loss	0.576016	2.862818	q11.2-q12.1	6.255703
OVA_047_C1	Primary	chr16	241	7	46387351	51172677	Del	0.576016	2.862818	q11.2-q12.1	4.785326
OVA_047_CO	Contralateral ovary	chr16	239	7	46404513	51172677	Del	0.55613	2.930893	q11.2-q12.1	4.768164
OVA_047_C2	Primary	chr16	232	6	46509850	51172677	Del	0.575254	2.829045	q11.2-q12.1	4.662827
OVA_003_C3	Primary	chr3	366	24	96713004	1.08E+08	Amp	0.820093	1.828085	q11.2-q13.13	11.22544
OVA_003_C2	Primary	chr3	365	24	96963004	1.08E+08	Loss	0.709607	3.665802	q11.2-q13.13	10.97544
OVA_003_C1	Primary	chr3	365	24	96963103	1.08E+08	Amp	0.852911	1.812048	q11.2-q13.13	10.97534
OVA_003_M1	Metastasis	chr3	324	20	97618089	1.08E+08	Gain	0.552447	1.852543	q11.2-q13.13	10.31932
OVA_003_M2	Metastasis	chr3	326	20	97652036	1.08E+08	Gain	0.439877	1.867794	q11.2-q13.13	10.42918
OVA_003_M3	Metastasis	chr3	323	20	97652036	1.08E+08	Gain	0.574876	1.865189	q11.2-q13.13	10.28913
OVA_003_C1	Primary	chr5	686	63	51376531	72354329	Loss	0.852911	1.812048	q11.2-q13.2	20.9778
OVA_003_C3	Primary	chr5	690	65	51376531	72354329	Loss	0.820093	1.828085	q11.2-q13.2	20.9778
OVA_013_C2	Primary	chr5	403	31	53183268	68868345	Del	0.755564	3.14838	q11.2-q13.2	15.68508
OVA_013_C1	Primary	chr5	432	34	53467817	68868345	Del	0.701164	3.125184	q11.2-q13.2	15.40053
OVA_013_C3	Primary	chr5	409	31	53467817	69325589	Del	0.75175	3.001491	q11.2-q13.2	15.85777
OVA_013_M1	Metastasis	chr5	436	36	53467817	68868345	Del	0.621947	2.883383	q11.2-q13.2	15.40053
OVA_047_CO	Contralateral ovary	chr14	1455	164	19114472	39871017	Loss	0.55613	2.930893	q11.2-q21.1	20.75655

Sample	Tissue type	chrom	num.mark	nhet	start	end	Classificati	Purity	Ploidy	Cyto	Size_Mb
OVA_047_C1	Primary	chr14	1473	166	19114472	45369714	Loss	0.576016	2.862818	q11.2-q21.2	26.25524
OVA_378_M1	Metastasis	chr17	1734	151	29315049	41607364	Del	0.753235	2.555202	q11.2-q21.31	12.29232
OVA_378_M2	Metastasis	chr17	1671	144	29315049	41607364	Del	0.840932	2.531102	q11.2-q21.31	12.29232
OVA_378_M3	Metastasis	chr17	1651	139	29324169	41607364	Del	0.692693	2.569487	q11.2-q21.31	12.2832
OVA_047_CO	Contralateral ovary	chr17	2560	206	30625205	53237230	Del	0.55613	2.930893	q11.2-q22	22.61203
OVA_047_C2	Primary	chr17	2560	207	30687864	53498990	Del	0.575254	2.829045	q11.2-q22	22.81113
OVA_047_C1	Primary	chr17	2556	205	30690095	53488764	Del	0.576016	2.862818	q11.2-q22	22.79867
OVA_013_C3	Primary	chr21	342	41	15309725	33103936	Del	0.75175	3.001491	q11.2-q22.11	17.79421
OVA_047_C2	Primary	chr21	1225	148	14916669	45679488	Loss	0.575254	2.829045	q11.2-q22.3	30.76282
OVA_048_M3	Metastasis	chr14	1610	137	19114472	57268665	Del	0.59385	3.387584	q11.2-q22.3	38.15419
OVA_047_C2	Primary	chr14	1128	80	24435565	55618524	Loss	0.575254	2.829045	q11.2-q22.3	31.18296
OVA_047_C1	Primary	chr15	2648	199	23687708	63908587	Del	0.576016	2.862818	q11.2-q22.31	40.22088
OVA_047_CO	Contralateral ovary	chr15	2638	198	23688957	63930993	Del	0.55613	2.930893	q11.2-q22.31	40.24204
OVA_047_C2	Primary	chr15	2527	192	25496028	63918279	Del	0.575254	2.829045	q11.2-q22.31	38.42225
OVA_365_C2	Primary	chr2	1738	141	98305342	1.49E+08	Loss	0.629033	1.960121	q11.2-q23.1	50.94187
OVA_365_M1	Metastasis	chr2	1691	130	98305342	1.49E+08	Loss	0.854003	1.907418	q11.2-q23.1	50.94249
OVA_365_M3	Metastasis	chr2	1718	140	98305342	1.49E+08	Loss	0.827915	1.890476	q11.2-q23.1	50.42572
OVA_047_M3	Metastasis	chr15	3687	262	21936772	75676597	Del	0.605088	2.815208	q11.2-q24.2	53.73983
OVA_047_M1	Metastasis	chr15	3712	261	21937970	75798270	Del	0.624347	2.86771	q11.2-q24.2	53.8603
OVA_047_M2	Metastasis	chr15	3705	263	21937970	75660765	Del	0.599725	2.678568	q11.2-q24.2	53.7228
OVA_048_M2	Metastasis	chr16	2086	193	46394927	88504239	Del	0.405007	3.72502	q11.2-q24.2	42.10931
OVA_048_M3	Metastasis	chr16	2088	193	46394927	88504868	Del	0.59385	3.387584	q11.2-q24.2	42.10994
OVA_048_M1	Metastasis	chr14	3769	326	19114472	1.04E+08	Del	0.625824	3.962486	q11.2-q32.33	85.27278
OVA_048_M2	Metastasis	chr14	3708	316	20760285	1.04E+08	Del	0.405007	3.72502	q11.2-q32.33	83.64696
OVA_013_C2	Primary	chr14	3726	322	24026174	1.05E+08	Del	0.755564	3.14838	q11.2-q32.33	81.12173
OVA_013_C3	Primary	chr14	3692	317	24026174	1.04E+08	Del	0.75175	3.001491	q11.2-q32.33	80.36101
OVA_013_M1	Metastasis	chr14	3877	334	24026174	1.06E+08	Del	0.621947	2.883383	q11.2-q32.33	81.78872
OVA_013_C1	Primary	chr14	3685	315	24031063	1.04E+08	Del	0.701164	3.125184	q11.2-q32.33	80.35612
OVA_013_C1	Primary	chr13	19	2	19255728	20207945	Del	0.701164	3.125184	q11-q12.11	0.952217
OVA_013_M1	Metastasis	chr13	19	2	19255728	20208136	Del	0.621947	2.883383	q11-q12.11	0.952408
OVA_013_C3	Primary	chr13	375	41	19255728	28367941	Loss	0.75175	3.001491	q11-q12.2	9.112213
OVA_003_C2	Primary	chr13	1136	96	19239460	45914957	Del	0.709607	3.665802	q11-q14.13	26.6755
OVA_047_M1	Metastasis	chr13	1485	130	19255728	60849890	Loss	0.624347	2.86771	q11-q21.2	41.59416
OVA_047_M3	Metastasis	chr13	1490	130	19255728	61083887	Loss	0.605088	2.815208	q11-q21.2	41.82816
OVA_047_M2	Metastasis	chr13	1482	129	19255746	60590299	Loss	0.599725	2.678568	q11-q21.2	41.33455
OVA_013_C2	Primary	chr13	1405	86	19255746	67205386	Del	0.755564	3.14838	q11-q21.32	47.94964
OVA_047_C1	Primary	chr13	1773	149	19255709	92380829	Loss	0.576016	2.862818	q11-q31.3	73.12512
OVA_047_CO	Contralateral ovary	chr13	1765	148	19255746	92380829	Del	0.55613	2.930893	q11-q31.3	73.12508
OVA_378_C1	Primary	chr13	2728	248	19239539	1.15E+08	Loss	0.616455	2.593914	q11-q34	95.85219
OVA_378_C2	Primary	chr13	2739	249	19239539	1.15E+08	Loss	0.607575	2.601752	q11-q34	95.85219
OVA_378_C3	Primary	chr13	2735	248	19239539	1.15E+08	Loss	0.517221	2.617793	q11-q34	95.85219
OVA_378_M1	Metastasis	chr13	2739	249	19239539	1.15E+08	Loss	0.753235	2.555202	q11-q34	95.85219
OVA_378_M2	Metastasis	chr13	2737	248	19239539	1.15E+08	Loss	0.840932	2.531102	q11-q34	95.85219
OVA_378_M3	Metastasis	chr13	2727	248	19239539	1.15E+08	Loss	0.692693	2.569487	q11-q34	95.85219
OVA_047_C2	Primary	chr13	26	1	21007864	21417993	Del	0.575254	2.829045	q12.11-q12.11	0.410129
OVA_013_C1	Primary	chr13	356	39	20208232	28367830	Del	0.701164	3.125184	q12.11-q12.2	8.159598
OVA_013_M1	Metastasis	chr13	356	39	20208232	28367830	Loss	0.621947	2.883383	q12.11-q12.2	8.159598
OVA_048_M1	Metastasis	chr13	1091	94	19601049	61058880	Del	0.625824	3.962486	q12.11-q21.2	41.45783
OVA_048_M2	Metastasis	chr13	1093	92	19601049	66878857	Del	0.405007	3.72502	q12.11-q21.32	47.27781
OVA_048_M3	Metastasis	chr13	1096	95	19601049	67801721	Del	0.59385	3.387584	q12.11-q21.32	48.20067
OVA_048_M1	Metastasis	chr18	113	7	28662899	31684050	Del	0.625824	3.962486	q12.1-q12.1	3.021151
OVA_047_C1	Primary	chr18	171	3	29890293	35145388	Del	0.576016	2.862818	q12.1-q12.2	5.255095
OVA_013_C2	Primary	chr18	155	8	30254600	35145388	Loss	0.755564	3.14838	q12.1-q12.2	4.890788
OVA_013_C3	Primary	chr18	156	8	30254600	35145388	Loss	0.75175	3.001491	q12.1-q12.2	4.890788
OVA_047_C2	Primary	chr18	165	2	30349640	35145388	Del	0.575254	2.829045	q12.1-q12.2	4.795748
OVA_048_M1	Metastasis	chr18	107	8	31801905	34475737	Del	0.625824	3.962486	q12.1-q12.2	2.673832
OVA_378_M1	Metastasis	chr8	72	2	59474247	62003453	Gain	0.753235	2.555202	q12.1-q12.2	2.529206
OVA_378_M2	Metastasis	chr8	72	2	59474247	62003453	Amp	0.840932	2.531102	q12.1-q12.2	2.529206
OVA_378_M3	Metastasis	chr8	72	2	59474247	62003453	Gain	0.692693	2.569487	q12.1-q12.2	2.529206
OVA_047_CO	Contralateral ovary	chr18	175	3	29850296	39535147	Del	0.55613	2.930893	q12.1-q12.3	9.684851
OVA_013_C1	Primary	chr18	157	8	30254600	37256682	Loss	0.701164	3.125184	q12.1-q12.3	7.002082
OVA_013_M1	Metastasis	chr16	317	26	50813997	57113168	Del	0.621947	2.883383	q12.1-q13	6.299171
OVA_378_C3	Primary	chr8	349	10	57876493	70771505	Amp	0.517221	2.617793	q12.1-q13.3	12.89501
OVA_013_M1	Metastasis	chr22	1745	197	26694919	46733831	Loss	0.621947	2.883383	q12.1-q13.31	20.03891
OVA_013_C1	Primary	chr22	1731	195	26860269	46752911	Del	0.701164	3.125184	q12.1-q13.31	19.89264
OVA_013_C2	Primary	chr22	1800	209	26860269	47882492	Del	0.755564	3.14838	q12.1-q13.31	21.02222

Sample	Tissue type	chrom	num.mark	nhet	start	end	Classificati	Purity	Ploidy	Cyto	Size_Mb
OVA_048_M3	Metastasis	chr22	1583	164	27456046	46681173	Del	0.59385	3.387584	q12.1-q13.31	19.22513
OVA_047_M2	Metastasis	chr16	631	78	51173013	66547767	Del	0.599725	2.678568	q12.1-q21	15.37475
OVA_047_M1	Metastasis	chr16	633	78	51173858	66570946	Del	0.624347	2.86771	q12.1-q21	15.39709
OVA_047_M3	Metastasis	chr16	640	83	51175324	66638804	Del	0.605088	2.815208	q12.1-q21	15.46348
OVA_003_M3	Metastasis	chr16	609	50	52472823	58752269	Gain	0.574876	1.865189	q12.1-q21	6.279446
OVA_378_C1	Primary	chr8	467	19	59345945	75262677	Gain	0.616455	2.593914	q12.1-q21.11	15.91673
OVA_378_C2	Primary	chr8	831	43	57876468	93156546	Gain	0.607575	2.601752	q12.1-q21.3	35.28008
OVA_047_C1	Primary	chr16	1343	144	51173013	70834675	Del	0.576016	2.862818	q12.1-q22.2	19.66166
OVA_047_C2	Primary	chr16	1355	150	51173013	70867990	Del	0.575254	2.829045	q12.1-q22.2	19.69498
OVA_047_CO	Contralateral ovary	chr16	1329	143	51173013	70867990	Del	0.55613	2.930893	q12.1-q22.2	19.69498
OVA_047_CO	Contralateral ovary	chr8	1054	76	59720368	1.01E+08	Loss	0.55613	2.930893	q12.1-q22.2	41.3002
OVA_047_C2	Primary	chr8	1411	119	59571856	1.11E+08	Loss	0.575254	2.829045	q12.1-q23.2	51.13156
OVA_048_C2	Primary	chr8	2277	191	56711713	1.46E+08	Amp	0.736255	1.845483	q12.1-q24.3	89.56764
OVA_048_M1	Metastasis	chr8	2273	190	56854583	1.46E+08	Del	0.625824	3.962486	q12.1-q24.3	89.4249
OVA_048_M3	Metastasis	chr8	2271	190	56863151	1.46E+08	Loss	0.59385	3.387584	q12.1-q24.3	89.41625
OVA_048_C1	Primary	chr8	2270	190	56866452	1.46E+08	Gain	0.682521	1.843999	q12.1-q24.3	89.41303
OVA_048_C3	Primary	chr8	2264	188	57026379	1.46E+08	Amp	0.715645	1.786574	q12.1-q24.3	89.25302
OVA_365_M1	Metastasis	chr5	1831	124	59443507	1.31E+08	Loss	0.854003	1.907418	q12.1-q31.1	71.83679
OVA_365_M3	Metastasis	chr5	1856	130	59443575	1.31E+08	Loss	0.827915	1.890476	q12.1-q31.1	71.83751
OVA_365_C2	Primary	chr5	1860	130	59443691	1.31E+08	Loss	0.629033	1.960121	q12.1-q31.1	71.83739
OVA_013_C1	Primary	chr13	109	7	28539185	31903712	Del	0.701164	3.125184	q12.2-q12.3	3.364527
OVA_013_C3	Primary	chr13	109	7	28539185	31903712	Del	0.75175	3.001491	q12.2-q12.3	3.364527
OVA_048_C3	Primary	chr18	32	0	34289142	39613942	Gain	0.715645	1.786574	q12.2-q12.3	5.3248
OVA_378_M2	Metastasis	chr11	326	23	60269521	62140075	Gain	0.840932	2.531102	q12.2-q12.3	1.870554
OVA_013_C1	Primary	chr11	182	30	60718792	62201458	Del	0.701164	3.125184	q12.2-q12.3	1.482666
OVA_013_M1	Metastasis	chr11	173	27	60870122	62201458	Del	0.621947	2.883383	q12.2-q12.3	1.331336
OVA_013_C2	Primary	chr11	465	49	60739313	63449138	Del	0.755564	3.14838	q12.2-q13.1	2.709825
OVA_013_C3	Primary	chr11	467	49	60739313	63449125	Del	0.75175	3.001491	q12.2-q13.1	2.709812
OVA_013_M1	Metastasis	chr13	276	20	28539185	36699762	Del	0.621947	2.883383	q12.2-q13.3	8.160577
OVA_003_M1	Metastasis	chr16	505	33	55571563	60393281	Gain	0.552447	1.852543	q12.2-q21	4.821718
OVA_003_C2	Primary	chr16	491	31	55731946	60393281	Del	0.709607	3.665802	q12.2-q21	4.661335
OVA_003_M2	Metastasis	chr16	398	18	56685916	64294619	Gain	0.439877	1.867794	q12.2-q21	7.608703
OVA_048_M1	Metastasis	chr18	139	14	34539379	44641541	Loss	0.625824	3.962486	q12.2-q21.1	10.10216
OVA_047_C1	Primary	chr8	721	47	61757724	92136780	Loss	0.576016	2.862818	q12.2-q21.3	30.37906
OVA_003_C2	Primary	chr18	1229	100	33077971	77933813	Del	0.709607	3.665802	q12.2-q23	44.85584
OVA_003_C1	Primary	chr18	1237	100	33171812	77933813	Loss	0.852911	1.812048	q12.2-q23	44.762
OVA_378_M3	Metastasis	chr11	267	12	62066438	62902172	Amp	0.692693	2.569487	q12.3-q12.3	0.835734
OVA_378_M1	Metastasis	chr11	263	12	62156608	62902172	Amp	0.753235	2.555202	q12.3-q12.3	0.745564
OVA_378_M2	Metastasis	chr11	263	12	62156608	62902172	Amp	0.840932	2.531102	q12.3-q12.3	0.745564
OVA_013_M1	Metastasis	chr11	287	21	62283782	63481378	Del	0.621947	2.883383	q12.3-q13.1	1.197596
OVA_013_C1	Primary	chr11	286	21	62283799	63449138	Del	0.701164	3.125184	q12.3-q13.1	1.165339
OVA_378_M1	Metastasis	chr11	1595	114	62910849	70281689	Loss	0.753235	2.555202	q12.3-q13.3	7.37084
OVA_378_M2	Metastasis	chr11	1595	114	62910849	70281845	Loss	0.840932	2.531102	q12.3-q13.3	7.370996
OVA_378_M3	Metastasis	chr11	1589	112	62910849	70281083	Loss	0.692693	2.569487	q12.3-q13.3	7.370234
OVA_048_C1	Primary	chr18	123	14	39613942	44639406	Amp	0.682521	1.843999	q12.3-q21.1	5.025464
OVA_048_C2	Primary	chr18	122	14	39617703	44639406	Amp	0.736255	1.845483	q12.3-q21.1	5.021703
OVA_048_C3	Primary	chr18	122	14	39617703	44639406	Amp	0.715645	1.786574	q12.3-q21.1	5.021703
OVA_013_C2	Primary	chr18	1034	94	37256682	77933813	Del	0.755564	3.14838	q12.3-q23	40.67713
OVA_013_C3	Primary	chr18	1040	95	37256682	77933813	Del	0.75175	3.001491	q12.3-q23	40.67713
OVA_013_C1	Primary	chr18	1042	95	39535175	77933813	Del	0.701164	3.125184	q12.3-q23	38.39864
OVA_365_C2	Primary	chr11	4246	330	63230909	1.24E+08	Gain	0.629033	1.960121	q12.3-q24.1	60.2935
OVA_047_M2	Metastasis	chr2	2269	217	109527343	1.73E+08	Loss	0.599725	2.678568	q12.3-q31.1	63.9333
OVA_047_M1	Metastasis	chr2	2278	218	109529258	1.74E+08	Loss	0.624347	2.86771	q12.3-q31.1	64.25768
OVA_047_M3	Metastasis	chr2	2299	220	109539743	1.74E+08	Loss	0.605088	2.815208	q12.3-q31.1	64.54616
OVA_048_M1	Metastasis	chr12	64	2	38710819	40757330	Del	0.625824	3.962486	q12-q12	2.046511
OVA_048_M2	Metastasis	chr12	198	23	38710819	43750375	Del	0.405007	3.72502	q12-q12	5.039556
OVA_048_M3	Metastasis	chr12	57	2	39096664	40757330	Del	0.59385	3.387584	q12-q12	1.660666
OVA_047_CO	Contralateral ovary	chr12	158	0	40158443	40957375	Del	0.55613	2.930893	q12-q12	0.798932
OVA_047_C2	Primary	chr6	15	0	64282579	64421268	Del	0.575254	2.829045	q12-q12	0.138689
OVA_047_CO	Contralateral ovary	chr6	16	1	64282579	64422505	Del	0.55613	2.930893	q12-q12	0.139926
OVA_048_M1	Metastasis	chr12	234	23	40799934	46761127	Del	0.625824	3.962486	q12-q13.11	5.961193
OVA_048_M3	Metastasis	chr12	234	23	40799939	46761127	Del	0.59385	3.387584	q12-q13.11	5.961188
OVA_048_M2	Metastasis	chr12	93	2	43763144	46633488	Del	0.405007	3.72502	q12-q13.11	2.870344
OVA_047_C2	Primary	chr20	537	36	39990371	46291937	Loss	0.575254	2.829045	q12-q13.12	6.301566
OVA_047_CO	Contralateral ovary	chr20	517	36	40034097	46251093	Loss	0.55613	2.930893	q12-q13.12	6.216996
OVA_047_C1	Primary	chr20	510	36	40162062	46291791	Loss	0.576016	2.862818	q12-q13.12	6.129729

Sample	Tissue type	chrom	num.mark	nhet	start	end	Classificati	Purity	Ploidy	Cyto	Size_Mb
OVA_048_C2	Primary	chr12	914	83	38710819	52470659	Loss	0.736255	1.845483	q12-q13.13	13.75984
OVA_365_M3	Metastasis	chr19	2237	265	30303562	45116947	Loss	0.827915	1.890476	q12-q13.31	14.81339
OVA_365_C2	Primary	chr19	2263	266	30303778	45116947	Loss	0.629033	1.960121	q12-q13.31	14.81317
OVA_378_M3	Metastasis	chr19	6074	681	30099577	59093484	Loss	0.692693	2.569487	q12-q13.43	28.99391
OVA_365_M1	Metastasis	chr19	5254	568	30303562	59093484	Loss	0.854003	1.907418	q12-q13.43	28.78992
OVA_047_CO	Contralateral ovary	chr6	822	45	64423229	94120688	Loss	0.55613	2.930893	q12-q16.1	29.69746
OVA_047_C2	Primary	chr4	810	94	52709426	77087389	Loss	0.575254	2.829045	q12-q21.1	24.37796
OVA_003_C2	Primary	chr9	200	16	65595106	74979326	Del	0.709607	3.665802	q12-q21.13	9.38422
OVA_013_C2	Primary	chr12	2659	204	44167854	88383120	Del	0.755564	3.14838	q12-q21.32	44.21527
OVA_013_C3	Primary	chr12	2658	201	44194352	88383120	Loss	0.75175	3.001491	q12-q21.32	44.18877
OVA_047_CO	Contralateral ovary	chr12	3185	239	40959415	95656704	Del	0.55613	2.930893	q12-q22	54.69729
OVA_003_M1	Metastasis	chr9	719	63	65595106	90744814	Gain	0.552447	1.852543	q12-q22.1	25.14971
OVA_003_M2	Metastasis	chr9	708	61	65595106	90582470	Gain	0.439877	1.867794	q12-q22.1	24.98736
OVA_003_C2	Primary	chr14	911	92	31416557	56763840	Del	0.709607	3.665802	q12-q22.3	25.34728
OVA_003_C3	Primary	chr14	931	96	31416557	57046707	Loss	0.820093	1.828085	q12-q22.3	25.63015
OVA_003_C1	Primary	chr14	920	94	31416573	57046707	Loss	0.852911	1.812048	q12-q22.3	25.63013
OVA_365_C2	Primary	chr12	3375	246	43945154	1.02E+08	Loss	0.629033	1.960121	q12-q23.2	58.11123
OVA_365_M3	Metastasis	chr12	3359	240	43945603	1.02E+08	Loss	0.827915	1.890476	q12-q23.2	58.11078
OVA_365_M1	Metastasis	chr12	3282	232	44124214	1.02E+08	Loss	0.854003	1.907418	q12-q23.2	57.92277
OVA_047_C1	Primary	chr6	1927	108	64282579	1.32E+08	Loss	0.576016	2.862818	q12-q23.2	67.25829
OVA_047_C2	Primary	chr6	1912	108	64422505	1.32E+08	Loss	0.575254	2.829045	q12-q23.2	67.11837
OVA_047_C2	Primary	chr12	4103	306	40114744	1.1E+08	Del	0.575254	2.829045	q12-q24.11	69.46249
OVA_047_M3	Metastasis	chr12	4377	328	40224052	1.12E+08	Del	0.605088	2.815208	q12-q24.12	71.98943
OVA_047_M2	Metastasis	chr12	4492	337	40114744	1.13E+08	Del	0.599725	2.678568	q12-q24.13	73.26477
OVA_047_M1	Metastasis	chr12	4310	336	40953023	1.13E+08	Del	0.624347	2.86771	q12-q24.13	71.97407
OVA_047_M1	Metastasis	chr14	1611	94	31922408	72921127	Del	0.624347	2.86771	q12-q24.2	40.99872
OVA_047_M2	Metastasis	chr14	1615	94	31922408	72818811	Del	0.599725	2.678568	q12-q24.2	40.8964
OVA_047_M3	Metastasis	chr14	1609	93	31922408	72818811	Del	0.605088	2.815208	q12-q24.2	40.8964
OVA_047_C1	Primary	chr12	5913	433	40158511	1.34E+08	Del	0.576016	2.862818	q12-q24.33	93.65237
OVA_013_C1	Primary	chr12	24	0	47471968	48131465	Del	0.701164	3.125184	q13.11-q13.11	0.659497
OVA_013_M1	Metastasis	chr19	499	62	33792175	38187583	Del	0.621947	2.883383	q13.11-q13.12	4.395408
OVA_013_C1	Primary	chr19	488	61	33968824	38187583	Del	0.701164	3.125184	q13.11-q13.12	4.218759
OVA_013_C3	Primary	chr19	501	65	33954908	38378822	Del	0.75175	3.001491	q13.11-q13.13	4.423914
OVA_013_C2	Primary	chr19	502	64	34262419	38384740	Del	0.755564	3.14838	q13.11-q13.13	4.122321
OVA_048_M2	Metastasis	chr12	627	59	46662700	52566831	Del	0.405007	3.72502	q13.11-q13.13	5.904131
OVA_048_M1	Metastasis	chr12	617	58	46764254	52565257	Del	0.625824	3.962486	q13.11-q13.13	5.801003
OVA_048_M3	Metastasis	chr12	621	59	46764254	52566887	Del	0.59385	3.387584	q13.11-q13.13	5.802633
OVA_013_C1	Primary	chr12	2557	194	48132451	88928434	Del	0.701164	3.125184	q13.11-q21.32	40.79598
OVA_013_M1	Metastasis	chr12	2612	196	47471439	91365717	Loss	0.621947	2.883383	q13.11-q21.33	43.89428
OVA_047_C2	Primary	chr3	121	11	106967937	1.1E+08	Amp	0.575254	2.829045	q13.12-q13.13	3.433549
OVA_047_C1	Primary	chr3	120	10	106968034	1.09E+08	Amp	0.576016	2.862818	q13.12-q13.13	2.16084
OVA_047_CO	Contralateral ovary	chr3	120	10	106968034	1.09E+08	Amp	0.55613	2.930893	q13.12-q13.13	2.16084
OVA_365_M1	Metastasis	chr20	352	12	44757289	50406513	Loss	0.854003	1.907418	q13.12-q13.2	5.649224
OVA_365_M3	Metastasis	chr20	353	13	44757814	50329483	Loss	0.827915	1.890476	q13.12-q13.2	5.571669
OVA_365_C2	Primary	chr20	357	13	44803287	50401023	Loss	0.629033	1.960121	q13.12-q13.2	5.597736
OVA_013_C1	Primary	chr19	1474	116	38189440	46546250	Del	0.701164	3.125184	q13.12-q13.32	8.35681
OVA_013_M1	Metastasis	chr19	1474	116	38189440	46546250	Loss	0.621947	2.883383	q13.12-q13.32	8.35681
OVA_003_M2	Metastasis	chr20	1460	181	44098542	62904703	Gain	0.439877	1.867794	q13.12-q13.33	18.80616
OVA_003_C1	Primary	chr20	1458	180	44108796	62904703	Gain	0.852911	1.812048	q13.12-q13.33	18.79591
OVA_003_M3	Metastasis	chr20	1458	180	44108796	62904703	Gain	0.574876	1.865189	q13.12-q13.33	18.79591
OVA_003_C3	Primary	chr20	1457	179	44141400	62904703	Gain	0.820093	1.828085	q13.12-q13.33	18.7633
OVA_003_M1	Metastasis	chr20	1456	179	44141400	62904703	Gain	0.552447	1.852543	q13.12-q13.33	18.7633
OVA_003_C2	Primary	chr20	1455	179	44163825	62904703	Loss	0.709607	3.665802	q13.12-q13.33	18.74088
OVA_003_M3	Metastasis	chr12	129	34	52437672	52942121	Amp	0.574876	1.865189	q13.13-q13.13	0.504449
OVA_003_C3	Primary	chr12	210	43	52467449	53346495	Gain	0.820093	1.828085	q13.13-q13.13	0.879046
OVA_003_C2	Primary	chr12	207	43	52565322	53346404	Loss	0.709607	3.665802	q13.13-q13.13	0.781082
OVA_003_M1	Metastasis	chr12	205	43	52565322	53345582	Gain	0.552447	1.852543	q13.13-q13.13	0.78026
OVA_048_M1	Metastasis	chr12	110	16	52565922	53291364	Del	0.625824	3.962486	q13.13-q13.13	0.725442
OVA_003_C1	Primary	chr12	205	42	52566231	53346422	Amp	0.852911	1.812048	q13.13-q13.13	0.780191
OVA_048_M3	Metastasis	chr12	107	15	52574514	53291364	Del	0.59385	3.387584	q13.13-q13.13	0.71685
OVA_048_M2	Metastasis	chr12	108	15	52574630	53292643	Del	0.405007	3.72502	q13.13-q13.13	0.718013
OVA_365_CO	Contralateral ovary	chr12	183	50	52713036	53291411	Loss	0.451025	1.890627	q13.13-q13.13	0.578375
OVA_013_C3	Primary	chr19	1460	113	38379200	46526169	Loss	0.75175	3.001491	q13.13-q13.32	8.146969
OVA_013_C2	Primary	chr19	1441	111	38385452	46464380	Loss	0.755564	3.14838	q13.13-q13.32	8.078928
OVA_048_C3	Primary	chr20	899	97	47244164	62904703	Gain	0.715645	1.786574	q13.13-q13.33	15.66054
OVA_048_M1	Metastasis	chr20	899	97	47244164	62904845	Del	0.625824	3.962486	q13.13-q13.33	15.66068

Sample	Tissue type	chrom	num.mark	nhet	start	end	Classificati	Purity	Ploidy	Cyto	Size_Mb
OVA_048_M2	Metastasis	chr20	899	97	47244164	62904703	Del	0.405007	3.72502	q13.13-q13.33	15.66054
OVA_048_M3	Metastasis	chr20	899	97	47244164	62904845	Loss	0.59385	3.387584	q13.13-q13.33	15.66068
OVA_048_M1	Metastasis	chr12	927	75	53292537	60164955	Del	0.625824	3.962486	q13.13-q14.1	6.872418
OVA_048_M3	Metastasis	chr12	935	75	53292643	62708636	Del	0.59385	3.387584	q13.13-q14.1	9.415993
OVA_048_M2	Metastasis	chr12	924	75	53298579	59313207	Del	0.405007	3.72502	q13.13-q14.1	6.014628
OVA_048_C1	Primary	chr12	921	74	53400440	60173363	Loss	0.682521	1.843999	q13.13-q14.1	6.772923
OVA_048_C2	Primary	chr12	925	74	53400440	60173363	Loss	0.736255	1.845483	q13.13-q14.1	6.772923
OVA_048_C3	Primary	chr12	914	73	53400440	60164955	Loss	0.715645	1.786574	q13.13-q14.1	6.764515
OVA_003_C2	Primary	chr12	1748	121	53400440	80615879	Del	0.709607	3.665802	q13.13-q21.31	27.21544
OVA_003_C2	Primary	chr3	2681	209	107940859	1.61E+08	Del	0.709607	3.665802	q13.13-q26.1	53.2804
OVA_013_C2	Primary	chr11	12	0	63481378	63533593	Loss	0.755564	3.14838	q13.1-q13.1	0.052215
OVA_013_C3	Primary	chr11	12	0	63481378	63533593	Loss	0.75175	3.001491	q13.1-q13.1	0.052215
OVA_013_C1	Primary	chr13	928	40	32313703	67205386	Del	0.701164	3.125184	q13.1-q21.32	34.89168
OVA_013_C3	Primary	chr13	929	40	32313703	67205386	Del	0.75175	3.001491	q13.1-q21.32	34.89168
OVA_013_M1	Metastasis	chr5	283	29	68902906	76028913	Del	0.621947	2.883383	q13.2-q13.3	7.126007
OVA_047_C2	Primary	chr20	904	103	50770005	62904845	Loss	0.575254	2.829045	q13.2-q13.33	12.13484
OVA_047_C1	Primary	chr20	904	103	50770782	62904845	Loss	0.576016	2.862818	q13.2-q13.33	12.13406
OVA_047_CO	Contralateral ovary	chr20	903	103	50770782	62904703	Loss	0.55613	2.930893	q13.2-q13.33	12.13392
OVA_003_C2	Primary	chr5	961	93	72359705	1.02E+08	Del	0.709607	3.665802	q13.2-q21.1	29.37567
OVA_003_C2	Primary	chr8	1007	88	68968166	1.04E+08	Del	0.709607	3.665802	q13.2-q22.3	34.85163
OVA_047_CO	Contralateral ovary	chrX	1073	49	72432838	1.19E+08	Loss	0.55613	2.930893	q13.2-q24	46.573
OVA_047_M2	Metastasis	chr3	2096	181	112052090	1.53E+08	Loss	0.599725	2.678568	q13.2-q25.2	40.82873
OVA_047_M3	Metastasis	chr3	2105	180	112052103	1.54E+08	Loss	0.605088	2.815208	q13.2-q25.2	41.92262
OVA_047_M1	Metastasis	chr3	2088	179	112068503	1.53E+08	Loss	0.624347	2.86771	q13.2-q25.2	40.81232
OVA_013_C1	Primary	chr5	2255	203	68902906	1.4E+08	Del	0.701164	3.125184	q13.2-q31.3	71.18238
OVA_013_C3	Primary	chr5	2247	204	69718560	1.4E+08	Del	0.75175	3.001491	q13.2-q31.3	70.36197
OVA_047_C2	Primary	chr5	2283	186	71015228	1.4E+08	Del	0.575254	2.829045	q13.2-q31.3	69.151
OVA_047_C1	Primary	chr5	2277	186	71146824	1.4E+08	Del	0.576016	2.862818	q13.2-q31.3	68.93371
OVA_013_C2	Primary	chr5	4145	406	68902906	1.78E+08	Del	0.755564	3.14838	q13.2-q35.3	109.1365
OVA_047_CO	Contralateral ovary	chr5	4717	462	71489695	1.81E+08	Del	0.55613	2.930893	q13.2-q35.3	109.1975
OVA_047_C2	Primary	chr3	10	0	115738506	1.19E+08	Loss	0.575254	2.829045	q13.31-q13.32	2.883017
OVA_048_M3	Metastasis	chr22	370	26	46684439	51237410	Del	0.59385	3.387584	q13.31-q13.33	4.552971
OVA_013_M1	Metastasis	chr22	382	54	46742405	51237486	Loss	0.621947	2.883383	q13.31-q13.33	4.495081
OVA_013_C1	Primary	chr22	376	53	46755682	51237364	Del	0.701164	3.125184	q13.31-q13.33	4.481682
OVA_013_C2	Primary	chr22	307	39	48023297	51237364	Loss	0.755564	3.14838	q13.31-q13.33	3.214067
OVA_047_CO	Contralateral ovary	chr3	1605	137	116569217	1.44E+08	Loss	0.55613	2.930893	q13.31-q24	27.12298
OVA_048_M1	Metastasis	chr19	924	69	45901184	50493019	Del	0.625824	3.962486	q13.32-q13.33	4.591835
OVA_048_M3	Metastasis	chr19	926	69	45901299	50498508	Del	0.59385	3.387584	q13.32-q13.33	4.597209
OVA_013_C2	Primary	chr19	769	96	46498353	50386340	Del	0.755564	3.14838	q13.32-q13.33	3.887987
OVA_013_C3	Primary	chr19	757	93	46544248	50386340	Del	0.75175	3.001491	q13.32-q13.33	3.842092
OVA_047_M1	Metastasis	chr19	2791	305	46142427	59093464	Del	0.624347	2.86771	q13.32-q13.43	12.95104
OVA_047_M2	Metastasis	chr19	2767	304	46260490	59093464	Del	0.599725	2.678568	q13.32-q13.43	12.83297
OVA_047_M3	Metastasis	chr19	2758	304	46260490	59093484	Del	0.605088	2.815208	q13.32-q13.43	12.83299
OVA_013_C1	Primary	chr19	2212	250	46627294	59093464	Del	0.701164	3.125184	q13.32-q13.43	12.46617
OVA_013_M1	Metastasis	chr19	2210	249	46627376	59093484	Del	0.621947	2.883383	q13.32-q13.43	12.46611
OVA_047_C2	Primary	chr3	1554	133	118648929	1.43E+08	Loss	0.575254	2.829045	q13.32-q23	23.89085
OVA_047_C1	Primary	chr3	1605	137	117716021	1.44E+08	Del	0.576016	2.862818	q13.32-q24	25.97618
OVA_048_M2	Metastasis	chr19	497	33	48821757	50493078	Del	0.405007	3.72502	q13.33-q13.33	1.671321
OVA_013_C3	Primary	chr20	286	36	60737812	62221784	Gain	0.75175	3.001491	q13.33-q13.33	1.483972
OVA_048_M3	Metastasis	chr19	582	74	50500697	54724635	Del	0.59385	3.387584	q13.33-q13.42	4.223938
OVA_365_C2	Primary	chr19	1945	220	50381095	59093484	Loss	0.629033	1.960121	q13.33-q13.43	8.712389
OVA_013_C2	Primary	chr19	1141	128	50387729	57325109	Del	0.755564	3.14838	q13.33-q13.43	6.93738
OVA_013_C3	Primary	chr19	1138	128	50387729	57184265	Del	0.75175	3.001491	q13.33-q13.43	6.796536
OVA_365_M3	Metastasis	chr19	1898	213	50453435	59093464	Loss	0.827915	1.890476	q13.33-q13.43	8.640029
OVA_048_M2	Metastasis	chr19	1340	165	50498078	59094003	Del	0.405007	3.72502	q13.33-q13.43	8.595925
OVA_048_M1	Metastasis	chr19	1340	165	50498080	59094003	Del	0.625824	3.962486	q13.33-q13.43	8.595923
OVA_378_M3	Metastasis	chr11	91	15	70281689	71260296	Amp	0.692693	2.569487	q13.3-q13.4	0.978607
OVA_378_M1	Metastasis	chr11	90	15	70319088	71260296	Amp	0.753235	2.555202	q13.3-q13.4	0.941208
OVA_378_M2	Metastasis	chr11	91	15	70319088	71276631	Amp	0.840932	2.531102	q13.3-q13.4	0.957543
OVA_013_M1	Metastasis	chr5	451	26	76115069	93812885	Del	0.621947	2.883383	q13.3-q15	17.69782
OVA_013_C1	Primary	chr4	314	26	74357695	79856305	Del	0.701164	3.125184	q13.3-q21.21	5.49861
OVA_013_C2	Primary	chr4	312	26	74367479	79856305	Del	0.755564	3.14838	q13.3-q21.21	5.488826
OVA_013_C3	Primary	chr4	307	26	74451092	79854414	Loss	0.75175	3.001491	q13.3-q21.21	5.403322
OVA_013_M1	Metastasis	chr4	307	26	74453632	79856305	Loss	0.621947	2.883383	q13.3-q21.21	5.402673
OVA_013_M1	Metastasis	chr13	764	26	36700239	67800536	Loss	0.621947	2.883383	q13.3-q21.32	31.1003
OVA_003_C3	Primary	chr11	3551	313	70277319	1.35E+08	Gain	0.820093	1.828085	q13.3-q25	64.57922

Sample	Tissue type	chrom	num.mark	nhet	start	end	Classificati	Purity	Ploidy	Cyto	Size_Mb
OVA_003_C1	Primary	chr4	3408	314	73414286	1.88E+08	Loss	0.852911	1.812048	q13.3-q35.2	114.2163
OVA_003_C2	Primary	chr4	3416	315	73414368	1.91E+08	Del	0.709607	3.665802	q13.3-q35.2	117.5331
OVA_003_C3	Primary	chr4	3440	319	73414368	1.88E+08	Loss	0.820093	1.828085	q13.3-q35.2	114.2162
OVA_048_M3	Metastasis	chr19	228	39	54726840	55774078	Loss	0.59385	3.387584	q13.42-q13.42	1.047238
OVA_048_M3	Metastasis	chr19	528	52	55776831	59094003	Del	0.59385	3.387584	q13.42-q13.43	3.317172
OVA_013_C3	Primary	chr19	320	29	57286602	59093484	Del	0.75175	3.001491	q13.43-q13.43	1.806882
OVA_013_C2	Primary	chr19	314	29	57325364	59093464	Del	0.755564	3.14838	q13.43-q13.43	1.7681
OVA_047_C2	Primary	chr19	40	7	59009949	59093464	Del	0.575254	2.829045	q13.43-q13.43	0.083515
OVA_047_CO	Contralateral ovary	chr19	39	7	59010457	59093484	Del	0.55613	2.930893	q13.43-q13.43	0.083027
OVA_047_C1	Primary	chr19	16	2	59067671	59093484	Del	0.576016	2.862818	q13.43-q13.43	0.025813
OVA_378_M3	Metastasis	chr11	1068	100	71276632	89956145	Loss	0.692693	2.569487	q13.4-q14.3	18.67951
OVA_378_M1	Metastasis	chr11	1071	101	71276739	89949668	Loss	0.753235	2.555202	q13.4-q14.3	18.67293
OVA_378_M2	Metastasis	chr11	1069	101	71277083	89955700	Loss	0.840932	2.531102	q13.4-q14.3	18.67862
OVA_047_CO	Contralateral ovary	chr11	1442	98	76804674	1.15E+08	Loss	0.55613	2.930893	q13.5-q23.3	38.2952
OVA_048_M1	Metastasis	chr6	1401	99	70411770	1.32E+08	Del	0.625824	3.962486	q13-q23.2	61.85992
OVA_048_M3	Metastasis	chr6	1405	99	70411770	1.32E+08	Del	0.59385	3.387584	q13-q23.2	61.8601
OVA_048_M2	Metastasis	chr6	1402	98	70506791	1.32E+08	Del	0.405007	3.72502	q13-q23.2	61.7649
OVA_013_M1	Metastasis	chr16	1824	159	57127983	88494747	Del	0.621947	2.883383	q13-q24.2	31.36676
OVA_013_C1	Primary	chr16	1825	158	57128096	88134304	Del	0.701164	3.125184	q13-q24.2	31.00621
OVA_013_C2	Primary	chr16	1872	162	57127983	88729573	Del	0.755564	3.14838	q13-q24.3	31.60159
OVA_013_C3	Primary	chr16	2175	184	57127983	90162379	Del	0.75175	3.001491	q13-q24.3	33.0344
OVA_047_C2	Primary	chr13	912	57	40230940	92536982	Del	0.575254	2.829045	q14.11-q31.3	52.30604
OVA_003_C2	Primary	chr13	698	51	45915332	80911782	Del	0.709607	3.665802	q14.13-q31.1	34.99645
OVA_003_C1	Primary	chr13	702	50	45965153	86370571	Loss	0.852911	1.812048	q14.13-q31.1	40.40542
OVA_003_C3	Primary	chr13	712	50	45965153	86370571	Loss	0.820093	1.828085	q14.13-q31.1	40.40542
OVA_013_C3	Primary	chr6	163	17	76617391	83900888	Loss	0.75175	3.001491	q14.1-q14.2	7.283497
OVA_013_C2	Primary	chr6	345	39	76617391	89871861	Loss	0.755564	3.14838	q14.1-q15	13.25447
OVA_013_C1	Primary	chr6	564	72	79581839	1.01E+08	Loss	0.701164	3.125184	q14.1-q16.3	21.58426
OVA_048_M2	Metastasis	chr12	833	64	60113910	96691171	Del	0.405007	3.72502	q14.1-q23.1	36.57726
OVA_048_M1	Metastasis	chr12	829	64	60169157	96680420	Del	0.625824	3.962486	q14.1-q23.1	36.51126
OVA_048_M3	Metastasis	chr12	824	64	62722105	96691171	Del	0.59385	3.387584	q14.1-q23.1	33.96907
OVA_378_M1	Metastasis	chr11	124	6	89955872	93754679	Amp	0.753235	2.555202	q14.3-q21	3.798807
OVA_378_M2	Metastasis	chr11	123	6	89956145	93754679	Amp	0.840932	2.531102	q14.3-q21	3.798534
OVA_378_M3	Metastasis	chr11	122	6	90015918	93754667	Amp	0.692693	2.569487	q14.3-q21	3.738749
OVA_013_C3	Primary	chr2	157	12	127818239	1.31E+08	Del	0.75175	3.001491	q14.3-q21.1	3.078995
OVA_013_M1	Metastasis	chr2	155	11	127819709	1.31E+08	Del	0.621947	2.883383	q14.3-q21.1	3.058073
OVA_013_C1	Primary	chr2	156	11	127819777	1.31E+08	Del	0.701164	3.125184	q14.3-q21.1	3.077441
OVA_013_C2	Primary	chr2	155	12	127819822	1.31E+08	Del	0.755564	3.14838	q14.3-q21.1	3.077469
OVA_047_M2	Metastasis	chr11	2333	181	91536584	1.34E+08	Del	0.599725	2.678568	q14.3-q25	42.62542
OVA_047_M1	Metastasis	chr11	2361	185	91544967	1.35E+08	Del	0.624347	2.86771	q14.3-q25	43.31169
OVA_047_M3	Metastasis	chr11	2333	177	91544967	1.34E+08	Del	0.605088	2.815208	q14.3-q25	42.64358
OVA_013_C3	Primary	chr15	28	0	38545542	39874681	Del	0.75175	3.001491	q14-q14	1.329139
OVA_048_M3	Metastasis	chr15	356	43	34355491	41766797	Del	0.59385	3.387584	q14-q15.1	7.411306
OVA_003_C2	Primary	chr15	541	39	38545580	42524065	Del	0.709607	3.665802	q14-q15.1	3.978485
OVA_003_C1	Primary	chr15	516	38	39874195	42510599	Loss	0.852911	1.812048	q14-q15.1	2.636404
OVA_013_C2	Primary	chr15	965	95	38545461	48580218	Del	0.755564	3.14838	q14-q21.1	10.03476
OVA_013_M1	Metastasis	chr15	910	91	38545580	45695612	Del	0.621947	2.883383	q14-q21.1	7.150032
OVA_013_C1	Primary	chr15	958	95	38746552	48580218	Del	0.701164	3.125184	q14-q21.1	9.833666
OVA_013_C3	Primary	chr15	935	95	39875929	48580233	Del	0.75175	3.001491	q14-q21.1	8.704304
OVA_048_M1	Metastasis	chr15	1171	108	34356163	51829812	Del	0.625824	3.962486	q14-q21.2	17.47365
OVA_003_C1	Primary	chr15	553	56	42524065	48063076	Gain	0.852911	1.812048	q15.1-q21.1	5.539011
OVA_003_C2	Primary	chr15	552	56	42528523	48063076	Loss	0.709607	3.665802	q15.1-q21.1	5.534553
OVA_003_C3	Primary	chr15	552	56	42528523	48063076	Gain	0.820093	1.828085	q15.1-q21.1	5.534553
OVA_003_M1	Metastasis	chr15	549	56	42553330	48063076	Gain	0.552447	1.852543	q15.1-q21.1	5.509746
OVA_003_M3	Metastasis	chr15	549	56	42553330	48063076	Gain	0.574876	1.865189	q15.1-q21.1	5.509746
OVA_003_M2	Metastasis	chr15	544	55	42566489	48059420	Gain	0.439877	1.867794	q15.1-q21.1	5.492931
OVA_048_M3	Metastasis	chr15	826	66	41768765	52017035	Del	0.59385	3.387584	q15.1-q21.2	10.24827
OVA_013_C2	Primary	chr6	243	35	89888569	1.01E+08	Loss	0.755564	3.14838	q15-q16.3	11.27753
OVA_013_M1	Metastasis	chr5	710	66	93820580	1.24E+08	Del	0.621947	2.883383	q15-q23.2	30.25995
OVA_047_CO	Contralateral ovary	chr6	2027	141	94124342	1.53E+08	Del	0.55613	2.930893	q16.1-q25.2	59.16906
OVA_013_C2	Primary	chr6	2542	237	101215022	1.71E+08	Del	0.755564	3.14838	q16.3-q27	69.67843
OVA_013_M1	Metastasis	chr6	2558	240	101215022	1.71E+08	Del	0.621947	2.883383	q16.3-q27	69.67783
OVA_013_C1	Primary	chr6	2560	244	101215148	1.71E+08	Del	0.701164	3.125184	q16.3-q27	69.67831
OVA_013_C3	Primary	chr6	2555	242	101215148	1.71E+08	Del	0.75175	3.001491	q16.3-q27	69.67831
OVA_003_M3	Metastasis	chr9	642	59	71628207	90584120	Amp	0.574876	1.865189	q21.11-q22.1	18.95591
OVA_365_M1	Metastasis	chr7	1670	140	86274087	1.13E+08	Amp	0.854003	1.907418	q21.11-q31.1	26.48446

Sample	Tissue type	chrom	num.mark	nhet	start	end	Classificati	Purity	Ploidy	Cyto	Size_Mb
OVA_048_M2	Metastasis	chr9	3151	290	71094487	1.38E+08	Del	0.405007	3.72502	q21.11-q34.3	66.68464
OVA_048_M3	Metastasis	chr9	3337	310	71094487	1.39E+08	Del	0.59385	3.387584	q21.11-q34.3	68.27528
OVA_365_C2	Primary	chr9	1068	106	72933598	1E+08	Loss	0.629033	1.960121	q21.12-q22.33	27.511
OVA_365_M1	Metastasis	chr9	1046	104	72933598	1.01E+08	Loss	0.854003	1.907418	q21.12-q22.33	27.74218
OVA_365_M3	Metastasis	chr9	1065	106	72961957	1E+08	Loss	0.827915	1.890476	q21.12-q22.33	27.48748
OVA_365_M3	Metastasis	chr7	1650	139	86688366	1.13E+08	Amp	0.827915	1.890476	q21.12-q31.1	26.03636
OVA_365_M2	Metastasis	chr7	2426	186	86688366	1.34E+08	Gain	0.387458	1.893344	q21.12-q33	47.67532
OVA_365_C2	Primary	chr7	4049	316	86688366	1.59E+08	Gain	0.629033	1.960121	q21.12-q36.3	72.33681
OVA_365_CO	Contralateral ovary	chr7	4049	316	86688441	1.59E+08	Gain	0.451025	1.890627	q21.12-q36.3	72.33674
OVA_003_C1	Primary	chr9	259	32	74764627	82337870	Amp	0.852911	1.812048	q21.13-q21.31	7.573243
OVA_003_C3	Primary	chr9	259	32	74764627	82337870	Amp	0.820093	1.828085	q21.13-q21.31	7.573243
OVA_047_C1	Primary	chr7	1107	76	89854446	1.02E+08	Loss	0.576016	2.862818	q21.13-q22.1	12.35584
OVA_047_C1	Primary	chr18	21	0	44221971	44560139	Del	0.576016	2.862818	q21.1-q21.1	0.338168
OVA_048_M1	Metastasis	chr18	17	0	44656650	45567131	Del	0.625824	3.962486	q21.1-q21.1	0.910481
OVA_013_M1	Metastasis	chr15	34	3	45695810	48063412	Del	0.621947	2.883383	q21.1-q21.1	2.367602
OVA_013_C1	Primary	chr15	33	0	48580627	48766314	Del	0.701164	3.125184	q21.1-q21.1	0.185687
OVA_013_C3	Primary	chr15	55	1	48580627	48905204	Del	0.75175	3.001491	q21.1-q21.1	0.324577
OVA_013_C2	Primary	chr15	208	18	48580627	51750846	Del	0.755564	3.14838	q21.1-q21.2	3.170219
OVA_013_C1	Primary	chr15	183	17	48766680	51750846	Del	0.701164	3.125184	q21.1-q21.2	2.984166
OVA_013_C3	Primary	chr15	160	16	48936811	51750846	Del	0.75175	3.001491	q21.1-q21.2	2.814035
OVA_013_M1	Metastasis	chr15	541	31	48429064	58287358	Del	0.621947	2.883383	q21.1-q21.3	9.858294
OVA_048_C3	Primary	chr18	400	33	44641541	64178933	Gain	0.715645	1.786574	q21.1-q22.1	19.53739
OVA_048_M3	Metastasis	chr18	402	33	44660923	64235726	Del	0.59385	3.387584	q21.1-q22.1	19.5748
OVA_048_M1	Metastasis	chr18	388	33	45688841	65179593	Del	0.625824	3.962486	q21.1-q22.1	19.49075
OVA_048_M2	Metastasis	chr18	386	33	45688841	65178816	Del	0.405007	3.72502	q21.1-q22.1	19.48998
OVA_047_CO	Contralateral ovary	chr2	404	47	130680470	1.38E+08	Loss	0.55613	2.930893	q21.1-q22.1	7.237452
OVA_047_M3	Metastasis	chr21	1136	134	17566807	45562985	Loss	0.605088	2.815208	q21.1-q22.3	27.99618
OVA_003_C1	Primary	chr5	309	19	101748741	1.15E+08	Loss	0.852911	1.812048	q21.1-q22.3	13.21266
OVA_003_C2	Primary	chr5	318	18	101748741	1.15E+08	Del	0.709607	3.665802	q21.1-q22.3	13.42898
OVA_003_C1	Primary	chr15	1279	95	48427204	66625395	Loss	0.852911	1.812048	q21.1-q22.31	18.19819
OVA_003_C2	Primary	chr15	1267	95	48427204	66625470	Del	0.709607	3.665802	q21.1-q22.31	18.19827
OVA_003_C3	Primary	chr15	1298	97	48427204	66679795	Loss	0.820093	1.828085	q21.1-q22.31	18.25259
OVA_047_CO	Contralateral ovary	chr18	940	76	44426790	77926886	Del	0.55613	2.930893	q21.1-q23	33.5001
OVA_047_C1	Primary	chr18	935	76	44560480	77926971	Del	0.576016	2.862818	q21.1-q23	33.36649
OVA_047_C2	Primary	chr18	897	76	44560520	77474921	Del	0.575254	2.829045	q21.1-q23	32.9144
OVA_003_C3	Primary	chr5	332	20	101735373	1.15E+08	Loss	0.820093	1.828085	q21.1-q23.1	13.47068
OVA_013_M1	Metastasis	chr2	522	39	130897218	1.52E+08	Loss	0.621947	2.883383	q21.1-q23.3	21.22122
OVA_013_C1	Primary	chr2	522	38	130897417	1.52E+08	Del	0.701164	3.125184	q21.1-q23.3	21.22103
OVA_013_C3	Primary	chr2	522	38	130897417	1.52E+08	Loss	0.75175	3.001491	q21.1-q23.3	21.22103
OVA_013_C2	Primary	chr2	526	40	130897859	1.52E+08	Del	0.755564	3.14838	q21.1-q23.3	21.31648
OVA_048_M3	Metastasis	chr2	1665	113	131266613	1.9E+08	Del	0.59385	3.387584	q21.1-q32.2	58.33288
OVA_047_CO	Contralateral ovary	chr14	3133	223	39900857	1.05E+08	Del	0.55613	2.930893	q21.1-q32.33	64.60579
OVA_047_C2	Primary	chr4	3110	282	77089557	1.91E+08	Del	0.575254	2.829045	q21.1-q35.2	113.8579
OVA_047_C1	Primary	chr4	3114	285	77095491	1.91E+08	Del	0.576016	2.862818	q21.1-q35.2	113.852
OVA_047_CO	Contralateral ovary	chr4	3081	278	77116964	1.91E+08	Del	0.55613	2.930893	q21.1-q35.2	113.8305
OVA_048_M3	Metastasis	chr4	82	5	82377666	84508448	Del	0.59385	3.387584	q21.21-q21.23	2.130782
OVA_013_C2	Primary	chr4	413	30	79860337	92240110	Del	0.755564	3.14838	q21.21-q22.1	12.37977
OVA_013_C3	Primary	chr4	537	42	79856305	1E+08	Del	0.75175	3.001491	q21.21-q23	20.27248
OVA_013_C1	Primary	chr4	544	46	79860374	1E+08	Del	0.701164	3.125184	q21.21-q23	20.27106
OVA_013_M1	Metastasis	chr4	546	45	79860374	1E+08	Del	0.621947	2.883383	q21.21-q23	20.27457
OVA_048_M1	Metastasis	chr4	2225	179	82377666	1.91E+08	Del	0.625824	3.962486	q21.21-q35.2	108.5698
OVA_048_M2	Metastasis	chr4	2223	178	82377666	1.91E+08	Del	0.405007	3.72502	q21.21-q35.2	108.5698
OVA_048_M3	Metastasis	chr4	1898	144	84511310	1.84E+08	Del	0.59385	3.387584	q21.23-q35.1	99.20952
OVA_048_M3	Metastasis	chr17	190	31	39018961	40021084	Del	0.59385	3.387584	q21.2-q21.2	1.002123
OVA_048_M1	Metastasis	chr17	186	29	39041309	40021498	Del	0.625824	3.962486	q21.2-q21.2	0.980189
OVA_013_C1	Primary	chr15	11	1	51757913	51780722	Del	0.701164	3.125184	q21.2-q21.2	0.022809
OVA_378_C1	Primary	chr2	46	2	132905758	1.33E+08	Del	0.616455	2.593914	q21.2-q21.2	0.133398
OVA_378_M1	Metastasis	chr2	49	2	132905758	1.33E+08	Del	0.753235	2.555202	q21.2-q21.2	0.169543
OVA_378_C3	Primary	chr2	45	2	132914500	1.33E+08	Del	0.517221	2.617793	q21.2-q21.2	0.124583
OVA_378_M2	Metastasis	chr2	48	2	132914587	1.33E+08	Del	0.840932	2.531102	q21.2-q21.2	0.160714
OVA_378_M3	Metastasis	chr2	46	2	132914587	1.33E+08	Del	0.692693	2.569487	q21.2-q21.2	0.155866
OVA_013_C2	Primary	chr15	157	3	51757913	55477604	Del	0.755564	3.14838	q21.2-q21.3	3.719691
OVA_013_C3	Primary	chr15	156	3	51757913	54919047	Del	0.75175	3.001491	q21.2-q21.3	3.161134
OVA_013_C1	Primary	chr15	145	2	51790979	54919047	Del	0.701164	3.125184	q21.2-q21.3	3.128068
OVA_048_M1	Metastasis	chr15	100	7	51834518	54919047	Loss	0.625824	3.962486	q21.2-q21.3	3.084529
OVA_048_M2	Metastasis	chr15	99	6	51834518	54786879	Loss	0.405007	3.72502	q21.2-q21.3	2.952361

Sample	Tissue type	chrom	num.mark	nhet	start	end	Classificati	Purity	Ploidy	Cyto	Size_Mb
OVA_048_C2	Primary	chr15	100	6	51914662	55484921	Amp	0.736255	1.845483	q21.2-q21.3	3.570259
OVA_048_C3	Primary	chr15	100	6	51914662	55484921	Amp	0.715645	1.786574	q21.2-q21.3	3.570259
OVA_048_C1	Primary	chr15	86	5	52015787	54307574	Amp	0.682521	1.843999	q21.2-q21.3	2.291787
OVA_048_M1	Metastasis	chr1	434	44	150281310	1.53E+08	Loss	0.625824	3.962486	q21.2-q21.3	3.149975
OVA_048_M2	Metastasis	chr17	335	23	40034420	41477093	Del	0.405007	3.72502	q21.2-q21.31	1.442673
OVA_048_M3	Metastasis	chr17	839	62	40021404	46196617	Del	0.59385	3.387584	q21.2-q21.32	6.175213
OVA_048_M1	Metastasis	chr17	838	61	40025285	46247926	Del	0.625824	3.962486	q21.2-q21.32	6.222641
OVA_048_M2	Metastasis	chr10	301	29	62631202	72536936	Del	0.405007	3.72502	q21.2-q22.1	9.905734
OVA_047_M1	Metastasis	chr8	485	36	85686774	1.01E+08	Loss	0.624347	2.86771	q21.2-q22.2	15.32948
OVA_048_C2	Primary	chr17	2829	249	40025285	75488768	Loss	0.736255	1.845483	q21.2-q25.3	35.46348
OVA_047_C1	Primary	chr14	3137	222	45370087	1.04E+08	Del	0.576016	2.862818	q21.2-q32.33	59.1208
OVA_047_M2	Metastasis	chr13	1148	123	60737653	1.15E+08	Del	0.599725	2.678568	q21.2-q34	54.35407
OVA_047_M1	Metastasis	chr13	1147	124	60849952	1.15E+08	Del	0.624347	2.86771	q21.2-q34	54.24177
OVA_048_M1	Metastasis	chr13	828	87	61060085	1.15E+08	Del	0.625824	3.962486	q21.2-q34	54.03164
OVA_047_M3	Metastasis	chr13	1139	124	61102589	1.15E+08	Del	0.605088	2.815208	q21.2-q34	53.98914
OVA_003_C2	Primary	chr12	21	0	80626768	80722873	Del	0.709607	3.665802	q21.31-q21.31	0.096105
OVA_048_M2	Metastasis	chr17	500	39	41568509	46153580	Del	0.405007	3.72502	q21.31-q21.32	4.585071
OVA_003_C1	Primary	chr12	1754	128	80732934	1.15E+08	Loss	0.852911	1.812048	q21.31-q24.21	34.38801
OVA_003_C3	Primary	chr12	1769	128	80732934	1.15E+08	Loss	0.820093	1.828085	q21.31-q24.21	34.3881
OVA_003_C2	Primary	chr12	1786	126	80732934	1.17E+08	Del	0.709607	3.665802	q21.31-q24.22	36.23996
OVA_378_M1	Metastasis	chr17	4485	482	41610270	81083660	Loss	0.753235	2.555202	q21.31-q25.3	39.47339
OVA_378_M2	Metastasis	chr17	4476	482	41610270	81083660	Loss	0.840932	2.531102	q21.31-q25.3	39.47339
OVA_378_M3	Metastasis	chr17	4466	480	41610270	81083660	Loss	0.692693	2.569487	q21.31-q25.3	39.47339
OVA_365_M1	Metastasis	chr17	3826	445	43474416	81188023	Amp	0.854003	1.907418	q21.31-q25.3	37.71361
OVA_365_M3	Metastasis	chr17	3826	446	43475375	81188023	Gain	0.827915	1.890476	q21.31-q25.3	37.71265
OVA_048_C1	Primary	chr17	15	2	46507417	46691718	Amp	0.682521	1.843999	q21.32-q21.32	0.184301
OVA_003_C2	Primary	chr9	271	17	84199152	90747137	Del	0.709607	3.665802	q21.32-q22.1	6.547985
OVA_013_C1	Primary	chr9	782	44	86570486	1.02E+08	Del	0.701164	3.125184	q21.32-q22.33	15.49773
OVA_013_C3	Primary	chr9	764	44	86617489	1.02E+08	Del	0.75175	3.001491	q21.32-q22.33	15.45072
OVA_013_M1	Metastasis	chr9	771	44	86617489	1.03E+08	Del	0.621947	2.883383	q21.32-q22.33	15.97748
OVA_013_C2	Primary	chr9	768	44	86894236	1.02E+08	Del	0.755564	3.14838	q21.32-q22.33	15.17463
OVA_047_C2	Primary	chrX	538	22	92928122	1.12E+08	Loss	0.575254	2.829045	q21.32-q23	18.69713
OVA_013_C3	Primary	chr12	2535	145	88390168	1.34E+08	Del	0.75175	3.001491	q21.32-q24.33	45.42077
OVA_013_C2	Primary	chr12	2528	145	88390214	1.34E+08	Del	0.755564	3.14838	q21.32-q24.33	45.42072
OVA_013_C1	Primary	chr12	2498	145	88939735	1.34E+08	Del	0.701164	3.125184	q21.32-q24.33	44.8712
OVA_048_M2	Metastasis	chr17	2830	280	46196617	81083507	Del	0.405007	3.72502	q21.32-q25.3	34.88689
OVA_048_M3	Metastasis	chr17	2830	280	46247926	81083561	Del	0.59385	3.387584	q21.32-q25.3	34.83564
OVA_048_M1	Metastasis	chr17	2829	280	46507417	81083561	Del	0.625824	3.962486	q21.32-q25.3	34.57614
OVA_048_C1	Primary	chr17	1976	183	46700352	75495529	Loss	0.682521	1.843999	q21.32-q25.3	28.79518
OVA_048_M2	Metastasis	chr13	823	86	67205386	1.15E+08	Del	0.405007	3.72502	q21.32-q34	47.88634
OVA_013_C3	Primary	chr13	1030	94	67799687	1.15E+08	Del	0.75175	3.001491	q21.32-q34	47.29164
OVA_013_C1	Primary	chr13	1032	95	67799808	1.15E+08	Del	0.701164	3.125184	q21.32-q34	47.29152
OVA_013_C2	Primary	chr13	1020	93	67799808	1.15E+08	Del	0.755564	3.14838	q21.32-q34	47.29153
OVA_013_M1	Metastasis	chr13	1030	95	67801178	1.15E+08	Del	0.621947	2.883383	q21.32-q34	47.29022
OVA_048_M3	Metastasis	chr13	818	86	67802032	1.15E+08	Del	0.59385	3.387584	q21.32-q34	47.28969
OVA_047_M3	Metastasis	chr17	446	41	47794909	55926640	Loss	0.605088	2.815208	q21.33-q22	8.131731
OVA_047_M2	Metastasis	chr17	461	41	47875826	56166499	Loss	0.599725	2.678568	q21.33-q22	8.290673
OVA_047_M1	Metastasis	chr18	498	41	59157704	77926750	Loss	0.624347	2.86771	q21.33-q23	18.76905
OVA_047_M2	Metastasis	chr18	497	40	59157903	77926862	Loss	0.599725	2.678568	q21.33-q23	18.76896
OVA_047_M3	Metastasis	chr18	495	41	59167581	77926750	Loss	0.605088	2.815208	q21.33-q23	18.75917
OVA_047_C1	Primary	chrX	534	21	96013329	1.11E+08	Loss	0.576016	2.862818	q21.33-q23	15.18226
OVA_047_M1	Metastasis	chr17	1069	78	47886508	62503211	Loss	0.624347	2.86771	q21.33-q23.3	14.6167
OVA_013_M1	Metastasis	chr12	2466	146	91366649	1.34E+08	Del	0.621947	2.883383	q21.33-q24.33	42.44429
OVA_378_M3	Metastasis	chr8	39	4	87623852	90777563	Amp	0.692693	2.569487	q21.3-q21.3	3.153711
OVA_378_M1	Metastasis	chr8	36	4	87638297	89340251	Amp	0.753235	2.555202	q21.3-q21.3	1.701954
OVA_378_M2	Metastasis	chr8	37	4	87638297	90770369	Amp	0.840932	2.531102	q21.3-q21.3	3.132072
OVA_048_M2	Metastasis	chr1	278	23	150429944	1.52E+08	Del	0.405007	3.72502	q21.3-q21.3	1.579453
OVA_048_M3	Metastasis	chr1	117	4	150429944	1.51E+08	Loss	0.59385	3.387584	q21.3-q21.3	0.633013
OVA_378_M1	Metastasis	chr1	48	4	150482145	1.51E+08	Amp	0.753235	2.555202	q21.3-q21.3	0.204384
OVA_378_C2	Primary	chr1	40	2	150485176	1.51E+08	Amp	0.607575	2.601752	q21.3-q21.3	0.201353
OVA_378_M3	Metastasis	chr1	40	2	150485176	1.51E+08	Amp	0.692693	2.569487	q21.3-q21.3	0.201353
OVA_378_C1	Primary	chr1	40	2	150485187	1.51E+08	Amp	0.616455	2.593914	q21.3-q21.3	0.201342
OVA_378_C3	Primary	chr1	40	2	150485187	1.51E+08	Amp	0.517221	2.617793	q21.3-q21.3	0.201342
OVA_378_M2	Metastasis	chr1	39	2	150522712	1.51E+08	Amp	0.840932	2.531102	q21.3-q21.3	0.163817
OVA_048_M3	Metastasis	chr1	161	19	151083657	1.52E+08	Del	0.59385	3.387584	q21.3-q21.3	0.92574
OVA_378_C1	Primary	chr1	85	5	151261189	1.52E+08	Amp	0.616455	2.593914	q21.3-q21.3	0.418711

Sample	Tissue type	chrom	num.mark	nhet	start	end	Classificati	Purity	Ploidy	Cyto	Size_Mb
OVA_378_C2	Primary	chr1	83	3	151261189	1.52E+08	Amp	0.607575	2.601752	q21.3-q21.3	0.417661
OVA_378_C3	Primary	chr1	81	3	151261189	1.52E+08	Amp	0.517221	2.617793	q21.3-q21.3	0.394648
OVA_378_M1	Metastasis	chr1	81	3	151261189	1.52E+08	Amp	0.753235	2.555202	q21.3-q21.3	0.394648
OVA_378_M2	Metastasis	chr1	81	3	151261189	1.52E+08	Amp	0.840932	2.531102	q21.3-q21.3	0.394648
OVA_378_M3	Metastasis	chr1	83	3	151261189	1.52E+08	Amp	0.692693	2.569487	q21.3-q21.3	0.417629
OVA_003_C1	Primary	chr1	233	28	151491034	1.53E+08	Gain	0.852911	1.812048	q21.3-q21.3	1.839753
OVA_003_C2	Primary	chr1	160	20	152052843	1.53E+08	Loss	0.709607	3.665802	q21.3-q21.3	1.377493
OVA_013_C1	Primary	chr1	451	23	153431435	1.56E+08	Del	0.701164	3.125184	q21.3-q22	2.28578
OVA_013_C2	Primary	chr1	451	23	153507077	1.56E+08	Del	0.755564	3.14838	q21.3-q22	2.21053
OVA_013_C3	Primary	chr1	537	24	153507176	1.56E+08	Del	0.75175	3.001491	q21.3-q22	2.679084
OVA_047_C2	Primary	chr7	781	51	96765730	1.02E+08	Loss	0.575254	2.829045	q21.3-q22.1	5.429232
OVA_013_C2	Primary	chr21	662	58	26972200	41427841	Del	0.755564	3.14838	q21.3-q22.2	14.45564
OVA_048_M3	Metastasis	chr15	343	41	54919047	63131139	Del	0.59385	3.387584	q21.3-q22.2	8.212092
OVA_013_C1	Primary	chr15	573	42	55475618	65054668	Del	0.701164	3.125184	q21.3-q22.31	9.57905
OVA_013_C3	Primary	chr15	571	41	55475618	65054668	Del	0.75175	3.001491	q21.3-q22.31	9.57905
OVA_013_C2	Primary	chr15	543	40	55484910	64687687	Del	0.755564	3.14838	q21.3-q22.31	9.202777
OVA_013_M1	Metastasis	chr15	361	29	58302732	64429048	Del	0.621947	2.883383	q21.3-q22.31	6.126316
OVA_013_C1	Primary	chr8	568	40	89131038	1.06E+08	Loss	0.701164	3.125184	q21.3-q23.1	17.29964
OVA_048_M1	Metastasis	chr1	665	50	153507077	1.57E+08	Del	0.625824	3.962486	q21.3-q23.1	3.276871
OVA_048_M2	Metastasis	chr1	1269	112	152975495	1.65E+08	Del	0.405007	3.72502	q21.3-q23.3	12.40211
OVA_048_M2	Metastasis	chr15	1054	93	54919047	74565181	Del	0.405007	3.72502	q21.3-q24.1	19.64613
OVA_048_M1	Metastasis	chr15	1053	92	55477604	74565181	Del	0.625824	3.962486	q21.3-q24.1	19.08758
OVA_003_C2	Primary	chr7	3414	269	96810439	1.52E+08	Del	0.709607	3.665802	q21.3-q36.1	54.8896
OVA_048_M3	Metastasis	chr1	3398	308	152975495	2.16E+08	Del	0.59385	3.387584	q21.3-q41	62.82664
OVA_003_C2	Primary	chr1	6070	533	153431435	2.47E+08	Del	0.709607	3.665802	q21.3-q44	93.93381
OVA_378_M1	Metastasis	chr11	141	14	93778909	96125196	Loss	0.753235	2.555202	q21-q21	2.346287
OVA_378_M2	Metastasis	chr11	141	14	93778909	96125196	Loss	0.840932	2.531102	q21-q21	2.346287
OVA_378_M3	Metastasis	chr11	141	14	93778909	96125196	Loss	0.692693	2.569487	q21-q21	2.346287
OVA_047_M2	Metastasis	chr6	18	0	111588039	1.12E+08	Del	0.599725	2.678568	q21-q21	0.106785
OVA_047_M2	Metastasis	chr16	732	74	66551066	70867990	Del	0.599725	2.678568	q21-q22.2	4.316924
OVA_047_M1	Metastasis	chr16	720	72	66582045	70867990	Del	0.624347	2.86771	q21-q22.2	4.285945
OVA_047_M3	Metastasis	chr16	708	64	66642177	70841460	Del	0.605088	2.815208	q21-q22.2	4.199283
OVA_003_C2	Primary	chr6	449	45	111067441	1.24E+08	Del	0.709607	3.665802	q21-q22.31	12.8246
OVA_047_M2	Metastasis	chr6	603	33	111695281	1.32E+08	Loss	0.599725	2.678568	q21-q23.2	19.87899
OVA_003_C1	Primary	chr16	2264	244	60393404	90232838	Loss	0.852911	1.812048	q21-q24.3	29.83943
OVA_003_C2	Primary	chr16	2251	243	61686915	90232838	Del	0.709607	3.665802	q21-q24.3	28.54592
OVA_003_C3	Primary	chr16	2256	242	61686915	90126866	Loss	0.820093	1.828085	q21-q24.3	28.43995
OVA_047_M3	Metastasis	chr6	2347	198	111628730	1.71E+08	Del	0.605088	2.815208	q21-q27	59.26477
OVA_013_C3	Primary	chr21	15	2	33312380	33691788	Loss	0.75175	3.001491	q22.11-q22.11	0.379408
OVA_013_M1	Metastasis	chr21	374	31	33103936	40250622	Loss	0.621947	2.883383	q22.11-q22.2	7.146686
OVA_013_C3	Primary	chr21	435	30	33694224	41434841	Del	0.75175	3.001491	q22.11-q22.2	7.740617
OVA_048_M1	Metastasis	chr10	71	6	71390393	72511362	Del	0.625824	3.962486	q22.1-q22.1	1.120969
OVA_048_M1	Metastasis	chr10	132	17	72513682	74098037	Del	0.625824	3.962486	q22.1-q22.1	1.584355
OVA_048_M3	Metastasis	chr10	135	17	72513682	74114592	Del	0.59385	3.387584	q22.1-q22.1	1.60091
OVA_048_M2	Metastasis	chr10	126	15	72537620	74098037	Del	0.405007	3.72502	q22.1-q22.1	1.560417
OVA_378_M3	Metastasis	chr11	149	16	99690343	1.03E+08	Amp	0.692693	2.569487	q22.1-q22.2	2.978359
OVA_378_M1	Metastasis	chr11	149	16	99690433	1.03E+08	Amp	0.753235	2.555202	q22.1-q22.2	2.978269
OVA_378_M2	Metastasis	chr11	149	16	99690433	1.03E+08	Amp	0.840932	2.531102	q22.1-q22.2	2.978269
OVA_003_C2	Primary	chr9	227	13	90747507	95768382	Del	0.709607	3.665802	q22.1-q22.31	5.020875
OVA_048_M3	Metastasis	chr18	268	28	65178816	77936017	Del	0.59385	3.387584	q22.1-q23	12.7572
OVA_048_M2	Metastasis	chr18	266	28	65179199	77936017	Del	0.405007	3.72502	q22.1-q23	12.75682
OVA_048_M1	Metastasis	chr18	265	28	65179829	77936017	Del	0.625824	3.962486	q22.1-q23	12.75619
OVA_048_M2	Metastasis	chr10	1130	87	74100765	1.02E+08	Del	0.405007	3.72502	q22.1-q24.2	27.51053
OVA_365_CO	Contralateral ovary	chr8	2016	225	97245393	1.46E+08	Gain	0.451025	1.890627	q22.1-q24.3	49.034
OVA_365_C2	Primary	chr8	1992	224	98288996	1.46E+08	Gain	0.629033	1.960121	q22.1-q24.3	47.9904
OVA_365_M2	Metastasis	chr8	1992	224	98289238	1.46E+08	Gain	0.387458	1.893344	q22.1-q24.3	47.99016
OVA_365_M1	Metastasis	chr8	1984	223	98787795	1.46E+08	Amp	0.854003	1.907418	q22.1-q24.3	47.4916
OVA_365_M3	Metastasis	chr8	1984	223	98787795	1.46E+08	Amp	0.827915	1.890476	q22.1-q24.3	47.4916
OVA_048_M1	Metastasis	chr10	2389	199	74100765	1.34E+08	Del	0.625824	3.962486	q22.1-q26.3	59.69437
OVA_048_M3	Metastasis	chr10	2393	199	74235587	1.34E+08	Del	0.59385	3.387584	q22.1-q26.3	59.72794
OVA_047_M1	Metastasis	chr6	2103	188	117900072	1.71E+08	Del	0.624347	2.86771	q22.1-q27	52.99338
OVA_013_C2	Primary	chr4	1109	63	93221990	1.41E+08	Del	0.755564	3.14838	q22.1-q31.1	47.36505
OVA_047_C1	Primary	chr7	492	33	102212828	1.16E+08	Del	0.576016	2.862818	q22.1-q31.2	13.95388
OVA_047_C2	Primary	chr7	797	56	102210364	1.27E+08	Loss	0.575254	2.829045	q22.1-q32.1	25.01964
OVA_047_CO	Contralateral ovary	chr2	2706	210	137990678	2.04E+08	Loss	0.55613	2.930893	q22.1-q33.2	66.35125
OVA_048_M3	Metastasis	chr7	1826	139	99032529	1.41E+08	Del	0.59385	3.387584	q22.1-q34	42.43182

Sample	Tissue type	chrom	num.mark	nhet	start	end	Classificati	Purity	Ploidy	Cyto	Size_Mb
OVA_047_M3	Metastasis	chr16	63	8	70867990	71218817	Loss	0.605088	2.815208	q22.2-q22.2	0.350827
OVA_047_M1	Metastasis	chr16	60	7	70883822	71196583	Loss	0.624347	2.86771	q22.2-q22.2	0.312761
OVA_047_M2	Metastasis	chr16	60	7	70883881	71196583	Loss	0.599725	2.678568	q22.2-q22.2	0.312702
OVA_013_M1	Metastasis	chr21	776	84	40250877	48084282	Del	0.621947	2.883383	q22.2-q22.3	7.833405
OVA_013_C2	Primary	chr21	703	85	41434841	48084177	Loss	0.755564	3.14838	q22.2-q22.3	6.649336
OVA_013_C3	Primary	chr21	701	85	41447103	48084282	Loss	0.75175	3.001491	q22.2-q22.3	6.637179
OVA_365_C2	Primary	chr18	272	22	68098095	77933813	Loss	0.629033	1.960121	q22.2-q23	9.835718
OVA_365_M3	Metastasis	chr18	269	22	68317984	77933813	Loss	0.827915	1.890476	q22.2-q23	9.615829
OVA_047_C2	Primary	chr16	297	8	71209504	75512671	Del	0.575254	2.829045	q22.2-q23.1	4.303167
OVA_047_CO	Contralateral ovary	chr16	290	8	71209504	75512671	Del	0.55613	2.930893	q22.2-q23.1	4.303167
OVA_047_M1	Metastasis	chr16	297	8	71209504	75512899	Del	0.624347	2.86771	q22.2-q23.1	4.303395
OVA_047_C1	Primary	chr16	291	7	71218817	75446565	Del	0.576016	2.862818	q22.2-q23.1	4.227748
OVA_047_M3	Metastasis	chr16	314	9	71220755	75634192	Del	0.605088	2.815208	q22.2-q23.1	4.413437
OVA_047_M2	Metastasis	chr16	436	24	71209504	81030671	Del	0.599725	2.678568	q22.2-q23.2	9.821167
OVA_048_M3	Metastasis	chr15	711	52	63335157	74565181	Del	0.59385	3.387584	q22.2-q24.1	11.23002
OVA_047_M1	Metastasis	chr8	792	84	101020572	1.31E+08	Gain	0.624347	2.86771	q22.2-q24.21	29.74385
OVA_047_M2	Metastasis	chr8	786	84	101051024	1.3E+08	Gain	0.599725	2.678568	q22.2-q24.21	29.44521
OVA_047_M3	Metastasis	chr8	785	83	101052261	1.3E+08	Gain	0.605088	2.815208	q22.2-q24.21	29.44404
OVA_047_C2	Primary	chr11	2009	170	102269679	1.35E+08	Loss	0.575254	2.829045	q22.2-q25	32.58698
OVA_378_M3	Metastasis	chr11	2088	211	102707305	1.35E+08	Loss	0.692693	2.569487	q22.2-q25	32.14923
OVA_378_M1	Metastasis	chr11	2093	211	102707366	1.35E+08	Loss	0.753235	2.555202	q22.2-q25	32.14917
OVA_378_M2	Metastasis	chr11	2092	211	102707366	1.35E+08	Loss	0.840932	2.531102	q22.2-q25	32.1493
OVA_003_C1	Primary	chr10	2319	196	75487081	1.17E+08	Loss	0.852911	1.812048	q22.2-q25.3	41.17217
OVA_003_C2	Primary	chr10	2302	191	75487081	1.17E+08	Del	0.709607	3.665802	q22.2-q25.3	41.17217
OVA_003_C3	Primary	chr10	2328	196	75487081	1.17E+08	Loss	0.820093	1.828085	q22.2-q25.3	41.17217
OVA_003_C2	Primary	chr2	1836	108	143913203	1.84E+08	Del	0.709607	3.665802	q22.2-q32.1	40.55897
OVA_013_M1	Metastasis	chr15	40	0	64429924	65033453	Del	0.621947	2.883383	q22.31-q22.31	0.603529
OVA_013_C2	Primary	chr15	20	0	64689851	65040719	Del	0.755564	3.14838	q22.31-q22.31	0.350868
OVA_047_C1	Primary	chr15	499	25	63915986	70371772	Loss	0.576016	2.862818	q22.31-q23	6.455786
OVA_047_C2	Primary	chr15	503	25	63929232	70952594	Loss	0.575254	2.829045	q22.31-q23	7.023362
OVA_047_CO	Contralateral ovary	chr15	502	25	63935147	70959576	Loss	0.55613	2.930893	q22.31-q23	7.024429
OVA_013_M1	Metastasis	chr15	801	37	65040719	75982428	Loss	0.621947	2.883383	q22.31-q24.2	10.94171
OVA_003_C2	Primary	chr15	1172	99	66629403	81637284	Del	0.709607	3.665802	q22.31-q25.1	15.00788
OVA_013_C2	Primary	chr15	1273	64	65042560	83952002	Del	0.755564	3.14838	q22.31-q25.2	18.90944
OVA_013_C1	Primary	chr15	1270	63	65108074	84116334	Del	0.701164	3.125184	q22.31-q25.2	19.00826
OVA_013_C3	Primary	chr15	1270	63	65108074	83951977	Loss	0.75175	3.001491	q22.31-q25.2	18.8439
OVA_003_C2	Primary	chr6	1352	135	124759050	1.57E+08	Del	0.709607	3.665802	q22.31-q25.3	32.64671
OVA_003_C3	Primary	chr6	1371	139	124759050	1.57E+08	Loss	0.820093	1.828085	q22.31-q25.3	32.43381
OVA_003_C1	Primary	chr6	1355	136	125232359	1.57E+08	Loss	0.852911	1.812048	q22.31-q25.3	32.1734
OVA_003_C2	Primary	chr9	2605	204	95772641	1.34E+08	Del	0.709607	3.665802	q22.31-q34.12	37.99635
OVA_003_C3	Primary	chr9	2613	205	95772641	1.34E+08	Loss	0.820093	1.828085	q22.31-q34.12	37.99648
OVA_003_C1	Primary	chr9	2594	203	95772666	1.34E+08	Loss	0.852911	1.812048	q22.31-q34.12	37.99642
OVA_047_C1	Primary	chr9	1605	136	97877088	1.29E+08	Del	0.576016	2.862818	q22.32-q33.3	31.50073
OVA_047_C2	Primary	chr9	1599	136	97879465	1.29E+08	Del	0.575254	2.829045	q22.32-q33.3	31.30463
OVA_047_CO	Contralateral ovary	chr9	1588	133	97934359	1.29E+08	Del	0.55613	2.930893	q22.32-q33.3	31.44346
OVA_047_M2	Metastasis	chr9	1136	115	100672338	1.25E+08	Loss	0.599725	2.678568	q22.33-q33.2	24.28997
OVA_013_C1	Primary	chr9	919	102	102068502	1.24E+08	Del	0.701164	3.125184	q22.33-q33.2	21.97537
OVA_013_C3	Primary	chr9	921	103	102068502	1.24E+08	Loss	0.75175	3.001491	q22.33-q33.2	21.97537
OVA_013_C2	Primary	chr9	918	103	102595069	1.24E+08	Del	0.755564	3.14838	q22.33-q33.2	21.4488
OVA_013_M1	Metastasis	chr9	917	103	102595685	1.24E+08	Loss	0.621947	2.883383	q22.33-q33.2	21.44818
OVA_047_M1	Metastasis	chr9	2264	191	100672338	1.35E+08	Loss	0.624347	2.86771	q22.33-q34.13	33.85401
OVA_047_M3	Metastasis	chr9	3437	223	100672338	1.41E+08	Loss	0.605088	2.815208	q22.33-q34.3	40.39937
OVA_047_M3	Metastasis	chr21	467	58	45563267	48084282	Loss	0.605088	2.815208	q22.3-q22.3	2.521015
OVA_047_C2	Primary	chr21	453	54	45705988	48084304	Loss	0.575254	2.829045	q22.3-q22.3	2.378316
OVA_013_C1	Primary	chr21	263	30	46193565	48084304	Loss	0.701164	3.125184	q22.3-q22.3	1.890739
OVA_365_M1	Metastasis	chr18	261	21	69447011	77933813	Loss	0.854003	1.907418	q22.3-q23	8.486802
OVA_003_C2	Primary	chr8	88	12	103840893	1.06E+08	Del	0.709607	3.665802	q22.3-q23.1	2.615607
OVA_047_M1	Metastasis	chr7	420	30	105615426	1.21E+08	Loss	0.624347	2.86771	q22.3-q31.31	15.07563
OVA_003_C2	Primary	chr5	1955	154	115188660	1.49E+08	Del	0.709607	3.665802	q22.3-q32	34.10584
OVA_003_C2	Primary	chr14	2262	217	56958247	96887282	Del	0.709607	3.665802	q22.3-q32.2	39.92904
OVA_047_M3	Metastasis	chr7	952	76	105656263	1.31E+08	Loss	0.605088	2.815208	q22.3-q32.3	25.69104
OVA_047_M2	Metastasis	chr7	931	74	106508264	1.31E+08	Loss	0.599725	2.678568	q22.3-q32.3	24.73282
OVA_047_C2	Primary	chr14	2712	198	55619429	1.05E+08	Del	0.575254	2.829045	q22.3-q32.33	48.88722
OVA_048_M3	Metastasis	chr14	2166	190	57672715	1.04E+08	Del	0.59385	3.387584	q22.3-q32.33	46.77981
OVA_047_M3	Metastasis	chr17	17	0	55927186	56166647	Del	0.605088	2.815208	q22-q22	0.239461
OVA_047_CO	Contralateral ovary	chr17	334	19	53342933	58928811	Del	0.55613	2.930893	q22-q23.2	5.585878

Sample	Tissue type	chrom	num.mark	nhet	start	end	Classificati	Purity	Ploidy	Cyto	Size_Mb
OVA_047_C1	Primary	chr17	354	19	53491455	59001886	Del	0.576016	2.862818	q22-q23.2	5.510431
OVA_047_C2	Primary	chr17	350	19	53638804	59104873	Del	0.575254	2.829045	q22-q23.2	5.466069
OVA_047_M3	Metastasis	chr17	601	35	56274734	62510497	Loss	0.605088	2.815208	q22-q23.3	6.235763
OVA_047_M2	Metastasis	chr17	638	37	56232719	62777200	Loss	0.599725	2.678568	q22-q24.1	6.544481
OVA_047_CO	Contralateral ovary	chr12	2294	181	95660320	1.32E+08	Del	0.55613	2.930893	q22-q24.33	36.53899
OVA_013_C1	Primary	chr1	5123	474	155717677	2.49E+08	Loss	0.701164	3.125184	q22-q44	93.49495
OVA_013_C2	Primary	chr1	5119	474	155718272	2.49E+08	Loss	0.755564	3.14838	q22-q44	93.49436
OVA_013_C3	Primary	chr10	46	7	82331231	87362141	Loss	0.75175	3.001491	q23.1-q23.1	5.03091
OVA_013_C2	Primary	chr10	34	7	84738781	87362282	Loss	0.755564	3.14838	q23.1-q23.1	2.623501
OVA_013_C1	Primary	chr10	30	7	85936160	87362282	Loss	0.701164	3.125184	q23.1-q23.1	1.426122
OVA_013_M1	Metastasis	chr10	26	7	85962720	87362141	Loss	0.621947	2.883383	q23.1-q23.1	1.399421
OVA_047_C1	Primary	chr1	646	68	156785771	1.62E+08	Gain	0.576016	2.862818	q23.1-q23.3	4.729047
OVA_048_M1	Metastasis	chr1	565	55	156785771	1.63E+08	Del	0.625824	3.962486	q23.1-q23.3	6.524363
OVA_047_C2	Primary	chr1	543	62	156814286	1.61E+08	Gain	0.575254	2.829045	q23.1-q23.3	4.235278
OVA_048_C1	Primary	chr12	526	26	96679853	1.09E+08	Gain	0.682521	1.843999	q23.1-q24.11	12.81187
OVA_048_C2	Primary	chr12	524	26	96691171	1.09E+08	Gain	0.736255	1.845483	q23.1-q24.11	12.80065
OVA_048_M1	Metastasis	chr12	524	26	96691171	1.09E+08	Loss	0.625824	3.962486	q23.1-q24.11	12.80065
OVA_048_C3	Primary	chr12	523	26	96717894	1.09E+08	Amp	0.715645	1.786574	q23.1-q24.11	12.77383
OVA_048_M2	Metastasis	chr12	522	26	96717894	1.09E+08	Loss	0.405007	3.72502	q23.1-q24.11	12.77271
OVA_047_C1	Primary	chr16	1083	133	75448480	90233312	Del	0.576016	2.862818	q23.1-q24.3	14.78483
OVA_047_C2	Primary	chr16	1078	132	75513041	90233312	Del	0.575254	2.829045	q23.1-q24.3	14.72027
OVA_047_CO	Contralateral ovary	chr16	1075	132	75513041	90233312	Del	0.55613	2.930893	q23.1-q24.3	14.72027
OVA_047_M1	Metastasis	chr16	1077	131	75513153	90233312	Del	0.624347	2.86771	q23.1-q24.3	14.72016
OVA_047_M3	Metastasis	chr16	1057	131	75636966	90233312	Del	0.605088	2.815208	q23.1-q24.3	14.59635
OVA_003_M1	Metastasis	chr8	1029	88	106431030	1.43E+08	Amp	0.552447	1.852543	q23.1-q24.3	36.96529
OVA_003_C1	Primary	chr8	1027	87	106573646	1.43E+08	Amp	0.852911	1.812048	q23.1-q24.3	36.82267
OVA_003_C3	Primary	chr8	1027	87	106573646	1.43E+08	Amp	0.820093	1.828085	q23.1-q24.3	36.82267
OVA_003_M2	Metastasis	chr8	1032	89	106628856	1.43E+08	Gain	0.439877	1.867794	q23.1-q24.3	36.79831
OVA_003_M3	Metastasis	chr8	1024	87	106628856	1.43E+08	Amp	0.574876	1.865189	q23.1-q24.3	36.73699
OVA_013_C1	Primary	chr10	1766	150	87373408	1.2E+08	Del	0.701164	3.125184	q23.1-q26.11	32.72237
OVA_013_C2	Primary	chr10	1762	147	87373408	1.2E+08	Del	0.755564	3.14838	q23.1-q26.11	32.72237
OVA_013_C3	Primary	chr10	1765	148	87373408	1.2E+08	Del	0.75175	3.001491	q23.1-q26.11	32.72133
OVA_013_M1	Metastasis	chr10	1766	151	87373408	1.2E+08	Del	0.621947	2.883383	q23.1-q26.11	32.72132
OVA_047_CO	Contralateral ovary	chr17	57	1	58946050	60062325	Del	0.55613	2.930893	q23.2-q23.2	1.116275
OVA_047_C1	Primary	chr17	45	1	59024649	60023969	Del	0.576016	2.862818	q23.2-q23.2	0.99932
OVA_047_C2	Primary	chr17	42	1	59112109	60023969	Del	0.575254	2.829045	q23.2-q23.2	0.91186
OVA_365_CO	Contralateral ovary	chr12	15	0	102153860	1.02E+08	Gain	0.451025	1.890627	q23.2-q23.2	0.2592
OVA_013_C1	Primary	chr11	10	0	113803108	1.14E+08	Loss	0.701164	3.125184	q23.2-q23.2	0.054239
OVA_013_C1	Primary	chr11	17	0	113860350	1.14E+08	Del	0.701164	3.125184	q23.2-q23.2	0.413323
OVA_013_M1	Metastasis	chr5	14	1	124410605	1.26E+08	Del	0.621947	2.883383	q23.2-q23.2	1.418045
OVA_048_C2	Primary	chr6	17	1	132618986	1.33E+08	Gain	0.736255	1.845483	q23.2-q23.2	0.272584
OVA_013_M1	Metastasis	chr11	876	98	113802600	1.25E+08	Loss	0.621947	2.883383	q23.2-q24.2	11.20685
OVA_013_C3	Primary	chr11	944	118	113846077	1.26E+08	Loss	0.75175	3.001491	q23.2-q24.2	12.23468
OVA_013_C2	Primary	chr11	943	118	113848442	1.26E+08	Del	0.755564	3.14838	q23.2-q24.2	12.23232
OVA_013_C1	Primary	chr11	848	97	114276596	1.25E+08	Del	0.701164	3.125184	q23.2-q24.2	10.73286
OVA_047_M2	Metastasis	chr16	941	116	81031080	90233312	Loss	0.599725	2.678568	q23.2-q24.3	9.202232
OVA_047_C1	Primary	chr6	987	83	131571649	1.54E+08	Del	0.576016	2.862818	q23.2-q25.2	22.84273
OVA_047_C2	Primary	chr6	964	82	131571663	1.53E+08	Del	0.575254	2.829045	q23.2-q25.2	21.50155
OVA_047_C1	Primary	chr17	2552	195	60024391	81188028	Loss	0.576016	2.862818	q23.2-q25.3	21.16364
OVA_047_C2	Primary	chr17	2555	196	60024391	81188041	Loss	0.575254	2.829045	q23.2-q25.3	21.16365
OVA_047_CO	Contralateral ovary	chr17	2542	194	60087932	81187950	Loss	0.55613	2.930893	q23.2-q25.3	21.10002
OVA_047_M2	Metastasis	chr6	1741	166	131602602	1.71E+08	Del	0.599725	2.678568	q23.2-q27	39.29089
OVA_048_M1	Metastasis	chr6	1390	153	132618986	1.71E+08	Del	0.625824	3.962486	q23.2-q27	38.27447
OVA_048_M2	Metastasis	chr6	1391	153	132618986	1.71E+08	Del	0.405007	3.72502	q23.2-q27	38.27447
OVA_048_M3	Metastasis	chr6	1390	153	132618986	1.71E+08	Del	0.59385	3.387584	q23.2-q27	38.27447
OVA_013_M1	Metastasis	chr5	788	79	125880746	1.4E+08	Loss	0.621947	2.883383	q23.2-q31.3	14.20454
OVA_047_C1	Primary	chr11	327	38	115109159	1.19E+08	Loss	0.576016	2.862818	q23.3-q23.3	3.423596
OVA_047_C1	Primary	chr1	547	47	161569523	1.73E+08	Loss	0.576016	2.862818	q23.3-q24.3	10.93325
OVA_047_C2	Primary	chr1	546	47	161569523	1.73E+08	Loss	0.575254	2.829045	q23.3-q24.3	10.9333
OVA_047_CO	Contralateral ovary	chr1	546	47	161569523	1.73E+08	Loss	0.55613	2.930893	q23.3-q24.3	10.93325
OVA_047_CO	Contralateral ovary	chr11	1380	130	115102245	1.35E+08	Del	0.55613	2.930893	q23.3-q25	19.75442
OVA_047_C1	Primary	chr11	1054	92	118533728	1.35E+08	Del	0.576016	2.862818	q23.3-q25	16.32293
OVA_048_M1	Metastasis	chr1	2109	192	164529388	2.15E+08	Del	0.625824	3.962486	q23.3-q41	50.09584
OVA_048_M2	Metastasis	chr1	2087	190	165389285	2.15E+08	Del	0.405007	3.72502	q23.3-q41	49.16041
OVA_378_C3	Primary	chr18	149	11	74583753	77936017	Loss	0.517221	2.617793	q23-q23	3.352264
OVA_378_C1	Primary	chr18	147	11	74592174	77936017	Loss	0.616455	2.593914	q23-q23	3.343843

Sample	Tissue type	chrom	num.mark	nhet	start	end	Classificati	Purity	Ploidy	Cyto	Size_Mb
OVA_378_C2	Primary	chr18	147	11	74592174	77936017	Loss	0.607575	2.601752	q23-q23	3.343843
OVA_378_M1	Metastasis	chr18	147	11	74592174	77936017	Loss	0.753235	2.555202	q23-q23	3.343843
OVA_378_M2	Metastasis	chr18	147	11	74592174	77936017	Loss	0.840932	2.531102	q23-q23	3.343843
OVA_378_M3	Metastasis	chr18	145	11	74607264	77936017	Loss	0.692693	2.569487	q23-q23	3.328753
OVA_047_C2	Primary	chr18	40	0	77475368	77926862	Del	0.575254	2.829045	q23-q23	0.451494
OVA_047_C1	Primary	chrX	212	14	111624515	1.19E+08	Loss	0.576016	2.862818	q23-q24	7.412782
OVA_047_C2	Primary	chrX	208	14	111698036	1.19E+08	Loss	0.575254	2.829045	q23-q24	7.307835
OVA_047_C2	Primary	chr3	46	4	142542415	1.44E+08	Loss	0.575254	2.829045	q23-q24	1.166004
OVA_048_C1	Primary	chr15	241	18	69223059	74427318	Amp	0.682521	1.843999	q23-q24.1	5.204259
OVA_048_C2	Primary	chr15	263	19	69223059	74565181	Amp	0.736255	1.845483	q23-q24.1	5.342122
OVA_048_C3	Primary	chr15	263	19	69223059	74565181	Amp	0.715645	1.786574	q23-q24.1	5.342122
OVA_047_C1	Primary	chr15	576	31	70387672	77450964	Del	0.576016	2.862818	q23-q24.3	7.063292
OVA_047_C2	Primary	chr15	1953	168	70959354	1.02E+08	Del	0.575254	2.829045	q23-q26.3	31.32882
OVA_047_CO	Contralateral ovary	chr15	1944	167	70959711	1.02E+08	Del	0.55613	2.930893	q23-q26.3	31.32846
OVA_048_M3	Metastasis	chrX	1069	70	110463693	1.55E+08	Del	0.59385	3.387584	q23-q28	44.29058
OVA_013_C1	Primary	chr4	981	51	100134888	1.4E+08	Del	0.701164	3.125184	q23-q31.1	40.08795
OVA_013_C3	Primary	chr4	998	52	100134888	1.41E+08	Del	0.75175	3.001491	q23-q31.1	40.45224
OVA_013_M1	Metastasis	chr4	978	52	100201190	1.4E+08	Del	0.621947	2.883383	q23-q31.1	40.02186
OVA_048_M1	Metastasis	chr12	15	0	109509481	1.1E+08	Del	0.625824	3.962486	q24.11-q24.11	0.038257
OVA_047_C1	Primary	chr8	358	36	118159388	1.31E+08	Gain	0.576016	2.862818	q24.11-q24.21	12.6051
OVA_048_M1	Metastasis	chr12	1217	99	109577344	1.29E+08	Del	0.625824	3.962486	q24.11-q24.32	19.32266
OVA_048_M3	Metastasis	chr12	1512	115	109490601	1.34E+08	Del	0.59385	3.387584	q24.11-q24.33	24.32033
OVA_048_M2	Metastasis	chr12	1511	113	109491818	1.34E+08	Del	0.405007	3.72502	q24.11-q24.33	24.31912
OVA_048_C1	Primary	chr12	1507	114	109509481	1.34E+08	Loss	0.682521	1.843999	q24.11-q24.33	24.3014
OVA_047_C2	Primary	chr12	1784	125	109577735	1.33E+08	Del	0.575254	2.829045	q24.11-q24.33	23.86851
OVA_047_M3	Metastasis	chr12	1526	98	112221055	1.34E+08	Loss	0.605088	2.815208	q24.12-q24.33	21.58988
OVA_047_M1	Metastasis	chr12	1443	97	113269597	1.34E+08	Loss	0.624347	2.86771	q24.13-q24.33	20.54128
OVA_047_M2	Metastasis	chr12	1424	94	113379598	1.34E+08	Loss	0.599725	2.678568	q24.13-q24.33	20.43128
OVA_048_C2	Primary	chr15	216	11	74611045	77310445	Loss	0.736255	1.845483	q24.1-q24.3	2.6994
OVA_048_M1	Metastasis	chr15	211	11	74611045	77224726	Del	0.625824	3.962486	q24.1-q24.3	2.613681
OVA_048_M2	Metastasis	chr15	212	11	74611045	77227960	Del	0.405007	3.72502	q24.1-q24.3	2.616915
OVA_048_M3	Metastasis	chr15	210	11	74611045	77224726	Del	0.59385	3.387584	q24.1-q24.3	2.613681
OVA_365_M3	Metastasis	chr11	659	69	123597194	1.35E+08	Loss	0.827915	1.890476	q24.1-q25	11.25947
OVA_365_M1	Metastasis	chr11	633	66	123597270	1.35E+08	Loss	0.854003	1.907418	q24.1-q25	11.25939
OVA_047_M2	Metastasis	chr8	397	44	130760850	1.42E+08	Amp	0.599725	2.678568	q24.21-q24.3	11.68393
OVA_047_M3	Metastasis	chr8	1126	119	130760887	1.46E+08	Amp	0.605088	2.815208	q24.21-q24.3	15.51859
OVA_047_CO	Contralateral ovary	chr8	1113	114	130762837	1.46E+08	Amp	0.55613	2.930893	q24.21-q24.3	15.44063
OVA_047_C1	Primary	chr8	1110	114	130764972	1.46E+08	Amp	0.576016	2.862818	q24.21-q24.3	15.43849
OVA_047_M1	Metastasis	chr8	1121	119	130765011	1.46E+08	Amp	0.624347	2.86771	q24.21-q24.3	15.51447
OVA_013_C1	Primary	chr8	894	128	131172081	1.46E+08	Del	0.701164	3.125184	q24.21-q24.3	15.10732
OVA_013_C2	Primary	chr8	890	128	131172081	1.46E+08	Del	0.755564	3.14838	q24.21-q24.3	15.10732
OVA_013_C3	Primary	chr8	894	128	131172081	1.46E+08	Del	0.75175	3.001491	q24.21-q24.3	15.10732
OVA_013_M1	Metastasis	chr8	896	128	131172081	1.46E+08	Del	0.621947	2.883383	q24.21-q24.3	15.10732
OVA_003_C2	Primary	chr12	1302	105	117013700	1.34E+08	Del	0.709607	3.665802	q24.22-q24.33	16.79718
OVA_047_C2	Primary	chr8	891	99	136659195	1.46E+08	Gain	0.575254	2.829045	q24.23-q24.3	9.620284
OVA_047_M1	Metastasis	chr14	78	9	72926469	73731012	Loss	0.624347	2.86771	q24.2-q24.2	0.804543
OVA_047_M3	Metastasis	chr14	115	15	72921127	74086415	Loss	0.605088	2.815208	q24.2-q24.3	1.165288
OVA_013_C1	Primary	chr16	348	25	88494747	90162379	Del	0.701164	3.125184	q24.2-q24.3	1.667632
OVA_013_M1	Metastasis	chr16	347	25	88495407	90162333	Del	0.621947	2.883383	q24.2-q24.3	1.666926
OVA_048_M1	Metastasis	chr16	330	36	88496008	90232838	Del	0.625824	3.962486	q24.2-q24.3	1.73683
OVA_048_M2	Metastasis	chr16	318	35	88504618	90232838	Del	0.405007	3.72502	q24.2-q24.3	1.72822
OVA_048_M3	Metastasis	chr16	316	35	88505635	90232838	Del	0.59385	3.387584	q24.2-q24.3	1.727203
OVA_013_M1	Metastasis	chr11	379	49	125128531	1.35E+08	Del	0.621947	2.883383	q24.2-q25	9.72813
OVA_013_C1	Primary	chr11	380	49	125128655	1.35E+08	Del	0.701164	3.125184	q24.2-q25	9.727956
OVA_013_C2	Primary	chr11	308	28	126081336	1.35E+08	Del	0.755564	3.14838	q24.2-q25	8.775325
OVA_013_C3	Primary	chr11	307	28	126081336	1.35E+08	Del	0.75175	3.001491	q24.2-q25	8.775325
OVA_013_M1	Metastasis	chr15	766	61	76016583	90167903	Del	0.621947	2.883383	q24.2-q26.1	14.15132
OVA_048_M2	Metastasis	chr10	1152	97	101639682	1.28E+08	Del	0.405007	3.72502	q24.2-q26.2	26.20407
OVA_047_M1	Metastasis	chr15	1549	143	75890901	1.03E+08	Loss	0.624347	2.86771	q24.2-q26.3	26.62536
OVA_047_M1	Metastasis	chr14	1248	110	73731262	97321689	Loss	0.624347	2.86771	q24.2-q32.2	23.59043
OVA_048_M1	Metastasis	chr12	279	15	129100820	1.34E+08	Del	0.625824	3.962486	q24.32-q24.33	4.710115
OVA_365_C2	Primary	chr12	366	46	129348666	1.34E+08	Gain	0.629033	1.960121	q24.33-q24.33	4.462269
OVA_365_M1	Metastasis	chr12	366	46	129348761	1.34E+08	Amp	0.854003	1.907418	q24.33-q24.33	4.462174
OVA_365_M2	Metastasis	chr12	366	46	129348770	1.34E+08	Gain	0.387458	1.893344	q24.33-q24.33	4.46211
OVA_365_CO	Contralateral ovary	chr12	365	46	129373247	1.34E+08	Gain	0.451025	1.890627	q24.33-q24.33	4.437688
OVA_365_M3	Metastasis	chr12	365	46	129373247	1.34E+08	Amp	0.827915	1.890476	q24.33-q24.33	4.437688

Sample	Tissue type	chrom	num.mark	nhet	start	end	Classificati	Purity	Ploidy	Cyto	Size_Mb
OVA_047_CO	Contralateral ovary	chr12	243	9	132204036	1.34E+08	Loss	0.55613	2.930893	q24.33-q24.33	1.606844
OVA_047_C2	Primary	chr12	25	1	133447242	1.34E+08	Del	0.575254	2.829045	q24.33-q24.33	0.363693
OVA_013_C2	Primary	chr16	294	21	88745019	90162412	Del	0.755564	3.14838	q24.3-q24.3	1.417393
OVA_047_M2	Metastasis	chr8	729	75	142444900	1.46E+08	Amp	0.599725	2.678568	q24.3-q24.3	3.834579
OVA_003_C2	Primary	chr8	693	62	143356261	1.46E+08	Del	0.709607	3.665802	q24.3-q24.3	2.923218
OVA_047_C1	Primary	chr8	11	5	146218441	1.46E+08	Amp	0.576016	2.862818	q24.3-q24.3	0.061038
OVA_047_CO	Contralateral ovary	chr8	11	5	146218441	1.46E+08	Gain	0.55613	2.930893	q24.3-q24.3	0.060955
OVA_047_C2	Primary	chr1	412	27	172520692	1.8E+08	Gain	0.575254	2.829045	q24.3-q25.3	7.878719
OVA_048_C1	Primary	chr15	1114	115	77224726	1.02E+08	Gain	0.682521	1.843999	q24.3-q26.3	25.16427
OVA_048_C3	Primary	chr15	1113	115	77227960	1.02E+08	Amp	0.715645	1.786574	q24.3-q26.3	25.16103
OVA_048_M1	Metastasis	chr15	1113	115	77227960	1.02E+08	Del	0.625824	3.962486	q24.3-q26.3	25.16103
OVA_048_M3	Metastasis	chr15	1113	115	77227960	1.02E+08	Del	0.59385	3.387584	q24.3-q26.3	25.16103
OVA_048_M2	Metastasis	chr15	1112	115	77240865	1.02E+08	Del	0.405007	3.72502	q24.3-q26.3	25.14813
OVA_048_C2	Primary	chr15	1109	115	77317660	1.02E+08	Amp	0.736255	1.845483	q24.3-q26.3	25.07133
OVA_047_C1	Primary	chr15	1273	132	77471226	1.01E+08	Del	0.576016	2.862818	q24.3-q26.3	23.62808
OVA_365_M1	Metastasis	chr6	1105	133	146977963	1.71E+08	Loss	0.854003	1.907418	q24.3-q27	23.91553
OVA_365_M3	Metastasis	chr6	1108	134	146977963	1.71E+08	Loss	0.827915	1.890476	q24.3-q27	23.91553
OVA_048_C2	Primary	chr6	957	130	146987930	1.71E+08	Gain	0.736255	1.845483	q24.3-q27	23.90552
OVA_048_C2	Primary	chr14	578	51	74764675	91791301	Gain	0.736255	1.845483	q24.3-q32.11	17.02663
OVA_047_M2	Metastasis	chr14	1698	134	74008412	1.05E+08	Loss	0.599725	2.678568	q24.3-q32.33	30.55151
OVA_047_M3	Metastasis	chr14	1688	132	74111783	1.05E+08	Loss	0.605088	2.815208	q24.3-q32.33	30.45011
OVA_365_M3	Metastasis	chr2	2068	110	168074705	2.12E+08	Gain	0.827915	1.890476	q24.3-q34	43.46796
OVA_365_M1	Metastasis	chr2	2068	110	168074706	2.12E+08	Amp	0.854003	1.907418	q24.3-q34	43.46796
OVA_013_C2	Primary	chr3	297	19	143513833	1.55E+08	Del	0.755564	3.14838	q24-q25.2	11.28901
OVA_013_C1	Primary	chr3	301	18	143550956	1.55E+08	Del	0.701164	3.125184	q24-q25.2	11.25117
OVA_013_C3	Primary	chr3	384	20	143513833	1.57E+08	Del	0.75175	3.001491	q24-q25.31	13.36407
OVA_013_M1	Metastasis	chr3	384	20	143567018	1.57E+08	Del	0.621947	2.883383	q24-q25.31	13.31069
OVA_003_C2	Primary	chr17	49	3	72200115	72368512	Del	0.709607	3.665802	q25.1-q25.1	0.168397
OVA_003_C1	Primary	chr17	172	14	72205586	73000061	Gain	0.852911	1.812048	q25.1-q25.1	0.794475
OVA_047_C2	Primary	chr10	11	2	111625016	1.12E+08	Del	0.575254	2.829045	q25.1-q25.1	0.05822
OVA_003_C1	Primary	chr15	156	18	81561897	84722706	Gain	0.852911	1.812048	q25.1-q25.2	3.160809
OVA_003_C3	Primary	chr15	155	18	81582319	84770808	Gain	0.820093	1.828085	q25.1-q25.2	3.188489
OVA_003_C2	Primary	chr15	124	8	81641814	84705645	Del	0.709607	3.665802	q25.1-q25.2	3.063831
OVA_047_CO	Contralateral ovary	chr10	65	6	111631665	1.13E+08	Del	0.55613	2.930893	q25.1-q25.2	1.02855
OVA_013_C3	Primary	chr17	1549	137	71375388	81187950	Loss	0.75175	3.001491	q25.1-q25.3	9.812562
OVA_013_C2	Primary	chr17	1503	129	72245471	81187975	Loss	0.755564	3.14838	q25.1-q25.3	8.942504
OVA_013_M1	Metastasis	chr17	1499	128	72281367	81188023	Loss	0.621947	2.883383	q25.1-q25.3	8.906656
OVA_013_C1	Primary	chr17	1498	128	72295966	81188023	Loss	0.701164	3.125184	q25.1-q25.3	8.892057
OVA_003_C2	Primary	chr17	745	79	72369809	76099466	Del	0.709607	3.665802	q25.1-q25.3	3.729657
OVA_047_C2	Primary	chr10	823	59	111701363	1.28E+08	Loss	0.575254	2.829045	q25.1-q26.2	16.02548
OVA_003_M1	Metastasis	chr15	1019	84	81558045	1.01E+08	Gain	0.552447	1.852543	q25.1-q26.3	19.89069
OVA_047_C1	Primary	chr10	1245	111	111631665	1.36E+08	Loss	0.576016	2.862818	q25.1-q26.3	23.8691
OVA_003_M3	Metastasis	chr15	122	6	82387776	84795184	Gain	0.574876	1.865189	q25.2-q25.2	2.407408
OVA_047_M1	Metastasis	chr3	15	0	153203925	1.54E+08	Loss	0.624347	2.86771	q25.2-q25.2	0.791601
OVA_047_CO	Contralateral ovary	chr6	15	0	153296479	1.54E+08	Del	0.55613	2.930893	q25.2-q25.2	1.114766
OVA_047_M3	Metastasis	chr15	187	22	83710606	86313731	Loss	0.605088	2.815208	q25.2-q25.3	2.603125
OVA_013_C3	Primary	chr15	158	20	84116334	86312198	Del	0.75175	3.001491	q25.2-q25.3	2.195864
OVA_013_C2	Primary	chr15	155	20	84116397	86287910	Del	0.755564	3.14838	q25.2-q25.3	2.171513
OVA_013_C1	Primary	chr15	184	24	84324441	89018461	Del	0.701164	3.125184	q25.2-q25.3	4.69402
OVA_003_C1	Primary	chr15	94	4	84748364	85747580	Amp	0.852911	1.812048	q25.2-q25.3	0.999216
OVA_003_C3	Primary	chr15	90	4	84773565	85747557	Amp	0.820093	1.828085	q25.2-q25.3	0.973992
OVA_003_M3	Metastasis	chr15	85	4	84859008	85747557	Amp	0.574876	1.865189	q25.2-q25.3	0.888549
OVA_047_C2	Primary	chr6	250	16	153076420	1.59E+08	Del	0.575254	2.829045	q25.2-q25.3	6.388767
OVA_047_CO	Contralateral ovary	chr6	221	17	154412320	1.59E+08	Del	0.55613	2.930893	q25.2-q25.3	5.050901
OVA_047_C1	Primary	chr6	225	17	154415435	1.59E+08	Del	0.576016	2.862818	q25.2-q25.3	5.049752
OVA_378_M1	Metastasis	chr1	29	1	180151344	1.81E+08	Amp	0.753235	2.555202	q25.2-q25.3	0.652845
OVA_378_M2	Metastasis	chr1	31	1	180151516	1.81E+08	Amp	0.840932	2.531102	q25.2-q25.3	0.73375
OVA_378_C2	Primary	chr1	30	1	180153254	1.81E+08	Amp	0.607575	2.601752	q25.2-q25.3	0.732036
OVA_378_M3	Metastasis	chr1	28	1	180153254	1.81E+08	Amp	0.692693	2.569487	q25.2-q25.3	0.650935
OVA_378_C1	Primary	chr1	27	1	180155172	1.81E+08	Amp	0.616455	2.593914	q25.2-q25.3	0.649017
OVA_378_C3	Primary	chr1	27	1	180155172	1.81E+08	Amp	0.517221	2.617793	q25.2-q25.3	0.649017
OVA_003_M2	Metastasis	chr15	766	50	82444663	93616284	Gain	0.439877	1.867794	q25.2-q26.1	11.17162
OVA_047_CO	Contralateral ovary	chr10	776	56	112660970	1.28E+08	Loss	0.55613	2.930893	q25.2-q26.2	15.09906
OVA_013_C2	Primary	chr3	1078	73	154832793	1.87E+08	Del	0.755564	3.14838	q25.2-q27.3	32.25339
OVA_013_C1	Primary	chr3	1101	76	154802839	1.89E+08	Del	0.701164	3.125184	q25.2-q28	33.78137
OVA_013_C3	Primary	chr3	999	72	156979089	1.87E+08	Del	0.75175	3.001491	q25.31-q27.3	30.10731

Sample	Tissue type	chrom	num.mark	nhet	start	end	Classificati	Purity	Ploidy	Cyto	Size_Mb
OVA_013_M1	Metastasis	chr3	938	68	156979089	1.86E+08	Loss	0.621947	2.883383	q25.31-q27.3	29.3543
OVA_048_C1	Primary	chr3	1194	107	160119821	1.98E+08	Gain	0.682521	1.843999	q25.33-q29	37.68424
OVA_003_C1	Primary	chr17	1050	109	75493387	81083522	Gain	0.852911	1.812048	q25.3-q25.3	5.590135
OVA_003_C2	Primary	chr17	1026	106	76100687	81083526	Del	0.709607	3.665802	q25.3-q25.3	4.982839
OVA_003_M3	Metastasis	chr15	559	40	86063810	93595736	Gain	0.574876	1.865189	q25.3-q26.1	7.531926
OVA_013_C2	Primary	chr15	146	17	86311593	90171840	Loss	0.755564	3.14838	q25.3-q26.1	3.860247
OVA_013_C3	Primary	chr15	144	17	86312714	90171840	Loss	0.75175	3.001491	q25.3-q26.1	3.859126
OVA_013_C1	Primary	chr15	113	12	89020302	90169331	Loss	0.701164	3.125184	q25.3-q26.1	1.149029
OVA_003_C1	Primary	chr15	751	58	86063810	1.01E+08	Gain	0.852911	1.812048	q25.3-q26.3	15.05719
OVA_003_C2	Primary	chr15	758	59	86063810	1.01E+08	Del	0.709607	3.665802	q25.3-q26.3	15.36391
OVA_003_C3	Primary	chr15	751	58	86063810	1.01E+08	Gain	0.820093	1.828085	q25.3-q26.3	15.05719
OVA_003_C2	Primary	chr10	965	108	116697960	1.36E+08	Del	0.709607	3.665802	q25.3-q26.3	18.8028
OVA_003_C2	Primary	chr6	702	105	157431475	1.71E+08	Del	0.709607	3.665802	q25.3-q27	13.46198
OVA_047_CO	Contralateral ovary	chr6	526	65	159465187	1.71E+08	Del	0.55613	2.930893	q25.3-q27	11.42827
OVA_047_C1	Primary	chr6	527	66	159618397	1.71E+08	Del	0.576016	2.862818	q25.3-q27	11.2751
OVA_047_C2	Primary	chr6	494	59	159618413	1.71E+08	Del	0.575254	2.829045	q25.3-q27	10.97629
OVA_047_CO	Contralateral ovary	chr1	1335	103	180601485	2.05E+08	Loss	0.55613	2.930893	q25.3-q32.1	24.43191
OVA_047_M2	Metastasis	chr11	28	7	134177110	1.35E+08	Loss	0.599725	2.678568	q25-q25	0.679551
OVA_047_M3	Metastasis	chr11	23	6	134202036	1.35E+08	Loss	0.605088	2.815208	q25-q25	0.654625
OVA_013_C3	Primary	chr10	519	53	120095773	1.3E+08	Loss	0.75175	3.001491	q26.11-q26.2	9.82555
OVA_013_M1	Metastasis	chr10	519	53	120095773	1.3E+08	Loss	0.621947	2.883383	q26.11-q26.2	9.82555
OVA_013_C1	Primary	chr10	517	53	120353632	1.3E+08	Del	0.701164	3.125184	q26.11-q26.2	9.563928
OVA_013_C2	Primary	chr10	739	74	120353843	1.35E+08	Del	0.755564	3.14838	q26.11-q26.3	15.13724
OVA_013_M1	Metastasis	chr15	308	25	90168628	93595771	Loss	0.621947	2.883383	q26.1-q26.1	3.427143
OVA_013_C1	Primary	chr15	570	47	90170237	1.02E+08	Loss	0.701164	3.125184	q26.1-q26.3	12.14271
OVA_013_C2	Primary	chr15	580	45	90172192	1.02E+08	Loss	0.755564	3.14838	q26.1-q26.3	12.24456
OVA_013_C3	Primary	chr15	581	46	90172192	1.02E+08	Loss	0.75175	3.001491	q26.1-q26.3	12.24456
OVA_013_M1	Metastasis	chr15	278	23	93609204	1.02E+08	Del	0.621947	2.883383	q26.1-q26.3	8.807552
OVA_003_M3	Metastasis	chr3	325	23	161147383	1.75E+08	Gain	0.574876	1.865189	q26.1-q26.31	13.78845
OVA_003_C1	Primary	chr3	320	23	164059123	1.75E+08	Amp	0.852911	1.812048	q26.1-q26.31	10.87671
OVA_003_C2	Primary	chr3	320	23	164059123	1.75E+08	Loss	0.709607	3.665802	q26.1-q26.31	10.87671
OVA_003_C3	Primary	chr3	320	23	164059123	1.75E+08	Amp	0.820093	1.828085	q26.1-q26.31	10.87671
OVA_003_M1	Metastasis	chr3	321	23	164059123	1.75E+08	Gain	0.552447	1.852543	q26.1-q26.31	10.89279
OVA_048_M1	Metastasis	chr3	367	28	160783255	1.83E+08	Loss	0.625824	3.962486	q26.1-q26.33	21.75479
OVA_048_M2	Metastasis	chr3	381	28	160942908	1.83E+08	Loss	0.405007	3.72502	q26.1-q27.1	21.89908
OVA_048_C3	Primary	chr3	1170	107	160945145	1.98E+08	Amp	0.715645	1.786574	q26.1-q29	36.85898
OVA_048_C2	Primary	chr3	1166	107	161090056	1.98E+08	Gain	0.736255	1.845483	q26.1-q29	36.714
OVA_003_M2	Metastasis	chr3	1485	135	161147383	1.98E+08	Gain	0.439877	1.867794	q26.1-q29	36.70018
OVA_047_C2	Primary	chr10	413	51	127727943	1.36E+08	Del	0.575254	2.829045	q26.2-q26.3	7.772817
OVA_047_CO	Contralateral ovary	chr10	401	50	127786969	1.36E+08	Del	0.55613	2.930893	q26.2-q26.3	7.713791
OVA_048_M2	Metastasis	chr10	277	34	127967524	1.36E+08	Del	0.405007	3.72502	q26.2-q26.3	7.533236
OVA_013_C1	Primary	chr10	224	21	129921323	1.35E+08	Del	0.701164	3.125184	q26.2-q26.3	5.569629
OVA_013_C3	Primary	chr10	223	21	129923847	1.35E+08	Del	0.75175	3.001491	q26.2-q26.3	5.567105
OVA_013_M1	Metastasis	chr10	223	21	129923847	1.35E+08	Del	0.621947	2.883383	q26.2-q26.3	5.567232
OVA_003_C1	Primary	chr3	610	50	174951912	1.87E+08	Amp	0.852911	1.812048	q26.31-q27.3	12.0019
OVA_003_C3	Primary	chr3	695	61	174951912	1.91E+08	Amp	0.820093	1.828085	q26.31-q28	15.98467
OVA_003_M3	Metastasis	chr3	682	57	174951912	1.9E+08	Amp	0.574876	1.865189	q26.31-q28	15.37498
OVA_003_M1	Metastasis	chr3	681	57	175165069	1.9E+08	Amp	0.552447	1.852543	q26.31-q28	15.16182
OVA_048_M3	Metastasis	chr3	436	40	182583365	1.91E+08	Gain	0.59385	3.387584	q26.33-q28	8.353212
OVA_047_CO	Contralateral ovary	chr3	986	92	180667161	1.98E+08	Loss	0.55613	2.930893	q26.33-q29	17.1804
OVA_365_M1	Metastasis	chr15	256	31	98504100	1.03E+08	Amp	0.854003	1.907418	q26.3-q26.3	4.01229
OVA_365_C2	Primary	chr15	251	29	98984375	1.03E+08	Gain	0.629033	1.960121	q26.3-q26.3	3.532015
OVA_365_M3	Metastasis	chr15	251	29	98984375	1.03E+08	Amp	0.827915	1.890476	q26.3-q26.3	3.532015
OVA_365_CO	Contralateral ovary	chr15	194	21	99478732	1.02E+08	Amp	0.451025	1.890627	q26.3-q26.3	2.785744
OVA_047_C1	Primary	chr15	144	3	101109794	1.03E+08	Del	0.576016	2.862818	q26.3-q26.3	1.406465
OVA_003_C2	Primary	chr15	131	11	101432404	1.02E+08	Del	0.709607	3.665802	q26.3-q26.3	1.063609
OVA_047_CO	Contralateral ovary	chr15	35	0	102291723	1.03E+08	Del	0.55613	2.930893	q26.3-q26.3	0.224535
OVA_047_C2	Primary	chr15	35	0	102291955	1.03E+08	Loss	0.575254	2.829045	q26.3-q26.3	0.224435
OVA_013_C1	Primary	chr15	13	0	102313270	1.02E+08	Del	0.701164	3.125184	q26.3-q26.3	0.103486
OVA_048_M1	Metastasis	chr10	169	19	133918420	1.36E+08	Del	0.625824	3.962486	q26.3-q26.3	1.58234
OVA_048_M3	Metastasis	chr10	162	19	133967385	1.36E+08	Del	0.59385	3.387584	q26.3-q26.3	1.533375
OVA_047_M2	Metastasis	chr4	1836	175	114302870	1.91E+08	Del	0.599725	2.678568	q26-q35.2	76.64461
OVA_047_M3	Metastasis	chr4	1825	175	114429407	1.91E+08	Del	0.605088	2.815208	q26-q35.2	76.51807
OVA_047_M1	Metastasis	chr4	1823	175	114680390	1.91E+08	Del	0.624347	2.86771	q26-q35.2	76.26709
OVA_365_CO	Contralateral ovary	chr3	140	19	183952049	1.85E+08	Gain	0.451025	1.890627	q27.1-q27.2	0.608913
OVA_047_M3	Metastasis	chr3	316	30	183755964	1.86E+08	Loss	0.605088	2.815208	q27.1-q27.3	2.703811

Sample	Tissue type	chrom	num.mark	nhet	start	end	Classificati	Purity	Ploidy	Cyto	Size_Mb
OVA_047_M1	Metastasis	chr3	893	89	183755964	1.98E+08	Loss	0.624347	2.86771	q27.1-q29	14.0916
OVA_047_M2	Metastasis	chr3	41	1	186015199	1.86E+08	Loss	0.599725	2.678568	q27.3-q27.3	0.446354
OVA_047_M3	Metastasis	chr3	572	58	186461553	1.98E+08	Del	0.605088	2.815208	q27.3-q29	11.38601
OVA_047_M2	Metastasis	chr3	575	59	186501502	1.98E+08	Loss	0.599725	2.678568	q27.3-q29	11.34606
OVA_003_C1	Primary	chr3	550	62	186954273	1.98E+08	Amp	0.852911	1.812048	q27.3-q29	10.89329
OVA_047_C2	Primary	chr6	35	7	170595317	1.71E+08	Del	0.575254	2.829045	q27-q27	0.298179
OVA_003_M1	Metastasis	chr3	478	55	190338265	1.98E+08	Gain	0.552447	1.852543	q28-q29	7.509298
OVA_003_M3	Metastasis	chr3	478	55	190338265	1.98E+08	Gain	0.574876	1.865189	q28-q29	7.509298
OVA_003_C3	Primary	chr3	465	51	190967888	1.98E+08	Amp	0.820093	1.828085	q28-q29	6.879675
OVA_048_M2	Metastasis	chr3	198	22	190967888	1.96E+08	Loss	0.405007	3.72502	q28-q29	4.645903
OVA_048_M1	Metastasis	chr3	176	19	193002772	1.96E+08	Del	0.625824	3.962486	q29-q29	2.611019
OVA_003_C2	Primary	chr3	382	44	193175281	1.98E+08	Loss	0.709607	3.665802	q29-q29	4.333403
OVA_048_M3	Metastasis	chr3	337	36	193311212	1.98E+08	Loss	0.59385	3.387584	q29-q29	4.492846
OVA_048_M1	Metastasis	chr3	174	17	195622236	1.98E+08	Del	0.625824	3.962486	q29-q29	2.181892
OVA_048_M2	Metastasis	chr3	174	17	195622236	1.98E+08	Del	0.405007	3.72502	q29-q29	2.181892
OVA_013_C2	Primary	chr3	38	1	197444809	1.98E+08	Loss	0.755564	3.14838	q29-q29	0.402754
OVA_003_C2	Primary	chr3	35	1	197518319	1.98E+08	Loss	0.709607	3.665802	q29-q29	0.329244
OVA_013_C1	Primary	chr4	241	11	140255428	1.47E+08	Del	0.701164	3.125184	q31.1-q31.22	7.182783
OVA_013_M1	Metastasis	chr4	240	11	140255428	1.47E+08	Loss	0.621947	2.883383	q31.1-q31.22	7.182783
OVA_013_C2	Primary	chr4	221	11	140599767	1.47E+08	Del	0.755564	3.14838	q31.1-q31.22	6.838444
OVA_013_C3	Primary	chr4	221	11	140599767	1.47E+08	Del	0.75175	3.001491	q31.1-q31.22	6.836524
OVA_003_C2	Primary	chr13	330	34	82265080	1.02E+08	Del	0.709607	3.665802	q31.1-q33.1	19.95511
OVA_047_M1	Metastasis	chr2	1509	115	173792821	2.04E+08	Loss	0.624347	2.86771	q31.1-q33.2	30.51989
OVA_378_M1	Metastasis	chr5	3418	317	131278267	1.81E+08	Loss	0.753235	2.555202	q31.1-q35.3	49.40895
OVA_378_M3	Metastasis	chr5	3123	292	134696174	1.81E+08	Loss	0.692693	2.569487	q31.1-q35.3	45.99104
OVA_013_C3	Primary	chr2	3513	265	175111529	2.42E+08	Del	0.75175	3.001491	q31.1-q37.3	67.08121
OVA_013_C1	Primary	chr2	3510	265	175202307	2.42E+08	Del	0.701164	3.125184	q31.1-q37.3	66.99044
OVA_013_M1	Metastasis	chr2	3507	265	175202307	2.42E+08	Loss	0.621947	2.883383	q31.1-q37.3	66.99044
OVA_013_C2	Primary	chr2	3480	259	175335360	2.42E+08	Del	0.755564	3.14838	q31.1-q37.3	66.85738
OVA_013_C3	Primary	chr4	97	3	147438211	1.52E+08	Del	0.75175	3.001491	q31.22-q31.3	4.404035
OVA_013_M1	Metastasis	chr4	160	4	147442694	1.54E+08	Del	0.621947	2.883383	q31.22-q31.3	6.534347
OVA_013_C1	Primary	chr4	158	4	147442852	1.54E+08	Del	0.701164	3.125184	q31.22-q31.3	6.454586
OVA_013_C2	Primary	chr4	158	4	147442852	1.54E+08	Del	0.755564	3.14838	q31.22-q31.3	6.454586
OVA_047_C1	Primary	chr7	2296	241	116199050	1.59E+08	Loss	0.576016	2.862818	q31.2-q36.3	42.82613
OVA_047_M1	Metastasis	chr5	271	67	140080532	1.41E+08	Del	0.624347	2.86771	q31.3-q31.3	0.661587
OVA_047_M2	Metastasis	chr5	321	67	140080532	1.41E+08	Loss	0.599725	2.678568	q31.3-q31.3	0.704411
OVA_013_C3	Primary	chr5	311	66	140085281	1.41E+08	Del	0.75175	3.001491	q31.3-q31.3	0.72713
OVA_047_C1	Primary	chr5	321	67	140085281	1.41E+08	Loss	0.576016	2.862818	q31.3-q31.3	0.702569
OVA_048_M1	Metastasis	chr5	287	51	140085281	1.41E+08	Del	0.625824	3.962486	q31.3-q31.3	0.726063
OVA_048_M2	Metastasis	chr5	282	51	140165826	1.41E+08	Del	0.405007	3.72502	q31.3-q31.3	0.641903
OVA_048_M3	Metastasis	chr5	254	45	140165826	1.41E+08	Del	0.59385	3.387584	q31.3-q31.3	0.618488
OVA_013_C1	Primary	chr5	310	66	140166271	1.41E+08	Del	0.701164	3.125184	q31.3-q31.3	0.64614
OVA_047_C2	Primary	chr5	287	67	140166953	1.41E+08	Loss	0.575254	2.829045	q31.3-q31.3	0.588847
OVA_003_C3	Primary	chr5	348	18	140181734	1.41E+08	Gain	0.820093	1.828085	q31.3-q31.3	0.63087
OVA_003_M3	Metastasis	chr5	348	18	140181734	1.41E+08	Gain	0.574876	1.865189	q31.3-q31.3	0.630866
OVA_378_M2	Metastasis	chr5	484	77	140182101	1.41E+08	Loss	0.840932	2.531102	q31.3-q31.3	1.15262
OVA_013_M1	Metastasis	chr5	22	4	140306559	1.4E+08	Loss	0.621947	2.883383	q31.3-q31.3	0.1262
OVA_013_C3	Primary	chr4	61	1	151935743	1.54E+08	Del	0.75175	3.001491	q31.3-q31.3	1.961695
OVA_048_M2	Metastasis	chr5	596	64	140810541	1.51E+08	Del	0.405007	3.72502	q31.3-q33.1	10.41974
OVA_048_M1	Metastasis	chr5	593	64	140811652	1.51E+08	Del	0.625824	3.962486	q31.3-q33.1	10.41863
OVA_047_CO	Contralateral ovary	chr13	849	103	92536982	1.15E+08	Del	0.55613	2.930893	q31.3-q34	22.55474
OVA_047_C1	Primary	chr13	858	105	92537018	1.15E+08	Del	0.576016	2.862818	q31.3-q34	22.55471
OVA_047_C2	Primary	chr13	853	102	92797112	1.15E+08	Del	0.575254	2.829045	q31.3-q34	22.29461
OVA_013_C1	Primary	chr4	822	69	153977041	1.91E+08	Del	0.701164	3.125184	q31.3-q35.2	36.97041
OVA_013_C2	Primary	chr4	778	65	153977041	1.91E+08	Del	0.755564	3.14838	q31.3-q35.2	36.97041
OVA_013_C3	Primary	chr4	794	64	154091210	1.91E+08	Del	0.75175	3.001491	q31.3-q35.2	36.85624
OVA_013_M1	Metastasis	chr4	846	68	154091210	1.91E+08	Del	0.621947	2.883383	q31.3-q35.2	36.85624
OVA_013_M1	Metastasis	chr5	1811	180	140474756	1.78E+08	Del	0.621947	2.883383	q31.3-q35.3	37.25923
OVA_047_M1	Metastasis	chr5	2201	214	140744300	1.81E+08	Del	0.624347	2.86771	q31.3-q35.3	39.94291
OVA_047_C2	Primary	chr5	2186	214	140762493	1.81E+08	Del	0.575254	2.829045	q31.3-q35.3	39.92472
OVA_048_M3	Metastasis	chr5	1441	139	140784791	1.79E+08	Del	0.59385	3.387584	q31.3-q35.3	38.16725
OVA_047_M2	Metastasis	chr5	2158	213	140787968	1.81E+08	Del	0.599725	2.678568	q31.3-q35.3	39.89924
OVA_047_C1	Primary	chr5	2153	214	140788264	1.81E+08	Del	0.576016	2.862818	q31.3-q35.3	39.89895
OVA_013_C1	Primary	chr5	1616	140	140821366	1.78E+08	Del	0.701164	3.125184	q31.3-q35.3	36.91259
OVA_013_C3	Primary	chr5	1613	141	140821366	1.78E+08	Del	0.75175	3.001491	q31.3-q35.3	36.89592
OVA_378_M2	Metastasis	chr5	2216	175	141334921	1.81E+08	Loss	0.840932	2.531102	q31.3-q35.3	39.35229

Sample	Tissue type	chrom	num.mark	nhet	start	end	Classificati	Purity	Ploidy	Cyto	Size_Mb
OVA_003_C3	Primary	chr2	67	2	185243509	1.87E+08	Amp	0.820093	1.828085	q32.1-q32.1	1.449402
OVA_003_C1	Primary	chr2	10	1	185243700	1.86E+08	Amp	0.852911	1.812048	q32.1-q32.1	0.559664
OVA_003_C2	Primary	chr2	67	2	185243700	1.87E+08	Amp	0.709607	3.665802	q32.1-q32.1	1.449211
OVA_003_C1	Primary	chr2	57	1	186412247	1.87E+08	Amp	0.852911	1.812048	q32.1-q32.1	0.280664
OVA_048_C1	Primary	chr2	291	24	183832151	1.93E+08	Gain	0.682521	1.843999	q32.1-q32.3	9.030751
OVA_048_M1	Metastasis	chr2	577	41	186689190	2.03E+08	Del	0.625824	3.962486	q32.1-q33.1	16.47294
OVA_003_M1	Metastasis	chr2	1293	101	184125964	2.13E+08	Amp	0.552447	1.852543	q32.1-q34	28.86359
OVA_003_M3	Metastasis	chr2	1283	101	184472177	2.12E+08	Amp	0.574876	1.865189	q32.1-q34	27.81451
OVA_003_C1	Primary	chr2	1224	99	187353022	2.13E+08	Amp	0.852911	1.812048	q32.1-q34	25.63654
OVA_003_C2	Primary	chr2	1189	96	187353022	2.11E+08	Loss	0.709607	3.665802	q32.1-q34	23.98945
OVA_003_C3	Primary	chr2	1225	99	187353022	2.13E+08	Amp	0.820093	1.828085	q32.1-q34	26.05033
OVA_003_M2	Metastasis	chr2	1319	103	184023042	2.16E+08	Gain	0.439877	1.867794	q32.1-q35	31.5726
OVA_378_C3	Primary	chr2	1860	118	188348899	2.21E+08	Loss	0.517221	2.617793	q32.1-q35	32.15462
OVA_047_CO	Contralateral ovary	chr7	1679	183	127235865	1.51E+08	Loss	0.55613	2.930893	q32.1-q36.1	23.85744
OVA_378_C2	Primary	chr2	1865	120	188348943	2.22E+08	Loss	0.607575	2.601752	q32.1-q36.1	33.94196
OVA_047_C2	Primary	chr7	1992	218	127231185	1.59E+08	Loss	0.575254	2.829045	q32.1-q36.3	31.794
OVA_048_M2	Metastasis	chr2	2506	219	187353022	2.42E+08	Del	0.405007	3.72502	q32.1-q37.3	54.83986
OVA_047_C1	Primary	chr1	720	72	205035648	2.19E+08	Del	0.576016	2.862818	q32.1-q41	14.34832
OVA_047_M3	Metastasis	chr1	2382	247	201437832	2.38E+08	Loss	0.605088	2.815208	q32.1-q43	36.61131
OVA_047_C2	Primary	chr1	2377	266	205035648	2.49E+08	Del	0.575254	2.829045	q32.1-q44	44.19527
OVA_047_CO	Contralateral ovary	chr1	2362	264	205035648	2.49E+08	Del	0.55613	2.930893	q32.1-q44	44.19526
OVA_003_M2	Metastasis	chr14	990	124	96875317	1.07E+08	Gain	0.439877	1.867794	q32.2-q32.33	10.40781
OVA_003_C1	Primary	chr14	753	82	96887282	1.06E+08	Amp	0.852911	1.812048	q32.2-q32.33	8.851824
OVA_003_C2	Primary	chr14	988	124	96909175	1.07E+08	Loss	0.709607	3.665802	q32.2-q32.33	10.37393
OVA_003_C3	Primary	chr14	876	96	96909175	1.06E+08	Amp	0.820093	1.828085	q32.2-q32.33	9.473522
OVA_003_M3	Metastasis	chr14	988	124	96909175	1.07E+08	Gain	0.574876	1.865189	q32.2-q32.33	10.3739
OVA_047_M1	Metastasis	chr14	937	112	97322892	1.07E+08	Loss	0.624347	2.86771	q32.2-q32.33	9.96023
OVA_003_M1	Metastasis	chr14	905	121	100357446	1.07E+08	Gain	0.552447	1.852543	q32.2-q32.33	6.925659
OVA_048_M3	Metastasis	chr2	524	35	189842822	2.03E+08	Del	0.59385	3.387584	q32.2-q33.1	13.3252
OVA_048_M1	Metastasis	chr14	276	58	104394879	1.07E+08	Del	0.625824	3.962486	q32.33-q32.33	2.123969
OVA_013_C1	Primary	chr14	376	53	104407243	1.07E+08	Loss	0.701164	3.125184	q32.33-q32.33	2.875913
OVA_013_C3	Primary	chr14	376	52	104407243	1.07E+08	Loss	0.75175	3.001491	q32.33-q32.33	2.875913
OVA_048_M2	Metastasis	chr14	274	58	104407754	1.07E+08	Loss	0.405007	3.72502	q32.33-q32.33	2.111094
OVA_048_M3	Metastasis	chr14	270	58	104473035	1.07E+08	Loss	0.59385	3.387584	q32.33-q32.33	2.045813
OVA_047_C1	Primary	chr14	469	88	104491990	1.07E+08	Loss	0.576016	2.862818	q32.33-q32.33	2.791132
OVA_047_C2	Primary	chr14	464	87	104518625	1.07E+08	Loss	0.575254	2.829045	q32.33-q32.33	2.764451
OVA_047_CO	Contralateral ovary	chr14	464	87	104518625	1.07E+08	Del	0.55613	2.930893	q32.33-q32.33	2.764451
OVA_013_C2	Primary	chr14	334	47	105167807	1.07E+08	Loss	0.755564	3.14838	q32.33-q32.33	2.115349
OVA_048_C3	Primary	chr14	203	49	105344761	1.07E+08	Amp	0.715645	1.786574	q32.33-q32.33	1.174087
OVA_003_C1	Primary	chr14	236	42	105767293	1.07E+08	Gain	0.852911	1.812048	q32.33-q32.33	1.515812
OVA_013_M1	Metastasis	chr14	195	36	105821457	1.07E+08	Loss	0.621947	2.883383	q32.33-q32.33	1.461699
OVA_003_C3	Primary	chr14	112	28	106385332	1.07E+08	Gain	0.820093	1.828085	q32.33-q32.33	0.897773
OVA_048_M3	Metastasis	chr14	72	16	106539306	1.07E+08	Del	0.59385	3.387584	q32.33-q32.33	0.74385
OVA_048_M1	Metastasis	chr14	71	15	106539361	1.07E+08	Del	0.625824	3.962486	q32.33-q32.33	0.743761
OVA_048_M2	Metastasis	chr14	72	16	106539361	1.07E+08	Del	0.405007	3.72502	q32.33-q32.33	0.743715
OVA_365_M3	Metastasis	chr13	648	58	99549915	1.15E+08	Loss	0.827915	1.890476	q32.3-q34	15.54148
OVA_365_M1	Metastasis	chr13	624	55	99853225	1.15E+08	Loss	0.854003	1.907418	q32.3-q34	15.23817
OVA_365_C2	Primary	chr13	644	58	99853332	1.15E+08	Loss	0.629033	1.960121	q32.3-q34	15.23807
OVA_048_C2	Primary	chr1	1464	180	214491536	2.49E+08	Gain	0.736255	1.845483	q32.3-q44	34.72037
OVA_003_C1	Primary	chr5	1675	137	149301194	1.81E+08	Loss	0.852911	1.812048	q32-q35.3	31.38602
OVA_003_C3	Primary	chr5	1682	137	149301213	1.81E+08	Loss	0.820093	1.828085	q32-q35.3	31.386
OVA_003_C2	Primary	chr5	1665	135	149301223	1.81E+08	Del	0.709607	3.665802	q32-q35.3	31.38599
OVA_048_C2	Primary	chr2	78	8	203168024	2.06E+08	Loss	0.736255	1.845483	q33.1-q33.3	2.382991
OVA_048_M1	Metastasis	chr2	77	8	203168024	2.06E+08	Del	0.625824	3.962486	q33.1-q33.3	2.382991
OVA_048_M3	Metastasis	chr2	77	8	203242264	2.06E+08	Del	0.59385	3.387584	q33.1-q33.3	2.308751
OVA_003_C2	Primary	chr13	518	64	102227872	1.15E+08	Del	0.709607	3.665802	q33.1-q34	12.27595
OVA_048_M1	Metastasis	chr5	978	95	151231140	1.81E+08	Del	0.625824	3.962486	q33.1-q35.3	29.39937
OVA_048_M2	Metastasis	chr5	1002	97	151231140	1.81E+08	Del	0.405007	3.72502	q33.1-q35.3	29.45607
OVA_048_C1	Primary	chr9	107	10	124882285	1.27E+08	Amp	0.682521	1.843999	q33.2-q33.3	2.15232
OVA_048_C2	Primary	chr9	113	9	124895292	1.27E+08	Amp	0.736255	1.845483	q33.2-q33.3	2.223719
OVA_048_C3	Primary	chr9	97	9	124931954	1.27E+08	Amp	0.715645	1.786574	q33.2-q33.3	1.845507
OVA_013_C1	Primary	chr9	1083	76	124044059	1.35E+08	Del	0.701164	3.125184	q33.2-q34.13	10.91081
OVA_013_C2	Primary	chr9	1136	81	124044059	1.36E+08	Del	0.755564	3.14838	q33.2-q34.13	11.5022
OVA_013_C3	Primary	chr9	1083	75	124044059	1.35E+08	Del	0.75175	3.001491	q33.2-q34.13	10.99829
OVA_013_M1	Metastasis	chr9	1082	76	124044059	1.35E+08	Del	0.621947	2.883383	q33.2-q34.13	10.99829
OVA_047_M2	Metastasis	chr9	1122	76	124967119	1.34E+08	Loss	0.599725	2.678568	q33.2-q34.13	9.530299

Sample	Tissue type	chrom	num.mark	nhet	start	end	Classificati	Purity	Ploidy	Cyto	Size_Mb
OVA_365_CO	Contralateral ovary	chr9	2585	229	123151547	1.41E+08	Gain	0.451025	1.890627	q33.2-q34.3	17.75601
OVA_047_M1	Metastasis	chr2	448	47	204322277	2.17E+08	Del	0.624347	2.86771	q33.2-q35	12.62415
OVA_047_M2	Metastasis	chr2	475	53	204322298	2.17E+08	Del	0.599725	2.678568	q33.2-q35	12.74685
OVA_047_M3	Metastasis	chr2	445	49	204322387	2.17E+08	Del	0.605088	2.815208	q33.2-q35	12.62404
OVA_047_C1	Primary	chr2	1806	211	204322298	2.35E+08	Loss	0.576016	2.862818	q33.2-q37.1	30.56965
OVA_047_CO	Contralateral ovary	chr2	1798	211	204354385	2.35E+08	Loss	0.55613	2.930893	q33.2-q37.1	30.51896
OVA_047_C2	Primary	chr9	1116	93	129246177	1.36E+08	Loss	0.575254	2.829045	q33.3-q34.2	7.163554
OVA_047_C1	Primary	chr9	1988	95	129453229	1.41E+08	Loss	0.576016	2.862818	q33.3-q34.3	11.21932
OVA_047_CO	Contralateral ovary	chr9	2016	95	129453229	1.41E+08	Loss	0.55613	2.930893	q33.3-q34.3	11.48503
OVA_048_M1	Metastasis	chr2	1854	170	205969220	2.42E+08	Del	0.625824	3.962486	q33.3-q37.3	36.22352
OVA_048_M3	Metastasis	chr2	1854	170	205969220	2.42E+08	Del	0.59385	3.387584	q33.3-q37.3	36.22366
OVA_003_C1	Primary	chr9	101	5	133779448	1.34E+08	Amp	0.852911	1.812048	q34.12-q34.13	0.591717
OVA_003_C3	Primary	chr9	100	5	133779448	1.34E+08	Amp	0.820093	1.828085	q34.12-q34.13	0.58735
OVA_003_M2	Metastasis	chr9	1325	108	133779448	1.41E+08	Gain	0.439877	1.867794	q34.12-q34.3	7.292262
OVA_003_M1	Metastasis	chr9	1325	108	133779557	1.41E+08	Gain	0.552447	1.852543	q34.12-q34.3	7.292153
OVA_003_M3	Metastasis	chr9	1325	108	133779557	1.41E+08	Gain	0.574876	1.865189	q34.12-q34.3	7.292153
OVA_013_C1	Primary	chr9	274	32	135042352	1.37E+08	Loss	0.701164	3.125184	q34.13-q34.2	2.278648
OVA_003_C2	Primary	chr9	475	43	134385582	1.38E+08	Del	0.709607	3.665802	q34.13-q34.3	3.850427
OVA_047_M2	Metastasis	chr9	1182	32	134501419	1.41E+08	Loss	0.599725	2.678568	q34.13-q34.3	6.570291
OVA_047_M1	Metastasis	chr9	1156	32	134585058	1.41E+08	Del	0.624347	2.86771	q34.13-q34.3	6.353205
OVA_013_C3	Primary	chr9	943	90	135073877	1.41E+08	Loss	0.75175	3.001491	q34.13-q34.3	5.667114
OVA_013_M1	Metastasis	chr9	374	53	135073967	1.38E+08	Loss	0.621947	2.883383	q34.13-q34.3	3.317738
OVA_013_C2	Primary	chr9	909	88	135553416	1.41E+08	Loss	0.755564	3.14838	q34.13-q34.3	5.518294
OVA_047_C2	Primary	chr9	918	3	136412236	1.41E+08	Loss	0.575254	2.829045	q34.2-q34.3	4.659474
OVA_013_C1	Primary	chr9	60	11	137323917	1.38E+08	Del	0.701164	3.125184	q34.2-q34.3	0.41002
OVA_013_C1	Primary	chr9	632	50	137740912	1.41E+08	Loss	0.701164	3.125184	q34.3-q34.3	3.330798
OVA_048_M2	Metastasis	chr9	577	64	137801416	1.41E+08	Del	0.405007	3.72502	q34.3-q34.3	3.270123
OVA_003_C2	Primary	chr9	745	60	138363577	1.41E+08	Loss	0.709607	3.665802	q34.3-q34.3	2.708133
OVA_003_C3	Primary	chr9	745	60	138363577	1.41E+08	Amp	0.820093	1.828085	q34.3-q34.3	2.708133
OVA_003_C1	Primary	chr9	737	59	138379404	1.41E+08	Amp	0.852911	1.812048	q34.3-q34.3	2.692306
OVA_048_M3	Metastasis	chr9	391	44	139370186	1.41E+08	Del	0.59385	3.387584	q34.3-q34.3	1.70131
OVA_047_M1	Metastasis	chr9	16	0	140943730	1.41E+08	Loss	0.624347	2.86771	q34.3-q34.3	0.12798
OVA_003_C2	Primary	chr13	65	4	114504761	1.15E+08	Del	0.709607	3.665802	q34-q34	0.586965
OVA_048_M1	Metastasis	chr7	166	35	141537735	1.43E+08	Del	0.625824	3.962486	q34-q34	1.381503
OVA_048_M2	Metastasis	chr7	476	76	141536243	1.5E+08	Del	0.405007	3.72502	q34-q36.1	8.954913
OVA_048_M3	Metastasis	chr7	823	114	141464765	1.59E+08	Del	0.59385	3.387584	q34-q36.3	17.06962
OVA_048_M1	Metastasis	chr7	686	80	142920057	1.59E+08	Del	0.625824	3.962486	q34-q36.3	16.10442
OVA_003_C2	Primary	chr2	1250	101	212483858	2.33E+08	Del	0.709607	3.665802	q34-q37.1	20.76721
OVA_003_C1	Primary	chr2	1246	104	213403353	2.33E+08	Gain	0.852911	1.812048	q34-q37.1	19.86957
OVA_003_M1	Metastasis	chr2	1245	104	213403353	2.33E+08	Gain	0.552447	1.852543	q34-q37.1	19.86957
OVA_003_C3	Primary	chr2	1241	102	213791128	2.33E+08	Gain	0.820093	1.828085	q34-q37.1	19.46024
OVA_365_M1	Metastasis	chr2	2009	203	212248673	2.42E+08	Loss	0.854003	1.907418	q34-q37.3	29.94421
OVA_365_M3	Metastasis	chr2	2084	217	212248673	2.42E+08	Loss	0.827915	1.890476	q34-q37.3	29.94421
OVA_003_M3	Metastasis	chr2	1697	132	212483858	2.39E+08	Gain	0.574876	1.865189	q34-q37.3	26.18885
OVA_048_M3	Metastasis	chr4	249	30	183721094	1.91E+08	Del	0.59385	3.387584	q35.1-q35.2	7.226387
OVA_048_M3	Metastasis	chr5	186	28	178996435	1.81E+08	Del	0.59385	3.387584	q35.3-q35.3	1.690777
OVA_048_M1	Metastasis	chr5	24	2	180649762	1.81E+08	Del	0.625824	3.962486	q35.3-q35.3	0.03745
OVA_003_M2	Metastasis	chr2	1656	126	215610598	2.39E+08	Gain	0.439877	1.867794	q35-q37.3	23.05431
OVA_047_M1	Metastasis	chr2	1879	215	216965002	2.42E+08	Del	0.624347	2.86771	q35-q37.3	25.22788
OVA_047_M3	Metastasis	chr2	1881	216	216965046	2.42E+08	Del	0.605088	2.815208	q35-q37.3	25.22784
OVA_047_M2	Metastasis	chr2	1861	213	217124227	2.42E+08	Del	0.599725	2.678568	q35-q37.3	25.06865
OVA_048_M2	Metastasis	chr7	346	38	150491889	1.59E+08	Del	0.405007	3.72502	q36.1-q36.3	8.044445
OVA_047_CO	Contralateral ovary	chr7	309	35	151097323	1.59E+08	Loss	0.55613	2.930893	q36.1-q36.3	7.927857
OVA_003_M1	Metastasis	chr7	306	27	151483445	1.59E+08	Gain	0.552447	1.852543	q36.1-q36.3	7.541883
OVA_003_M2	Metastasis	chr7	306	27	151483445	1.59E+08	Gain	0.439877	1.867794	q36.1-q36.3	7.541883
OVA_003_C3	Primary	chr7	300	25	151700036	1.59E+08	Amp	0.820093	1.828085	q36.1-q36.3	7.325292
OVA_003_C1	Primary	chr7	299	25	151704883	1.59E+08	Amp	0.852911	1.812048	q36.1-q36.3	7.320445
OVA_003_M3	Metastasis	chr7	243	25	151949735	1.59E+08	Amp	0.574876	1.865189	q36.1-q36.3	7.075593
OVA_013_C2	Primary	chr7	154	29	155301717	1.59E+08	Loss	0.755564	3.14838	q36.3-q36.3	3.72276
OVA_048_M3	Metastasis	chr7	33	2	158536345	1.59E+08	Del	0.59385	3.387584	q36.3-q36.3	0.488132
OVA_048_M2	Metastasis	chr7	32	1	158540880	1.59E+08	Del	0.405007	3.72502	q36.3-q36.3	0.483597
OVA_003_C2	Primary	chr2	338	25	233251366	2.37E+08	Loss	0.709607	3.665802	q37.1-q37.3	4.238747
OVA_003_C3	Primary	chr2	337	24	233271824	2.37E+08	Amp	0.820093	1.828085	q37.1-q37.3	4.218289
OVA_003_M1	Metastasis	chr2	844	85	233273219	2.42E+08	Gain	0.552447	1.852543	q37.1-q37.3	8.919662
OVA_003_C1	Primary	chr2	333	22	233273244	2.37E+08	Amp	0.852911	1.812048	q37.1-q37.3	4.21692
OVA_047_CO	Contralateral ovary	chr2	107	17	234878977	2.38E+08	Del	0.55613	2.930893	q37.1-q37.3	3.391401

Sample	Tissue type	chrom	num.mark	nhet	start	end	Classificati	Purity	Ploidy	Cyto	Size_Mb
OVA_047_C2	Primary	chr2	107	18	234891951	2.38E+08	Del	0.575254	2.829045	q37.1-q37.3	3.382619
OVA_047_C1	Primary	chr2	113	18	234894553	2.38E+08	Del	0.576016	2.862818	q37.1-q37.3	3.386408
OVA_003_C2	Primary	chr2	106	4	237994571	2.39E+08	Del	0.709607	3.665802	q37.3-q37.3	0.676599
OVA_047_CO	Contralateral ovary	chr2	438	39	238274359	2.42E+08	Loss	0.55613	2.930893	q37.3-q37.3	3.918522
OVA_047_C1	Primary	chr2	136	14	238283278	2.39E+08	Loss	0.576016	2.862818	q37.3-q37.3	0.901291
OVA_003_M2	Metastasis	chr2	284	43	238667311	2.42E+08	Gain	0.439877	1.867794	q37.3-q37.3	3.167633
OVA_003_C3	Primary	chr2	104	22	238668802	2.39E+08	Amp	0.820093	1.828085	q37.3-q37.3	0.684369
OVA_003_M3	Metastasis	chr2	98	18	238678650	2.39E+08	Amp	0.574876	1.865189	q37.3-q37.3	0.666013
OVA_003_C1	Primary	chr2	99	18	238725768	2.4E+08	Amp	0.852911	1.812048	q37.3-q37.3	1.031476
OVA_047_C2	Primary	chr2	205	25	239170938	2.42E+08	Gain	0.575254	2.829045	q37.3-q37.3	2.733843
OVA_003_M3	Metastasis	chr2	304	39	239353013	2.42E+08	Gain	0.574876	1.865189	q37.3-q37.3	2.83973
OVA_003_C2	Primary	chr2	275	38	239355184	2.42E+08	Del	0.709607	3.665802	q37.3-q37.3	2.766852
OVA_003_C3	Primary	chr2	303	39	239355184	2.42E+08	Gain	0.820093	1.828085	q37.3-q37.3	2.837697
OVA_003_C1	Primary	chr2	273	38	239974655	2.42E+08	Gain	0.852911	1.812048	q37.3-q37.3	2.147381
OVA_048_C1	Primary	chr2	50	7	242029657	2.42E+08	Gain	0.682521	1.843999	q37.3-q37.3	0.163224
OVA_003_C2	Primary	chr2	28	1	242122775	2.42E+08	Loss	0.709607	3.665802	q37.3-q37.3	0.069968
OVA_003_C1	Primary	chr2	28	1	242122845	2.42E+08	Amp	0.852911	1.812048	q37.3-q37.3	0.070036
OVA_048_M2	Metastasis	chr1	1454	180	214556810	2.49E+08	Loss	0.405007	3.72502	q41-q44	34.65522
OVA_048_M1	Metastasis	chr1	1443	180	214638172	2.49E+08	Loss	0.625824	3.962486	q41-q44	34.57373
OVA_047_C1	Primary	chr1	1644	193	219384945	2.49E+08	Del	0.576016	2.862818	q41-q44	29.7648
OVA_048_C1	Primary	chr1	1355	170	219706428	2.49E+08	Gain	0.682521	1.843999	q41-q44	29.50548
OVA_048_C3	Primary	chr1	1355	170	219706428	2.49E+08	Amp	0.715645	1.786574	q41-q44	29.5056
OVA_378_M1	Metastasis	chr1	33	2	242222534	2.44E+08	Amp	0.753235	2.555202	q43-q43	1.453314
OVA_378_M2	Metastasis	chr1	33	2	242222534	2.44E+08	Amp	0.840932	2.531102	q43-q43	1.453314
OVA_378_M3	Metastasis	chr1	33	2	242222534	2.44E+08	Amp	0.692693	2.569487	q43-q43	1.453314
OVA_378_M1	Metastasis	chr1	23	3	245582792	2.46E+08	Amp	0.753235	2.555202	q44-q44	0.439089
OVA_378_M2	Metastasis	chr1	22	3	245703990	2.46E+08	Amp	0.840932	2.531102	q44-q44	0.317861
OVA_003_C2	Primary	chr1	176	14	247419534	2.49E+08	Loss	0.709607	3.665802	q44-q44	1.803401
OVA_047_M1	Metastasis	chr1	16	2	249148163	2.49E+08	Loss	0.624347	2.86771	q44-q44	0.082751
OVA_047_C1	Primary	chr1	14	1	249150022	2.49E+08	Del	0.576016	2.862818	q44-q44	0.080892
OVA_047_M3	Metastasis	chr1	13	1	249150205	2.49E+08	Loss	0.605088	2.815208	q44-q44	0.080709
OVA_013_M1	Metastasis	chrX	11	0	154318875	1.55E+08	Loss	0.621947	2.883383		0.43538

Supplementary Table S4: Significant pathways (P < 0.01) of subclonal mutations using Ingenuity pathway analysis

S.N	Pathway	p-value	Genes
1	Role of BRCA1 in DNA Damage Response	1.99526E-06	BRIP1,FANCE,MSH6,ATR,MSH2
2	Colorectal Cancer Metastasis Signaling	6.30957E-05	STAT3,SRC,GNAS,MSH6,SMO,MSH2
3	Wnt/β-catenin Signaling	8.31764E-05	AXIN2,GNAQ,SRC,RARA,SMO
4	Role of JAK2 in Hormone-like Cytokine Signaling	9.12011E-05	STAT3,PTPN6,STAT5B
5	Tec Kinase Signaling	0.0001	GNAQ,STAT3,SRC,GNAS,STAT5B
6	JAK/Stat Signaling	0.000102329	GNAQ,STAT3,PTPN6,STAT5B
7	ATM Signaling	0.000114815	ATR,ZEB1,TRRAP,MDM4
8	Adipogenesis pathway	0.000398107	SMO,STAT5B,PPARG,SETDB1
9	Role of Tissue Factor in Cancer	0.00040738	GNAQ,SRC,ITGAV,STAT5B
10	STAT3 Pathway	0.00040738	STAT3,SRC,NTRK1,PTPN6
11	Aryl Hydrocarbon Receptor Signaling	0.000512861	SRC,RARA,ATR,ARNT
12	Human Embryonic Stem Cell Pluripotency	0.000562341	GNAS,SALL4,SMO,NTRK1
13	Protein Kinase A Signaling	0.000562341	GNAQ,GNAS,NFATC2,SMO,PTPRT,PTPN6
14	Molecular Mechanisms of Cancer	0.00057544	GNAQ,SRC,GNAS,CDKN2C,SMO,ATR
15	Hereditary Breast Cancer Signaling	0.000691831	FANCE,MSH6,ATR,MSH2
16	Ovarian Cancer Signaling	0.000691831	SRC,MSH6,SMO,MSH2
17	Mismatch Repair in Eukaryotes	0.000758578	MSH6,MSH2
18	ERK5 Signaling	0.000851138	GNAQ,SRC,NTRK1
19	Ephrin Receptor Signaling	0.001202264	GNAQ,STAT3,SRC,GNAS
20	Growth Hormone Signaling	0.00144544	STAT3,PTPN6,STAT5B
21	RAR Activation	0.001479108	REL,SRC,RARA,STAT5B
22	T Cell Exhaustion Signaling Pathway	0.001479108	STAT3,NFATC2,IRF4,PTPN6
23	Role of Macrophages, Fibroblasts and Endothelial Cells in Rheumatoid Arthritis	0.001548817	GNAQ,STAT3,SRC,NFATC2,SMO
24	Role of NFAT in Regulation of the Immune Response	0.001659587	GNAQ,XPO1,GNAS,NFATC2
25	Erythropoietin Signaling	0.001698244	SRC,PTPN6,STAT5B
26	Th17 Activation Pathway	0.001698244	STAT3,NFATC2,IRF4
27	IL-22 Signaling	0.001737801	STAT3,STAT5B
28	Role of JAK1, JAK2 and TYK2 in Interferon Signaling	0.001737801	STAT3,PTPN6
29	IL-3 Signaling	0.001778279	STAT3,PTPN6,STAT5B
30	Role of JAK family kinases in IL-6-type Cytokine Signaling	0.001905461	STAT3,STAT5B
31	Adrenomedullin signaling pathway	0.002089296	GNAQ,GNAS,ARNT,PPARG
32	Glucocorticoid Receptor Signaling	0.002089296	TAF15,STAT3,NFATC2,PBX1,STAT5B
33	IL-4 Signaling	0.002137962	NFATC2,IRF4,PTPN6
34	Acute Myeloid Leukemia Signaling	0.002398833	STAT3,RARA,STAT5B
35	Sperm Motility	0.002691535	SRC,GNAS,PTK6,NTRK1
36	Melanocyte Development and Pigmentation Signaling	0.002754229	SRC,GNAS,PTPN6
37	G Protein Signaling Mediated by Tubby	0.002884032	GNAQ,GNAS
38	VEGF Signaling	0.003162278	SRC,ARNT,PTPN6
39	IL-15 Production	0.003801894	SRC,PTK6,NTRK1
40	G Beta Gamma Signaling	0.003890451	GNAQ,SRC,GNAS
41	GÎ±i Signaling	0.004168694	STAT3,SRC,GNAS
42	Phospholipase C Signaling	0.004365158	GNAQ,SRC,GNAS,NFATC2
43	April Mediated Signaling	0.004570882	TNFRSF17,NFATC2
44	Renin-Angiotensin Signaling	0.004897788	GNAQ,STAT3,PTPN6
45	Role of NANOG in Mammalian Embryonic Stem Cell Pluripotency	0.004897788	STAT3,SALL4,SMO
46	B Cell Activating Factor Signaling	0.005011872	TNFRSF17,NFATC2
47	Androgen Signaling	0.005248075	GNAQ,SRC,GNAS
48	Estrogen Receptor Signaling	0.005370318	TAF15,SRC,TRRAP
49	Iron homeostasis signaling pathway	0.005370318	STAT3,ARNT,STAT5B
50	Oncostatin M Signaling	0.005495409	STAT3,STAT5B
51	Corticotropin Releasing Hormone Signaling	0.006025596	GNAQ,GNAS,SMO
52	G-Protein Coupled Receptor Signaling	0.006309573	GNAQ,STAT3,SRC,GNAS
53	Role of Oct4 in Mammalian Embryonic Stem Cell Pluripotency	0.006309573	RARA,SALL4
54	IL-9 Signaling	0.006606934	STAT3,STAT5B
55	Cell Cycle: G2/M DNA Damage Checkpoint Regulation	0.007079458	ATR,MDM4
56	Thyroid Cancer Signaling	0.007762471	NTRK1,PPARG
57	UVC-Induced MAPK Signaling	0.007943282	SRC,ATR
58	Cardiac Hypertrophy Signaling (Enhanced)	0.009120108	STAT3,GNAQ,GNAS,NFATC2,SMO
59	Axonal Guidance Signaling	0.009332543	GNAQ,GNAS,NFATC2,SMO,NTRK1

Supplementary Table S5: Metastatic specific mutations with VAF

##Abbreviations of column headers

##chr: Chromosome

##position: hg19 start coordinate

##Ref: Reference base

##Alt: Altered base

##gene: Hugo gene symbol

##pri_ref:Primary tissue, total read count of the reference base

##pri_alt:Primary tissue, total read count of the altered base

##pri_total:Primary tissue, total read count

##pri_vaf:Primary tissue, variant allele frequency

##met_ref:Metastatic tissue, total read count of the reference base

##met_alt:Metastatic tissue, total read count of the altered base

##met_total:Metastatic tissue, total read count

##met_vaf:Metastatic tissue, variant allele frequency

##p-value:P-value

Sample	Chr	Position	Ref	Alt	gene	pri_ref	pri_alt	pri_total	pri_vaf	met_ref	met_alt	met_total	met_vaf	p-value
OVA_003	chr11	69468115	C	T	AK294004	5634	3	5637	0.05322	4757	306	5063	6.043848	1.84E-143
OVA_003	chr20	41419958	G	C	PTPRT	2779	1	2780	0.035971	2380	131	2511	5.217045	7.94E-62
OVA_013	chr10	20290838	G	A	PLXDC2	6589	20	6609	0.302618	1150	339	1489	22.76696	0
OVA_013	chr11	102743610	T	A	MMP12	6402	1	6403	0.015618	1253	235	1488	15.79301	3.61E-266
OVA_013	chr1	113245652	C	G	RHOC	3280	0	3280	0	643	173	816	21.20098	3.20E-180
OVA_013	chr1	160922392	A	T	ITLN2	5647	3	5650	0.053097	1065	234	1299	18.01386	5.28E-272
OVA_013	chr11	66260244	G	C	DPP3	2502	0	2502	0	364	96	460	20.86957	2.73E-106
OVA_013	chr16	65025929	A	T	CDH11	3767	0	3767	0	628	275	903	30.45404	0
OVA_013	chr17	78401505	G	T	ENDOV	2669	0	2669	0	438	156	594	26.26263	1.24E-183
OVA_013	chr3	39452702	C	A	RPSA	6242	3	6245	0.048038	1231	422	1653	25.52934	0
OVA_013	chr4	42592858	A	C	ATP8A1	4840	0	4840	0	1053	262	1315	19.92395	4.52E-298
OVA_013	chr5	148424122	C	T	SH3TC2	2483	6	2489	0.241061	477	179	656	27.28659	4.86E-174
OVA_013	chr6	44197705	A	C	SLC29A1	4304	2	4306	0.046447	694	146	840	17.38095	7.54E-174
OVA_013	chr6	51892757	A	G	PKHD1	5067	0	5067	0	884	227	1111	20.43204	7.94E-263
OVA_013	chr6	75898197	A	G	COL12A1	7518	1	7519	0.0133	1464	454	1918	23.67049	0
OVA_013	chr7	139655316	G	A	TBXAS1	3850	3	3853	0.077861	738	170	908	18.72247	6.29E-176
OVA_013	chr8	95565611	A	G	KIAA1429	4756	812	5568	14.58333	1498	234	1732	13.51039	1
OVA_047	chr10	101611394	G	T	ABCC2	990	1	991	0.100908	672	313	985	31.77665	2.61E-140
OVA_047	chr10	105821154	A	G	COL17A1	1358	0	1358	0	754	248	1002	24.7505	5.81E-128
OVA_047	chr10	12139705	A	C	DHTKD1	5264	3	5267	0.056958	3142	1782	4924	36.19009	0
OVA_047	chr10	126150525	G	T	LHPP	176	0	176	0	130	31	161	19.25466	6.56E-13
OVA_047	chr10	18250713	T	A	SLC39A12	4587	1	4588	0.021796	2967	829	3796	21.83878	0
OVA_047	chr10	93756861	G	A	BTAF1	3552	0	3552	0	2830	292	3122	9.352979	1.63E-151
OVA_047	chr1	110456855	G	A	CSF1	3488	3	3491	0.085935	4360	69	4429	1.557914	7.76E-19
OVA_047	chr11	108263943	G	A	C11orf65	6357	6	6363	0.094295	2742	1271	4013	31.67207	0
OVA_047	chr1	112018628	T	A	C1orf162	4121	4	4125	0.09697	4183	980	5163	18.98121	0
OVA_047	chr1	1225978	C	G	SCNN1D	1040	0	1040	0	552	241	793	30.39092	8.75E-114
OVA_047	chr11	281391	G	T	NLRP6	1640	0	1640	0	1496	342	1838	18.60718	6.46E-140
OVA_047	chr11	33054552	C	A	DEPDC7	3847	4	3851	0.103869	2333	550	2883	19.07735	1.46E-279
OVA_047	chr1	150808855	G	T	ARNT	8474	7	8481	0.082537	5460	944	6404	14.74079	0
OVA_047	chr11	60468827	C	A	MS4A8	3384	1	3385	0.029542	1953	622	2575	24.15534	0
OVA_047	chr11	86519121	C	T	PRSS23	6437	6	6443	0.093124	4526	985	5511	17.87334	0
OVA_047	chr1	196451359	A	G	KCNT2	3972	1	3973	0.02517	3757	237	3994	5.933901	4.21E-98
OVA_047	chr1	200946402	G	A	KIF21B	2489	0	2489	0	1344	350	1694	20.66116	3.71E-197
OVA_047	chr1	209878264	G	T	HSD11B1	2048	0	2048	0	1718	886	2604	34.02458	0
OVA_047	chr12	104256013	G	T	GNN	3780	1	3781	0.026448	2068	941	3009	31.27285	0
OVA_047	chr12	122243740	G	C	SETD1B	2305	0	2305	0	1773	522	2295	22.7451	5.65E-227
OVA_047	chr12	124184197	C	G	TCTN2	4568	1	4569	0.021887	4270	358	4628	7.735523	1.23E-166
OVA_047	chr1	213415524	C	G	RPS6KC1	3535	2	3537	0.056545	2812	1576	4388	35.91613	0
OVA_047	chr12	4763714	T	C	NDUFA9	6057	0	6057	0	4401	630	5031	12.52236	0
OVA_047	chr12	52980689	T	A	KRT72	3151	0	3151	0	2420	278	2698	10.30393	6.34E-136
OVA_047	chr1	26349122	G	C	EXTL1	1962	0	1962	0	904	460	1364	33.72434	8.17E-271
OVA_047	chr1	27210702	C	G	GPN2	3581	0	3581	0	1941	578	2519	22.94561	0
OVA_047	chr1	28167527	C	A	PPP1R8	6304	1	6305	0.01586	3110	1371	4481	30.59585	0
OVA_047	chr13	23909502	G	T	SACS	5457	1	5458	0.018322	3180	864	4044	21.36499	0
OVA_047	chr1	32568255	T	G	TMEM39B	2272	0	2272	0	2233	529	2762	19.15279	4.44E-189
OVA_047	chr13	51519641	G	A	RNASEH2B	5345	2	5347	0.037404	4591	284	4875	5.825641	9.34E-125
OVA_047	chr14	105055225	G	C	C14orf180	1507	0	1507	0	1122	272	1394	19.5122	9.67E-115
OVA_047	chr14	21500520	G	T	NDRG2	5883	4	5887	0.067946	3155	893	4048	22.06028	0
OVA_047	chr1	43616438	C	A	FAM183A	3302	3	3305	0.090772	2510	1451	3961	36.63216	0
OVA_047	chr14	60933669	T	G	C14orf39	3886	1	3887	0.025727	2274	994	3268	30.41616	0
OVA_047	chr15	99671814	T	A	SYNM	2086	2	2088	0.095785	1904	454	2358	19.2536	1.41E-175
OVA_047	chr16	31092739	C	A	ZNF646	3128	5	3133	0.159591	1948	589	2537	23.2164	6.01E-295
OVA_047	chr16	67235954	C	T	ELMO3	1086	1	1087	0.091996	436	363	799	45.43179	2.91E-209
OVA_047	chr16	88932088	C	A	PABPN1L	1196	2	1198	0.166945	920	175	1095	15.98174	6.00E-80
OVA_047	chr17	47302343	G	A	PHOSPHO1	2581	4	2585	0.154739	2078	145	2223	6.522717	3.29E-67

Sample	Chr	Position	Ref	Alt	gene	pri_ref	pri_alt	pri_total	pri_vaf	met_ref	met_alt	met_total	met_vaf	p-value	
OVA_047	chr17	47394823	C	T	ZNF652	5480	3	5483	0.054715	3087	1454	4541	32.01938	0	
OVA_047	chr17	7758556	T	C	TMEM88	1934	2	1936	0.103306	1004	96	1100	8.727273	5.07E-52	
OVA_047	chr17	79911090	C	A	NOTUM	1133	1	1134	0.088183	1041	211	1252	16.85304	2.71E-75	
OVA_047	chr17	882820	C	A	NXN	949	0	949	0	508	36	544	6.617647	4.31E-21	
OVA_047	chr18	3879149	T	A	DLGAP1	3811	4	3815	0.104849	3270	760	4030	18.85856	0	
OVA_047	chr18	7012173	A	T	LAMA1	2388	0	2388	0	2417	154	2571	5.989887	5.97E-64	
OVA_047	chr19	12783761	G	C	WDR83	651	0	651	0	354	161	515	31.26214	7.81E-80	
OVA_047	chr19	13041385	T	A	FARSA	1340	0	1340	0	695	362	1057	34.24787	2.27E-174	
OVA_047	chr19	17720772	G	C	UNC13A	3700	0	3700	0	3599	183	3782	4.83871	3.33E-83	
OVA_047	chr19	1986631	G	A	BTBD2	1074	1	1075	0.093023	788	56	844	6.635071	1.01E-23	
OVA_047	chr19	36303084	G	T	PRODH2	2153	1	2154	0.046425	1233	427	1660	25.72289	1.49E-223	
OVA_047	chr19	6908817	T	A	EMR1	3048	5	3053	0.163773	1702	740	2442	30.30303	0	
OVA_047	chr19	9056398	C	G	MUC16	4801	2	4803	0.041641	3578	307	3885	7.902188	3.00E-181	
OVA_047	chr20	19496156	T	G	SLC24A3	6866	4	6870	0.058224	4752	1089	5841	18.64407	0	
OVA_047	chr20	3214965	G	C	SLC4A11	2364	1	2365	0.042283	1299	280	1579	17.73274	4.94E-155	
OVA_047	chr20	47292729	A	C	PREX1	4250	3	4253	0.070538	2698	612	3310	18.48943	1.60E-305	
OVA_047	chr20	49348330	C	G	PARD6B	61	0	61	0	53	17	70	24.28571	7.14E-06	
OVA_047	chr20	49621067	G	C	KCNG1	739	0	739	0	441	105	546	19.23077	1.91E-52	
OVA_047	chr20	52570049	G	T	BCAS1	5080	0	5080	0	4409	920	5329	17.26403	0	
OVA_047	chr20	61638072	C	A	BHLHE23	117	0	117	0	73	32	105	30.47619	2.19E-14	
OVA_047	chr2	119604084	G	T	EN1	305	0	305	0	164	68	232	29.31034	4.15E-33	
OVA_047	chr21	26979751	G	T	MRPL39	1782	0	1782	0	1308	84	1392	6.034483	5.98E-38	
OVA_047	chr2	179400166	C	T	TTN	10181	11	10192	0.107928	6756	1583	8339	18.98309	0	
OVA_047	chr2	179428925	C	T	TTN	8290	10	8300	0.120482	5491	1218	6709	18.15472	0	
OVA_047	chr2	187529236	C	G	ITGAV	7464	0	7464	0	4822	1035	5857	17.67116	0	
OVA_047	chr2	189872665	G	T	COL3A1	6583	1	6584	0.015188	4203	686	4889	14.0315	0	
OVA_047	chr22	45937119	G	T	FBLN1	4759	2	4761	0.042008	2364	1085	3449	31.45839	0	
OVA_047	chr2	74656043	T	C	RTKN	5566	2	5568	0.03592	4498	1085	5583	19.434	0	
OVA_047	chr3	12475528	A	G	PPARG	4640	1	4641	0.021547	3091	831	3922	21.18817	0	
OVA_047	chr3	133118886	G	A	BFSP2	1994	1	1995	0.050125	1401	549	1950	28.15385	3.28E-225	
OVA_047	chr3	134331867	C	T	KY	3729	5	3734	0.133905	2858	768	3626	21.18036	0	
OVA_047	chr3	150931275	T	C	P2RY14	10038	4	10042	0.039833	4531	1593	6124	26.01241	0	
OVA_047	chr3	151087242	T	A	MED12L	8098	4	8102	0.049371	3683	1243	4926	25.23346	0	
OVA_047	chr3	48684391	A	C	CELSR3	3382	1	3383	0.02956	1657	534	2191	24.37243	302342e-311	
OVA_047	chr3	50230755	C	T	GNAT1	5954	3	5957	0.050361	3924	65	3989	1.629481	5.57E-37	
OVA_047	chr3	52416390	G	T	DNAH1	6097	1	6098	0.016399	3154	1016	4170	24.36451	0	
OVA_047	chr3	65364932	T	A	AK124433	4003	0	4003	0	2647	744	3391	21.94043	0	
OVA_047	chr3	68802128	C	T	FAM19A4	3599	5	3604	0.138735	2827	867	3694	23.47049	0	
OVA_047	chr3	78696782	T	C	ROBO1	5640	4	5644	0.070872	4210	871	5081	17.14229	0	
OVA_047	chr3	85851368	T	C	CADM2-AS2	1840	3	1843	0.162778	1680	439	2119	20.71732	4.18E-149	
OVA_047	chr4	152638115	T	C	PET112	4163	1	4164	0.024015	3195	220	3415	6.442167	7.98E-111	
OVA_047	chr4	7016217	A	C	TBC1D14	6761	1	6762	0.014789	3891	1081	4972	21.74175	0	
OVA_047	chr5	10262811	C	G	CCT5	10290	2	10292	0.019433	5891	1319	7210	18.29404	0	
OVA_047	chr5	124080118	C	G	ZNF608	1880	0	1880	0	1037	359	1396	25.71633	4.84E-185	
OVA_047	chr5	136315121	G	C	SPOCK1	2966	0	2966	0	1699	787	2486	31.65728	0	
OVA_047	chr5	5232442	G	A	ADAMTS16	6634	3	6637	0.045201	4799	761	5560	13.68705	0	
OVA_047	chr5	95735615	G	C	PCSK1	3237	0	3237	0	1846	854	2700	31.62963	0	
OVA_047	chr6	110988691	C	A	CDK19	3812	6	3818	0.15715	2889	816	3705	22.02429	0	
OVA_047	chr6	160491056	C	T	IGF2R	5460	3	5463	0.054915	2750	1218	3968	30.69556	0	
OVA_047	chr6	161011883	G	T	LPA	3258	2	3260	0.06135	2453	166	2619	6.338297	4.96E-74	
OVA_047	chr6	161011884	T	C	LPA	3301	0	3301	0	2484	168	2652	6.334842	2.46E-80	
OVA_047	chr6	38670851	G	C	GLO1	3458	0	3458	0	2281	451	2732	16.50805	4.89E-252	
OVA_047	chr6	65303164	C	A	EYS	4932	5	4937	0.101276	3182	974	4156	23.436	0	
OVA_047	chr7	103835647	C	T	ORC5	3593	845	4438	19.04011	2333	980	3313	29.58044	3.05E-37	
OVA_047	chr7	112407360	A	G	TMEM168	6079	2	6081	0.032889	3921	1243	5164	24.07049	0	
OVA_047	chr7	150217635	A	T	GIMAP7	6243	3	6246	0.048031	5820	1130	6950	16.25899	0	
OVA_047	chr7	156802922	g	T	MNX1	140	0	140	0	145	47	192	24.47917	3.23E-15	
OVA_047	chr7	48451920	G	C	ABCA13	3955	0	3955	0	2766	648	3414	18.98067	0	
OVA_047	chr7	6544447	G	C	GRID2IP	1395	2	1397	0.143164	695	600	1295	46.33205	2.22E-296	
OVA_047	chr7	86781654	T	A	035377;DM	3738	2	3740	0.053476	1682	672	2354	28.54715	0	
OVA_047	chr8	143746002	c	G	JRK	2078	0	2078	0	1510	199	1709	11.64424	2.89E-84	
OVA_047	chr8	43157190	T	A	POTEA	3128	1	3129	0.031959	2198	610	2808	21.72365	3.21E-275	
OVA_047	chr8	98711981	G	T	MTDH	5693	8	5701	0.140326	3154	1815	4969	36.52646	0	
OVA_047	chr9	113563128	C	T	MUSK	4757	4	4761	0.084016	4010	1000	5010	19.96008	0	
OVA_047	chr9	133352355	G	A	ASS1	1362	0	1362	0	899	236	1135	20.79295	1.78E-105	
OVA_047	chr9	133455206	G	T	FUBP3	241	0	241	0	190	15	205	7.317073	4.00E-06	
OVA_047	chr9	87563385	G	A	NTRK2	7842	5	7847	0.063719	5002	1146	6148	18.64021	0	
OVA_047	chr9	94486599	A	G	ROR2	2133	1	2134	0.04686	1186	246	1432	17.17877	2.86E-126	
OVA_047	chr9	99581474	T	A	ZNF782	3893	0	3893	0	5596	916	6512	14.06634	3.66E-288	
OVA_047	chrX	114541295	C	T	LUZP4	3601	4	3605	0.110957	2225	696	2921	23.82746	0	
OVA_047	chrX	125299898	G	T	DCAF12L2	704	0	704	0	321	101	422	23.93365	6.54E-54	
OVA_047	chrX	12809717	A	T	PRPS2	581	0	581	0	366	104	470	22.12766	2.73E-51	

Sample	Chr	Position	Ref	Alt	gene	pri_ref	pri_alt	pri_total	pri_vaf	met_ref	met_alt	met_total	met_vaf	p-value	
OVA_047	chrX	21675953	G	A	KLHL34	1580	2	1582	0.126422	1139	107	1246	8.58748	8.64E-54	
OVA_047	chrX	47775433	T	A	ZNF81	3171	2	3173	0.063032	2334	633	2967	21.33468	6.09E-272	
OVA_047	chrX	53279887	T	A	IQSEC2	1188	1	1189	0.084104	553	187	740	25.27027	2.40E-99	
OVA_048	chr10	102746642	A	C	MRPL43	3219	0	3219	0	3137	1020	4157	24.53693	0	
OVA_048	chr10	105173871	G	C	PDCD11	6076	2	6078	0.032906	7083	523	7606	6.87615	0	
OVA_048	chr10	107007099	C	A	SORCS3	1756	4	1760	0.227273	1264	698	1962	35.57594	2.94E-304	
OVA_048	chr10	116057085	T	C	AFAP1L2	2263	2	2265	0.0883	2534	282	2816	10.0142	2.01E-140	
OVA_048	chr10	12288259	G	A	CDC123	3661	6	3667	0.163621	2374	1111	3485	31.87948	0	
OVA_048	chr10	61868056	C	G	ANK3	3068	0	3068	0	3362	111	3473	3.196084	1.24E-50	
OVA_048	chr10	69571270	C	T	DNAJC12	4630	13	4643	0.279991	4177	1717	5894	29.13132	0	
OVA_048	chr1	100856461	T	C	CDC14A	2516	0	2516	0	2503	1862	4365	42.6575	0	
OVA_048	chr11	123479191	G	A	GRAMD1B	4251	17	4268	0.398313	3997	2825	6822	41.41014	0	
OVA_048	chr1	114401967	A	G	B1-AS1;PTP	1975	0	1975	0	2705	514	3219	15.96769	1.04E-166	
OVA_048	chr1	114442499	C	T	4B1;AP4B1-A	1885	0	1885	0	3650	103	3753	2.744471	8.20E-27	
OVA_048	chr11	34176296	G	C	ABTB2	1340	0	1340	0	1899	250	2149	11.63332	1.68E-114	
OVA_048	chr1	149881045	A	T	SV2A	483	6	489	1.226994	746	73	819	8.913309	5.14E-13	
OVA_048	chr1	153659735	C	T	NPR1	3739	2	3741	0.053462	6485	491	6976	7.038417	2.68E-182	
OVA_048	chr1	156836704	A	G	NTRK1	1219	0	1219	0	973	750	1723	43.52873	7.77E-293	
OVA_048	chr1	1747328	T	C	GNB1	3673	0	3673	0	6282	1888	8170	23.10894	0	
OVA_048	chr1	179401558	C	A	AXDND1	1767	1	1768	0.056561	1879	655	2534	25.84846	7.10E-241	
OVA_048	chr1	180904917	C	T	KIAA1614	817	0	817	0	1237	158	1395	11.32616	1.45E-55	
OVA_048	chr1	181620542	C	G	CACNA1E	4101	3	4104	0.073099	3823	2295	6118	37.51226	0	
OVA_048	chr11	92577560	C	T	FAT3	2974	2	2976	0.067204	6046	187	6233	3.00016	1.16E-55	
OVA_048	chr1	201816331	G	A	IPO9	2459	3	2462	0.121852	2047	293	2340	12.52137	1.43E-124	
OVA_048	chr1	204985553	T	G	NFASC	3057	1	3058	0.032701	5592	706	6298	11.20991	2.71E-258	
OVA_048	chr12	108985583	C	T	TMEM119	281	0	281	0	266	27	293	9.215017	1.63E-10	
OVA_048	chr12	114832600	A	T	TBX5	1714	1	1715	0.058309	3162	68	3230	2.105263	4.69E-25	
OVA_048	chr12	129189760	G	T	TMEM132C	757	0	757	0	1262	183	1445	12.66436	7.24E-53	
OVA_048	chr12	39719694	T	G	KIF21A	1160	0	1160	0	639	560	1199	46.70559	435183e-320	
OVA_048	chr1	25572949	C	G	C1orf63	3863	1	3864	0.02588	3865	2490	6355	39.18175	0	
OVA_048	chr12	58007183	G	A	BC073932	1140	1	1141	0.087642	1516	110	1626	6.765068	2.05E-34	
OVA_048	chr13	114149951	G	A	TMCO3	1626	2	1628	0.12285	1274	950	2224	42.71583	0	
OVA_048	chr1	33231446	G	T	KIAA1522	1598	3	1601	0.187383	1860	49	1909	2.566789	1.82E-13	
OVA_048	chr14	101004460	T	A	BEGAIN	50	18	68	26.47059	78	25	103	24.27184	0.706604	
OVA_048	chr14	103596332	C	A	TNFAIP2	2009	1	2010	0.049751	2994	67	3061	2.188827	6.64E-21	
OVA_048	chr14	31614057	C	A	HECTD1	6050	2	6052	0.033047	6786	1314	8100	16.22222	0	
OVA_048	chr14	64954541	G	A	ZBTB25	2796	2	2798	0.07148	2440	889	3329	26.70472	0	
OVA_048	chr16	30528968	A	T	ITGAL	487	0	487	0	671	22	693	3.174603	3.07E-06	
OVA_048	chr16	3711948	G	A	BC095475	901	0	901	0	1263	41	1304	3.144172	2.13E-12	
OVA_048	chr16	4414664	G	A	)7;CORO7-P	385	0	385	0	478	52	530	9.811321	7.08E-19	
OVA_048	chr16	57989136	G	A	CNGB1	1633	3	1636	0.183374	1693	113	1806	6.256921	4.65E-49	
OVA_048	chr16	57989148	G	C	CNGB1	1747	0	1747	0	1772	110	1882	5.844846	2.00E-52	
OVA_048	chr16	67197105	G	C	FBXL8	1308	0	1308	0	829	439	1268	34.62145	9.77E-217	
OVA_048	chr16	67688253	C	T	RLTPR	355	0	355	0	370	13	383	3.394256	0.000105	
OVA_048	chr16	84600434	T	C	COTL1	570	0	570	0	559	69	628	10.98726	2.78E-27	
OVA_048	chr17	26861690	G	T	FOXN1	423	1	424	0.235849	414	45	459	9.803922	5.45E-17	
OVA_048	chr17	27234780	C	T	PHF12	1867	2	1869	0.107009	2953	78	3031	2.573408	5.38E-35	
OVA_048	chr17	2995529	T	A	OR1D2	1818	3	1821	0.164745	2955	838	3793	22.09333	5.87E-258	
OVA_048	chr17	39882046	G	A	HAP1;JUP	673	0	673	0	656	427	1083	39.42752	9.69E-146	
OVA_048	chr17	72868835	C	A	FDXR	2210	3	2213	0.135563	3612	332	3944	8.41785	8.80E-121	
OVA_048	chr17	7330259	G	C	C17orf74	426	0	426	0	260	462	722	63.98892	1.28E-221	
OVA_048	chr17	8383800	C	T	MYH10	314	3	317	0.946372	581	19	600	3.166667	0.003855	
OVA_048	chr18	22804534	T	A	ZNF521	2834	0	2834	0	3558	1375	4933	27.8735	0	
OVA_048	chr18	2960707	C	G	LPIN2	6775	1	6776	0.014758	4895	2923	7818	37.38808	0	
OVA_048	chr1	84991595	C	G	SPATA1	2643	0	2643	0	4001	562	4563	12.31646	3.79E-183	
OVA_048	chr1	90152010	G	C	LRRC8C	1882	2	1884	0.106157	3909	116	4025	2.881988	1.32E-23	
OVA_048	chr19	14270308	G	T	LPHN1	2843	1	2844	0.035162	3766	882	4648	18.9759	0	
OVA_048	chr19	36168972	C	T	UPK1A	1790	1	1791	0.055835	3220	93	3313	2.807123	1.34E-31	
OVA_048	chr19	38573615	C	T	SIPA1L3	837	0	837	0	555	357	912	39.14474	1.13E-163	
OVA_048	chr19	41198202	C	A	ADCK4	361	0	361	0	191	113	304	37.17105	2.33E-63	
OVA_048	chr19	51380134	G	T	KLK2	1487	2	1489	0.134318	1497	833	2330	35.75107	0	
OVA_048	chr19	7937071	C	T	FLJ22184	212	0	212	0	140	238	378	62.96296	4.55E-81	
OVA_048	chr19	9251072	c	T	ZNF317	2893	0	2893	0	2145	217	2362	9.18713	7.27E-101	
OVA_048	chr20	62699475	C	A	TCEA2	1104	1	1105	0.090498	1129	853	1982	43.03734	5.04E-300	
OVA_048	chr2	10102642	T	A	GRHL1	2547	2	2549	0.078462	2833	1573	4406	35.70132	0	
OVA_048	chr2	120005606	C	T	STEAP3	1459	0	1459	0	2001	379	2380	15.92437	1.33E-135	
OVA_048	chr2	169830439	T	A	ABCB11	5684	1	5685	0.01759	7081	582	7663	7.594937	083521e-315	
OVA_048	chr2	193056705	G	C	TMEFF2	2338	0	2338	0	2365	56	2421	2.313094	4.33E-29	
OVA_048	chr2	201876137	G	T	FAM126B	746	257	1003	25.62313	685	460	1145	40.17467	5.39E-20	
OVA_048	chr22	26351072	C	G	MYO18B	549	0	549	0	639	459	1098	41.80328	3.97E-133	
OVA_048	chr22	43015885	T	C	CYB5R3	926	0	926	0	2115	82	2197	3.732362	3.29E-18	
OVA_048	chr22	46611248	C	G	PPARA	1673	0	1673	0	3631	87	3718	2.339968	4.10E-20	

Sample	Chr	Position	Ref	Alt	gene	pri_ref	pri_alt	pri_total	pri_vaf	met_ref	met_alt	met_total	met_vaf	p-value	
OVA_048	chr22	50471709	T	C	.17REL;MLC	1859	1	1860	0.053763	5542	116	5658	2.050194	7.34E-25	
OVA_048	chr2	26534415	C	T	GPR113	1006	1	1007	0.099305	2324	54	2378	2.270816	3.59E-11	
OVA_048	chr2	31417292	G	T	CAPN14	1205	0	1205	0	1370	667	2037	32.74423	1.66E-220	
OVA_048	chr2	39535093	T	G	MAP4K3	2202	1	2203	0.045393	1748	964	2712	35.54572	0	
OVA_048	chr2	71797954	G	T	DYSF	3091	1	3092	0.032342	4655	1950	6605	29.52309	0	
OVA_048	chr2	74042301	C	T	C2orf78	3547	1	3548	0.028185	3752	274	4026	6.805763	4.17E-106	
OVA_048	chr3	119459549	C	T	MAATS1	922	0	922	0	827	93	920	10.1087	6.96E-38	
OVA_048	chr3	134264557	C	T	CEP63	3417	8	3425	0.233577	2605	1529	4134	36.98597	0	
OVA_048	chr3	171844772	C	A	FNDC3B	6379	4	6383	0.062666	9540	507	10047	5.046282	4.08E-271	
OVA_048	chr3	49040154	C	T	P4HTM	1332	2	1334	0.149925	1243	92	1335	6.891386	1.98E-33	
OVA_048	chr3	50324655	C	T	LSMEM2	879	2	881	0.227015	778	446	1224	36.43791	2.40E-161	
OVA_048	chr3	66455764	C	T	LRIG1	1282	2	1284	0.155763	1377	183	1560	11.73077	2.19E-64	
OVA_048	chr3	71834134	C	T	PROK2	121	0	121	0	178	13	191	6.806283	0.000215	
OVA_048	chr3	71834147	C	G	PROK2	134	0	134	0	173	15	188	7.978723	2.45E-05	
OVA_048	chr3	97454908	A	C	EPHA6	4163	0	4163	0	4071	2366	6437	36.75625	0	
OVA_048	chr3	99514530	G	T	COL8A1	2283	2	2285	0.087527	3131	447	3578	12.49301	2.56E-172	
OVA_048	chr4	153897079	G	C	FHDC1	152	0	152	0	167	8	175	4.571429	0.005326	
OVA_048	chr5	133536118	C	A	PPP2CA	3205	1	3206	0.031192	1873	2466	4339	56.83337	0	
OVA_048	chr5	140736302	G	T	PCDHGA4	2070	0	2070	0	3651	129	3780	3.412698	2.78E-46	
OVA_048	chr6	13978053	G	A	RNF182	3668	2	3670	0.054496	5219	377	5596	6.736955	4.32E-136	
OVA_048	chr6	159399385	T	G	RSPH3	1579	0	1579	0	991	645	1636	39.42543	1.33E-302	
OVA_048	chr6	170059154	A	T	WDR27	2324	2	2326	0.085985	2958	117	3075	3.804878	3.86E-44	
OVA_048	chr6	36274103	C	T	PNPLA1	2152	7	2159	0.324224	1640	888	2528	35.12658	0	
OVA_048	chr6	39283130	C	T	KCNK16	1803	4	1807	0.221361	2535	53	2588	2.047913	5.18E-11	
OVA_048	chr6	43402571	G	C	ABCC10	0	0	0	#DIV/0!	1	144	145	99.31034	2.04E-55	
OVA_048	chr6	6320968	G	C	F13A1	742	0	742	0	982	78	1060	7.358491	4.99E-22	
OVA_048	chr6	73951871	G	C	KHDC1	3243	0	3243	0	2882	3652	6534	55.89226	0	
OVA_048	chr6	89790893	C	A	PNRC1	296	2	298	0.671141	162	21	183	11.47541	1.39E-11	
OVA_048	chr6	96044626	C	G	MANEA	2340	2	2342	0.085397	2038	569	2607	21.82585	4.48E-248	
OVA_048	chr7	100797855	G	T	AP1S1	51	0	51	0	43	23	66	34.84848	4.16E-08	
OVA_048	chr7	4798660	G	A	FOXK1	305	0	305	0	159	306	465	65.80645	1.93E-126	
OVA_048	chr7	48313918	A	G	CA13;AX746	831	2	833	0.240096	512	256	768	33.33333	4.64E-117	
OVA_048	chr7	56065091	C	T	GBAS	5230	10	5240	0.19084	7142	1146	8288	13.82722	0	
OVA_048	chr8	55539149	T	G	RP1	2151	1	2152	0.046468	1395	620	2015	30.76923	2.57E-306	
OVA_048	chr8	59535979	T	A	NSMAF	5395	0	5395	0	4734	1656	6390	25.91549	0	
OVA_048	chr8	99042813	G	C	MATN2	5923	1	5924	0.01688	6877	1901	8778	21.65641	0	
OVA_048	chr9	116811615	C	G	ZNF618	1540	0	1540	0	2285	364	2649	13.74103	9.79E-118	
OVA_048	chr9	134394025	C	A	POMT1	1786	0	1786	0	2442	238	2680	8.880597	1.57E-88	
OVA_048	chrX	100911666	T	G	ARMCX2	2292	0	2292	0	2131	895	3026	29.577	0	
OVA_048	chrX	138871562	T	A	ATP11C	5793	2	5795	0.034513	5956	1630	7586	21.48695	0	
OVA_048	chrX	50121596	G	A	DGKK	3541	0	3541	0	5259	730	5989	12.18901	0	
OVA_048	chrX	83442803	G	A	RPS6KA6	818	0	818	0	852	21	873	2.405498	1.93E-09	
OVA_365	chr10	75832579	C	T	VCL	3790	5	3795	0.131752	5462	963	6425	14.98833	0	
OVA_365	chr11	68825028	C	T	TPCN2	983	2	985	0.203046	838	266	1104	24.0942	6.27E-128	
OVA_365	chr12	14609563	T	G	ATF7IP	2359	1	2360	0.042373	3934	167	4101	4.072178	1.44E-57	
OVA_365	chr1	228401949	G	T	OBSCN	470	0	470	0	683	20	703	2.84495	3.34E-07	
OVA_365	chr13	111368091	G	A	ING1	83	0	83	0	33	76	109	69.72477	2.03E-37	
OVA_365	chr13	77459768	G	A	KCTD12	45	0	45	0	43	23	66	34.84848	2.87E-09	
OVA_365	chr14	23845069	C	T	IL25	1060	4	1064	0.37594	1163	558	1721	32.42301	2.97E-213	
OVA_365	chr14	64473851	C	A	SYNE2	1274	3	1277	0.234926	1939	197	2136	9.222846	5.79E-55	
OVA_365	chr17	45915779	C	G	SCRN2	446	0	446	0	1074	145	1219	11.895	1.46E-32	
OVA_365	chr17	52991985	G	C	TOM1L1	2690	3	2693	0.1114	6218	104	6322	1.645049	1.58E-28	
OVA_365	chr17	62502375	G	A	DDX5	1098	2	1100	0.181818	1823	130	1953	6.656426	8.53E-44	
OVA_365	chr17	79278647	C	A	LINC00482	195	142	337	42.1365	74	397	471	84.28875	7.30E-57	
OVA_365	chr18	21735998	A	C	CABYR	3629	2	3631	0.055081	3847	1968	5815	33.84351	0	
OVA_365	chr18	23720176	g	C	PSMA8	723	201	924	21.75325	955	505	1460	34.58904	3.12E-23	
OVA_365	chr19	11473410	G	C	LPPR2	1601	2	1603	0.124766	1176	53	1229	4.312449	9.36E-28	
OVA_365	chr21	43710181	A	T	ABCG1	2218	0	2218	0	2656	59	2715	2.173112	2.23E-39	
OVA_365	chr2	174774899	T	C	SP3	3625	0	3625	0	6139	961	7100	13.53521	0	
OVA_365	chr2	208425099	A	C	CREB1	2036	0	2036	0	3980	171	4151	4.119489	3.62E-47	
OVA_365	chr22	22890659	T	C	PRAME	633	4	637	0.627943	342	375	717	52.30126	2.85E-183	
OVA_365	chr22	40068179	C	G	CACNA1I	1740	0	1740	0	2296	96	2392	4.013378	2.21E-54	
OVA_365	chr22	42828396	G	C	NFAM1	178	0	178	0	75	127	202	62.87129	2.08E-62	
OVA_365	chr2	44223108	G	A	LRPPRC	1230	0	1230	0	1397	22	1419	1.550388	5.54E-08	
OVA_365	chr2	74782367	C	G	DOK1	966	0	966	0	1185	30	1215	2.469136	2.46E-12	
OVA_365	chr3	14724369	C	G	C3orf20	1748	0	1748	0	2409	187	2596	7.20339	4.65E-71	
OVA_365	chr3	160119759	C	T	SMC4	1951	2	1953	0.102407	2881	76	2957	2.570172	1.34E-21	
OVA_365	chr3	42734629	G	C	HHATL	1073	0	1073	0	1449	79	1528	5.170157	5.46E-31	
OVA_365	chr3	49400091	C	G	RHOA	1526	0	1526	0	1950	427	2377	17.96382	2.56E-171	
OVA_365	chr3	52472035	T	C	SEMA3G	539	0	539	0	427	201	628	32.00637	2.25E-91	
OVA_365	chr4	1659302	C	A	FAM53A	1640	0	1640	0	2094	424	2518	16.83876	2.28E-214	
OVA_365	chr4	56301603	A	T	CLOCK	4230	10	4240	0.235849	4368	2362	6730	35.09658	0	

Sample	Chr	Position	Ref	Alt	gene	pri_ref	pri_alt	pri_total	pri_vaf	met_ref	met_alt	met_total	met_vaf	p-value	
OVA_365	chr4	70620855	G	T	SULT1B1	2345	1	2346	0.042626	3823	335	4158	8.056758	2.30E-147	
OVA_365	chr5	150071302	G	T	RBM22	856	2	858	0.2331	820	551	1371	40.18964	4.89E-200	
OVA_365	chr5	167630747	A	C	TENM2	1231	2	1233	0.162206	1883	217	2100	10.33333	2.35E-65	
OVA_365	chr5	33637773	C	A	ADAMTS12	2939	2	2941	0.068004	4942	309	5251	5.884593	8.62E-163	
OVA_365	chr6	130389526	C	A	L3MBTL3	2679	0	2679	0	4588	344	4932	6.974858	2.00E-139	
OVA_365	chr6	32150709	T	A	AGER	1297	0	1297	0	2395	75	2470	3.036437	7.78E-21	
OVA_365	chr6	36260932	G	A	PNPLA1	858	0	858	0	1374	41	1415	2.897527	7.62E-13	
OVA_365	chr7	100205381	C	T	PCOLCE	2580	3	2583	0.116144	3424	75	3499	2.14347	2.31E-34	
OVA_365	chr7	99474086	C	T	OR2AE1	3865	9	3874	0.232318	5203	1557	6760	23.03254	0	
OVA_365	chr8	100821579	G	C	VPS13B	4180	4	4184	0.095602	7674	1843	9517	19.36535	0	
OVA_365	chr8	133584537	C	T	LRRC6	3435	4	3439	0.116313	6949	679	7628	8.901416	8.42E-268	
OVA_365	chr8	9562252	A	T	TNKS	2554	3	2557	0.117325	5174	140	5314	2.63455	1.97E-48	
OVA_365	chrX	100243315	G	C	ARL13A	2334	2	2336	0.085616	3880	87	3967	2.193093	1.40E-40	
OVA_365	chrX	135741311	G	C	CD40LG	1751	0	1751	0	2603	470	3073	15.2945	3.35E-183	
OVA_378	chr12	12332942	A	G	LRP6	1443	12	1455	0.824742	1127	568	1695	33.51032	1.19E-197	
OVA_378	chr13	101881914	T	C	NALCN	1957	0	1957	0	1811	186	1997	9.313971	7.58E-69	
OVA_378	chr14	22928635	T	C	AK093552	7596	50	7646	0.653937	5289	2112	7401	28.53668	0	
OVA_378	chr15	101597023	C	T	LRRK1	3995	29	4024	0.720676	2319	1684	4003	42.06845	0	
OVA_378	chr16	48145707	G	C	ABCC12	2608	20	2628	0.761035	1446	1003	2449	40.95549	0	
OVA_378	chr18	67563165	T	A	CD226	8298	43	8341	0.515526	5701	2332	8033	29.03025	0	
OVA_378	chr2	11767117	C	T	GREB1	5345	26	5371	0.484081	4412	957	5369	17.82455	0	
OVA_378	chr2	179451890	C	T	TTN	8506	780	9286	8.399742	8673	2131	10804	19.72418	9.34E-196	
OVA_378	chr22	21348253	C	T	LZTR1	1645	19	1664	1.141827	1162	654	1816	36.01322	4.30E-239	
OVA_378	chr2	31425111	G	A	CAPN14	4673	1	4674	0.021395	4997	296	5293	5.592292	4.05E-118	
OVA_378	chr3	169656141	A	T	SAMD7	4678	7	4685	0.149413	4123	690	4813	14.33617	2.90E-292	
OVA_378	chr5	127448613	C	A	SLC12A2	3222	10	3232	0.309406	2056	1086	3142	34.56397	0	
OVA_378	chr5	89979692	A	G	GPR98	3026	8	3034	0.263678	2913	200	3113	6.424671	2.52E-63	
OVA_378	chr6	71012630	T	C	COL9A1	2832	10	2842	0.351865	2533	398	2931	13.57898	8.22E-133	

Supplementary Table S6: dN/dS ratio					
**Cancer type**		**Mutations type**	**mle**	**cilow**	**cihigh**
Primary_all	wmis	Missense	1.00481	0.8034352	1.256657
Primary_all	wnon	Nonsense	1.012904	0.6131273	1.673347
Primary_clonal	wmis	Missense	0.936351	0.7080285	1.238302
Primary_clonal	wnon	Nonsense	0.8897847	0.4803563	1.648187
Primary_subclonal	wmis	Missense	1.162666	0.8006717	1.688324
Primary_subclonal	wnon	Nonsense	1.33361	0.5638494	3.154239
Metastasis_all	wmis	Missense	0.9767878	0.7696088	1.23974
Metastasis_all	wnon	Nonsense	1.071679	0.6437012	1.784206
Metastasis_clonal	wmis	Missense	0.9308156	0.6918959	1.252237
Metastasis_clonal	wnon	Nonsense	1.1925614	0.642897	2.212178
Metastasis_subclonal	wmis	Missense	1.063653	0.7121544	1.588641
Metastasis_subclonal	wnon	Nonsense	0.8595513	0.3439302	2.148193

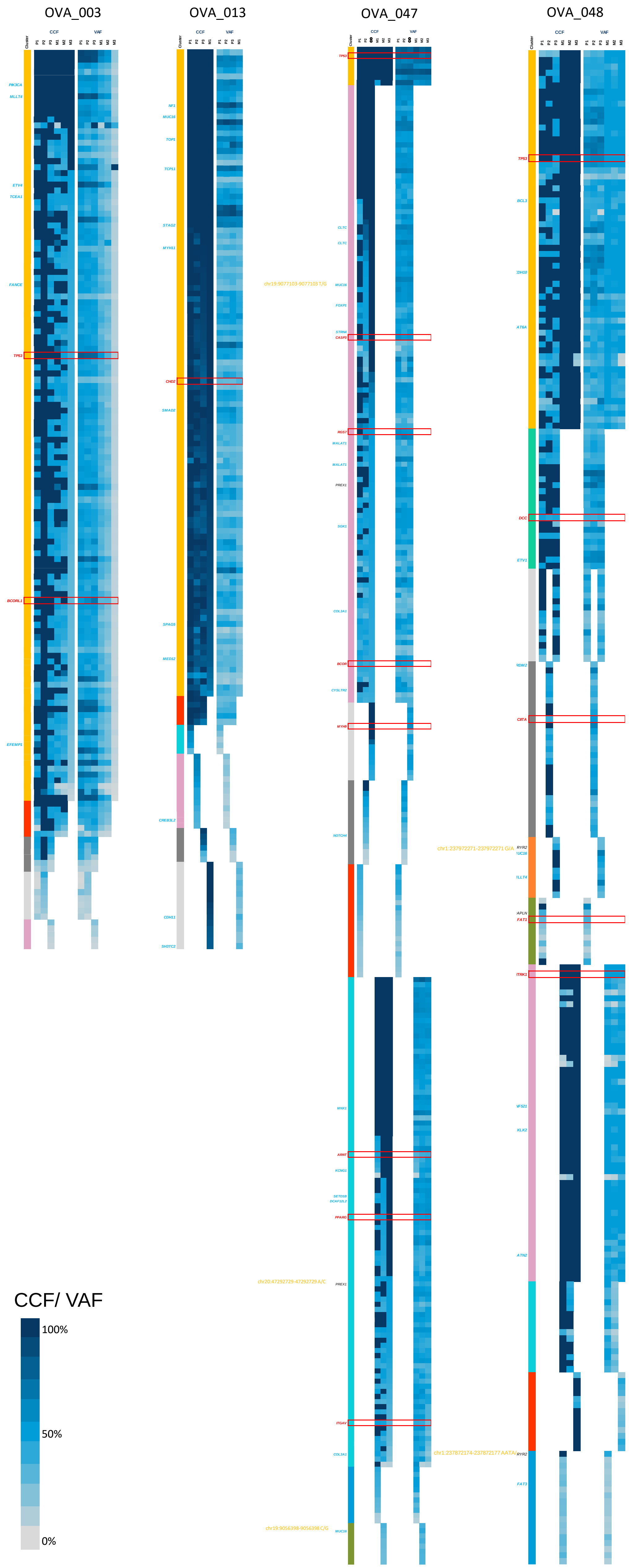

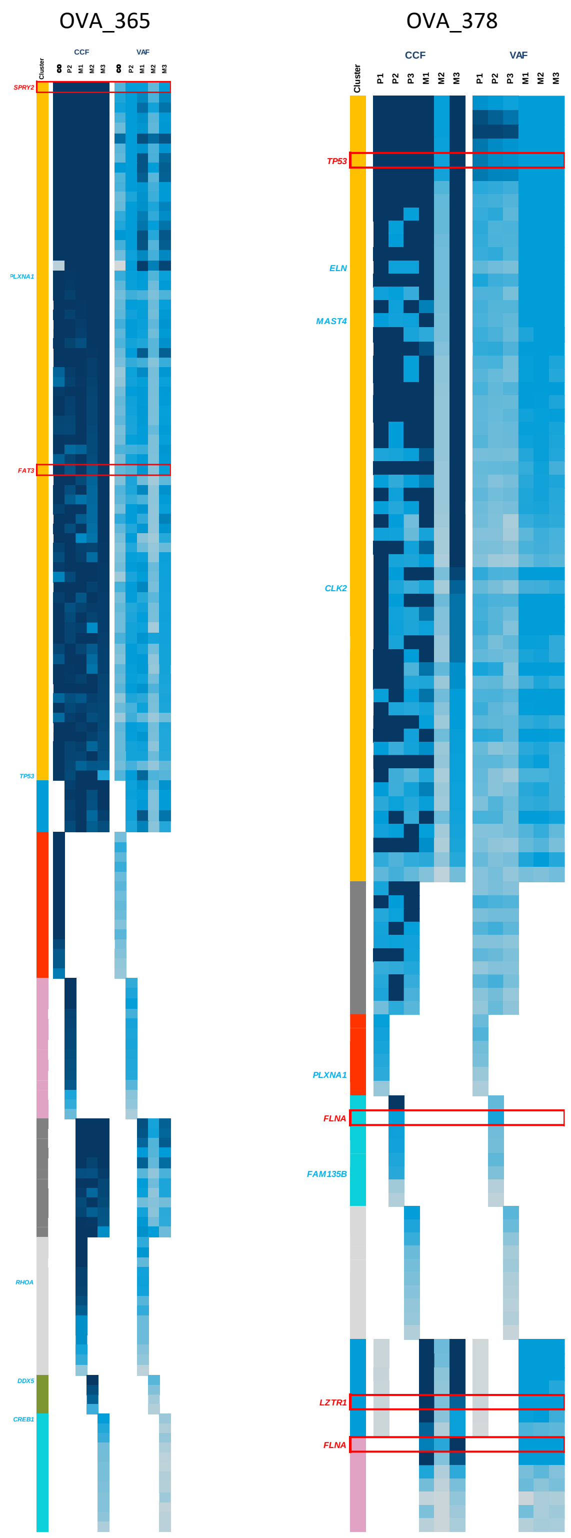

Supplementary Fig. S1: Mutations cluster heatmap of individual samples. Coloured bar represents all mutation clusters, with corresponding cancer cell fractions (CCF) and variant allele frequency (VAF) values as a heatmap. Driver genes are highlighted in red and cancer genes in blue and non-cancer genes in black, with corresponding parallel mutations converging at the same gene indicated in orange.

Supplementary Fig. S2: Phylogenetic trees.

Phylogenetic trees for 6 metastatic HGSOC tumours. For each sample, only the optimal tree derived using LICHeE (Lineage Inference for Cancer Heterogeneity and Evolution) has been presented. Highlighted are driver genes (in red), and parallel events converging at cancer genes (in blue) and non-cancer genes (in black). The middle top pane shows the complete phylogenetic tree as constructed based on mutation clusters, with primary and metastatic phylogenetic trees have been shown separately in a black box. Different coloured nodes represent distinct clones, with the number of mutations contributing to the cluster displayed inside each node. Below are the phylogenetic trees shown for each region, where clusters not found in a given region have been represented as grey empty nodes. “P”, represents regions from primary tumours; “CO”, represents contralateral ovarian metastatic tumour tissue; “M”, represents metastatic tumour tissue. Beneath each regional phylogenetic tree, a grid of a 100 representative cells shows the mutational clusters present via coinciding cluster colours.

OVA_003

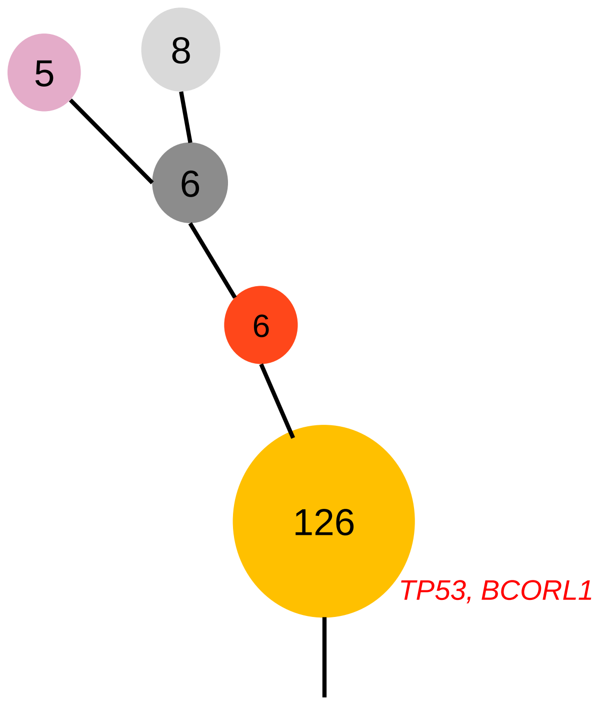

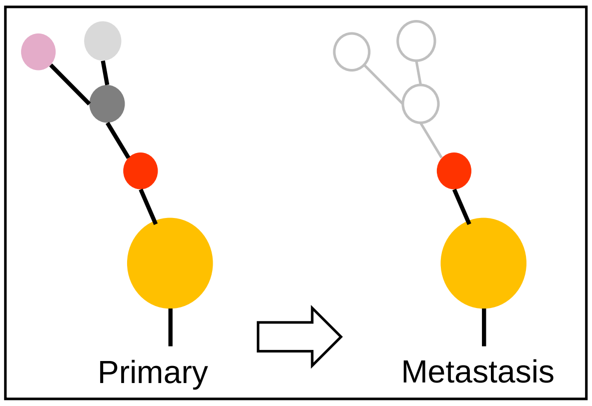

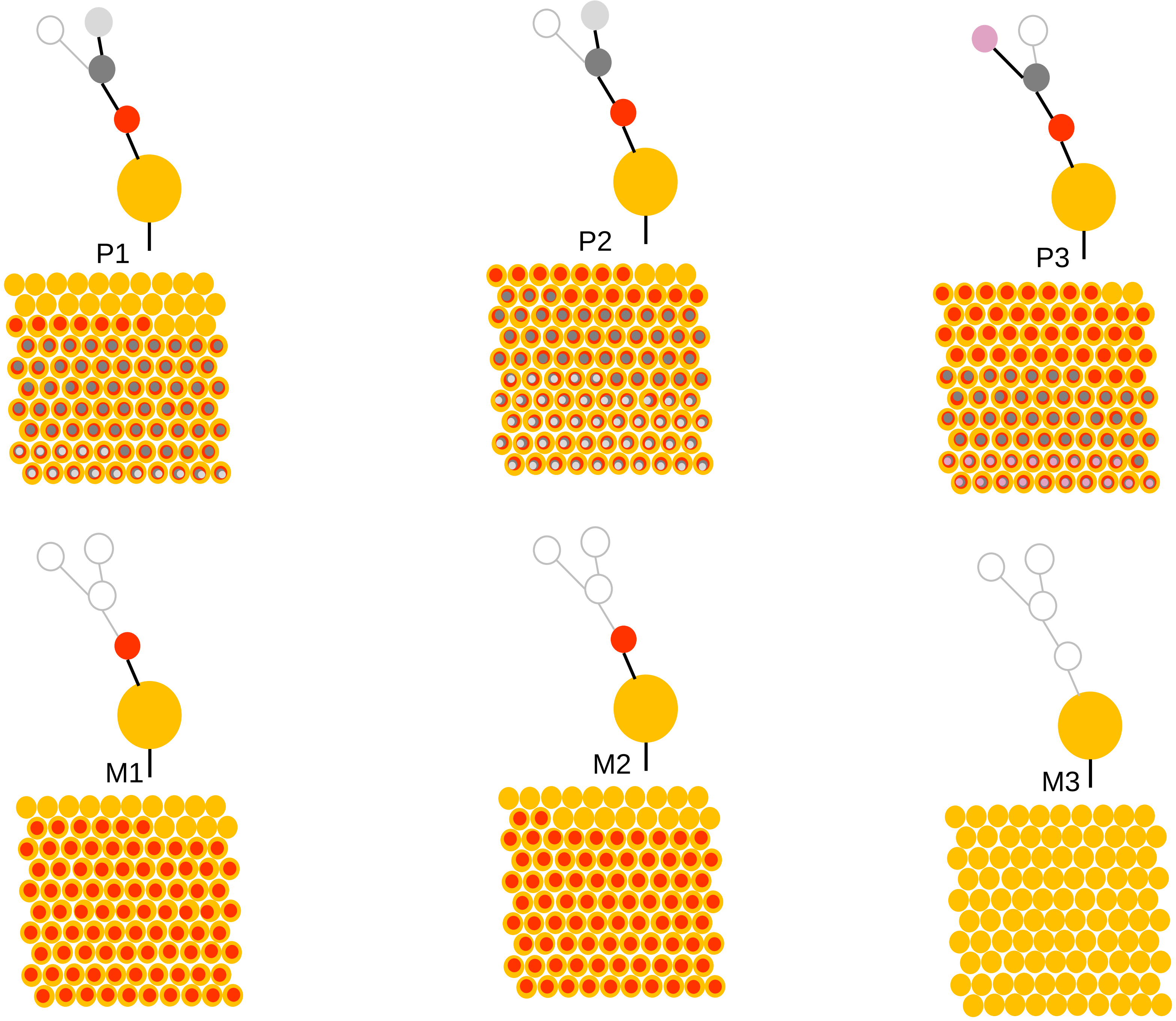

OVA_013

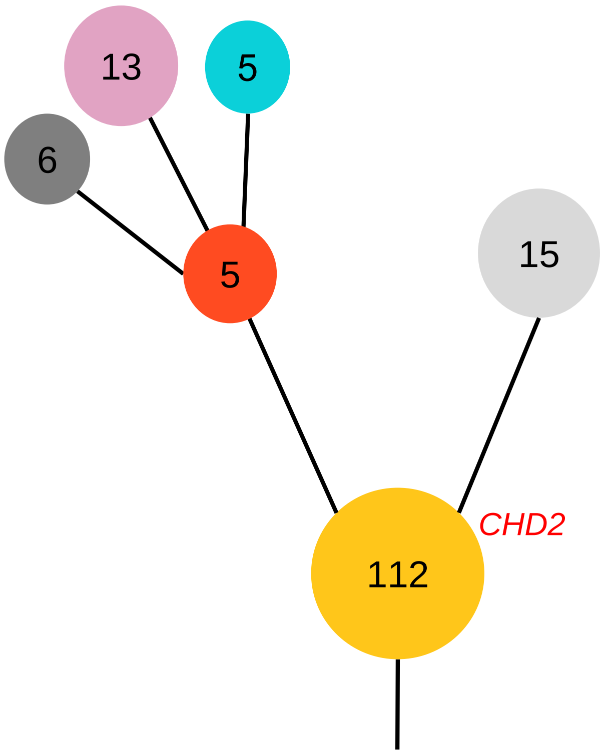

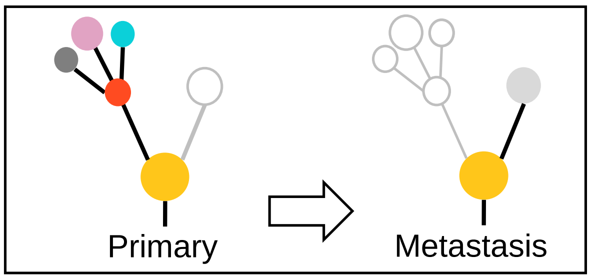

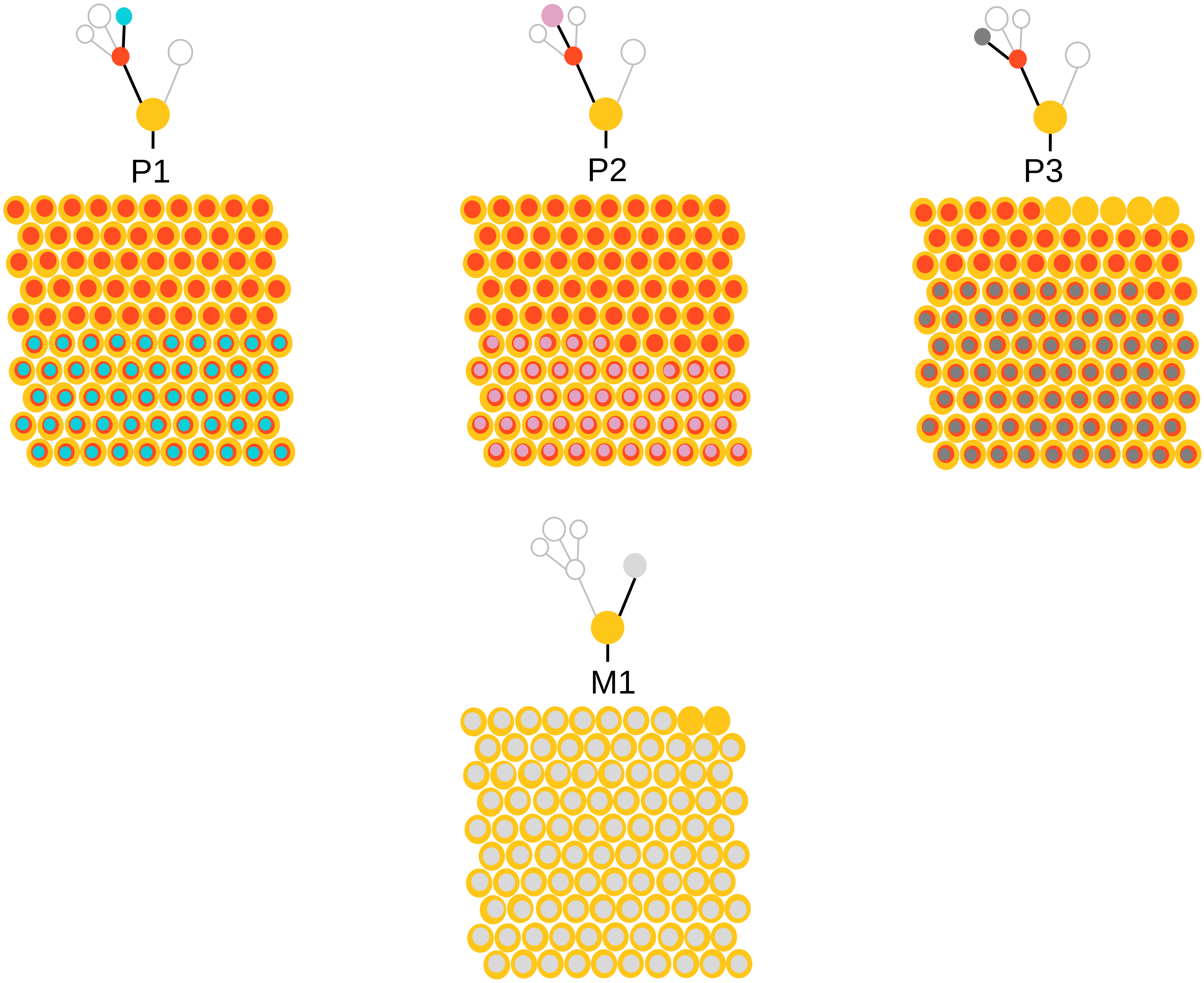

OVA_047

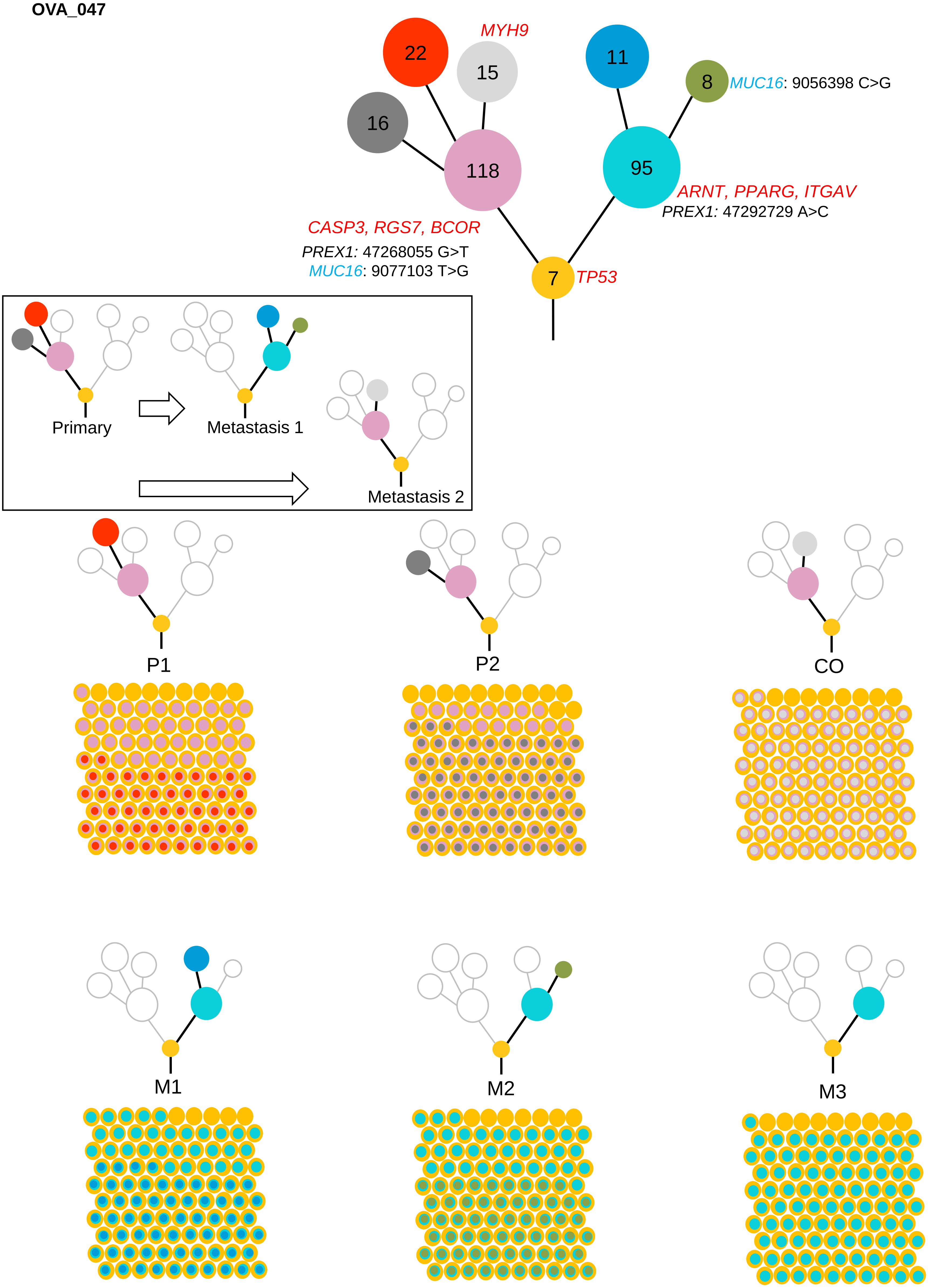
22
15
MYH9
11
8
MUC16: 9056398 C>G
16
118
95
ARNT, PPARG, ITGAV
PREX1: 47292729 A>C
CASP3, RGS7, BCOR
PREX1: 47268055 G>T
MUC16: 9077103 T>G
7
TP53
Primary
Metastasis 1
Metastasis 2
P1
P2
CO
M1
M2
M3

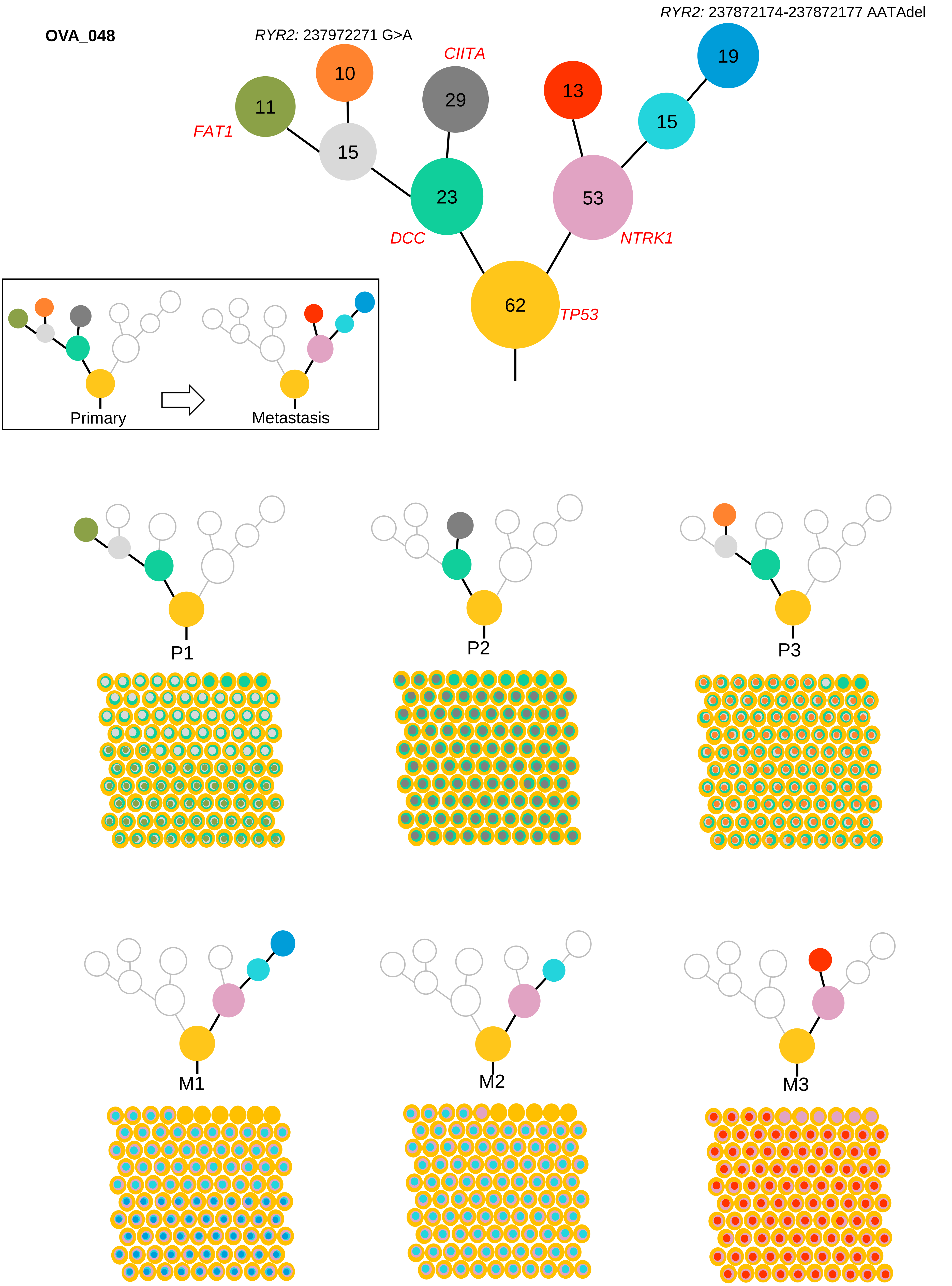
OVA_048
RYR2: 237972271 G>A
CIITA
RYR2: 237872174-237872177 AATAdel
10
11
29
13
19
15
FAT1
15
23
53
DCC
NTRK1
62
TP53
Primary
Metastasis
P1
P2
P3
M1
M2
M3

OVA_365

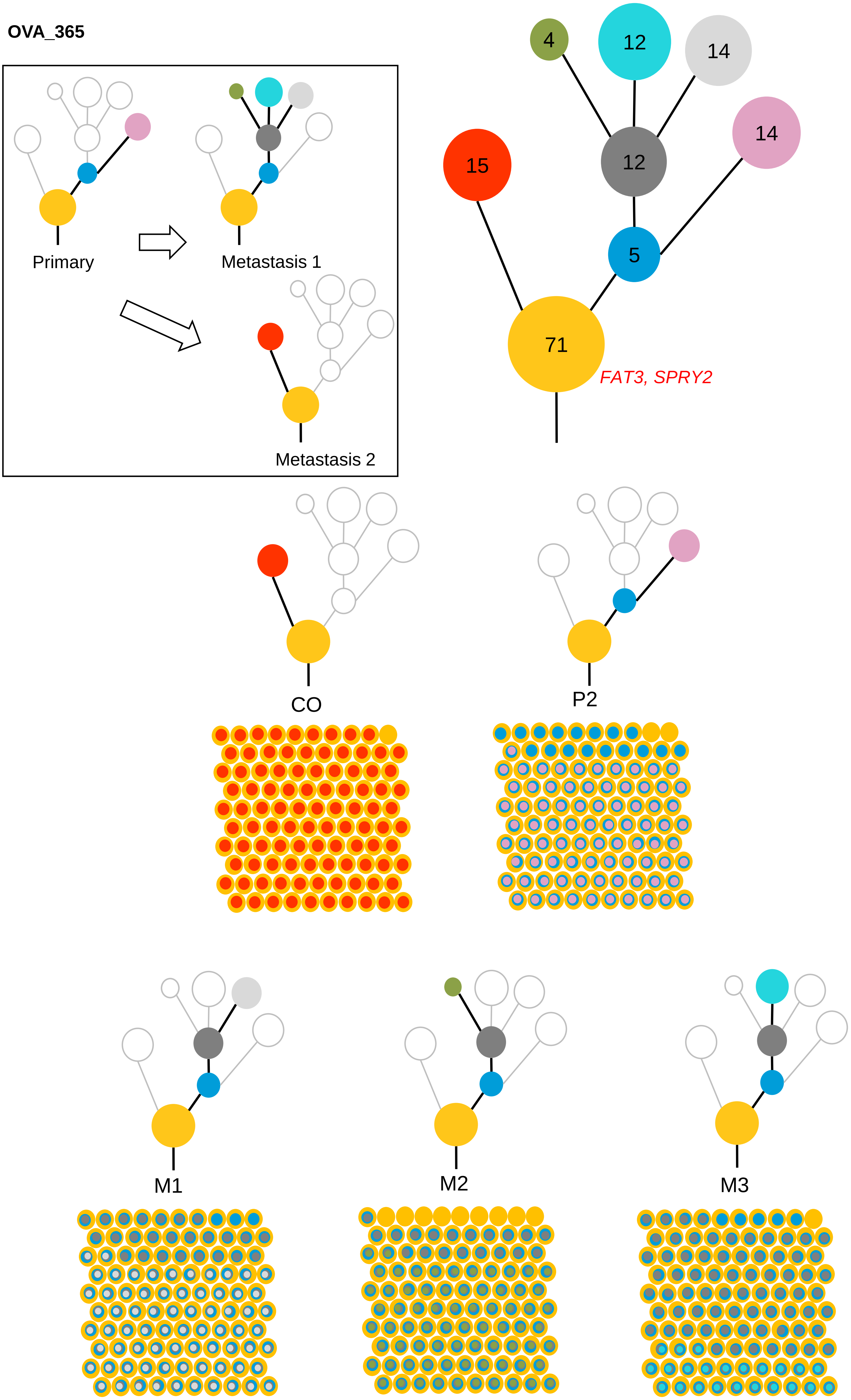
Primary
Metastasis 1
Metastasis 2
4
12
14
15
12
14
5
71
FAT3, SPRY2
CO
P2
M1
M2
M3

OVA_378

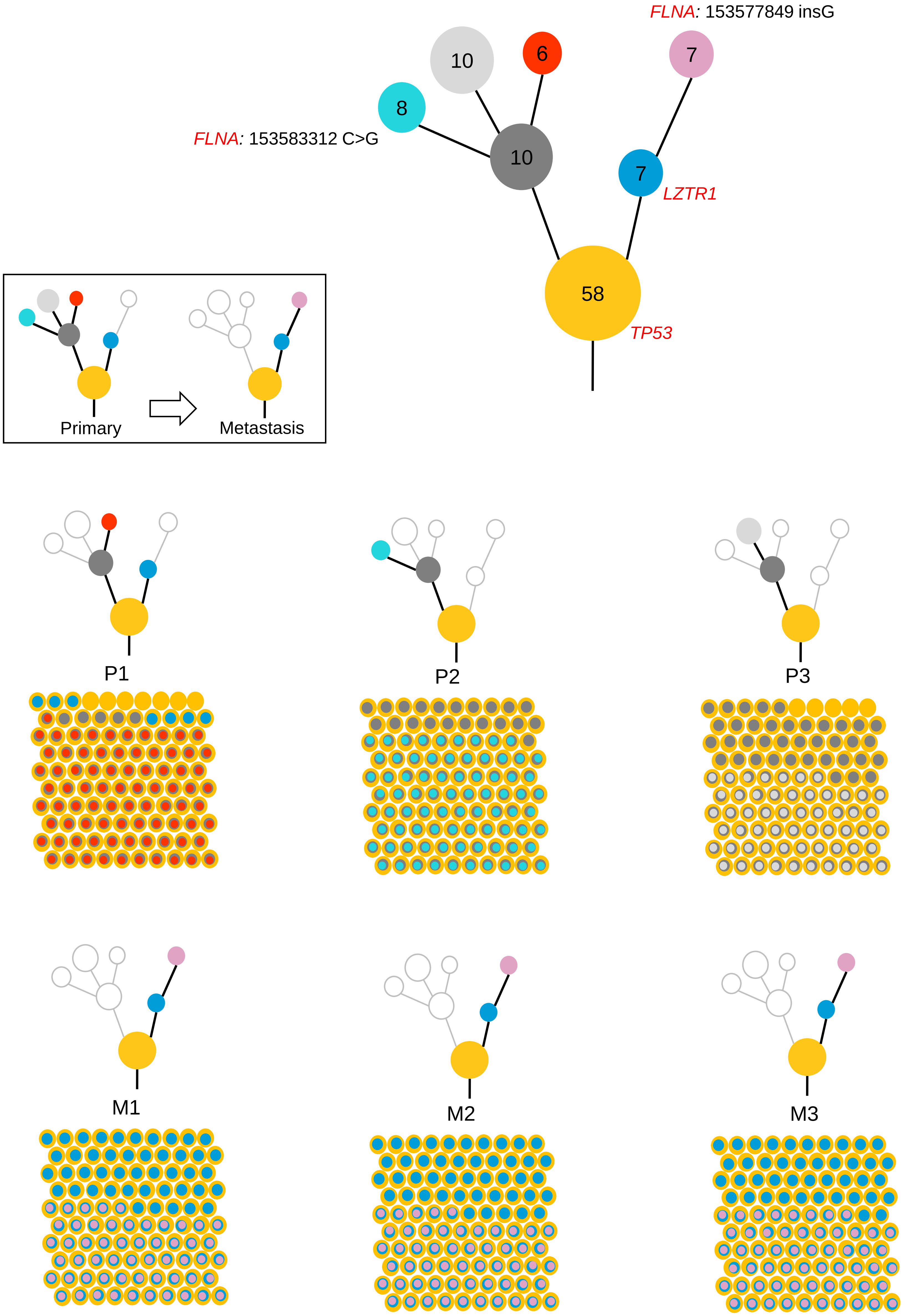

FLNA: 153577849 insG
10
6
7
8
FLNA: 153583312 C>G
10
7
LZTR1
58
TP53
Primary
Metastasis
P1
P2
P3
M1
M2
M3

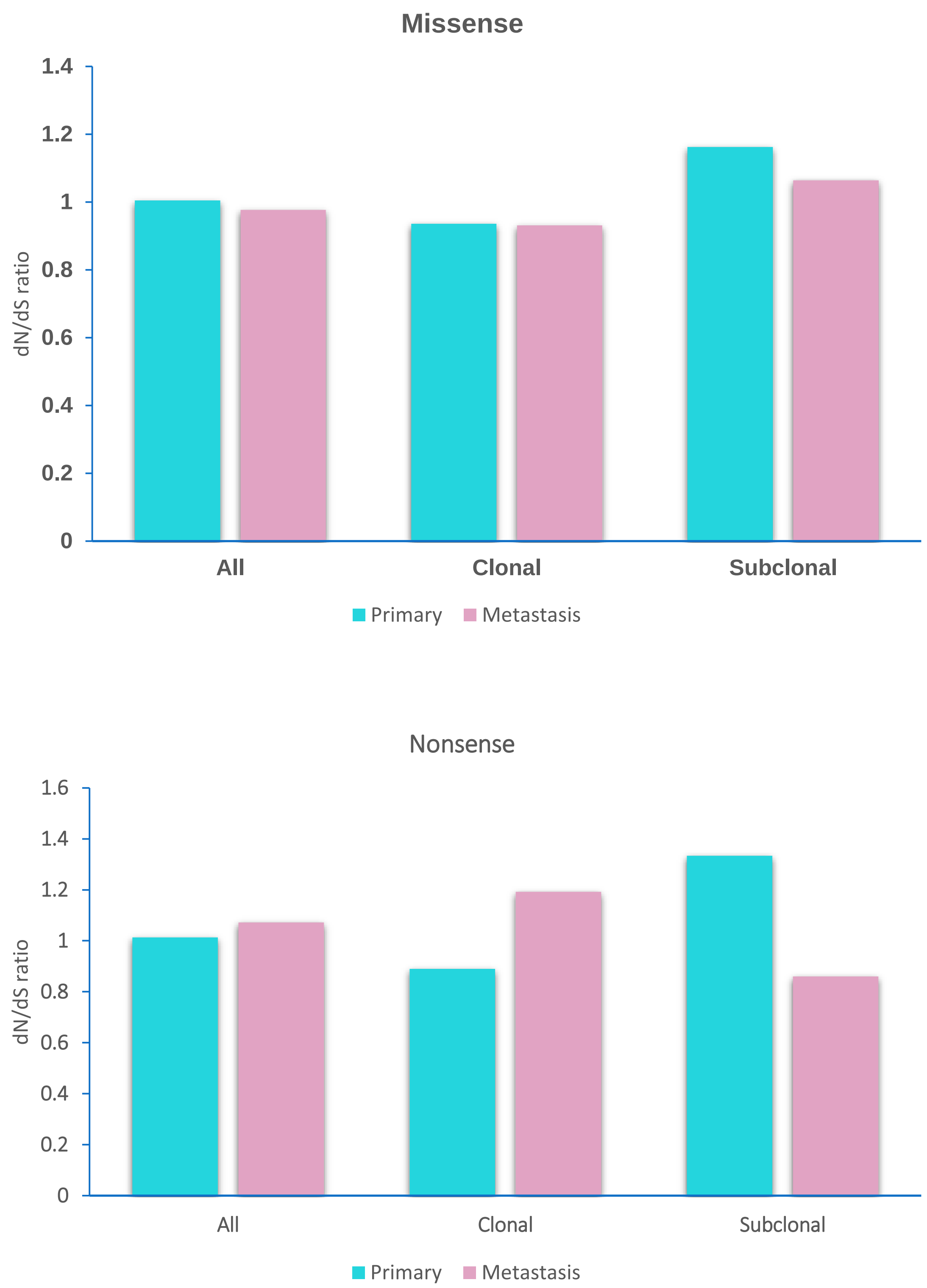

Supplementary Fig. S3: dN/dS ratio

Plot showing dN/dS ratio for overall and temporally dissected (clonal and subclonal) mutations, calculated as Missense and Nonsense mutations, for metastatic HGSOC cohort.

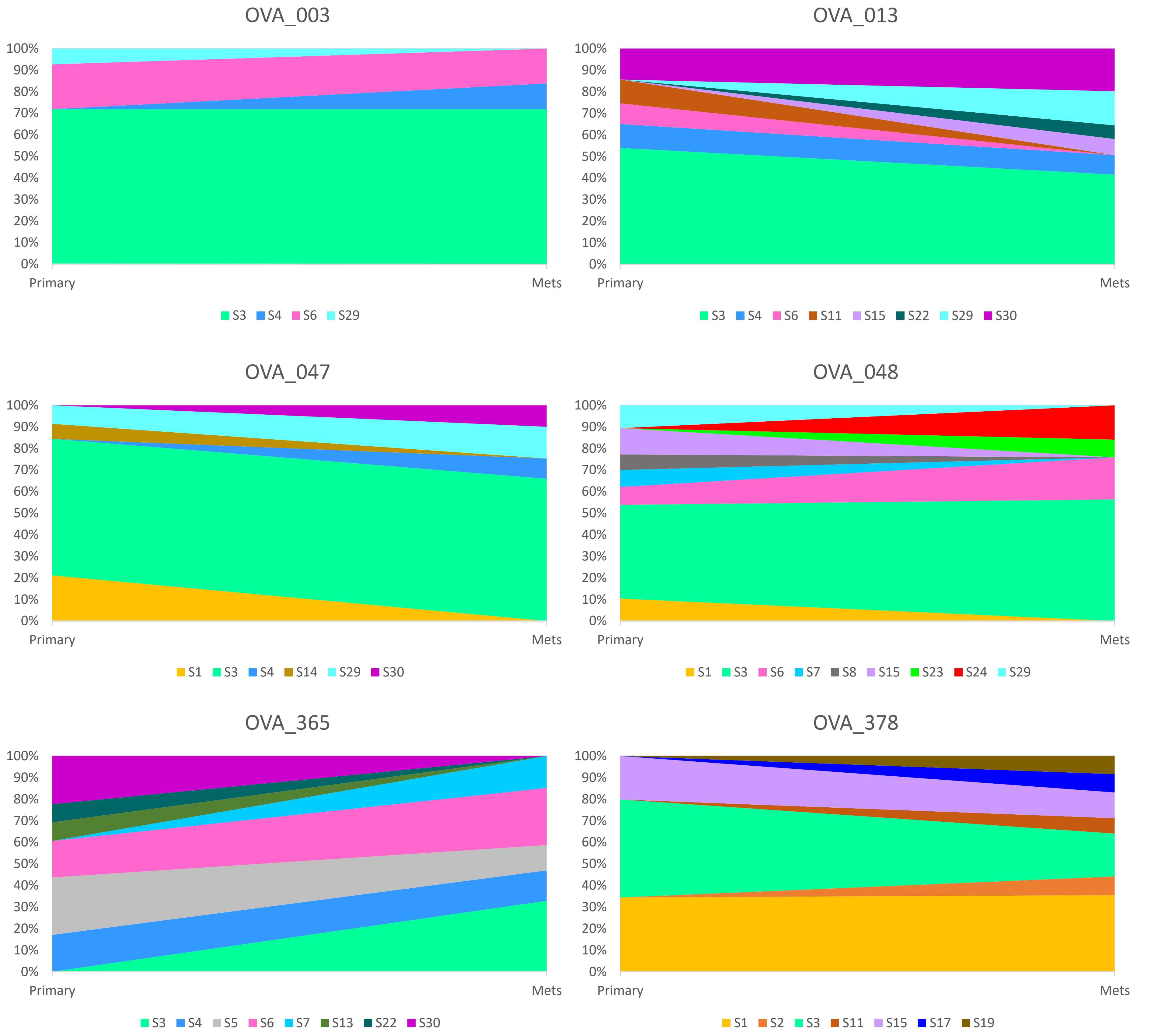

Supplementary Fig. S4: Overall signature shifts.
Overall signature shifts between primary and metastasis. Individual legends have been included with each sample. “Primary” represents primary tumour whereas “Mets” represents metastatic tumour.

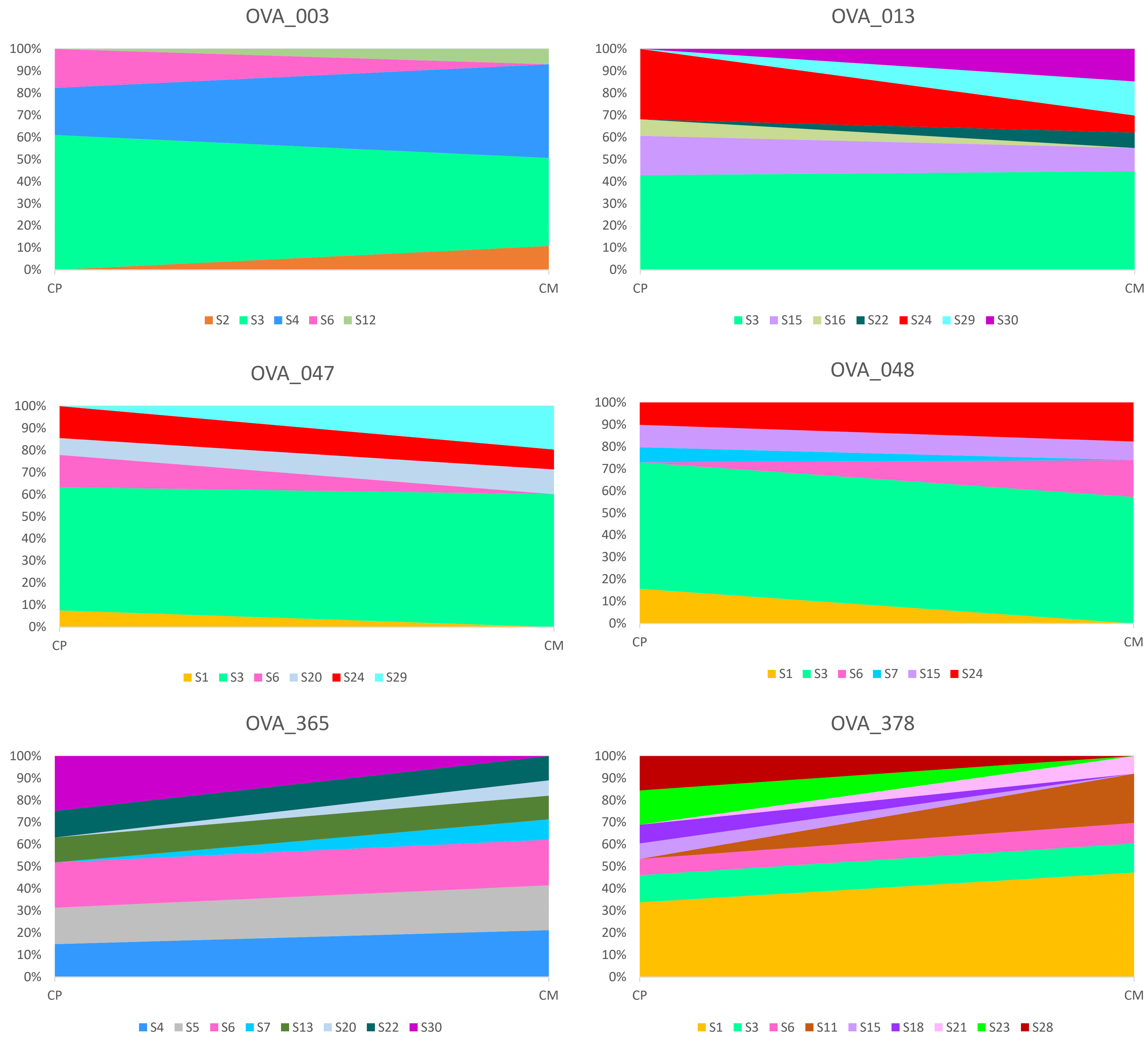

Supplementary Fig. S5: Clonal signature shifts.
Clonal signature shifts between primary and metastasis. Individual legends have been included with each sample. “CP” represents clonal mutations in the primary tumour, whereas “CM” represents clonal mutations in the metastatic tumour.

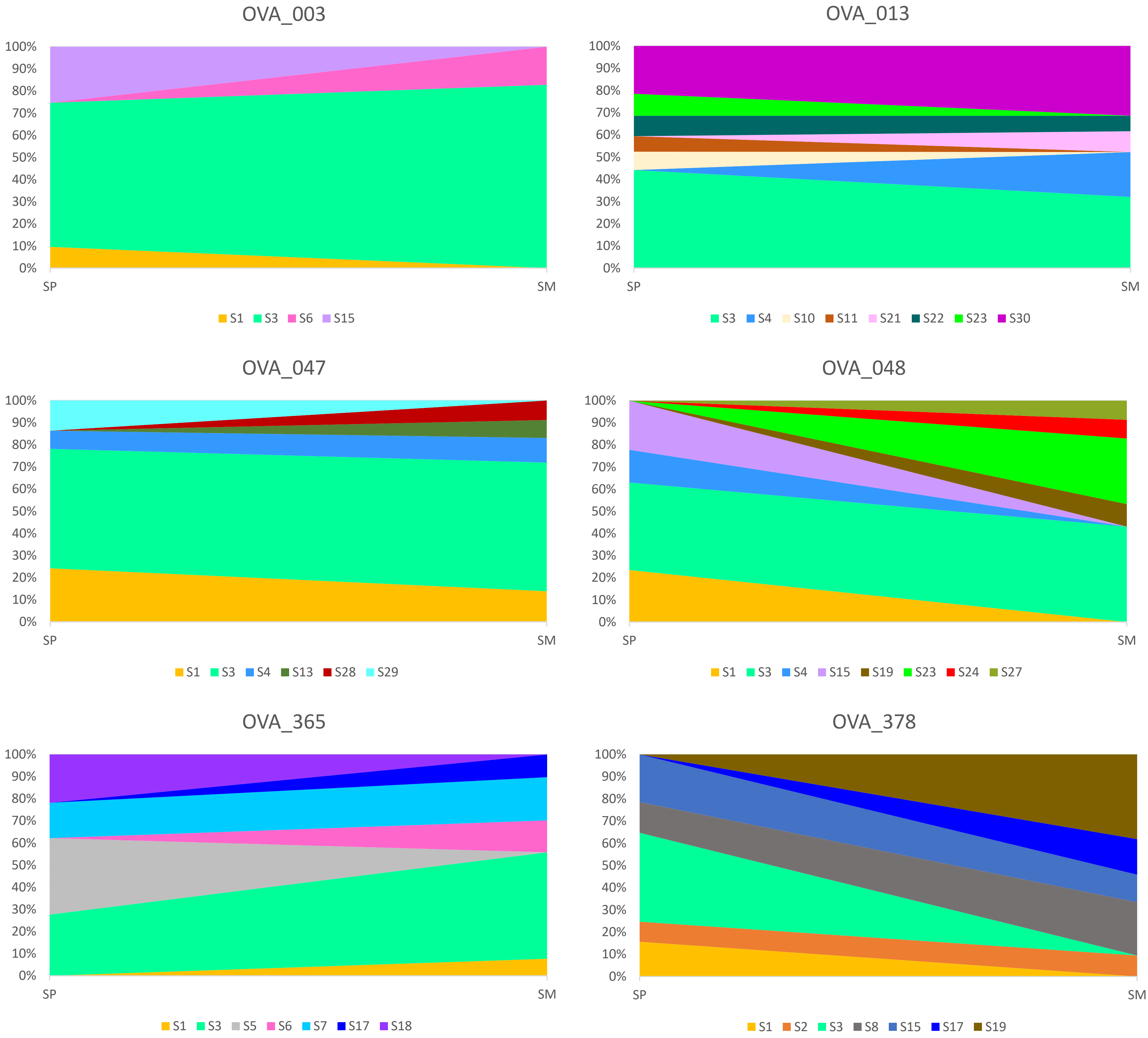

Supplementary Fig. S6: Subclonal signature shifts.
Subclonal signature shifts between primary and metastasis. Individual legends have been included with each sample. “SP” represents subclonal mutations in the primary tumour, whereas “SM” represents subclonal mutations in the metastatic tumour.
